# Arterial endothelial methylome: differential DNA methylation in athero-susceptible disturbed flow regions *in vivo*

**DOI:** 10.1186/s12864-015-1656-4

**Published:** 2015-07-07

**Authors:** Yi-Zhou Jiang, Elisabetta Manduchi, Christian J. Stoeckert, Peter F. Davies

**Affiliations:** Department of Pathology & Laboratory Medicine and Institute for Medicine & Engineering, Perelman School of Medicine, University of Pennsylvania, 1010 Vagelos Building, 3340 Smith Walk, Philadelphia, PA 19104 USA; Department of Genetics and Institute for Biomedical Informatics, Perelman School of Medicine, University of Pennsylvania, Philadelphia, PA 19104 USA

**Keywords:** Endothelium, DNA Methylation, Epigenetics, Hemodynamics, Disturbed Flow, HOX Genes, Atherosclerosis, Endothelial Gene Transcription

## Abstract

**Background:**

Atherosclerosis is a heterogeneously distributed disease of arteries in which the endothelium plays an important central role. Spatial transcriptome profiling of endothelium in pre-lesional arteries has demonstrated differential phenotypes primed for athero-susceptibility at hemodynamic sites associated with disturbed blood flow. DNA methylation is a powerful epigenetic regulator of endothelial transcription recently associated with flow characteristics. We investigated differential DNA methylation in flow region-specific aortic endothelial cells *in vivo* in adult domestic male and female swine.

**Results:**

Genome-wide DNA methylation was profiled in endothelial cells (EC) isolated from two robust locations of differing patho-susceptibility: − an athero-susceptible site located at the inner curvature of the aortic arch (AA) and an athero-protected region in the descending thoracic (DT) aorta. Complete methylated DNA immunoprecipitation sequencing (MeDIP-seq) identified over 5500 endothelial differentially methylated regions (DMRs). DMR density was significantly enriched in exons and 5’UTR sequences of annotated genes, 60 of which are linked to cardiovascular disease. The set of DMR-associated genes was enriched in transcriptional regulation, pattern specification HOX loci, oxidative stress and the ER stress adaptive pathway, all categories linked to athero-susceptible endothelium. Examination of the relationship between DMR and mRNA in HOXA genes demonstrated a significant inverse relationship between CpG island promoter methylation and gene expression. Methylation-specific PCR (MSP) confirmed differential CpG methylation of HOXA genes, the ER stress gene ATF4, inflammatory regulator microRNA-10a and ARHGAP25 that encodes a negative regulator of Rho GTPases involved in cytoskeleton remodeling. Gender-specific DMRs associated with ciliogenesis that may be linked to defects in cilia development were also identified in AA DMRs.

**Conclusions:**

An endothelial methylome analysis identifies epigenetic DMR characteristics associated with transcriptional regulation in regions of atherosusceptibility in swine aorta *in vivo*. The data represent the first methylome blueprint for spatio-temporal analyses of lesion susceptibility predisposing to endothelial dysfunction in complex flow environments *in vivo*.

**Electronic supplementary material:**

The online version of this article (doi:10.1186/s12864-015-1656-4) contains supplementary material, which is available to authorized users.

## Background

Atherosclerosis initiates as a focal disease resulting from complex gene-environment interactions [1]. Although associated with systemic risk factors that act throughout the arterial circulation, the distribution of lesions and pre-lesional susceptibility is localized and predictive. Lesions preferentially originate and develop in the subendothelial arterial intima at branches, inner curvatures, and bifurcations dominated by complex hemodynamic conditions collectively referred to as disturbed flow [2, 3]. The endothelium is the major cellular interface where local flow-related biomechanical and transport characteristics are linked to the initiation of atherosclerosis^2^. Pro-inflammatory endothelial phenotypes are characteristic of atherosusceptible, disturbed flow regions [4]. Such sites express enhanced endoplasmic reticulum (ER) stress/unfolded protein responses (UPR) [5–9] and accumulate pro-atherogenic reactive oxygen species (ROS) [7]. A hierarchy of flow-sensitive transcriptional, post-transcriptional and translational mechanisms regulate regional arterial endothelial phenotype [10, 11]. Following prescient early demonstrations of flow effects upon chromatin remodeling [12] and histone code [13], other epigenetic mechanisms - microRNAs [14–17] and DNA methyltransferases [18–20] - have recently been added to the regulation of flow sensitive endothelial phenotype.

DNA methylation, a potent mechanism of transcriptional silencing [21], is critical for the organization of chromatin and the regulation of tissue-specific gene expression [22]. In mammals DNA methylation is primarily localized to carbon 5 of cytosine residues of CpG dinucleotides. DNA (cytosine-5-)-methyltransferases (DNMT) establish DNA methylation patterns during development and maintain tissue-specific DNA methylation during cell division [22]. Methylation by DNMTs is counterbalanced by passive and/or active DNA demethylation in which the tet methylcytosine dioxygenase (TET) pathway has been suggested to play a central role [23]. CpG islands (CGI), which contain dense CpG dinucleotides, are found within the promoters of ~70 % of mammalian genes [22, 23]. Promoter-associated CGI tend to be unmethylated, but a subset are methylated or partially methylated in differentiated tissues resulting in transcriptional repression of the adjacent genes. In contrast, gene body methylation regulates alternative promoter selection and enhances transcription efficiency and may be more closely linked to tissue-specific functions [24–27]. Changes in DNA methylation patterns have been linked with oxidative stress resulting in dysregulation of gene expression in various disease states [28]. We have recently reported induced CpG methylation of endothelial Kruppel -like Factor 4 (KLF4) promoter in response to disturbed flow *in vitro* that resulted in DNMT3A-mediated hypermethylation and inhibition of KLF4 transcription [19, 29]. Hypermethylation of mechanosensitive endothelial genes following surgical intervention to replace undisturbed flow with disturbed flow in mouse common carotid artery [20] and the induction of DNMT1 by disturbed flow in vitro [18, 20] is further evidence of flow-related plasticity of endothelial DNA methylation.

**Fig. 1:**
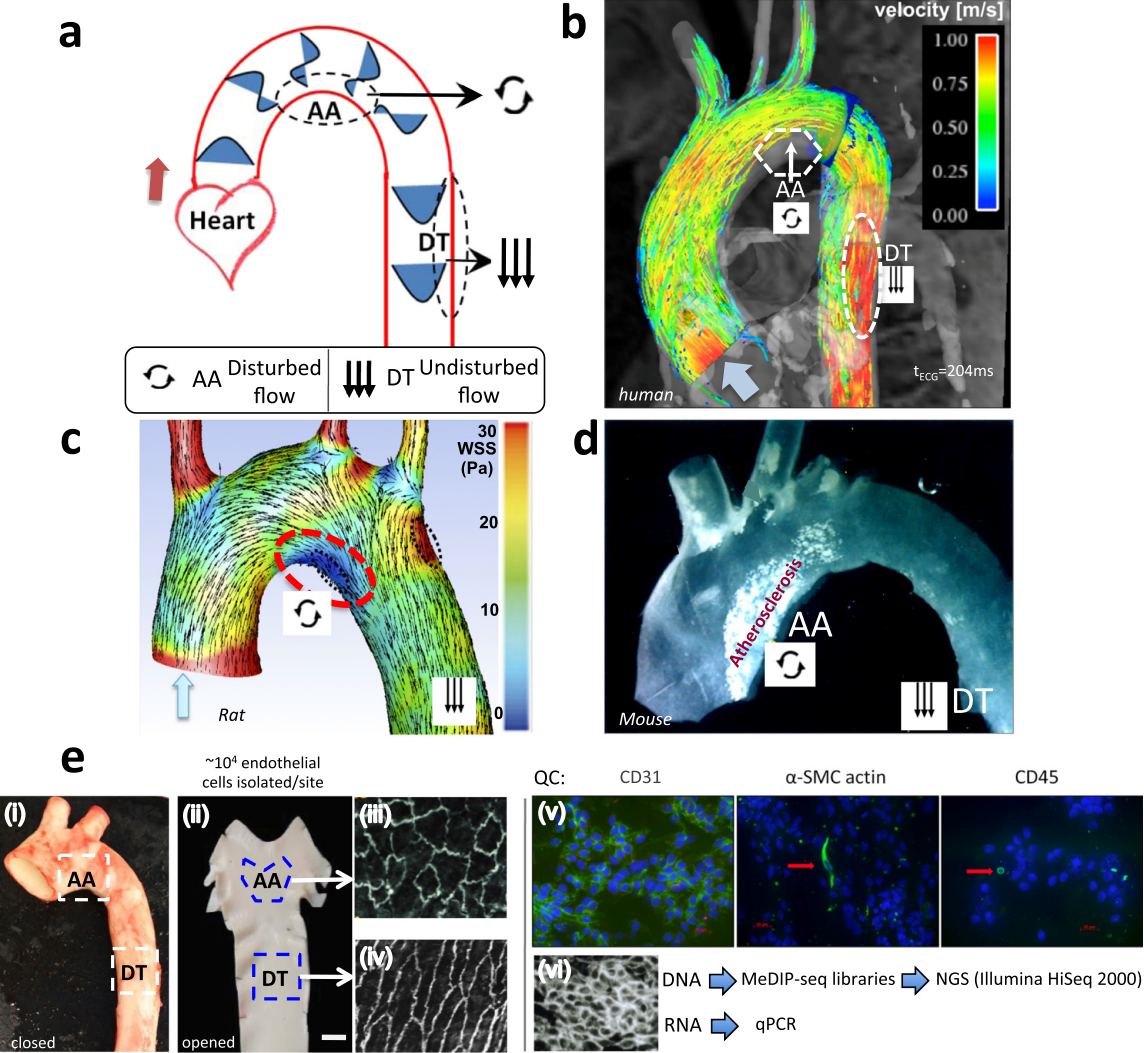
Site-specific disturbed blood flow in the aorta. Flow separation in the pig aortic arch (AA) defines an athero-susceptible site characterized by disturbed blood flow and oxidative stress. **a**: Flow velocity vectors in the aorta illustrating flow separation with reversal in the inner curvature of the AA during systole. Unidirectional flow recovers in the athero-protected descending thoracic (DT) segment. **b**: MRI-generated computational fluid dynamics of human systolic aortic blood flow. Low net flow in the AA separated flow region is below system detection. Unidirectional pulsatile laminar flow dominates all other regions. (From Markl et al. 2011) [61], reproduced with permission. **c**: Computed wall shear stress (WSS) and velocity distributions during systole in rat aorta. (Adapted from Bjorck et al. 2012) [62] with open access permission to reproduce. **d**: Aortic arch of apoE^−/−^ mouse fed a high fat high cholesterol diet for 6 weeks, illustrating fatty streak atherosclerosis at the inner curvature of the AA. From Cheng et al. [63] reprinted with permission. **e**. Pig AA and DT endothelial harvest sites (i and ii) with their respective endothelial cell morphologies (iii and iv). An occasional smooth muscle cell or leukocyte was identified within the harvested endothelium (v). Endothelial nucleic acids were isolated for methylated DNA immunoprecipitation sequencing (MeDIP-seq) and for qRT-PCR (vi)

To investigate the prevalence of flow-related site-specific DMRs in a physiological arterial setting, we mapped the DNA methylation landscape in swine endothelial cells from the inner curvature of the aortic arch (AA; disturbed flow) and from the nearby descending thoracic aorta (DT; undisturbed flow) representing athero-susceptible and athero-protected sites respectively. Figure 1 illustrates the regional flow differences (Fig. 1a,b) and wall shear stresses (Fig. 1c) in the AA and DT and the predisposition of AA to atherosclerosis (Fig. 1d).

## Results

Alterations in global DNA methylation are a hallmark of embryonic development and cell identity and are frequently associated with mutations during aging and cancer [22, 23]; however they have not been investigated in regions of athero-susceptibility where the endothelium is chronically stressed by disturbed flow hemodynamics but otherwise physiologically normal.

### Characteristics of endothelial methylome by MeDIP-seq

The genome-wide DNA methylation pattern in endothelial cells was investigated in detail by MeDIP-sequencing [30]. Twenty-four MeDIP libraries from AA and DT of 6 male and 6 female swine were sequenced. 19 ± 0.38 million uniquely aligned 50 bp reads per sample library were generated. MeDIP-seq experiment annotation and data were uploaded into ArrayExpress (https://www.ebi.ac.uk/arrayexpress/) with accession number E-MTAB-1930 and into the European Nucleotide Archive (http://www.ebi.ac.uk/ena/) with accession ERP004025.

The average methylation density over all 24 samples showed a decline in DNA methylation near transcription start sites (TSS), followed by a sharp rise and steadily increasing methylation in the gene body region which ended in a sharp decrease at transcription termination sites (TTS) to a lower plateau (Additional file 1: Figure S1a). The exons in general tended to be methylated as compared to the introns (Additional file 1: Figure S1b). Similar genome-wide methylation patterns have been reported in various tissues of other species such as rat and chicken [31, 32].

### Identification of site-specific differential methylated regions (DMRs)

**Fig. 2:**
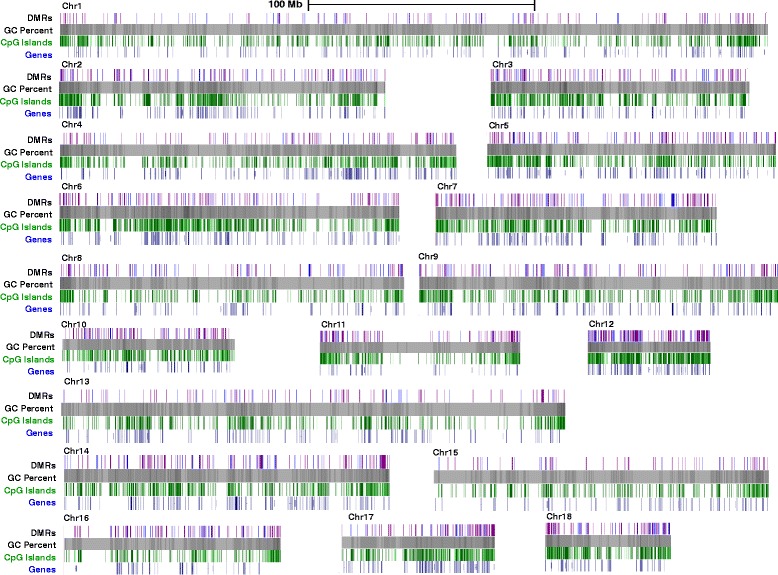
Athero-susceptible DMRs and genome features. UCSC Genome Browser (http://genome.ucsc.edu) was used to show athero-susceptible DMRs in each chromosome. DMRs (AA vs. DT) are shown as vertical lines which represent AA hypermethylation (*blue*) or AA hypomethylation (*purple*) respectively (*n* = 24 sequence libraries, FDR < 0.1). GC percent (*grey*); CpG islands (*green*); genes (*dark blue*). Bar = 100 Mb

The BALM algorithm identified an average of 0.78 million methylation-enriched regions (MRs) per sample averaging 240 bp in length. By comparing the methylation-enriched regions of AA and DT, a total of 5517 differential methylated regions (DMRs) were identified in somatic chromosomes (chr) with an average length of 804 ± 45 bp and consisting of about 0.2 % of the genome (Fig. 2 and Additional file 2: Table S1a, *n* = 12, FDR < 0.1). 4019 regions were hypomethylated while 1498 regions were hypermethylated in AA relative to DT. Analyses of DMRs in chrX were restricted to male samples because changes in DNA methylation are accompanied by chrX inactivation and the methylation level is different between the two chrX in females [33]. Three DMRs were identified on chrX between AA and DT (Additional file 2: Table S1a, *n* = 6, FDR < 0.1). No DMRs were found in chrY.

### Genome-wide distribution of athero-susceptible MRs and DMRs

**Fig. 3:**
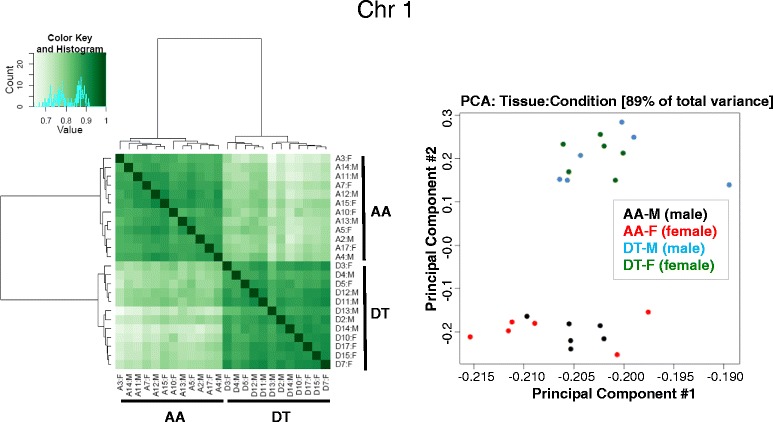
Correlation heatmap and Principal Component Analysis (PCA) distinguishes site-specific DMRs in both male (M) and female (F) tissues. Correlation heatmap and PCA plot (generated with the R bioconductor package DiffBind) based on the DMRs between AA and DT are illustrated for chromosome 1. Similar heatmaps and plots for all 18 somatic chromosomes are shown in Additional file 1: Figure S2

The macroscopic display of global athero-susceptible DMRs in each chromosome showed that DMR-rich regions predominantly overlapped with gene- and CGI-rich regions, in contrast to gene- and CGI-poor regions (Fig. 2). Correlation heatmaps and Principal Component Analyses (PCA) plots based on the DMRs confirmed that the DMR clearly distinguish atherosusceptible AA from atheroresistant DT in tissue from both genders. Figure 3 shows these for chromosome 1; similar patterns for all 18 somatic chromosomes are shown in Additional file 1: Figure S2.

**Fig. 4:**
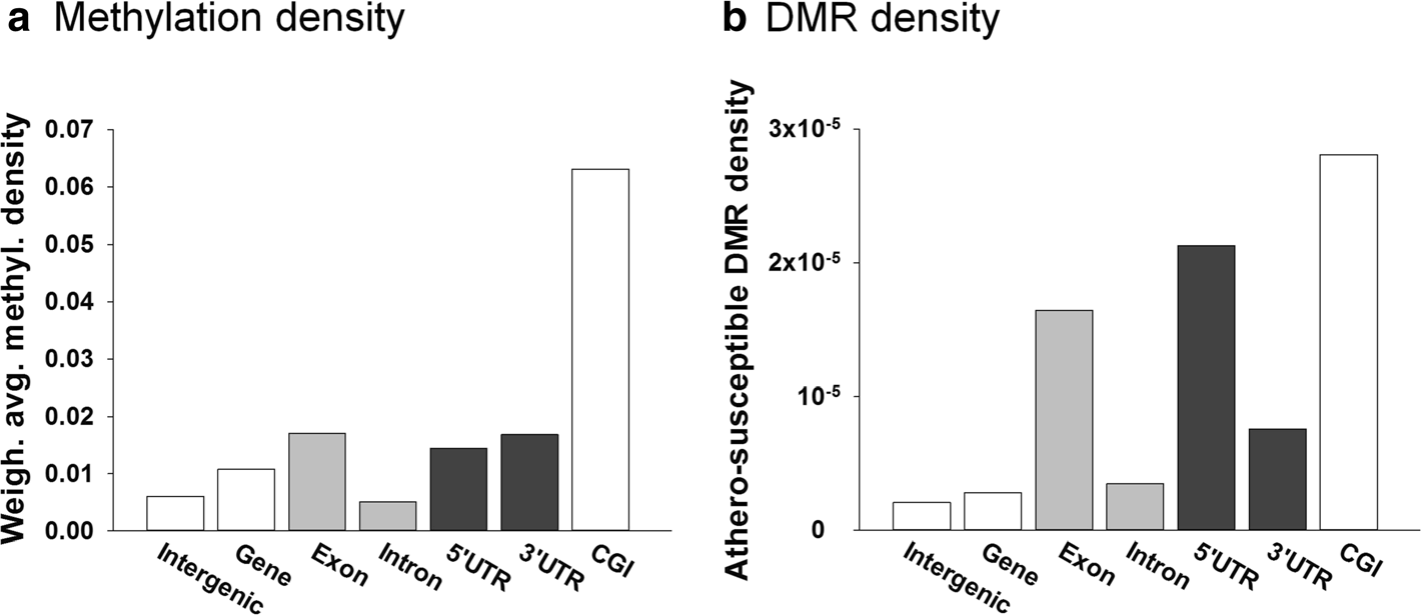
Methylation and Athero-susceptible DMR distributions in genomic features. **a**: DNA methylation and **b**: DMR density in genomic features. For each genomic feature (intergenic, gene, etc.), DNA methylation or DMR density was computed as the number of reads or DMRs overlapping the portion of the genome covered by that feature type divided by the size of such portion of the genome. In this figure, the flanked gene was defined as the region from 10 kb upstream of the transcription start site to the end of the transcript. Intergenic regions were defined as the part of the genome not contained in that region. As gene models we used ENSEMBL 71 for Sscrofa10.2 (30,586 transcripts). *n* = 12 animals, 24 MeDIP seq libraries

To characterize the relation of athero-susceptible DMRs to genomic features, we analyzed the density of DNA methylation and DMRs in functional genomic elements. As gene models we used ENSEMBL GENES 71 for Sscrofa10.2 (30,586 transcripts). Dot-blot data showed that the global methylation level is not significantly different between AA and DT (Additional file 1: Figure S3); however the distributions to features within each site (AA, DT) were significantly different. Consistent with Fig. 2, DNA methylation and DMRs were mostly enriched in the CGIs. Although methylation regions (MRs) density was comparable between 5’ and 3’UTR (Fig. 4a), athero-susceptible DMR density was 2.8-fold higher in the 5’UTR than in the 3’UTR (Fig. 4b). The enrichment of DMRs in the 5’UTR suggests a functional role of DMRs in contributing to the transcriptional activity of genes. Higher methylation and DMR densities were also noted in exons (Fig. 4a,b); 3.3-fold and 4.7-fold higher than in introns for methylation and DMR densities respectively. Numerous studies have shown that DNA methylation in the promoter, gene body and exons regulates transcription by promoter suppression, transcription elongation efficiency, alternative RNA splicing and exon recognition [22, 26, 27]. Thus, these data suggest that athero-susceptible DMRs functionally associate with gene regulation at the level of the 5’ UTR and individual gene/exons, consistent with a contribution to the heterogeneity of endothelia in diverse chemical and physical environments.

### Functional enrichment analysis for athero-susceptible DMRs

**Fig. 5:**
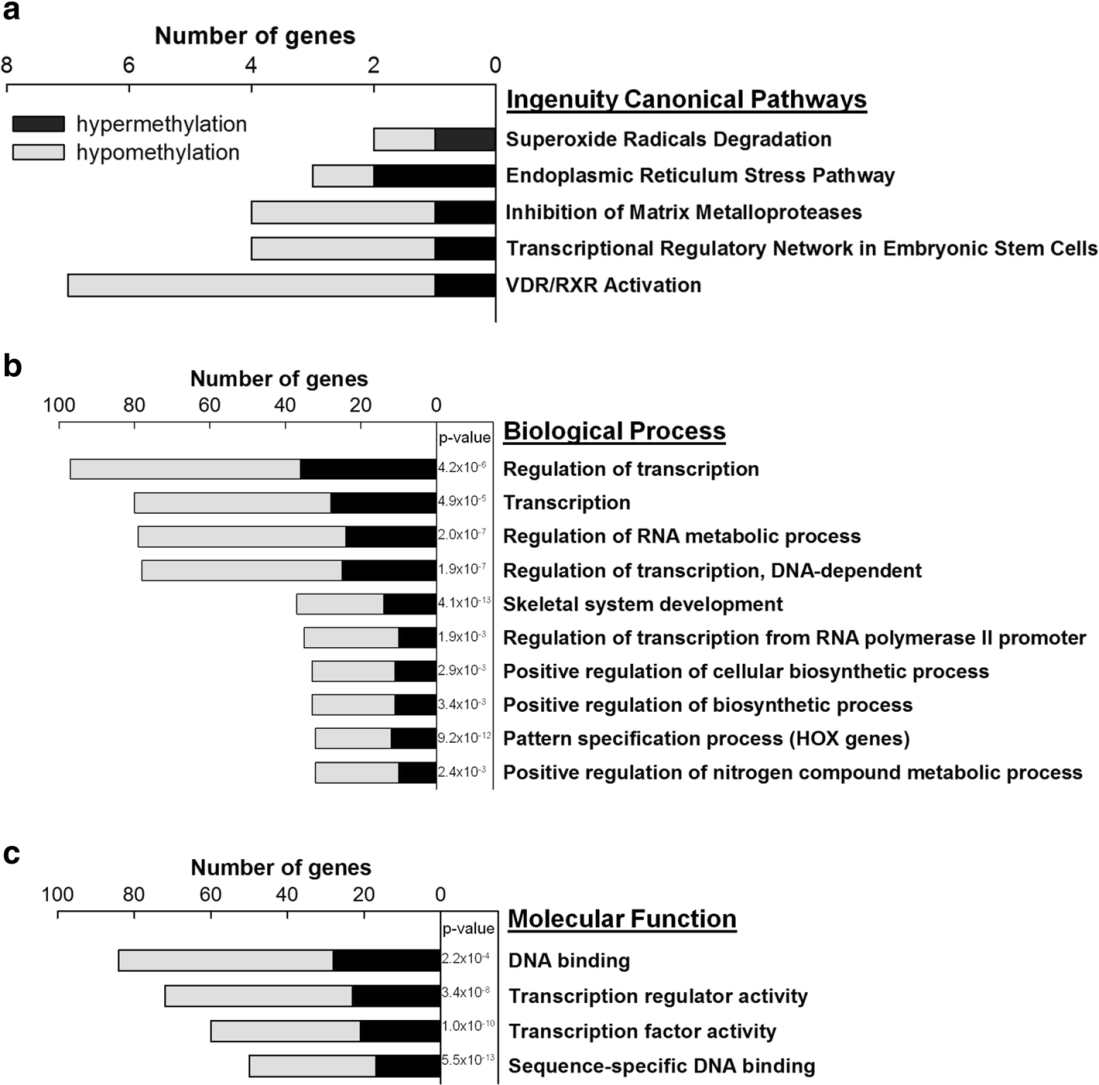
Functional annotations of genes associated with athero-susceptible DMRs. **a**: Ingenuity Pathway Analysis of DMR-associated genes. The top five pathways and associated genes are listed. **b**: Gene ontology (GO) Biological Processes and **c**: Molecular Function categories enriched for DMR-associated genes. The enrichment analysis was performed using online tool DAVID (the Database for Annotation, Visualization and Integrated Discovery). The p-value for each GO term was adjusted by using Benjamini-Hochberg for multiple-testing correction. In **b** and **c**, categories are ranked according to the number of genes containing hypermethylated (*black bar*) and hypomethylated (*grey bar*) DMRs in AA

Additional file 2: Table S1a details athero-susceptible DMRs, including genomic coordinates, length, distance to the nearest transcripts and methylation fold change. We found 632 pig Ensembl genes that contain at least one DMR in the proximal promoter region (2 kb upstream to 1 kb downstream TSS) or distal regulatory region (up to 10kb upstream) as shown in Additional file 3: Table S1b. Of these pig genes, 431 have human homologs. 60 DMR-associated genes were found by Ingenuity Pathway Analysis (IPA) to be linked to cardiovascular disease in the top 5 Disease and Dysfunction category (Table 1). Previously we reported that chronic low level activation of ER stress, unfolded protein response and oxidative stress are associated with athero-susceptible endothelium [6, 7, 10]. Genes related to superoxide radical degradation and ER stress were over-represented among those containing athero- susceptible DMRs (Fig. 5a) supporting a role for DMRs in ROS and ER mechanisms in prelesional AA regions. Athero-susceptible DMRs were also associated with genes of the matrix metalloproteinase pathway and vitamin D receptor activation pathway, both of which have been shown to play critical roles in cardiovascular disease [34, 35].

**Table 1:** DMR-associated genes linked to cardiovascular disease

Hypermethylation and Hypomethylation in Atherosusceptible Endothelium *in vivo*	
*Hyper*methylation	
Gene Symbol	Gene name
AGT	Angiotensinogen (serpin peptidase inhibitor, clade A, member 8)
ASIC1	Acid-sensing (proton-gated) ion channel 1
BGLAP	Bone gamma-carboxyglutamate (gla) protein; polyamine-modulated factor 1
CA3	Carbonic anhydrase III, muscle specific
CCM2	Cerebral cavernous malformation 2
COL28A1	Collagen, type XXVIII, alpha 1
DICER1	Dicer 1, ribonuclease type III
EGLN3	egl nine homolog 3 (C. elegans)
ERN1	Endoplasmic reticulum to nucleus signaling 1
HAND2	Heart and neural crest derivatives expressed 2
HOXA4	Homeobox A4
HOXA5^#^	Homeobox A5 [20]
ISL1	ISL LIM homeobox 1
ITGA5	Integrin, alpha 5 (fibronectin receptor, alpha polypeptide)
LCP2	Lymphocyte cytosolic protein 2 (SH2 domain containing leukocyte protein of 76 kDa)
mir-10a	microRNA 10a
MMP9	Matrix metallopeptidase 9 (gelatinase B, 92 kDa gelatinase, 92 kDa type IV collagenase)
PITX2	Paired-like homeodomain 2
SGCB	Sarcoglycan, beta (43 kDa dystrophin-associated glycoprotein)
SLC25A4	Solute carrier family 25 (mitochondrial carrier; adenine nucleotide translocator), member 4
SOD2	Superoxide dismutase 2, mitochondrial
SOX9	SRY (sex determining region Y)-box 9
TBX20	T-box 20
VSNL1	Visinin-like 1
*Hypo*methylation	
Gene Symbol	Gene Name
ADRA2A	Adrenergic, alpha-2A-, receptor
ALS	Superoxide dismutase 1, soluble
APLN	Apelin
BMP7	Bone morphogenetic protein 7
CACNA1E	Calcium channel, voltage-dependent, R type, alpha 1E subunit
CHRNA2	Cholinergic receptor, nicotinic, alpha 2 (neuronal)
EPHX2	Epoxide hydrolase 2, cytoplasmic
EPO	Erythropoietin
FOXO1	Forkhead box O1
GNAS	GNAS complex locus
HOPX	HOP homeobox
HOXA3	Homeobox A3
HTR1B	5-hydroxytryptamine (serotonin) receptor 1B
IGF1R	Insulin-like growth factor 1 receptor
IL4	Interleukin 4
IL8	Interleukin 8
IMPDH1	IMP (inosine monophosphate) dehydrogenase 1
ITGA2B	Integrin, alpha 2b (platelet glycoprotein IIb of IIb/IIIa complex, antigen CD41)
KLF4	Kruppel-like factor 4 (gut)
LIF	Leukemia inhibitory factor (cholinergic differentiation factor)
LIMK1	LIM domain kinase 1
LIMS1	LIM and senescent cell antigen-like domains 1
LTB4R	leukotriene B4 receptor
mir-199a-1	microRNA 199a1
mir-214	microRNA 214
MMP11	Matrix metallopeptidase 11 (stromelysin 3)
NAALADL2	N-acetylated alpha-linked acidic dipeptidase-like 2
NGFR	nerve growth factor receptor (TNFR superfamily, member 16)
PDZD2	PDZ domain containing 2
PLEKHB1	Pleckstrin homology domain containing, family B (evectins) member 1
PPARD	Peroxisome proliferator-activated receptor delta
RETN	Resistin
RORC	RAR-related orphan receptor C
SERPINA5	Serpin peptidase inhibitor, clade A (alpha-1 antiproteinase, antitrypsin), member 5
THBS2	Thrombospondin 2
TNS3	Tensin 3
TTR	Transthyretin

^#^NOTE: The cardiovascular function of HOXA5 was recently discovered [20] and manually added to this table

Gene ontology (GO) annotations of DMR-associated genes indicated significant enrichment of genes involved in transcription (Fig. 5b,c). For example, 97 DMR-associated genes, including 23 homeobox (HOX) genes and 8 homeobox-containing genes, were annotated by the GO term ‘Regulation of Transcription’ (adjusted *p* = 4.2×10^−6^). Other biological processes related to transcription were overrepresented (Fig. 5b). DMR-associated genes were also enriched consistently in GO categories of transcription-related Molecular Functions (Fig. 5c) such as sequence-specific DNA binding. These data suggest that DMRs, acting as a driver of the specific gene expression, not only globally regulate gene expression via modification of cis-acting DNA elements [21, 22, 27] but also govern gene transcription through effects on the expression of trans-acting factors.

### Inverse relationship between 5’UTR DMRs and HOX gene expression

**Fig. 6:**
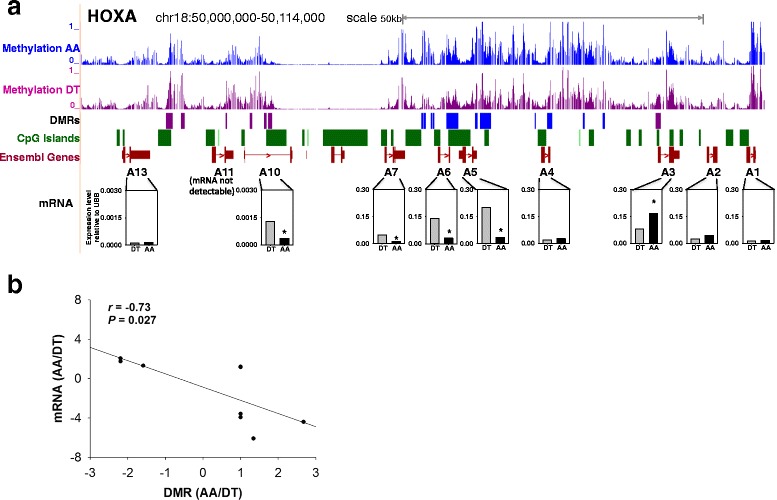
DNA methylation and DMRs in HOXA loci and the relationships to mRNA expression. **a**: The UCSC Genome Browser was used to visualize DNA methylation level of CpG sites in AA (*blue lines*) and DT (purple lines) in HOXA loci (chr18). Bar = 10 kb. 1 = 100 % methylated, 0 = unmethylated. DMRs are shown as blue and purple regions, which represent AA hypermethylation and hypomethylation respectively (*n* = 12 animals, FDR < 0.1). The positions of CpG islands (*green*) and HOX genes (*dark red*) are shown. mRNA expression in DT and AA was measured for HOXA1-A7, HOXA10 AND HOXA13. **b**: Relative (AA/DT) gene expression plotted against relative DMR demonstrating an inverse relationship

The prominence of HOX gene association with DMRs warranted further evaluation of these relationships. The recent identification of HOXA5 methylation in disturbed flow in mouse carotid endothelium^20^ led us to examine HOXA loci in more detail in our large animal model. Figure 6a shows a high resolution map of 18 DMRs clustering at gene- and CGI-rich regions of HOXA loci on chr18. Hypermethylated or hypomethylated DMR regions tended to be contiguous, perhaps associated with higher-order chromatin structure [36]. For example, 11 consecutive DMRs spanning 15 kb in the HOXA4 to HOXA7 locus were hypermethylated in AA.

**Fig. 7:**
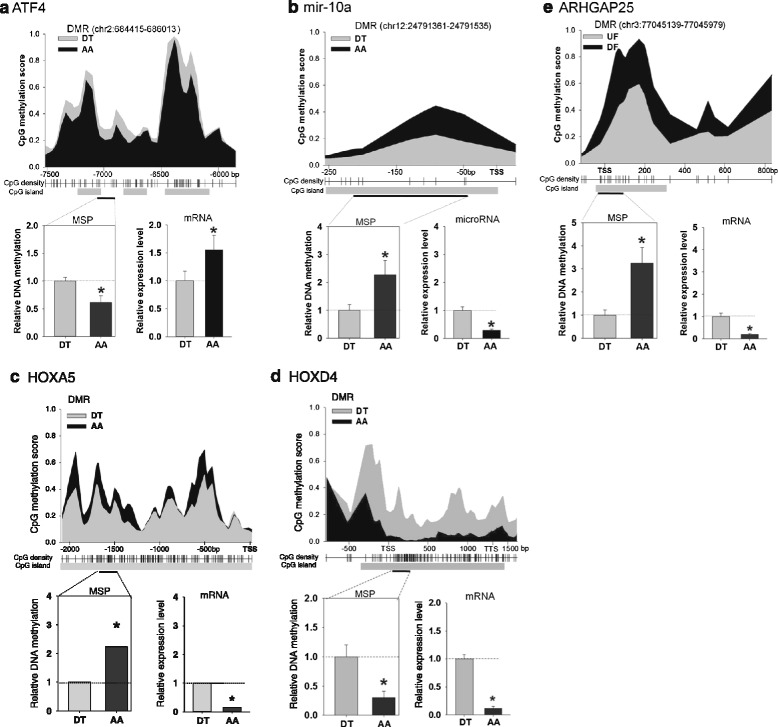
MSP analyses of selected DMRs relative to gene expression in athero-susceptible endothelia. Methylation scores and mRNA levels of **a** ATF4, **b** microRNA-10a, **c** HOXA5, d HOXD4 and **e** ARHGAP25 were measured. The methylation scores of CpG sites were calculated by BALM. 1 = 100 % methylated, 0 = unmethylated. The average methylation scores of AA and DT are shown as black and grey areas respectively (*upper panel*, *n* = 12 animals). Distance to the TSS is shown on the x-axis. Relative methylation levels of regions within the DMRs were analyzed by MSP (*bottom left*). mRNA levels were quantified by qPCR (bottom right). MSP and qPCR data were normalized (See Methods for further details) and are expressed as fold of DT. *indicates significant difference between AA and DT (*n* = 6, *p* < 0.05). chr, chromosome. TSS, transcription start site. TTS, transcription termination site. mRNA data for ATF4 and microRNA-10a were adapted from Civelek et al. [6]. and Fang et al. [14]; reprinted with permission.

Transcript mRNA levels of annotated HOXA genes were measured by qPCR in endothelium from atherosusceptible AA and athero-protected DT sites to determine the relationship between AA/DT transcript expression (mRNA) and AA/DT DMR methylation score for 9 HOXA genes: − HOXA1-A7, HOXA10 and HOXA13 (Fig. 6). HOXA DMRs expressed a significant inverse relationship to transcript expression (Fig. 6b; r = −0.73, *p* = 0.027). AA hypermethylation predicted AA transcript suppression and hypomethylation predicted enhanced mRNA. However an exception to the inverse relationship is HOXA10 which is both hypomethylated in AA and transcriptionally suppressed in AA. This occurs because the DMR is located in the gene body (similar to HOXD4 in Fig. 7d below). Notably, HOXA5 transcription in AA was the most suppressed of the HOXA genes, consistent with its identification in disturbed flow regions of mouse carotid artery [20].

### Validation of selective athero-susceptible DMRs and gene expression

To verify DMRs, methylation specific PCR (MSP) was used to quantify the cytosine methylation in bisulfite-converted DNA from regions encoding several selected genes. We tested the DNA methylation in activating transcription factor 4 (ATF4), microRNA-10a (mir-10a), HOXA5 and HOXD4 and Rho GTPase activating protein 25 (ARHGAP25). These gene promoters contain only one DMR and at least one CGI (Fig. 7). ATF4 is a key transcription factor in regulating cellular response to ER stress and unfolded protein response [6], while mir-10a plays an anti-inflammatory role by modulating NF-κB signaling [14]. ARHGAP25 may be actively involved in tension regulation at endothelial intercellular junctions via rac-1 mediated activation of Rho GTPase [37] and a SNP in ARHGAP25 promoter was reported to be associated with blood pressure [38]. MeDIP-seq and MSP (Fig. 7) confirmed AA hypomethylation of ATF4 (Fig. 7a) and HOXD4 (Fig. 7d) at a distal regulatory site and in the gene body respectively, and AA hypermethylation of mir-10a (Fig. 7b), HOXA5 (Fig. 7c), and ARHGAP25 (Fig. 7e). When mRNA was measured by qPCR, reciprocal relationships with MSP methylation scores were noted in these genes except for HOXD4 (Fig. 7d). ATF4 mRNA was 1.6 fold enhanced in AA, which was inversely associated with its AA promoter methylation (Fig. 7a). Similar inverse mRNA/methylation relationships were noted for mir-10a, HOXA5 and ARHGAP25 (Figs. 7b,c,e). The athero-protective and anti-inflammatory mir-10a was decreased by 71 % in AA, while its promoter was highly methylated in AA (2.3 fold of DT). Although little is known about miRNA transcription, the results suggest that DNA methylation 5’ upstream of pre-microRNA TSS contributes to transcriptional silencing of mature microRNAs. These data are consistent with the inverse relationships predicted by DMR high throughput measurement of regional DMRs. In contrast to DMR in the promoter regions or, in the case of ATF4, the distal regulatory region, DNA methylation in the gene body did not follow a reciprocal relationship with gene expression; for example, the highly methylated gene body of HOXD4 in DT was accompanied by increased HOXD4 mRNA in DT (Fig. 7d). Although the underlying mechanism of this positive association is unclear, gene body methylation is known to enhance gene transcription elongation [24, 25]. We conclude that promoter methylation state contributes to the regulation of gene expression in regions in which consistently disturbed blood flow is prevalent and may function as a mechanism of physiological phenotype adaptability.

### Gender-specific DMRs

In general, males are at higher risk for cardiovascular disease than pre-menopausal females; the differences narrow after menopause. Intensive studies have focused on genetic background and sex hormones in contributing to gender differences in the development of CVD [39]. In this study, male and female MeDIP-seq data in the somatic chromosomes were compared in the same anatomic sites (Additional files 4 and 5: Tables S2a and S2b). In male vs female AA samples, 87 gender-specific DMRs were found. Thirteen Ensembl genes contained at least one DMR located between 10 kb upstream TSS and 10 kb downstream TTS. In DT, 166 gender-specific DMRs were found. Thirty nine Ensembl genes contained at least one DMR located between 10 kb upstream TSS and 10 kb downstream TTS. Even though the number of sex-specific DMR was low, we observed epigenomic evidence which may contribute to the sexual difference in susceptibility to CVD. Arterial endothelial cells with primary cilia are more prevalent in AA where complex hemodynamic forces dominate and the atherosclerosis lesions develop [40]. Our data showed that in the athero-susceptible sites, sex-specific DMRs were associated with the TRAF3IP1 and BBS7 genes that are involved in the assembly of primary cilia. Cilia extend into the flow field acting as a biomechanical signal amplifier; however, the mechanotransduction mechanisms that promote an atherogenic endothelial phenotype are not known. TRAF3IP1 plays a critical role in cilia assembly during development in mouse [41], while BBS7 encodes one of seven proteins that form the BBSome complex that promotes ciliogenesis [42].

## Discussion

The complex geometry of arteries influences local hemodynamic characteristics that greatly influence the susceptibility of specific regions to atherogenesis. Mechanisms that link site-specific flow to atherosusceptibility are (i) biomechanical effects of disturbed vs undisturbed flow that differentially regulate endothelial phenotype through mechanotransduction mechanisms and (ii) local transport differences at the endothelial/blood interface that elicit different signaling signatures during disturbed vs undisturbed flows. These mechanisms are not mutually exclusive. Following identification of pro-inflammatory gene and protein expression in atherosusceptible endothelium in vivo [4], many in vitro studies identified a hierarchy of regulation of gene and protein expression in cultured endothelial cells in response to recapitulated arterial flows (reviews [10, 11]). Interest in regulation of flow-mediated gene expression at the epigenetics/epigenomics level revealed potent post-transcriptional microRNA effects [14–17]. Recently, DNA methylation mechanisms have been shown to be surprisingly responsive to disturbed flow, suppressing endothelial transcription of several important molecules known to be inhibited by disturbed flow [19, 20]. In this paper we returned in vivo to regions of robust disturbed and undisturbed flow in the swine aorta that are well characterized for differential endothelial phenotypes to examine the endothelial methylome and more specifically to identify differential methylated regions (DMRs) associated with promoter CpG dinucleotides of atherosusceptibility genes. A substantial number of new DMR-gene associations were identified in addition to confirmation of DMRs associated with several genes that responded robustly to disturbed flow in vitro (KLFs, HOX genes) [19, 20]. Association of DMR with suppression (hypermethylation) or enhancement (hypomethylation) of transcription in this study is not necessarily indicative of flow-related methylation plasticity. During development many genes, notably of the HOX family, determine cell identity, position and differentiation status by DNA methylation and these relationships may not be responsive to the local post-natal environment; further in vitro flow experiments will resolve such differences. However, our data are the first comparative map of sites where the flow characteristics are chronically different, allowing a first approximation of the site-specific effect of hemodynamic environment on the endothelial methylome in vivo and linking it to cell phenotype.

DNA methylation maps are critical for understanding the molecular basis of complex diseases. Induced DNA methylation changes can accumulate and propagate in cell populations during cell division, proliferation and aging, resulting in permanent maintenance of the acquired pathophysiological phenotype. By translating the effects of environmental stimuli into coordinated gene expression programs for cellular adaptation, epigenomic pathways are mechanistic links between the genome and environment that are important in understanding common cardiovascular diseases. In the current study we used a genome-wide approach to map athero-susceptible endothelium in a large animal model, reflecting conditions that underlie endothelial diversity and susceptibility. Endothelial cells from animals of both genders were randomized from different genetic backgrounds to reflect a general population. The DMR atlas provides a search framework for etiological factors and heritability determinants related to endothelial phenotype susceptibility to atherosclerosis, a spatially-predictable complex disease caused by a combination of genetic and environmental factors.

Previously we identified ER stress and oxidative stress as common features in athero-susceptible regions of both non-coronary and coronary arteries [7]. Several DMR-associated genes were related to ER stress and superoxide radical degradation pathways in the present study. In the arterial system the local physical, chemical and hemodynamic mechanical environment varies spatially to create regions of persistent changes in endothelial gene and protein expression that can promote atherogenesis. ER stress, an adaptive response that corrects excess unfolded protein biosynthesis in the cell, is linked to local accumulation of ROS in susceptible sites [7, 43]. ROS, which are present in regions of hemodynamic complexity, have been shown to be generated in ER-stressed cells *in vitro* and in endothelial cells exposed to disturbed flow [44, 45] linking ROS to blood flow characteristics as a determinant of endothelial phenotype plasticity possibly mediated via mitochondrial oxidative stress [43].

DNA methylation has been suggested to play a role in endothelial differentiation in which the DMRs may be a residue of a molecular ‘fossil record’ during development [21–23]. As noted above, these signatures will be endogenously embedded in the DMRs and may be resistant to change by environmental influences. Since Barker (2004) hypothesized that environmental factors in crucial periods of early life can influence risks for cardiovascular disease later in life [46], animal studies have suggested that diet-induced and fetal-originated cardiovascular disease are related to oxidative stress and anti-oxidant defense systems [47, 48].

HOX transcriptional factors, master regulators of body patterning [49], have been reported to specify positional identities in arterial blood vessels [50, 51]. Therefore they are highly relevant for understanding the mechanisms underlying regional physiological and phenotypic diversities in the cardiovascular system. HOX genes regulate endothelial cell proliferation, migration, differentiation, morphogenesis and permeability during development and vascular remodeling in adults. For example HOXA3/9, HOXB3/5 and HOXD3 regulate endothelial cell activation, whereas HOXA5 and HOXD10 sustain quiescent endothelial phenotype [50, 52, 53]. HOXA3 and HOXD3, which promote a proliferative and migration phenotype, are induced in early phase of endothelial differentiation; HOXA5 and HOXD10, which maintain a quiescent EC phenotype, are increased during the maturation of endothelial cells [50]. Although in pluripotent stem cells the HOX clusters are repressed by epigenomic mechanism such as DNA methylation, histone deacetylation, ncRNA and polycomb/ trithorax repressor complexes [54–56], the epigenetic control and determinants of HOX gene expression in endothelial cells are not well-understood. In this study, epigenomic-wide comparisons between athero-susceptible and -protected sites revealed a sharp distinction of DNA methylation in the HOX loci, which are associated with our previously identified differentially expressed genes, including HOXA4/5/7/10/11 and mir-10a/b [4, 14, 57]. The DMR atlas and athero-susceptible gene profiles reveal epigenomic controls in athero-susceptible genes in the HOX locus worthy of further investigation.

SNP and genetic variants can result in loss or gain of CpG sites which are possible sites of DNA methylation. For example, a T/C polymorphism (refSNP# rs13423988) in a CpG dinucleotide context of the human ARHGAP25 promoter region was found to be associated with blood pressure in genome-wide association studies (GWASs) [38, 58]. It is unknown whether this SNP is associated with its gene expression; however our data suggest that this CpG-SNP may affect ARHGAP25 expression because the methylation of CpG was associated with a decrease of ARHGAP25 expression (Fig. 7e). Similarly, CpG-SNPs that create a CpG in the promoter region of the NDUFB6 gene can be methylated, leading to suppression of gene expression and increasing the susceptibility to type 2 diabetes [57]. These findings support the concept that epigenetic modifications can influence risk in complex diseases. Despite the success of GWASs in identifying loci associated with atherosclerosis, a substantial proportion of the causality remains unexplained as most of the associated genes do not appear to be associated with atherosclerosis [1]. Recently, epigenome-wide association studies (EWASs) have been suggested to be novel opportunities for common human diseases [59, 60]. The methylome and athero-susceptible DMR map are a blueprint for searching epigenomic determinants of atherosclerosis in human and animal models with hypercholesterolemia. Further animal studies with high-cholesterol diet will help to illustrate the functional role of protein-coding and non-coding genes and their epigenomic control in atherogenesis.

## Conclusions

The distribution of atherosclerotic lesions has been linked to arterial branches, bifurcations and curvatures where the local geometry causes the flow to separate from a unidirectional flow trajectory, creating hemodynamics with complex transient flow reversal characteristics referred to as disturbed flow. Endothelial cells are sensitive to local hemodynamic shear stresses. In locally disturbed flow the endothelial transcriptome is significantly different from that of nearby cells outside the region. The cells are considered to be ‘athero-susceptible’ a pre-pathological state of partial activation of pro-inflammatory and ER-stress adaptation to the local hemodynamic environment. Here, following genome-wide analyses of the swine endothelial methylome, we show that in atherosusceptible endothelium *in vivo*, a substantial number of genes linked to the development of cardiovascular disease are associated with regions of DNA hyper/hypomethylation. Over 5500 arterial site-specific DMRs were identified some of which are likely to significantly regulate transcription. Significant links to previously identified transcriptome characteristics of endothelium from these respective arterial sites were identified and validated by MSP. Of particular note are strong associations of DMRs with HOX loci. The study presents the first genome-wide endothelial DNA methylation atlas for exploring site-specific epigenomic mechanisms of regional atherosclerosis susceptibility *in vivo*. Epigenomic DMR characteristics are identified that, in conjunction with miRNAs and transcription factors, regulate gene and protein expression and are a blueprint for spatio-temporal analyses of lesion initiation driven by endothelial dysfunction. Our MeDIP-seq annotation and data are deposited into public archives for data mining.

## Methods

### DNA and RNA isolation from swine aortic endothelial cells

Endothelial cells were obtained from adult pigs of both sexes (Landrace × Yorkshire, 6-month-old, ~250 lb). Pig hearts with attached aortas were provided by the staff of a local abattoir (Clemens Industries, Hatfield, PA) under USDA inspector supervision. A permit for on-site tissue and cell isolation, #6-2015 (to PFD) was issued by the resident USDA inspector. Ascending and descending aortas were dissected, and each vessel lumen was rinsed with ice-cold PBS. Endothelial cells were freshly harvested by gentle scraping of regions (1–2 cm^2^) located at AA and nearby DT, representing athero-susceptible and athero-protective sites respectively. Figure 1e illustrates the morphological phenotypes in AA and DT and examples of immunocytochemical QC monitoring for occasional contaminating smooth muscle and leukocytic cells (undetectable in western analyses). Endothelial purity (>97 %) was as previously described [7].

Genomic DNA was isolated by DNeasy Kit (Qiagen). Total RNA was isolated by RNeasy Kit (Qiagen). The quality of DNA and RNA was evaluated by NanoDrop 1000 Spectrophotometer and TapeStation 2200 (Agilent Technologies). Only high quality DNA (260/280 > 1.8) and RNA (260/280 > 2.0, RNA integrity number >8) were used in this study. The sex of each pig was confirmed by the presence (male) or absence (female) of the sex determining region Y (SRY) gene in chrY. The primers of SRY were F. AACCAGCTCACTTCTGCAAC; R. AACCACGGTGAAAAGGCAAG.

### Multiplexed MeDIP-seq

AA and DT genomic DNA randomly selected from 6 female and 6 male pigs was used in the MeDIP-seq study. One μg genomic DNA was sheared into ~200 bp fragments using a Covaris S2 sonication system (Covaris, Woburn, MA). 24 MeDIP DNA libraries were constructed using DNA Library Prep kit for Illumina (NEB) consisting of DNA end repair, 3’-dA tailing and adapter ligation. After adapter ligation, the DNA was immunoprecipitated by MagMeDIP (Diagenode) with antibody against methylated DNA according to the manufacturer’s protocol. The MeDIP DNA was amplified by PCR using index primers and phusion high-fidelity DNA polymerase (NEB). The PCR cycles consisted of 98 °C 30s,15 cycles of 98 °C 10s, 65 °C 30s, 72 °C 30s, followed by prolonged extension for 5 min at 72 °C. After amplification and purification, MeDIP DNA libraries with 50-100 bp insert size were selected by Sage Science’s Pippin Prep in 2 % agarose gel. Library quality and quantity were evaluated using an Agilent 2100 Analyzer and DNA 1000 chips. The specificity and efficiency of MeDIP enrichment were verified by qPCR with primers targeting ACTA2 promoter F. TTCTAGTGGCCCTGATATTCCC; R. AATTTCGGAGTACGTGAACCC (methylated in endothelial cells) and UBB promoter F. GGGAAGGTGGGAAAGAGGTAG; R. AGCATTGAAATTCCCGTTGGG (unmethylated in endothelial cells). The mean ratio of methylated/unmethylated DNA was 244,700 fold indicating high enrichment of methylated DNA. A total of 24 MeDIP-seq libraries randomly arranged in 4 lanes were sequenced by the Next Generation Sequencing Core at the University of Pennsylvania using Illumina HiSeq 2000 with a 50 bp single-end read length.

### Identification of differentially methylated regions (DMRs)

Sequencing reads were aligned to the pig reference genome Sscrofa10.2 using ELAND. BALM (http://motif.bmi.ohio-state.edu/BALM),^30^ with -*p* = 0.95 and -*w* = 50, was applied to each of the 24 alignment files in order to attach methylation scores to every CpG site and to identify regions enriched in methylation. DMRs were obtained by applying the Bioconductor DiffBind package (with R version = 2.15.0; DiffBind version = 1.2.0; method = EDGER GLM, blocked by animal for the AA vs DT comparison; counts normalized with TMM_READS_EFFECTIVE; minOveralp = 6; FDR threshold = 0.1) to the enriched regions from BALM.

Endothelial cell phenotypes are heterogeneous throughout the cardiovascular system – including at sites within a single vascular bed. Here, each DMR represents the average of several thousand cells harvested from AA and DT arterial sites. Hyper- / hypo-methylation and gene expression may be digital (0,1) for a given gene in individual cells within the site-specific mosaic to yield average DMR and transcript expression values.

### Real-time RT-PCR

cDNA was synthesized by reverse transcribing 0.5-1 μg total RNA with 50 ng random hexamers and SuperScript III reverse transcriptase (Life Technologies). Quantitative-PCR (qPCR) was carried out in a Light Cycler 480 with SYBR Green I Master (Roche, Indianapolis, IN) with 2 μl of first strand cDNA and 500nM primers in a final volume of 20 μl. To exclude the presence of contaminating genomic DNA, a similar analysis was performed in the absence of reverse transcriptase. Reactions were begun with an initial denaturation at 95 °C for 10 min. 35 to 45 cycles were run: 95 °C for 10s, 60 °C for 10s, 72 °C for 10s. A melting curve was run to detect the desired amplicon. Amplicon size was verified by a 2200 TapeStation (Agilent Technologies, Santa Clara, CA). qPCR primers targeting swine genes are listed in Additional file 6: Table S3. The housekeeping genes ubiquitin B (UBB), glyceraldehyde-3-phosphate dehydrogenase (GAPDH), and platelet/endothelial cell adhesion molecule 1 (PECAM1) were used for normalization.

### Methylation specific PCR (MSP)

Methylation specific PCR (MSP) was used to determine DNA methylation at specific loci. All unmethylated cytosines in genomic DNA were converted to uracil by sodium bisulfite conversion (EpiTect kit, Qiagen), while the methylated cytosines were protected and unchanged. Methylation-specific primers which target the methylated DNA were designed by Methyl Primer Express software (Life Technologies). The methylated DNA was amplified during PCR by methylation-specific primers while the unmethylated DNA was not amplified. The MSP primers targeting swine genomic sequence are listed in Additional file 7: Table S4. To normalize the input DNA, we used primers that target the UBB promoter sequence that does not contain any CpG site. The methylation ratio was calculated as ECt _(region of interest)_ / ECt _(UBB)_, where E is the specific amplification efficiency and Ct is the crossing point for sequence region and UBB, respectively.

### Functional enrichment and IPA analyses for DMRs

Gene Ontology (GO) enrichment analysis was performed using the DAVID (Database for Annotation, Visualization and Integrated Discovery) web server (http://david.abcc.ncifcrf.gov/). “Canonical pathways” and “Disease and Bio Function” analyses were performed using Ingenuity Pathway Analysis (IPA) software (www.ingenuity.com/products/ipa). Genes containing DMRs in the promoter region (10 kb upstream and 1 kb downstream of TSS) were mapped to their corresponding human homologs. The gene list was submitted to DAVID for enrichment analysis of the significant overrepresentation of GO Biological Processes and Molecular Function terms. In all tests, the whole set of known genes were appointed as the background. p-values from the DAVID analysis were corrected for multiple testing using Benjamini-Hochberg.

## Additional files

Additional file 1: Figure S1. Endothelial DNA methylation at TSS, TTS, gene body, exon and intron. **Figure S3.** Equivalent global methylation levels of endothelial genome at DT and AA. *n* = 4 animals, paired sites. ns: no significant differences in dot blot O.D. Additional file 2: Table S1a. AA vs DT DMRs. Additional file 3: Table S1b. AA vs DT DMRs in the peri-promoter region (10 kb upstream and 1 kb downstream TSS). Additional file 4: Table S2a. Male-DT vs Female-DT DMR (FDR < 0.1). Additional file 5: Table S2b. Male-AA vs Female-AA DMR (FDR < 0.1). Additional file 6: Table S3. Primers for qPCR analysis. Additional file 7: Table S4. Primers for MSP analysis of swine gene promoter methylation.

## Abbreviations

AA: Aortic arch
ER stress: Endoplasmic reticulum stress
CGI: CpG islands
DT: Descending thoracic aorta
DMRs: Differential methylation regions
GO: Gene Ontology
HOX: Homeobox
IPA: Ingenuity Pathway Analysis
MeDIP-seq: Methylated DNA immunoprecipitation sequencing
MSP: Methylation specific PCR
BP: Biological processes
MF: Molecular function
qPCR: quantitative-PCR
ROS: Reactive oxygen species
SNP: Single nucleotide polymorphisms
TSS: Near transcription start sites
TTS: Transcription termination sites
