# Supplementary material for: Arterial endothelial methylome: differential DNA methylation in athero-susceptible disturbed flow regions in vivo
Source: BMC Genomics. 2015 Jul 7;16:506. doi: 10.1186/s12864-015-1656-4 (PMC4492093; doi:10.1186/s12864-015-1656-4)
Supplement: Additional file 1: Figure S1. — Endothelial DNA methylation at TSS, TTS, gene body, exon and intron. Figure S3. Equivalent global methylation levels of endothelial genome at DT and AA. n = 4 animals, paired sites. ns: no significant differences in dot blot O.D. [file 12864_2015_1656_MOESM1_ESM.pdf]

Supplementary Figures

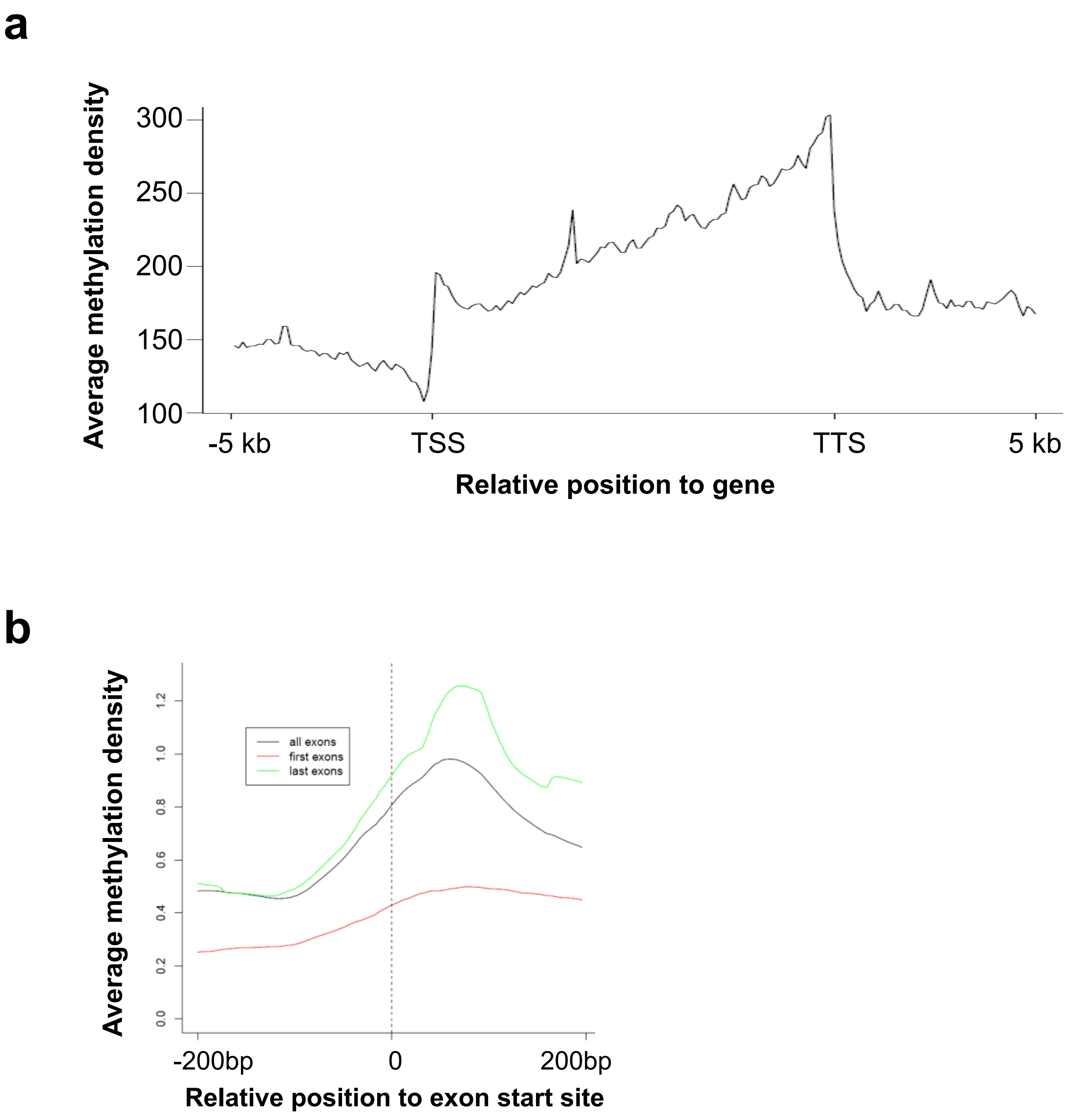

Supplementary Fig. 1

Supplementary Figure 1. Endothelial DNA methylation at TSS, TTS, gene body, exon and intron.
(**a**) Average methylation density over all 24 samples. For each transcript (longer than 2000 bp), the region between its TSS and TTS (referred here as "gene body") was divided into 100 bins and its 5kb flanking regions were divided into 100bp bins, yielding 200 bins. For each sample, the density for the i-th bin was obtained by dividing the count of reads overlapping the i-th bin in all transcripts by the bin's length. (**b**) Average methylation density around exon start sites. For each sample, we flanked each exon start site with 200 bp and took a sliding 10 bp window (with 4 bp overlaps between consecutive windows). Total read counts for the i-th window over all exons were divided by the number of exons.

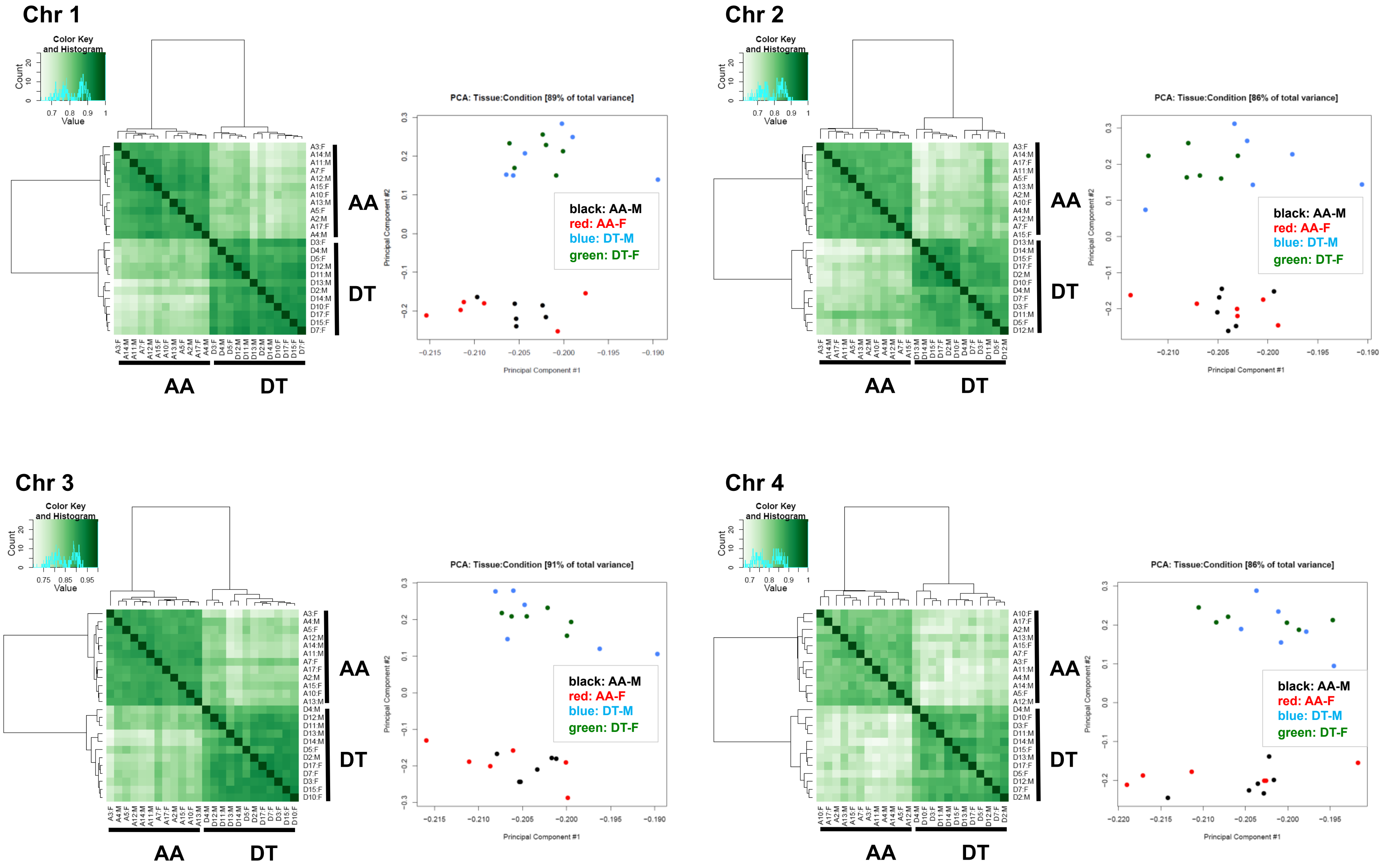

Supplementary Fig. 2 (part 1)

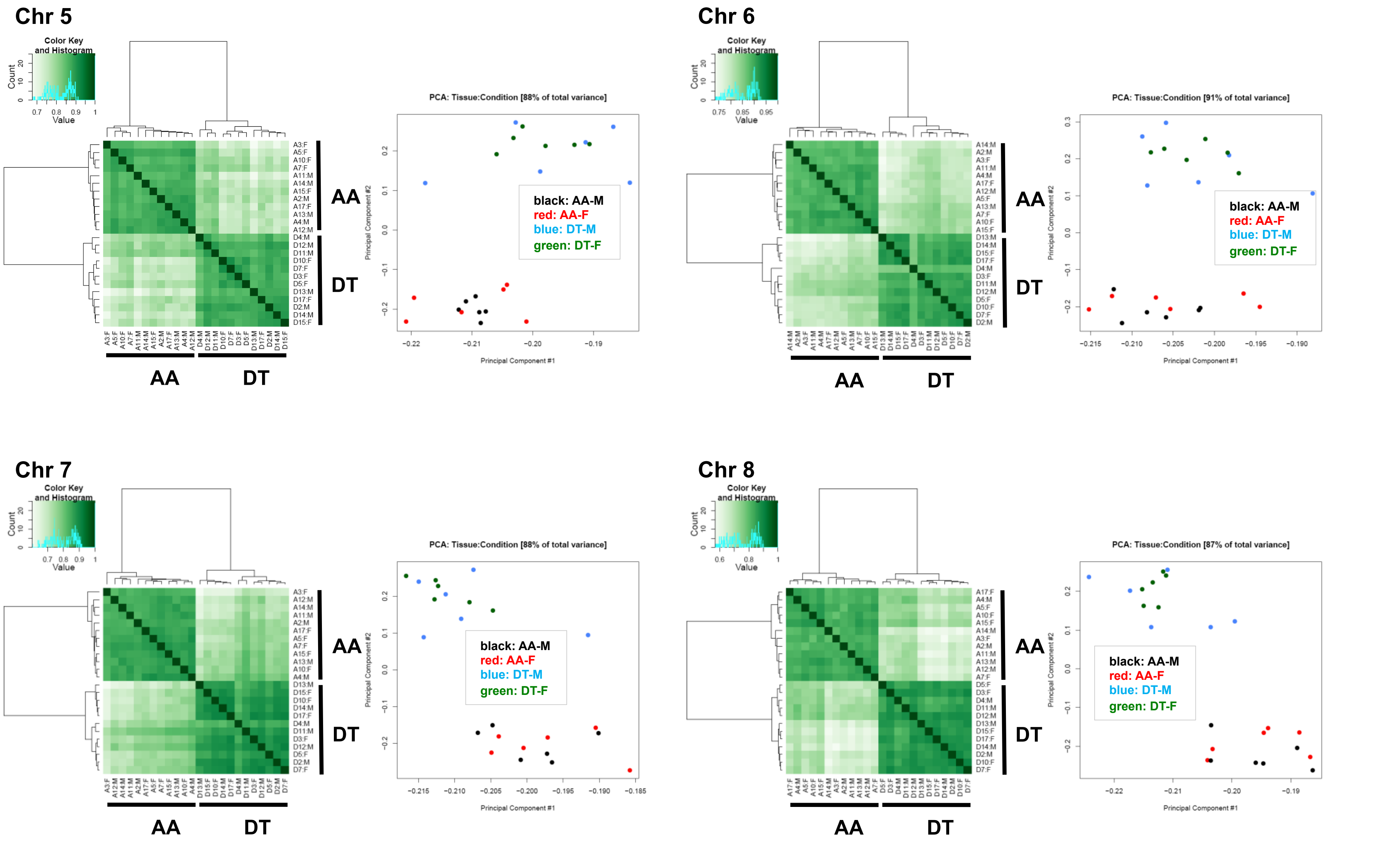

Supplementary Fig. 2 (part 2)

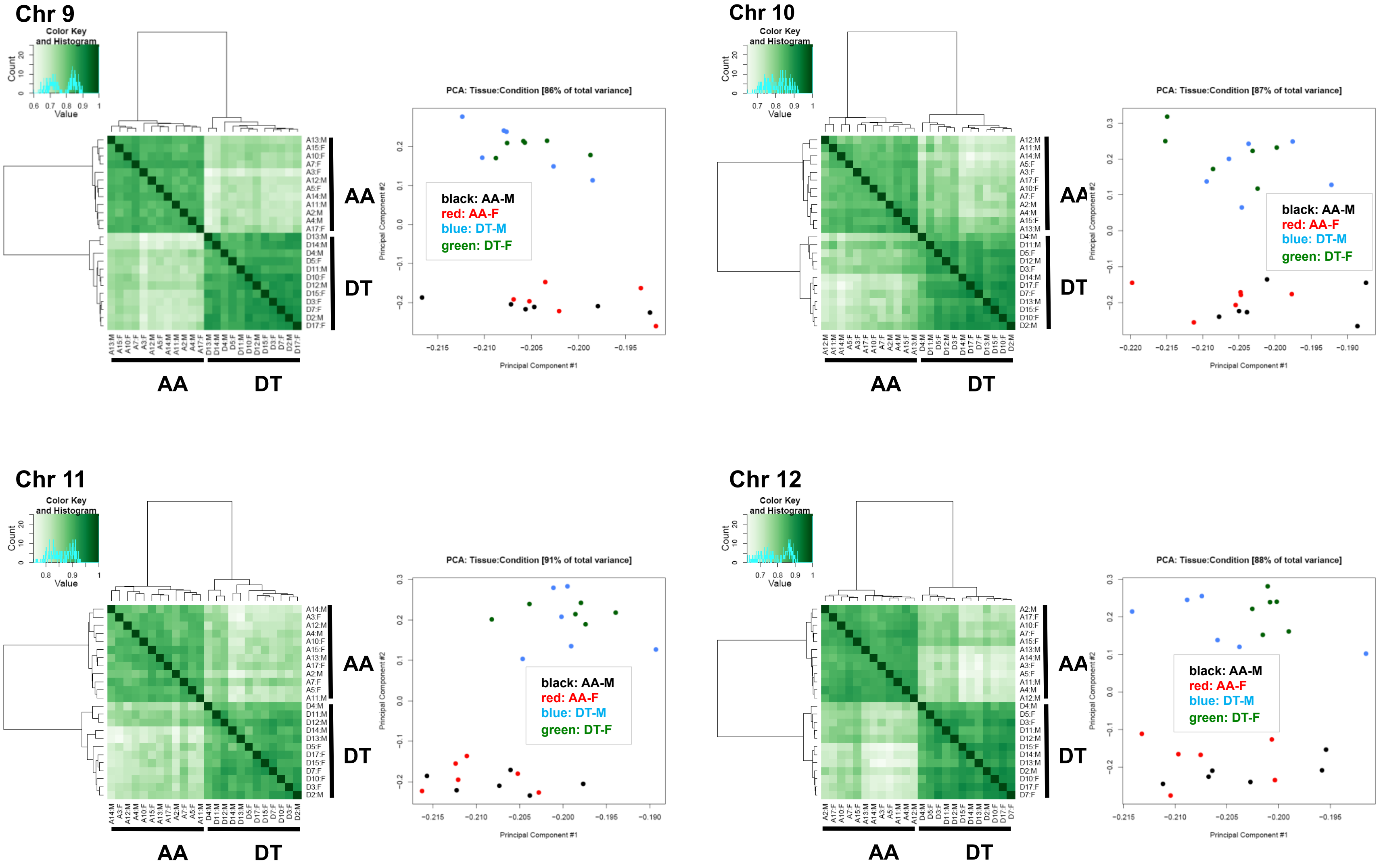

Supplementary Fig. 2 (part 3)

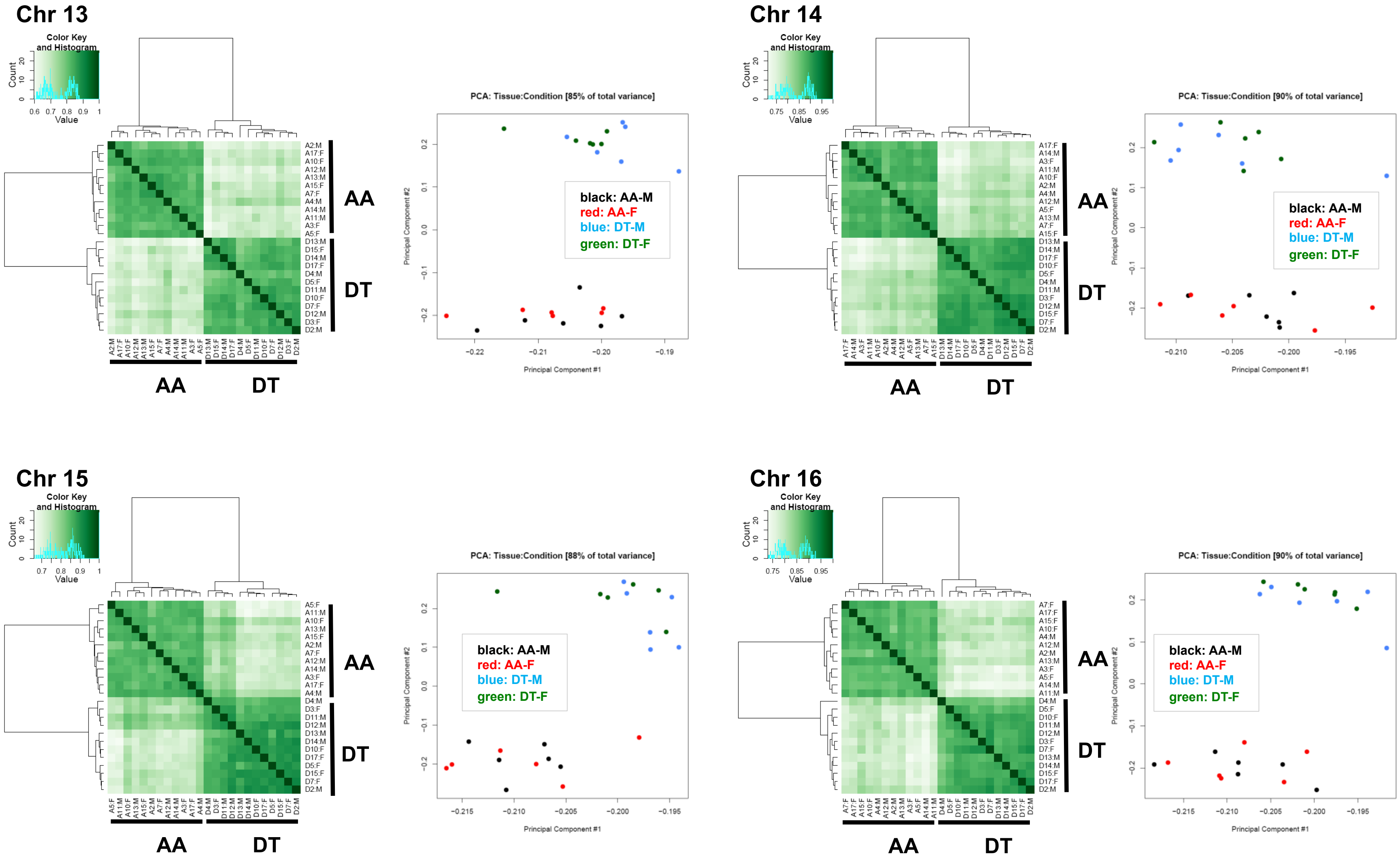

Supplementary Fig. 2 (part 4)

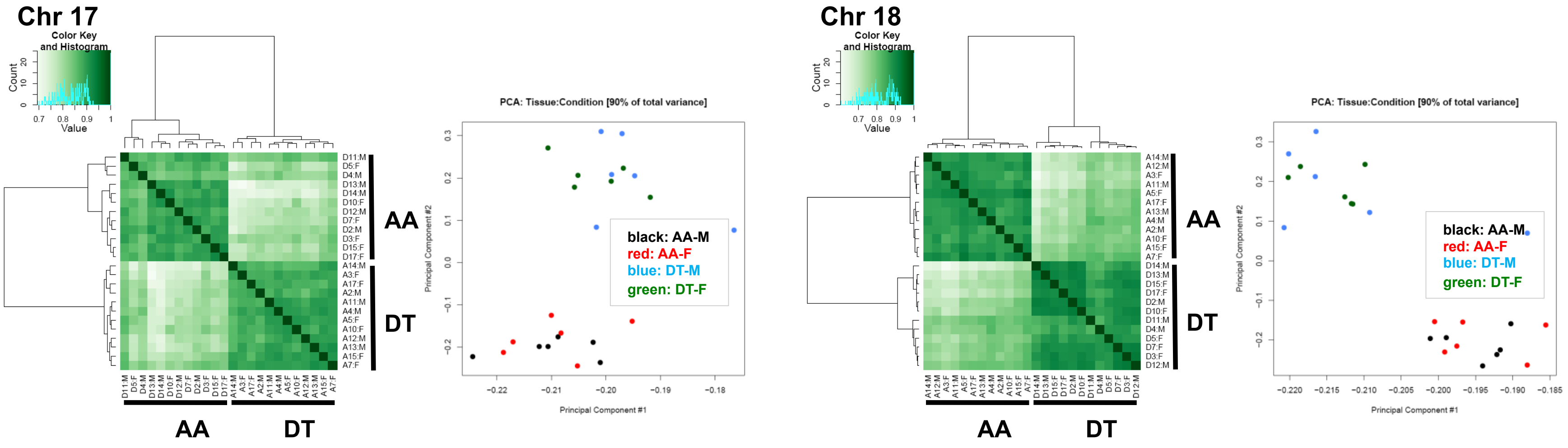

Supplementary Fig. 2 (part 5)

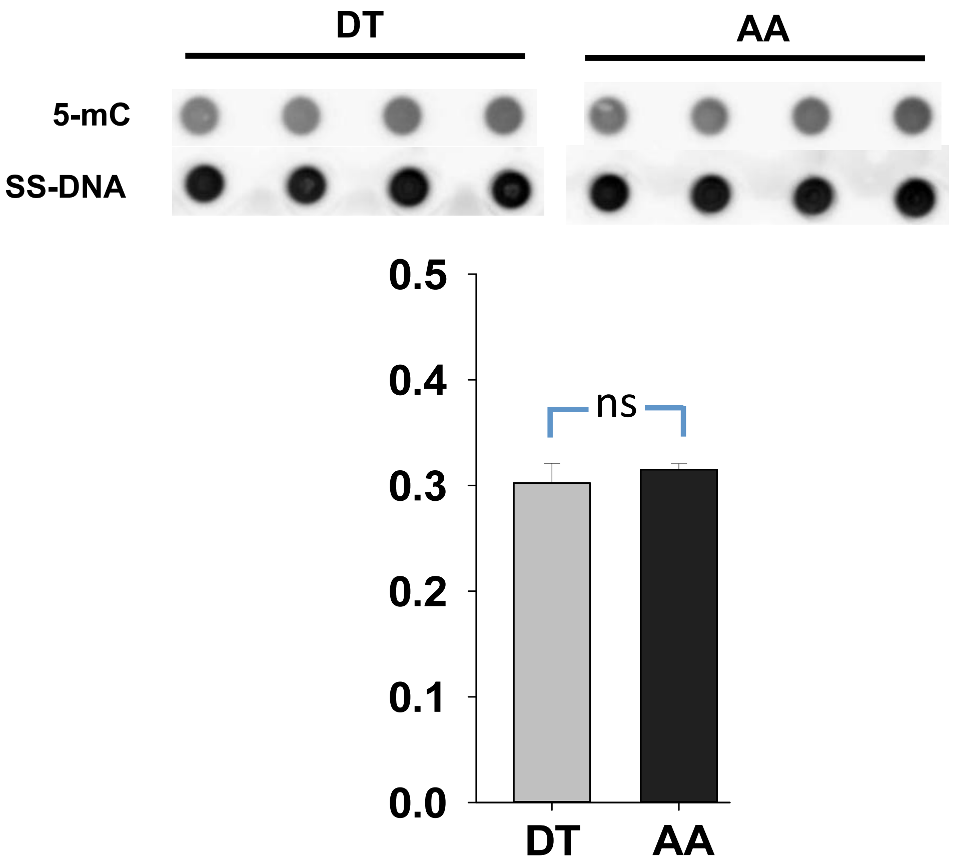

Supplementary Figure 3: Equivalent global methylation levels of endothelial genome at DT and AA. n=4 animals, paired sites. ns: no significant differences in dot blot O.D.

Supplementary Fig. 3
