# Supplementary material for: Arterial endothelial methylome: differential DNA methylation in athero-susceptible disturbed flow regions in vivo
Source: BMC Genomics. 2015 Jul 7;16:506. doi: 10.1186/s12864-015-1656-4 (PMC4492093; doi:10.1186/s12864-015-1656-4)
Supplement: Additional file 2: Table S1a. — AA vs DT DMRs. [file 12864_2015_1656_MOESM2_ESM.pdf]

Supplementary Table 1a. AA vs DT DMRs

DMR	DMR size	Conc	Conc_ AA	Conc_ DT		Fold, log2 (AA/DT)	p-value	FDR
chr1:10072648-10073607	960	4.2	3.8	4.5	↓	-0.7	1.1E-05	5.8E-03
chr1:101194381-101195145	765	4.3	4.1	4.6	↓	-0.5	5.2E-04	9.0E-02
chr1:101207924-101208329	406	2.9	2.3	3.3	↓	-1.0	7.8E-05	2.5E-02
chr1:101209833-101211029	1197	4.9	4.6	5.2	↓	-0.7	4.8E-07	4.1E-04
chr1:101211632-101214877	3246	6.8	6.2	7.2	↓	-1.0	4.7E-14	2.8E-10
chr1:101214973-101215773	801	3.6	3.0	4.0	↓	-1.0	6.9E-07	5.6E-04
chr1:101216313-101217304	992	5.0	4.4	5.5	↓	-1.1	1.2E-18	1.6E-14
chr1:101218305-101220130	1826	5.0	4.7	5.3	↓	-0.6	1.2E-05	6.0E-03
chr1:101220482-101221441	960	4.0	3.3	4.4	↓	-1.2	4.4E-09	8.1E-06
chr1:101771454-101772635	1182	4.8	4.4	5.0	↓	-0.7	4.5E-04	8.2E-02
chr1:10232708-10233241	534	3.9	3.5	4.2	↓	-0.7	5.8E-04	9.6E-02
chr1:103707995-103708493	499	2.4	1.7	2.9	↓	-1.2	2.2E-05	8.9E-03
chr1:103708825-103709398	574	4.1	3.5	4.5	↓	-1.0	5.4E-10	1.4E-06
chr1:103710936-103711398	463	3.3	2.3	3.8	↓	-1.5	9.5E-10	2.3E-06
chr1:106130999-106132248	1250	5.5	5.0	5.8	↓	-0.8	5.3E-09	9.5E-06
chr1:106455466-106455916	451	2.4	2.9	1.7	↑	1.3	6.6E-06	3.9E-03
chr1:107242621-107243912	1292	5.0	4.7	5.3	↓	-0.6	9.6E-06	5.1E-03
chr1:107642230-107642997	768	3.2	3.6	2.7	↑	1.0	1.4E-05	6.5E-03
chr1:107942815-107943461	647	3.2	2.6	3.7	↓	-1.1	5.4E-08	6.6E-05
chr1:108696178-108697032	855	4.1	4.4	3.6	↑	0.9	6.9E-06	4.0E-03
chr1:10897515-10897806	292	2.1	1.2	2.7	↓	-1.5	2.2E-06	1.6E-03
chr1:109034185-109035746	1562	5.6	5.3	5.8	↓	-0.5	5.5E-06	3.3E-03
chr1:109093611-109094169	559	2.4	2.8	1.8	↑	1.0	4.1E-04	7.7E-02
chr1:110359417-110359731	315	1.7	2.3	0.6	↑	1.7	1.7E-06	1.3E-03
chr1:113737036-113738066	1031	3.5	3.1	3.9	↓	-0.8	1.9E-05	7.9E-03
chr1:114972305-114972750	446	4.0	3.7	4.2	↓	-0.6	3.5E-04	7.0E-02
chr1:115036868-115037313	446	2.4	1.8	2.8	↓	-1.1	7.2E-05	2.3E-02
chr1:115390685-115391181	497	4.1	4.4	3.6	↑	0.8	2.3E-05	9.3E-03
chr1:11563699-11565760	2062	6.0	5.7	6.2	↓	-0.5	5.8E-07	4.8E-04
chr1:117585981-117586344	364	2.4	1.8	2.8	↓	-1.1	5.4E-05	1.8E-02
chr1:117807968-117808808	841	4.6	4.8	4.2	↑	0.6	1.4E-05	6.5E-03
chr1:118405608-118405990	383	2.4	1.8	2.8	↓	-1.0	4.2E-04	7.8E-02
chr1:119725750-119726592	843	4.1	4.4	3.6	↑	0.9	4.6E-06	2.9E-03
chr1:121819342-121819953	612	2.8	1.9	3.3	↓	-1.5	2.5E-06	1.7E-03
chr1:122467299-122468350	1052	3.3	3.6	2.9	↑	0.7	2.0E-04	4.8E-02
chr1:122518872-122519386	515	2.8	3.2	2.3	↑	0.9	2.0E-04	4.8E-02
chr1:122840654-122841213	560	4.4	4.0	4.7	↓	-0.7	1.4E-06	1.1E-03
chr1:123082756-123083263	508	3.6	3.9	3.1	↑	0.9	8.8E-05	2.8E-02
chr1:123142113-123144469	2357	6.1	5.9	6.2	↓	-0.3	1.7E-04	4.4E-02
chr1:123174530-123174793	264	1.8	0.8	2.3	↓	-1.6	1.3E-05	6.4E-03
chr1:123355578-123355861	284	2.4	2.8	1.8	↑	0.9	5.5E-04	9.3E-02
chr1:126027396-126028275	880	3.7	4.0	3.4	↑	0.6	6.3E-04	1.0E-01
chr1:126466760-126467786	1027	4.1	3.8	4.4	↓	-0.6	8.9E-05	2.8E-02

chr1:126520788-126521791	1004	4.9	4.6	5.0	↓	-0.4	5.1E-04	8.9E-02
chr1:126756689-126757301	613	4.2	3.9	4.5	↓	-0.6	4.6E-04	8.3E-02
chr1:126813988-126814826	839	3.6	3.9	3.2	↑	0.7	5.9E-04	9.7E-02
chr1:126869640-126870431	792	3.2	3.6	2.8	↑	0.8	2.9E-04	6.4E-02
chr1:127112334-127112634	301	2.3	2.7	1.6	↑	1.2	8.6E-05	2.7E-02
chr1:127216864-127217563	700	4.9	4.6	5.2	↓	-0.6	2.1E-06	1.5E-03
chr1:127236225-127236529	305	2.5	2.9	1.9	↑	1.0	1.9E-04	4.6E-02
chr1:127856483-127856840	358	2.8	1.6	3.5	↓	-1.9	1.2E-12	6.2E-09
chr1:127857586-127858223	638	3.6	3.1	4.0	↓	-0.8	4.8E-05	1.6E-02
chr1:127861770-127864198	2429	4.9	4.4	5.3	↓	-1.0	2.9E-08	4.0E-05
chr1:129111338-129111910	573	3.1	2.6	3.4	↓	-0.9	3.4E-04	6.9E-02
chr1:136551552-136552913	1362	4.1	3.8	4.4	↓	-0.6	2.5E-04	5.6E-02
chr1:136572442-136573135	694	4.7	4.5	4.9	↓	-0.4	2.4E-04	5.6E-02
chr1:140899891-140900294	404	2.6	3.0	2.0	↑	1.0	5.3E-04	9.1E-02
chr1:14123234-14123490	257	2.1	2.5	1.5	↑	1.1	5.6E-04	9.4E-02
chr1:142360174-142360396	223	1.8	1.1	2.3	↓	-1.3	2.0E-04	4.8E-02
chr1:14263639-14265502	1864	5.6	5.8	5.4	↑	0.4	1.9E-06	1.4E-03
chr1:14291905-14293254	1350	4.8	4.6	5.0	↓	-0.4	1.8E-04	4.5E-02
chr1:14340675-14342198	1524	5.9	5.7	6.0	↓	-0.4	3.7E-06	2.4E-03
chr1:144122700-144123045	346	2.5	3.0	1.9	↑	1.0	2.8E-04	6.2E-02
chr1:146289415-146290197	783	5.5	5.3	5.8	↓	-0.5	1.2E-07	1.3E-04
chr1:147127720-147128778	1059	3.9	3.4	4.2	↓	-0.8	3.9E-05	1.4E-02
chr1:148553442-148554842	1401	4.4	4.1	4.6	↓	-0.5	1.6E-04	4.3E-02
chr1:149725943-149727104	1162	4.5	3.9	4.9	↓	-1.0	1.6E-07	1.7E-04
chr1:149728920-149729779	860	4.3	3.5	4.8	↓	-1.3	6.2E-09	1.1E-05
chr1:149731032-149732241	1210	3.2	2.6	3.6	↓	-1.0	2.9E-04	6.3E-02
chr1:149732975-149733385	411	2.5	1.9	2.9	↓	-1.0	1.1E-04	3.3E-02
chr1:149816460-149816948	489	3.0	3.5	2.4	↑	1.1	1.4E-05	6.5E-03
chr1:149819116-149819631	516	3.1	3.6	2.3	↑	1.3	4.8E-08	6.1E-05
chr1:153861939-153862402	464	3.1	2.7	3.5	↓	-0.7	5.6E-04	9.4E-02
chr1:154101394-154102902	1509	5.4	5.7	5.1	↑	0.6	5.2E-08	6.4E-05
chr1:154770011-154770694	684	3.7	3.2	4.0	↓	-0.8	2.3E-05	9.4E-03
chr1:155498582-155499623	1042	3.3	3.7	2.8	↑	0.9	6.8E-05	2.2E-02
chr1:155500639-155504575	3937	6.9	7.1	6.8	↑	0.3	2.1E-04	4.9E-02
chr1:155647870-155648500	631	3.6	3.9	3.2	↑	0.7	3.9E-04	7.5E-02
chr1:155680758-155681374	617	3.3	2.8	3.7	↓	-1.0	3.8E-04	7.4E-02
chr1:155681593-155682566	974	3.4	2.9	3.8	↓	-0.9	1.3E-05	6.5E-03
chr1:155682789-155684082	1294	4.4	3.7	4.8	↓	-1.1	2.4E-09	4.9E-06
chr1:155739539-155740117	579	3.3	2.9	3.6	↓	-0.8	1.6E-04	4.3E-02
chr1:157469564-157470108	545	3.5	2.8	3.9	↓	-1.1	4.7E-07	4.1E-04
chr1:159379450-159379803	354	2.5	1.9	3.0	↓	-1.0	2.1E-04	5.0E-02
chr1:1613273-1616603	3331	5.8	5.4	6.2	↓	-0.8	7.8E-09	1.3E-05
chr1:161610854-161611473	620	4.4	4.7	4.2	↑	0.5	2.2E-04	5.2E-02
chr1:162054177-162054769	593	3.8	3.3	4.1	↓	-0.8	1.1E-04	3.4E-02
chr1:162068109-162069708	1600	5.2	5.0	5.3	↓	-0.4	3.6E-04	7.2E-02
chr1:162075514-162076174	661	4.0	4.3	3.7	↑	0.7	3.0E-05	1.1E-02
chr1:162322008-162322641	634	3.8	3.2	4.2	↓	-1.0	7.8E-09	1.3E-05

chr1:1626113-1628185	2073	6.7	6.6	6.9	↓	-0.3	1.1E-05	5.7E-03
chr1:163538636-163543809	5174	8.0	7.9	8.1	↓	-0.2	1.5E-04	4.1E-02
chr1:163546426-163548408	1983	6.7	6.5	6.8	↓	-0.3	1.4E-06	1.1E-03
chr1:163634046-163641667	7622	8.8	8.7	8.8	↓	-0.2	4.1E-04	7.7E-02
chr1:163805985-163806823	839	3.8	3.4	4.1	↓	-0.7	2.7E-04	6.1E-02
chr1:163828089-163828413	325	2.0	2.5	1.2	↑	1.3	4.7E-05	1.6E-02
chr1:164419501-164420118	618	3.9	3.3	4.4	↓	-1.0	4.2E-06	2.7E-03
chr1:164499497-164499895	399	2.9	3.2	2.4	↑	0.9	6.0E-04	9.8E-02
chr1:164742301-164742693	393	3.2	2.8	3.5	↓	-0.7	4.6E-04	8.3E-02
chr1:164777826-164778963	1138	5.1	4.6	5.5	↓	-0.9	1.2E-11	5.4E-08
chr1:164779262-164781377	2116	5.1	4.3	5.6	↓	-1.3	4.2E-16	3.9E-12
chr1:164782977-164783908	932	4.7	4.3	5.0	↓	-0.6	3.7E-05	1.3E-02
chr1:164867566-164868723	1158	4.5	4.1	4.8	↓	-0.7	5.2E-08	6.4E-05
chr1:165500766-165501244	479	2.5	1.8	2.9	↓	-1.0	2.2E-04	5.1E-02
chr1:16550690-16552228	1539	5.6	5.4	5.7	↓	-0.4	1.3E-04	3.8E-02
chr1:168269626-168269964	339	3.0	2.6	3.3	↓	-0.8	2.8E-04	6.2E-02
chr1:170050987-170051672	686	3.4	3.9	2.4	↑	1.5	1.3E-07	1.4E-04
chr1:171961346-171961714	369	2.1	2.5	1.3	↑	1.2	1.3E-04	3.8E-02
chr1:17258711-17259784	1074	4.5	4.7	4.2	↑	0.5	5.9E-04	9.7E-02
chr1:174007112-174007421	310	2.5	1.9	2.9	↓	-1.0	1.6E-04	4.2E-02
chr1:176156089-176156491	403	3.1	3.5	2.6	↑	0.9	7.8E-05	2.5E-02
chr1:176594382-176596724	2343	6.3	6.1	6.4	↓	-0.4	1.2E-07	1.3E-04
chr1:176608564-176609283	720	4.8	5.0	4.5	↑	0.5	1.4E-04	4.0E-02
chr1:176619287-176620164	878	5.8	5.6	6.0	↓	-0.5	9.8E-09	1.5E-05
chr1:176899901-176900240	340	2.9	2.4	3.3	↓	-1.0	8.9E-05	2.8E-02
chr1:176923783-176924461	679	3.4	2.6	3.8	↓	-1.2	4.3E-08	5.7E-05
chr1:179586932-179588835	1904	4.9	5.1	4.7	↑	0.4	3.1E-04	6.7E-02
chr1:179661302-179663608	2307	5.9	6.2	5.6	↑	0.6	7.5E-08	8.7E-05
chr1:179663787-179664702	916	4.5	4.8	4.0	↑	0.7	4.0E-07	3.6E-04
chr1:179667496-179667971	476	2.5	3.1	1.5	↑	1.7	8.8E-09	1.4E-05
chr1:179668128-179670953	2826	6.1	6.5	5.6	↑	0.9	3.8E-15	3.1E-11
chr1:179672958-179674187	1230	4.0	4.4	3.5	↑	0.9	1.7E-07	1.7E-04
chr1:179675803-179676642	840	3.2	3.6	2.8	↑	0.8	2.4E-04	5.6E-02
chr1:179680040-179680939	900	3.8	4.1	3.4	↑	0.8	9.6E-06	5.1E-03
chr1:180577369-180578925	1557	5.4	5.7	5.1	↑	0.6	8.2E-09	1.3E-05
chr1:180741836-180742257	422	2.9	2.4	3.2	↓	-0.8	6.1E-04	9.9E-02
chr1:181156308-181156676	369	2.3	2.7	1.7	↑	1.0	4.0E-04	7.6E-02
chr1:181895050-181895447	398	2.3	2.8	1.8	↑	1.0	3.9E-04	7.4E-02
chr1:182455370-182455856	487	3.1	2.6	3.5	↓	-0.8	5.7E-04	9.6E-02
chr1:182460105-182461248	1144	3.9	3.5	4.3	↓	-0.7	3.9E-06	2.5E-03
chr1:183195350-183195956	607	2.8	3.2	2.1	↑	1.1	1.9E-05	7.9E-03
chr1:184688283-184688548	266	2.1	2.5	1.4	↑	1.1	5.9E-04	9.7E-02
chr1:185315856-185316242	387	2.2	2.7	1.6	↑	1.1	2.0E-04	4.8E-02
chr1:185399973-185400234	262	2.0	1.3	2.5	↓	-1.2	1.5E-04	4.1E-02
chr1:185408217-185408773	557	3.3	2.8	3.6	↓	-0.8	1.5E-04	4.1E-02
chr1:185684850-185685395	546	3.7	3.2	4.1	↓	-0.8	7.0E-06	4.0E-03
chr1:186585509-186586710	1202	4.5	4.2	4.7	↓	-0.5	2.4E-05	9.5E-03

chr1:186599010-186599394	385	2.5	1.8	3.0	↓	-1.1	1.7E-05	7.4E-03
chr1:186601301-186601935	635	2.5	1.9	2.9	↓	-1.0	9.1E-05	2.8E-02
chr1:186764097-186764400	304	2.3	1.7	2.7	↓	-1.0	3.8E-04	7.4E-02
chr1:187435626-187435883	258	2.2	1.6	2.7	↓	-1.0	3.6E-04	7.2E-02
chr1:187725341-187725593	253	1.6	2.1	0.9	↑	1.3	6.1E-04	9.9E-02
chr1:187802796-187803849	1054	4.2	3.7	4.5	↓	-0.7	4.1E-05	1.4E-02
chr1:187913503-187914972	1470	5.4	5.7	5.1	↑	0.6	1.7E-07	1.7E-04
chr1:189390134-189391172	1039	3.6	3.2	3.8	↓	-0.6	4.9E-04	8.7E-02
chr1:189404554-189404786	233	1.9	1.1	2.4	↓	-1.2	1.5E-04	4.1E-02
chr1:1923928-1924870	943	4.8	4.5	5.1	↓	-0.6	1.4E-05	6.5E-03
chr1:19261286-19263375	2090	6.0	5.8	6.2	↓	-0.4	1.8E-04	4.5E-02
chr1:19482230-19482804	575	3.2	2.5	3.7	↓	-1.2	6.9E-08	8.2E-05
chr1:1955707-1957311	1605	5.0	4.7	5.2	↓	-0.5	3.1E-04	6.6E-02
chr1:200857415-200858442	1028	4.1	4.3	3.8	↑	0.6	1.8E-04	4.5E-02
chr1:201009449-201010318	870	4.1	3.5	4.5	↓	-1.0	8.5E-06	4.7E-03
chr1:201317973-201318431	459	3.6	3.2	3.9	↓	-0.7	5.9E-04	9.7E-02
chr1:201827298-201828000	703	3.0	3.5	2.4	↑	1.1	3.9E-05	1.4E-02
chr1:201828182-201828821	640	4.1	4.5	3.6	↑	0.9	3.4E-05	1.3E-02
chr1:201834826-201835190	365	2.3	2.8	1.6	↑	1.2	1.3E-04	3.7E-02
chr1:201860888-201861407	520	3.2	3.6	2.5	↑	1.1	1.7E-06	1.3E-03
chr1:204048481-204049229	749	3.9	3.5	4.2	↓	-0.7	2.6E-05	1.0E-02
chr1:204078354-204078937	584	2.9	2.5	3.3	↓	-0.8	2.8E-04	6.2E-02
chr1:204081861-204082144	284	2.1	1.2	2.6	↓	-1.4	1.8E-05	7.9E-03
chr1:204129526-204131032	1507	5.2	5.5	4.8	↑	0.7	3.8E-10	1.1E-06
chr1:204443632-204444192	561	2.8	2.3	3.2	↓	-0.9	2.0E-04	4.9E-02
chr1:205868761-205869317	557	4.5	4.1	4.8	↓	-0.7	2.4E-05	9.5E-03
chr1:206514937-206515579	643	3.1	3.4	2.6	↑	0.8	1.6E-04	4.2E-02
chr1:206555896-206556583	688	3.2	3.7	2.3	↑	1.5	2.3E-10	7.3E-07
chr1:206572438-206573032	595	2.3	2.9	1.4	↑	1.5	5.5E-06	3.3E-03
chr1:206576707-206577044	338	2.5	3.0	1.8	↑	1.2	7.0E-06	4.0E-03
chr1:206640466-206641380	915	4.0	4.4	3.3	↑	1.1	3.2E-09	6.2E-06
chr1:206802814-206803173	360	3.4	3.8	2.8	↑	1.0	4.4E-04	8.0E-02
chr1:207160746-207161130	385	2.0	1.3	2.5	↓	-1.2	3.2E-04	6.8E-02
chr1:207566846-207567387	542	3.2	2.7	3.5	↓	-0.7	3.5E-04	7.0E-02
chr1:209441336-209441969	634	3.7	3.3	4.0	↓	-0.7	1.5E-05	7.0E-03
chr1:209537063-209537850	788	5.0	4.6	5.3	↓	-0.7	1.5E-08	2.2E-05
chr1:210233494-210233818	325	2.5	1.9	2.9	↓	-0.9	4.1E-04	7.7E-02
chr1:21065647-21066301	655	4.4	4.1	4.7	↓	-0.6	2.2E-05	9.0E-03
chr1:210724259-210724842	584	3.4	4.0	2.4	↑	1.6	2.3E-10	7.3E-07
chr1:210725131-210727895	2765	5.7	6.3	4.7	↑	1.6	7.1E-20	1.2E-15
chr1:210727930-210728367	438	2.4	2.9	1.7	↑	1.2	4.6E-05	1.6E-02
chr1:210728371-210732351	3981	7.1	7.7	6.3	↑	1.3	2.3E-23	7.5E-19
chr1:210736110-210737153	1044	3.1	3.6	2.3	↑	1.3	2.4E-07	2.4E-04
chr1:210737190-210738562	1373	4.5	4.9	3.9	↑	0.9	5.6E-11	2.3E-07
chr1:210739364-210741392	2029	6.1	6.4	5.8	↑	0.5	5.7E-14	3.1E-10
chr1:210789295-210790211	917	3.3	2.7	3.7	↓	-1.0	1.6E-07	1.7E-04
chr1:21159419-21159773	355	2.6	2.0	3.0	↓	-1.0	3.2E-04	6.8E-02

chr1:212907961-212908500	540	3.6	3.2	3.9	↓	-0.7	3.4E-04	7.0E-02
chr1:213026915-213027638	724	3.9	3.4	4.2	↓	-0.8	2.3E-06	1.6E-03
chr1:213223140-213223685	546	3.5	3.1	3.8	↓	-0.7	4.7E-04	8.4E-02
chr1:213320819-213321097	279	1.9	1.2	2.4	↓	-1.2	2.1E-04	5.0E-02
chr1:215937393-215937819	427	2.4	2.9	1.6	↑	1.3	7.8E-05	2.5E-02
chr1:216553322-216553979	658	3.0	3.4	2.5	↑	1.0	2.1E-04	4.9E-02
chr1:216927156-216927590	435	2.4	1.8	2.8	↓	-0.9	5.1E-04	8.9E-02
chr1:216993405-216993820	416	2.3	1.5	2.8	↓	-1.3	1.3E-05	6.2E-03
chr1:217050593-217050890	298	2.5	1.9	3.0	↓	-1.1	1.7E-04	4.4E-02
chr1:22147163-22147869	707	3.5	2.9	3.9	↓	-0.9	3.1E-06	2.1E-03
chr1:221910767-221911047	281	2.0	1.3	2.5	↓	-1.2	1.7E-04	4.4E-02
chr1:222701331-222701831	501	2.5	1.8	3.0	↓	-1.1	1.7E-04	4.4E-02
chr1:22559439-22559643	205	1.5	0.7	2.0	↓	-1.3	3.7E-04	7.2E-02
chr1:22561633-22562370	738	3.1	1.8	3.8	↓	-2.0	4.7E-17	5.1E-13
chr1:22564131-22564393	263	1.7	0.6	2.2	↓	-1.6	4.8E-05	1.6E-02
chr1:225952553-225953788	1236	4.4	4.8	4.0	↑	0.8	3.0E-06	2.1E-03
chr1:226322064-226324025	1962	5.8	5.3	6.2	↓	-0.8	3.2E-09	6.2E-06
chr1:226327024-226328526	1503	5.5	4.9	5.9	↓	-1.0	1.8E-12	8.3E-09
chr1:226440604-226441372	769	2.8	3.2	2.3	↑	0.9	2.4E-04	5.6E-02
chr1:226475145-226475506	362	3.1	2.6	3.4	↓	-0.9	1.4E-04	4.0E-02
chr1:226525613-226526150	538	2.5	1.9	2.9	↓	-1.0	5.7E-04	9.4E-02
chr1:229744215-229745797	1583	4.5	4.0	4.9	↓	-0.9	3.1E-07	3.0E-04
chr1:229806731-229807746	1016	3.6	3.0	4.0	↓	-1.0	2.4E-05	9.7E-03
chr1:2298109-2298444	336	3.7	3.3	4.0	↓	-0.6	3.6E-04	7.2E-02
chr1:231181282-231181911	630	2.6	2.0	2.9	↓	-0.9	5.5E-04	9.4E-02
chr1:232397726-232398564	839	4.7	4.1	5.1	↓	-1.0	1.6E-14	1.0E-10
chr1:232778898-232779585	688	3.7	3.2	4.1	↓	-0.8	1.7E-04	4.4E-02
chr1:2359413-2359733	321	3.6	3.1	3.9	↓	-0.8	3.1E-05	1.2E-02
chr1:236487914-236488482	569	4.2	4.6	3.7	↑	0.8	8.9E-06	4.8E-03
chr1:23718876-23719344	469	3.5	2.8	3.9	↓	-1.0	1.0E-06	8.4E-04
chr1:238946704-238947382	679	3.0	3.4	2.5	↑	0.8	2.5E-04	5.7E-02
chr1:239988919-239989315	397	3.5	3.7	3.1	↑	0.7	3.5E-04	7.0E-02
chr1:24212605-24213210	606	3.1	2.7	3.5	↓	-0.8	1.3E-04	3.8E-02
chr1:24344400-24345228	829	4.8	4.5	5.1	↓	-0.6	7.3E-06	4.2E-03
chr1:24430364-24432562	2199	5.5	5.7	5.3	↑	0.4	2.1E-05	8.7E-03
chr1:2460230-2465847	5618	8.7	8.6	8.8	↓	-0.1	4.6E-04	8.3E-02
chr1:248236378-248236928	551	3.3	2.6	3.8	↓	-1.2	7.6E-06	4.3E-03
chr1:248239138-248239904	767	4.7	4.4	5.0	↓	-0.6	5.8E-04	9.6E-02
chr1:248240065-248240469	405	3.4	3.1	3.8	↓	-0.7	2.9E-04	6.4E-02
chr1:24825960-24827095	1136	4.6	4.2	4.9	↓	-0.7	3.3E-04	6.9E-02
chr1:248304883-248305174	292	2.5	2.9	1.9	↑	1.0	1.5E-04	4.1E-02
chr1:24857406-24857638	233	1.2	0.2	1.7	↓	-1.5	3.3E-04	6.9E-02
chr1:248741996-248742682	687	2.7	3.1	2.2	↑	1.0	3.1E-04	6.6E-02
chr1:25156941-25157353	413	2.3	1.5	2.9	↓	-1.3	1.4E-05	6.5E-03
chr1:2535551-2541607	6057	8.5	8.4	8.6	↓	-0.2	1.2E-04	3.6E-02
chr1:255090164-255090951	788	3.1	3.5	2.6	↑	0.9	1.6E-04	4.3E-02
chr1:255531337-255532069	733	3.3	2.7	3.7	↓	-1.1	4.7E-06	2.9E-03

chr1:256665909-256668072	2164	5.0	4.6	5.3	↓	-0.7	3.6E-06	2.3E-03
chr1:257333896-257334781	886	4.0	3.7	4.3	↓	-0.6	5.8E-04	9.7E-02
chr1:257981105-257981355	251	1.8	0.1	2.5	↓	-2.4	1.3E-09	2.8E-06
chr1:258084833-258085976	1144	5.2	5.0	5.4	↓	-0.4	6.3E-04	1.0E-01
chr1:259387668-259389054	1387	4.2	3.9	4.5	↓	-0.6	1.4E-04	3.9E-02
chr1:259391988-259392473	486	2.8	2.3	3.2	↓	-0.9	3.2E-04	6.8E-02
chr1:259395967-259396389	423	2.1	1.3	2.7	↓	-1.3	4.0E-05	1.4E-02
chr1:261759312-261759889	578	2.8	3.2	2.3	↑	0.9	6.2E-04	9.9E-02
chr1:261801510-261801790	281	1.9	1.2	2.4	↓	-1.2	3.2E-04	6.8E-02
chr1:261806567-261807230	664	4.1	3.7	4.5	↓	-0.8	4.3E-07	3.8E-04
chr1:261933618-261934580	963	3.8	3.5	4.1	↓	-0.7	4.6E-05	1.6E-02
chr1:261947715-261949010	1296	4.1	3.8	4.4	↓	-0.6	1.7E-05	7.4E-03
chr1:261949353-261950552	1200	3.8	3.5	4.1	↓	-0.6	4.0E-04	7.6E-02
chr1:263299759-263300175	417	2.2	1.5	2.7	↓	-1.2	7.3E-05	2.4E-02
chr1:265397394-265398806	1413	4.6	4.1	4.9	↓	-0.8	3.6E-05	1.3E-02
chr1:265433172-265433839	668	4.3	3.9	4.6	↓	-0.6	2.3E-05	9.4E-03
chr1:265474764-265475916	1153	5.1	4.7	5.5	↓	-0.7	1.9E-05	8.0E-03
chr1:266238243-266238719	477	2.6	3.0	2.1	↑	1.0	3.0E-04	6.5E-02
chr1:266835475-266835984	510	2.8	2.3	3.2	↓	-0.9	5.2E-04	9.0E-02
chr1:266864477-266865119	643	3.5	3.1	3.8	↓	-0.7	4.5E-04	8.2E-02
chr1:268329155-268331199	2045	6.0	5.9	6.1	↓	-0.3	5.3E-04	9.2E-02
chr1:26837427-26837811	385	2.4	1.8	2.8	↓	-1.0	4.3E-04	8.0E-02
chr1:268953752-268954777	1026	4.7	4.3	5.0	↓	-0.7	3.8E-07	3.5E-04
chr1:269046677-269047747	1071	5.4	5.1	5.6	↓	-0.4	2.9E-05	1.1E-02
chr1:269285958-269286374	417	2.2	1.4	2.7	↓	-1.4	4.6E-06	2.9E-03
chr1:269286692-269287105	414	1.9	0.8	2.5	↓	-1.7	5.4E-07	4.6E-04
chr1:269408199-269408497	299	2.3	2.7	1.7	↑	1.1	1.6E-04	4.2E-02
chr1:269461875-269462317	443	3.4	3.0	3.7	↓	-0.7	5.3E-04	9.1E-02
chr1:26960071-26960608	538	3.7	3.1	4.1	↓	-0.9	2.5E-07	2.5E-04
chr1:269917047-269918612	1566	5.8	5.5	6.1	↓	-0.6	1.4E-05	6.5E-03
chr1:269918634-269919043	410	2.9	2.4	3.3	↓	-0.9	7.0E-05	2.3E-02
chr1:269920467-269920809	343	3.0	2.6	3.4	↓	-0.8	2.7E-04	6.0E-02
chr1:269921323-269921724	402	2.1	1.4	2.6	↓	-1.2	1.7E-04	4.4E-02
chr1:27170683-27171055	373	2.3	1.6	2.7	↓	-1.2	2.8E-04	6.2E-02
chr1:278271775-278272711	937	4.4	4.0	4.7	↓	-0.6	1.8E-05	7.9E-03
chr1:278504313-278505023	711	3.3	2.2	3.9	↓	-1.7	4.4E-15	3.2E-11
chr1:279062208-279063677	1470	4.9	4.6	5.2	↓	-0.6	2.0E-06	1.4E-03
chr1:279303535-279304215	681	3.1	3.4	2.6	↑	0.8	1.6E-04	4.3E-02
chr1:280051483-280052092	610	3.5	3.1	3.9	↓	-0.7	1.8E-04	4.5E-02
chr1:280778323-280778787	465	3.2	2.5	3.7	↓	-1.2	4.8E-08	6.1E-05
chr1:284870613-284871938	1326	4.5	4.2	4.8	↓	-0.6	3.4E-04	6.9E-02
chr1:284916867-284918525	1659	5.6	5.4	5.8	↓	-0.4	3.9E-04	7.4E-02
chr1:285025563-285026329	767	4.4	4.0	4.6	↓	-0.6	6.5E-05	2.2E-02
chr1:285938971-285940685	1715	4.6	4.2	4.9	↓	-0.7	2.5E-05	9.8E-03
chr1:285977194-285982495	5302	7.1	6.9	7.2	↓	-0.2	2.5E-06	1.7E-03
chr1:286014672-286016544	1873	4.9	5.1	4.7	↑	0.4	5.9E-04	9.7E-02
chr1:286827631-286828592	962	5.5	5.1	5.9	↓	-0.7	4.5E-10	1.3E-06

chr1:286830470-286831128	659	4.1	3.6	4.5	↓	-0.9	1.3E-06	1.1E-03
chr1:286849609-286850437	829	4.9	4.7	5.1	↓	-0.4	6.3E-05	2.1E-02
chr1:287298292-287299083	792	3.8	3.5	4.1	↓	-0.6	3.5E-04	7.1E-02
chr1:287431166-287431831	666	2.9	3.3	2.5	↑	0.8	4.7E-04	8.4E-02
chr1:288476950-288477679	730	3.0	3.4	2.5	↑	0.8	4.1E-04	7.7E-02
chr1:288649691-288650178	488	3.6	3.2	3.9	↓	-0.7	2.8E-04	6.2E-02
chr1:288696853-288697513	661	3.2	3.5	2.6	↑	0.9	4.3E-05	1.5E-02
chr1:289426963-289427284	322	1.9	1.2	2.4	↓	-1.2	2.0E-04	4.8E-02
chr1:291673335-291674008	674	4.2	3.8	4.4	↓	-0.6	2.7E-04	5.9E-02
chr1:291864494-291865323	830	3.6	3.2	3.8	↓	-0.6	4.0E-04	7.6E-02
chr1:293555624-293556094	471	2.4	1.9	2.8	↓	-1.0	4.0E-04	7.6E-02
chr1:297466605-297467043	439	3.4	3.7	3.0	↑	0.7	5.5E-04	9.3E-02
chr1:2977960-2980512	2553	6.8	6.7	7.0	↓	-0.2	9.9E-06	5.2E-03
chr1:298127327-298128141	815	3.9	4.2	3.4	↑	0.7	7.3E-05	2.4E-02
chr1:298695221-298695859	639	3.4	3.0	3.7	↓	-0.7	1.4E-04	3.9E-02
chr1:298723734-298725376	1643	5.2	5.5	5.0	↑	0.5	2.7E-05	1.0E-02
chr1:299004447-299004772	326	2.5	2.9	1.9	↑	0.9	5.1E-04	8.9E-02
chr1:299492273-299492679	407	2.4	2.8	1.9	↑	1.0	3.9E-04	7.4E-02
chr1:299825335-299826031	697	4.2	3.8	4.5	↓	-0.7	1.6E-05	7.2E-03
chr1:300552080-300554197	2118	5.3	5.0	5.6	↓	-0.6	3.3E-06	2.2E-03
chr1:301051865-301052802	938	3.9	3.2	4.3	↓	-1.1	2.6E-07	2.5E-04
chr1:301104326-301105544	1219	5.1	4.8	5.3	↓	-0.4	6.1E-04	9.9E-02
chr1:301254378-301255156	779	4.3	4.0	4.5	↓	-0.6	3.6E-04	7.1E-02
chr1:301619694-301620386	693	5.4	5.0	5.6	↓	-0.6	5.0E-10	1.4E-06
chr1:301631911-301633368	1458	5.9	5.7	6.1	↓	-0.3	3.0E-04	6.4E-02
chr1:302065427-302066537	1111	4.0	3.3	4.4	↓	-1.1	1.2E-09	2.8E-06
chr1:302311986-302313808	1823	4.9	5.3	4.5	↑	0.7	1.0E-04	3.1E-02
chr1:302548186-302551014	2829	6.1	5.7	6.4	↓	-0.7	1.1E-09	2.5E-06
chr1:303196529-303196898	370	2.3	1.7	2.7	↓	-1.0	4.7E-04	8.5E-02
chr1:303441794-303443706	1913	5.8	5.6	6.0	↓	-0.4	9.6E-05	2.9E-02
chr1:303493953-303495362	1410	5.8	6.1	5.5	↑	0.6	3.0E-08	4.1E-05
chr1:303749710-303750047	338	1.7	0.8	2.3	↓	-1.5	3.4E-05	1.3E-02
chr1:304279687-304280493	807	5.0	4.8	5.2	↓	-0.5	1.1E-05	5.7E-03
chr1:304409020-304410741	1722	5.6	5.8	5.5	↑	0.3	1.4E-04	3.9E-02
chr1:304533547-304534020	474	2.7	2.0	3.2	↓	-1.2	3.4E-05	1.3E-02
chr1:304540842-304541827	986	5.1	4.8	5.4	↓	-0.5	1.5E-04	4.1E-02
chr1:305256824-305257472	649	3.8	3.5	4.1	↓	-0.6	4.6E-04	8.3E-02
chr1:305465806-305466491	686	4.2	3.9	4.4	↓	-0.6	1.1E-04	3.4E-02
chr1:305589149-305590006	858	4.3	4.6	4.1	↑	0.5	2.2E-04	5.1E-02
chr1:305669249-305669934	686	4.5	4.3	4.7	↓	-0.4	4.9E-04	8.8E-02
chr1:305956065-305956774	710	5.3	5.1	5.5	↓	-0.5	9.3E-05	2.9E-02
chr1:306111869-306112506	638	5.1	4.7	5.4	↓	-0.7	1.6E-05	7.3E-03
chr1:306374565-306379978	5414	7.6	7.5	7.7	↓	-0.2	1.0E-05	5.2E-03
chr1:306908919-306910314	1396	6.4	6.1	6.6	↓	-0.5	1.8E-05	7.9E-03
chr1:307135779-307143457	7679	7.9	7.9	8.0	↓	-0.2	7.7E-06	4.3E-03
chr1:307149662-307156580	6919	7.9	7.8	7.9	↓	-0.1	5.4E-04	9.2E-02
chr1:307498325-307499012	688	4.4	4.1	4.6	↓	-0.5	2.3E-04	5.3E-02

chr1:307636292-307644957	8666	8.9	8.8	8.9	↓	-0.2	2.5E-08	3.5E-05
chr1:307920059-307926578	6520	8.4	8.3	8.5	↓	-0.2	1.0E-05	5.2E-03
chr1:308377264-308379832	2569	7.1	6.9	7.2	↓	-0.3	3.2E-04	6.7E-02
chr1:308660765-308670086	9322	9.1	9.0	9.2	↓	-0.2	2.0E-04	4.8E-02
chr1:308700700-308707497	6798	8.5	8.4	8.7	↓	-0.3	1.1E-07	1.3E-04
chr1:309257578-309258438	861	4.1	4.4	3.8	↑	0.6	2.6E-04	5.9E-02
chr1:310103777-310104476	700	2.2	2.7	1.5	↑	1.1	3.2E-04	6.8E-02
chr1:311242718-311243578	861	3.8	4.1	3.4	↑	0.7	1.5E-04	4.1E-02
chr1:311712723-311714686	1964	5.9	6.1	5.8	↑	0.3	5.0E-04	8.8E-02
chr1:311922269-311922695	427	4.1	3.8	4.4	↓	-0.6	2.5E-05	9.8E-03
chr1:314085330-314088714	3385	7.2	7.1	7.4	↓	-0.3	3.7E-07	3.4E-04
chr1:3193261-3195620	2360	6.0	5.8	6.2	↓	-0.4	3.0E-05	1.1E-02
chr1:3346901-3350094	3194	7.2	7.1	7.3	↓	-0.2	1.4E-05	6.5E-03
chr1:33598147-33598729	583	4.1	3.8	4.4	↓	-0.6	3.7E-05	1.3E-02
chr1:3408899-3412949	4051	7.7	7.6	7.8	↓	-0.2	1.1E-04	3.3E-02
chr1:34177024-34177283	260	2.0	2.5	1.2	↑	1.4	2.5E-05	9.9E-03
chr1:35233508-35233836	329	1.9	1.2	2.3	↓	-1.2	3.5E-04	7.0E-02
chr1:35708127-35708531	405	3.3	2.9	3.7	↓	-0.8	2.1E-04	5.0E-02
chr1:3664185-3665104	920	5.4	5.3	5.6	↓	-0.3	3.8E-04	7.4E-02
chr1:37117401-37118167	767	3.3	2.8	3.6	↓	-0.8	1.6E-04	4.3E-02
chr1:38618336-38619359	1024	3.3	2.8	3.6	↓	-0.8	8.6E-05	2.7E-02
chr1:38650707-38651204	498	3.5	3.0	3.8	↓	-0.8	4.4E-05	1.5E-02
chr1:38771211-38772509	1299	4.5	4.2	4.7	↓	-0.5	3.8E-05	1.4E-02
chr1:387923-388477	555	4.7	4.4	4.9	↓	-0.5	1.3E-04	3.7E-02
chr1:40569921-40570412	492	2.3	1.7	2.7	↓	-1.0	6.2E-04	9.9E-02
chr1:4177406-4178081	676	4.6	4.1	4.9	↓	-0.8	1.7E-08	2.4E-05
chr1:4184374-4185352	979	4.0	3.7	4.3	↓	-0.5	4.1E-04	7.7E-02
chr1:42074231-42074810	580	4.4	4.0	4.7	↓	-0.7	1.8E-04	4.5E-02
chr1:45249260-45249621	362	2.4	1.8	2.9	↓	-1.1	5.4E-04	9.2E-02
chr1:45426746-45427469	724	2.7	3.1	2.2	↑	0.9	3.7E-04	7.4E-02
chr1:45947457-45947711	255	2.2	1.5	2.7	↓	-1.1	1.5E-04	4.1E-02
chr1:4670822-4672685	1864	6.5	6.3	6.7	↓	-0.4	8.7E-09	1.4E-05
chr1:47820983-47821591	609	2.9	2.3	3.3	↓	-1.0	1.1E-04	3.3E-02
chr1:4816140-4817223	1084	4.5	4.1	4.8	↓	-0.7	1.2E-05	6.1E-03
chr1:48513733-48514802	1070	4.2	3.9	4.5	↓	-0.6	1.0E-04	3.0E-02
chr1:49337284-49337510	227	2.0	1.0	2.5	↓	-1.5	7.3E-06	4.2E-03
chr1:502210-502653	444	4.3	3.7	4.6	↓	-0.9	2.2E-09	4.7E-06
chr1:507934-508534	601	4.9	4.6	5.1	↓	-0.5	5.6E-05	1.9E-02
chr1:52880133-52880524	392	2.0	2.4	1.4	↑	1.1	5.0E-04	8.8E-02
chr1:560555-563468	2914	7.5	7.4	7.7	↓	-0.3	5.7E-05	1.9E-02
chr1:57849283-57849598	316	2.3	1.8	2.7	↓	-1.0	5.5E-04	9.3E-02
chr1:58923010-58923513	504	3.1	3.5	2.5	↑	1.0	4.4E-06	2.8E-03
chr1:58988929-58990365	1437	5.1	5.4	4.7	↑	0.7	4.5E-07	4.0E-04
chr1:6011974-6012905	932	5.3	5.1	5.5	↓	-0.4	5.6E-04	9.4E-02
chr1:60327316-60328977	1662	5.6	5.9	5.3	↑	0.6	8.9E-06	4.8E-03
chr1:65051474-65052967	1494	4.3	4.5	4.0	↑	0.5	3.4E-04	7.0E-02
chr1:6532769-6533153	385	2.2	2.6	1.6	↑	1.1	6.2E-04	9.9E-02

chr1:6535966-6536391	426	2.2	2.7	1.6	↑	1.1	3.6E-04	7.2E-02
chr1:65479342-65479713	372	2.7	3.1	2.1	↑	0.9	2.5E-04	5.8E-02
chr1:7077449-7077943	495	2.6	3.0	2.0	↑	1.0	3.9E-04	7.4E-02
chr1:7331450-7332017	568	4.1	3.7	4.4	↓	-0.7	3.1E-04	6.6E-02
chr1:73389807-73390785	979	4.6	4.2	4.9	↓	-0.7	1.3E-06	1.0E-03
chr1:75632639-75633137	499	2.4	1.6	3.0	↓	-1.4	6.6E-07	5.4E-04
chr1:760663-761127	465	2.1	1.5	2.6	↓	-1.1	4.9E-04	8.7E-02
chr1:76884335-76884889	555	3.4	3.9	2.7	↑	1.2	1.3E-05	6.5E-03
chr1:83242899-83243416	518	3.1	2.6	3.4	↓	-0.8	5.6E-04	9.4E-02
chr1:8364257-8364665	409	3.0	2.3	3.5	↓	-1.2	3.7E-07	3.4E-04
chr1:83972591-83973167	577	2.8	2.3	3.3	↓	-1.0	1.5E-04	4.1E-02
chr1:84283679-84284484	806	3.9	3.2	4.4	↓	-1.1	2.0E-10	6.8E-07
chr1:84323207-84324622	1416	4.7	4.4	4.9	↓	-0.5	1.5E-04	4.1E-02
chr1:8619162-8619454	293	2.0	1.1	2.5	↓	-1.4	1.9E-05	7.9E-03
chr1:86703912-86704396	485	2.6	3.1	1.8	↑	1.3	1.9E-06	1.4E-03
chr1:87167420-87170288	2869	6.1	5.9	6.3	↓	-0.5	5.4E-04	9.3E-02
chr1:87192325-87192965	641	3.3	3.7	2.9	↑	0.8	2.6E-04	5.9E-02
chr1:87193-89842	2650	7.7	7.6	7.8	↓	-0.2	1.3E-04	3.7E-02
chr1:88080994-88081617	624	3.0	3.3	2.5	↑	0.8	2.3E-04	5.4E-02
chr1:89102728-89103434	707	4.6	4.2	5.0	↓	-0.9	6.5E-10	1.6E-06
chr1:89536078-89536731	654	4.0	3.7	4.3	↓	-0.6	6.2E-04	9.9E-02
chr1:92164880-92165408	529	3.0	3.4	2.5	↑	0.8	4.1E-04	7.7E-02
chr1:94007875-94008554	680	2.8	3.2	2.3	↑	0.8	6.3E-04	1.0E-01
chr1:9469418-9469959	542	3.0	3.4	2.5	↑	0.8	1.8E-04	4.5E-02
chr1:95089126-95092501	3376	6.3	6.6	6.0	↑	0.6	6.2E-11	2.4E-07
chr1:95604322-95604626	305	2.4	1.7	2.9	↓	-1.2	1.8E-05	7.9E-03
chr1:96966738-96967050	313	1.8	1.1	2.3	↓	-1.2	3.8E-04	7.4E-02
chr1:97166218-97166744	527	2.7	3.1	2.2	↑	0.9	4.3E-04	7.9E-02
chr1:97176947-97178807	1861	5.3	4.3	5.9	↓	-1.6	2.8E-53	1.8E-48
chr1:97179907-97180964	1058	4.0	2.6	4.7	↓	-2.0	7.6E-20	1.2E-15
chr1:97528117-97528918	802	3.3	2.8	3.6	↓	-0.8	1.3E-04	3.7E-02
chr1:97542772-97543559	788	3.1	2.5	3.5	↓	-1.0	9.1E-06	4.9E-03
chr1:97563730-97564016	287	2.0	1.3	2.5	↓	-1.2	2.7E-04	5.9E-02
chr1:97574440-97575281	842	3.4	2.6	3.9	↓	-1.3	7.6E-11	2.8E-07
chr1:97703748-97704039	292	2.3	1.4	2.8	↓	-1.4	5.0E-06	3.0E-03
chr1:98050120-98050380	261	1.8	2.3	1.1	↑	1.2	4.6E-04	8.3E-02
chr1:9845900-9846487	588	3.4	3.7	3.0	↑	0.7	4.8E-04	8.5E-02
chr1:99461207-99461582	376	2.3	1.6	2.8	↓	-1.2	6.5E-05	2.2E-02
chr10:10062911-10063227	317	3.5	3.1	3.8	↓	-0.7	1.7E-04	4.4E-02
chr10:10183468-10183752	285	2.1	2.5	1.5	↑	1.1	4.4E-04	8.1E-02
chr10:10632071-10633089	1019	5.1	4.8	5.3	↓	-0.5	4.1E-05	1.6E-02
chr10:10733490-10733951	462	3.8	3.2	4.2	↓	-1.0	3.9E-07	3.2E-04
chr10:10734546-10735261	716	4.7	5.0	4.3	↑	0.7	1.4E-06	1.0E-03
chr10:11017628-11017989	362	3.9	3.3	4.3	↓	-1.0	2.7E-07	2.5E-04
chr10:11322329-11322766	438	3.9	3.5	4.1	↓	-0.6	2.7E-04	5.8E-02
chr10:11363598-11364229	632	4.6	4.0	5.1	↓	-1.1	3.5E-16	2.4E-12
chr10:11862483-11864521	2039	6.8	6.6	6.9	↓	-0.3	3.0E-06	2.0E-03

chr10:12452836-12453407	572	3.3	3.7	2.9	↑	0.8	8.2E-05	2.8E-02
chr10:12579047-12579497	451	4.1	3.4	4.5	↓	-1.1	5.0E-11	1.4E-07
chr10:12640738-12641343	606	5.5	5.2	5.7	↓	-0.5	4.7E-04	8.5E-02
chr10:12820762-12821563	802	4.2	3.7	4.6	↓	-0.9	3.8E-07	3.2E-04
chr10:12890359-12890676	318	3.2	2.8	3.5	↓	-0.7	2.8E-04	6.1E-02
chr10:13935735-13936189	455	2.9	2.4	3.2	↓	-0.8	4.1E-04	7.9E-02
chr10:13937017-13937473	457	4.3	4.5	4.0	↑	0.5	1.1E-04	3.4E-02
chr10:14792830-14793427	598	4.5	4.0	4.9	↓	-0.9	3.6E-08	4.3E-05
chr10:15292279-15293196	918	4.8	4.5	5.1	↓	-0.6	8.0E-06	4.4E-03
chr10:15331814-15333183	1370	5.3	5.1	5.5	↓	-0.4	8.2E-05	2.8E-02
chr10:16084850-16085177	328	3.5	3.0	3.9	↓	-0.9	1.7E-05	7.9E-03
chr10:1635065-1635473	409	3.0	2.4	3.4	↓	-1.0	1.9E-04	4.6E-02
chr10:16659018-16659747	730	4.4	4.1	4.7	↓	-0.6	2.8E-04	6.0E-02
chr10:16739854-16740408	555	4.1	3.7	4.3	↓	-0.6	3.4E-04	7.0E-02
chr10:17191053-17191309	257	2.1	2.5	1.4	↑	1.1	3.4E-04	7.0E-02
chr10:17877111-17877618	508	3.4	2.9	3.7	↓	-0.8	9.4E-05	3.0E-02
chr10:19056316-19056742	427	3.4	3.0	3.7	↓	-0.7	5.6E-04	9.3E-02
chr10:19093032-19094384	1353	6.4	6.3	6.5	↓	-0.3	1.6E-04	4.2E-02
chr10:19446896-19447137	242	1.9	2.4	1.2	↑	1.2	3.9E-04	7.8E-02
chr10:20400980-20401480	501	4.1	3.7	4.4	↓	-0.7	6.6E-05	2.4E-02
chr10:21097558-21098605	1048	4.8	4.5	5.1	↓	-0.6	1.0E-05	5.3E-03
chr10:21099017-21100589	1573	5.8	5.6	6.1	↓	-0.5	1.4E-05	6.5E-03
chr10:21103872-21104283	412	3.4	2.9	3.7	↓	-0.8	8.6E-05	2.8E-02
chr10:2130956-2131422	467	4.1	3.8	4.4	↓	-0.7	1.8E-05	7.9E-03
chr10:21882139-21882439	301	2.2	2.8	1.1	↑	1.6	1.0E-07	1.1E-04
chr10:21884472-21884953	482	3.4	3.8	3.0	↑	0.8	1.3E-05	6.2E-03
chr10:22009477-22009911	435	3.6	3.1	3.9	↓	-0.8	1.1E-05	5.7E-03
chr10:22010284-22010598	315	2.9	2.2	3.4	↓	-1.3	2.8E-07	2.5E-04
chr10:22033416-22033741	326	2.4	2.8	1.8	↑	1.0	2.0E-04	4.8E-02
chr10:22243639-22244634	996	3.3	3.6	2.8	↑	0.8	2.8E-04	6.0E-02
chr10:22993630-22993848	219	2.2	1.5	2.7	↓	-1.2	3.0E-04	6.3E-02
chr10:23645531-23645794	264	3.1	2.6	3.4	↓	-0.8	3.0E-04	6.3E-02
chr10:23751898-23752530	633	5.1	4.4	5.5	↓	-1.1	1.5E-10	3.4E-07
chr10:24948217-24948500	284	3.1	2.4	3.6	↓	-1.2	1.9E-06	1.3E-03
chr10:24970673-24971206	534	4.8	4.5	5.0	↓	-0.5	4.3E-04	8.0E-02
chr10:25200326-25200700	375	3.4	3.7	3.0	↑	0.7	5.1E-04	8.8E-02
chr10:25283919-25284454	536	5.0	4.8	5.2	↓	-0.5	1.3E-04	3.7E-02
chr10:25479984-25480841	858	4.7	4.4	5.0	↓	-0.6	8.9E-06	4.6E-03
chr10:26257638-26257948	311	3.2	2.7	3.5	↓	-0.9	2.4E-04	5.5E-02
chr10:26680940-26681298	359	4.3	3.8	4.6	↓	-0.9	1.8E-06	1.2E-03
chr10:26732255-26732740	486	4.3	3.9	4.5	↓	-0.6	5.6E-04	9.4E-02
chr10:26738239-26738624	386	4.3	3.8	4.6	↓	-0.9	1.1E-07	1.1E-04
chr10:27123819-27124336	518	4.6	4.4	4.8	↓	-0.4	3.9E-04	7.7E-02
chr10:27585612-27586556	945	5.1	4.6	5.5	↓	-0.9	1.4E-12	4.7E-09
chr10:27923582-27924071	490	3.7	3.1	4.1	↓	-1.0	6.4E-07	5.0E-04
chr10:28389173-28389444	272	3.1	2.5	3.5	↓	-1.1	7.6E-05	2.6E-02
chr10:28617022-28617595	574	4.3	4.5	4.0	↑	0.5	4.1E-04	7.9E-02

chr10:28666165-28667254	1090	5.1	4.7	5.4	↓	-0.8	3.3E-08	4.1E-05
chr10:28758667-28760344	1678	5.9	6.1	5.7	↑	0.3	1.5E-04	4.0E-02
chr10:28840963-28841676	714	5.8	5.7	6.0	↓	-0.3	8.9E-05	2.9E-02
chr10:28842870-28843295	426	3.1	2.1	3.6	↓	-1.5	1.4E-10	3.4E-07
chr10:28843507-28843963	457	4.0	3.6	4.2	↓	-0.6	1.7E-04	4.5E-02
chr10:29275439-29275891	453	3.6	3.8	3.2	↑	0.6	5.0E-04	8.6E-02
chr10:29445514-29448853	3340	11.3	11.2	11.4	↓	-0.1	2.5E-05	1.1E-02
chr10:29510029-29510357	329	2.9	3.3	2.5	↑	0.8	3.7E-04	7.4E-02
chr10:30061866-30062223	358	2.7	2.1	3.1	↓	-0.9	1.4E-04	3.9E-02
chr10:30068432-30068852	421	2.7	2.1	3.1	↓	-1.1	6.5E-05	2.4E-02
chr10:30568923-30569341	419	2.6	3.0	2.0	↑	1.0	2.0E-04	4.8E-02
chr10:30806044-30806450	407	2.3	2.7	1.7	↑	1.0	4.2E-04	7.9E-02
chr10:31217655-31218599	945	4.8	4.4	5.1	↓	-0.6	1.0E-04	3.2E-02
chr10:31305405-31305949	545	3.6	3.2	3.9	↓	-0.8	2.2E-04	5.1E-02
chr10:31307468-31310241	2774	6.7	6.5	6.8	↓	-0.2	4.1E-04	7.9E-02
chr10:31734026-31734631	606	2.8	2.2	3.3	↓	-1.1	2.1E-04	4.9E-02
chr10:32181294-32181924	631	3.5	2.8	4.0	↓	-1.2	4.5E-08	5.2E-05
chr10:32189511-32190111	601	3.8	3.3	4.1	↓	-0.8	1.7E-04	4.5E-02
chr10:32634772-32634929	158	1.6	0.8	2.1	↓	-1.3	3.0E-04	6.3E-02
chr10:32809575-32811063	1489	6.6	6.3	6.9	↓	-0.7	2.1E-13	9.1E-10
chr10:33115024-33115457	434	3.4	2.7	3.9	↓	-1.2	1.4E-07	1.3E-04
chr10:3414912-3415819	908	6.7	6.6	6.9	↓	-0.3	8.8E-06	4.6E-03
chr10:34533641-34533872	232	2.9	2.4	3.3	↓	-0.9	4.4E-04	8.1E-02
chr10:3519463-3519962	500	3.7	4.0	3.3	↑	0.7	5.2E-04	8.8E-02
chr10:36008118-36008572	455	4.9	4.7	5.1	↓	-0.4	2.5E-04	5.7E-02
chr10:36334934-36335585	652	3.3	3.6	2.8	↑	0.7	2.7E-04	5.8E-02
chr10:36366633-36368534	1902	6.5	6.6	6.4	↑	0.2	3.4E-04	7.0E-02
chr10:42247282-42247480	199	2.2	1.4	2.7	↓	-1.3	7.0E-06	3.9E-03
chr10:44364008-44364265	258	2.0	2.5	1.3	↑	1.2	1.3E-04	3.7E-02
chr10:45254995-45255227	233	2.7	2.1	3.2	↓	-1.1	5.1E-04	8.8E-02
chr10:45387502-45387935	434	3.2	2.4	3.7	↓	-1.3	1.6E-06	1.1E-03
chr10:45390285-45390538	254	2.9	1.9	3.5	↓	-1.6	3.0E-09	4.7E-06
chr10:45404036-45406479	2444	6.2	5.6	6.6	↓	-1.0	9.7E-23	1.7E-18
chr10:45407390-45408962	1573	5.4	4.7	5.9	↓	-1.3	7.2E-18	8.3E-14
chr10:45414420-45414834	415	3.5	3.0	3.9	↓	-0.9	5.2E-05	2.0E-02
chr10:45416390-45417007	618	3.9	3.2	4.4	↓	-1.3	8.0E-12	2.5E-08
chr10:45418205-45419488	1284	6.1	5.9	6.4	↓	-0.5	3.6E-06	2.2E-03
chr10:45420106-45421593	1488	5.0	4.4	5.5	↓	-1.1	7.7E-15	3.8E-11
chr10:45421688-45422286	599	4.8	4.4	5.0	↓	-0.6	1.0E-04	3.2E-02
chr10:45422333-45422882	550	3.8	3.4	4.1	↓	-0.8	1.3E-05	6.1E-03
chr10:45895813-45896222	410	3.6	3.1	3.9	↓	-0.9	1.1E-04	3.3E-02
chr10:46636825-46637071	247	2.7	3.1	2.2	↑	0.9	2.8E-04	6.0E-02
chr10:47007400-47007721	322	2.2	1.6	2.7	↓	-1.1	1.1E-04	3.4E-02
chr10:47432927-47433347	421	2.5	1.9	2.9	↓	-1.0	2.5E-04	5.7E-02
chr10:47902348-47902729	382	2.7	3.1	2.1	↑	1.0	2.6E-04	5.8E-02
chr10:47913960-47914334	375	3.2	2.7	3.5	↓	-0.8	1.5E-04	4.0E-02
chr10:47914874-47915594	721	5.0	4.8	5.2	↓	-0.4	1.8E-04	4.6E-02

chr10:48730593-48730906	314	3.4	3.8	3.0	↑	0.8	8.3E-05	2.8E-02
chr10:5079565-5079978	414	3.4	3.0	3.8	↓	-0.7	1.8E-04	4.6E-02
chr10:52155931-52156124	194	1.7	0.9	2.2	↓	-1.4	1.2E-04	3.5E-02
chr10:52276314-52276653	340	3.9	4.2	3.7	↑	0.5	5.2E-04	8.8E-02
chr10:52468130-52468957	828	5.3	4.7	5.7	↓	-1.0	4.4E-09	6.7E-06
chr10:52642076-52642339	264	2.7	2.2	3.1	↓	-0.9	4.2E-04	7.9E-02
chr10:52723183-52723381	199	2.2	1.6	2.6	↓	-1.0	4.8E-04	8.6E-02
chr10:52767619-52767884	266	2.4	2.8	1.8	↑	1.1	1.9E-04	4.6E-02
chr10:5283539-5283997	459	4.4	4.1	4.7	↓	-0.5	3.2E-04	6.6E-02
chr10:53743202-53744150	949	5.3	5.6	4.9	↑	0.7	9.9E-09	1.4E-05
chr10:54071823-54072144	322	3.7	4.0	3.4	↑	0.7	5.1E-04	8.8E-02
chr10:56142221-56142855	635	3.7	3.2	4.0	↓	-0.8	1.7E-06	1.2E-03
chr10:56238304-56238586	283	2.6	2.0	3.1	↓	-1.1	6.9E-05	2.4E-02
chr10:57882522-57883006	485	3.2	2.8	3.5	↓	-0.8	2.4E-04	5.5E-02
chr10:58394687-58394890	204	2.0	2.4	1.3	↑	1.1	4.2E-04	7.9E-02
chr10:59231367-59232009	643	4.7	4.5	4.9	↓	-0.4	5.0E-04	8.6E-02
chr10:59566814-59567084	271	2.3	1.6	2.8	↓	-1.2	2.5E-05	1.1E-02
chr10:59603519-59603761	243	3.1	2.6	3.6	↓	-1.0	8.1E-06	4.4E-03
chr10:60756658-60757527	870	5.5	5.3	5.7	↓	-0.4	4.7E-05	1.8E-02
chr10:61592522-61592816	295	2.5	2.9	2.0	↑	0.9	5.7E-04	9.4E-02
chr10:61787126-61787378	253	2.4	2.8	1.8	↑	1.0	4.3E-04	7.9E-02
chr10:61932447-61932759	313	2.6	3.0	1.9	↑	1.2	4.2E-06	2.5E-03
chr10:62270854-62271209	356	3.6	3.3	3.9	↓	-0.6	4.9E-04	8.6E-02
chr10:62284828-62285764	937	5.7	5.0	6.2	↓	-1.2	1.4E-16	1.2E-12
chr10:62442946-62443534	589	4.2	3.5	4.6	↓	-1.1	7.5E-08	8.3E-05
chr10:62514792-62515949	1158	5.5	5.3	5.7	↓	-0.3	1.3E-04	3.7E-02
chr10:62684861-62685269	409	3.5	2.6	4.0	↓	-1.4	1.7E-11	4.8E-08
chr10:62770257-62772040	1784	5.7	5.3	5.9	↓	-0.6	2.1E-09	3.7E-06
chr10:62822977-62823190	214	1.7	0.8	2.3	↓	-1.4	6.3E-05	2.3E-02
chr10:62824442-62825726	1285	4.9	4.7	5.1	↓	-0.4	3.4E-04	6.9E-02
chr10:62878598-62879163	566	3.9	3.6	4.2	↓	-0.6	5.2E-04	8.8E-02
chr10:62927490-62927910	421	2.6	2.0	3.1	↓	-1.1	1.7E-04	4.4E-02
chr10:63090723-63091658	936	4.4	4.0	4.8	↓	-0.8	2.0E-09	3.7E-06
chr10:63091768-63092478	711	3.7	3.3	4.0	↓	-0.7	5.0E-05	2.0E-02
chr10:63123899-63124718	820	4.3	3.9	4.5	↓	-0.6	1.4E-05	6.7E-03
chr10:63175445-63176231	787	5.0	4.7	5.2	↓	-0.5	4.0E-05	1.6E-02
chr10:64242463-64242739	277	3.0	2.5	3.4	↓	-0.9	1.2E-04	3.5E-02
chr10:64636522-64636872	351	2.5	3.0	1.7	↑	1.3	2.6E-05	1.1E-02
chr10:64901254-64902155	902	3.9	4.1	3.5	↑	0.7	1.3E-04	3.7E-02
chr10:65121660-65122157	498	3.7	3.3	4.0	↓	-0.7	1.2E-04	3.5E-02
chr10:65424268-65425109	842	5.0	5.2	4.8	↑	0.4	2.1E-04	5.0E-02
chr10:65425918-65426504	587	4.6	4.2	4.9	↓	-0.7	3.3E-06	2.1E-03
chr10:6576629-6577099	471	3.6	3.1	3.9	↓	-0.8	1.9E-04	4.6E-02
chr10:6577159-6577535	377	2.7	2.2	3.1	↓	-0.9	3.7E-04	7.5E-02
chr10:6580671-6580996	326	3.9	3.4	4.3	↓	-0.9	3.4E-06	2.2E-03
chr10:65816023-65816865	843	5.6	5.4	5.7	↓	-0.3	4.1E-04	7.9E-02
chr10:65861950-65863323	1374	6.0	5.8	6.2	↓	-0.4	4.0E-05	1.6E-02

chr10:65863783-65865821	2039	6.5	6.3	6.7	↓	-0.5	2.9E-07	2.5E-04
chr10:65880257-65881895	1639	6.3	6.1	6.5	↓	-0.4	6.0E-06	3.5E-03
chr10:65884409-65885267	859	6.1	5.9	6.3	↓	-0.5	1.8E-04	4.5E-02
chr10:65887578-65888094	517	3.9	3.1	4.4	↓	-1.3	4.3E-13	1.7E-09
chr10:66721560-66721707	148	1.5	0.7	2.0	↓	-1.3	4.1E-04	7.9E-02
chr10:67999011-67999588	578	4.2	3.7	4.6	↓	-0.9	3.3E-08	4.1E-05
chr10:68225059-68225430	372	2.8	2.0	3.3	↓	-1.3	5.2E-07	4.2E-04
chr10:68231343-68231595	253	1.7	0.8	2.2	↓	-1.5	6.0E-05	2.3E-02
chr10:68323663-68324012	350	2.7	3.1	2.0	↑	1.1	6.4E-05	2.3E-02
chr10:69125868-69126375	508	4.0	3.5	4.4	↓	-1.0	3.6E-06	2.2E-03
chr10:69143428-69143851	424	3.2	2.4	3.6	↓	-1.2	1.2E-08	1.6E-05
chr10:69156751-69157132	382	2.7	2.1	3.1	↓	-1.0	1.2E-04	3.5E-02
chr10:69354455-69354862	408	4.2	3.7	4.6	↓	-0.9	2.7E-09	4.4E-06
chr10:69472857-69473380	524	3.7	3.4	4.0	↓	-0.7	4.9E-04	8.6E-02
chr10:69544440-69545914	1475	5.5	5.3	5.7	↓	-0.4	8.4E-05	2.8E-02
chr10:69653554-69654036	483	3.1	2.4	3.5	↓	-1.1	1.6E-05	7.5E-03
chr10:69815586-69816074	489	3.0	2.1	3.5	↓	-1.4	9.6E-08	1.0E-04
chr10:69842949-69844048	1100	4.7	4.1	5.1	↓	-1.0	4.9E-15	2.8E-11
chr10:69856203-69858094	1892	5.9	5.8	6.1	↓	-0.3	1.1E-04	3.4E-02
chr10:69858136-69858968	833	4.4	4.0	4.7	↓	-0.7	1.5E-04	4.1E-02
chr10:69898433-69898760	328	2.6	2.0	3.0	↓	-1.0	1.7E-04	4.4E-02
chr10:70220088-70220871	784	5.9	5.7	6.1	↓	-0.4	6.1E-06	3.5E-03
chr10:70340693-70341152	460	3.5	3.0	3.9	↓	-0.9	7.4E-07	5.7E-04
chr10:70405254-70405843	590	5.2	5.0	5.4	↓	-0.4	2.4E-04	5.4E-02
chr10:70551793-70552704	912	4.2	3.9	4.5	↓	-0.6	5.2E-05	2.0E-02
chr10:70874259-70876329	2071	6.6	6.4	6.7	↓	-0.3	4.4E-06	2.6E-03
chr10:71074500-71074804	305	2.3	1.5	2.8	↓	-1.3	1.2E-05	5.9E-03
chr10:71430323-71432217	1895	7.7	7.6	7.8	↓	-0.2	1.8E-04	4.6E-02
chr10:71434998-71436244	1247	4.7	4.5	5.0	↓	-0.5	1.1E-04	3.4E-02
chr10:71489243-71490860	1618	5.9	5.7	6.1	↓	-0.4	4.6E-05	1.8E-02
chr10:7235610-7235977	368	2.0	2.5	1.3	↑	1.2	2.6E-04	5.8E-02
chr10:72546225-72546761	537	3.7	3.3	4.0	↓	-0.7	2.0E-04	4.8E-02
chr10:72910283-72911250	968	4.2	3.7	4.6	↓	-0.9	1.9E-09	3.7E-06
chr10:72911350-72911724	375	4.2	3.8	4.5	↓	-0.7	3.7E-05	1.6E-02
chr10:73180279-73180774	496	2.6	2.0	3.0	↓	-1.0	1.9E-04	4.6E-02
chr10:73231694-73232223	530	4.9	4.4	5.2	↓	-0.8	6.3E-09	9.2E-06
chr10:7349397-7349683	287	3.0	2.5	3.4	↓	-0.9	4.2E-04	7.9E-02
chr10:73714593-73714970	378	3.5	3.1	3.8	↓	-0.7	1.2E-04	3.5E-02
chr10:73786723-73787159	437	4.0	3.6	4.3	↓	-0.7	2.6E-06	1.7E-03
chr10:73798440-73799125	686	4.7	4.4	4.9	↓	-0.5	5.8E-04	9.5E-02
chr10:73869504-73870596	1093	5.0	4.8	5.2	↓	-0.4	5.8E-04	9.5E-02
chr10:73876630-73877039	410	4.3	3.9	4.6	↓	-0.8	8.4E-06	4.5E-03
chr10:73879812-73880521	710	5.6	5.2	6.0	↓	-0.8	5.8E-10	1.3E-06
chr10:73880979-73881511	533	4.6	4.2	4.9	↓	-0.7	7.8E-07	5.9E-04
chr10:74062504-74062983	480	3.0	2.5	3.3	↓	-0.8	4.9E-04	8.6E-02
chr10:74260443-74261975	1533	5.6	5.4	5.8	↓	-0.4	4.7E-04	8.5E-02
chr10:74637093-74637635	543	3.1	2.4	3.5	↓	-1.1	2.0E-07	1.9E-04

chr10:74781106-74783070	1965	6.7	6.6	6.8	↓	-0.2	4.7E-04	8.5E-02
chr10:74784834-74785122	289	2.5	1.7	3.0	↓	-1.2	1.6E-05	7.4E-03
chr10:74940948-74941713	766	5.4	5.3	5.6	↓	-0.4	1.2E-04	3.5E-02
chr10:75056819-75057271	453	3.3	2.8	3.7	↓	-0.9	2.6E-05	1.1E-02
chr10:76436309-76436979	671	5.3	5.0	5.6	↓	-0.6	4.4E-04	8.1E-02
chr10:8056775-8057498	724	5.3	5.1	5.6	↓	-0.5	1.1E-07	1.1E-04
chr10:8079895-8080178	284	2.9	2.4	3.3	↓	-0.8	2.2E-04	5.2E-02
chr10:8644797-8645156	360	4.7	3.9	5.2	↓	-1.3	1.1E-23	3.6E-19
chr10:8665951-8666552	602	3.6	3.2	4.0	↓	-0.7	1.1E-04	3.4E-02
chr10:8672911-8673218	308	3.2	2.3	3.8	↓	-1.5	8.0E-10	1.6E-06
chr10:8841845-8842451	607	4.0	3.6	4.3	↓	-0.7	7.6E-05	2.6E-02
chr10:8938311-8938880	570	4.0	3.7	4.3	↓	-0.6	5.7E-05	2.1E-02
chr10:9377962-9378219	258	2.4	1.8	2.8	↓	-0.9	4.9E-04	8.6E-02
chr10:9430682-9431034	353	2.0	2.5	1.4	↑	1.1	3.9E-04	7.7E-02
chr11:10182940-10183292	353	2.5	2.9	2.0	↑	1.0	3.7E-04	5.8E-02
chr11:10189766-10190100	335	3.8	4.1	3.3	↑	0.9	2.5E-06	1.6E-03
chr11:10190337-10191342	1006	5.2	5.0	5.4	↓	-0.4	2.9E-04	5.0E-02
chr11:10326962-10327375	414	3.9	3.3	4.2	↓	-0.9	3.0E-07	3.2E-04
chr11:1065092-1066149	1058	5.4	5.1	5.6	↓	-0.4	4.9E-06	2.5E-03
chr11:10791415-10792265	851	4.8	4.5	5.1	↓	-0.5	1.4E-05	5.4E-03
chr11:1128228-1132028	3801	8.2	8.2	8.3	↓	-0.1	2.2E-04	4.2E-02
chr11:11606657-11607624	968	4.6	4.4	4.8	↓	-0.4	5.8E-04	7.7E-02
chr11:11609078-11609517	440	2.3	2.7	1.6	↑	1.1	3.0E-04	5.0E-02
chr11:12030227-12030570	344	2.8	3.2	2.2	↑	0.9	3.3E-04	5.3E-02
chr11:12336194-12336416	223	2.5	1.8	3.0	↓	-1.2	8.4E-06	3.6E-03
chr11:1289199-1290054	856	5.1	4.8	5.3	↓	-0.5	3.2E-05	1.0E-02
chr11:13803605-13804044	440	4.0	3.5	4.3	↓	-0.9	1.5E-07	1.9E-04
chr11:14107961-14108547	587	4.4	4.2	4.7	↓	-0.5	6.5E-04	8.4E-02
chr11:14204067-14204305	239	1.9	1.1	2.4	↓	-1.2	1.4E-04	2.8E-02
chr11:14636475-14637063	589	4.8	4.5	5.0	↓	-0.6	6.2E-05	1.7E-02
chr11:15253998-15254317	320	2.9	3.2	2.4	↑	0.8	6.7E-04	8.6E-02
chr11:15304561-15307335	2775	7.1	7.0	7.2	↓	-0.2	4.7E-04	6.7E-02
chr11:15553130-15553772	643	4.2	3.9	4.5	↓	-0.6	3.5E-05	1.1E-02
chr11:16561457-16562481	1025	4.6	4.9	4.4	↑	0.5	6.2E-05	1.7E-02
chr11:16675009-16675421	413	2.5	2.9	1.9	↑	1.0	3.2E-04	5.3E-02
chr11:18000308-18000736	429	3.6	3.9	3.2	↑	0.7	2.0E-04	3.9E-02
chr11:18193564-18193922	359	3.0	3.4	2.5	↑	0.9	6.9E-05	1.8E-02
chr11:19105926-19106318	393	2.5	1.8	3.0	↓	-1.3	2.2E-06	1.4E-03
chr11:19353702-19354008	307	3.0	2.5	3.4	↓	-0.8	2.2E-04	4.1E-02
chr11:1941605-1942227	623	3.8	3.4	4.2	↓	-0.8	6.2E-06	2.9E-03
chr11:19447885-19449181	1297	5.1	4.9	5.3	↓	-0.4	1.3E-04	2.7E-02
chr11:19747851-19748324	474	3.8	3.4	4.1	↓	-0.7	1.3E-04	2.7E-02
chr11:20055690-20056083	394	3.8	3.0	4.3	↓	-1.3	3.6E-12	2.4E-08
chr11:20223526-20223817	292	3.5	3.0	3.9	↓	-0.9	2.6E-06	1.6E-03
chr11:20389626-20390520	895	4.3	4.0	4.6	↓	-0.7	2.2E-06	1.4E-03
chr11:20981271-20981766	496	2.9	2.4	3.3	↓	-0.9	1.1E-04	2.5E-02
chr11:21055942-21058214	2273	5.7	6.0	5.5	↑	0.4	1.2E-06	9.0E-04

chr11:21066561-21066909	349	3.7	4.1	3.2	↑	0.9	7.3E-07	6.1E-04
chr11:21069934-21071423	1490	5.2	5.5	5.0	↑	0.5	4.9E-06	2.5E-03
chr11:21079348-21079761	414	2.8	3.1	2.3	↑	0.8	7.4E-04	9.3E-02
chr11:21143478-21143697	220	2.2	2.6	1.5	↑	1.1	4.6E-04	6.6E-02
chr11:21972074-21972637	564	3.9	3.4	4.2	↓	-0.8	6.2E-05	1.7E-02
chr11:22072253-22072705	453	2.5	2.0	2.9	↓	-1.0	2.7E-04	4.8E-02
chr11:2210460-2212110	1651	5.4	5.1	5.7	↓	-0.5	2.1E-06	1.4E-03
chr11:22110750-22111330	581	2.9	3.3	2.4	↑	0.9	2.0E-04	3.8E-02
chr11:2276228-2276425	198	2.0	2.5	1.1	↑	1.4	7.7E-06	3.4E-03
chr11:236787-237092	306	2.1	2.5	1.4	↑	1.1	5.6E-04	7.7E-02
chr11:23815302-23815829	528	4.8	4.6	5.0	↓	-0.4	5.7E-04	7.7E-02
chr11:23862440-23863525	1086	5.9	5.5	6.1	↓	-0.6	5.4E-09	1.1E-05
chr11:23870994-23871691	698	4.5	4.2	4.7	↓	-0.5	1.3E-04	2.8E-02
chr11:24014073-24014381	309	3.9	3.5	4.2	↓	-0.8	4.9E-06	2.5E-03
chr11:24102098-24103100	1003	4.4	4.0	4.6	↓	-0.6	5.4E-05	1.5E-02
chr11:2413598-2415419	1822	6.4	6.2	6.5	↓	-0.3	3.6E-06	2.1E-03
chr11:24143698-24144608	911	4.9	4.5	5.2	↓	-0.8	2.6E-11	9.7E-08
chr11:25106335-25106774	440	2.9	3.3	2.4	↑	0.9	2.0E-04	3.9E-02
chr11:25250853-25251394	542	2.8	3.2	2.3	↑	0.9	2.9E-04	5.0E-02
chr11:25345307-25346652	1346	5.7	5.5	5.9	↓	-0.3	3.0E-04	5.0E-02
chr11:26006711-26006998	288	2.7	2.2	3.1	↓	-0.9	3.9E-04	6.1E-02
chr11:26023842-26024719	878	3.8	3.5	4.1	↓	-0.6	1.6E-04	3.2E-02
chr11:2661663-2663081	1419	6.0	5.8	6.2	↓	-0.3	1.8E-04	3.5E-02
chr11:26759919-26760591	673	3.5	3.1	3.8	↓	-0.7	3.1E-04	5.1E-02
chr11:27324552-27324915	364	2.6	2.1	3.0	↓	-0.9	6.6E-04	8.6E-02
chr11:2810808-2811920	1113	5.1	4.8	5.3	↓	-0.5	2.8E-06	1.6E-03
chr11:28153562-28154449	888	5.3	5.1	5.5	↓	-0.4	5.2E-04	7.2E-02
chr11:2845891-2847442	1552	5.7	5.5	5.9	↓	-0.4	1.8E-06	1.3E-03
chr11:29017852-29018161	310	2.3	1.7	2.7	↓	-1.0	3.9E-04	6.1E-02
chr11:2908956-2909288	333	3.0	2.4	3.4	↓	-1.0	1.1E-05	4.5E-03
chr11:2966217-2966971	755	3.3	2.9	3.6	↓	-0.7	3.5E-04	5.6E-02
chr11:298627-299139	513	3.7	3.1	4.1	↓	-1.0	1.6E-07	2.0E-04
chr11:299499-301047	1549	7.4	7.2	7.6	↓	-0.4	2.8E-06	1.6E-03
chr11:3089567-3091499	1933	6.5	6.2	6.7	↓	-0.5	2.2E-08	3.7E-05
chr11:3098627-3098994	368	2.4	1.9	2.8	↓	-0.9	7.0E-04	9.0E-02
chr11:32001130-32001709	580	2.6	2.0	3.1	↓	-1.0	5.8E-04	7.7E-02
chr11:32002392-32003328	937	3.7	3.1	4.1	↓	-1.0	8.0E-07	6.4E-04
chr11:32513536-32513801	266	2.7	2.2	3.1	↓	-1.0	1.3E-04	2.7E-02
chr11:3337847-3338244	398	3.2	2.7	3.5	↓	-0.8	7.4E-04	9.3E-02
chr11:3354140-3354527	388	3.0	2.5	3.4	↓	-0.9	7.4E-04	9.3E-02
chr11:33808229-33808595	367	2.3	1.5	2.8	↓	-1.3	3.9E-06	2.2E-03
chr11:33858954-33860520	1567	5.4	5.1	5.7	↓	-0.6	1.3E-05	5.1E-03
chr11:33862865-33863863	999	5.0	4.6	5.3	↓	-0.6	2.2E-05	7.5E-03
chr11:33881630-33882040	411	3.3	2.9	3.6	↓	-0.8	2.3E-04	4.3E-02
chr11:3909501-3910088	588	3.6	3.2	3.9	↓	-0.8	4.7E-05	1.4E-02
chr11:4007746-4008461	716	4.4	4.2	4.6	↓	-0.5	4.9E-04	6.9E-02
chr11:417715-418457	743	3.9	3.5	4.2	↓	-0.7	2.8E-04	4.9E-02

chr11:4236076-4236465	390	3.7	3.3	4.0	↓	-0.7	4.4E-04	6.5E-02
chr11:43015353-43015695	343	2.5	1.9	2.9	↓	-1.0	1.1E-04	2.5E-02
chr11:430757-431523	767	5.4	5.3	5.6	↓	-0.3	5.7E-04	7.7E-02
chr11:48445169-48445526	358	2.4	1.6	2.9	↓	-1.3	1.2E-06	9.0E-04
chr11:49236516-49236902	387	4.4	4.1	4.6	↓	-0.5	1.2E-04	2.6E-02
chr11:50717791-50718333	543	4.9	4.5	5.1	↓	-0.6	1.2E-05	4.9E-03
chr11:52066690-52067796	1107	4.1	4.5	3.5	↑	1.0	5.8E-06	2.8E-03
chr11:53150025-53150387	363	2.3	2.7	1.6	↑	1.1	2.9E-04	5.0E-02
chr11:5332936-5333426	491	3.8	4.2	3.2	↑	1.0	9.7E-09	1.7E-05
chr11:5341859-5343590	1732	6.1	6.2	6.0	↑	0.3	5.8E-04	7.7E-02
chr11:53522999-53523325	327	3.4	2.9	3.8	↓	-0.8	1.4E-04	2.9E-02
chr11:53596937-53597487	551	2.6	2.1	3.0	↓	-0.9	5.7E-04	7.7E-02
chr11:54086172-54086435	264	1.8	0.9	2.4	↓	-1.4	6.9E-05	1.8E-02
chr11:55560872-55561284	413	5.1	4.8	5.4	↓	-0.6	1.6E-05	5.9E-03
chr11:55831796-55832335	540	3.3	2.8	3.7	↓	-0.9	8.4E-05	2.0E-02
chr11:56495017-56495661	645	4.5	4.1	4.7	↓	-0.6	5.1E-06	2.6E-03
chr11:5661169-5661894	726	4.7	4.3	5.0	↓	-0.8	9.3E-07	7.2E-04
chr11:5737912-5738554	643	5.3	5.0	5.5	↓	-0.5	1.1E-04	2.5E-02
chr11:57509289-57509694	406	3.4	3.0	3.7	↓	-0.7	2.3E-04	4.3E-02
chr11:57529098-57529285	188	1.7	0.5	2.3	↓	-1.9	3.3E-07	3.4E-04
chr11:5784199-5785238	1040	5.3	4.8	5.6	↓	-0.8	2.0E-12	1.8E-08
chr11:5886174-5887454	1281	6.0	5.8	6.1	↓	-0.3	5.3E-04	7.4E-02
chr11:597493-598397	905	5.2	5.4	5.0	↑	0.4	3.3E-04	5.3E-02
chr11:6003105-6003541	437	4.5	4.2	4.8	↓	-0.6	7.4E-06	3.4E-03
chr11:60393474-60393753	280	2.1	2.6	1.4	↑	1.2	5.6E-05	1.6E-02
chr11:6073489-6073878	390	2.5	1.9	2.9	↓	-0.9	3.3E-04	5.3E-02
chr11:6105830-6107855	2026	6.2	5.9	6.5	↓	-0.5	4.6E-11	1.3E-07
chr11:61676729-61677001	273	2.2	2.7	1.7	↑	1.0	4.7E-04	6.7E-02
chr11:643365-644658	1294	6.2	5.9	6.5	↓	-0.6	3.8E-07	3.8E-04
chr11:65488118-65489107	990	5.2	5.0	5.4	↓	-0.4	6.4E-04	8.4E-02
chr11:65630688-65630902	215	1.8	0.8	2.3	↓	-1.5	2.0E-05	7.4E-03
chr11:6575599-6575900	302	2.5	1.8	3.0	↓	-1.2	1.5E-05	5.8E-03
chr11:66216846-66217057	212	2.1	1.5	2.6	↓	-1.0	7.5E-04	9.4E-02
chr11:6635636-6637193	1558	6.5	6.3	6.6	↓	-0.3	7.6E-05	1.9E-02
chr11:66578852-66579158	307	2.9	2.3	3.2	↓	-0.9	2.4E-04	4.4E-02
chr11:6836833-6837405	573	4.8	4.3	5.1	↓	-0.8	7.4E-07	6.1E-04
chr11:69087957-69088280	324	3.1	2.5	3.5	↓	-1.0	7.4E-06	3.4E-03
chr11:69088418-69088812	395	3.0	2.5	3.4	↓	-0.9	4.8E-05	1.4E-02
chr11:6920955-6921286	332	3.1	2.3	3.6	↓	-1.3	3.6E-08	5.5E-05
chr11:6922462-6923323	862	5.2	4.9	5.4	↓	-0.5	4.4E-04	6.5E-02
chr11:69397621-69398090	470	2.4	2.9	1.8	↑	1.1	9.4E-05	2.2E-02
chr11:69958098-69958942	845	4.5	4.2	4.8	↓	-0.5	2.4E-04	4.4E-02
chr11:70457442-70457809	368	2.6	2.1	3.0	↓	-0.9	3.2E-04	5.3E-02
chr11:72413155-72413469	315	2.4	1.8	2.8	↓	-0.9	5.7E-04	7.7E-02
chr11:73194409-73195095	687	4.3	3.9	4.6	↓	-0.7	3.7E-06	2.1E-03
chr11:7323442-7324317	876	5.2	4.9	5.4	↓	-0.5	2.1E-05	7.4E-03
chr11:7364250-7364498	249	2.3	1.8	2.7	↓	-1.0	7.8E-04	9.6E-02

chr11:73907227-73907597	371	2.3	2.7	1.8	↑	0.9	6.3E-04	8.2E-02
chr11:7391431-7391902	472	5.4	5.2	5.7	↓	-0.5	4.8E-07	4.5E-04
chr11:73940194-73941102	909	4.5	4.2	4.8	↓	-0.6	2.6E-05	8.7E-03
chr11:7431879-7432146	268	2.1	2.5	1.4	↑	1.1	4.4E-04	6.5E-02
chr11:74900541-74900929	389	3.6	3.2	3.9	↓	-0.7	1.1E-04	2.5E-02
chr11:75621908-75622499	592	5.2	5.0	5.5	↓	-0.5	2.8E-04	4.9E-02
chr11:75891520-75892101	582	4.5	4.1	4.8	↓	-0.6	1.3E-05	5.1E-03
chr11:75985174-75985794	621	3.7	3.9	3.3	↑	0.6	4.2E-04	6.3E-02
chr11:76031817-76032349	533	3.7	3.3	3.9	↓	-0.7	3.7E-04	5.8E-02
chr11:76372793-76373270	478	3.4	2.7	3.9	↓	-1.2	6.1E-09	1.2E-05
chr11:76490273-76490615	343	1.9	0.9	2.5	↓	-1.5	8.0E-06	3.5E-03
chr11:76541875-76544594	2720	7.6	7.7	7.5	↑	0.2	5.0E-05	1.4E-02
chr11:76722019-76722487	469	3.5	3.1	3.8	↓	-0.7	1.1E-04	2.5E-02
chr11:76752191-76752917	727	4.9	4.6	5.1	↓	-0.6	4.9E-06	2.5E-03
chr11:76831709-76832371	663	5.5	5.3	5.7	↓	-0.4	3.4E-05	1.1E-02
chr11:77021483-77022000	518	6.0	5.8	6.1	↓	-0.3	3.7E-04	5.8E-02
chr11:771615-773418	1804	7.1	7.0	7.2	↓	-0.2	6.7E-05	1.8E-02
chr11:78172767-78173124	358	2.6	2.0	3.0	↓	-1.1	3.5E-05	1.1E-02
chr11:78912542-78912967	426	3.6	3.0	4.0	↓	-1.0	3.7E-08	5.5E-05
chr11:78934470-78934697	228	1.5	0.7	2.1	↓	-1.3	5.1E-04	7.1E-02
chr11:7899772-7901198	1427	5.2	5.5	4.9	↑	0.6	2.8E-05	9.1E-03
chr11:79399019-79399922	904	5.3	4.9	5.6	↓	-0.7	1.9E-11	8.4E-08
chr11:79700849-79701161	313	2.2	1.5	2.7	↓	-1.2	2.6E-05	8.7E-03
chr11:8017630-8018635	1006	4.5	4.7	4.3	↑	0.5	1.8E-04	3.5E-02
chr11:80632132-80632784	653	3.7	3.4	4.0	↓	-0.7	2.8E-04	4.9E-02
chr11:80673845-80674408	564	4.7	4.4	5.0	↓	-0.6	5.4E-06	2.6E-03
chr11:8111200-8111491	292	3.0	2.5	3.4	↓	-1.0	8.5E-05	2.0E-02
chr11:81358646-81358910	265	1.7	2.2	1.0	↑	1.2	7.3E-04	9.3E-02
chr11:81411081-81411853	773	4.5	4.2	4.7	↓	-0.5	6.8E-04	8.8E-02
chr11:81500192-81501172	981	5.1	5.3	4.8	↑	0.4	2.8E-05	9.1E-03
chr11:81504876-81505802	927	4.7	4.5	5.0	↓	-0.5	4.2E-04	6.4E-02
chr11:81507469-81508219	751	4.2	3.9	4.5	↓	-0.6	4.6E-04	6.7E-02
chr11:81508291-81508764	474	2.6	1.8	3.1	↓	-1.3	6.6E-07	5.9E-04
chr11:82008162-82009648	1487	5.4	5.1	5.6	↓	-0.5	1.4E-04	2.8E-02
chr11:82165581-82165934	354	2.7	2.2	3.1	↓	-0.9	1.2E-04	2.6E-02
chr11:82192448-82192955	508	3.6	3.9	3.2	↑	0.7	2.1E-04	4.0E-02
chr11:82193576-82194221	646	4.3	3.7	4.7	↓	-1.0	6.9E-11	1.8E-07
chr11:82205811-82206717	907	5.1	5.3	4.9	↑	0.4	7.8E-04	9.6E-02
chr11:82315549-82316096	548	2.9	2.4	3.2	↓	-0.8	4.2E-04	6.3E-02
chr11:82354487-82355092	606	4.0	3.6	4.4	↓	-0.8	1.0E-05	4.2E-03
chr11:82468407-82468703	297	3.1	2.5	3.4	↓	-0.9	6.4E-05	1.7E-02
chr11:82490829-82491546	718	4.1	3.8	4.4	↓	-0.6	5.0E-04	7.0E-02
chr11:829193-830991	1799	7.5	7.3	7.6	↓	-0.4	3.8E-11	1.3E-07
chr11:83000153-83000608	456	5.1	4.4	5.6	↓	-1.2	1.2E-22	3.3E-18
chr11:83095147-83095489	343	2.4	1.9	2.8	↓	-0.9	7.2E-04	9.1E-02
chr11:83595062-83595497	436	3.2	2.8	3.5	↓	-0.7	4.7E-04	6.7E-02
chr11:83731442-83732498	1057	4.7	4.4	5.0	↓	-0.6	2.1E-06	1.4E-03

chr11:83822327-83823014	688	4.8	4.4	5.1	↓	-0.7	1.9E-07	2.2E-04
chr11:83824875-83825524	650	4.6	3.9	5.0	↓	-1.1	5.9E-12	3.1E-08
chr11:83836261-83838404	2144	6.4	6.1	6.6	↓	-0.5	1.5E-12	1.8E-08
chr11:83963320-83964658	1339	5.3	5.1	5.5	↓	-0.4	3.3E-04	5.3E-02
chr11:83980135-83980840	706	4.6	4.3	4.8	↓	-0.5	1.9E-04	3.7E-02
chr11:83985669-83986732	1064	5.6	5.4	5.7	↓	-0.3	1.5E-04	3.0E-02
chr11:83986882-83987244	363	3.2	2.7	3.6	↓	-1.0	6.9E-05	1.8E-02
chr11:84078191-84078664	474	4.8	4.5	5.0	↓	-0.5	2.1E-04	4.0E-02
chr11:84089901-84091163	1263	4.5	4.7	4.1	↑	0.6	2.6E-05	8.7E-03
chr11:84100054-84100361	308	2.7	3.2	2.1	↑	1.0	4.0E-05	1.2E-02
chr11:84106955-84107522	568	3.9	3.5	4.2	↓	-0.7	3.0E-04	5.0E-02
chr11:84147476-84148248	773	5.2	4.8	5.4	↓	-0.6	4.4E-08	6.2E-05
chr11:84370773-84371754	982	5.5	5.2	5.7	↓	-0.5	1.0E-04	2.3E-02
chr11:84605177-84606203	1027	5.3	5.0	5.6	↓	-0.6	6.7E-07	5.9E-04
chr11:84606347-84608141	1795	6.6	6.5	6.7	↓	-0.3	7.9E-04	9.7E-02
chr11:84869167-84869995	829	6.0	5.8	6.1	↓	-0.3	8.0E-05	2.0E-02
chr11:85028649-85031814	3166	7.5	7.3	7.6	↓	-0.3	5.5E-08	7.3E-05
chr11:85069823-85073335	3513	7.7	7.6	7.8	↓	-0.2	1.8E-05	6.8E-03
chr11:85164114-85168323	4210	8.1	8.0	8.2	↓	-0.2	1.3E-05	5.1E-03
chr11:85302691-85303659	969	5.9	5.6	6.1	↓	-0.4	4.2E-05	1.2E-02
chr11:85956922-85957565	644	4.5	4.1	4.9	↓	-0.8	2.3E-10	5.5E-07
chr11:86029607-86036027	6421	9.0	8.9	9.1	↓	-0.1	4.7E-04	6.7E-02
chr11:86037229-86038274	1046	5.5	5.2	5.7	↓	-0.5	2.9E-05	9.1E-03
chr11:86257853-86263224	5372	8.7	8.6	8.8	↓	-0.2	1.0E-04	2.4E-02
chr11:86432630-86434705	2076	7.2	7.1	7.3	↓	-0.2	2.1E-05	7.4E-03
chr11:86541930-86545080	3151	7.0	7.1	6.9	↑	0.2	2.3E-04	4.2E-02
chr11:86643596-86644229	634	4.5	4.2	4.7	↓	-0.5	1.0E-04	2.3E-02
chr11:86644960-86645790	831	4.7	5.0	4.4	↑	0.6	2.4E-05	8.1E-03
chr11:86755504-86755889	386	2.1	2.6	1.5	↑	1.1	2.9E-04	5.0E-02
chr11:87539518-87540628	1111	5.9	5.8	6.1	↓	-0.3	4.1E-04	6.3E-02
chr11:87624601-87626454	1854	5.4	5.2	5.6	↓	-0.3	5.7E-04	7.7E-02
chr11:880504-880872	369	3.0	2.4	3.3	↓	-0.9	4.4E-05	1.3E-02
chr11:9617092-9617368	277	2.1	1.5	2.6	↓	-1.1	2.5E-04	4.5E-02
chr11:9665632-9666359	728	4.3	3.7	4.6	↓	-0.9	2.5E-09	5.4E-06
chr11:9776771-9777173	403	2.5	1.9	2.9	↓	-0.9	3.9E-04	6.1E-02
chr11:9779595-9780120	526	4.5	4.1	4.9	↓	-0.7	8.3E-06	3.6E-03
chr11:9791302-9791543	242	1.9	1.2	2.4	↓	-1.3	8.2E-05	2.0E-02
chr11:9797319-9797852	534	3.5	2.9	3.9	↓	-1.0	2.8E-07	3.1E-04
chr11:9828482-9829243	762	4.3	4.0	4.6	↓	-0.6	8.9E-05	2.1E-02
chr11:9833596-9833818	223	2.1	1.5	2.5	↓	-1.1	4.3E-04	6.4E-02
chr11:9850116-9850681	566	2.8	2.2	3.2	↓	-1.0	6.5E-05	1.7E-02
chr11:9968758-9969034	277	2.2	1.5	2.7	↓	-1.2	7.3E-05	1.8E-02
chr12:10256649-10256748	100	1.3	1.8	0.5	↑	1.3	1.1E-03	9.7E-02
chr12:10520790-10520943	154	1.9	1.3	2.4	↓	-1.1	6.3E-04	6.7E-02
chr12:10610930-10611288	359	3.1	2.6	3.5	↓	-0.9	4.8E-05	9.1E-03
chr12:10611337-10611946	610	4.7	4.4	4.9	↓	-0.5	4.8E-05	9.1E-03
chr12:10623898-10624072	175	2.8	2.3	3.2	↓	-0.9	6.6E-04	6.9E-02

chr12:11005874-11006252	379	2.9	3.3	2.5	↑	0.8	9.1E-04	8.7E-02
chr12:11038048-11038627	580	3.7	3.3	3.9	↓	-0.6	1.1E-03	9.8E-02
chr12:11320323-11320459	137	1.6	0.9	2.1	↓	-1.3	5.1E-04	5.8E-02
chr12:12031907-12032248	342	2.5	2.9	1.9	↑	0.9	7.4E-04	7.5E-02
chr12:12112418-12112658	241	2.9	2.4	3.3	↓	-0.8	6.2E-04	6.6E-02
chr12:12164191-12164496	306	3.1	2.7	3.5	↓	-0.8	6.3E-04	6.7E-02
chr12:1225403-1226074	672	5.9	5.7	6.1	↓	-0.4	3.9E-06	1.2E-03
chr12:1227700-1228324	625	4.8	4.5	5.1	↓	-0.6	1.4E-04	2.1E-02
chr12:12309230-12309481	252	2.4	1.8	2.8	↓	-1.0	1.3E-04	2.0E-02
chr12:12309626-12310287	662	5.3	5.1	5.5	↓	-0.3	5.2E-04	5.8E-02
chr12:1232202-1232632	431	3.7	3.2	4.1	↓	-0.8	9.6E-05	1.6E-02
chr12:1232873-1233436	564	4.2	3.6	4.6	↓	-1.0	2.4E-09	2.0E-06
chr12:12343314-12343528	215	1.9	2.4	1.1	↑	1.3	3.8E-05	7.7E-03
chr12:12357901-12358502	602	5.8	5.5	6.0	↓	-0.5	8.6E-06	2.2E-03
chr12:12670811-12670982	172	2.0	2.4	1.4	↑	1.0	7.1E-04	7.3E-02
chr12:12680187-12680392	206	2.4	2.9	1.7	↑	1.2	4.5E-06	1.4E-03
chr12:1276901-1277750	850	5.6	5.4	5.8	↓	-0.3	1.1E-03	9.8E-02
chr12:12781404-12781734	331	2.6	3.0	2.0	↑	1.0	2.7E-04	3.5E-02
chr12:1290705-1291074	370	2.6	1.8	3.1	↓	-1.3	2.1E-06	7.3E-04
chr12:13139543-13139823	281	3.1	3.4	2.7	↑	0.8	3.1E-04	4.0E-02
chr12:13264590-13264845	256	4.0	4.3	3.6	↑	0.6	5.5E-05	1.0E-02
chr12:13387474-13387929	456	4.4	4.0	4.7	↓	-0.7	4.4E-05	8.6E-03
chr12:13401114-13401258	145	1.5	0.7	2.0	↓	-1.3	5.7E-04	6.3E-02
chr12:13457149-13457356	208	2.1	2.5	1.5	↑	1.1	3.8E-04	4.6E-02
chr12:13578081-13578427	347	3.1	3.5	2.6	↑	0.8	1.0E-04	1.7E-02
chr12:1456218-1457583	1366	6.0	6.2	5.9	↑	0.3	1.1E-03	1.0E-01
chr12:1486054-1489495	3442	7.9	7.8	8.0	↓	-0.2	8.8E-05	1.5E-02
chr12:14903302-14903505	204	2.6	3.0	2.2	↑	0.8	9.5E-04	9.0E-02
chr12:14910436-14910546	111	1.7	2.2	0.9	↑	1.3	1.6E-04	2.3E-02
chr12:14950352-14951923	1572	6.5	6.3	6.7	↓	-0.4	1.0E-03	9.6E-02
chr12:14983011-14983718	708	4.6	4.2	4.9	↓	-0.7	7.5E-06	2.0E-03
chr12:15041820-15042028	209	2.2	2.6	1.6	↑	1.0	4.3E-04	5.1E-02
chr12:15044518-15045400	883	4.3	4.6	3.9	↑	0.7	2.0E-06	7.1E-04
chr12:1506850-1509065	2216	7.3	7.1	7.4	↓	-0.3	2.5E-06	8.7E-04
chr12:1511358-1511824	467	4.3	4.0	4.6	↓	-0.6	1.3E-04	2.0E-02
chr12:1562192-1562782	591	4.6	4.2	4.9	↓	-0.7	1.0E-05	2.5E-03
chr12:15923103-15923982	880	4.9	4.6	5.1	↓	-0.5	7.4E-05	1.3E-02
chr12:15979165-15979348	184	2.7	2.2	3.1	↓	-0.9	3.3E-04	4.1E-02
chr12:16760514-16760652	139	1.8	2.4	1.0	↑	1.4	4.3E-05	8.5E-03
chr12:1694803-1695147	345	3.1	3.6	2.5	↑	1.0	5.9E-06	1.7E-03
chr12:1699644-1703092	3449	8.3	8.4	8.2	↑	0.2	2.5E-07	1.3E-04
chr12:1705682-1706274	593	3.9	3.4	4.4	↓	-1.0	1.1E-06	4.3E-04
chr12:1708359-1710304	1946	7.7	7.8	7.6	↑	0.2	6.0E-04	6.5E-02
chr12:17152414-17152846	433	4.0	3.7	4.3	↓	-0.6	1.1E-04	1.7E-02
chr12:17220697-17221086	390	2.3	1.3	2.8	↓	-1.5	2.2E-07	1.2E-04
chr12:180040-180548	509	3.6	3.9	3.3	↑	0.6	6.8E-04	7.0E-02
chr12:18358605-18359025	421	3.6	3.0	4.0	↓	-1.0	8.4E-06	2.1E-03

chr12:18600032-18600784	753	5.1	5.3	5.0	↑	0.3	9.3E-04	8.8E-02
chr12:18691220-18691550	331	3.4	3.0	3.7	↓	-0.7	7.7E-04	7.6E-02
chr12:18741994-18742401	408	5.7	5.4	6.0	↓	-0.6	5.5E-07	2.7E-04
chr12:18802932-18803107	176	2.1	2.5	1.4	↑	1.1	2.7E-04	3.5E-02
chr12:18954521-18954906	386	3.9	3.5	4.1	↓	-0.6	7.1E-04	7.2E-02
chr12:1895950-1896966	1017	5.2	5.0	5.4	↓	-0.4	1.4E-04	2.1E-02
chr12:19012577-19013176	600	3.9	3.5	4.2	↓	-0.7	3.7E-05	7.6E-03
chr12:19217341-19217545	205	1.9	1.0	2.4	↓	-1.5	1.9E-05	4.4E-03
chr12:19244898-19245350	453	3.7	3.4	4.0	↓	-0.6	7.5E-04	7.5E-02
chr12:19270923-19271167	245	2.7	3.1	2.1	↑	1.0	8.1E-04	7.9E-02
chr12:19271616-19272072	457	4.2	4.5	3.8	↑	0.8	6.1E-06	1.7E-03
chr12:19405275-19405479	205	2.5	2.0	2.9	↓	-0.9	8.0E-04	7.8E-02
chr12:19427976-19428388	413	3.9	3.2	4.3	↓	-1.1	7.7E-12	1.1E-08
chr12:19907170-19907618	449	4.5	4.1	4.7	↓	-0.6	1.3E-04	2.0E-02
chr12:20311469-20311803	335	4.6	4.9	4.2	↑	0.7	1.1E-07	6.8E-05
chr12:20380255-20380724	470	3.7	3.2	4.1	↓	-1.0	1.1E-07	7.0E-05
chr12:20403189-20403391	203	3.0	2.6	3.4	↓	-0.9	2.7E-04	3.5E-02
chr12:20847729-20848004	276	3.3	3.6	3.0	↑	0.7	7.6E-04	7.6E-02
chr12:21025956-21026202	247	3.2	3.6	2.6	↑	1.0	1.1E-06	4.3E-04
chr12:21042349-21042544	196	2.5	2.9	1.8	↑	1.1	5.0E-05	9.4E-03
chr12:21159421-21160152	732	6.0	6.1	5.9	↑	0.2	6.7E-04	7.0E-02
chr12:21431907-21432199	293	3.7	3.4	4.0	↓	-0.6	2.5E-04	3.4E-02
chr12:21729234-21729578	345	4.7	4.5	4.9	↓	-0.5	1.9E-04	2.7E-02
chr12:21737041-21737788	748	5.4	5.6	5.2	↑	0.4	7.0E-04	7.2E-02
chr12:22161953-22162449	497	4.7	4.5	4.9	↓	-0.4	3.2E-04	4.0E-02
chr12:22257127-22257466	340	3.0	2.5	3.4	↓	-0.9	3.3E-05	6.9E-03
chr12:22307585-22307808	224	2.0	2.4	1.4	↑	1.0	1.1E-03	9.9E-02
chr12:2241591-2241934	344	3.7	4.1	3.1	↑	1.0	1.4E-07	8.1E-05
chr12:22478765-22479235	471	3.8	3.4	4.1	↓	-0.7	9.2E-05	1.6E-02
chr12:227058-230433	3376	7.9	8.0	7.8	↑	0.1	1.1E-03	9.7E-02
chr12:23119648-23120050	403	3.2	2.6	3.6	↓	-1.0	1.9E-05	4.3E-03
chr12:23499936-23500246	311	3.8	3.4	4.2	↓	-0.8	7.8E-06	2.0E-03
chr12:23527340-23527523	184	1.7	0.5	2.3	↓	-1.8	1.2E-06	4.8E-04
chr12:23532948-23533559	612	4.7	4.3	5.0	↓	-0.7	9.7E-07	4.2E-04
chr12:23533670-23533881	212	2.7	1.9	3.2	↓	-1.2	3.5E-06	1.2E-03
chr12:23543427-23543637	211	3.0	2.2	3.5	↓	-1.3	1.5E-08	1.1E-05
chr12:23571770-23572024	255	2.2	1.3	2.7	↓	-1.4	3.1E-06	1.0E-03
chr12:23660797-23660952	156	2.5	1.8	3.0	↓	-1.2	4.1E-06	1.3E-03
chr12:24000379-24000574	196	2.1	1.4	2.6	↓	-1.2	1.0E-04	1.6E-02
chr12:24027352-24027652	301	4.1	4.4	3.8	↑	0.5	6.2E-04	6.6E-02
chr12:24036603-24036816	214	2.2	2.6	1.6	↑	1.0	7.7E-04	7.6E-02
chr12:2406256-2408002	1747	7.0	6.9	7.1	↓	-0.2	3.4E-04	4.3E-02
chr12:24110029-24110243	215	3.8	3.5	4.1	↓	-0.6	1.0E-04	1.7E-02
chr12:24182148-24182302	155	2.2	1.5	2.6	↓	-1.1	2.1E-04	2.9E-02
chr12:24231851-24232106	256	2.8	2.3	3.1	↓	-0.9	4.7E-04	5.4E-02
chr12:24366863-24367062	200	3.0	2.5	3.3	↓	-0.8	3.4E-04	4.2E-02
chr12:24368711-24368884	174	1.8	0.9	2.4	↓	-1.5	2.7E-05	5.8E-03

chr12:24370356-24370551	196	3.0	2.4	3.5	↓	-1.1	5.8E-06	1.7E-03
chr12:24470836-24471130	295	2.5	1.9	3.0	↓	-1.0	9.4E-05	1.6E-02
chr12:24493273-24493450	178	2.4	0.5	3.2	↓	-2.7	7.0E-18	2.2E-14
chr12:24607662-24607980	319	2.4	1.6	3.0	↓	-1.3	1.6E-06	6.3E-04
chr12:24635588-24636164	577	5.0	4.6	5.3	↓	-0.7	8.1E-08	5.3E-05
chr12:24637268-24637698	431	3.9	3.0	4.5	↓	-1.5	4.7E-18	1.6E-14
chr12:24695358-24695666	309	2.4	1.5	3.0	↓	-1.5	1.4E-07	8.0E-05
chr12:24724276-24724522	247	2.5	1.5	3.0	↓	-1.6	3.4E-08	2.4E-05
chr12:24734273-24734678	406	3.4	2.8	3.8	↓	-1.0	1.7E-06	6.4E-04
chr12:24742161-24742670	510	4.8	4.5	5.1	↓	-0.6	3.9E-04	4.7E-02
chr12:24743463-24744094	632	5.0	4.5	5.4	↓	-0.9	1.6E-11	2.1E-08
chr12:24744283-24745147	865	5.0	4.4	5.4	↓	-1.0	8.0E-15	1.8E-11
chr12:24745462-24746030	569	4.7	4.3	5.0	↓	-0.8	1.8E-10	1.8E-07
chr12:24768346-24768832	487	3.8	4.2	3.3	↑	0.9	5.5E-07	2.7E-04
chr12:24775294-24776610	1317	6.7	6.0	7.2	↓	-1.2	4.6E-17	1.3E-13
chr12:24776729-24777032	304	2.8	3.3	2.2	↑	1.1	6.7E-06	1.8E-03
chr12:24777047-24777492	446	4.5	4.9	4.0	↑	0.8	8.7E-10	7.9E-07
chr12:24779893-24780524	632	4.6	5.1	3.7	↑	1.4	1.6E-24	6.5E-21
chr12:24782307-24783111	805	5.4	5.7	5.0	↑	0.8	1.7E-06	6.4E-04
chr12:24787325-24787818	494	4.0	4.4	3.4	↑	1.1	2.4E-07	1.3E-04
chr12:24791361-24791535	175	2.1	2.5	1.4	↑	1.0	6.5E-04	6.9E-02
chr12:24823589-24823883	295	2.5	1.6	3.0	↓	-1.4	7.3E-07	3.3E-04
chr12:24827590-24827951	362	3.3	2.2	3.9	↓	-1.7	5.8E-15	1.3E-11
chr12:24831209-24831655	447	3.2	2.2	3.7	↓	-1.5	5.0E-11	5.9E-08
chr12:24834468-24834767	300	2.7	1.7	3.3	↓	-1.7	7.7E-11	8.7E-08
chr12:24835086-24835290	205	2.3	0.8	3.0	↓	-2.2	2.7E-12	4.1E-09
chr12:24835745-24836051	307	2.7	1.6	3.3	↓	-1.7	1.3E-10	1.4E-07
chr12:24838227-24838388	162	2.1	1.5	2.6	↓	-1.1	2.0E-04	2.9E-02
chr12:24838675-24838918	244	2.9	2.2	3.3	↓	-1.1	5.3E-06	1.6E-03
chr12:24840865-24841303	439	3.6	2.4	4.2	↓	-1.8	4.7E-17	1.3E-13
chr12:24841392-24842007	616	5.1	3.8	5.7	↓	-1.9	2.0E-29	1.5E-25
chr12:24842159-24842534	376	2.7	1.6	3.3	↓	-1.7	3.2E-11	4.0E-08
chr12:24846060-24846435	376	3.8	2.1	4.6	↓	-2.5	3.3E-34	4.1E-30
chr12:24847341-24848292	952	4.5	3.0	5.2	↓	-2.1	5.0E-33	4.6E-29
chr12:24848350-24848589	240	2.6	1.3	3.3	↓	-2.1	2.6E-14	5.5E-11
chr12:24848753-24849845	1093	5.6	3.8	6.4	↓	-2.5	3.8E-49	1.4E-44
chr12:24850919-24852307	1389	6.0	4.1	6.7	↓	-2.6	8.6E-45	1.6E-40
chr12:24853395-24853694	300	3.2	1.6	3.9	↓	-2.3	5.9E-21	2.2E-17
chr12:24853803-24854149	347	4.3	2.7	5.1	↓	-2.4	3.5E-28	2.2E-24
chr12:24861067-24861467	401	3.5	2.9	3.9	↓	-1.0	6.0E-07	2.8E-04
chr12:24872400-24872756	357	3.5	2.3	4.1	↓	-1.8	1.6E-13	3.0E-10
chr12:24876927-24877137	211	2.6	3.2	1.8	↑	1.4	8.1E-06	2.1E-03
chr12:24877374-24878051	678	4.0	4.5	3.5	↑	1.0	1.2E-09	1.0E-06
chr12:24878724-24879563	840	5.1	5.8	3.9	↑	1.9	5.3E-28	2.8E-24
chr12:24881806-24882028	223	2.4	2.9	1.6	↑	1.3	7.8E-06	2.0E-03
chr12:24882054-24882306	253	2.9	3.4	2.2	↑	1.1	1.1E-06	4.5E-04
chr12:24882545-24882941	397	4.0	4.7	2.4	↑	2.3	7.0E-28	3.2E-24

chr12:24886562-24886723	162	1.9	2.4	1.0	↑	1.4	4.1E-05	8.2E-03
chr12:24997022-24997295	274	3.4	3.0	3.7	↓	-0.8	1.2E-04	1.9E-02
chr12:25130500-25130744	245	4.0	3.6	4.2	↓	-0.6	3.6E-04	4.4E-02
chr12:25177409-25178111	703	5.1	4.8	5.4	↓	-0.6	2.5E-04	3.3E-02
chr12:25353363-25353637	275	3.6	3.2	4.0	↓	-0.8	3.0E-05	6.4E-03
chr12:25363318-25363664	347	3.7	3.3	4.0	↓	-0.7	6.4E-04	6.8E-02
chr12:25440302-25440546	245	3.1	2.7	3.4	↓	-0.7	1.1E-03	1.0E-01
chr12:25535884-25536422	539	3.8	4.1	3.4	↑	0.8	9.2E-06	2.3E-03
chr12:25689314-25689705	392	2.4	1.9	2.8	↓	-0.9	1.1E-03	9.9E-02
chr12:25701151-25701522	372	4.6	4.3	4.8	↓	-0.5	9.5E-05	1.6E-02
chr12:26022391-26022635	245	3.1	2.6	3.4	↓	-0.8	1.5E-04	2.2E-02
chr12:26165379-26166168	790	5.1	4.9	5.3	↓	-0.4	9.2E-04	8.7E-02
chr12:26418901-26419267	367	3.3	2.8	3.6	↓	-0.8	2.0E-04	2.8E-02
chr12:26490179-26490526	348	3.6	3.2	4.0	↓	-0.7	4.1E-04	4.9E-02
chr12:26492380-26492890	511	4.9	4.6	5.1	↓	-0.5	1.7E-05	4.0E-03
chr12:26503062-26503386	325	4.3	4.6	3.9	↑	0.6	4.8E-05	9.1E-03
chr12:26531230-26531650	421	3.7	4.0	3.3	↑	0.7	2.6E-05	5.7E-03
chr12:26927204-26927371	168	2.6	2.1	3.0	↓	-1.0	1.2E-04	1.9E-02
chr12:26994695-26995001	307	3.3	2.7	3.7	↓	-1.1	1.2E-06	4.8E-04
chr12:26999335-26999563	229	3.1	3.5	2.6	↑	0.8	1.3E-04	2.0E-02
chr12:27281446-27282299	854	6.0	5.6	6.3	↓	-0.6	3.5E-07	1.8E-04
chr12:27430479-27430692	214	1.8	0.8	2.4	↓	-1.6	3.6E-06	1.2E-03
chr12:27599229-27599368	140	2.1	2.6	1.3	↑	1.3	4.2E-05	8.2E-03
chr12:27674543-27674817	275	2.6	3.0	2.2	↑	0.9	4.6E-04	5.3E-02
chr12:27981347-27981565	219	2.6	2.0	3.0	↓	-1.0	6.4E-05	1.2E-02
chr12:27981696-27981835	140	2.0	1.3	2.4	↓	-1.2	3.5E-04	4.4E-02
chr12:2978027-2978741	715	6.5	6.3	6.7	↓	-0.4	1.2E-04	1.9E-02
chr12:3012270-3013896	1627	7.0	6.9	7.1	↓	-0.2	4.7E-05	9.0E-03
chr12:3090219-3090677	459	4.5	4.1	4.7	↓	-0.6	1.2E-04	1.8E-02
chr12:3111315-3112446	1132	6.3	6.1	6.4	↓	-0.3	2.7E-05	5.8E-03
chr12:3142272-3143578	1307	5.9	5.7	6.0	↓	-0.3	2.2E-04	3.0E-02
chr12:31583718-31583939	222	3.1	2.5	3.6	↓	-1.0	9.4E-05	1.6E-02
chr12:32253320-32253472	153	1.9	2.4	1.2	↑	1.1	6.0E-04	6.5E-02
chr12:32263553-32263820	268	2.1	1.3	2.6	↓	-1.3	1.3E-05	3.1E-03
chr12:32343514-32343701	188	2.4	2.8	1.8	↑	1.0	1.8E-04	2.6E-02
chr12:329771-329932	162	1.8	2.3	1.1	↑	1.2	6.0E-04	6.5E-02
chr12:3303826-3304491	666	4.5	4.2	4.8	↓	-0.6	9.0E-05	1.5E-02
chr12:33193618-33193899	282	3.7	2.8	4.2	↓	-1.4	1.8E-13	3.3E-10
chr12:3347815-3348034	220	2.1	2.6	1.4	↑	1.1	1.8E-04	2.6E-02
chr12:33572460-33572712	253	3.4	2.9	3.7	↓	-0.8	1.1E-04	1.7E-02
chr12:34228223-34228343	121	1.3	1.9	0.5	↑	1.3	5.5E-04	6.1E-02
chr12:34256594-34256818	225	2.8	2.3	3.2	↓	-0.9	3.0E-04	3.9E-02
chr12:34523573-34523826	254	2.2	1.6	2.6	↓	-1.0	5.2E-04	5.8E-02
chr12:34584452-34584825	374	4.1	3.5	4.5	↓	-1.0	2.4E-10	2.4E-07
chr12:34777188-34777674	487	3.8	3.2	4.2	↓	-1.0	6.1E-07	2.8E-04
chr12:35029153-35029300	148	2.2	1.7	2.6	↓	-0.9	1.1E-03	1.0E-01
chr12:3540132-3540783	652	5.6	5.2	5.9	↓	-0.6	3.4E-13	5.8E-10

chr12:3575442-3576053	612	4.5	3.9	4.9	↓	-1.0	2.2E-07	1.2E-04
chr12:3595965-3596357	393	5.2	4.9	5.4	↓	-0.5	1.6E-04	2.3E-02
chr12:36721480-36721715	236	3.3	2.8	3.6	↓	-0.8	1.3E-04	2.0E-02
chr12:3677349-3678019	671	4.6	4.8	4.2	↑	0.6	2.6E-05	5.7E-03
chr12:37965685-37965808	124	1.5	0.4	2.1	↓	-1.8	5.8E-06	1.7E-03
chr12:38249716-38249990	275	4.1	3.7	4.4	↓	-0.6	3.8E-05	7.7E-03
chr12:38251650-38252734	1085	5.1	4.7	5.4	↓	-0.6	2.2E-08	1.6E-05
chr12:38259578-38260341	764	5.0	4.5	5.3	↓	-0.8	1.5E-08	1.1E-05
chr12:38332593-38332976	384	3.0	3.5	2.4	↑	1.1	7.5E-06	2.0E-03
chr12:38333280-38333509	230	2.8	3.2	2.2	↑	1.0	1.4E-04	2.1E-02
chr12:3843454-3843763	310	4.3	4.0	4.6	↓	-0.5	3.2E-04	4.1E-02
chr12:38516537-38516867	331	4.4	3.9	4.8	↓	-1.0	4.8E-11	5.7E-08
chr12:3856041-3856881	841	5.6	5.2	5.8	↓	-0.6	8.3E-11	8.8E-08
chr12:3884307-3885269	963	5.4	5.6	5.2	↑	0.4	2.6E-05	5.7E-03
chr12:39161162-39161363	202	2.3	2.7	1.8	↑	1.0	5.3E-04	6.0E-02
chr12:3938364-3940690	2327	7.3	7.4	7.1	↑	0.2	5.2E-06	1.5E-03
chr12:3995395-3996151	757	4.9	4.7	5.1	↓	-0.4	6.7E-04	7.0E-02
chr12:39983675-39983933	259	3.4	2.9	3.7	↓	-0.8	1.7E-05	4.1E-03
chr12:40213391-40213554	164	1.9	2.4	0.9	↑	1.6	8.4E-06	2.1E-03
chr12:4032515-4033047	533	3.4	3.7	3.0	↑	0.7	1.4E-04	2.1E-02
chr12:40629033-40629177	145	2.2	1.4	2.8	↓	-1.3	4.2E-06	1.3E-03
chr12:40796901-40797139	239	2.7	2.1	3.2	↓	-1.1	7.7E-06	2.0E-03
chr12:4087316-4087575	260	3.9	3.5	4.2	↓	-0.7	2.8E-05	5.9E-03
chr12:41278388-41278593	206	1.9	2.4	1.2	↑	1.2	1.6E-04	2.4E-02
chr12:4134737-4135251	515	3.6	3.9	3.2	↑	0.7	7.7E-05	1.3E-02
chr12:41541773-41542639	867	5.4	5.1	5.6	↓	-0.5	6.4E-07	2.9E-04
chr12:41697042-41699144	2103	7.6	7.5	7.7	↓	-0.2	2.5E-05	5.7E-03
chr12:41774112-41774397	286	2.4	1.9	2.8	↓	-0.9	6.5E-04	6.9E-02
chr12:41895699-41896290	592	4.5	4.2	4.7	↓	-0.4	8.7E-04	8.4E-02
chr12:42410206-42410671	466	3.8	3.4	4.0	↓	-0.6	7.7E-04	7.6E-02
chr12:42466689-42467011	323	3.6	3.0	4.0	↓	-1.0	1.3E-07	8.0E-05
chr12:42530489-42530777	289	3.2	2.7	3.6	↓	-1.0	5.9E-06	1.7E-03
chr12:42549939-42550336	398	4.2	4.4	3.8	↑	0.6	5.2E-05	9.6E-03
chr12:42573627-42573900	274	3.9	3.6	4.2	↓	-0.7	7.4E-05	1.3E-02
chr12:42630136-42630462	327	3.8	3.5	4.1	↓	-0.6	4.8E-04	5.5E-02
chr12:42673197-42673537	341	2.9	2.3	3.3	↓	-1.0	7.5E-05	1.3E-02
chr12:4286279-4286499	221	2.5	2.0	3.0	↓	-1.0	3.5E-04	4.3E-02
chr12:42876658-42876905	248	2.5	2.9	1.9	↑	1.0	9.3E-05	1.6E-02
chr12:42990158-42990319	162	1.9	1.2	2.4	↓	-1.2	1.0E-03	9.6E-02
chr12:4301138-4301424	287	3.7	3.3	4.0	↓	-0.7	1.5E-04	2.3E-02
chr12:43187495-43187802	308	5.1	4.8	5.3	↓	-0.5	2.1E-06	7.3E-04
chr12:43284609-43285165	557	4.5	4.2	4.7	↓	-0.6	6.7E-04	7.0E-02
chr12:43348716-43349404	689	4.3	3.9	4.6	↓	-0.7	7.1E-06	1.9E-03
chr12:43722167-43722740	574	5.3	5.1	5.5	↓	-0.4	3.2E-04	4.1E-02
chr12:43806843-43807126	284	3.3	2.6	3.8	↓	-1.2	1.1E-06	4.5E-04
chr12:43807917-43808377	461	4.2	3.7	4.6	↓	-0.9	6.7E-08	4.5E-05
chr12:43809370-43809832	463	3.8	3.4	4.1	↓	-0.6	3.8E-04	4.6E-02

chr12:43810897-43811622	726	5.1	4.6	5.4	↓	-0.9	2.4E-11	3.1E-08
chr12:43839663-43840267	605	4.8	4.3	5.2	↓	-0.9	8.2E-11	8.8E-08
chr12:44534399-44534938	540	4.8	4.3	5.1	↓	-0.8	3.6E-06	1.2E-03
chr12:4474912-4475253	342	3.4	2.8	3.8	↓	-0.9	5.9E-06	1.7E-03
chr12:45211912-45212377	466	3.8	3.5	4.1	↓	-0.6	6.6E-04	6.9E-02
chr12:45282356-45282695	340	3.2	2.8	3.5	↓	-0.7	9.0E-04	8.6E-02
chr12:4529839-4530948	1110	5.6	5.3	5.8	↓	-0.5	1.4E-07	8.0E-05
chr12:45726052-45726204	153	2.3	1.8	2.7	↓	-1.0	1.1E-03	9.9E-02
chr12:45870808-45871063	256	3.7	3.2	4.1	↓	-0.9	1.8E-06	6.4E-04
chr12:45925390-45925916	527	5.1	4.8	5.3	↓	-0.4	1.1E-04	1.7E-02
chr12:45940724-45941239	516	5.3	4.9	5.5	↓	-0.6	7.8E-05	1.4E-02
chr12:46025406-46025613	208	2.4	1.9	2.8	↓	-1.0	3.2E-04	4.1E-02
chr12:46046773-46047130	358	3.4	3.8	2.9	↑	1.0	2.0E-06	7.1E-04
chr12:46211935-46212423	489	3.1	2.7	3.4	↓	-0.8	2.2E-04	3.0E-02
chr12:46401674-46401956	283	2.0	2.5	1.3	↑	1.1	4.7E-04	5.4E-02
chr12:47380334-47380631	298	2.7	3.1	2.2	↑	0.9	3.3E-04	4.1E-02
chr12:4801416-4802525	1110	6.8	6.6	6.9	↓	-0.3	5.9E-06	1.7E-03
chr12:48080394-48080553	160	2.5	2.8	1.9	↑	0.9	1.1E-03	9.7E-02
chr12:48098387-48098585	199	2.8	3.1	2.3	↑	0.8	8.7E-04	8.4E-02
chr12:48137386-48137932	547	4.8	5.0	4.5	↑	0.5	1.3E-04	2.0E-02
chr12:48184024-48184511	488	3.9	4.2	3.6	↑	0.6	9.6E-04	9.0E-02
chr12:4821092-4822134	1043	6.3	6.4	6.1	↑	0.3	3.9E-04	4.6E-02
chr12:4822291-4822796	506	3.1	2.6	3.4	↓	-0.8	1.7E-04	2.5E-02
chr12:48224937-48225114	178	1.4	2.0	0.5	↑	1.5	1.5E-04	2.3E-02
chr12:48852697-48853114	418	3.9	3.4	4.2	↓	-0.8	9.9E-07	4.2E-04
chr12:48939012-48939370	359	5.8	5.6	6.0	↓	-0.5	2.7E-04	3.6E-02
chr12:48958892-48959171	280	2.7	2.2	3.1	↓	-0.8	5.7E-04	6.3E-02
chr12:48966444-48966761	318	3.3	2.8	3.7	↓	-0.9	3.8E-06	1.2E-03
chr12:49058645-49058807	163	2.4	1.7	2.8	↓	-1.2	2.7E-05	5.8E-03
chr12:49200092-49200303	212	2.7	2.2	3.1	↓	-0.9	5.8E-04	6.3E-02
chr12:49329277-49330346	1070	5.7	5.6	5.9	↓	-0.3	1.0E-03	9.5E-02
chr12:49357339-49357686	348	3.2	2.8	3.5	↓	-0.7	3.2E-04	4.1E-02
chr12:49484038-49484762	725	6.6	6.5	6.8	↓	-0.3	2.6E-05	5.7E-03
chr12:49644919-49645157	239	3.6	3.3	3.9	↓	-0.6	1.1E-03	9.8E-02
chr12:4994689-4995481	793	5.0	4.5	5.3	↓	-0.7	3.1E-10	3.0E-07
chr12:49947623-49948021	399	5.8	6.0	5.6	↑	0.4	8.6E-05	1.5E-02
chr12:49972156-49972493	338	3.0	3.4	2.6	↑	0.7	1.1E-03	1.0E-01
chr12:50021755-50024961	3207	8.9	9.1	8.7	↑	0.4	1.1E-04	1.8E-02
chr12:50062376-50062550	175	1.8	1.1	2.3	↓	-1.2	4.6E-04	5.3E-02
chr12:50073144-50073452	309	2.7	3.1	2.0	↑	1.1	1.3E-05	3.1E-03
chr12:5071929-5072056	128	1.5	0.7	1.9	↓	-1.2	9.1E-04	8.7E-02
chr12:51544305-51544707	403	4.6	4.2	4.9	↓	-0.7	2.3E-04	3.1E-02
chr12:51610546-51610872	327	3.1	2.7	3.4	↓	-0.7	4.2E-04	4.9E-02
chr12:52409957-52410153	197	2.2	2.7	1.6	↑	1.1	2.4E-04	3.3E-02
chr12:52460480-52461602	1123	6.5	6.6	6.4	↑	0.2	4.4E-04	5.2E-02
chr12:52822102-52822540	439	4.9	4.7	5.1	↓	-0.5	5.4E-05	9.9E-03
chr12:53017032-53017572	541	3.9	3.6	4.2	↓	-0.6	6.9E-04	7.1E-02

chr12:53532952-53533127	176	2.7	3.1	2.3	↑	0.8	1.1E-03	9.9E-02
chr12:53540385-53540476	92	1.4	1.9	0.5	↑	1.3	7.0E-04	7.1E-02
chr12:5382279-5382864	586	5.8	5.4	6.0	↓	-0.6	2.7E-04	3.5E-02
chr12:53994021-53994398	378	3.2	2.7	3.5	↓	-0.9	2.6E-05	5.7E-03
chr12:5455586-5455764	179	2.0	2.4	1.3	↑	1.2	2.2E-04	3.0E-02
chr12:54654725-54654958	234	3.2	2.6	3.7	↓	-1.1	2.7E-05	5.8E-03
chr12:54655080-54655730	651	4.0	3.4	4.4	↓	-1.0	5.7E-07	2.7E-04
chr12:55457559-55457872	314	3.2	1.9	3.9	↓	-2.0	3.0E-15	7.4E-12
chr12:556656-557164	509	3.6	3.1	4.0	↓	-0.9	9.8E-07	4.2E-04
chr12:5575157-5575344	188	2.5	2.9	2.0	↑	0.9	1.1E-03	9.8E-02
chr12:55999960-56000328	369	2.6	3.0	2.1	↑	0.9	5.8E-04	6.3E-02
chr12:56340375-56340574	200	2.7	2.0	3.1	↓	-1.1	4.2E-05	8.3E-03
chr12:56572541-56572815	275	2.6	2.0	3.0	↓	-1.0	3.8E-05	7.7E-03
chr12:5661515-5662031	517	4.3	3.9	4.6	↓	-0.6	3.9E-06	1.2E-03
chr12:56676030-56676416	387	5.1	4.8	5.3	↓	-0.5	6.9E-04	7.1E-02
chr12:56724453-56724688	236	1.7	1.0	2.2	↓	-1.2	9.0E-04	8.6E-02
chr12:56786916-56787276	361	4.8	4.5	5.0	↓	-0.4	2.6E-04	3.4E-02
chr12:56824057-56824544	488	3.2	3.7	2.4	↑	1.3	2.8E-09	2.3E-06
chr12:56824591-56824775	185	1.9	2.4	1.1	↑	1.3	5.7E-05	1.0E-02
chr12:56824813-56825231	419	4.6	5.0	4.1	↑	0.9	2.2E-06	7.7E-04
chr12:56866016-56866506	491	3.8	3.4	4.1	↓	-0.8	6.4E-05	1.2E-02
chr12:56869804-56870138	335	3.9	3.5	4.3	↓	-0.8	4.9E-06	1.5E-03
chr12:56978371-56978766	396	3.1	2.7	3.5	↓	-0.8	2.3E-04	3.1E-02
chr12:56983554-56983925	372	3.5	3.8	3.1	↑	0.7	4.2E-04	5.0E-02
chr12:56983964-56984929	966	5.0	5.2	4.7	↑	0.5	4.4E-04	5.2E-02
chr12:56994208-56994594	387	3.6	3.9	3.2	↑	0.7	8.3E-05	1.4E-02
chr12:57019481-57019925	445	4.4	4.0	4.7	↓	-0.7	3.8E-07	1.9E-04
chr12:57127746-57128436	691	6.1	6.0	6.3	↓	-0.2	5.1E-04	5.8E-02
chr12:57408164-57408778	615	3.7	4.0	3.4	↑	0.6	9.1E-04	8.7E-02
chr12:57473826-57475461	1636	6.7	6.4	6.9	↓	-0.4	1.7E-08	1.2E-05
chr12:57478119-57478348	230	2.6	2.0	3.0	↓	-1.0	5.0E-05	9.4E-03
chr12:57492531-57494716	2186	6.3	6.2	6.4	↓	-0.2	4.4E-04	5.2E-02
chr12:57508949-57510093	1145	5.9	5.7	6.1	↓	-0.3	5.1E-04	5.8E-02
chr12:57651481-57651756	276	3.8	4.1	3.4	↑	0.7	4.1E-05	8.1E-03
chr12:57676742-57677390	649	5.7	6.0	5.4	↑	0.6	7.2E-05	1.3E-02
chr12:57818208-57818753	546	5.7	5.4	5.9	↓	-0.4	3.6E-05	7.5E-03
chr12:57986797-57987011	215	2.1	1.5	2.5	↓	-1.0	8.4E-04	8.2E-02
chr12:58101772-58102028	257	3.6	3.2	4.0	↓	-0.8	2.5E-05	5.6E-03
chr12:58112847-58113687	841	5.7	5.5	5.9	↓	-0.4	3.6E-06	1.2E-03
chr12:58172734-58172986	253	3.8	3.2	4.3	↓	-1.1	1.0E-09	9.1E-07
chr12:58812810-58813176	367	3.7	3.2	4.1	↓	-0.9	6.2E-06	1.7E-03
chr12:59035246-59035552	307	3.1	2.6	3.5	↓	-1.0	1.1E-05	2.6E-03
chr12:59159527-59159762	236	3.3	2.9	3.7	↓	-0.7	4.5E-04	5.2E-02
chr12:59187160-59187661	502	4.3	4.0	4.5	↓	-0.5	2.9E-04	3.8E-02
chr12:59347087-59348383	1297	5.8	6.0	5.7	↑	0.3	1.0E-03	9.6E-02
chr12:59440563-59442093	1531	7.1	6.9	7.2	↓	-0.3	5.7E-04	6.3E-02
chr12:59560489-59560973	485	5.6	5.5	5.8	↓	-0.3	7.4E-04	7.5E-02

chr12:59571840-59572078	239	2.5	2.0	2.9	↓	-0.9	4.7E-04	5.4E-02
chr12:59580262-59581121	860	4.9	4.6	5.1	↓	-0.6	1.9E-06	6.9E-04
chr12:59585995-59586131	137	1.6	0.8	2.1	↓	-1.2	8.0E-04	7.8E-02
chr12:59590200-59591858	1659	6.9	6.6	7.0	↓	-0.4	7.1E-06	1.9E-03
chr12:59626408-59626643	236	2.4	1.8	2.9	↓	-1.1	5.7E-05	1.0E-02
chr12:59637561-59637872	312	2.6	1.9	3.0	↓	-1.1	3.4E-05	7.1E-03
chr12:59655596-59656000	405	4.7	4.4	4.9	↓	-0.5	2.4E-04	3.2E-02
chr12:59660557-59661045	489	3.4	2.9	3.8	↓	-1.0	7.9E-07	3.5E-04
chr12:59697640-59698401	762	5.5	5.3	5.7	↓	-0.4	9.0E-04	8.6E-02
chr12:59708610-59708959	350	4.6	4.2	4.8	↓	-0.6	1.8E-05	4.2E-03
chr12:59753951-59754517	567	4.0	3.6	4.2	↓	-0.6	1.1E-03	9.7E-02
chr12:59760435-59761092	658	5.0	4.6	5.3	↓	-0.6	1.4E-07	8.0E-05
chr12:59879557-59879852	296	3.7	3.2	4.0	↓	-0.8	2.2E-04	3.0E-02
chr12:59884772-59885397	626	4.7	4.4	5.0	↓	-0.6	2.0E-05	4.5E-03
chr12:59885437-59886019	583	4.1	3.7	4.5	↓	-0.8	8.1E-08	5.3E-05
chr12:59913447-59913853	407	3.3	2.6	3.8	↓	-1.2	9.9E-08	6.4E-05
chr12:60019322-60019960	639	4.1	3.8	4.4	↓	-0.6	5.8E-04	6.3E-02
chr12:60047102-60048536	1435	6.3	6.0	6.6	↓	-0.6	4.5E-10	4.2E-07
chr12:60049492-60050063	572	5.2	4.8	5.4	↓	-0.6	2.0E-07	1.1E-04
chr12:60226972-60227213	242	3.4	2.9	3.8	↓	-0.9	2.7E-04	3.5E-02
chr12:60310701-60311064	364	2.8	3.3	2.1	↑	1.2	1.7E-06	6.3E-04
chr12:60324468-60324819	352	4.4	4.2	4.6	↓	-0.5	3.7E-04	4.5E-02
chr12:60539527-60540189	663	5.5	5.3	5.7	↓	-0.4	5.4E-04	6.0E-02
chr12:60542061-60542314	254	2.8	3.1	2.3	↑	0.8	8.6E-04	8.4E-02
chr12:60598295-60599035	741	4.9	4.5	5.2	↓	-0.7	1.1E-08	8.7E-06
chr12:60609413-60609957	545	4.3	3.6	4.7	↓	-1.1	1.1E-11	1.6E-08
chr12:60625567-60626534	968	5.9	5.8	6.1	↓	-0.3	7.2E-04	7.3E-02
chr12:60929343-60929748	406	3.0	2.5	3.4	↓	-0.9	8.1E-05	1.4E-02
chr12:60954263-60954441	179	2.8	2.3	3.2	↓	-0.9	2.1E-04	2.9E-02
chr12:61316506-61316885	380	5.6	5.4	5.8	↓	-0.4	4.2E-06	1.3E-03
chr12:61316968-61317499	532	4.2	3.4	4.6	↓	-1.2	1.1E-13	2.1E-10
chr12:61317505-61317705	201	3.2	2.3	3.8	↓	-1.5	1.6E-08	1.2E-05
chr12:61424105-61424683	579	5.2	4.8	5.5	↓	-0.7	2.4E-07	1.3E-04
chr12:61585515-61586033	519	3.6	3.1	4.0	↓	-0.9	2.2E-06	7.7E-04
chr12:61637469-61637918	450	4.0	3.6	4.3	↓	-0.7	9.4E-06	2.3E-03
chr12:61649477-61650112	636	4.4	4.0	4.7	↓	-0.7	5.9E-05	1.1E-02
chr12:61758255-61758460	206	2.5	1.7	3.0	↓	-1.2	2.2E-05	5.1E-03
chr12:61763957-61764154	198	2.7	3.1	2.1	↑	1.0	4.1E-05	8.2E-03
chr12:61765771-61766236	466	4.0	3.6	4.3	↓	-0.7	4.4E-06	1.3E-03
chr12:61794974-61795508	535	3.5	2.9	3.9	↓	-1.0	3.0E-06	1.0E-03
chr12:61921689-61922074	386	2.5	1.5	3.1	↓	-1.5	2.5E-08	1.8E-05
chr12:61950065-61952628	2564	7.1	7.0	7.2	↓	-0.2	1.1E-03	9.8E-02
chr12:61973734-61974960	1227	5.2	4.9	5.4	↓	-0.5	4.9E-05	9.3E-03
chr12:61980769-61981156	388	4.6	4.3	4.8	↓	-0.5	9.9E-04	9.3E-02
chr12:62255788-62256579	792	4.9	5.1	4.7	↑	0.4	8.8E-04	8.5E-02
chr12:62264035-62264555	521	5.2	5.0	5.3	↓	-0.4	5.5E-04	6.1E-02
chr12:62667034-62667610	577	5.2	5.3	5.0	↑	0.3	7.6E-04	7.6E-02

chr12:62818005-62818149	145	1.8	2.3	1.1	↑	1.2	1.9E-04	2.7E-02
chr12:62818679-62819715	1037	5.3	5.4	5.1	↑	0.4	1.2E-04	1.9E-02
chr12:63095727-63097136	1410	6.4	6.1	6.6	↓	-0.5	3.8E-05	7.7E-03
chr12:63100075-63100334	260	2.9	2.4	3.2	↓	-0.8	9.6E-04	9.0E-02
chr12:63186301-63186783	483	3.6	3.0	4.0	↓	-1.1	1.5E-06	5.9E-04
chr12:63238703-63238989	287	3.1	2.5	3.6	↓	-1.0	1.1E-06	4.3E-04
chr12:63315328-63316348	1021	5.6	5.4	5.8	↓	-0.4	2.3E-04	3.1E-02
chr12:6348555-6348890	336	3.5	3.2	3.8	↓	-0.7	8.4E-04	8.2E-02
chr12:6459481-6459829	349	2.8	2.3	3.1	↓	-0.8	5.7E-04	6.3E-02
chr12:656188-657921	1734	6.4	6.5	6.2	↑	0.3	5.3E-04	6.0E-02
chr12:6782458-6782827	370	4.1	3.6	4.5	↓	-0.9	3.5E-06	1.2E-03
chr12:7016926-7017593	668	4.2	3.8	4.5	↓	-0.7	2.2E-05	5.1E-03
chr12:7729339-7729742	404	5.1	4.5	5.6	↓	-1.1	6.2E-13	1.0E-09
chr12:7798128-7799143	1016	6.7	6.6	6.8	↓	-0.3	7.8E-05	1.4E-02
chr12:7812175-7812918	744	4.7	4.5	4.9	↓	-0.5	2.7E-04	3.5E-02
chr12:7921599-7921787	189	2.1	1.4	2.5	↓	-1.1	3.7E-04	4.5E-02
chr12:8060331-8060505	175	2.1	2.6	1.4	↑	1.2	1.0E-04	1.7E-02
chr12:8115785-8116187	403	3.9	3.6	4.2	↓	-0.6	7.1E-05	1.3E-02
chr12:8264334-8264990	657	5.8	5.6	6.0	↓	-0.4	5.3E-06	1.5E-03
chr12:8556730-8556867	138	1.4	0.6	1.9	↓	-1.3	5.1E-04	5.8E-02
chr12:8655137-8655995	859	5.2	4.9	5.4	↓	-0.4	3.8E-04	4.6E-02
chr12:8665415-8665588	174	1.8	1.1	2.2	↓	-1.1	1.0E-03	9.6E-02
chr12:8677461-8677770	310	3.0	2.3	3.4	↓	-1.2	1.8E-07	1.0E-04
chr12:8677831-8678107	277	2.8	2.4	3.2	↓	-0.8	7.4E-04	7.4E-02
chr12:8787849-8788046	198	2.4	1.6	2.9	↓	-1.2	1.8E-05	4.2E-03
chr12:8790417-8790824	408	4.1	3.7	4.5	↓	-0.8	3.4E-05	7.1E-03
chr12:8801900-8802443	544	3.6	3.1	4.0	↓	-0.9	9.0E-07	4.0E-04
chr12:8879384-8880336	953	5.3	4.9	5.6	↓	-0.7	7.7E-07	3.5E-04
chr12:8914073-8915658	1586	6.1	5.9	6.2	↓	-0.3	1.1E-03	9.8E-02
chr12:8928890-8929220	331	2.8	3.2	2.3	↑	1.0	1.0E-04	1.7E-02
chr12:9001678-9002631	954	4.6	4.2	4.9	↓	-0.8	2.7E-09	2.3E-06
chr12:9028491-9028806	316	3.0	3.4	2.4	↑	1.0	1.8E-04	2.5E-02
chr12:9029392-9030909	1518	6.4	6.7	6.1	↑	0.6	2.8E-04	3.6E-02
chr12:9036007-9036495	489	4.0	4.2	3.6	↑	0.6	2.0E-04	2.8E-02
chr12:9268042-9268443	402	3.8	3.5	4.1	↓	-0.7	6.1E-04	6.6E-02
chr12:9403423-9403790	368	3.5	2.9	3.9	↓	-1.0	4.2E-07	2.1E-04
chr12:9457060-9457581	522	4.2	3.5	4.6	↓	-1.1	7.0E-13	1.1E-09
chr12:9469057-9469225	169	2.4	1.8	2.8	↓	-1.0	3.6E-04	4.4E-02
chr12:958455-958928	474	3.8	4.1	3.5	↑	0.6	3.5E-04	4.4E-02
chr12:9599640-9599833	194	2.2	1.6	2.6	↓	-1.0	7.9E-04	7.8E-02
chr12:9631788-9632272	485	4.1	3.7	4.5	↓	-0.7	1.8E-04	2.6E-02
chr13:100920909-100921345	437	2.6	2.0	3.0	↓	-1.0	2.8E-04	6.2E-02
chr13:100923515-100924022	508	2.9	2.0	3.4	↓	-1.4	3.8E-08	6.9E-05
chr13:101333208-101333566	359	2.2	2.7	1.5	↑	1.1	8.9E-05	3.0E-02
chr13:101651405-101651772	368	2.1	2.6	1.4	↑	1.2	1.6E-04	4.3E-02
chr13:102205859-102206986	1128	5.3	5.7	4.6	↑	1.1	3.5E-14	2.7E-10
chr13:102308453-102308823	371	2.5	2.9	1.9	↑	1.0	1.3E-04	3.7E-02

chr13:105381184-105381641	458	3.3	3.7	2.9	↑	0.8	3.1E-04	6.7E-02
chr13:108226363-108226734	372	2.9	2.4	3.2	↓	-0.8	5.7E-04	9.6E-02
chr13:108237970-108238262	293	1.7	0.8	2.2	↓	-1.5	4.0E-05	1.7E-02
chr13:109008866-109009245	380	2.6	1.9	3.0	↓	-1.1	2.1E-04	5.1E-02
chr13:11162249-11163790	1542	4.8	4.6	5.0	↓	-0.4	5.6E-04	9.4E-02
chr13:113432302-113432657	356	2.2	2.6	1.6	↑	1.0	4.5E-04	8.6E-02
chr13:113445952-113446543	592	3.2	3.5	2.7	↑	0.9	2.8E-04	6.2E-02
chr13:11369876-11370159	284	2.1	1.3	2.6	↓	-1.3	2.9E-05	1.4E-02
chr13:116927105-116927532	428	3.6	3.0	3.9	↓	-0.9	1.0E-06	9.0E-04
chr13:117042028-117042441	414	4.4	4.2	4.7	↓	-0.5	5.3E-04	9.2E-02
chr13:117142471-117142836	366	2.1	1.4	2.6	↓	-1.2	6.3E-05	2.3E-02
chr13:118846536-118847437	902	3.9	4.2	3.5	↑	0.6	1.2E-04	3.7E-02
chr13:118850303-118851904	1602	4.2	4.6	3.7	↑	0.9	2.2E-07	2.6E-04
chr13:119394519-119395142	624	3.3	2.9	3.6	↓	-0.7	5.6E-04	9.4E-02
chr13:119520977-119521689	713	3.4	2.9	3.8	↓	-0.9	2.1E-04	5.1E-02
chr13:12058359-12058972	614	3.3	2.9	3.6	↓	-0.7	5.4E-04	9.3E-02
chr13:120623196-120623726	531	2.3	2.8	1.6	↑	1.2	3.2E-05	1.5E-02
chr13:120647750-120648494	745	2.9	3.3	2.4	↑	0.9	4.9E-04	9.0E-02
chr13:121652332-121652873	542	2.3	1.6	2.8	↓	-1.3	1.5E-05	7.9E-03
chr13:122164240-122165514	1275	4.6	4.2	4.9	↓	-0.6	4.0E-05	1.7E-02
chr13:122226272-122226592	321	2.5	1.5	3.0	↓	-1.5	5.2E-07	5.4E-04
chr13:122229063-122229614	552	2.9	2.1	3.5	↓	-1.4	7.7E-09	1.9E-05
chr13:122229807-122230224	418	2.6	1.7	3.1	↓	-1.5	3.0E-07	3.3E-04
chr13:122230893-122231297	405	2.7	1.5	3.4	↓	-1.9	7.8E-12	3.5E-08
chr13:122422432-122423287	856	4.0	3.7	4.3	↓	-0.6	3.7E-04	7.5E-02
chr13:122741824-122742578	755	4.6	4.4	4.9	↓	-0.5	1.3E-04	3.8E-02
chr13:123000506-123001024	519	2.7	3.1	2.2	↑	0.9	4.5E-04	8.6E-02
chr13:123957458-123958271	814	4.0	3.7	4.3	↓	-0.7	1.7E-05	8.8E-03
chr13:124053865-124054583	719	3.1	2.5	3.5	↓	-1.0	2.0E-05	1.0E-02
chr13:124715775-124717001	1227	4.5	4.1	4.8	↓	-0.7	1.2E-06	1.1E-03
chr13:124753759-124755276	1518	4.2	3.8	4.4	↓	-0.6	9.6E-05	3.0E-02
chr13:124890504-124891566	1063	3.3	2.9	3.7	↓	-0.8	2.2E-04	5.3E-02
chr13:12519742-12520219	478	2.6	2.0	3.0	↓	-0.9	3.6E-04	7.3E-02
chr13:125231847-125232615	769	3.0	2.3	3.5	↓	-1.2	7.4E-07	7.0E-04
chr13:125232652-125232976	325	2.9	2.2	3.4	↓	-1.1	7.0E-07	6.8E-04
chr13:125234080-125236117	2038	5.7	5.4	5.9	↓	-0.5	3.3E-06	2.4E-03
chr13:125675885-125676230	346	2.0	1.3	2.5	↓	-1.3	8.7E-05	2.9E-02
chr13:125677417-125678637	1221	4.8	4.4	5.1	↓	-0.6	1.5E-05	7.9E-03
chr13:125850397-125850865	469	4.9	4.5	5.1	↓	-0.6	1.7E-05	8.8E-03
chr13:126095094-126095500	407	2.5	1.9	2.9	↓	-1.0	2.9E-04	6.5E-02
chr13:126096801-126097384	584	2.9	2.3	3.4	↓	-1.2	8.3E-06	5.2E-03
chr13:129382494-129382900	407	4.1	3.6	4.4	↓	-0.7	4.5E-06	3.2E-03
chr13:129499847-129500128	282	1.8	1.1	2.3	↓	-1.2	5.2E-04	9.2E-02
chr13:129586215-129586821	607	3.1	2.2	3.7	↓	-1.5	4.1E-11	1.7E-07
chr13:129587170-129587559	390	1.9	1.0	2.4	↓	-1.4	3.2E-05	1.5E-02
chr13:129590203-129590875	673	4.0	3.4	4.4	↓	-1.0	8.1E-08	1.2E-04
chr13:130794706-130795293	588	4.2	3.8	4.4	↓	-0.6	7.7E-05	2.7E-02

chr13:131575714-131576530	817	4.9	4.6	5.1	↓	-0.5	8.9E-06	5.4E-03
chr13:131690032-131690935	904	4.0	3.7	4.3	↓	-0.6	4.3E-04	8.4E-02
chr13:131942513-131943287	775	3.0	3.4	2.5	↑	0.8	1.2E-04	3.6E-02
chr13:132055722-132056400	679	3.2	3.5	2.8	↑	0.8	2.5E-04	5.7E-02
chr13:132177530-132177959	430	3.1	2.6	3.4	↓	-0.9	1.4E-04	4.0E-02
chr13:133156834-133157804	971	3.2	3.6	2.7	↑	0.8	8.0E-05	2.7E-02
chr13:134323664-134324214	551	3.0	2.5	3.3	↓	-0.8	3.9E-04	7.7E-02
chr13:134324218-134324917	700	2.9	2.2	3.3	↓	-1.1	2.2E-05	1.1E-02
chr13:135620323-135620647	325	3.2	2.7	3.6	↓	-0.9	6.6E-05	2.4E-02
chr13:135718019-135719353	1335	5.6	5.4	5.8	↓	-0.4	2.0E-04	4.9E-02
chr13:135867389-135867793	405	1.9	0.5	2.6	↓	-2.1	3.9E-09	1.1E-05
chr13:135869535-135870099	565	3.6	2.4	4.2	↓	-1.8	3.6E-16	3.3E-12
chr13:13725693-13726797	1105	4.6	4.0	5.0	↓	-1.0	2.5E-10	8.8E-07
chr13:13726877-13727743	867	3.1	2.6	3.5	↓	-1.0	1.3E-04	3.8E-02
chr13:13797634-13798026	393	2.3	2.8	1.7	↑	1.1	1.3E-04	3.8E-02
chr13:139418046-139418722	677	3.8	3.4	4.1	↓	-0.7	9.4E-05	3.0E-02
chr13:139520547-139521203	657	2.5	1.9	3.0	↓	-1.1	4.4E-05	1.8E-02
chr13:139859323-139860087	765	5.5	5.0	5.9	↓	-0.9	2.5E-12	1.3E-08
chr13:140881190-140882619	1430	4.6	4.9	4.2	↑	0.7	1.2E-07	1.6E-04
chr13:141109420-141110781	1362	4.5	4.8	4.3	↑	0.5	5.0E-05	2.0E-02
chr13:141112711-141113952	1242	4.6	4.9	4.0	↑	0.9	1.8E-10	6.9E-07
chr13:141158774-141159677	904	3.8	4.1	3.5	↑	0.6	4.8E-04	8.8E-02
chr13:141167437-141169203	1767	5.2	5.5	4.9	↑	0.6	4.4E-06	3.2E-03
chr13:141241671-141244033	2363	5.5	5.7	5.4	↑	0.3	2.0E-04	4.9E-02
chr13:141737998-141741046	3049	7.0	7.2	6.8	↑	0.3	1.3E-04	3.8E-02
chr13:14210513-14211550	1038	5.0	5.2	4.8	↑	0.4	1.6E-04	4.3E-02
chr13:143326138-143326664	527	2.4	2.8	1.9	↑	1.0	4.0E-04	7.8E-02
chr13:1452574-1452963	390	2.4	2.8	1.9	↑	0.9	5.9E-04	9.9E-02
chr13:145949967-145950976	1010	3.9	3.5	4.2	↓	-0.6	3.6E-04	7.3E-02
chr13:146493533-146495330	1798	4.4	4.7	4.0	↑	0.7	9.5E-06	5.5E-03
chr13:146913155-146914081	927	3.8	3.2	4.2	↓	-0.9	1.3E-04	3.7E-02
chr13:146914419-146919175	4757	7.7	7.4	7.9	↓	-0.5	2.7E-07	3.0E-04
chr13:146919527-146922052	2526	6.8	6.6	6.9	↓	-0.4	7.7E-05	2.7E-02
chr13:147307508-147308321	814	3.5	3.8	3.0	↑	0.8	3.5E-05	1.6E-02
chr13:147422453-147423075	623	3.1	3.4	2.6	↑	0.8	5.0E-04	9.0E-02
chr13:150065453-150065803	351	2.7	3.1	2.2	↑	0.9	2.8E-04	6.1E-02
chr13:150144869-150145291	423	2.7	2.1	3.1	↓	-1.0	5.2E-05	2.0E-02
chr13:155162842-155163357	516	2.1	1.4	2.5	↓	-1.1	2.4E-04	5.7E-02
chr13:155228153-155228591	439	2.3	1.8	2.7	↓	-1.0	4.7E-04	8.6E-02
chr13:155918542-155919087	546	3.1	2.4	3.6	↓	-1.2	7.8E-08	1.2E-04
chr13:155981255-155982673	1419	5.1	5.3	4.9	↑	0.5	7.1E-05	2.5E-02
chr13:156338152-156339042	891	4.7	4.3	4.9	↓	-0.7	5.9E-08	9.5E-05
chr13:156835983-156836259	277	2.1	1.4	2.6	↓	-1.1	3.6E-04	7.3E-02
chr13:157030771-157031140	370	2.5	1.5	3.1	↓	-1.6	5.1E-08	8.6E-05
chr13:157693362-157693810	449	3.9	3.4	4.3	↓	-0.9	4.7E-06	3.4E-03
chr13:160563922-160564648	727	3.9	3.5	4.2	↓	-0.7	5.4E-06	3.6E-03
chr13:161282457-161282893	437	3.8	3.3	4.2	↓	-0.9	2.6E-06	2.0E-03

chr13:163023166-163024013	848	3.5	2.9	4.0	↓	-1.0	1.6E-07	2.0E-04
chr13:163025494-163025860	367	2.6	2.0	3.0	↓	-1.0	3.8E-04	7.6E-02
chr13:163026245-163028881	2637	5.9	5.2	6.4	↓	-1.2	9.1E-32	4.1E-27
chr13:163031642-163031920	279	2.1	1.4	2.6	↓	-1.2	2.6E-04	5.8E-02
chr13:163113568-163114313	746	3.7	3.2	4.1	↓	-0.9	6.4E-07	6.4E-04
chr13:163417267-163417632	366	2.2	1.6	2.7	↓	-1.1	1.0E-04	3.2E-02
chr13:1644446-1645726	1281	4.2	3.7	4.5	↓	-0.9	3.1E-06	2.4E-03
chr13:166325806-166326556	751	4.3	3.9	4.6	↓	-0.6	5.4E-05	2.0E-02
chr13:168517303-168517677	375	2.0	2.5	1.4	↑	1.1	4.3E-04	8.4E-02
chr13:169271250-169271774	525	2.6	3.0	2.1	↑	0.9	5.7E-04	9.6E-02
chr13:169381816-169384970	3155	7.0	7.2	6.8	↑	0.3	4.5E-04	8.6E-02
chr13:17772923-17773358	436	2.6	1.9	3.0	↓	-1.2	1.4E-05	7.7E-03
chr13:178525437-178527054	1618	5.0	4.3	5.5	↓	-1.3	6.6E-14	4.3E-10
chr13:185719915-185720210	296	1.6	2.1	0.9	↑	1.3	4.7E-04	8.6E-02
chr13:18605380-18605791	412	3.3	2.8	3.6	↓	-0.8	5.0E-04	9.0E-02
chr13:186418410-186418757	348	1.9	1.2	2.4	↓	-1.2	1.8E-04	4.6E-02
chr13:190516323-190516708	386	3.6	3.2	3.9	↓	-0.7	2.3E-04	5.5E-02
chr13:191092883-191093441	559	2.6	1.8	3.1	↓	-1.3	5.9E-05	2.2E-02
chr13:191181993-191182684	692	3.5	3.1	3.8	↓	-0.7	2.2E-04	5.4E-02
chr13:19308727-19309577	851	4.6	4.1	4.9	↓	-0.8	7.9E-09	1.9E-05
chr13:19315050-19315650	601	2.6	2.1	3.0	↓	-1.0	2.4E-04	5.7E-02
chr13:19392455-19393069	615	3.4	3.7	3.0	↑	0.7	4.6E-04	8.6E-02
chr13:199337554-199338164	611	2.9	3.3	2.3	↑	1.0	4.7E-05	1.9E-02
chr13:199338189-199339452	1264	4.4	4.6	4.1	↑	0.6	5.3E-05	2.0E-02
chr13:19970997-19971830	834	3.0	2.6	3.4	↓	-0.8	2.5E-04	5.7E-02
chr13:201049506-201049824	319	3.0	2.5	3.4	↓	-0.9	1.8E-04	4.6E-02
chr13:201888024-201888358	335	2.5	1.8	3.0	↓	-1.2	5.1E-06	3.5E-03
chr13:202362769-202363186	418	3.2	3.6	2.6	↑	1.0	5.6E-06	3.7E-03
chr13:203108939-203109660	722	4.0	3.6	4.3	↓	-0.7	3.0E-04	6.5E-02
chr13:204621609-204622008	400	3.8	3.4	4.1	↓	-0.6	2.3E-04	5.4E-02
chr13:204863801-204864389	589	4.0	3.7	4.3	↓	-0.6	5.2E-04	9.2E-02
chr13:20539660-20540475	816	4.4	4.0	4.7	↓	-0.7	2.3E-04	5.5E-02
chr13:206172429-206173409	981	4.3	3.7	4.7	↓	-0.9	3.7E-07	4.0E-04
chr13:206531294-206531597	304	1.9	1.2	2.4	↓	-1.2	4.7E-04	8.6E-02
chr13:208168932-208170395	1464	5.4	5.2	5.6	↓	-0.5	7.8E-06	4.9E-03
chr13:208266134-208267302	1169	4.4	4.1	4.6	↓	-0.5	1.6E-04	4.4E-02
chr13:208272140-208273656	1517	5.8	5.6	5.9	↓	-0.4	5.4E-04	9.3E-02
chr13:208285308-208285926	619	3.6	3.1	4.0	↓	-0.9	1.5E-06	1.2E-03
chr13:208299003-208299849	847	3.7	3.1	4.1	↓	-1.0	4.1E-08	7.2E-05
chr13:208302585-208303066	482	2.6	2.0	3.0	↓	-1.0	3.0E-04	6.6E-02
chr13:208348150-208348559	410	2.8	2.2	3.2	↓	-1.0	3.2E-05	1.5E-02
chr13:208376337-208377366	1030	4.2	3.8	4.6	↓	-0.7	6.7E-05	2.4E-02
chr13:208477578-208478812	1235	5.2	4.9	5.4	↓	-0.4	9.5E-05	3.0E-02
chr13:208591413-208592083	671	3.3	2.9	3.7	↓	-0.8	6.5E-05	2.3E-02
chr13:208650748-208651587	840	4.5	4.1	4.8	↓	-0.7	9.9E-06	5.6E-03
chr13:208722894-208723506	613	2.9	2.3	3.3	↓	-1.0	1.2E-05	6.4E-03
chr13:208910100-208910825	726	4.1	3.1	4.7	↓	-1.6	2.0E-16	2.3E-12

chr13:209054732-209055260	529	2.9	2.4	3.2	↓	-0.8	5.1E-04	9.1E-02
chr13:209067884-209068317	434	2.0	1.2	2.5	↓	-1.3	8.9E-05	3.0E-02
chr13:209104024-209104501	478	3.1	2.1	3.7	↓	-1.6	2.3E-09	6.6E-06
chr13:209106073-209107968	1896	5.6	5.1	6.0	↓	-0.9	2.4E-22	3.6E-18
chr13:209111318-209112005	688	3.3	2.4	3.9	↓	-1.4	1.8E-09	5.4E-06
chr13:209114093-209115609	1517	5.2	4.4	5.7	↓	-1.2	5.0E-28	1.1E-23
chr13:209115679-209116243	565	2.7	1.8	3.3	↓	-1.5	1.9E-08	3.7E-05
chr13:209130940-209131801	862	4.0	3.4	4.4	↓	-0.9	8.9E-07	8.1E-04
chr13:209136127-209137211	1085	3.8	3.3	4.2	↓	-0.9	3.4E-07	3.7E-04
chr13:209137262-209137843	582	4.3	3.8	4.7	↓	-0.9	1.8E-07	2.1E-04
chr13:209149240-209149851	612	2.9	2.4	3.3	↓	-0.9	1.7E-04	4.5E-02
chr13:209175763-209176228	466	3.0	2.4	3.4	↓	-1.0	9.0E-06	5.5E-03
chr13:209195086-209195493	408	2.5	1.9	3.0	↓	-1.1	1.8E-04	4.6E-02
chr13:209197557-209198236	680	2.8	2.0	3.3	↓	-1.3	9.8E-06	5.6E-03
chr13:209221749-209222363	615	4.5	4.2	4.7	↓	-0.5	4.1E-04	8.1E-02
chr13:209234870-209235821	952	4.2	3.5	4.6	↓	-1.2	1.8E-08	3.6E-05
chr13:209249694-209250347	654	2.9	2.3	3.4	↓	-1.1	6.7E-06	4.4E-03
chr13:209250375-209251040	666	3.7	3.1	4.1	↓	-1.1	7.7E-08	1.2E-04
chr13:210559949-210560642	694	3.4	2.8	3.8	↓	-0.9	1.7E-04	4.5E-02
chr13:211292488-211293538	1051	3.8	3.5	4.1	↓	-0.7	7.9E-05	2.7E-02
chr13:212439227-212439643	417	2.8	2.0	3.3	↓	-1.3	1.3E-06	1.1E-03
chr13:212442703-212444212	1510	5.1	4.8	5.3	↓	-0.5	9.2E-06	5.5E-03
chr13:213033381-213033850	470	3.0	3.3	2.5	↑	0.9	9.3E-05	3.0E-02
chr13:213091098-213091488	391	2.8	2.3	3.2	↓	-0.9	2.0E-04	5.0E-02
chr13:213440073-213441724	1652	5.4	5.6	5.2	↑	0.5	7.7E-07	7.2E-04
chr13:213646437-213647277	841	3.8	3.4	4.2	↓	-0.8	1.3E-04	3.8E-02
chr13:214067477-214068026	550	4.5	4.2	4.8	↓	-0.5	2.3E-04	5.5E-02
chr13:214734044-214734656	613	2.4	1.6	2.9	↓	-1.3	5.2E-05	2.0E-02
chr13:215321413-215324772	3360	6.9	6.8	7.0	↓	-0.2	5.8E-04	9.7E-02
chr13:215719675-215721719	2045	8.2	8.1	8.3	↓	-0.2	3.7E-04	7.5E-02
chr13:216783215-216784681	1467	6.8	6.6	6.9	↓	-0.2	5.1E-04	9.1E-02
chr13:217472058-217473352	1295	4.8	5.0	4.6	↑	0.4	5.3E-04	9.2E-02
chr13:218006320-218011510	5191	8.3	8.2	8.4	↓	-0.2	1.5E-04	4.1E-02
chr13:23871273-23871593	321	2.4	1.8	2.8	↓	-1.1	3.5E-04	7.2E-02
chr13:25002024-25002924	901	4.8	4.4	5.1	↓	-0.7	1.8E-04	4.6E-02
chr13:25621353-25621910	558	2.8	3.1	2.3	↑	0.9	2.4E-04	5.6E-02
chr13:27828490-27829815	1326	4.4	4.0	4.7	↓	-0.7	1.5E-05	7.9E-03
chr13:27986016-27986311	296	2.4	2.8	1.8	↑	1.0	1.6E-04	4.4E-02
chr13:29361420-29361778	359	2.4	2.9	1.9	↑	1.0	2.6E-04	5.9E-02
chr13:30527257-30528115	859	3.6	3.9	3.3	↑	0.6	5.3E-04	9.2E-02
chr13:34045521-34045793	273	1.9	2.3	1.2	↑	1.2	4.5E-04	8.6E-02
chr13:36024916-36027454	2539	6.5	6.3	6.6	↓	-0.3	5.4E-04	9.3E-02
chr13:37327790-37328196	407	2.2	1.6	2.7	↓	-1.0	5.3E-04	9.2E-02
chr13:3827374-3827727	354	2.7	3.1	2.2	↑	0.9	3.7E-04	7.5E-02
chr13:39183706-39184032	327	2.0	1.3	2.5	↓	-1.1	5.2E-04	9.2E-02
chr13:40610403-40611797	1395	4.8	4.5	5.0	↓	-0.5	5.3E-05	2.0E-02
chr13:40936288-40937045	758	3.1	2.3	3.6	↓	-1.2	6.4E-07	6.4E-04

chr13:41500774-41501081	308	1.8	1.0	2.3	↓	-1.3	3.6E-04	7.3E-02
chr13:41620617-41621189	573	2.7	1.6	3.2	↓	-1.6	1.5E-08	3.2E-05
chr13:44593273-44593534	262	1.7	2.2	0.9	↑	1.4	3.2E-04	6.8E-02
chr13:45841928-45842499	572	3.7	3.4	4.0	↓	-0.7	4.7E-04	8.6E-02
chr13:47681982-47682598	617	4.0	3.6	4.2	↓	-0.6	3.0E-04	6.5E-02
chr13:47690534-47691645	1112	5.0	4.7	5.2	↓	-0.5	9.5E-05	3.0E-02
chr13:50699842-50701304	1463	4.8	5.1	4.5	↑	0.5	7.6E-06	4.9E-03
chr13:52652748-52653927	1180	5.0	4.6	5.3	↓	-0.7	3.6E-08	6.8E-05
chr13:52676747-52677240	494	3.6	3.0	4.1	↓	-1.1	1.6E-07	2.0E-04
chr13:54166986-54167162	177	1.7	1.0	2.2	↓	-1.2	6.0E-04	1.0E-01
chr13:55150582-55151357	776	3.5	3.0	3.9	↓	-0.8	9.4E-05	3.0E-02
chr13:55818008-55818370	363	2.5	2.9	1.9	↑	1.0	4.4E-04	8.6E-02
chr13:55936422-55937008	587	3.7	3.2	4.0	↓	-0.8	3.3E-04	7.0E-02
chr13:56111802-56112286	485	2.4	1.4	2.9	↓	-1.5	1.1E-07	1.4E-04
chr13:56516601-56517544	944	4.0	3.6	4.3	↓	-0.6	1.7E-04	4.5E-02
chr13:57434522-57435760	1239	4.0	3.6	4.3	↓	-0.7	1.1E-05	6.0E-03
chr13:57563664-57564582	919	3.5	3.8	3.1	↑	0.6	5.5E-04	9.4E-02
chr13:57601647-57602174	528	3.4	3.0	3.8	↓	-0.8	2.2E-05	1.1E-02
chr13:58380789-58381321	533	3.1	2.4	3.6	↓	-1.2	8.6E-08	1.2E-04
chr13:58963630-58963946	317	2.5	2.9	1.8	↑	1.0	1.9E-04	4.8E-02
chr13:59115591-59116462	872	3.5	3.8	3.1	↑	0.7	1.9E-04	4.9E-02
chr13:59181215-59183484	2270	6.0	5.7	6.2	↓	-0.5	3.7E-05	1.6E-02
chr13:59611485-59611863	379	1.7	0.7	2.3	↓	-1.5	2.2E-05	1.1E-02
chr13:5966220-5966536	317	2.0	1.0	2.6	↓	-1.6	1.7E-06	1.4E-03
chr13:61293011-61293764	754	4.1	3.6	4.4	↓	-0.8	2.0E-05	1.0E-02
chr13:61551443-61551967	525	3.8	3.4	4.1	↓	-0.7	2.9E-05	1.4E-02
chr13:61626026-61626765	740	4.3	3.9	4.7	↓	-0.8	8.0E-05	2.7E-02
chr13:61671656-61672238	583	2.9	2.3	3.3	↓	-1.0	1.2E-04	3.7E-02
chr13:65793008-65793559	552	2.0	2.5	1.2	↑	1.3	1.1E-04	3.4E-02
chr13:65797202-65797544	343	2.1	2.7	1.1	↑	1.5	5.1E-06	3.5E-03
chr13:67300824-67301433	610	2.8	2.3	3.2	↓	-0.9	2.6E-04	5.8E-02
chr13:68820987-68821275	289	2.8	2.3	3.2	↓	-0.9	2.2E-04	5.4E-02
chr13:69529882-69530479	598	2.3	2.8	1.5	↑	1.3	6.2E-06	4.1E-03
chr13:70999744-71000035	292	1.5	2.1	0.5	↑	1.6	3.7E-05	1.6E-02
chr13:71679476-71679952	477	3.5	3.9	2.9	↑	1.0	1.4E-06	1.2E-03
chr13:73834906-73835569	664	3.1	2.6	3.5	↓	-0.9	1.4E-04	3.9E-02
chr13:74216542-74217152	611	4.4	4.0	4.8	↓	-0.8	2.9E-06	2.2E-03
chr13:75191705-75191972	268	2.6	3.0	2.0	↑	0.9	4.6E-04	8.6E-02
chr13:75630601-75631170	570	3.7	3.2	4.0	↓	-0.9	1.7E-06	1.3E-03
chr13:76004396-76004781	386	3.6	3.2	3.9	↓	-0.7	3.8E-05	1.7E-02
chr13:76345507-76345889	383	2.6	2.1	3.0	↓	-0.9	3.3E-04	6.9E-02
chr13:7689943-7690329	387	2.1	1.4	2.6	↓	-1.2	1.4E-04	3.9E-02
chr13:77534346-77534717	372	2.3	2.8	1.6	↑	1.1	1.0E-04	3.2E-02
chr13:77590710-77591408	699	3.3	3.6	2.9	↑	0.7	3.3E-04	6.9E-02
chr13:77664567-77665734	1168	4.3	4.7	3.9	↑	0.8	4.5E-04	8.6E-02
chr13:77667299-77668209	911	4.0	4.2	3.7	↑	0.6	3.2E-04	6.8E-02
chr13:78702985-78704016	1032	5.4	5.2	5.6	↓	-0.4	1.2E-04	3.7E-02

chr13:78836958-78837656	699	4.2	3.8	4.5	↓	-0.7	2.9E-04	6.3E-02
chr13:79103598-79105929	2332	6.4	6.2	6.5	↓	-0.3	3.9E-05	1.7E-02
chr13:79564563-79565311	749	4.5	4.2	4.8	↓	-0.6	1.2E-04	3.7E-02
chr13:79573287-79574593	1307	6.0	5.7	6.2	↓	-0.5	4.5E-04	8.6E-02
chr13:79913139-79913729	591	4.2	3.8	4.6	↓	-0.8	1.8E-05	9.0E-03
chr13:7991991-7993871	1881	5.6	5.8	5.3	↑	0.6	5.4E-05	2.0E-02
chr13:7995932-7996794	863	4.0	4.4	3.4	↑	1.0	1.0E-08	2.3E-05
chr13:8049226-8049624	399	1.9	2.4	1.1	↑	1.3	1.5E-04	4.0E-02
chr13:81635055-81635575	521	2.7	2.1	3.1	↓	-1.0	3.9E-04	7.7E-02
chr13:82796371-82798104	1734	5.0	4.7	5.2	↓	-0.5	1.9E-04	4.9E-02
chr13:82831283-82831701	419	3.4	2.6	3.9	↓	-1.3	6.6E-10	2.2E-06
chr13:83261585-83262057	473	2.8	2.2	3.2	↓	-1.0	5.1E-05	2.0E-02
chr13:83269651-83270603	953	3.1	3.4	2.6	↑	0.8	5.4E-04	9.3E-02
chr13:83679130-83679409	280	1.7	2.2	0.9	↑	1.3	1.4E-04	3.9E-02
chr13:86949560-86951780	2221	5.9	5.7	6.0	↓	-0.3	4.6E-05	1.8E-02
chr13:87344439-87345648	1210	4.2	3.9	4.4	↓	-0.6	9.6E-05	3.0E-02
chr13:87348389-87350628	2240	5.8	5.6	6.0	↓	-0.3	1.8E-04	4.7E-02
chr13:88831926-88833265	1340	6.1	5.8	6.4	↓	-0.6	1.8E-07	2.1E-04
chr13:89275482-89275938	457	2.8	3.2	2.2	↑	1.0	1.2E-04	3.7E-02
chr13:90114253-90114614	362	3.5	3.1	3.8	↓	-0.6	4.6E-04	8.6E-02
chr13:90308646-90309204	559	3.3	2.8	3.6	↓	-0.8	5.9E-05	2.2E-02
chr13:90325420-90325828	409	2.9	2.4	3.3	↓	-0.9	5.0E-04	9.0E-02
chr13:9105407-9105950	544	2.3	1.6	2.8	↓	-1.2	3.9E-05	1.7E-02
chr13:91734546-91735675	1130	5.1	4.9	5.2	↓	-0.4	3.5E-04	7.3E-02
chr13:91856353-91857320	968	3.8	4.1	3.3	↑	0.8	2.7E-04	6.1E-02
chr13:91966360-91966843	484	3.0	3.4	2.5	↑	0.9	3.6E-05	1.6E-02
chr13:91970332-91970717	386	2.0	2.5	1.2	↑	1.4	3.9E-05	1.7E-02
chr13:93041703-93042006	304	1.8	2.3	1.1	↑	1.3	3.8E-04	7.6E-02
chr13:97333050-97333645	596	3.1	1.9	3.7	↓	-1.8	4.0E-13	2.3E-09
chr13:97333761-97334049	289	2.1	1.3	2.7	↓	-1.4	2.7E-05	1.3E-02
chr13:97638294-97639312	1019	3.1	2.6	3.5	↓	-0.9	2.6E-04	5.8E-02
chr13:99136405-99136782	378	2.1	1.3	2.7	↓	-1.4	9.5E-06	5.5E-03
chr13:99297949-99298404	456	2.0	1.2	2.5	↓	-1.3	7.1E-05	2.5E-02
chr14:100352856-100353638	783	5.2	4.9	5.4	↓	-0.5	8.6E-06	3.8E-03
chr14:100353714-100354115	402	2.6	2.1	2.9	↓	-0.9	6.0E-04	8.3E-02
chr14:100355416-100355818	403	2.8	2.1	3.3	↓	-1.1	3.1E-06	1.8E-03
chr14:100376484-100376832	349	3.0	2.4	3.4	↓	-0.9	3.4E-05	1.2E-02
chr14:100394875-100395221	347	3.6	2.6	4.1	↓	-1.5	7.0E-10	1.5E-06
chr14:100425068-100426764	1697	6.8	6.6	6.9	↓	-0.4	1.7E-04	3.9E-02
chr14:103347649-103347986	338	2.2	2.6	1.5	↑	1.1	2.4E-04	4.9E-02
chr14:106394604-106394983	380	2.8	2.2	3.3	↓	-1.1	6.9E-06	3.4E-03
chr14:106478842-106479473	632	2.7	1.9	3.2	↓	-1.3	3.3E-07	2.9E-04
chr14:106479515-106479899	385	2.4	1.8	2.8	↓	-1.0	3.1E-04	5.6E-02
chr14:107593739-107594013	275	3.0	2.5	3.3	↓	-0.8	6.6E-04	8.8E-02
chr14:107927738-107928083	346	2.7	3.1	2.1	↑	0.9	4.5E-04	7.1E-02
chr14:108405171-108406066	896	4.2	3.8	4.5	↓	-0.7	8.3E-05	2.3E-02
chr14:109203563-109203885	323	1.9	2.4	1.0	↑	1.4	7.0E-05	2.0E-02

chr14:109963652-109964369	718	3.3	3.7	2.9	↑	0.8	1.6E-04	3.8E-02
chr14:11010537-11011893	1357	5.4	5.2	5.6	↓	-0.5	1.3E-04	3.3E-02
chr14:11064819-11065855	1037	4.5	4.0	4.9	↓	-0.8	3.8E-08	5.0E-05
chr14:11065912-11068154	2243	6.2	5.7	6.6	↓	-0.8	3.2E-12	1.5E-08
chr14:11068267-11068763	497	2.9	2.2	3.3	↓	-1.1	3.2E-06	1.8E-03
chr14:111759374-111759720	347	2.1	1.5	2.6	↓	-1.1	2.5E-04	4.9E-02
chr14:113931712-113932171	460	3.1	2.6	3.5	↓	-0.9	5.1E-05	1.6E-02
chr14:114202827-114203725	899	3.6	3.2	4.0	↓	-0.8	8.6E-06	3.8E-03
chr14:115017752-115018260	509	3.1	2.5	3.6	↓	-1.2	6.9E-05	2.0E-02
chr14:115123108-115123361	254	1.9	1.3	2.4	↓	-1.2	2.8E-04	5.2E-02
chr14:116625914-116626672	759	3.7	3.3	4.0	↓	-0.7	4.8E-04	7.3E-02
chr14:11671181-11671683	503	3.8	4.0	3.4	↑	0.6	6.2E-04	8.5E-02
chr14:116852276-116852616	341	2.3	1.6	2.7	↓	-1.1	4.7E-04	7.3E-02
chr14:11689503-11690743	1241	5.6	5.4	5.7	↓	-0.4	1.1E-04	2.7E-02
chr14:117603895-117604458	564	4.0	3.6	4.2	↓	-0.6	5.1E-04	7.7E-02
chr14:117760267-117760606	340	3.1	2.7	3.5	↓	-0.8	7.1E-04	9.2E-02
chr14:117915822-117916020	199	1.5	0.5	2.1	↓	-1.6	8.7E-05	2.4E-02
chr14:117971909-117972160	252	1.6	2.1	0.8	↑	1.3	4.0E-04	6.6E-02
chr14:118304269-118304755	487	3.0	3.4	2.6	↑	0.8	6.9E-04	9.1E-02
chr14:118349987-118350369	383	2.4	2.9	1.8	↑	1.0	1.5E-04	3.6E-02
chr14:120102148-120102419	272	1.9	1.2	2.4	↓	-1.1	5.2E-04	7.7E-02
chr14:120154656-120156793	2138	6.3	5.8	6.7	↓	-0.9	8.8E-13	5.0E-09
chr14:120170244-120171636	1393	4.6	3.7	5.2	↓	-1.5	4.0E-12	1.5E-08
chr14:120171958-120172427	470	2.5	1.5	3.1	↓	-1.6	2.8E-08	3.9E-05
chr14:120176737-120177274	538	2.6	1.9	3.0	↓	-1.1	1.7E-05	6.9E-03
chr14:120186281-120186644	364	2.2	1.5	2.6	↓	-1.1	4.2E-04	6.8E-02
chr14:120241427-120242557	1131	6.0	6.2	5.8	↑	0.4	7.9E-09	1.4E-05
chr14:121931863-121933359	1497	5.2	5.6	4.8	↑	0.8	7.8E-07	5.7E-04
chr14:121952010-121952363	354	2.3	1.3	2.9	↓	-1.6	2.1E-07	2.0E-04
chr14:121953176-121954614	1439	5.9	6.2	5.5	↑	0.8	8.6E-11	2.7E-07
chr14:121954942-121956579	1638	5.3	5.5	5.0	↑	0.5	2.1E-05	8.2E-03
chr14:121958335-121960363	2029	5.5	5.7	5.2	↑	0.5	1.4E-05	5.9E-03
chr14:122174373-122174716	344	2.7	3.0	2.2	↑	0.9	6.4E-04	8.6E-02
chr14:123038066-123038491	426	3.0	2.5	3.3	↓	-0.8	2.8E-04	5.2E-02
chr14:12326886-12327245	360	3.7	3.2	4.0	↓	-0.8	7.1E-05	2.0E-02
chr14:12388001-12388504	504	4.3	4.0	4.6	↓	-0.6	2.9E-05	1.0E-02
chr14:124456577-124457624	1048	4.9	4.5	5.3	↓	-0.8	1.4E-08	2.4E-05
chr14:124463130-124463718	589	4.1	3.7	4.4	↓	-0.6	4.0E-04	6.6E-02
chr14:124620113-124620561	449	2.9	2.4	3.3	↓	-0.9	4.9E-04	7.5E-02
chr14:125485486-125485926	441	2.5	1.8	3.0	↓	-1.2	2.6E-05	9.4E-03
chr14:126371633-126371991	359	3.6	3.1	4.0	↓	-0.9	4.1E-05	1.3E-02
chr14:12854366-12855083	718	4.8	4.4	5.1	↓	-0.7	4.5E-07	3.7E-04
chr14:12899234-12899671	438	3.5	3.2	3.9	↓	-0.7	1.7E-04	3.8E-02
chr14:130453329-130453593	265	3.7	3.4	4.0	↓	-0.6	2.7E-04	5.2E-02
chr14:130461079-130461483	405	2.4	1.8	2.9	↓	-1.1	2.0E-04	4.2E-02
chr14:130461644-130461848	205	1.8	0.8	2.4	↓	-1.5	1.3E-05	5.4E-03
chr14:130461936-130462389	454	2.9	1.8	3.5	↓	-1.6	6.5E-10	1.5E-06

chr14:130463743-130464488	746	2.8	2.1	3.3	↓	-1.2	1.1E-06	7.6E-04
chr14:130464538-130464879	342	3.3	2.2	3.9	↓	-1.7	6.8E-13	4.3E-09
chr14:131102571-131103264	694	4.0	3.5	4.3	↓	-0.8	5.4E-06	2.8E-03
chr14:132404047-132405639	1593	5.0	4.4	5.5	↓	-1.1	5.8E-09	1.1E-05
chr14:133027718-133028062	345	2.0	1.2	2.5	↓	-1.3	2.1E-04	4.4E-02
chr14:133131870-133132165	296	2.5	1.9	2.9	↓	-0.9	5.0E-04	7.5E-02
chr14:133281605-133281833	229	2.1	1.4	2.5	↓	-1.1	5.7E-04	8.0E-02
chr14:133284877-133285144	268	2.5	1.9	2.9	↓	-0.9	3.7E-04	6.3E-02
chr14:13334443-13335131	689	3.8	4.0	3.5	↑	0.6	8.1E-04	1.0E-01
chr14:133489181-133489837	657	4.0	3.5	4.3	↓	-0.8	2.3E-05	8.4E-03
chr14:134374923-134375505	583	3.5	3.2	3.8	↓	-0.6	8.0E-04	9.9E-02
chr14:134383252-134383568	317	2.6	2.1	3.0	↓	-0.9	4.0E-04	6.6E-02
chr14:134391800-134393156	1357	5.1	4.9	5.3	↓	-0.4	1.7E-04	3.9E-02
chr14:134693012-134693223	212	1.8	1.0	2.3	↓	-1.3	2.5E-04	4.9E-02
chr14:134702274-134703911	1638	5.7	5.5	6.0	↓	-0.5	1.3E-05	5.4E-03
chr14:134730757-134731356	600	3.8	3.3	4.2	↓	-0.9	5.0E-05	1.6E-02
chr14:134766618-134767373	756	4.5	4.7	4.1	↑	0.6	7.0E-06	3.4E-03
chr14:135045068-135045384	317	2.1	1.5	2.6	↓	-1.0	7.4E-04	9.4E-02
chr14:135567452-135568640	1189	5.5	5.3	5.7	↓	-0.4	9.8E-05	2.6E-02
chr14:135850390-135851126	737	4.5	4.7	4.3	↑	0.5	5.2E-04	7.7E-02
chr14:135864234-135864831	598	3.2	2.8	3.6	↓	-0.8	3.5E-05	1.2E-02
chr14:13731573-13732084	512	3.5	3.1	3.8	↓	-0.7	1.1E-04	2.9E-02
chr14:137640751-137641491	741	4.2	4.5	4.0	↑	0.5	7.6E-04	9.6E-02
chr14:138803119-138803609	491	2.3	2.7	1.6	↑	1.1	1.5E-04	3.5E-02
chr14:138948958-138949855	898	4.8	4.6	5.0	↓	-0.4	2.3E-04	4.6E-02
chr14:139603437-139603772	336	3.6	3.1	4.0	↓	-0.9	5.4E-06	2.8E-03
chr14:139721011-139721457	447	2.6	3.0	2.1	↑	0.9	8.2E-04	1.0E-01
chr14:140080739-140081147	409	3.4	2.6	3.9	↓	-1.3	3.1E-10	8.1E-07
chr14:140255071-140255403	333	2.6	3.0	2.0	↑	1.0	1.2E-04	2.9E-02
chr14:140729408-140729904	497	3.9	3.5	4.1	↓	-0.6	7.0E-04	9.1E-02
chr14:140807472-140808136	665	5.7	5.5	5.9	↓	-0.3	5.5E-04	7.9E-02
chr14:140920900-140922122	1223	4.8	4.5	5.0	↓	-0.5	1.3E-04	3.2E-02
chr14:141014959-141015453	495	3.4	3.0	3.7	↓	-0.8	2.8E-04	5.2E-02
chr14:141705328-141705692	365	3.2	2.8	3.6	↓	-0.8	3.9E-04	6.6E-02
chr14:142181388-142181642	255	2.6	1.9	3.0	↓	-1.1	4.4E-05	1.4E-02
chr14:142415954-142416542	589	3.9	3.5	4.1	↓	-0.6	3.2E-04	5.6E-02
chr14:142417898-142418659	762	5.4	5.1	5.6	↓	-0.5	7.8E-05	2.2E-02
chr14:143013003-143014220	1218	4.8	5.1	4.4	↑	0.8	2.5E-06	1.5E-03
chr14:143051410-143054247	2838	6.4	6.6	6.3	↑	0.3	2.9E-04	5.3E-02
chr14:14320645-14320925	281	2.5	1.7	3.0	↓	-1.3	1.7E-06	1.0E-03
chr14:14321216-14321570	355	2.3	1.6	2.8	↓	-1.1	1.7E-04	3.8E-02
chr14:143551925-143552368	444	2.9	3.2	2.4	↑	0.8	2.9E-04	5.4E-02
chr14:143718929-143719393	465	2.6	2.9	2.0	↑	0.9	5.3E-04	7.7E-02
chr14:144651383-144651849	467	4.0	3.7	4.3	↓	-0.6	1.2E-04	2.9E-02
chr14:145022803-145024124	1322	5.2	5.0	5.4	↓	-0.4	5.1E-04	7.7E-02
chr14:1451329-1451757	429	3.2	2.7	3.5	↓	-0.8	1.7E-04	3.9E-02
chr14:145172866-145173297	432	3.2	3.7	2.4	↑	1.3	2.0E-09	4.0E-06

chr14:145173343-145175661	2319	5.6	5.9	5.3	↑	0.6	8.9E-07	6.3E-04
chr14:145186837-145189206	2370	6.2	6.4	6.0	↑	0.4	3.8E-06	2.1E-03
chr14:145214197-145215479	1283	4.3	4.6	4.0	↑	0.6	4.2E-05	1.4E-02
chr14:1456683-1457535	853	4.1	3.6	4.4	↓	-0.8	4.5E-07	3.7E-04
chr14:145973871-145975133	1263	5.5	5.2	5.7	↓	-0.5	1.2E-04	2.9E-02
chr14:146070923-146071359	437	2.7	3.1	2.1	↑	1.0	2.1E-04	4.4E-02
chr14:146302031-146302996	966	5.4	5.2	5.6	↓	-0.4	5.2E-04	7.7E-02
chr14:146699522-146699763	242	2.3	1.8	2.7	↓	-1.0	8.0E-04	9.9E-02
chr14:146812286-146814190	1905	6.7	6.5	6.8	↓	-0.3	1.8E-04	4.1E-02
chr14:147238394-147238890	497	3.4	3.0	3.7	↓	-0.7	6.4E-04	8.6E-02
chr14:147250713-147251178	466	3.1	2.7	3.4	↓	-0.7	4.9E-04	7.5E-02
chr14:147631000-147632630	1631	6.4	6.2	6.6	↓	-0.4	1.0E-05	4.4E-03
chr14:147831186-147831963	778	4.8	5.0	4.5	↑	0.5	2.5E-04	4.9E-02
chr14:149002153-149003009	857	4.9	4.7	5.1	↓	-0.5	6.5E-04	8.7E-02
chr14:149251437-149252018	582	4.0	3.4	4.4	↓	-1.0	8.5E-10	1.8E-06
chr14:149321661-149322499	839	4.6	4.8	4.3	↑	0.5	1.1E-04	2.8E-02
chr14:149331196-149332932	1737	5.9	5.4	6.3	↓	-0.8	9.2E-15	1.2E-10
chr14:149419494-149419856	363	2.6	3.0	1.9	↑	1.1	1.9E-04	4.2E-02
chr14:149578699-149579356	658	3.9	3.6	4.2	↓	-0.6	6.8E-04	8.9E-02
chr14:149866599-149867098	500	4.2	3.8	4.5	↓	-0.7	2.3E-04	4.7E-02
chr14:149872613-149873532	920	5.0	4.5	5.3	↓	-0.8	7.3E-06	3.4E-03
chr14:150101241-150101666	426	2.7	1.9	3.2	↓	-1.3	1.6E-06	9.8E-04
chr14:150240143-150240384	242	2.6	2.0	3.1	↓	-1.1	6.7E-05	2.0E-02
chr14:150558232-150560131	1900	6.7	6.4	6.9	↓	-0.4	1.5E-08	2.5E-05
chr14:150701844-150702191	348	3.1	2.6	3.4	↓	-0.8	5.3E-04	7.7E-02
chr14:150730227-150730718	492	3.8	3.4	4.1	↓	-0.7	2.8E-04	5.2E-02
chr14:150886921-150892824	5904	8.8	8.5	9.0	↓	-0.5	5.7E-18	1.5E-13
chr14:150902771-150905129	2359	7.9	7.7	8.0	↓	-0.2	4.6E-04	7.2E-02
chr14:150934252-150936431	2180	6.7	6.5	6.8	↓	-0.3	3.2E-04	5.6E-02
chr14:150968259-150969736	1478	5.6	5.4	5.8	↓	-0.4	3.0E-04	5.4E-02
chr14:150985958-150986363	406	3.4	2.9	3.8	↓	-0.9	1.0E-04	2.6E-02
chr14:151005007-151008055	3049	7.7	7.5	7.8	↓	-0.3	2.5E-08	3.6E-05
chr14:151269096-151272113	3018	7.4	7.3	7.5	↓	-0.2	3.2E-04	5.6E-02
chr14:151273541-151274333	793	4.8	4.3	5.1	↓	-0.7	1.2E-07	1.2E-04
chr14:151346207-151349996	3790	7.8	7.6	7.9	↓	-0.4	1.5E-06	9.6E-04
chr14:151361749-151362076	328	2.5	1.9	2.9	↓	-0.9	6.2E-04	8.4E-02
chr14:151365008-151365902	895	5.3	5.0	5.5	↓	-0.4	7.1E-04	9.2E-02
chr14:151385970-151387216	1247	5.5	5.2	5.7	↓	-0.5	1.2E-06	8.2E-04
chr14:151467796-151468972	1177	6.3	6.0	6.5	↓	-0.5	8.3E-06	3.7E-03
chr14:151556413-151557967	1555	6.7	6.4	6.8	↓	-0.4	3.4E-06	1.9E-03
chr14:151564742-151566396	1655	6.2	6.0	6.3	↓	-0.3	1.9E-04	4.2E-02
chr14:151603797-151605507	1711	6.5	6.4	6.6	↓	-0.2	8.1E-04	1.0E-01
chr14:151634068-151636487	2420	7.1	7.0	7.2	↓	-0.2	5.0E-04	7.6E-02
chr14:151700651-151702852	2202	6.2	6.0	6.4	↓	-0.4	5.5E-04	7.9E-02
chr14:151755008-151755379	372	3.0	2.5	3.4	↓	-0.9	8.4E-05	2.3E-02
chr14:151797692-151799018	1327	6.5	6.3	6.6	↓	-0.3	1.7E-05	6.8E-03
chr14:151810863-151813989	3127	7.2	7.1	7.3	↓	-0.2	1.1E-04	2.8E-02

chr14:151902842-151904704	1863	6.5	6.3	6.6	↓	-0.2	1.6E-04	3.7E-02
chr14:151950450-151951449	1000	5.0	4.6	5.4	↓	-0.8	2.2E-06	1.3E-03
chr14:151986484-151998368	11885	9.3	9.2	9.3	↓	-0.1	7.6E-04	9.6E-02
chr14:152003738-152010365	6628	8.7	8.6	8.8	↓	-0.2	3.4E-07	3.0E-04
chr14:152021460-152023031	1572	6.1	5.9	6.3	↓	-0.3	2.1E-04	4.4E-02
chr14:152213140-152216623	3484	7.4	7.2	7.5	↓	-0.3	7.3E-08	8.5E-05
chr14:152216781-152221032	4252	8.3	8.2	8.5	↓	-0.3	5.1E-06	2.7E-03
chr14:152246914-152248276	1363	5.6	5.4	5.8	↓	-0.4	2.6E-04	5.0E-02
chr14:152268382-152276368	7987	9.1	9.0	9.1	↓	-0.2	2.1E-04	4.4E-02
chr14:152277415-152278807	1393	5.5	5.2	5.7	↓	-0.5	2.8E-07	2.6E-04
chr14:15287519-15289536	2018	5.7	5.9	5.5	↑	0.4	8.1E-04	9.9E-02
chr14:152932107-152939378	7272	8.7	8.6	8.8	↓	-0.1	3.7E-05	1.2E-02
chr14:153795552-153799743	4192	7.7	7.6	7.8	↓	-0.2	6.8E-05	2.0E-02
chr14:15483332-15483747	416	3.1	2.6	3.4	↓	-0.8	6.0E-04	8.3E-02
chr14:15527044-15527433	390	3.9	4.2	3.6	↑	0.6	5.5E-04	7.9E-02
chr14:1592393-1592973	581	4.0	4.2	3.6	↑	0.6	4.5E-04	7.1E-02
chr14:15961079-15961449	371	3.2	2.8	3.6	↓	-0.8	4.2E-04	6.8E-02
chr14:16017516-16017861	346	2.1	2.5	1.4	↑	1.1	2.5E-04	4.9E-02
chr14:16049621-16050256	636	2.3	3.0	1.2	↑	1.7	4.0E-07	3.4E-04
chr14:16279463-16280541	1079	5.1	4.7	5.4	↓	-0.7	4.2E-08	5.5E-05
chr14:17320624-17321180	557	2.7	3.1	2.2	↑	0.9	8.2E-04	1.0E-01
chr14:17510978-17512244	1267	4.8	5.2	4.4	↑	0.7	3.8E-08	5.0E-05
chr14:17512993-17513404	412	3.5	4.1	2.5	↑	1.5	1.1E-12	5.5E-09
chr14:17517541-17518166	626	3.0	3.5	2.3	↑	1.2	5.5E-07	4.3E-04
chr14:17518670-17519234	565	2.5	3.0	1.7	↑	1.3	1.5E-04	3.6E-02
chr14:17521264-17521750	487	2.2	2.7	1.4	↑	1.4	2.2E-05	8.3E-03
chr14:17525206-17525625	420	2.6	3.1	2.0	↑	1.1	3.8E-05	1.3E-02
chr14:17526386-17527856	1471	4.9	5.4	4.1	↑	1.2	2.5E-15	4.2E-11
chr14:17532357-17533224	868	3.5	4.0	2.8	↑	1.3	9.2E-08	1.0E-04
chr14:17537156-17537500	345	2.1	2.6	1.5	↑	1.1	2.9E-04	5.3E-02
chr14:17538467-17539013	547	2.4	2.9	1.5	↑	1.4	1.3E-06	8.5E-04
chr14:17605865-17606228	364	4.0	3.7	4.3	↓	-0.6	3.7E-04	6.3E-02
chr14:17936020-17936469	450	3.0	3.3	2.5	↑	0.9	2.0E-04	4.3E-02
chr14:19268995-19269284	290	2.2	1.5	2.7	↓	-1.2	5.0E-05	1.6E-02
chr14:19576354-19578887	2534	6.3	6.0	6.5	↓	-0.5	2.1E-08	3.1E-05
chr14:20066438-20066667	230	1.7	2.2	0.9	↑	1.2	5.0E-04	7.5E-02
chr14:21675541-21675999	459	3.3	2.3	3.9	↓	-1.6	2.6E-10	7.4E-07
chr14:21930628-21931808	1181	6.4	6.2	6.5	↓	-0.3	1.9E-04	4.2E-02
chr14:22021034-22021639	606	4.6	4.3	4.9	↓	-0.6	5.2E-05	1.6E-02
chr14:22045766-22046958	1193	5.5	5.2	5.7	↓	-0.4	1.5E-04	3.6E-02
chr14:22100641-22102424	1784	5.4	5.2	5.6	↓	-0.4	2.9E-04	5.4E-02
chr14:23445321-23445653	333	1.9	1.2	2.4	↓	-1.2	4.3E-04	6.9E-02
chr14:252506-252698	193	2.1	1.4	2.6	↓	-1.3	2.4E-05	8.8E-03
chr14:25771518-25771755	238	2.4	1.7	2.8	↓	-1.2	3.8E-05	1.3E-02
chr14:25841559-25841825	267	2.0	2.4	1.3	↑	1.1	7.6E-04	9.6E-02
chr14:26102433-26103231	799	3.9	3.4	4.2	↓	-0.8	8.4E-05	2.3E-02
chr14:26546145-26546489	345	3.5	2.9	3.9	↓	-1.0	6.2E-06	3.2E-03

chr14:27815932-27816516	585	3.4	2.9	3.8	↓	-0.8	5.3E-05	1.6E-02
chr14:28179979-28180362	384	2.5	1.7	3.0	↓	-1.4	7.0E-07	5.2E-04
chr14:28522518-28523008	491	3.9	3.3	4.3	↓	-1.1	1.8E-08	2.9E-05
chr14:28527264-28527622	359	2.2	1.6	2.6	↓	-1.0	4.7E-04	7.2E-02
chr14:28679861-28680345	485	2.5	2.9	2.0	↑	0.9	3.3E-04	5.8E-02
chr14:29343396-29343981	586	4.3	3.5	4.8	↓	-1.3	4.5E-11	1.5E-07
chr14:29643059-29643434	376	3.3	2.7	3.8	↓	-1.1	1.4E-06	9.0E-04
chr14:29992887-29993324	438	2.2	2.6	1.6	↑	1.1	2.6E-04	5.0E-02
chr14:30233291-30233701	411	3.0	3.3	2.5	↑	0.8	5.9E-04	8.2E-02
chr14:30337505-30338352	848	5.7	5.5	5.9	↓	-0.5	1.7E-04	3.9E-02
chr14:30556522-30557777	1256	6.8	6.6	7.0	↓	-0.4	9.9E-07	6.9E-04
chr14:30620099-30620589	491	4.6	4.4	4.8	↓	-0.5	8.7E-05	2.4E-02
chr14:30632325-30633143	819	3.8	3.4	4.1	↓	-0.7	2.4E-05	8.8E-03
chr14:31323331-31323952	622	3.6	3.2	3.9	↓	-0.7	2.5E-04	4.9E-02
chr14:31340861-31341984	1124	5.8	5.5	6.0	↓	-0.5	8.2E-07	5.8E-04
chr14:3263621-3264509	889	3.9	4.2	3.5	↑	0.8	5.9E-05	1.8E-02
chr14:3264853-3266822	1970	6.6	6.7	6.5	↑	0.2	1.9E-04	4.2E-02
chr14:35126700-35126937	238	1.4	1.9	0.6	↑	1.3	8.0E-04	9.9E-02
chr14:35949466-35949785	320	2.8	2.2	3.2	↓	-1.0	5.6E-04	7.9E-02
chr14:36052231-36053302	1072	5.1	4.8	5.3	↓	-0.5	6.6E-06	3.3E-03
chr14:37067068-37067517	450	2.9	2.2	3.3	↓	-1.1	7.1E-06	3.4E-03
chr14:37067636-37068167	532	4.3	3.9	4.7	↓	-0.8	3.0E-07	2.8E-04
chr14:37105266-37105874	609	4.1	3.8	4.3	↓	-0.6	4.5E-04	7.1E-02
chr14:37143967-37144850	884	4.9	4.5	5.2	↓	-0.8	5.1E-08	6.5E-05
chr14:37207648-37208827	1180	5.0	4.7	5.2	↓	-0.5	2.8E-04	5.2E-02
chr14:37343273-37344840	1568	5.8	5.6	5.9	↓	-0.3	7.9E-04	9.8E-02
chr14:37652895-37653872	978	3.7	3.4	4.0	↓	-0.6	1.4E-04	3.4E-02
chr14:37692374-37692661	288	2.3	2.7	1.7	↑	1.0	5.3E-04	7.7E-02
chr14:37715856-37716066	211	1.6	2.1	0.9	↑	1.2	6.3E-04	8.6E-02
chr14:38233274-38233561	288	2.9	2.4	3.2	↓	-0.8	5.6E-04	7.9E-02
chr14:38588323-38590157	1835	5.1	4.8	5.3	↓	-0.5	5.0E-06	2.6E-03
chr14:38810625-38811000	376	4.0	3.7	4.3	↓	-0.6	3.6E-04	6.2E-02
chr14:39056387-39056831	445	3.6	3.3	3.9	↓	-0.7	3.0E-04	5.4E-02
chr14:39632141-39632413	273	2.7	2.1	3.1	↓	-1.0	4.9E-05	1.6E-02
chr14:39912155-39912954	800	4.0	3.6	4.3	↓	-0.7	6.9E-05	2.0E-02
chr14:40021871-40022802	932	5.6	5.3	5.9	↓	-0.6	3.1E-07	2.8E-04
chr14:40388754-40389305	552	3.4	2.8	3.8	↓	-0.9	8.0E-07	5.8E-04
chr14:40449502-40450014	513	3.6	3.1	4.0	↓	-0.9	1.6E-06	9.9E-04
chr14:40492621-40493164	544	3.1	2.4	3.5	↓	-1.1	1.5E-06	9.6E-04
chr14:40675673-40677014	1342	4.2	3.8	4.6	↓	-0.8	6.8E-08	8.3E-05
chr14:40894828-40895756	929	4.0	3.7	4.3	↓	-0.6	2.7E-04	5.1E-02
chr14:41105555-41106008	454	2.3	1.6	2.8	↓	-1.2	2.4E-04	4.8E-02
chr14:41583725-41584158	434	4.1	3.7	4.4	↓	-0.7	8.0E-06	3.7E-03
chr14:42686721-42687274	554	3.4	3.7	2.9	↑	0.8	5.0E-04	7.5E-02
chr14:42736632-42737201	570	4.0	3.4	4.4	↓	-1.0	1.9E-08	3.0E-05
chr14:43019945-43020592	648	5.9	5.6	6.1	↓	-0.5	1.5E-05	6.2E-03
chr14:43022891-43023336	446	3.6	3.2	3.9	↓	-0.7	4.6E-04	7.2E-02

chr14:43049882-43050496	615	4.2	3.8	4.5	↓	-0.6	4.1E-04	6.8E-02
chr14:44336599-44337442	844	4.0	4.3	3.7	↑	0.6	4.8E-04	7.3E-02
chr14:44414954-44415272	319	3.1	2.7	3.4	↓	-0.7	6.4E-04	8.6E-02
chr14:44785596-44785855	260	1.9	0.8	2.5	↓	-1.7	4.9E-07	4.0E-04
chr14:45786752-45787025	274	2.3	1.7	2.7	↓	-1.0	5.1E-04	7.7E-02
chr14:46390154-46390640	487	4.7	4.4	5.0	↓	-0.6	3.1E-05	1.1E-02
chr14:46484071-46484501	431	4.1	3.8	4.3	↓	-0.5	7.0E-04	9.1E-02
chr14:47476008-47478095	2088	5.9	5.6	6.1	↓	-0.5	5.8E-08	7.2E-05
chr14:475534-476177	644	3.6	3.3	3.9	↓	-0.6	3.4E-04	5.9E-02
chr14:47618664-47619048	385	2.8	2.3	3.2	↓	-0.9	2.8E-04	5.2E-02
chr14:47632448-47634290	1843	6.0	5.9	6.2	↓	-0.3	7.1E-04	9.2E-02
chr14:47635906-47636897	992	3.7	3.1	4.1	↓	-1.0	5.4E-07	4.3E-04
chr14:47637585-47639761	2177	6.0	5.6	6.3	↓	-0.7	4.5E-10	1.1E-06
chr14:47644670-47647381	2712	6.2	6.0	6.5	↓	-0.5	2.7E-14	2.7E-10
chr14:47986958-47989946	2989	8.0	7.7	8.3	↓	-0.5	3.5E-12	1.5E-08
chr14:47991496-47991937	442	3.6	2.8	4.1	↓	-1.3	4.9E-12	1.8E-08
chr14:49716863-49717697	835	5.8	6.0	5.6	↑	0.4	4.1E-06	2.3E-03
chr14:50273300-50273819	520	3.1	2.6	3.5	↓	-0.9	2.8E-05	1.0E-02
chr14:50300587-50300811	225	1.7	2.2	1.0	↑	1.2	6.9E-04	9.1E-02
chr14:50846765-50847982	1218	4.8	4.5	5.0	↓	-0.5	6.5E-05	1.9E-02
chr14:50884775-50885089	315	2.5	1.9	2.9	↓	-1.0	2.3E-04	4.7E-02
chr14:51176116-51176436	321	3.2	3.6	2.7	↑	0.9	2.0E-05	7.9E-03
chr14:51188321-51189644	1324	5.6	5.3	5.9	↓	-0.6	2.7E-07	2.6E-04
chr14:52844565-52845457	893	4.3	4.0	4.5	↓	-0.6	4.1E-05	1.3E-02
chr14:52919221-52919602	382	2.5	1.9	2.9	↓	-0.9	3.9E-04	6.5E-02
chr14:53070613-53071540	928	4.9	5.1	4.6	↑	0.4	3.4E-04	5.9E-02
chr14:53358528-53359204	677	4.2	3.9	4.5	↓	-0.7	3.0E-05	1.0E-02
chr14:5337972-5338394	423	3.3	2.9	3.6	↓	-0.7	4.0E-04	6.6E-02
chr14:53645436-53646475	1040	4.2	4.4	3.9	↑	0.5	7.6E-04	9.6E-02
chr14:53844189-53845323	1135	5.1	5.2	4.9	↑	0.4	7.6E-04	9.6E-02
chr14:54834145-54835000	856	5.3	5.0	5.6	↓	-0.6	1.7E-07	1.7E-04
chr14:54835497-54837667	2171	6.6	6.8	6.4	↑	0.5	6.5E-13	4.3E-09
chr14:54883483-54884316	834	5.5	5.2	5.7	↓	-0.6	9.8E-08	1.1E-04
chr14:54925037-54927740	2704	6.7	6.9	6.5	↑	0.4	2.0E-04	4.3E-02
chr14:54930601-54931169	569	2.8	2.1	3.3	↓	-1.2	1.7E-06	1.0E-03
chr14:54931194-54933446	2253	5.7	5.1	6.1	↓	-1.0	7.5E-20	3.8E-15
chr14:55151770-55153426	1657	6.6	6.4	6.8	↓	-0.4	8.4E-08	9.7E-05
chr14:55549453-55549749	297	2.9	3.2	2.4	↑	0.8	7.5E-04	9.6E-02
chr14:55996217-55996924	708	3.5	3.8	3.0	↑	0.7	2.2E-04	4.5E-02
chr14:56103250-56103419	170	1.6	2.1	0.8	↑	1.2	5.4E-04	7.9E-02
chr14:56104321-56104576	256	1.9	2.5	1.0	↑	1.5	8.0E-06	3.7E-03
chr14:57485673-57486463	791	4.7	4.2	5.1	↓	-0.9	1.1E-07	1.1E-04
chr14:58896323-58896726	404	2.2	2.7	1.6	↑	1.0	4.0E-04	6.6E-02
chr14:59029347-59029573	227	1.5	2.0	0.7	↑	1.3	4.1E-04	6.8E-02
chr14:59687658-59687911	254	2.2	2.7	1.3	↑	1.4	6.2E-06	3.2E-03
chr14:60367916-60368203	288	2.2	1.6	2.7	↓	-1.1	1.3E-04	3.3E-02
chr14:60470166-60471292	1127	5.1	4.8	5.3	↓	-0.5	4.3E-06	2.4E-03

chr14:60648635-60648958	324	2.9	2.3	3.3	↓	-1.0	1.2E-05	5.0E-03
chr14:60698475-60698937	463	2.9	2.4	3.3	↓	-0.9	2.6E-04	5.0E-02
chr14:60725305-60725545	241	1.5	2.0	0.5	↑	1.5	9.8E-05	2.6E-02
chr14:60910444-60911005	562	2.6	3.0	2.0	↑	0.9	4.9E-04	7.5E-02
chr14:60932165-60932862	698	3.6	3.9	3.3	↑	0.6	2.0E-04	4.2E-02
chr14:61545787-61546071	285	2.0	1.3	2.5	↓	-1.2	1.2E-04	2.9E-02
chr14:62480932-62481993	1062	4.9	5.1	4.7	↑	0.4	1.5E-04	3.6E-02
chr14:63368264-63368721	458	2.7	3.1	2.2	↑	0.9	3.6E-04	6.2E-02
chr14:63929346-63930156	811	5.9	5.6	6.2	↓	-0.5	4.0E-04	6.6E-02
chr14:63998999-63999665	667	3.8	4.1	3.4	↑	0.7	6.2E-04	8.5E-02
chr14:64072444-64072747	304	2.1	2.6	1.5	↑	1.0	5.8E-04	8.2E-02
chr14:6413382-6413955	574	3.1	2.6	3.5	↓	-0.9	7.2E-04	9.3E-02
chr14:64268627-64269225	599	3.0	2.5	3.3	↓	-0.8	4.7E-04	7.2E-02
chr14:64398057-64398498	442	2.1	2.5	1.5	↑	1.0	7.7E-04	9.6E-02
chr14:64540039-64540711	673	3.9	3.5	4.2	↓	-0.6	2.0E-04	4.3E-02
chr14:64868343-64868856	514	2.6	3.0	2.1	↑	1.0	2.5E-04	4.9E-02
chr14:65399574-65401331	1758	5.6	5.4	5.7	↓	-0.3	8.1E-04	9.9E-02
chr14:65728036-65728452	417	2.5	1.9	3.0	↓	-1.1	9.0E-05	2.4E-02
chr14:66051598-66053385	1788	5.4	5.2	5.5	↓	-0.3	3.8E-04	6.4E-02
chr14:66469392-66469716	325	1.9	1.1	2.4	↓	-1.3	4.3E-04	6.9E-02
chr14:66729226-66729644	419	2.4	1.9	2.8	↓	-1.0	5.3E-04	7.7E-02
chr14:67760093-67760535	443	2.4	1.8	2.8	↓	-1.1	2.6E-04	5.0E-02
chr14:67781094-67781709	616	3.7	3.3	4.0	↓	-0.7	3.3E-04	5.7E-02
chr14:68136302-68137295	994	4.3	4.0	4.5	↓	-0.5	6.0E-04	8.3E-02
chr14:6842235-6842902	668	5.7	5.5	5.9	↓	-0.5	2.8E-05	1.0E-02
chr14:68670700-68670997	298	2.4	1.6	2.8	↓	-1.2	2.3E-05	8.5E-03
chr14:68684928-68685716	789	3.3	2.8	3.6	↓	-0.8	2.2E-04	4.5E-02
chr14:70338573-70338989	417	2.5	2.0	2.9	↓	-0.9	5.8E-04	8.2E-02
chr14:70837150-70837394	245	1.6	2.1	0.8	↑	1.3	4.3E-04	6.9E-02
chr14:72429543-72430198	656	3.2	3.7	2.7	↑	1.0	1.9E-05	7.3E-03
chr14:7282955-7283843	889	4.4	4.1	4.6	↓	-0.5	7.8E-04	9.7E-02
chr14:73815347-73815575	229	2.3	2.7	1.7	↑	1.0	5.6E-04	8.0E-02
chr14:7417299-7418309	1011	4.8	4.6	5.1	↓	-0.5	7.8E-06	3.6E-03
chr14:77677881-77678307	427	3.8	3.5	4.1	↓	-0.6	7.3E-04	9.4E-02
chr14:78643414-78644066	653	3.6	3.9	3.1	↑	0.8	3.1E-05	1.1E-02
chr14:78696996-78697339	344	3.7	4.1	3.3	↑	0.8	2.2E-05	8.3E-03
chr14:78775350-78776322	973	4.4	4.0	4.7	↓	-0.8	6.5E-07	4.9E-04
chr14:78790015-78790550	536	3.5	3.1	3.8	↓	-0.7	6.1E-04	8.4E-02
chr14:78816073-78817062	990	5.2	5.4	4.9	↑	0.5	3.2E-04	5.6E-02
chr14:78984410-78984573	164	1.8	2.3	1.2	↑	1.1	7.4E-04	9.5E-02
chr14:79166427-79167357	931	5.3	5.5	5.1	↑	0.4	1.3E-04	3.2E-02
chr14:79420149-79420622	474	2.7	2.0	3.1	↓	-1.1	3.2E-05	1.1E-02
chr14:79755187-79755810	624	3.6	3.1	4.1	↓	-1.0	1.4E-08	2.4E-05
chr14:79767156-79767558	403	3.2	2.6	3.6	↓	-0.9	1.2E-05	5.3E-03
chr14:79794589-79795418	830	5.2	5.0	5.4	↓	-0.4	7.5E-05	2.1E-02
chr14:80323525-80324093	569	3.3	2.8	3.6	↓	-0.8	1.9E-04	4.1E-02
chr14:80478642-80479234	593	3.9	3.6	4.2	↓	-0.6	2.6E-04	5.0E-02

chr14:80595096-80596036	941	5.6	5.4	5.8	↓	-0.4	1.6E-04	3.8E-02
chr14:80640251-80640588	338	2.0	1.2	2.5	↓	-1.3	3.1E-05	1.1E-02
chr14:81358610-81358966	357	2.3	2.7	1.7	↑	1.0	7.4E-04	9.5E-02
chr14:82622276-82624172	1897	5.9	6.0	5.7	↑	0.3	3.2E-04	5.7E-02
chr14:83236895-83237923	1029	5.2	4.9	5.4	↓	-0.5	2.1E-04	4.4E-02
chr14:83256490-83256738	249	1.8	1.0	2.3	↓	-1.3	1.6E-04	3.7E-02
chr14:83716365-83716990	626	3.7	4.0	3.3	↑	0.7	6.4E-05	1.9E-02
chr14:83736186-83736454	269	2.5	2.9	1.9	↑	1.0	6.0E-04	8.3E-02
chr14:83877794-83878252	459	2.6	3.0	2.1	↑	0.9	1.4E-04	3.4E-02
chr14:84021860-84022746	887	5.2	5.5	4.9	↑	0.7	2.2E-05	8.4E-03
chr14:84023037-84023436	400	2.1	2.6	1.5	↑	1.1	2.5E-04	4.9E-02
chr14:84381031-84381701	671	5.4	5.1	5.6	↓	-0.5	1.0E-06	7.0E-04
chr14:84552943-84553606	664	3.9	4.2	3.6	↑	0.6	7.0E-04	9.1E-02
chr14:84571651-84575081	3431	6.9	7.1	6.7	↑	0.4	6.7E-06	3.3E-03
chr14:84577233-84579394	2162	5.5	5.7	5.4	↑	0.3	5.3E-04	7.7E-02
chr14:84790214-84790834	621	3.6	3.1	3.9	↓	-0.8	5.0E-05	1.6E-02
chr14:85729878-85730275	398	2.0	2.5	1.4	↑	1.1	6.4E-04	8.6E-02
chr14:86070210-86070618	409	4.1	4.3	3.7	↑	0.6	3.9E-04	6.6E-02
chr14:86235063-86236115	1053	6.2	5.9	6.4	↓	-0.5	1.1E-07	1.1E-04
chr14:86345656-86346217	562	2.6	3.1	1.7	↑	1.4	5.3E-07	4.3E-04
chr14:8696266-8697458	1193	5.3	5.1	5.5	↓	-0.3	5.6E-04	8.0E-02
chr14:87578972-87579696	725	4.1	4.4	3.8	↑	0.6	4.0E-04	6.6E-02
chr14:87587060-87587514	455	2.9	3.3	2.4	↑	1.0	1.0E-04	2.7E-02
chr14:8767328-8767729	402	3.7	3.2	4.0	↓	-0.7	6.6E-04	8.8E-02
chr14:87767228-87767808	581	4.8	4.5	5.1	↓	-0.6	7.3E-06	3.4E-03
chr14:87945735-87946568	834	3.4	3.0	3.8	↓	-0.8	4.8E-05	1.5E-02
chr14:88119697-88120853	1157	4.8	4.6	5.1	↓	-0.4	1.4E-04	3.5E-02
chr14:88132000-88132728	729	3.7	4.0	3.3	↑	0.6	3.9E-04	6.6E-02
chr14:881935-883082	1148	4.2	4.5	3.9	↑	0.6	6.6E-05	1.9E-02
chr14:8821922-8822582	661	4.8	4.6	5.0	↓	-0.4	7.2E-04	9.3E-02
chr14:8958978-8959292	315	2.4	1.8	2.8	↓	-1.0	5.9E-04	8.2E-02
chr14:89863187-89863433	247	2.0	1.2	2.5	↓	-1.3	2.4E-04	4.9E-02
chr14:91280698-91281005	308	2.8	2.3	3.3	↓	-1.0	3.9E-05	1.3E-02
chr14:91705313-91705679	367	2.6	3.0	1.9	↑	1.1	5.6E-04	8.0E-02
chr14:92401104-92401358	255	2.3	1.5	2.8	↓	-1.2	1.5E-05	6.2E-03
chr14:92592593-92593460	868	5.7	5.4	5.9	↓	-0.5	1.1E-10	3.4E-07
chr14:92765004-92765223	220	1.8	1.1	2.3	↓	-1.3	1.6E-04	3.7E-02
chr14:92874340-92874686	347	2.7	2.2	3.1	↓	-0.9	6.0E-04	8.3E-02
chr14:92920549-92920927	379	2.8	2.3	3.1	↓	-0.8	6.7E-04	8.8E-02
chr14:93025417-93026028	612	4.0	3.5	4.3	↓	-0.8	6.4E-04	8.6E-02
chr14:93158717-93159157	441	2.6	2.0	3.0	↓	-1.0	3.0E-04	5.5E-02
chr14:93323021-93323324	304	2.7	2.0	3.2	↓	-1.2	4.8E-06	2.6E-03
chr14:93500545-93501346	802	4.5	4.8	4.2	↑	0.6	1.5E-04	3.6E-02
chr14:93501788-93502518	731	4.6	4.9	4.1	↑	0.8	3.2E-05	1.1E-02
chr14:93516188-93516578	391	2.4	2.8	1.8	↑	1.0	2.5E-04	4.9E-02
chr14:93542240-93542627	388	1.9	2.5	1.0	↑	1.5	1.1E-05	4.8E-03
chr14:93545441-93545930	490	2.6	3.0	2.0	↑	1.0	7.6E-05	2.1E-02

chr14:93560107-93561475	1369	4.9	5.2	4.6	↑	0.7	1.7E-07	1.7E-04
chr14:93586008-93589044	3037	6.0	6.2	5.7	↑	0.5	6.6E-10	1.5E-06
chr14:93818900-93819249	350	3.4	3.7	3.0	↑	0.8	8.9E-05	2.4E-02
chr14:93859859-93860719	861	4.5	4.7	4.2	↑	0.5	2.3E-04	4.7E-02
chr14:93900690-93901841	1152	4.8	5.1	4.5	↑	0.6	1.9E-04	4.2E-02
chr14:93902129-93902875	747	3.9	4.2	3.5	↑	0.6	8.4E-05	2.3E-02
chr14:94046456-94046897	442	2.4	2.8	1.9	↑	0.9	5.1E-04	7.7E-02
chr14:94091685-94092027	343	2.3	1.6	2.8	↓	-1.2	1.6E-05	6.6E-03
chr14:94992801-94993488	688	2.9	2.3	3.2	↓	-0.9	5.3E-04	7.7E-02
chr14:9522401-9523018	618	4.6	4.8	4.2	↑	0.6	5.8E-05	1.7E-02
chr14:95610331-95610836	506	3.0	3.4	2.6	↑	0.8	4.3E-04	6.9E-02
chr14:96182163-96182656	494	3.9	4.5	2.9	↑	1.7	7.5E-14	6.4E-10
chr14:96231126-96232189	1064	3.6	3.9	3.2	↑	0.7	1.9E-04	4.2E-02
chr14:96258819-96259332	514	3.2	3.5	2.7	↑	0.9	4.3E-04	6.9E-02
chr14:96359568-96360512	945	4.8	4.3	5.2	↓	-0.9	3.0E-09	5.9E-06
chr14:97421682-97422289	608	3.6	3.9	3.2	↑	0.7	4.5E-04	7.1E-02
chr14:97430691-97432286	1596	5.5	5.7	5.3	↑	0.4	9.8E-05	2.6E-02
chr14:97811994-97812279	286	2.0	1.4	2.5	↓	-1.1	3.5E-04	6.1E-02
chr14:98572577-98572862	286	2.4	1.7	2.9	↓	-1.2	1.6E-05	6.6E-03
chr14:98590778-98591197	420	2.5	1.8	2.9	↓	-1.1	8.6E-05	2.4E-02
chr14:98597665-98599887	2223	6.0	5.7	6.1	↓	-0.4	5.6E-07	4.3E-04
chr14:98601592-98601846	255	2.2	1.6	2.7	↓	-1.0	3.2E-04	5.6E-02
chr14:98662190-98662875	686	3.6	4.0	3.1	↑	0.9	6.8E-06	3.3E-03
chr14:99764256-99764490	235	1.7	2.2	1.0	↑	1.2	5.2E-04	7.7E-02
chr14:99874697-99875183	487	4.0	3.5	4.3	↓	-0.9	1.6E-05	6.4E-03
chr14:99877019-99878261	1243	4.1	4.5	3.6	↑	1.0	1.3E-06	8.5E-04
chr15:10073710-10074274	565	3.7	3.2	4.1	↓	-1.0	1.5E-07	1.1E-04
chr15:102319165-102319661	497	3.8	3.4	4.0	↓	-0.6	2.8E-04	5.4E-02
chr15:102329736-102330274	539	3.2	2.8	3.5	↓	-0.7	6.2E-04	9.1E-02
chr15:103515052-103515579	528	4.3	4.0	4.5	↓	-0.5	3.1E-04	5.7E-02
chr15:103554249-103555259	1011	3.8	3.5	4.1	↓	-0.7	6.7E-04	9.6E-02
chr15:103705338-103706178	841	4.9	4.4	5.2	↓	-0.8	1.3E-07	9.7E-05
chr15:104105673-104105998	326	2.2	1.3	2.7	↓	-1.4	3.5E-05	1.1E-02
chr15:107094853-107095282	430	3.3	2.8	3.7	↓	-0.8	2.5E-04	5.0E-02
chr15:107152251-107152585	335	2.0	2.5	1.2	↑	1.4	2.4E-05	7.7E-03
chr15:107648053-107648839	787	3.8	3.5	4.1	↓	-0.7	5.1E-04	7.9E-02
chr15:108387405-108387826	422	2.8	3.2	2.2	↑	1.1	2.3E-05	7.7E-03
chr15:109000152-109000467	316	1.9	2.3	1.2	↑	1.2	3.6E-04	6.2E-02
chr15:111113683-111114000	318	1.9	1.2	2.4	↓	-1.2	2.8E-04	5.4E-02
chr15:112092821-112093367	547	3.9	3.5	4.2	↓	-0.7	1.2E-05	4.7E-03
chr15:113149522-113150070	549	4.1	3.6	4.4	↓	-0.8	1.2E-07	8.7E-05
chr15:1133880-1134429	550	3.8	3.5	4.1	↓	-0.7	8.1E-05	2.1E-02
chr15:115184264-115187121	2858	6.2	5.9	6.4	↓	-0.5	4.1E-09	4.5E-06
chr15:117026233-117027307	1075	4.0	3.5	4.3	↓	-0.8	6.0E-05	1.7E-02
chr15:118303363-118303821	459	2.7	1.9	3.2	↓	-1.2	1.2E-06	6.4E-04
chr15:118384220-118387034	2815	6.2	5.7	6.5	↓	-0.8	3.2E-09	3.8E-06
chr15:118387193-118388236	1044	4.7	4.4	5.0	↓	-0.6	3.9E-07	2.5E-04

chr15:118388245-118388620	376	2.7	1.9	3.1	↓	-1.2	1.5E-06	8.0E-04
chr15:118389759-118390032	274	2.0	1.3	2.4	↓	-1.1	5.7E-04	8.5E-02
chr15:118391714-118393839	2126	5.3	5.0	5.6	↓	-0.6	1.1E-05	4.4E-03
chr15:118782096-118782839	744	2.5	3.0	2.0	↑	1.0	1.5E-04	3.5E-02
chr15:118868108-118868704	597	3.4	2.9	3.8	↓	-0.9	2.2E-04	4.6E-02
chr15:121738759-121739423	665	3.3	2.9	3.6	↓	-0.7	4.6E-04	7.5E-02
chr15:122610923-122611792	870	3.8	3.4	4.1	↓	-0.7	2.3E-05	7.7E-03
chr15:122611808-122612210	403	2.6	1.6	3.1	↓	-1.5	2.0E-08	1.9E-05
chr15:122613299-122613919	621	2.8	2.1	3.2	↓	-1.1	2.2E-05	7.6E-03
chr15:122614992-122615351	360	2.4	1.8	2.8	↓	-1.1	1.1E-04	2.7E-02
chr15:122634927-122635480	554	3.2	2.6	3.5	↓	-0.9	1.9E-04	4.1E-02
chr15:123293758-123294173	416	3.3	2.4	3.8	↓	-1.4	2.9E-10	3.8E-07
chr15:123609452-123610160	709	3.1	2.7	3.4	↓	-0.8	6.6E-04	9.5E-02
chr15:124803435-124803987	553	3.1	2.6	3.4	↓	-0.8	4.1E-04	6.9E-02
chr15:126535854-126536104	251	2.1	2.6	1.4	↑	1.2	4.6E-04	7.5E-02
chr15:130466581-130467017	437	3.1	2.6	3.4	↓	-0.9	8.1E-05	2.1E-02
chr15:134237288-134237662	375	2.2	1.5	2.6	↓	-1.1	4.0E-04	6.8E-02
chr15:134610521-134611933	1413	5.3	5.5	5.0	↑	0.6	4.2E-05	1.3E-02
chr15:134615606-134620338	4733	7.6	7.7	7.5	↑	0.2	3.7E-06	1.7E-03
chr15:134679075-134681675	2601	6.6	6.7	6.4	↑	0.3	1.4E-06	7.4E-04
chr15:136028578-136029448	871	3.9	3.5	4.2	↓	-0.6	9.5E-05	2.4E-02
chr15:136404091-136405998	1908	4.8	4.6	5.1	↓	-0.5	1.1E-04	2.7E-02
chr15:136775738-136776178	441	3.4	3.6	3.0	↑	0.7	4.3E-04	7.1E-02
chr15:138579349-138580616	1268	4.9	4.3	5.3	↓	-1.0	2.4E-10	3.4E-07
chr15:138588412-138588899	488	3.0	3.3	2.5	↑	0.8	4.8E-04	7.7E-02
chr15:139152603-139152944	342	3.6	3.2	3.9	↓	-0.8	3.1E-04	5.7E-02
chr15:139407160-139407938	779	4.3	4.0	4.5	↓	-0.5	3.7E-04	6.3E-02
chr15:139804524-139804905	382	3.0	2.5	3.4	↓	-0.9	1.2E-04	2.8E-02
chr15:140342478-140342857	380	2.5	1.8	2.9	↓	-1.0	8.4E-05	2.2E-02
chr15:141655385-141655885	501	2.6	3.0	2.0	↑	1.0	5.8E-04	8.6E-02
chr15:142092776-142093239	464	2.6	2.0	3.0	↓	-1.0	2.2E-04	4.6E-02
chr15:142122819-142123563	745	3.5	3.1	3.9	↓	-0.8	3.3E-04	5.9E-02
chr15:144875902-144876312	411	2.5	3.0	1.9	↑	1.2	1.7E-05	6.5E-03
chr15:145631442-145632848	1407	5.3	4.9	5.6	↓	-0.7	2.7E-08	2.4E-05
chr15:146036871-146037953	1083	5.6	5.4	5.8	↓	-0.4	5.1E-05	1.5E-02
chr15:146527414-146529046	1633	6.0	5.6	6.2	↓	-0.6	6.8E-07	4.0E-04
chr15:147478414-147480885	2472	7.0	7.1	6.8	↑	0.2	5.0E-05	1.5E-02
chr15:148869671-148870522	852	4.4	3.9	4.8	↓	-0.9	1.8E-06	9.0E-04
chr15:149822905-149824803	1899	5.6	5.4	5.8	↓	-0.4	2.6E-04	5.2E-02
chr15:149843013-149850197	7185	7.6	7.5	7.8	↓	-0.3	2.3E-06	1.1E-03
chr15:150420616-150420882	267	1.8	2.3	1.0	↑	1.3	2.1E-04	4.4E-02
chr15:150497843-150499098	1256	4.8	4.5	5.1	↓	-0.6	2.2E-04	4.6E-02
chr15:150537341-150537693	353	2.8	2.3	3.1	↓	-0.8	5.0E-04	7.9E-02
chr15:150777023-150780066	3044	6.8	6.7	6.9	↓	-0.2	6.0E-05	1.7E-02
chr15:151021855-151022416	562	3.3	2.5	3.8	↓	-1.3	7.4E-09	7.7E-06
chr15:151362606-151363648	1043	3.9	4.2	3.6	↑	0.6	5.8E-04	8.7E-02
chr15:151462986-151464294	1309	5.9	5.7	6.0	↓	-0.4	1.1E-04	2.7E-02

chr15:151520165-151521548	1384	4.6	4.4	4.9	↓	-0.5	3.4E-05	1.0E-02
chr15:151941672-151942652	981	4.3	4.5	4.0	↑	0.5	5.2E-04	8.1E-02
chr15:152121112-152122355	1244	6.2	6.1	6.3	↓	-0.3	4.4E-04	7.3E-02
chr15:152577876-152578355	480	2.5	1.9	2.9	↓	-1.0	6.3E-04	9.2E-02
chr15:152584095-152585137	1043	4.5	4.7	4.2	↑	0.5	4.1E-04	6.9E-02
chr15:152796415-152800997	4583	7.7	7.6	7.8	↓	-0.2	2.6E-04	5.2E-02
chr15:153364965-153365562	598	4.7	3.8	5.2	↓	-1.4	5.9E-13	1.1E-09
chr15:153719411-153719817	407	1.9	1.2	2.3	↓	-1.2	4.7E-04	7.6E-02
chr15:153721664-153723794	2131	5.4	5.2	5.6	↓	-0.4	5.0E-04	7.9E-02
chr15:154233948-154236860	2913	6.5	6.3	6.6	↓	-0.3	1.6E-06	8.2E-04
chr15:155205319-155212140	6822	9.0	8.9	9.0	↓	-0.1	4.7E-04	7.5E-02
chr15:155451202-155457529	6328	9.6	9.5	9.6	↓	-0.1	1.7E-05	6.6E-03
chr15:155588123-155591880	3758	7.3	7.2	7.4	↓	-0.2	5.0E-04	7.9E-02
chr15:156827771-156828405	635	3.1	2.3	3.6	↓	-1.3	3.2E-07	2.1E-04
chr15:17796990-17797276	287	2.4	1.8	2.8	↓	-1.0	2.1E-04	4.4E-02
chr15:1814859-1815206	348	2.1	1.4	2.5	↓	-1.2	1.9E-04	4.1E-02
chr15:18767141-18767578	438	2.6	2.0	3.1	↓	-1.1	7.1E-05	1.9E-02
chr15:20511318-20512204	887	3.7	3.2	4.1	↓	-1.0	2.4E-06	1.1E-03
chr15:20741638-20742100	463	3.8	2.8	4.4	↓	-1.6	2.1E-12	3.5E-09
chr15:20742248-20742888	641	3.4	2.6	3.8	↓	-1.2	9.6E-09	9.4E-06
chr15:20784550-20785164	615	3.2	2.8	3.5	↓	-0.7	5.5E-04	8.4E-02
chr15:20834415-20835366	952	4.3	3.9	4.6	↓	-0.6	2.1E-05	7.5E-03
chr15:21048949-21049364	416	2.3	1.6	2.7	↓	-1.1	1.5E-04	3.5E-02
chr15:22098868-22099776	909	2.9	3.5	1.9	↑	1.6	4.2E-07	2.6E-04
chr15:22845724-22846136	413	2.6	2.1	3.0	↓	-0.9	5.7E-04	8.6E-02
chr15:2486040-2486386	347	2.5	2.9	1.9	↑	1.0	4.0E-04	6.8E-02
chr15:25329562-25330039	478	3.0	3.4	2.6	↑	0.8	5.5E-04	8.4E-02
chr15:2684875-2686095	1221	4.4	4.7	4.1	↑	0.6	5.2E-05	1.5E-02
chr15:27035646-27036042	397	2.7	2.2	3.1	↓	-0.9	3.5E-04	6.1E-02
chr15:27036475-27036841	367	2.6	2.0	3.0	↓	-1.0	9.9E-05	2.5E-02
chr15:27312986-27313446	461	2.9	2.3	3.3	↓	-1.0	2.3E-05	7.7E-03
chr15:28279304-28279940	637	3.0	2.5	3.3	↓	-0.8	5.5E-04	8.4E-02
chr15:31213679-31214042	364	2.1	1.5	2.6	↓	-1.1	3.3E-04	5.9E-02
chr15:32034568-32035163	596	3.3	2.7	3.7	↓	-1.0	1.3E-04	2.9E-02
chr15:32035862-32037198	1337	4.9	4.5	5.2	↓	-0.6	3.2E-07	2.1E-04
chr15:32038242-32040721	2480	6.4	6.3	6.6	↓	-0.3	5.2E-04	8.1E-02
chr15:32041729-32042131	403	1.9	1.1	2.4	↓	-1.3	6.3E-05	1.7E-02
chr15:34399873-34400556	684	4.0	3.6	4.3	↓	-0.6	1.2E-04	2.9E-02
chr15:35610448-35610900	453	2.7	2.1	3.1	↓	-1.0	1.6E-04	3.7E-02
chr15:36892764-36895570	2807	6.6	6.4	6.7	↓	-0.3	5.9E-05	1.7E-02
chr15:37060197-37060855	659	4.4	3.9	4.7	↓	-0.9	1.1E-05	4.4E-03
chr15:37091745-37092801	1057	4.5	4.2	4.8	↓	-0.7	2.6E-04	5.2E-02
chr15:38047782-38048770	989	3.8	3.3	4.1	↓	-0.9	2.2E-05	7.6E-03
chr15:38049046-38051268	2223	7.1	7.3	7.0	↑	0.3	2.4E-05	7.7E-03
chr15:39593265-39593692	428	2.6	2.1	3.0	↓	-0.9	1.9E-04	4.1E-02
chr15:42406741-42407263	523	5.1	4.8	5.3	↓	-0.6	2.2E-05	7.6E-03
chr15:42649637-42651685	2049	5.1	4.8	5.3	↓	-0.5	7.1E-05	1.9E-02

chr15:43355632-43356149	518	3.0	3.4	2.4	↑	1.0	7.4E-05	2.0E-02
chr15:44061535-44062580	1046	4.5	4.8	4.2	↑	0.6	6.0E-04	8.9E-02
chr15:44521651-44522033	383	2.1	2.7	1.2	↑	1.5	3.1E-06	1.4E-03
chr15:489556-489870	315	2.5	1.8	3.0	↓	-1.1	6.9E-05	1.9E-02
chr15:49844932-49846060	1129	4.7	4.3	5.0	↓	-0.7	4.7E-06	2.1E-03
chr15:50389035-50389396	362	2.6	3.0	1.9	↑	1.1	2.2E-05	7.6E-03
chr15:50389519-50389929	411	2.3	2.8	1.5	↑	1.3	6.5E-06	2.8E-03
chr15:50518343-50519142	800	3.0	3.5	2.2	↑	1.3	5.9E-08	4.8E-05
chr15:50519747-50521209	1463	5.0	5.4	4.4	↑	1.1	1.7E-08	1.6E-05
chr15:50591952-50592913	962	3.7	3.3	4.0	↓	-0.7	1.9E-04	4.1E-02
chr15:51322055-51322638	584	2.8	3.1	2.3	↑	0.8	6.2E-04	9.1E-02
chr15:51986323-51987027	705	2.8	3.2	2.4	↑	0.8	6.7E-04	9.6E-02
chr15:52679046-52679306	261	2.7	2.2	3.1	↓	-0.9	6.4E-04	9.3E-02
chr15:52915254-52915760	507	2.9	3.3	2.4	↑	0.9	3.5E-04	6.1E-02
chr15:53329672-53330640	969	3.3	2.8	3.6	↓	-0.9	9.5E-05	2.4E-02
chr15:53584078-53584818	741	3.5	2.8	4.0	↓	-1.2	1.7E-10	2.5E-07
chr15:54616661-54617776	1116	3.8	3.1	4.3	↓	-1.2	3.8E-08	3.2E-05
chr15:54618694-54619139	446	2.3	1.4	2.8	↓	-1.4	6.9E-07	4.0E-04
chr15:54621207-54621741	535	2.7	2.1	3.1	↓	-0.9	2.6E-04	5.2E-02
chr15:54626608-54627139	532	2.8	1.1	3.6	↓	-2.5	5.4E-19	2.1E-15
chr15:54630719-54632494	1776	5.1	4.7	5.4	↓	-0.7	2.4E-07	1.7E-04
chr15:54633339-54638033	4695	7.0	6.0	7.5	↓	-1.5	1.7E-26	1.3E-22
chr15:54642272-54642852	581	2.7	1.2	3.4	↓	-2.2	5.4E-15	1.2E-11
chr15:54672443-54672927	485	2.7	2.0	3.2	↓	-1.3	2.3E-06	1.1E-03
chr15:54855472-54856568	1097	4.3	4.0	4.6	↓	-0.6	3.4E-04	6.1E-02
chr15:54910903-54912239	1337	6.3	5.9	6.5	↓	-0.6	3.2E-08	2.8E-05
chr15:55482454-55483096	643	2.7	2.0	3.2	↓	-1.2	9.2E-07	5.2E-04
chr15:55736223-55736948	726	3.7	3.3	3.9	↓	-0.6	3.1E-04	5.7E-02
chr15:55809415-55811991	2577	6.9	6.6	7.1	↓	-0.5	1.1E-09	1.4E-06
chr15:55814330-55816217	1888	5.5	5.6	5.3	↑	0.3	1.3E-04	2.9E-02
chr15:56055183-56055795	613	4.1	3.8	4.4	↓	-0.6	3.1E-05	9.8E-03
chr15:56193050-56193691	642	4.0	3.6	4.3	↓	-0.7	2.1E-05	7.5E-03
chr15:56228137-56228945	809	3.9	3.5	4.2	↓	-0.7	2.5E-04	5.0E-02
chr15:57706349-57707218	870	3.9	3.5	4.2	↓	-0.6	2.9E-04	5.6E-02
chr15:59415818-59416306	489	4.6	4.1	4.9	↓	-0.8	5.2E-08	4.3E-05
chr15:60012450-60013003	554	3.5	2.8	3.9	↓	-1.1	2.7E-07	1.9E-04
chr15:62521495-62522212	718	3.6	3.9	3.2	↑	0.7	8.6E-05	2.2E-02
chr15:6515198-6517217	2020	5.4	5.2	5.6	↓	-0.4	1.8E-04	4.0E-02
chr15:65446418-65448698	2281	6.0	6.2	5.7	↑	0.4	3.6E-04	6.2E-02
chr15:66896131-66896848	718	3.9	3.6	4.2	↓	-0.6	4.0E-04	6.8E-02
chr15:66900954-66901225	272	1.9	1.0	2.5	↓	-1.4	2.0E-05	7.5E-03
chr15:66904524-66904856	333	2.2	2.6	1.5	↑	1.1	3.5E-04	6.1E-02
chr15:7072675-7073122	448	2.9	2.2	3.3	↓	-1.1	1.6E-06	8.2E-04
chr15:71693402-71694355	954	3.6	3.0	4.0	↓	-1.0	1.0E-05	4.4E-03
chr15:71694382-71695366	985	5.1	4.5	5.5	↓	-1.1	3.0E-21	1.3E-17
chr15:71695447-71696214	768	3.8	3.2	4.1	↓	-0.9	1.7E-05	6.6E-03
chr15:71698767-71700483	1717	4.6	4.0	5.0	↓	-1.0	1.2E-12	2.1E-09

chr15:71700879-71701753	875	4.5	3.7	5.0	↓	-1.3	3.9E-11	5.9E-08
chr15:71749231-71750632	1402	5.5	5.2	5.7	↓	-0.5	2.1E-04	4.4E-02
chr15:71769767-71770284	518	3.6	2.9	4.1	↓	-1.2	1.8E-05	6.6E-03
chr15:71784865-71785391	527	3.3	2.9	3.7	↓	-0.8	2.2E-04	4.6E-02
chr15:71820181-71820678	498	3.5	3.1	3.9	↓	-0.8	3.2E-05	9.9E-03
chr15:71821354-71821783	430	2.0	0.9	2.6	↓	-1.7	5.7E-07	3.4E-04
chr15:71825675-71826477	803	3.7	3.3	4.0	↓	-0.7	1.6E-04	3.6E-02
chr15:73052478-73053090	613	3.6	3.9	3.3	↑	0.6	7.0E-04	9.9E-02
chr15:73953666-73954209	544	3.3	3.7	2.9	↑	0.7	6.8E-04	9.7E-02
chr15:74195097-74195593	497	3.8	3.5	4.1	↓	-0.6	1.6E-04	3.6E-02
chr15:74623373-74624007	635	3.3	3.7	2.8	↑	0.8	6.8E-05	1.9E-02
chr15:79747502-79748063	562	2.9	2.2	3.4	↓	-1.2	6.8E-08	5.3E-05
chr15:84711586-84712572	987	4.8	4.5	5.0	↓	-0.5	4.4E-04	7.3E-02
chr15:86393336-86394106	771	3.3	2.7	3.8	↓	-1.0	2.4E-07	1.7E-04
chr15:86678115-86678644	530	3.0	2.4	3.4	↓	-1.0	1.4E-05	5.4E-03
chr15:86719245-86719586	342	2.6	2.0	3.1	↓	-1.0	3.2E-04	5.8E-02
chr15:88997841-88998921	1081	4.1	3.8	4.4	↓	-0.6	2.7E-05	8.7E-03
chr15:89002932-89003386	455	3.4	3.0	3.8	↓	-0.7	1.2E-04	2.9E-02
chr15:89789852-89790292	441	3.2	2.6	3.5	↓	-0.9	1.0E-04	2.5E-02
chr15:89924648-89925092	445	2.7	2.1	3.1	↓	-0.9	3.4E-04	6.1E-02
chr15:90022065-90023919	1855	5.4	4.9	5.8	↓	-1.0	4.1E-09	4.5E-06
chr15:90025530-90026749	1220	4.6	4.0	5.0	↓	-1.0	1.5E-06	7.8E-04
chr15:90028355-90028893	539	3.5	2.9	3.9	↓	-0.9	2.4E-05	7.7E-03
chr15:91356348-91356735	388	2.4	1.5	2.9	↓	-1.5	1.8E-06	9.0E-04
chr15:91369015-91369433	419	2.6	2.0	3.0	↓	-0.9	5.4E-04	8.3E-02
chr15:91377308-91378073	766	3.7	2.9	4.2	↓	-1.3	3.5E-10	4.5E-07
chr15:91392209-91393390	1182	4.1	2.8	4.8	↓	-1.9	3.3E-26	1.7E-22
chr15:91393905-91394559	655	3.3	2.6	3.8	↓	-1.2	8.6E-09	8.7E-06
chr15:91396125-91397757	1633	5.0	3.9	5.7	↓	-1.7	1.5E-14	3.1E-11
chr15:91402925-91403280	356	2.3	1.7	2.7	↓	-1.0	3.1E-04	5.7E-02
chr15:91407889-91408912	1024	3.9	2.6	4.5	↓	-1.9	1.7E-16	5.0E-13
chr15:91415665-91417285	1621	5.7	4.4	6.4	↓	-2.1	1.7E-32	2.6E-28
chr15:91418147-91418787	641	3.1	2.6	3.5	↓	-0.9	2.8E-05	8.8E-03
chr15:91419540-91421853	2314	5.6	4.1	6.3	↓	-2.2	6.7E-28	6.9E-24
chr15:91423044-91423706	663	3.8	2.9	4.3	↓	-1.4	3.3E-11	5.3E-08
chr15:91425236-91427259	2024	5.0	3.7	5.7	↓	-2.0	1.5E-36	4.4E-32
chr15:91427408-91428894	1487	5.2	4.0	5.9	↓	-1.8	3.7E-16	9.5E-13
chr15:91428908-91430392	1485	5.2	4.0	5.8	↓	-1.9	3.3E-26	1.7E-22
chr15:91430932-91432101	1170	4.7	3.6	5.3	↓	-1.7	1.8E-16	5.0E-13
chr15:91433788-91434591	804	4.4	3.4	4.9	↓	-1.5	4.5E-18	1.5E-14
chr15:91439147-91441300	2154	5.6	5.2	5.9	↓	-0.6	5.4E-06	2.3E-03
chr15:91444515-91445236	722	3.8	2.7	4.4	↓	-1.6	8.9E-16	2.1E-12
chr15:91447031-91447425	395	1.8	0.8	2.4	↓	-1.6	3.8E-06	1.7E-03
chr15:91456171-91456765	595	3.3	2.6	3.7	↓	-1.2	2.3E-05	7.7E-03
chr15:91571052-91571383	332	2.8	2.3	3.1	↓	-0.8	3.7E-04	6.4E-02
chr15:91743710-91744492	783	3.7	3.2	4.0	↓	-0.8	1.6E-05	6.1E-03
chr15:91874096-91874747	652	3.2	2.7	3.5	↓	-0.8	2.0E-04	4.4E-02

chr15:91889390-91890127	738	3.7	3.3	4.0	↓	-0.7	5.3E-05	1.5E-02
chr15:92013455-92014169	715	4.2	3.8	4.5	↓	-0.7	2.8E-04	5.4E-02
chr15:93046721-93047005	285	2.5	1.9	3.0	↓	-1.1	2.4E-04	4.9E-02
chr15:93413217-93413731	515	3.6	4.0	3.2	↑	0.8	3.1E-04	5.7E-02
chr15:96200803-96201390	588	3.5	2.9	3.9	↓	-1.0	4.3E-07	2.6E-04
chr15:96836677-96837173	497	3.4	2.9	3.7	↓	-0.8	2.9E-04	5.5E-02
chr16:18623958-18624295	338	2.0	2.4	1.4	↑	1.1	8.2E-04	7.5E-02
chr16:18705558-18705870	313	2.1	2.5	1.3	↑	1.2	1.3E-04	2.1E-02
chr16:18764431-18764668	238	1.8	2.2	1.1	↑	1.1	1.1E-03	8.6E-02
chr16:18780447-18781573	1127	5.5	5.3	5.7	↓	-0.4	9.4E-04	8.1E-02
chr16:18796629-18797271	643	4.2	4.4	3.9	↑	0.5	5.7E-04	6.1E-02
chr16:18809179-18811284	2106	6.0	6.1	5.9	↑	0.3	8.5E-04	7.6E-02
chr16:18869907-18870340	434	2.5	1.8	3.0	↓	-1.2	2.7E-05	5.8E-03
chr16:18896302-18897687	1386	5.4	5.1	5.6	↓	-0.5	1.9E-06	6.1E-04
chr16:20163153-20163801	649	3.2	2.7	3.6	↓	-0.9	1.2E-05	3.0E-03
chr16:20521192-20521510	319	2.7	2.2	3.1	↓	-1.0	6.5E-04	6.5E-02
chr16:20656854-20657199	346	2.4	1.7	2.9	↓	-1.1	5.3E-05	1.0E-02
chr16:21031067-21031470	404	2.1	2.5	1.4	↑	1.1	2.5E-04	3.5E-02
chr16:21065798-21066156	359	3.0	2.5	3.3	↓	-0.8	6.4E-04	6.4E-02
chr16:21474474-21474959	486	3.3	2.9	3.6	↓	-0.7	3.5E-04	4.3E-02
chr16:21614737-21615336	600	2.8	3.2	2.3	↑	0.8	4.5E-04	5.2E-02
chr16:21794170-21794758	589	3.4	3.0	3.7	↓	-0.7	8.8E-04	7.8E-02
chr16:23671816-23672129	314	1.9	2.4	1.1	↑	1.3	2.7E-04	3.7E-02
chr16:23697789-23698167	379	2.6	2.0	3.0	↓	-1.0	3.1E-04	3.9E-02
chr16:24664114-24664603	490	2.6	2.0	3.0	↓	-1.1	3.3E-05	6.9E-03
chr16:24680563-24681647	1085	4.8	4.5	5.1	↓	-0.7	6.1E-05	1.2E-02
chr16:25057012-25058381	1370	5.5	5.7	5.3	↑	0.4	8.2E-04	7.5E-02
chr16:25302648-25303287	640	3.7	3.3	4.0	↓	-0.7	4.2E-04	4.9E-02
chr16:25509735-25510041	307	2.7	2.2	3.1	↓	-0.9	8.8E-04	7.8E-02
chr16:26052538-26053219	682	3.6	3.0	4.0	↓	-1.0	2.7E-07	1.3E-04
chr16:26409243-26409819	577	3.2	2.2	3.8	↓	-1.6	1.4E-09	1.1E-06
chr16:26446628-26446900	273	2.6	2.0	3.1	↓	-1.0	8.9E-05	1.6E-02
chr16:26463062-26463630	569	3.3	2.9	3.7	↓	-0.8	1.2E-04	2.0E-02
chr16:26489578-26490068	491	2.6	2.0	3.0	↓	-1.0	2.3E-04	3.3E-02
chr16:26504758-26505224	467	3.7	3.0	4.2	↓	-1.1	3.5E-11	4.1E-08
chr16:26512261-26513810	1550	4.4	4.1	4.7	↓	-0.6	9.5E-05	1.7E-02
chr16:26516894-26517180	287	2.5	1.8	3.0	↓	-1.2	1.9E-05	4.6E-03
chr16:26517523-26517998	476	2.6	1.7	3.1	↓	-1.4	7.9E-08	4.5E-05
chr16:26533370-26533830	461	2.8	2.3	3.1	↓	-0.8	6.0E-04	6.3E-02
chr16:26540507-26540833	327	3.0	2.4	3.5	↓	-1.1	2.5E-05	5.5E-03
chr16:26652961-26653587	627	5.2	4.9	5.5	↓	-0.6	1.8E-07	9.3E-05
chr16:26654310-26654678	369	2.1	1.4	2.6	↓	-1.2	2.6E-04	3.6E-02
chr16:26659759-26660016	258	2.2	1.4	2.7	↓	-1.3	7.5E-06	2.0E-03
chr16:26697041-26697614	574	3.6	2.9	4.1	↓	-1.2	1.4E-08	9.9E-06
chr16:26699543-26700137	595	4.0	3.7	4.3	↓	-0.6	6.8E-04	6.7E-02
chr16:26740330-26741057	728	3.3	2.9	3.6	↓	-0.7	1.1E-03	8.6E-02
chr16:26744463-26745153	691	2.8	2.4	3.1	↓	-0.8	1.1E-03	8.8E-02

chr16:28728797-28729401	605	2.4	2.8	1.8	↑	1.0	1.4E-04	2.2E-02
chr16:29283827-29284909	1083	3.8	3.2	4.2	↓	-1.0	5.0E-07	2.1E-04
chr16:29289461-29290205	745	3.9	3.6	4.2	↓	-0.7	7.4E-04	7.1E-02
chr16:29518279-29518986	708	3.6	3.2	3.9	↓	-0.7	7.3E-04	7.1E-02
chr16:30315482-30316298	817	3.8	3.4	4.2	↓	-0.8	3.9E-05	8.1E-03
chr16:30936401-30937237	837	3.9	3.4	4.3	↓	-0.9	1.7E-06	5.7E-04
chr16:31729858-31730339	482	2.4	2.8	1.8	↑	0.9	1.3E-03	9.9E-02
chr16:32150526-32150947	422	2.7	3.3	1.8	↑	1.5	5.7E-08	3.4E-05
chr16:32542237-32542746	510	2.3	3.0	0.9	↑	2.1	9.3E-11	9.3E-08
chr16:32546654-32547462	809	4.1	4.8	2.5	↑	2.3	2.6E-35	5.6E-31
chr16:32552406-32553611	1206	4.9	5.5	3.9	↑	1.6	6.5E-25	4.6E-21
chr16:32563250-32563601	352	2.8	3.5	1.6	↑	1.9	2.1E-11	2.8E-08
chr16:32563879-32565177	1299	3.4	4.0	2.4	↑	1.7	1.0E-13	2.1E-10
chr16:33130186-33130541	356	2.5	1.8	3.0	↓	-1.1	1.1E-04	1.9E-02
chr16:33836186-33836546	361	2.7	2.2	3.1	↓	-0.9	4.5E-04	5.2E-02
chr16:34224396-34225424	1029	4.3	4.5	4.0	↑	0.5	1.0E-03	8.4E-02
chr16:34623974-34624585	612	4.0	4.3	3.6	↑	0.7	6.3E-04	6.4E-02
chr16:35441935-35442287	353	2.1	1.4	2.6	↓	-1.2	4.6E-05	9.1E-03
chr16:36040135-36040499	365	3.4	3.0	3.8	↓	-0.8	1.8E-05	4.4E-03
chr16:36433725-36434634	910	4.8	4.3	5.1	↓	-0.8	2.7E-08	1.7E-05
chr16:36509500-36510142	643	3.4	3.7	3.0	↑	0.6	1.2E-03	9.8E-02
chr16:36780579-36787741	7163	16.9	16.8	16.9	↓	-0.1	2.6E-06	8.3E-04
chr16:37210003-37210407	405	3.0	1.9	3.6	↓	-1.7	5.2E-13	9.2E-10
chr16:37210892-37211380	489	3.6	3.1	3.9	↓	-0.8	2.7E-06	8.4E-04
chr16:37213769-37214165	397	2.2	1.5	2.6	↓	-1.1	3.9E-04	4.6E-02
chr16:37223774-37225177	1404	5.2	4.5	5.7	↓	-1.2	2.6E-18	1.4E-14
chr16:37225320-37226135	816	5.0	4.1	5.5	↓	-1.4	1.4E-12	2.3E-09
chr16:37227239-37229096	1858	5.1	4.0	5.7	↓	-1.7	7.8E-29	8.3E-25
chr16:37229558-37230158	601	3.4	1.8	4.2	↓	-2.4	1.1E-17	4.8E-14
chr16:37230202-37230374	173	1.4	0.5	1.9	↓	-1.4	2.6E-04	3.6E-02
chr16:37230505-37230915	411	3.5	2.7	4.0	↓	-1.2	3.4E-10	3.3E-07
chr16:37232188-37232762	575	2.4	1.6	2.9	↓	-1.4	8.3E-07	3.1E-04
chr16:37232790-37234182	1393	4.1	3.0	4.7	↓	-1.7	1.6E-17	5.6E-14
chr16:37234208-37236731	2524	6.2	5.9	6.4	↓	-0.5	4.0E-06	1.2E-03
chr16:37237209-37238021	813	4.1	3.7	4.4	↓	-0.6	4.7E-04	5.3E-02
chr16:37239092-37239388	297	3.2	2.7	3.5	↓	-0.8	6.8E-04	6.7E-02
chr16:37264566-37265238	673	3.9	3.4	4.3	↓	-0.9	1.4E-06	4.9E-04
chr16:37266127-37266738	612	4.2	3.9	4.5	↓	-0.5	2.6E-04	3.6E-02
chr16:37436012-37436675	664	3.4	2.8	3.8	↓	-1.1	2.2E-05	5.2E-03
chr16:37756640-37757158	519	2.5	1.9	2.9	↓	-1.1	1.3E-04	2.1E-02
chr16:37771748-37772394	647	3.7	3.2	4.1	↓	-0.9	8.1E-07	3.1E-04
chr16:37773682-37774411	730	3.6	3.1	4.0	↓	-0.9	8.9E-07	3.2E-04
chr16:37784419-37784658	240	2.0	1.3	2.5	↓	-1.2	2.4E-04	3.4E-02
chr16:37916798-37917190	393	3.5	2.9	3.9	↓	-1.0	4.4E-06	1.3E-03
chr16:37920016-37921031	1016	4.1	3.8	4.3	↓	-0.5	2.5E-04	3.5E-02
chr16:37921327-37921876	550	3.4	3.0	3.7	↓	-0.8	1.2E-04	2.0E-02
chr16:38056947-38058027	1081	5.0	4.7	5.2	↓	-0.6	5.0E-07	2.1E-04

chr16:38111332-38111745	414	2.5	1.9	2.9	↓	-1.0	6.1E-04	6.3E-02
chr16:38747251-38748020	770	4.5	3.7	5.0	↓	-1.3	2.2E-11	2.8E-08
chr16:38807362-38808121	760	4.5	4.3	4.7	↓	-0.4	8.0E-04	7.5E-02
chr16:39017460-39017711	252	2.0	1.3	2.4	↓	-1.2	3.7E-04	4.5E-02
chr16:39018559-39019431	873	5.2	4.8	5.5	↓	-0.7	2.0E-07	1.0E-04
chr16:39081210-39081938	729	4.5	4.3	4.7	↓	-0.4	9.6E-04	8.1E-02
chr16:39209954-39210486	533	2.4	1.8	2.9	↓	-1.1	1.9E-04	2.7E-02
chr16:39212903-39213407	505	2.5	1.7	3.0	↓	-1.3	2.9E-06	8.7E-04
chr16:39463521-39463732	212	1.7	2.2	1.0	↑	1.2	5.6E-04	5.9E-02
chr16:39661069-39661715	647	3.3	2.9	3.7	↓	-0.8	2.3E-05	5.2E-03
chr16:39661749-39662273	525	3.4	2.9	3.7	↓	-0.8	2.8E-04	3.7E-02
chr16:39663979-39665199	1221	4.2	3.7	4.5	↓	-0.8	3.2E-07	1.5E-04
chr16:39713017-39713655	639	3.2	2.7	3.6	↓	-0.8	2.0E-04	3.0E-02
chr16:39714368-39716754	2387	6.0	5.7	6.2	↓	-0.5	4.5E-10	4.0E-07
chr16:39723346-39724261	916	3.9	3.4	4.2	↓	-0.8	1.5E-04	2.3E-02
chr16:39726249-39726774	526	3.5	3.1	3.8	↓	-0.7	9.0E-04	7.8E-02
chr16:39730096-39730804	709	4.4	4.1	4.7	↓	-0.6	8.1E-04	7.5E-02
chr16:39758708-39758981	274	1.7	1.0	2.2	↓	-1.1	1.0E-03	8.5E-02
chr16:39763513-39763914	402	2.8	2.3	3.2	↓	-0.9	3.0E-04	3.9E-02
chr16:40961921-40962194	274	3.0	2.5	3.3	↓	-0.9	7.5E-05	1.4E-02
chr16:41104902-41105375	474	3.3	2.8	3.6	↓	-0.8	1.6E-04	2.4E-02
chr16:41195234-41195810	577	2.2	1.7	2.6	↓	-1.0	5.8E-04	6.1E-02
chr16:41293229-41293598	370	3.8	3.5	4.0	↓	-0.6	5.1E-04	5.6E-02
chr16:41293639-41294290	652	3.4	3.0	3.7	↓	-0.7	5.2E-04	5.7E-02
chr16:41317904-41318425	522	2.5	1.9	2.9	↓	-0.9	8.1E-04	7.5E-02
chr16:4150427-4151614	1188	4.3	4.0	4.6	↓	-0.6	2.2E-05	5.2E-03
chr16:42011484-42011983	500	2.6	3.0	2.0	↑	0.9	4.1E-04	4.9E-02
chr16:42149252-42149604	353	2.2	1.7	2.7	↓	-1.0	6.4E-04	6.4E-02
chr16:42157730-42158157	428	3.2	2.3	3.8	↓	-1.4	2.5E-07	1.2E-04
chr16:42188997-42189730	734	3.7	3.3	4.1	↓	-0.7	1.4E-04	2.1E-02
chr16:42518079-42518504	426	1.9	2.5	0.9	↑	1.6	4.3E-06	1.3E-03
chr16:4294704-4296274	1571	5.3	5.6	5.0	↑	0.6	1.9E-05	4.6E-03
chr16:43197546-43198281	736	3.1	2.6	3.5	↓	-0.9	1.2E-04	2.0E-02
chr16:43201154-43201547	394	2.5	1.5	3.1	↓	-1.6	1.2E-08	8.4E-06
chr16:43201891-43202493	603	2.8	2.1	3.2	↓	-1.1	2.6E-06	8.3E-04
chr16:43202541-43204485	1945	5.3	4.7	5.7	↓	-1.0	6.9E-10	5.8E-07
chr16:43207818-43208066	249	1.8	1.1	2.3	↓	-1.2	3.3E-04	4.1E-02
chr16:4337716-4338412	697	3.3	2.8	3.7	↓	-0.9	1.0E-04	1.8E-02
chr16:43495660-43496292	633	2.9	3.3	2.4	↑	0.9	2.4E-04	3.4E-02
chr16:44384390-44384692	303	2.6	3.0	2.1	↑	0.9	3.2E-04	4.0E-02
chr16:4535707-4536968	1262	5.2	5.0	5.4	↓	-0.4	8.7E-04	7.7E-02
chr16:45940415-45940797	383	3.1	2.5	3.5	↓	-1.0	2.8E-05	6.1E-03
chr16:48243817-48244128	312	2.8	2.3	3.2	↓	-0.9	2.0E-04	3.0E-02
chr16:48458461-48458803	343	2.9	2.2	3.3	↓	-1.1	1.1E-04	1.8E-02
chr16:4874367-4874743	377	2.2	2.7	1.6	↑	1.1	1.7E-04	2.6E-02
chr16:48968155-48968530	376	2.3	1.5	2.8	↓	-1.4	5.2E-06	1.5E-03
chr16:48974903-48975653	751	4.2	3.9	4.4	↓	-0.5	1.1E-03	8.7E-02

chr16:49894085-49894581	497	4.4	4.6	4.1	↑	0.5	1.1E-04	1.9E-02
chr16:49897747-49898392	646	3.7	4.0	3.3	↑	0.7	3.1E-04	3.9E-02
chr16:49986239-49987633	1395	4.0	4.3	3.7	↑	0.6	4.7E-04	5.3E-02
chr16:50449485-50450063	579	2.5	2.8	1.9	↑	0.9	4.2E-04	4.9E-02
chr16:50483973-50484376	404	2.5	2.0	2.9	↓	-0.9	9.4E-04	8.1E-02
chr16:50729427-50729932	506	3.8	3.4	4.1	↓	-0.6	7.3E-04	7.1E-02
chr16:51600864-51601417	554	3.3	2.8	3.7	↓	-0.9	5.9E-06	1.6E-03
chr16:52014208-52015052	845	4.3	4.0	4.5	↓	-0.5	7.5E-04	7.2E-02
chr16:52434895-52435853	959	3.8	4.1	3.5	↑	0.6	9.5E-04	8.1E-02
chr16:5353282-5353776	495	2.7	3.1	2.2	↑	0.8	6.3E-04	6.4E-02
chr16:54063348-54063629	282	2.9	2.3	3.2	↓	-0.9	5.9E-04	6.2E-02
chr16:54119862-54120591	730	3.0	2.6	3.3	↓	-0.7	1.3E-03	9.9E-02
chr16:54359674-54361283	1610	5.3	5.0	5.5	↓	-0.5	6.0E-08	3.5E-05
chr16:5436756-5437341	586	3.2	2.7	3.6	↓	-0.9	7.1E-06	1.9E-03
chr16:54478993-54479446	454	3.2	2.6	3.5	↓	-0.9	4.0E-05	8.1E-03
chr16:54522095-54523668	1574	5.8	5.6	6.0	↓	-0.3	3.1E-04	3.9E-02
chr16:55018983-55019830	848	4.5	4.2	4.8	↓	-0.7	1.3E-04	2.1E-02
chr16:55165578-55166314	737	4.9	4.7	5.1	↓	-0.4	1.3E-03	9.9E-02
chr16:55194821-55195695	875	4.0	3.7	4.2	↓	-0.5	8.5E-04	7.6E-02
chr16:55308501-55309214	714	3.2	3.5	2.8	↑	0.7	5.6E-04	5.9E-02
chr16:55639055-55639509	455	3.2	2.8	3.6	↓	-0.8	3.6E-04	4.3E-02
chr16:56012252-56013081	830	3.7	4.0	3.4	↑	0.6	8.2E-04	7.5E-02
chr16:56015390-56016052	663	3.3	3.6	2.9	↑	0.7	5.0E-04	5.6E-02
chr16:56017396-56020208	2813	6.4	6.6	6.1	↑	0.4	3.8E-07	1.7E-04
chr16:56027022-56028284	1263	5.3	5.5	5.1	↑	0.4	2.3E-05	5.3E-03
chr16:56252101-56252571	471	2.2	1.6	2.6	↓	-1.0	9.3E-04	8.1E-02
chr16:56819936-56823336	3401	7.1	7.3	6.8	↑	0.4	2.9E-04	3.7E-02
chr16:56942429-56944395	1967	5.7	5.8	5.5	↑	0.3	8.8E-04	7.8E-02
chr16:57730558-57731510	953	4.1	3.8	4.3	↓	-0.6	7.8E-04	7.4E-02
chr16:57748561-57749043	483	3.2	2.8	3.5	↓	-0.7	7.4E-04	7.1E-02
chr16:57763640-57764239	600	2.9	2.4	3.2	↓	-0.8	1.2E-03	9.6E-02
chr16:57770092-57771104	1013	5.6	5.7	5.4	↑	0.4	5.3E-05	1.0E-02
chr16:58029185-58029900	716	2.8	3.3	2.2	↑	1.1	2.6E-05	5.7E-03
chr16:58220862-58222014	1153	4.2	3.9	4.5	↓	-0.6	9.6E-04	8.1E-02
chr16:58344600-58344972	373	2.2	2.7	1.6	↑	1.1	2.7E-04	3.6E-02
chr16:59008963-59009590	628	3.7	4.0	3.4	↑	0.7	4.7E-04	5.3E-02
chr16:59233296-59235454	2159	6.2	6.1	6.3	↓	-0.2	7.6E-04	7.2E-02
chr16:59276010-59276615	606	2.9	2.4	3.3	↓	-0.9	3.9E-05	8.1E-03
chr16:59423869-59427454	3586	7.2	7.1	7.3	↓	-0.2	4.2E-04	4.9E-02
chr16:59428865-59429377	513	3.6	3.1	4.0	↓	-0.9	6.8E-07	2.7E-04
chr16:59440444-59441565	1122	5.1	4.8	5.4	↓	-0.6	1.3E-04	2.0E-02
chr16:59475586-59477519	1934	6.7	6.5	6.9	↓	-0.4	6.7E-06	1.8E-03
chr16:60346232-60346857	626	3.8	3.3	4.2	↓	-0.9	4.3E-06	1.3E-03
chr16:60434448-60435131	684	3.0	2.5	3.3	↓	-0.8	6.4E-04	6.4E-02
chr16:61840564-61840836	273	1.9	1.1	2.4	↓	-1.3	1.3E-04	2.1E-02
chr16:61983857-61984368	512	3.9	3.5	4.2	↓	-0.7	6.9E-05	1.3E-02
chr16:62002258-62002699	442	2.3	1.5	2.8	↓	-1.3	7.6E-06	2.0E-03

chr16:62047154-62047525	372	2.9	1.7	3.6	↓	-1.8	9.7E-14	2.1E-10
chr16:6219927-6221018	1092	4.6	4.1	4.9	↓	-0.8	1.6E-09	1.2E-06
chr16:62201711-62202122	412	2.0	2.4	1.3	↑	1.1	9.9E-04	8.3E-02
chr16:6254315-6254720	406	3.0	3.3	2.6	↑	0.7	1.2E-03	9.4E-02
chr16:62951122-62951542	421	5.1	4.9	5.3	↓	-0.4	6.5E-04	6.5E-02
chr16:64611184-64611461	278	2.8	3.2	2.4	↑	0.8	1.1E-03	8.6E-02
chr16:6499572-6500072	501	4.4	3.8	4.7	↓	-0.9	6.8E-06	1.8E-03
chr16:65452926-65453408	483	4.3	3.7	4.7	↓	-1.0	1.8E-12	2.7E-09
chr16:65635573-65636040	468	3.1	2.4	3.6	↓	-1.2	1.3E-07	7.2E-05
chr16:65636170-65636557	388	2.0	1.3	2.5	↓	-1.2	1.7E-04	2.6E-02
chr16:66197397-66197680	284	1.6	0.9	2.1	↓	-1.2	8.5E-04	7.6E-02
chr16:68547476-68548035	560	4.8	4.4	5.1	↓	-0.7	4.6E-06	1.3E-03
chr16:69041334-69041928	595	3.3	2.9	3.6	↓	-0.7	6.3E-04	6.4E-02
chr16:71184560-71184920	361	2.7	2.2	3.1	↓	-0.9	8.2E-04	7.5E-02
chr16:71330722-71331264	543	2.8	1.9	3.4	↓	-1.5	1.0E-09	8.5E-07
chr16:71333478-71333849	372	2.0	1.4	2.4	↓	-1.0	1.1E-03	8.6E-02
chr16:71767916-71768683	768	3.5	3.2	3.8	↓	-0.6	9.5E-04	8.1E-02
chr16:71816296-71816618	323	3.1	2.5	3.5	↓	-0.9	5.3E-05	1.0E-02
chr16:71913644-71914404	761	4.5	4.7	4.2	↑	0.5	6.2E-04	6.4E-02
chr16:72156167-72157156	990	4.4	4.1	4.6	↓	-0.5	5.3E-04	5.7E-02
chr16:72165219-72165632	414	1.9	2.4	1.1	↑	1.2	3.1E-04	3.9E-02
chr16:73389839-73390250	412	3.2	2.8	3.6	↓	-0.8	3.8E-04	4.6E-02
chr16:73763500-73763993	494	4.3	3.9	4.6	↓	-0.7	2.2E-07	1.1E-04
chr16:74096826-74097220	395	3.7	3.1	4.2	↓	-1.0	8.7E-09	6.3E-06
chr16:74881577-74882194	618	3.2	2.7	3.6	↓	-0.9	5.1E-04	5.6E-02
chr16:75214551-75214946	396	2.7	2.2	3.0	↓	-0.8	1.3E-03	9.9E-02
chr16:75447551-75447871	321	2.1	1.4	2.5	↓	-1.2	1.3E-04	2.1E-02
chr16:77470034-77470801	768	3.0	3.5	2.1	↑	1.4	8.2E-08	4.6E-05
chr16:77471355-77471939	585	3.0	3.4	2.5	↑	0.9	2.6E-05	5.6E-03
chr16:77860821-77862006	1186	4.3	4.0	4.5	↓	-0.5	8.1E-04	7.5E-02
chr16:77974521-77975047	527	2.7	3.2	2.1	↑	1.1	1.4E-04	2.1E-02
chr16:78078008-78078449	442	3.1	3.4	2.6	↑	0.8	5.1E-04	5.6E-02
chr16:78423010-78423440	431	4.9	5.1	4.6	↑	0.5	1.1E-03	8.9E-02
chr16:78545916-78546654	739	4.4	3.7	4.8	↓	-1.1	7.1E-15	1.9E-11
chr16:78671722-78672015	294	2.2	2.7	1.2	↑	1.5	5.6E-07	2.3E-04
chr16:78896052-78896423	372	3.0	2.2	3.6	↓	-1.3	1.8E-08	1.2E-05
chr16:79619469-79619664	196	1.6	2.1	0.9	↑	1.2	9.8E-04	8.2E-02
chr16:79639396-79639852	457	2.8	2.3	3.2	↓	-0.9	9.9E-05	1.8E-02
chr16:79652867-79653252	386	2.3	2.7	1.7	↑	1.1	2.8E-04	3.7E-02
chr16:80180430-80181512	1083	5.5	5.1	5.8	↓	-0.7	6.6E-11	7.0E-08
chr16:80192103-80192678	576	3.8	3.1	4.3	↓	-1.2	4.6E-10	4.0E-07
chr16:80192748-80193070	323	3.3	2.6	3.8	↓	-1.2	2.7E-08	1.7E-05
chr16:80262433-80262881	449	3.1	2.6	3.5	↓	-0.8	8.1E-04	7.5E-02
chr16:80346661-80347181	521	4.6	4.9	4.3	↑	0.6	2.6E-04	3.6E-02
chr16:80351071-80352027	957	5.0	5.2	4.8	↑	0.4	1.1E-03	8.8E-02
chr16:80387511-80388630	1120	4.9	5.1	4.6	↑	0.5	9.9E-07	3.5E-04
chr16:80517030-80518865	1836	6.4	6.6	6.2	↑	0.3	8.3E-07	3.1E-04

chr16:80836916-80838562	1647	5.4	5.5	5.2	↑	0.3	1.0E-03	8.6E-02
chr16:81606336-81607236	901	3.7	3.3	4.0	↓	-0.6	1.3E-03	9.9E-02
chr16:81614986-81617508	2523	7.1	7.0	7.2	↓	-0.2	4.2E-05	8.3E-03
chr16:81643601-81644479	879	4.9	5.1	4.7	↑	0.5	1.0E-04	1.8E-02
chr16:82159594-82160617	1024	4.7	4.5	4.9	↓	-0.4	1.2E-03	9.3E-02
chr16:82593173-82594929	1757	6.1	5.9	6.2	↓	-0.3	2.2E-05	5.2E-03
chr16:82831534-82831913	380	3.1	2.7	3.4	↓	-0.7	6.4E-04	6.4E-02
chr16:82981737-82982630	894	4.7	4.4	4.9	↓	-0.5	3.9E-04	4.6E-02
chr16:83252840-83253399	560	3.5	3.1	3.8	↓	-0.7	1.2E-04	2.0E-02
chr16:83255482-83256461	980	4.3	4.0	4.6	↓	-0.6	1.2E-03	9.3E-02
chr16:83265771-83266988	1218	5.3	5.0	5.5	↓	-0.5	3.8E-05	8.0E-03
chr16:83282598-83282978	381	2.3	1.8	2.8	↓	-1.0	5.7E-04	6.1E-02
chr16:83520036-83520500	465	2.6	2.0	3.0	↓	-1.0	5.2E-04	5.7E-02
chr16:83626940-83627566	627	3.5	2.8	3.9	↓	-1.1	7.3E-07	2.9E-04
chr16:83632214-83634340	2127	5.5	5.3	5.7	↓	-0.4	5.0E-04	5.6E-02
chr16:83784141-83784934	794	3.9	3.4	4.2	↓	-0.8	8.0E-05	1.5E-02
chr16:83820113-83821230	1118	4.1	3.6	4.4	↓	-0.8	2.4E-05	5.3E-03
chr16:83873703-83874913	1211	5.2	5.0	5.5	↓	-0.5	4.2E-06	1.2E-03
chr16:83901915-83903940	2026	6.3	6.1	6.4	↓	-0.3	2.8E-04	3.7E-02
chr16:83957009-83958435	1427	5.3	5.0	5.5	↓	-0.5	3.0E-04	3.8E-02
chr16:84111767-84112577	811	3.9	3.1	4.4	↓	-1.2	6.1E-11	6.8E-08
chr16:84122898-84124337	1440	5.1	4.9	5.3	↓	-0.4	8.7E-04	7.8E-02
chr16:84126168-84129579	3412	7.5	7.4	7.6	↓	-0.3	1.1E-04	1.8E-02
chr16:84251612-84251965	354	3.4	2.3	4.0	↓	-1.7	2.2E-12	3.1E-09
chr16:84264367-84267625	3259	7.6	7.5	7.7	↓	-0.2	7.8E-04	7.4E-02
chr16:84364316-84365046	731	3.4	2.8	3.8	↓	-1.0	5.5E-07	2.3E-04
chr16:84369636-84370389	754	4.5	4.2	4.7	↓	-0.5	9.4E-05	1.7E-02
chr16:84378770-84382476	3707	6.7	6.5	6.9	↓	-0.4	1.8E-07	9.3E-05
chr16:84406861-84407320	460	4.4	4.0	4.7	↓	-0.7	2.8E-04	3.7E-02
chr16:84409250-84412360	3111	7.0	6.7	7.2	↓	-0.5	3.0E-13	5.8E-10
chr16:85053191-85054316	1126	6.1	6.2	5.9	↑	0.3	9.2E-04	8.0E-02
chr16:85242149-85244757	2609	6.3	6.1	6.4	↓	-0.3	9.8E-04	8.2E-02
chr16:85280460-85291833	11374	8.9	8.8	9.0	↓	-0.2	1.6E-06	5.5E-04
chr16:85312881-85314609	1729	6.7	6.5	6.8	↓	-0.3	4.0E-05	8.1E-03
chr16:85491976-85494986	3011	7.1	7.0	7.2	↓	-0.2	2.1E-04	3.1E-02
chr16:85580015-85589551	9537	9.5	9.4	9.6	↓	-0.2	1.0E-06	3.7E-04
chr16:85900807-85902418	1612	6.9	6.7	7.0	↓	-0.3	1.1E-06	3.8E-04
chr16:86017327-86017789	463	3.1	2.6	3.5	↓	-0.9	1.1E-05	2.7E-03
chr16:86118216-86118488	273	2.6	2.1	3.0	↓	-0.9	1.1E-03	8.9E-02
chr16:86343951-86344350	400	1.9	1.2	2.3	↓	-1.1	1.3E-03	9.8E-02
chr16:86545615-86546339	725	3.3	2.8	3.6	↓	-0.9	6.8E-05	1.3E-02
chr16:86614991-86616972	1982	4.9	4.5	5.1	↓	-0.6	1.5E-04	2.3E-02
chr16:86669674-86670248	575	2.7	2.2	3.1	↓	-0.9	3.5E-04	4.3E-02
chr16:9508563-9508913	351	3.1	3.5	2.7	↑	0.8	2.8E-04	3.7E-02
chr16:9514580-9514863	284	2.0	1.1	2.5	↓	-1.4	1.6E-05	4.0E-03
chr16:9676837-9678556	1720	5.3	5.0	5.5	↓	-0.5	4.4E-07	1.9E-04
chr16:9680977-9681984	1008	4.9	4.4	5.3	↓	-0.9	4.5E-15	1.4E-11

chr17:1129397-1129712	316	2.5	1.9	3.0	↓	-1.1	3.6E-05	1.3E-02
chr17:11345322-11345539	218	2.1	1.5	2.6	↓	-1.1	3.8E-04	6.4E-02
chr17:11845384-11846195	812	4.1	4.3	3.8	↑	0.6	6.8E-04	9.3E-02
chr17:11957183-11957553	371	2.8	2.0	3.3	↓	-1.3	1.8E-07	1.7E-04
chr17:12517567-12518033	467	3.1	2.7	3.5	↓	-0.8	5.0E-04	7.7E-02
chr17:14547453-14548040	588	4.4	4.0	4.7	↓	-0.7	5.6E-05	1.8E-02
chr17:14552459-14552837	379	3.5	2.3	4.1	↓	-1.7	1.4E-12	7.4E-09
chr17:14553288-14553541	254	1.8	1.1	2.3	↓	-1.3	1.4E-04	3.4E-02
chr17:14787544-14788091	548	3.9	3.5	4.1	↓	-0.6	1.3E-04	3.4E-02
chr17:15770858-15771532	675	3.7	4.0	3.2	↑	0.8	1.3E-05	5.7E-03
chr17:16300572-16300827	256	2.0	1.4	2.4	↓	-1.1	5.2E-04	7.9E-02
chr17:17803077-17803370	294	3.2	2.5	3.6	↓	-1.1	4.9E-07	4.1E-04
chr17:18873223-18873601	379	3.9	3.6	4.1	↓	-0.5	6.5E-04	9.1E-02
chr17:19007034-19007460	427	3.4	3.1	3.7	↓	-0.7	3.7E-04	6.3E-02
chr17:19073461-19073786	326	3.2	3.6	2.6	↑	1.0	1.5E-05	6.3E-03
chr17:19969840-19970103	264	2.2	1.6	2.7	↓	-1.1	2.4E-04	4.8E-02
chr17:20428474-20429380	907	3.9	3.5	4.2	↓	-0.6	9.2E-05	2.6E-02
chr17:20432722-20433081	360	3.4	2.9	3.7	↓	-0.8	7.7E-05	2.3E-02
chr17:21884200-21884421	222	2.2	1.5	2.6	↓	-1.0	6.5E-04	9.1E-02
chr17:21930226-21930651	426	3.6	3.1	4.0	↓	-0.9	1.8E-04	4.1E-02
chr17:21930716-21931744	1029	4.9	4.6	5.2	↓	-0.6	2.7E-06	1.5E-03
chr17:22067187-22068237	1051	5.9	5.7	6.1	↓	-0.4	2.3E-04	4.8E-02
chr17:22094403-22094790	388	3.2	2.7	3.5	↓	-0.7	7.2E-04	9.7E-02
chr17:22097779-22098318	540	3.5	3.0	3.9	↓	-0.9	4.7E-05	1.5E-02
chr17:22278491-22279201	711	3.2	2.6	3.6	↓	-1.0	7.0E-06	3.5E-03
chr17:22437271-22437819	549	4.3	3.9	4.6	↓	-0.7	1.2E-06	7.8E-04
chr17:22462021-22462588	568	3.3	2.7	3.7	↓	-1.0	4.0E-06	2.1E-03
chr17:22463076-22464347	1272	5.6	5.3	5.9	↓	-0.6	5.4E-08	7.0E-05
chr17:22509817-22510174	358	2.3	1.3	2.8	↓	-1.5	1.4E-06	8.4E-04
chr17:22510968-22511147	180	1.8	0.7	2.4	↓	-1.7	4.2E-07	3.6E-04
chr17:22512815-22513078	264	3.2	2.8	3.6	↓	-0.8	2.3E-04	4.8E-02
chr17:22877709-22877970	262	2.9	2.3	3.3	↓	-1.0	2.5E-05	9.6E-03
chr17:22964855-22965044	190	1.5	0.5	2.1	↓	-1.5	7.5E-05	2.3E-02
chr17:22965763-22966466	704	3.9	3.3	4.3	↓	-0.9	9.8E-08	1.1E-04
chr17:22968083-22968597	515	2.8	1.5	3.5	↓	-2.0	4.2E-14	2.7E-10
chr17:22969375-22969857	483	3.5	2.8	4.0	↓	-1.3	3.4E-11	1.1E-07
chr17:24440851-24441329	479	3.6	3.2	3.9	↓	-0.7	5.7E-04	8.3E-02
chr17:27387818-27388529	712	3.8	4.1	3.3	↑	0.9	3.4E-06	1.8E-03
chr17:27837256-27837609	354	3.0	2.4	3.4	↓	-1.0	3.4E-05	1.2E-02
chr17:28687259-28687626	368	3.0	2.5	3.4	↓	-0.9	2.5E-04	5.0E-02
chr17:31452326-31452713	388	3.2	2.7	3.5	↓	-0.8	4.2E-04	6.9E-02
chr17:32306448-32306763	316	3.2	2.8	3.6	↓	-0.8	1.6E-04	3.7E-02
chr17:33454976-33455828	853	4.3	3.8	4.7	↓	-0.9	7.8E-10	1.8E-06
chr17:33478997-33479766	770	4.2	3.9	4.4	↓	-0.5	5.1E-04	7.8E-02
chr17:33839532-33839832	301	2.3	2.8	1.6	↑	1.2	2.1E-05	8.2E-03
chr17:33980179-33981824	1646	5.8	5.6	5.9	↓	-0.3	2.7E-04	5.3E-02
chr17:33995424-33996270	847	4.8	4.5	5.0	↓	-0.6	6.2E-05	1.9E-02

chr17:34159603-34160367	765	4.1	3.8	4.4	↓	-0.7	2.3E-05	8.9E-03
chr17:34393355-34394852	1498	5.6	5.9	5.4	↑	0.5	1.6E-04	3.7E-02
chr17:34848788-34848982	195	2.0	2.4	1.3	↑	1.1	3.8E-04	6.4E-02
chr17:34877045-34878281	1237	5.6	5.4	5.8	↓	-0.4	3.8E-05	1.3E-02
chr17:34881092-34881846	755	4.0	3.6	4.3	↓	-0.6	6.7E-05	2.0E-02
chr17:36323348-36323847	500	4.0	4.3	3.7	↑	0.6	1.5E-04	3.7E-02
chr17:36829047-36829482	436	3.6	3.2	3.9	↓	-0.6	4.6E-04	7.3E-02
chr17:37255569-37256963	1395	5.9	6.1	5.7	↑	0.4	3.3E-06	1.8E-03
chr17:38802958-38803381	424	3.1	2.3	3.7	↓	-1.3	5.7E-07	4.5E-04
chr17:39243988-39244513	526	5.2	4.9	5.3	↓	-0.4	4.6E-04	7.3E-02
chr17:40664267-40664596	330	2.9	2.3	3.3	↓	-1.0	1.1E-04	2.8E-02
chr17:40720930-40721242	313	3.1	3.4	2.6	↑	0.7	4.6E-04	7.3E-02
chr17:41010277-41011514	1238	5.6	5.4	5.8	↓	-0.3	4.6E-04	7.3E-02
chr17:41166336-41166664	329	2.3	2.9	1.4	↑	1.5	5.2E-07	4.2E-04
chr17:41560728-41561028	301	3.8	3.4	4.1	↓	-0.7	2.0E-04	4.3E-02
chr17:42185938-42187088	1151	5.6	5.8	5.4	↑	0.3	5.1E-04	7.8E-02
chr17:43916954-43917359	406	3.8	3.4	4.0	↓	-0.6	5.1E-04	7.8E-02
chr17:45250823-45251131	309	2.4	2.9	1.4	↑	1.5	1.8E-07	1.7E-04
chr17:45976755-45977467	713	4.1	3.8	4.4	↓	-0.6	1.0E-04	2.7E-02
chr17:46018323-46019300	978	5.3	5.1	5.5	↓	-0.4	5.3E-04	7.9E-02
chr17:46482256-46482742	487	3.8	4.1	3.5	↑	0.6	4.8E-04	7.5E-02
chr17:46580527-46580843	317	2.3	1.5	2.8	↓	-1.3	8.2E-05	2.3E-02
chr17:46607726-46609159	1434	5.6	5.3	5.9	↓	-0.7	1.1E-10	3.2E-07
chr17:46852563-46852935	373	2.9	2.4	3.3	↓	-0.9	1.0E-04	2.7E-02
chr17:47275143-47275680	538	3.7	2.8	4.2	↓	-1.4	2.8E-14	2.5E-10
chr17:47399071-47399666	596	2.8	2.1	3.2	↓	-1.1	3.9E-05	1.3E-02
chr17:47537562-47537829	268	2.6	3.0	1.9	↑	1.1	5.9E-05	1.8E-02
chr17:47788955-47789248	294	2.3	2.7	1.7	↑	1.1	6.9E-04	9.4E-02
chr17:48071838-48072310	473	4.1	3.6	4.4	↓	-0.8	4.6E-06	2.4E-03
chr17:48374714-48375875	1162	6.4	6.0	6.7	↓	-0.6	2.0E-09	4.0E-06
chr17:48606144-48606539	396	4.2	3.8	4.4	↓	-0.6	2.3E-04	4.8E-02
chr17:48702830-48703418	589	3.7	4.2	2.9	↑	1.3	2.0E-06	1.2E-03
chr17:48703534-48704027	494	2.9	3.3	2.4	↑	0.9	1.7E-04	4.0E-02
chr17:48704370-48704678	309	2.6	3.0	2.1	↑	0.9	4.2E-04	6.9E-02
chr17:48929458-48930020	563	4.2	3.9	4.5	↓	-0.6	5.4E-04	7.9E-02
chr17:48944218-48944394	177	1.6	0.8	2.1	↓	-1.4	1.5E-04	3.7E-02
chr17:48988822-48989888	1067	5.3	5.1	5.5	↓	-0.4	3.2E-04	5.8E-02
chr17:50597639-50597983	345	2.4	1.6	2.9	↓	-1.4	2.0E-06	1.2E-03
chr17:50764717-50765045	329	2.2	1.6	2.7	↓	-1.0	5.5E-04	8.1E-02
chr17:5094639-5095025	387	2.8	3.2	2.3	↑	0.9	1.9E-04	4.2E-02
chr17:51894571-51895063	493	4.7	4.3	5.1	↓	-0.9	1.9E-07	1.7E-04
chr17:51945080-51945418	339	2.1	1.5	2.6	↓	-1.1	1.4E-04	3.4E-02
chr17:52048038-52048774	737	4.7	4.5	4.9	↓	-0.4	3.3E-04	6.0E-02
chr17:52054098-52054784	687	3.5	3.2	3.8	↓	-0.6	5.6E-04	8.2E-02
chr17:52303181-52303596	416	4.2	4.5	3.7	↑	0.8	1.1E-06	7.4E-04
chr17:52465449-52465942	494	3.0	2.3	3.5	↓	-1.3	7.4E-08	9.1E-05
chr17:52480773-52481969	1197	5.4	5.5	5.2	↑	0.4	7.8E-05	2.3E-02

chr17:52640516-52641345	830	4.2	4.4	3.8	↑	0.6	3.0E-04	5.7E-02
chr17:52780001-52780420	420	4.1	3.7	4.4	↓	-0.7	3.2E-04	5.8E-02
chr17:53261906-53262206	301	2.9	2.0	3.4	↓	-1.4	6.9E-09	1.3E-05
chr17:53726868-53727315	448	3.8	4.0	3.4	↑	0.6	2.0E-04	4.3E-02
chr17:53813931-53814365	435	3.3	3.7	2.9	↑	0.7	4.2E-04	6.9E-02
chr17:54162445-54162848	404	3.7	3.2	4.0	↓	-0.9	1.3E-05	5.7E-03
chr17:54866946-54867572	627	4.7	4.5	4.9	↓	-0.4	4.2E-04	6.9E-02
chr17:54890636-54891385	750	5.1	4.9	5.4	↓	-0.5	8.6E-06	3.9E-03
chr17:55001273-55001987	715	3.7	3.2	4.0	↓	-0.8	1.1E-04	3.0E-02
chr17:55014848-55015791	944	4.2	3.9	4.5	↓	-0.7	1.3E-04	3.4E-02
chr17:55170859-55171111	253	2.1	2.5	1.4	↑	1.2	1.5E-04	3.6E-02
chr17:56198624-56200139	1516	6.1	6.0	6.3	↓	-0.3	2.2E-04	4.7E-02
chr17:56843313-56844302	990	4.5	4.7	4.3	↑	0.4	6.2E-04	8.8E-02
chr17:57386784-57387431	648	3.9	4.2	3.6	↑	0.6	6.0E-04	8.6E-02
chr17:57487544-57488093	550	4.7	4.9	4.4	↑	0.4	3.6E-04	6.2E-02
chr17:57499157-57501563	2407	6.4	6.6	6.2	↑	0.3	1.6E-04	3.7E-02
chr17:57913671-57914628	958	6.5	6.3	6.6	↓	-0.3	1.4E-04	3.4E-02
chr17:58343372-58344170	799	5.2	5.0	5.4	↓	-0.4	1.8E-04	4.1E-02
chr17:58488040-58488309	270	2.0	1.2	2.4	↓	-1.2	3.4E-04	6.0E-02
chr17:58566826-58567659	834	4.8	4.4	5.1	↓	-0.6	8.7E-06	3.9E-03
chr17:58704231-58706107	1877	6.7	6.6	6.8	↓	-0.2	4.8E-04	7.5E-02
chr17:59003992-59005146	1155	5.1	4.8	5.3	↓	-0.5	4.5E-06	2.3E-03
chr17:59105687-59106122	436	2.9	3.3	2.3	↑	1.0	3.2E-05	1.2E-02
chr17:59346360-59347141	782	5.0	4.7	5.2	↓	-0.5	2.9E-05	1.1E-02
chr17:59347443-59347758	316	2.6	2.1	3.0	↓	-0.9	6.7E-04	9.3E-02
chr17:59402726-59405438	2713	7.8	7.7	7.9	↓	-0.2	3.1E-04	5.7E-02
chr17:59493633-59493948	316	1.9	2.3	1.2	↑	1.1	5.9E-04	8.6E-02
chr17:59839537-59840672	1136	5.3	4.9	5.6	↓	-0.6	4.5E-08	7.0E-05
chr17:59923255-59926231	2977	7.1	7.2	6.9	↑	0.3	4.9E-05	1.6E-02
chr17:60146417-60147109	693	4.4	4.2	4.7	↓	-0.5	5.2E-04	7.8E-02
chr17:60152291-60153032	742	3.9	3.4	4.2	↓	-0.8	9.5E-07	6.6E-04
chr17:60793568-60794006	439	4.5	4.1	4.8	↓	-0.7	2.9E-04	5.6E-02
chr17:60897343-60897821	479	3.3	2.8	3.7	↓	-0.8	6.1E-04	8.7E-02
chr17:60944251-60944949	699	5.1	4.7	5.4	↓	-0.6	9.0E-07	6.4E-04
chr17:61031999-61032715	717	4.1	3.7	4.3	↓	-0.6	1.8E-04	4.1E-02
chr17:61172144-61172433	290	2.8	2.2	3.2	↓	-1.0	2.0E-05	8.0E-03
chr17:61627119-61627506	388	3.2	2.8	3.5	↓	-0.7	3.0E-04	5.7E-02
chr17:61870487-61871096	610	4.2	3.9	4.5	↓	-0.6	2.0E-05	8.0E-03
chr17:62185879-62186101	223	2.2	1.5	2.7	↓	-1.3	3.8E-05	1.3E-02
chr17:62410047-62410286	240	2.5	1.4	3.0	↓	-1.6	4.9E-08	7.0E-05
chr17:62755888-62756170	283	2.8	2.0	3.3	↓	-1.3	9.6E-08	1.1E-04
chr17:62879201-62879398	198	1.8	1.0	2.3	↓	-1.4	9.8E-05	2.7E-02
chr17:62971546-62971970	425	4.0	3.6	4.3	↓	-0.7	1.6E-05	6.4E-03
chr17:63133127-63133847	721	5.2	4.9	5.4	↓	-0.4	3.4E-04	6.0E-02
chr17:63241319-63241622	304	2.5	2.9	2.0	↑	1.0	3.7E-04	6.3E-02
chr17:63712784-63713411	628	3.7	4.0	3.3	↑	0.6	2.5E-04	5.0E-02
chr17:63822062-63822295	234	2.2	1.6	2.7	↓	-1.1	1.2E-04	3.1E-02

chr17:64038690-64039436	747	3.9	4.2	3.6	↑	0.6	3.1E-04	5.8E-02
chr17:64475658-64476168	511	3.8	3.5	4.1	↓	-0.7	2.0E-04	4.3E-02
chr17:64634199-64634641	443	3.4	2.8	3.8	↓	-1.0	6.1E-04	8.7E-02
chr17:64738872-64740337	1466	6.2	6.0	6.5	↓	-0.5	1.2E-07	1.3E-04
chr17:64758796-64761116	2321	6.5	6.4	6.6	↓	-0.2	3.1E-04	5.7E-02
chr17:64789854-64792183	2330	6.4	6.3	6.6	↓	-0.3	2.9E-04	5.5E-02
chr17:64833928-64835904	1977	6.2	6.0	6.3	↓	-0.4	3.9E-05	1.3E-02
chr17:64841666-64843237	1572	4.9	4.6	5.2	↓	-0.6	1.1E-05	4.6E-03
chr17:64878350-64878918	569	4.7	4.2	5.1	↓	-0.9	4.5E-12	2.0E-08
chr17:65053502-65053896	395	2.4	2.8	1.8	↑	1.0	3.1E-04	5.8E-02
chr17:65334133-65334560	428	3.1	2.6	3.4	↓	-0.8	7.9E-05	2.3E-02
chr17:65462080-65463080	1001	4.3	3.9	4.6	↓	-0.6	2.4E-05	9.3E-03
chr17:65550820-65551160	341	2.9	3.2	2.4	↑	0.8	7.2E-04	9.7E-02
chr17:65648847-65649497	651	5.6	5.8	5.4	↑	0.3	5.3E-05	1.7E-02
chr17:65778251-65779156	906	4.6	4.3	4.9	↓	-0.5	1.0E-04	2.7E-02
chr17:65889750-65891179	1430	6.4	6.2	6.5	↓	-0.3	1.4E-04	3.4E-02
chr17:66192106-66193118	1013	5.9	5.7	6.2	↓	-0.5	9.6E-07	6.6E-04
chr17:66414708-66415982	1275	5.4	5.0	5.7	↓	-0.6	2.0E-07	1.8E-04
chr17:66624748-66625266	519	3.3	2.9	3.6	↓	-0.7	6.8E-04	9.3E-02
chr17:66694728-66695690	963	5.4	5.2	5.6	↓	-0.5	1.6E-07	1.6E-04
chr17:66696827-66700331	3505	7.6	7.5	7.7	↓	-0.2	3.2E-05	1.2E-02
chr17:66871474-66872022	549	5.1	4.9	5.3	↓	-0.4	2.5E-04	5.0E-02
chr17:66937654-66940330	2677	6.5	6.3	6.7	↓	-0.4	1.2E-06	7.8E-04
chr17:66961150-66961999	850	3.9	3.4	4.2	↓	-0.8	2.7E-06	1.5E-03
chr17:66970034-66971439	1406	5.8	5.5	6.1	↓	-0.6	5.3E-08	7.0E-05
chr17:66979849-66980970	1122	5.7	5.4	5.9	↓	-0.5	5.0E-08	7.0E-05
chr17:67181836-67182241	406	3.0	2.5	3.4	↓	-0.9	7.8E-05	2.3E-02
chr17:67245003-67246489	1487	5.5	5.1	5.8	↓	-0.7	8.3E-09	1.4E-05
chr17:67274543-67275784	1242	5.0	4.7	5.2	↓	-0.6	2.0E-04	4.3E-02
chr17:67717149-67717405	257	2.1	1.1	2.7	↓	-1.6	6.1E-07	4.7E-04
chr17:67736903-67737580	678	5.0	4.6	5.3	↓	-0.7	7.1E-07	5.3E-04
chr17:67977893-67980187	2295	6.3	6.2	6.5	↓	-0.3	7.5E-06	3.6E-03
chr17:68069431-68071187	1757	6.2	5.9	6.4	↓	-0.4	3.3E-10	8.4E-07
chr17:68102562-68104592	2031	6.7	6.6	6.8	↓	-0.2	3.4E-04	6.0E-02
chr17:68251452-68252462	1011	4.8	4.6	5.0	↓	-0.5	2.2E-04	4.6E-02
chr17:68291891-68293589	1699	7.1	7.0	7.2	↓	-0.2	5.3E-04	7.9E-02
chr17:68295861-68296711	851	6.1	5.9	6.4	↓	-0.5	8.8E-06	3.9E-03
chr17:68468388-68469781	1394	6.9	6.8	7.0	↓	-0.2	3.8E-04	6.4E-02
chr17:68595834-68596386	553	4.6	3.9	5.1	↓	-1.2	8.4E-20	1.1E-15
chr17:68615475-68616438	964	5.7	5.2	6.0	↓	-0.8	8.0E-12	3.0E-08
chr17:68737233-68738542	1310	6.2	6.1	6.4	↓	-0.3	2.5E-04	5.0E-02
chr17:68739075-68739628	554	3.6	3.2	3.9	↓	-0.7	3.5E-04	6.2E-02
chr17:68797244-68797966	723	4.2	3.8	4.5	↓	-0.7	7.7E-06	3.6E-03
chr17:68889406-68892820	3415	7.5	7.4	7.6	↓	-0.2	7.2E-06	3.5E-03
chr17:68896896-68897824	929	4.9	4.6	5.1	↓	-0.5	2.3E-04	4.8E-02
chr17:68926940-68932848	5909	8.8	8.7	8.9	↓	-0.2	1.8E-09	3.9E-06
chr17:69254480-69254912	433	3.0	2.4	3.4	↓	-0.9	4.3E-04	7.0E-02

chr17:69332530-69333118	589	4.1	3.7	4.3	↓	-0.6	5.4E-04	7.9E-02
chr17:69333195-69333728	534	4.9	4.0	5.4	↓	-1.4	1.2E-21	3.2E-17
chr17:8026295-8026583	289	2.0	2.4	1.3	↑	1.2	2.8E-04	5.5E-02
chr17:9946127-9946601	475	3.2	3.5	2.7	↑	0.8	3.7E-04	6.3E-02
chr18:10568957-10569343	387	3.4	3.7	3.0	↑	0.7	3.7E-04	6.7E-02
chr18:10570560-10571176	617	4.8	5.0	4.5	↑	0.5	9.6E-05	2.5E-02
chr18:1103190-1105138	1949	6.8	6.6	7.0	↓	-0.4	1.4E-05	6.8E-03
chr18:1112623-1113915	1293	6.7	6.6	6.9	↓	-0.3	4.3E-05	1.5E-02
chr18:12436384-12436993	610	5.5	5.2	5.7	↓	-0.5	1.2E-07	1.1E-04
chr18:12436997-12437456	460	2.9	2.3	3.3	↓	-0.9	5.4E-05	1.8E-02
chr18:1252994-1257489	4496	7.8	7.8	7.9	↓	-0.2	2.8E-05	1.1E-02
chr18:12791645-12792623	979	4.7	3.9	5.2	↓	-1.3	2.3E-12	7.7E-09
chr18:12820631-12820902	272	3.2	2.4	3.8	↓	-1.4	5.1E-10	9.1E-07
chr18:12849855-12850488	634	4.0	3.7	4.3	↓	-0.6	6.0E-05	1.9E-02
chr18:13536471-13536725	255	3.0	2.4	3.5	↓	-1.1	5.1E-05	1.7E-02
chr18:13542629-13542870	242	2.2	1.5	2.6	↓	-1.1	4.4E-04	7.2E-02
chr18:1453397-1455671	2275	7.3	7.2	7.4	↓	-0.2	5.7E-04	8.5E-02
chr18:16055945-16056328	384	3.2	3.6	2.8	↑	0.8	7.8E-05	2.2E-02
chr18:16793802-16794112	311	2.6	3.0	2.0	↑	1.0	7.5E-05	2.2E-02
chr18:1679663-1680126	464	4.4	4.2	4.6	↓	-0.5	4.5E-04	7.2E-02
chr18:17191163-17191448	286	1.9	2.4	1.1	↑	1.3	7.8E-05	2.2E-02
chr18:17569276-17569540	265	2.7	2.1	3.1	↓	-1.0	2.7E-04	5.5E-02
chr18:17569845-17571675	1831	6.7	6.6	6.8	↓	-0.3	5.5E-04	8.4E-02
chr18:17636877-17637338	462	3.4	3.7	3.0	↑	0.7	6.3E-04	9.1E-02
chr18:17707141-17708193	1053	5.2	5.0	5.4	↓	-0.4	2.7E-04	5.4E-02
chr18:17747956-17748437	482	3.9	3.6	4.2	↓	-0.6	1.7E-04	4.0E-02
chr18:17795429-17796167	739	4.6	4.1	4.9	↓	-0.9	9.3E-07	7.0E-04
chr18:17820474-17821175	702	5.3	5.0	5.5	↓	-0.5	3.3E-04	6.4E-02
chr18:1897073-1897363	291	3.9	3.6	4.2	↓	-0.6	2.9E-04	5.7E-02
chr18:19024453-19024964	512	3.3	3.0	3.6	↓	-0.7	5.1E-04	7.8E-02
chr18:19886045-19886594	550	5.8	5.6	5.9	↓	-0.4	4.2E-05	1.5E-02
chr18:20622278-20622694	417	3.5	3.8	3.1	↑	0.7	3.5E-04	6.6E-02
chr18:20760298-20760870	573	5.3	5.5	5.0	↑	0.5	1.8E-06	1.2E-03
chr18:2078471-2080537	2067	7.2	7.1	7.4	↓	-0.2	1.4E-04	3.5E-02
chr18:20888056-20888832	777	6.8	6.7	6.9	↓	-0.2	2.4E-04	5.3E-02
chr18:21043738-21044181	444	3.8	3.3	4.1	↓	-0.8	8.2E-05	2.2E-02
chr18:21139657-21140925	1269	6.4	6.3	6.5	↓	-0.2	6.2E-04	9.1E-02
chr18:2233736-2234040	305	4.4	4.0	4.7	↓	-0.7	7.8E-07	6.0E-04
chr18:23406809-23407006	198	2.6	2.0	3.0	↓	-1.0	1.3E-04	3.4E-02
chr18:2724281-2725416	1136	5.0	4.7	5.2	↓	-0.4	7.1E-04	9.8E-02
chr18:27340313-27340684	372	2.9	2.3	3.3	↓	-1.0	1.5E-05	6.8E-03
chr18:27476337-27477207	871	5.5	5.2	5.7	↓	-0.5	3.5E-06	2.0E-03
chr18:27536678-27536873	196	2.1	1.4	2.5	↓	-1.1	3.6E-04	6.6E-02
chr18:27540413-27540621	209	1.7	1.0	2.2	↓	-1.2	5.7E-04	8.5E-02
chr18:2832988-2833327	340	2.8	2.3	3.2	↓	-0.8	3.6E-04	6.6E-02
chr18:28938858-28939342	485	2.9	1.7	3.6	↓	-1.8	2.1E-13	9.5E-10
chr18:2909996-2911306	1311	5.4	5.0	5.7	↓	-0.7	2.1E-11	4.8E-08

chr18:3032618-3032898	281	2.2	1.6	2.6	↓	-1.0	4.2E-04	7.1E-02
chr18:31946652-31946883	232	2.5	2.0	2.9	↓	-1.0	4.1E-04	7.1E-02
chr18:3199948-3202579	2632	7.4	7.3	7.5	↓	-0.2	4.0E-04	6.9E-02
chr18:32542149-32542413	265	3.0	2.5	3.4	↓	-0.9	4.3E-05	1.5E-02
chr18:32793237-32793706	470	4.4	4.6	4.2	↑	0.5	3.9E-04	6.9E-02
chr18:33660493-33660777	285	2.6	2.2	3.0	↓	-0.8	7.4E-04	9.9E-02
chr18:3387368-3389683	2316	6.8	6.6	7.0	↓	-0.4	5.2E-05	1.8E-02
chr18:3427721-3430506	2786	7.3	7.2	7.4	↓	-0.2	1.9E-04	4.3E-02
chr18:34343907-34344189	283	3.3	3.6	2.8	↑	0.8	3.6E-04	6.6E-02
chr18:34457008-34457174	167	1.8	1.0	2.4	↓	-1.4	5.5E-05	1.8E-02
chr18:36326893-36327255	363	3.6	3.3	3.9	↓	-0.7	4.2E-04	7.1E-02
chr18:36943732-36944187	456	4.5	4.2	4.7	↓	-0.5	6.6E-04	9.3E-02
chr18:37270073-37270436	364	4.0	3.5	4.4	↓	-0.9	2.9E-07	2.5E-04
chr18:3773773-3774726	954	5.5	5.3	5.7	↓	-0.4	6.1E-05	1.9E-02
chr18:386121-386838	718	6.8	6.6	6.9	↓	-0.2	6.7E-04	9.4E-02
chr18:3920070-3920545	476	3.7	3.3	4.1	↓	-0.8	2.6E-06	1.5E-03
chr18:40460296-40460688	393	2.1	2.6	1.5	↑	1.1	4.7E-04	7.4E-02
chr18:4099017-4099452	436	2.3	2.8	1.6	↑	1.2	1.4E-05	6.8E-03
chr18:41126184-41126359	176	2.0	1.3	2.5	↓	-1.2	1.7E-04	4.0E-02
chr18:41834708-41834984	277	2.6	1.9	3.1	↓	-1.2	4.3E-04	7.2E-02
chr18:42459008-42459249	242	1.8	2.6	0.1	↑	2.5	3.1E-11	6.4E-08
chr18:42459356-42459711	356	3.0	3.6	1.8	↑	1.8	1.5E-12	5.6E-09
chr18:42459803-42460125	323	2.3	2.7	1.7	↑	1.1	4.1E-04	7.0E-02
chr18:42461249-42461824	576	3.5	4.0	2.7	↑	1.2	1.8E-09	2.9E-06
chr18:42461841-42462696	856	4.1	4.8	2.8	↑	2.0	2.6E-16	1.8E-12
chr18:42465700-42465970	271	2.0	2.5	1.1	↑	1.4	2.4E-05	9.6E-03
chr18:42467345-42467653	309	2.4	2.9	1.7	↑	1.1	6.6E-05	2.0E-02
chr18:42471516-42471864	349	2.3	2.9	1.2	↑	1.7	4.7E-08	5.1E-05
chr18:43386106-43386371	266	3.0	3.4	2.6	↑	0.8	6.7E-04	9.4E-02
chr18:43446919-43447167	249	2.5	2.9	2.0	↑	1.0	3.4E-04	6.4E-02
chr18:43497759-43498826	1068	4.4	4.0	4.7	↓	-0.7	7.6E-07	6.0E-04
chr18:4378706-4379267	562	4.5	4.2	4.8	↓	-0.6	4.3E-04	7.2E-02
chr18:43915415-43915732	318	3.5	3.2	3.8	↓	-0.7	4.5E-04	7.2E-02
chr18:44692531-44692976	446	2.7	3.1	2.1	↑	1.0	7.9E-05	2.2E-02
chr18:4469364-4470256	893	4.6	4.4	4.8	↓	-0.4	6.8E-04	9.4E-02
chr18:4479946-4480403	458	4.0	3.7	4.3	↓	-0.6	1.7E-04	4.0E-02
chr18:45044808-45045111	304	2.3	1.5	2.8	↓	-1.2	2.7E-05	1.0E-02
chr18:46107520-46107809	290	2.8	2.3	3.2	↓	-0.9	3.7E-04	6.7E-02
chr18:46580213-46580575	363	4.6	4.3	4.9	↓	-0.6	2.5E-05	1.0E-02
chr18:46671669-46672124	456	5.3	5.1	5.5	↓	-0.4	2.6E-05	1.0E-02
chr18:47183962-47184388	427	3.0	2.3	3.5	↓	-1.2	6.4E-06	3.3E-03
chr18:47770641-47772013	1373	5.0	5.2	4.7	↑	0.5	3.7E-04	6.7E-02
chr18:47772321-47773307	987	4.5	4.8	4.1	↑	0.7	3.7E-05	1.4E-02
chr18:47974410-47974930	521	4.2	3.8	4.5	↓	-0.7	1.6E-06	1.1E-03
chr18:4801025-4801289	265	2.5	2.9	1.9	↑	1.0	2.1E-04	4.7E-02
chr18:4819066-4819631	566	4.2	3.8	4.5	↓	-0.7	2.3E-06	1.4E-03
chr18:4819740-4821128	1389	5.8	5.6	6.0	↓	-0.4	2.1E-06	1.3E-03

chr18:48227673-48228173	501	4.2	3.8	4.5	↓	-0.7	1.3E-06	9.5E-04
chr18:48466345-48466833	489	3.9	3.6	4.2	↓	-0.6	2.5E-04	5.3E-02
chr18:48678628-48678843	216	2.5	1.9	2.9	↓	-1.1	1.8E-04	4.1E-02
chr18:4868498-4868715	218	2.3	2.7	1.6	↑	1.1	8.5E-05	2.3E-02
chr18:49063379-49063945	567	4.6	4.3	4.9	↓	-0.6	4.0E-05	1.4E-02
chr18:49988798-49989474	677	4.7	4.5	5.0	↓	-0.5	1.7E-04	4.0E-02
chr18:50014116-50015260	1145	5.9	5.5	6.2	↓	-0.7	7.8E-05	2.2E-02
chr18:50016609-50017276	668	3.0	2.0	3.5	↓	-1.6	2.8E-05	1.1E-02
chr18:50024067-50024353	287	2.3	1.4	2.9	↓	-1.5	1.8E-06	1.2E-03
chr18:50028086-50028506	421	2.4	1.3	3.0	↓	-1.7	7.6E-08	7.4E-05
chr18:50030428-50030914	487	3.1	2.2	3.7	↓	-1.5	7.3E-08	7.3E-05
chr18:50031124-50031838	715	3.2	1.9	3.9	↓	-2.0	5.0E-11	9.7E-08
chr18:50056659-50057053	395	3.1	3.6	2.3	↑	1.2	3.1E-06	1.7E-03
chr18:50057118-50057405	288	1.9	2.7	0.5	↑	2.2	5.6E-10	9.5E-07
chr18:50058249-50058464	216	1.5	2.1	0.5	↑	1.6	1.4E-05	6.8E-03
chr18:50058538-50058831	294	2.3	2.8	1.4	↑	1.4	2.3E-06	1.4E-03
chr18:50060829-50062868	2040	6.5	6.7	6.3	↑	0.4	2.6E-04	5.4E-02
chr18:50065609-50066048	440	4.1	4.4	3.6	↑	0.9	3.5E-04	6.6E-02
chr18:50066403-50068266	1864	6.3	6.6	5.9	↑	0.7	9.0E-20	1.2E-15
chr18:50075553-50075747	195	2.2	2.7	1.6	↑	1.1	2.7E-04	5.4E-02
chr18:50077613-50078478	866	4.5	4.7	4.2	↑	0.5	4.8E-04	7.5E-02
chr18:50083558-50083849	292	3.0	3.3	2.5	↑	0.9	1.0E-04	2.7E-02
chr18:50086847-50087249	403	2.7	3.1	2.0	↑	1.1	3.6E-04	6.6E-02
chr18:50095682-50096557	876	5.3	4.6	5.7	↓	-1.1	9.8E-16	5.3E-12
chr18:50134189-50134593	405	3.4	2.9	3.7	↓	-0.8	1.9E-05	8.2E-03
chr18:50400665-50401402	738	4.4	4.1	4.7	↓	-0.6	9.0E-05	2.4E-02
chr18:50688113-50688417	305	3.7	3.3	4.0	↓	-0.7	3.8E-04	6.7E-02
chr18:50760569-50760825	257	1.8	1.1	2.3	↓	-1.2	5.3E-04	8.2E-02
chr18:50809733-50810031	299	3.3	2.8	3.7	↓	-0.9	6.2E-05	1.9E-02
chr18:50838697-50839169	473	3.0	3.5	2.1	↑	1.4	1.5E-08	2.0E-05
chr18:50840029-50840722	694	3.5	3.9	3.1	↑	0.7	2.5E-04	5.4E-02
chr18:50870393-50871801	1409	5.4	5.6	5.3	↑	0.3	5.3E-04	8.2E-02
chr18:50898574-50898935	362	3.8	3.5	4.1	↓	-0.6	6.3E-04	9.1E-02
chr18:5134945-5136315	1371	6.4	6.2	6.6	↓	-0.4	8.6E-05	2.3E-02
chr18:51362307-51362555	249	2.3	2.8	1.7	↑	1.0	2.3E-04	5.2E-02
chr18:51400954-51401444	491	3.8	4.1	3.4	↑	0.7	1.8E-04	4.1E-02
chr18:51687791-51688815	1025	5.5	5.7	5.3	↑	0.3	7.0E-04	9.6E-02
chr18:52117097-52117559	463	4.1	3.7	4.4	↓	-0.6	1.7E-04	4.0E-02
chr18:52152596-52153319	724	4.7	4.4	4.9	↓	-0.6	8.2E-05	2.2E-02
chr18:52236778-52237684	907	4.6	4.1	4.9	↓	-0.8	2.8E-05	1.1E-02
chr18:52237746-52238307	562	3.6	2.9	4.1	↓	-1.3	3.1E-12	9.2E-09
chr18:52344871-52345426	556	4.3	3.9	4.6	↓	-0.7	1.6E-05	7.3E-03
chr18:52562102-52562401	300	3.2	3.6	2.8	↑	0.8	3.5E-04	6.6E-02
chr18:5275208-5275787	580	5.5	5.1	5.9	↓	-0.8	7.4E-12	1.8E-08
chr18:52880175-52880407	233	2.3	1.3	2.8	↓	-1.5	3.3E-07	2.7E-04
chr18:53137201-53137921	721	4.9	4.6	5.2	↓	-0.5	1.0E-04	2.6E-02
chr18:53193163-53193480	318	3.4	2.7	3.9	↓	-1.2	1.4E-06	9.9E-04

chr18:53287823-53288049	227	2.2	1.4	2.7	↓	-1.3	1.7E-05	7.4E-03
chr18:53402976-53403220	245	2.4	2.8	1.8	↑	1.0	6.9E-04	9.5E-02
chr18:53522780-53522943	164	2.2	2.6	1.6	↑	1.0	3.0E-04	6.0E-02
chr18:53593612-53593838	227	2.9	2.4	3.3	↓	-0.9	1.4E-04	3.6E-02
chr18:53595642-53596471	830	4.9	4.7	5.1	↓	-0.4	4.7E-04	7.4E-02
chr18:53597167-53597546	380	3.4	3.7	3.0	↑	0.7	5.0E-04	7.8E-02
chr18:53694314-53695397	1084	4.5	4.1	4.8	↓	-0.7	2.5E-04	5.3E-02
chr18:53714011-53714936	926	4.8	4.5	5.0	↓	-0.5	7.8E-05	2.2E-02
chr18:54277528-54278125	598	3.4	3.8	3.0	↑	0.7	2.5E-04	5.3E-02
chr18:54278250-54278974	725	4.7	4.3	5.0	↓	-0.8	2.1E-08	2.5E-05
chr18:54358765-54359604	840	6.0	5.8	6.2	↓	-0.4	6.5E-05	2.0E-02
chr18:54453350-54453655	306	3.1	3.5	2.6	↑	0.8	3.8E-04	6.7E-02
chr18:54474023-54474754	732	4.9	4.7	5.2	↓	-0.5	7.1E-06	3.6E-03
chr18:54508159-54508416	258	2.1	1.5	2.6	↓	-1.0	5.7E-04	8.5E-02
chr18:54508915-54509286	372	2.8	2.2	3.2	↓	-0.9	4.5E-04	7.2E-02
chr18:54592178-54592533	356	3.6	3.2	4.0	↓	-0.8	3.2E-04	6.3E-02
chr18:54852524-54852869	346	3.3	2.6	3.7	↓	-1.1	2.5E-07	2.2E-04
chr18:54852905-54853099	195	1.6	0.9	2.1	↓	-1.2	6.3E-04	9.1E-02
chr18:55266095-55266815	721	6.2	6.4	5.9	↑	0.5	1.3E-07	1.2E-04
chr18:55667934-55668302	369	3.3	3.6	2.8	↑	0.8	5.5E-05	1.8E-02
chr18:55754613-55755973	1361	6.8	6.7	6.9	↓	-0.2	2.6E-04	5.4E-02
chr18:5576039-5576374	336	4.3	3.7	4.7	↓	-1.0	1.3E-06	9.5E-04
chr18:57123253-57123482	230	1.6	2.1	0.9	↑	1.2	4.5E-04	7.2E-02
chr18:57125894-57126107	214	2.7	3.0	2.2	↑	0.8	6.4E-04	9.2E-02
chr18:57329594-57330299	706	5.7	5.5	5.9	↓	-0.5	2.1E-08	2.5E-05
chr18:57371843-57372177	335	3.8	4.2	3.2	↑	1.0	1.2E-08	1.7E-05
chr18:57602814-57603297	484	4.1	4.4	3.8	↑	0.6	2.4E-04	5.3E-02
chr18:57742561-57743103	543	5.8	5.7	6.0	↓	-0.3	6.0E-04	8.8E-02
chr18:57840648-57841464	817	4.5	4.2	4.8	↓	-0.6	1.9E-05	8.2E-03
chr18:57881383-57881842	460	4.9	4.6	5.2	↓	-0.6	1.2E-05	6.0E-03
chr18:5803267-5805961	2695	7.3	7.4	7.2	↑	0.2	1.9E-04	4.3E-02
chr18:58132154-58132454	301	3.0	2.4	3.3	↓	-0.9	1.4E-04	3.5E-02
chr18:58346621-58347739	1119	6.0	6.1	5.8	↑	0.3	1.7E-05	7.6E-03
chr18:58371012-58371630	619	4.5	4.2	4.8	↓	-0.5	7.7E-05	2.2E-02
chr18:58421566-58421987	422	5.2	4.7	5.6	↓	-0.9	3.4E-12	9.3E-09
chr18:58613583-58614595	1013	5.1	4.3	5.6	↓	-1.3	8.8E-18	7.9E-14
chr18:58684399-58684982	584	5.0	4.7	5.3	↓	-0.6	6.3E-06	3.3E-03
chr18:5886100-5886955	856	4.5	3.8	5.0	↓	-1.2	7.3E-09	1.1E-05
chr18:59182438-59182678	241	2.0	2.5	1.2	↑	1.3	5.6E-05	1.8E-02
chr18:59192319-59192968	650	3.6	3.9	3.2	↑	0.7	6.6E-05	2.0E-02
chr18:60011211-60011555	345	4.2	4.4	3.8	↑	0.6	4.4E-04	7.2E-02
chr18:60130194-60131000	807	5.9	5.7	6.0	↓	-0.3	2.8E-04	5.5E-02
chr18:60193187-60193560	374	4.2	4.5	3.7	↑	0.8	2.2E-06	1.3E-03
chr18:60260480-60260793	314	2.8	2.3	3.2	↓	-0.9	3.2E-04	6.2E-02
chr18:60346883-60348715	1833	6.0	5.7	6.2	↓	-0.6	8.2E-09	1.2E-05
chr18:60359334-60359725	392	5.3	4.9	5.6	↓	-0.7	2.2E-05	9.2E-03
chr18:60362854-60363326	473	4.3	4.0	4.6	↓	-0.6	2.5E-05	9.9E-03

chr18:60400731-60401081	351	2.6	2.0	3.0	↓	-1.0	1.7E-04	4.0E-02
chr18:60403307-60403715	409	3.2	2.6	3.7	↓	-1.1	1.6E-05	7.3E-03
chr18:60449635-60450783	1149	5.8	5.7	5.9	↓	-0.3	4.6E-04	7.4E-02
chr18:60593701-60593948	248	2.9	3.3	2.4	↑	0.9	1.1E-04	2.9E-02
chr18:6213663-6214783	1121	5.7	5.3	5.9	↓	-0.6	5.6E-08	5.8E-05
chr18:6531571-6532335	765	4.6	4.4	4.8	↓	-0.4	5.8E-04	8.7E-02
chr18:6556729-6557377	649	5.0	4.8	5.2	↓	-0.4	6.6E-04	9.4E-02
chr18:6704484-6705619	1136	5.0	4.7	5.2	↓	-0.5	5.7E-06	3.1E-03
chr18:6712458-6713434	977	5.7	5.4	5.9	↓	-0.5	3.6E-08	4.1E-05
chr18:6982782-6983245	464	2.9	3.3	2.5	↑	0.8	7.0E-04	9.6E-02
chr18:713304-714179	876	5.9	5.2	6.4	↓	-1.1	2.5E-21	6.6E-17
chr18:731180-731609	430	3.5	3.1	3.9	↓	-0.7	4.1E-04	7.0E-02
chr18:8089762-8090483	722	4.4	4.6	4.1	↑	0.5	2.7E-04	5.4E-02
chr18:8091364-8091577	214	2.4	2.8	1.9	↑	1.0	4.2E-04	7.1E-02
chr18:9210038-9210302	265	2.3	1.8	2.7	↓	-0.9	7.3E-04	9.9E-02
chr18:9213611-9214098	488	5.3	5.1	5.6	↓	-0.5	5.9E-04	8.8E-02
chr2:1004371-1009540	5170	8.2	8.1	8.3	↓	-0.2	1.4E-04	3.8E-02
chr2:100931936-100932173	238	2.2	2.7	1.6	↑	1.0	4.2E-04	7.7E-02
chr2:101625695-101626358	664	3.4	2.8	3.7	↓	-0.9	7.9E-06	4.7E-03
chr2:1018903-1020629	1727	6.6	6.5	6.7	↓	-0.2	5.0E-04	8.3E-02
chr2:103851470-103852353	884	4.8	4.5	5.1	↓	-0.6	2.4E-05	1.1E-02
chr2:104345946-104346320	375	2.5	2.9	1.9	↑	0.9	4.2E-04	7.6E-02
chr2:1046275-1053664	7390	9.0	8.9	9.1	↓	-0.2	1.9E-05	9.0E-03
chr2:105744811-105745169	359	3.1	2.5	3.6	↓	-1.0	8.6E-07	7.7E-04
chr2:106944019-106944803	785	4.0	3.2	4.6	↓	-1.4	3.5E-15	7.9E-11
chr2:106945197-106945464	268	2.2	1.4	2.7	↓	-1.3	8.5E-05	2.7E-02
chr2:106946447-106947023	577	3.4	2.9	3.8	↓	-0.9	1.3E-05	7.0E-03
chr2:106948044-106948592	549	3.6	2.9	4.0	↓	-1.1	1.0E-08	1.4E-05
chr2:106949938-106950666	729	2.9	2.2	3.4	↓	-1.2	1.3E-06	1.1E-03
chr2:106950986-106951536	551	2.5	1.9	2.9	↓	-1.0	5.2E-04	8.4E-02
chr2:106952240-106952793	554	2.4	1.7	2.8	↓	-1.2	5.2E-05	1.9E-02
chr2:106953268-106953753	486	2.9	2.4	3.2	↓	-0.9	6.4E-04	9.7E-02
chr2:106954226-106954588	363	2.9	1.6	3.5	↓	-1.9	1.1E-13	1.0E-09
chr2:107028105-107028398	294	2.2	1.5	2.6	↓	-1.1	3.5E-04	6.8E-02
chr2:107103889-107104221	333	2.6	2.1	3.0	↓	-0.9	4.8E-04	8.1E-02
chr2:107448843-107449211	369	2.9	3.3	2.3	↑	1.0	4.9E-05	1.8E-02
chr2:108902577-108903358	782	3.7	2.8	4.2	↓	-1.3	7.1E-09	1.1E-05
chr2:109454254-109454584	331	2.7	2.0	3.1	↓	-1.1	4.1E-05	1.6E-02
chr2:110579539-110579845	307	3.3	2.7	3.7	↓	-1.0	2.9E-05	1.2E-02
chr2:110761969-110762244	276	2.5	1.9	2.9	↓	-1.0	5.7E-04	8.9E-02
chr2:112236205-112236602	398	2.8	2.0	3.3	↓	-1.3	8.9E-06	5.0E-03
chr2:112561785-112562023	239	1.5	2.0	0.6	↑	1.4	3.9E-04	7.3E-02
chr2:117188437-117188779	343	2.7	3.2	2.1	↑	1.0	1.2E-04	3.5E-02
chr2:11733372-11733641	270	3.3	2.8	3.6	↓	-0.7	4.5E-04	7.7E-02
chr2:11893807-11894388	582	2.7	2.2	3.1	↓	-0.9	3.6E-04	6.9E-02
chr2:121058042-121059597	1556	5.3	4.9	5.6	↓	-0.7	1.5E-09	3.5E-06
chr2:1215602-1217249	1648	5.8	5.6	6.0	↓	-0.5	7.4E-05	2.4E-02

chr2:1217271-1218388	1118	5.7	5.4	6.0	↓	-0.6	5.0E-09	8.8E-06
chr2:122434665-122435387	723	3.6	3.9	3.1	↑	0.8	2.9E-04	6.3E-02
chr2:123046486-123046897	412	2.6	2.0	3.0	↓	-1.0	1.7E-04	4.5E-02
chr2:123722551-123722987	437	3.5	3.0	3.8	↓	-0.8	7.1E-05	2.4E-02
chr2:123727138-123727489	352	2.2	1.5	2.6	↓	-1.1	4.5E-04	7.7E-02
chr2:124197337-124197752	416	3.9	3.6	4.2	↓	-0.6	3.5E-04	6.8E-02
chr2:125423784-125424441	658	3.4	3.7	3.0	↑	0.7	5.2E-04	8.4E-02
chr2:125991536-125991885	350	2.4	2.9	1.7	↑	1.2	2.4E-05	1.1E-02
chr2:127682413-127683501	1089	4.9	4.5	5.2	↓	-0.7	1.1E-06	9.2E-04
chr2:127912826-127914585	1760	5.1	4.8	5.4	↓	-0.6	4.4E-06	3.0E-03
chr2:128888575-128889067	493	3.0	2.4	3.4	↓	-1.0	2.9E-05	1.2E-02
chr2:131098564-131099077	514	4.1	3.7	4.3	↓	-0.6	1.6E-04	4.3E-02
chr2:131433787-131434344	558	3.1	2.7	3.5	↓	-0.8	6.1E-04	9.4E-02
chr2:133011624-133012064	441	2.2	2.7	1.6	↑	1.0	4.5E-04	7.7E-02
chr2:133033313-133033716	404	2.7	3.1	2.2	↑	1.0	5.2E-04	8.4E-02
chr2:133129693-133130910	1218	6.1	5.9	6.2	↓	-0.3	2.7E-04	6.0E-02
chr2:133328049-133328546	498	3.5	3.8	3.1	↑	0.7	5.2E-04	8.4E-02
chr2:133938847-133939814	968	3.8	4.1	3.3	↑	0.8	1.7E-05	8.2E-03
chr2:133983162-133983467	306	2.1	1.5	2.6	↓	-1.1	4.3E-04	7.7E-02
chr2:133986996-133987419	424	2.4	1.6	2.8	↓	-1.2	1.1E-04	3.3E-02
chr2:134118004-134118508	505	3.6	3.8	3.2	↑	0.6	4.2E-04	7.6E-02
chr2:134118630-134119352	723	4.3	4.5	4.0	↑	0.5	2.9E-04	6.3E-02
chr2:134240921-134241987	1067	4.9	5.2	4.7	↑	0.4	9.0E-05	2.8E-02
chr2:134256564-134256882	319	3.2	2.5	3.7	↓	-1.2	1.5E-08	2.0E-05
chr2:134658572-134659207	636	4.6	4.3	4.8	↓	-0.5	5.4E-04	8.6E-02
chr2:134878243-134878593	351	3.5	3.2	3.8	↓	-0.6	5.7E-04	8.9E-02
chr2:135207917-135208424	508	3.4	2.9	3.7	↓	-0.8	6.8E-05	2.3E-02
chr2:135469477-135469790	314	2.2	1.5	2.7	↓	-1.2	1.3E-04	3.6E-02
chr2:135613241-135613570	330	2.9	2.1	3.4	↓	-1.3	1.8E-06	1.4E-03
chr2:135616488-135617256	769	4.1	3.4	4.6	↓	-1.1	2.3E-12	1.5E-08
chr2:136142710-136143134	425	3.4	2.9	3.7	↓	-0.8	4.4E-04	7.7E-02
chr2:136295848-136298317	2470	6.3	6.5	6.2	↑	0.4	2.8E-04	6.2E-02
chr2:136563439-136564094	656	4.9	4.2	5.3	↓	-1.1	1.0E-10	3.6E-07
chr2:136696823-136697559	737	4.8	4.4	5.2	↓	-0.8	2.8E-06	2.1E-03
chr2:136801557-136801849	293	2.4	2.9	1.6	↑	1.3	5.0E-06	3.3E-03
chr2:136899950-136900314	365	2.9	3.3	2.4	↑	0.9	3.3E-04	6.6E-02
chr2:140024617-140025251	635	2.9	3.3	2.5	↑	0.8	5.4E-04	8.6E-02
chr2:140448980-140449519	540	3.9	3.4	4.2	↓	-0.8	8.2E-06	4.8E-03
chr2:14055076-14055777	702	3.4	2.8	3.8	↓	-1.0	4.0E-06	2.8E-03
chr2:14056326-14058246	1921	5.0	4.7	5.2	↓	-0.5	2.6E-04	5.8E-02
chr2:1407679-1408021	343	3.7	3.2	4.1	↓	-0.9	1.0E-04	3.0E-02
chr2:141418152-141418862	711	3.2	3.6	2.8	↑	0.8	5.0E-04	8.3E-02
chr2:142242891-142243130	240	1.9	1.3	2.4	↓	-1.1	2.9E-04	6.3E-02
chr2:142282703-142282964	262	1.8	0.9	2.4	↓	-1.5	3.4E-05	1.4E-02
chr2:142794336-142794754	419	3.4	2.8	3.8	↓	-1.0	4.1E-05	1.6E-02
chr2:142834319-142835814	1496	5.5	5.3	5.7	↓	-0.4	4.7E-05	1.7E-02
chr2:143019933-143022258	2326	6.5	6.4	6.7	↓	-0.3	2.0E-05	9.2E-03

chr2:143195309-143195855	547	3.6	3.1	4.0	↓	-0.9	2.8E-06	2.1E-03
chr2:143392287-143392571	285	2.3	1.7	2.8	↓	-1.0	2.3E-04	5.6E-02
chr2:143879120-143880326	1207	5.7	5.4	5.9	↓	-0.4	4.3E-04	7.7E-02
chr2:143902659-143903096	438	4.0	3.6	4.2	↓	-0.6	3.7E-04	7.1E-02
chr2:144473655-144474079	425	5.1	4.9	5.3	↓	-0.4	3.9E-04	7.3E-02
chr2:145045338-145046068	731	4.8	4.5	5.0	↓	-0.5	9.2E-05	2.8E-02
chr2:145155320-145155761	442	3.1	2.6	3.5	↓	-0.9	1.5E-04	4.1E-02
chr2:145401550-145402888	1339	5.3	5.1	5.5	↓	-0.4	2.4E-04	5.6E-02
chr2:146805224-146805919	696	5.0	4.7	5.2	↓	-0.5	2.6E-04	5.9E-02
chr2:147451128-147452741	1614	5.8	5.5	6.0	↓	-0.6	5.8E-06	3.5E-03
chr2:147589908-147590367	460	2.9	3.3	2.4	↑	0.8	5.0E-04	8.3E-02
chr2:147733166-147733868	703	3.3	2.9	3.6	↓	-0.7	3.1E-04	6.6E-02
chr2:147737171-147737998	828	4.4	4.1	4.6	↓	-0.6	3.2E-05	1.3E-02
chr2:14787265-14787594	330	3.3	2.8	3.6	↓	-0.8	1.6E-04	4.3E-02
chr2:148422098-148422471	374	1.9	2.5	1.0	↑	1.5	1.1E-05	6.0E-03
chr2:149257321-149257732	412	3.9	3.5	4.3	↓	-0.8	3.6E-05	1.4E-02
chr2:149266213-149266488	276	2.3	1.5	2.8	↓	-1.3	7.4E-06	4.5E-03
chr2:149568346-149569717	1372	4.9	4.6	5.1	↓	-0.4	3.5E-04	6.8E-02
chr2:149862255-149863370	1116	4.6	4.3	4.8	↓	-0.5	6.2E-04	9.6E-02
chr2:149881170-149881906	737	2.9	3.3	2.4	↑	0.9	1.7E-04	4.5E-02
chr2:150054437-150056637	2201	6.0	5.8	6.2	↓	-0.3	4.8E-04	8.1E-02
chr2:150192800-150193081	282	2.6	2.0	3.0	↓	-1.0	1.5E-04	4.2E-02
chr2:150481981-150482385	405	2.6	2.1	3.0	↓	-0.9	2.8E-04	6.2E-02
chr2:150540724-150541242	519	3.8	3.4	4.1	↓	-0.7	6.3E-05	2.2E-02
chr2:150623608-150625200	1593	5.6	5.7	5.4	↑	0.3	9.5E-05	2.9E-02
chr2:151045-152606	1562	5.4	5.0	5.6	↓	-0.6	5.8E-09	1.0E-05
chr2:1512312-1512874	563	4.7	4.2	5.1	↓	-0.8	7.0E-10	2.0E-06
chr2:151642788-151643147	360	3.5	3.1	3.8	↓	-0.7	5.7E-04	8.9E-02
chr2:152996861-152997173	313	2.4	1.8	2.8	↓	-1.0	2.5E-04	5.8E-02
chr2:153129505-153129936	432	4.4	3.9	4.8	↓	-0.9	2.9E-09	6.1E-06
chr2:15385740-15386192	453	3.2	3.6	2.7	↑	0.9	1.0E-04	3.0E-02
chr2:154406906-154407143	238	1.8	1.0	2.3	↓	-1.3	2.3E-04	5.6E-02
chr2:154422132-154423157	1026	4.7	4.4	4.9	↓	-0.5	4.0E-04	7.4E-02
chr2:155405387-155406055	669	3.5	3.8	3.0	↑	0.8	9.0E-05	2.8E-02
chr2:156540337-156540806	470	3.7	3.2	4.0	↓	-0.8	1.3E-05	6.8E-03
chr2:156546451-156546862	412	3.5	3.1	3.7	↓	-0.7	3.3E-04	6.6E-02
chr2:15656675-15657611	937	4.1	3.7	4.3	↓	-0.6	1.9E-04	4.9E-02
chr2:157069855-157070840	986	4.6	4.2	4.9	↓	-0.6	2.5E-05	1.1E-02
chr2:157077629-157080620	2992	7.9	7.8	8.0	↓	-0.2	1.4E-05	7.0E-03
chr2:157373672-157374671	1000	5.4	5.1	5.6	↓	-0.6	9.1E-06	5.1E-03
chr2:158288832-158289045	214	2.2	2.6	1.5	↑	1.1	4.5E-04	7.7E-02
chr2:15935536-15935781	246	2.5	3.0	2.0	↑	1.0	2.5E-04	5.7E-02
chr2:1598601-1600735	2135	7.3	7.1	7.6	↓	-0.5	3.4E-14	3.9E-10
chr2:159923439-159923950	512	3.0	2.4	3.4	↓	-0.9	9.4E-05	2.9E-02
chr2:16006820-16007636	817	3.9	3.3	4.4	↓	-1.1	1.0E-09	2.6E-06
chr2:161501025-161501484	460	3.4	3.0	3.8	↓	-0.8	1.3E-04	3.8E-02
chr2:162290052-162291453	1402	4.9	5.1	4.7	↑	0.4	3.0E-04	6.3E-02

chr2:162300667-162301306	640	5.9	5.7	6.1	↓	-0.5	2.9E-09	6.1E-06
chr2:17803374-17803777	404	3.1	2.5	3.5	↓	-1.0	8.0E-06	4.7E-03
chr2:18645872-18648608	2737	6.3	6.4	6.1	↑	0.4	1.5E-05	7.7E-03
chr2:18943012-18943356	345	2.7	3.0	2.2	↑	0.8	6.3E-04	9.6E-02
chr2:19023954-19025765	1812	5.5	5.3	5.6	↓	-0.4	4.7E-04	8.0E-02
chr2:19099273-19100139	867	4.3	4.0	4.6	↓	-0.6	2.5E-04	5.7E-02
chr2:19346739-19348089	1351	5.6	5.4	5.8	↓	-0.4	2.0E-04	5.0E-02
chr2:19359374-19359691	318	2.9	3.3	2.5	↑	0.8	3.0E-04	6.3E-02
chr2:19411941-19413149	1209	5.4	5.1	5.6	↓	-0.6	9.8E-09	1.4E-05
chr2:19947357-19948052	696	3.5	3.8	3.0	↑	0.8	2.0E-04	5.0E-02
chr2:2041255-2042434	1180	4.4	4.7	4.1	↑	0.7	4.3E-06	3.0E-03
chr2:2042506-2045275	2770	6.8	6.7	6.9	↓	-0.2	9.8E-05	3.0E-02
chr2:21320997-21322741	1745	5.5	5.2	5.7	↓	-0.5	1.7E-04	4.5E-02
chr2:21554186-21554563	378	3.2	3.6	2.5	↑	1.1	1.3E-06	1.1E-03
chr2:21618208-21618480	273	2.1	0.7	2.8	↓	-2.1	3.5E-10	1.1E-06
chr2:22296684-22297212	529	4.7	4.4	5.0	↓	-0.6	4.5E-04	7.7E-02
chr2:22337571-22338286	716	4.3	3.7	4.7	↓	-1.0	5.0E-06	3.3E-03
chr2:22714465-22714829	365	3.0	2.5	3.3	↓	-0.8	3.9E-04	7.3E-02
chr2:25767974-25768360	387	2.8	3.2	2.3	↑	0.9	3.3E-04	6.6E-02
chr2:2584508-2585137	630	3.7	3.1	4.2	↓	-1.1	6.1E-09	1.0E-05
chr2:26824348-26825801	1454	5.4	5.2	5.5	↓	-0.4	5.0E-04	8.3E-02
chr2:26883114-26883854	741	4.7	4.3	4.9	↓	-0.6	3.2E-04	6.6E-02
chr2:27425283-27426244	962	4.0	3.5	4.4	↓	-0.9	5.0E-06	3.3E-03
chr2:27700719-27702094	1376	5.1	4.3	5.6	↓	-1.3	5.8E-11	2.4E-07
chr2:28691218-28691586	369	3.0	2.5	3.3	↓	-0.9	2.0E-04	4.9E-02
chr2:29013246-29014034	789	4.4	4.7	4.1	↑	0.6	2.3E-04	5.5E-02
chr2:2919959-2920888	930	5.3	5.1	5.5	↓	-0.4	1.0E-04	3.0E-02
chr2:29398201-29399108	908	3.7	3.4	4.0	↓	-0.6	6.1E-04	9.4E-02
chr2:29502281-29502678	398	3.2	2.9	3.5	↓	-0.7	5.3E-04	8.5E-02
chr2:29886246-29886561	316	2.6	2.1	3.0	↓	-1.0	2.2E-04	5.4E-02
chr2:30159134-30159381	248	3.0	3.4	2.5	↑	0.8	4.3E-04	7.7E-02
chr2:30192461-30192730	270	2.1	1.5	2.6	↓	-1.1	4.5E-04	7.7E-02
chr2:30793729-30794173	445	4.4	4.1	4.7	↓	-0.5	3.7E-04	7.1E-02
chr2:31317137-31317540	404	3.8	3.4	4.1	↓	-0.7	9.6E-05	2.9E-02
chr2:3202995-3204445	1451	5.6	5.8	5.5	↑	0.3	1.9E-04	4.9E-02
chr2:32245902-32246341	440	3.6	3.9	3.2	↑	0.7	2.2E-05	1.0E-02
chr2:32531093-32531555	463	4.5	4.1	4.9	↓	-0.8	5.0E-07	4.9E-04
chr2:32543451-32543862	412	2.2	1.3	2.8	↓	-1.5	2.1E-06	1.6E-03
chr2:32644031-32644696	666	3.6	3.1	3.9	↓	-0.8	1.5E-05	7.7E-03
chr2:32647429-32647735	307	2.7	2.1	3.2	↓	-1.1	1.3E-05	7.0E-03
chr2:32658227-32660148	1922	5.1	4.8	5.4	↓	-0.6	1.7E-05	8.2E-03
chr2:3336032-3337336	1305	5.7	5.5	5.8	↓	-0.3	6.5E-04	9.8E-02
chr2:33756047-33756468	422	4.4	4.1	4.6	↓	-0.5	2.7E-04	6.1E-02
chr2:3417865-3421228	3364	7.0	6.8	7.2	↓	-0.4	6.3E-05	2.2E-02
chr2:35308452-35308678	227	2.2	2.7	1.5	↑	1.2	3.8E-05	1.5E-02
chr2:35456452-35457412	961	4.2	3.7	4.6	↓	-0.9	2.0E-07	2.1E-04
chr2:35511432-35511755	324	2.2	2.7	1.6	↑	1.1	2.4E-04	5.7E-02

chr2:35699209-35700595	1387	5.4	5.2	5.7	↓	-0.5	4.4E-08	5.0E-05
chr2:35742054-35742354	301	2.2	1.6	2.7	↓	-1.1	1.4E-04	3.8E-02
chr2:35744958-35745477	520	3.5	2.7	4.0	↓	-1.3	7.3E-11	2.8E-07
chr2:35757055-35757332	278	2.3	1.5	2.8	↓	-1.3	2.9E-05	1.2E-02
chr2:35757367-35759277	1911	5.3	4.8	5.7	↓	-0.9	3.7E-13	2.8E-09
chr2:35766177-35766791	615	4.6	4.3	4.9	↓	-0.6	2.0E-04	5.0E-02
chr2:35768583-35771232	2650	6.3	6.0	6.6	↓	-0.6	2.3E-11	1.1E-07
chr2:35773142-35774949	1808	6.0	5.7	6.3	↓	-0.5	3.7E-12	2.1E-08
chr2:35880545-35880925	381	2.4	1.8	2.8	↓	-1.0	3.3E-04	6.6E-02
chr2:35883330-35884258	929	4.1	3.7	4.4	↓	-0.7	3.2E-05	1.3E-02
chr2:36007794-36008162	369	3.7	3.2	4.1	↓	-0.9	8.6E-06	4.9E-03
chr2:36067687-36068605	919	4.0	3.1	4.6	↓	-1.5	3.0E-14	3.9E-10
chr2:36143816-36144099	284	1.9	2.4	0.9	↑	1.6	5.4E-06	3.4E-03
chr2:42138681-42139019	339	2.8	2.3	3.2	↓	-0.9	3.0E-04	6.3E-02
chr2:42235928-42237273	1346	4.2	3.7	4.6	↓	-0.9	4.7E-06	3.2E-03
chr2:42331250-42332004	755	4.8	5.0	4.5	↑	0.5	5.2E-04	8.4E-02
chr2:4246273-4246655	383	3.1	3.4	2.6	↑	0.9	4.9E-04	8.2E-02
chr2:42564078-42564954	877	4.5	4.2	4.7	↓	-0.5	1.4E-04	3.8E-02
chr2:42735551-42736451	901	4.5	4.2	4.8	↓	-0.6	5.5E-05	2.0E-02
chr2:42855748-42856606	859	4.3	4.5	4.0	↑	0.6	4.3E-04	7.7E-02
chr2:42885524-42886070	547	3.0	3.6	2.1	↑	1.4	3.6E-09	7.2E-06
chr2:42892106-42892604	499	2.8	3.1	2.3	↑	0.9	3.5E-04	6.8E-02
chr2:42910885-42911520	636	3.3	3.6	2.8	↑	0.8	7.0E-05	2.4E-02
chr2:42913944-42915802	1859	4.7	5.0	4.3	↑	0.7	1.4E-06	1.1E-03
chr2:42917966-42918367	402	3.3	3.6	2.8	↑	0.8	2.8E-04	6.2E-02
chr2:43516423-43517023	601	5.5	5.3	5.7	↓	-0.4	7.2E-05	2.4E-02
chr2:44293954-44294523	570	2.9	2.3	3.2	↓	-0.9	1.1E-04	3.3E-02
chr2:44378960-44380462	1503	4.9	5.1	4.6	↑	0.5	1.3E-05	7.0E-03
chr2:44451495-44453307	1813	4.9	5.1	4.7	↑	0.4	1.0E-04	3.0E-02
chr2:44495139-44495814	676	3.7	3.3	4.1	↓	-0.7	1.7E-05	8.2E-03
chr2:44697555-44698160	606	4.0	3.6	4.2	↓	-0.6	2.5E-04	5.8E-02
chr2:46236094-46237390	1297	5.7	5.4	5.9	↓	-0.5	2.4E-07	2.4E-04
chr2:46492295-46492697	403	4.0	3.5	4.4	↓	-0.9	1.7E-05	8.2E-03
chr2:47179112-47179859	748	4.1	3.8	4.4	↓	-0.6	4.6E-05	1.7E-02
chr2:48607134-48607717	584	2.9	3.3	2.2	↑	1.1	5.1E-06	3.3E-03
chr2:50076388-50079781	3394	7.2	7.1	7.4	↓	-0.3	2.9E-04	6.3E-02
chr2:50097530-50097945	416	4.1	3.5	4.6	↓	-1.1	1.3E-08	1.8E-05
chr2:50251133-50252467	1335	4.7	4.9	4.4	↑	0.5	3.0E-05	1.2E-02
chr2:50492272-50492870	599	5.4	5.1	5.7	↓	-0.5	7.4E-07	6.8E-04
chr2:50549581-50550038	458	3.5	3.1	3.8	↓	-0.7	4.9E-04	8.2E-02
chr2:51811636-51812022	387	3.8	4.1	3.4	↑	0.8	2.6E-05	1.2E-02
chr2:51816323-51816803	481	3.4	2.9	3.8	↓	-1.0	1.7E-06	1.4E-03
chr2:52551700-52552176	477	3.9	3.6	4.2	↓	-0.6	4.2E-04	7.7E-02
chr2:52575975-52577447	1473	5.9	5.5	6.2	↓	-0.7	9.4E-06	5.2E-03
chr2:53133780-53134063	284	2.9	2.4	3.2	↓	-0.9	1.3E-04	3.7E-02
chr2:53917041-53917570	530	3.5	3.0	3.9	↓	-0.8	5.2E-04	8.4E-02
chr2:5488993-5491727	2735	6.4	6.2	6.5	↓	-0.3	3.6E-06	2.6E-03

chr2:559893-560729	837	4.8	5.0	4.6	↑	0.5	2.1E-04	5.1E-02
chr2:5850286-5850657	372	3.0	2.5	3.4	↓	-0.8	5.1E-04	8.4E-02
chr2:58949200-58949697	498	2.8	3.2	2.4	↑	0.8	6.5E-04	9.8E-02
chr2:59530316-59530775	460	3.3	3.6	2.9	↑	0.7	2.1E-04	5.1E-02
chr2:60844875-60845153	279	2.4	1.5	2.9	↓	-1.5	1.2E-06	1.1E-03
chr2:6101907-6103370	1464	5.1	4.7	5.3	↓	-0.6	4.3E-05	1.6E-02
chr2:6266278-6268249	1972	5.8	5.6	6.0	↓	-0.4	2.7E-05	1.2E-02
chr2:6282481-6282749	269	3.0	3.3	2.6	↑	0.8	4.0E-04	7.4E-02
chr2:6445188-6446971	1784	5.7	5.4	5.9	↓	-0.4	2.8E-07	2.8E-04
chr2:65766032-65766417	386	2.2	1.6	2.7	↓	-1.1	4.4E-04	7.7E-02
chr2:65948583-65949188	606	2.9	3.3	2.5	↑	0.8	5.7E-04	8.9E-02
chr2:6622119-6623322	1204	4.5	4.2	4.7	↓	-0.6	1.2E-05	6.5E-03
chr2:66397804-66398192	389	2.9	2.4	3.3	↓	-1.0	2.7E-05	1.2E-02
chr2:6639932-6640554	623	4.1	3.7	4.4	↓	-0.8	5.7E-07	5.5E-04
chr2:66402031-66402806	776	3.8	3.5	4.1	↓	-0.6	3.2E-04	6.6E-02
chr2:67379174-67379717	544	3.8	3.4	4.1	↓	-0.7	2.5E-04	5.7E-02
chr2:684415-686013	1599	6.3	6.1	6.5	↓	-0.4	8.7E-07	7.7E-04
chr2:6866828-6867211	384	3.8	3.5	4.1	↓	-0.7	4.4E-04	7.7E-02
chr2:69767905-69768705	801	4.1	3.7	4.4	↓	-0.6	6.7E-05	2.3E-02
chr2:69846710-69847080	371	2.0	2.5	1.3	↑	1.2	2.4E-04	5.6E-02
chr2:70088967-70089489	523	3.4	3.7	3.0	↑	0.7	3.2E-04	6.6E-02
chr2:70195827-70196257	431	3.6	3.0	4.0	↓	-1.0	1.0E-07	1.1E-04
chr2:70357909-70358873	965	4.4	4.7	4.0	↑	0.6	2.1E-04	5.1E-02
chr2:7050881-7051379	499	3.2	3.5	2.7	↑	0.9	4.2E-05	1.6E-02
chr2:71166145-71167025	881	4.0	3.2	4.5	↓	-1.3	7.6E-10	2.1E-06
chr2:71527722-71528234	513	2.7	2.2	3.1	↓	-0.9	5.7E-04	8.9E-02
chr2:71871465-71871828	364	2.2	1.6	2.6	↓	-1.0	5.5E-04	8.6E-02
chr2:72288353-72289141	789	3.8	4.1	3.4	↑	0.8	2.0E-04	5.0E-02
chr2:72458726-72458981	256	1.9	1.1	2.4	↓	-1.3	7.1E-05	2.4E-02
chr2:73449079-73449539	461	4.4	4.6	4.2	↑	0.4	6.5E-04	9.8E-02
chr2:75296424-75296855	432	3.9	3.5	4.2	↓	-0.7	7.9E-05	2.6E-02
chr2:75357333-75357894	562	5.1	4.9	5.3	↓	-0.4	1.8E-04	4.6E-02
chr2:75478160-75478664	505	2.9	2.3	3.4	↓	-1.1	1.6E-05	7.9E-03
chr2:75814645-75814865	221	2.0	1.2	2.5	↓	-1.3	6.6E-05	2.3E-02
chr2:75841995-75842566	572	4.3	4.6	4.0	↑	0.6	3.9E-04	7.3E-02
chr2:75906693-75907468	776	4.2	4.4	3.8	↑	0.6	4.7E-05	1.7E-02
chr2:75914557-75915930	1374	6.6	6.8	6.4	↑	0.4	3.2E-04	6.6E-02
chr2:75982486-75982915	430	1.8	2.4	1.0	↑	1.3	7.9E-05	2.6E-02
chr2:77148643-77150650	2008	5.7	5.4	5.9	↓	-0.5	3.6E-08	4.2E-05
chr2:77335809-77336398	590	3.4	3.7	3.0	↑	0.7	5.0E-04	8.3E-02
chr2:77687935-77690178	2244	6.2	6.0	6.3	↓	-0.3	2.1E-05	9.5E-03
chr2:77846120-77846773	654	4.4	3.8	4.8	↓	-1.0	5.6E-08	6.3E-05
chr2:7785053-7785685	633	2.6	3.0	2.1	↑	0.9	4.3E-04	7.7E-02
chr2:79488885-79490869	1985	7.2	6.9	7.4	↓	-0.5	4.6E-16	2.1E-11
chr2:79769142-79769854	713	4.0	3.6	4.2	↓	-0.6	8.1E-05	2.6E-02
chr2:79796019-79796570	552	3.6	3.2	3.8	↓	-0.6	3.4E-04	6.8E-02
chr2:80770396-80770926	531	4.4	3.9	4.8	↓	-0.9	7.3E-09	1.1E-05

chr2:81134089-81134518	430	3.0	3.3	2.5	↑	0.9	3.3E-04	6.6E-02
chr2:8152039-8152990	952	5.2	4.9	5.4	↓	-0.4	8.9E-05	2.8E-02
chr2:82172796-82173255	460	3.3	3.7	2.9	↑	0.7	4.5E-04	7.7E-02
chr2:82286579-82287938	1360	5.8	5.4	6.1	↓	-0.6	2.2E-08	2.7E-05
chr2:82750010-82750373	364	3.4	2.7	3.9	↓	-1.1	2.8E-08	3.4E-05
chr2:82791139-82791920	782	4.6	4.2	5.0	↓	-0.8	1.8E-08	2.4E-05
chr2:84790509-84790979	471	4.4	3.4	5.0	↓	-1.6	1.3E-11	6.4E-08
chr2:84791426-84793307	1882	5.3	5.5	5.0	↑	0.5	5.6E-05	2.0E-02
chr2:84901382-84901942	561	3.7	3.3	4.0	↓	-0.7	2.9E-04	6.3E-02
chr2:84971290-84971784	495	3.6	3.1	3.9	↓	-0.8	4.2E-05	1.6E-02
chr2:84971806-84972158	353	2.6	1.6	3.2	↓	-1.7	4.5E-09	8.3E-06
chr2:84997484-84999100	1617	5.9	5.7	6.0	↓	-0.4	3.8E-04	7.1E-02
chr2:85005700-85007528	1829	5.6	5.2	5.9	↓	-0.7	7.6E-09	1.1E-05
chr2:85007729-85009069	1341	5.3	4.8	5.7	↓	-0.9	1.1E-09	2.7E-06
chr2:85011204-85012003	800	4.0	3.5	4.3	↓	-0.8	5.4E-06	3.4E-03
chr2:85056879-85057242	364	2.2	1.5	2.6	↓	-1.0	3.8E-04	7.2E-02
chr2:85118782-85119401	620	4.0	3.4	4.4	↓	-0.9	1.4E-07	1.5E-04
chr2:85123094-85123383	290	2.4	1.9	2.8	↓	-0.9	6.6E-04	1.0E-01
chr2:85280865-85281286	422	3.0	2.3	3.5	↓	-1.2	7.2E-07	6.8E-04
chr2:85281894-85282627	734	2.9	2.4	3.3	↓	-0.9	3.3E-04	6.6E-02
chr2:85952929-85953393	465	3.9	3.5	4.2	↓	-0.7	1.0E-04	3.0E-02
chr2:86851800-86852293	494	4.0	3.3	4.4	↓	-1.1	3.9E-10	1.2E-06
chr2:8762891-8763392	502	4.0	3.6	4.2	↓	-0.6	2.8E-04	6.2E-02
chr2:87840615-87841256	642	3.6	3.2	3.9	↓	-0.7	3.3E-04	6.6E-02
chr2:88884359-88884927	569	2.6	1.9	3.1	↓	-1.3	1.8E-06	1.4E-03
chr2:88886231-88887447	1217	4.7	4.5	4.9	↓	-0.4	3.3E-04	6.6E-02
chr2:88903245-88903987	743	5.2	5.0	5.4	↓	-0.4	1.8E-04	4.6E-02
chr2:89364758-89365136	379	3.3	2.9	3.7	↓	-0.8	3.4E-04	6.8E-02
chr2:9023048-9023537	490	2.7	2.2	3.1	↓	-0.9	2.7E-04	6.0E-02
chr2:91561189-91562435	1247	4.9	4.6	5.1	↓	-0.5	7.6E-05	2.5E-02
chr2:92737005-92737976	972	4.8	4.4	5.2	↓	-0.8	4.3E-09	8.3E-06
chr2:93524199-93524592	394	2.7	3.1	2.2	↑	0.9	3.4E-04	6.7E-02
chr2:93768174-93768583	410	2.1	1.5	2.6	↓	-1.2	8.2E-05	2.6E-02
chr2:93783277-93783702	426	2.2	1.4	2.6	↓	-1.2	4.5E-05	1.7E-02
chr2:9396978-9398049	1072	4.3	4.0	4.5	↓	-0.5	1.9E-04	4.9E-02
chr2:94117611-94117974	364	2.2	2.6	1.5	↑	1.1	5.3E-04	8.5E-02
chr2:9481814-9482389	576	5.1	4.8	5.2	↓	-0.4	6.3E-04	9.6E-02
chr2:9583395-9583989	595	3.9	3.6	4.2	↓	-0.6	4.2E-04	7.6E-02
chr2:9584744-9586956	2213	6.0	5.8	6.1	↓	-0.4	1.9E-04	4.9E-02
chr2:98206691-98207088	398	1.9	2.4	1.3	↑	1.1	4.8E-04	8.1E-02
chr2:98828087-98828735	649	3.4	3.7	2.9	↑	0.7	1.7E-04	4.5E-02
chr3:100177111-100177406	296	2.5	1.9	2.9	↓	-1.0	2.9E-04	5.5E-02
chr3:100972068-100972690	623	4.2	3.7	4.6	↓	-0.8	2.7E-08	3.3E-05
chr3:101119219-101119930	712	4.7	4.4	5.0	↓	-0.7	1.1E-05	4.9E-03
chr3:101121476-101121994	519	3.2	2.6	3.6	↓	-0.9	5.5E-04	8.1E-02
chr3:101122190-101122502	313	2.7	1.8	3.3	↓	-1.4	4.1E-08	4.8E-05
chr3:101174992-101175245	254	1.8	1.1	2.3	↓	-1.3	1.7E-04	4.0E-02

chr3:101218001-101218227	227	2.8	2.2	3.2	↓	-1.0	6.6E-05	1.9E-02
chr3:101223254-101224414	1161	7.1	6.6	7.4	↓	-0.8	2.5E-09	3.7E-06
chr3:101225217-101225752	536	3.9	3.5	4.2	↓	-0.7	1.6E-04	3.7E-02
chr3:101292941-101293278	338	3.6	4.0	3.1	↑	0.8	1.8E-04	4.1E-02
chr3:101321505-101321724	220	2.0	2.4	1.3	↑	1.2	3.8E-04	6.6E-02
chr3:101423178-101424303	1126	4.4	4.7	4.1	↑	0.6	5.8E-05	1.8E-02
chr3:101440098-101441448	1351	5.6	5.9	5.0	↑	0.9	1.7E-10	4.2E-07
chr3:101450455-101450937	483	3.9	3.6	4.2	↓	-0.7	7.5E-04	9.6E-02
chr3:101452219-101452449	231	2.0	2.4	1.3	↑	1.1	7.7E-04	9.8E-02
chr3:101502161-101503148	988	5.0	5.5	4.1	↑	1.4	4.2E-11	1.2E-07
chr3:101503180-101504772	1593	5.2	5.8	4.3	↑	1.5	1.8E-16	1.4E-12
chr3:101504957-101505473	517	2.9	3.3	2.3	↑	1.0	1.1E-05	4.9E-03
chr3:101505627-101506088	462	3.9	4.3	3.5	↑	0.7	5.3E-04	7.9E-02
chr3:101506651-101507460	810	4.7	5.1	4.0	↑	1.1	7.9E-15	4.7E-11
chr3:101507465-101509426	1962	6.5	7.0	5.9	↑	1.0	5.4E-12	1.7E-08
chr3:101510090-101510997	908	4.3	4.6	3.9	↑	0.7	8.6E-05	2.4E-02
chr3:101514259-101514689	431	3.8	4.1	3.4	↑	0.7	5.0E-05	1.6E-02
chr3:101515184-101516132	949	4.7	5.1	4.1	↑	1.0	1.2E-09	2.1E-06
chr3:1020038-1021197	1160	6.8	6.6	7.0	↓	-0.3	3.1E-04	5.8E-02
chr3:102547467-102547621	155	1.6	2.1	0.8	↑	1.2	5.9E-04	8.5E-02
chr3:102942209-102942460	252	2.5	1.8	2.9	↓	-1.1	7.1E-05	2.1E-02
chr3:102942521-102943187	667	3.1	2.5	3.5	↓	-1.0	3.6E-06	2.0E-03
chr3:103116702-103116903	202	2.0	1.4	2.5	↓	-1.1	3.7E-04	6.5E-02
chr3:103313684-103314266	583	3.5	3.1	3.8	↓	-0.7	7.2E-04	9.4E-02
chr3:103391371-103392630	1260	4.8	4.6	5.0	↓	-0.4	3.1E-04	5.8E-02
chr3:103514862-103516420	1559	5.0	4.8	5.2	↓	-0.4	7.2E-04	9.4E-02
chr3:103967747-103968200	454	2.9	2.4	3.2	↓	-0.8	6.0E-04	8.5E-02
chr3:10408251-10408731	481	3.8	3.5	4.1	↓	-0.7	6.0E-04	8.5E-02
chr3:104319988-104320546	559	3.1	2.6	3.5	↓	-0.9	5.6E-05	1.7E-02
chr3:104329800-104331004	1205	4.9	4.6	5.1	↓	-0.5	3.5E-05	1.2E-02
chr3:104334037-104334851	815	4.9	4.7	5.1	↓	-0.5	5.7E-04	8.2E-02
chr3:104349952-104351091	1140	5.1	4.8	5.4	↓	-0.6	9.9E-08	1.0E-04
chr3:104402399-104402671	273	2.1	2.6	1.4	↑	1.2	1.8E-04	4.1E-02
chr3:10516228-10516676	449	3.9	3.5	4.2	↓	-0.7	1.6E-04	3.8E-02
chr3:105379-105848	470	3.7	3.4	4.0	↓	-0.6	4.5E-04	7.2E-02
chr3:10603881-10604585	705	4.2	4.5	3.9	↑	0.6	8.7E-06	4.2E-03
chr3:106169799-106170116	318	2.9	2.5	3.3	↓	-0.8	1.8E-04	4.1E-02
chr3:106230218-106230705	488	4.3	4.0	4.5	↓	-0.5	2.6E-04	5.2E-02
chr3:106875768-106876103	336	4.8	4.6	5.1	↓	-0.4	1.8E-04	4.1E-02
chr3:108757602-108758088	487	3.8	3.5	4.1	↓	-0.6	3.3E-04	6.0E-02
chr3:10906022-10906273	252	2.2	1.6	2.6	↓	-1.0	7.2E-04	9.4E-02
chr3:109281449-109281738	290	3.2	2.7	3.6	↓	-0.9	9.6E-05	2.6E-02
chr3:10931100-10931450	351	2.9	2.4	3.2	↓	-0.8	4.3E-04	7.0E-02
chr3:11107270-11108073	804	5.5	5.2	5.7	↓	-0.6	7.1E-07	5.3E-04
chr3:111933175-111933447	273	2.4	1.6	2.9	↓	-1.4	1.1E-06	8.0E-04
chr3:112590006-112590351	346	2.8	2.4	3.2	↓	-0.9	2.1E-04	4.5E-02
chr3:11271103-11271584	482	3.4	2.6	3.8	↓	-1.2	1.6E-08	2.1E-05

chr3:113123435-113123863	429	5.1	4.7	5.4	↓	-0.7	1.3E-09	2.3E-06
chr3:113284560-113284836	277	2.0	2.6	1.2	↑	1.4	1.4E-05	6.2E-03
chr3:113284896-113285295	400	3.8	4.2	3.2	↑	1.0	1.8E-09	3.0E-06
chr3:11341391-11342520	1130	6.1	6.0	6.3	↓	-0.3	4.8E-04	7.5E-02
chr3:11342676-11343231	556	4.2	3.7	4.5	↓	-0.7	1.9E-04	4.3E-02
chr3:113466260-113466763	504	3.4	2.8	3.8	↓	-1.0	7.7E-07	5.7E-04
chr3:113467086-113467567	482	3.7	3.1	4.1	↓	-1.0	3.4E-07	3.0E-04
chr3:113468556-113469547	992	4.5	4.2	4.7	↓	-0.5	7.1E-04	9.3E-02
chr3:115014071-115014623	553	4.4	4.1	4.7	↓	-0.6	5.0E-04	7.7E-02
chr3:115096931-115097274	344	2.7	2.2	3.1	↓	-0.9	6.3E-04	8.7E-02
chr3:115135644-115135935	292	2.6	3.1	1.9	↑	1.2	7.8E-06	3.8E-03
chr3:116723657-116724208	552	4.5	4.2	4.8	↓	-0.6	1.6E-05	6.8E-03
chr3:116775345-116775722	378	2.9	2.4	3.3	↓	-1.0	2.0E-04	4.3E-02
chr3:11707314-11707587	274	1.7	2.2	0.9	↑	1.3	1.7E-04	4.0E-02
chr3:11741020-11741224	205	1.8	2.3	1.1	↑	1.2	5.1E-04	7.8E-02
chr3:118282925-118283370	446	3.1	3.5	2.7	↑	0.8	2.1E-04	4.5E-02
chr3:118491974-118492247	274	2.9	3.3	2.5	↑	0.8	6.6E-04	8.9E-02
chr3:118782268-118783282	1015	5.3	5.4	5.0	↑	0.4	2.1E-04	4.5E-02
chr3:118956615-118957270	656	3.0	2.4	3.3	↓	-0.9	1.6E-04	3.7E-02
chr3:119220706-119221525	820	4.1	3.7	4.4	↓	-0.7	3.7E-06	2.0E-03
chr3:119312658-119313548	891	4.5	4.0	4.8	↓	-0.8	5.5E-07	4.4E-04
chr3:119314038-119316174	2137	6.1	6.3	5.9	↑	0.3	1.6E-06	1.1E-03
chr3:119346872-119347121	250	2.4	2.8	1.7	↑	1.1	1.3E-04	3.2E-02
chr3:12034283-12034682	400	4.0	3.5	4.4	↓	-0.9	4.1E-07	3.5E-04
chr3:120388810-120389460	651	3.3	3.6	2.9	↑	0.7	3.9E-04	6.7E-02
chr3:120477702-120478227	526	4.2	3.9	4.5	↓	-0.5	3.2E-04	5.9E-02
chr3:120924038-120924475	438	3.2	3.6	2.7	↑	0.8	4.1E-04	6.8E-02
chr3:121801414-121801919	506	4.6	4.1	5.0	↓	-0.8	2.1E-09	3.4E-06
chr3:121924100-121924399	300	2.3	2.7	1.6	↑	1.2	3.0E-05	1.1E-02
chr3:122211324-122212217	894	6.4	6.2	6.5	↓	-0.3	4.1E-04	6.8E-02
chr3:122220473-122220769	297	3.0	2.5	3.3	↓	-0.8	3.8E-04	6.6E-02
chr3:122321573-122322793	1221	5.0	5.2	4.7	↑	0.5	1.2E-04	3.2E-02
chr3:122322794-122323053	260	2.9	3.3	2.5	↑	0.8	3.8E-04	6.6E-02
chr3:122323812-122325319	1508	6.0	6.1	5.8	↑	0.3	6.2E-04	8.6E-02
chr3:12246944-12247341	398	4.3	3.6	4.8	↓	-1.2	1.3E-14	7.2E-11
chr3:12257978-12258573	596	3.1	3.6	2.4	↑	1.1	1.2E-07	1.2E-04
chr3:122636041-122636290	250	2.8	2.2	3.2	↓	-0.9	2.1E-04	4.5E-02
chr3:122669118-122669454	337	3.9	3.6	4.2	↓	-0.6	6.1E-04	8.5E-02
chr3:12270723-12271140	418	2.5	3.0	1.8	↑	1.3	5.0E-06	2.6E-03
chr3:12274932-12275620	689	3.9	4.2	3.4	↑	0.9	5.1E-06	2.7E-03
chr3:12275884-12276369	486	3.3	3.8	2.7	↑	1.0	2.1E-06	1.3E-03
chr3:12328699-12328904	206	2.3	2.8	1.8	↑	1.0	4.1E-04	6.9E-02
chr3:123761389-123761850	462	2.9	3.3	2.5	↑	0.8	3.9E-04	6.7E-02
chr3:125307840-125308239	400	2.4	2.8	1.7	↑	1.1	3.7E-05	1.2E-02
chr3:125308281-125309004	724	4.0	4.4	3.5	↑	0.9	1.6E-06	1.1E-03
chr3:125337324-125337620	297	3.0	3.4	2.5	↑	0.9	1.5E-04	3.6E-02
chr3:125774151-125774664	514	4.0	4.2	3.7	↑	0.5	5.8E-04	8.3E-02

chr3:126199780-126200377	598	3.9	3.6	4.1	↓	-0.6	5.2E-04	7.9E-02
chr3:126332547-126334085	1539	6.5	6.6	6.3	↑	0.3	3.1E-04	5.8E-02
chr3:126363405-126363605	201	2.4	2.9	1.8	↑	1.1	3.1E-05	1.1E-02
chr3:126396458-126396844	387	3.7	4.1	3.2	↑	0.8	6.0E-05	1.8E-02
chr3:126421361-126421695	335	4.0	3.7	4.3	↓	-0.6	6.6E-04	8.9E-02
chr3:126421771-126422269	499	3.5	2.9	3.9	↓	-1.0	9.5E-06	4.5E-03
chr3:126564683-126565449	767	4.4	4.1	4.6	↓	-0.6	9.5E-05	2.6E-02
chr3:126581352-126581567	216	2.0	1.4	2.4	↓	-1.1	5.8E-04	8.3E-02
chr3:126582533-126585649	3117	6.9	7.0	6.7	↑	0.2	2.6E-04	5.2E-02
chr3:126938979-126939328	350	2.8	2.3	3.2	↓	-0.9	6.6E-04	9.0E-02
chr3:127309859-127310712	854	3.4	2.9	3.8	↓	-0.9	7.7E-06	3.8E-03
chr3:128237166-128237438	273	3.0	2.6	3.4	↓	-0.8	3.1E-04	5.7E-02
chr3:128403355-128403848	494	4.2	4.5	3.9	↑	0.7	1.8E-04	4.1E-02
chr3:129561641-129562772	1132	4.9	4.7	5.1	↓	-0.4	7.4E-04	9.6E-02
chr3:129576838-129577954	1117	4.9	4.7	5.1	↓	-0.5	4.5E-04	7.2E-02
chr3:129688880-129689236	357	2.5	3.0	1.9	↑	1.0	4.0E-04	6.8E-02
chr3:129771570-129772687	1118	5.3	5.0	5.5	↓	-0.4	1.8E-05	7.1E-03
chr3:129894952-129895334	383	3.4	3.0	3.8	↓	-0.8	4.0E-05	1.3E-02
chr3:129896491-129898354	1864	5.8	6.0	5.6	↑	0.4	7.0E-04	9.2E-02
chr3:129916271-129916942	672	3.6	3.1	4.0	↓	-0.9	1.6E-07	1.5E-04
chr3:129962606-129963237	632	7.2	7.0	7.4	↓	-0.4	8.3E-07	6.1E-04
chr3:130019919-130020193	275	1.9	1.2	2.3	↓	-1.1	5.8E-04	8.3E-02
chr3:130124214-130124705	492	3.6	3.1	4.0	↓	-0.9	2.9E-05	1.0E-02
chr3:13216776-13217248	473	4.3	3.9	4.5	↓	-0.6	5.3E-04	7.9E-02
chr3:132315118-132315326	209	2.2	1.5	2.7	↓	-1.2	3.2E-05	1.1E-02
chr3:132634647-132634980	334	2.9	3.3	2.4	↑	1.0	3.7E-05	1.2E-02
chr3:132804918-132805313	396	3.3	2.9	3.6	↓	-0.8	2.5E-04	5.1E-02
chr3:133509158-133509749	592	3.2	3.6	2.8	↑	0.8	2.6E-04	5.1E-02
chr3:133523491-133523912	422	4.0	4.2	3.6	↑	0.6	2.7E-04	5.4E-02
chr3:13382788-13384289	1502	5.7	5.1	6.1	↓	-0.9	7.8E-23	2.1E-18
chr3:133883767-133884071	305	3.1	3.5	2.7	↑	0.8	2.9E-04	5.6E-02
chr3:133926300-133926697	398	3.7	3.4	4.0	↓	-0.6	7.2E-04	9.4E-02
chr3:134014083-134019326	5244	8.1	8.0	8.2	↓	-0.2	2.3E-04	4.9E-02
chr3:134410586-134411050	465	4.7	4.5	4.9	↓	-0.4	2.6E-04	5.1E-02
chr3:134731820-134733776	1957	5.8	6.0	5.6	↑	0.4	6.2E-05	1.9E-02
chr3:134947589-134948404	816	4.4	4.1	4.6	↓	-0.5	4.3E-04	7.1E-02
chr3:135027566-135029412	1847	7.6	7.5	7.8	↓	-0.2	4.4E-04	7.2E-02
chr3:135052705-135056591	3887	7.2	7.1	7.3	↓	-0.2	1.6E-04	3.8E-02
chr3:135391083-135392137	1055	5.5	5.3	5.6	↓	-0.3	6.7E-04	9.0E-02
chr3:135490414-135490947	534	4.0	3.6	4.2	↓	-0.7	2.4E-05	9.0E-03
chr3:13623591-13623954	364	2.8	3.2	2.3	↑	0.9	1.6E-04	3.7E-02
chr3:136304860-136305117	258	3.3	2.8	3.6	↓	-0.8	1.8E-04	4.1E-02
chr3:136371917-136372396	480	4.1	3.7	4.5	↓	-0.8	5.9E-07	4.6E-04
chr3:136733473-136733705	233	2.2	1.7	2.6	↓	-1.0	6.1E-04	8.5E-02
chr3:136738547-136738769	223	2.4	2.8	1.9	↑	1.0	2.8E-04	5.5E-02
chr3:136756427-136756805	379	3.7	3.3	4.0	↓	-0.7	4.5E-04	7.3E-02
chr3:137630539-137630868	330	2.8	3.2	2.3	↑	0.8	6.0E-04	8.5E-02

chr3:137650811-137651506	696	3.8	4.1	3.5	↑	0.6	2.9E-04	5.5E-02
chr3:137715822-137716370	549	4.4	4.0	4.6	↓	-0.6	8.2E-06	4.0E-03
chr3:137779356-137779704	349	3.3	3.7	2.8	↑	0.8	6.5E-05	1.9E-02
chr3:138066309-138066708	400	3.3	2.7	3.6	↓	-0.9	1.3E-05	5.6E-03
chr3:138085442-138085845	404	3.3	2.7	3.8	↓	-1.1	4.0E-08	4.7E-05
chr3:138189247-138189587	341	2.7	2.1	3.2	↓	-1.1	2.1E-05	7.9E-03
chr3:138352538-138353125	588	4.6	4.0	5.0	↓	-1.0	4.0E-09	5.8E-06
chr3:138686027-138686966	940	5.5	5.2	5.7	↓	-0.4	1.8E-04	4.1E-02
chr3:1390419-1392204	1786	7.3	7.1	7.5	↓	-0.3	4.6E-07	3.8E-04
chr3:139066866-139067422	557	4.4	3.7	4.8	↓	-1.1	5.8E-13	2.1E-09
chr3:139113008-139113363	356	3.2	2.8	3.5	↓	-0.7	5.4E-04	8.0E-02
chr3:139131824-139132101	278	3.1	2.7	3.4	↓	-0.7	6.6E-04	8.9E-02
chr3:139316860-139317363	504	3.7	4.0	3.3	↑	0.7	6.3E-04	8.7E-02
chr3:139460881-139461078	198	1.7	2.2	1.0	↑	1.3	4.1E-04	6.9E-02
chr3:139501597-139502053	457	4.9	4.2	5.3	↓	-1.1	3.1E-13	1.4E-09
chr3:139591569-139591863	295	1.9	1.1	2.4	↓	-1.3	1.1E-04	3.0E-02
chr3:140183109-140184169	1061	4.6	4.4	4.9	↓	-0.5	1.9E-04	4.2E-02
chr3:14024348-14024880	533	3.7	4.0	3.2	↑	0.9	1.5E-05	6.2E-03
chr3:140294655-140295783	1129	6.3	6.0	6.6	↓	-0.6	1.1E-10	2.7E-07
chr3:140310416-140311508	1093	6.2	5.6	6.6	↓	-1.0	4.0E-13	1.6E-09
chr3:140362049-140365097	3049	7.3	7.2	7.4	↓	-0.2	3.8E-05	1.3E-02
chr3:140690691-140691752	1062	6.1	5.8	6.3	↓	-0.5	1.4E-04	3.6E-02
chr3:140707259-140709583	2325	6.9	6.7	7.0	↓	-0.3	7.2E-05	2.1E-02
chr3:140787387-140787670	284	2.0	1.0	2.6	↓	-1.5	2.6E-06	1.6E-03
chr3:140814626-140818587	3962	7.7	7.6	7.8	↓	-0.2	1.1E-04	3.0E-02
chr3:140823682-140824841	1160	4.9	4.4	5.3	↓	-0.9	2.2E-09	3.4E-06
chr3:140860051-140862229	2179	7.2	7.0	7.4	↓	-0.3	8.0E-10	1.5E-06
chr3:140875953-140877198	1246	5.7	5.6	5.9	↓	-0.3	6.8E-04	9.0E-02
chr3:14095966-14096156	191	2.3	2.7	1.7	↑	1.1	1.2E-04	3.1E-02
chr3:141030723-141033143	2421	7.3	7.2	7.4	↓	-0.2	3.0E-04	5.6E-02
chr3:141194503-141194849	347	3.5	2.9	3.9	↓	-1.0	6.2E-06	3.2E-03
chr3:141245595-141247237	1643	6.6	6.5	6.8	↓	-0.3	9.5E-05	2.6E-02
chr3:141267770-141269122	1353	6.9	6.8	7.0	↓	-0.2	3.7E-04	6.5E-02
chr3:141410111-141412596	2486	6.9	6.8	7.1	↓	-0.2	2.8E-05	1.0E-02
chr3:141427389-141430490	3102	8.7	8.6	8.7	↓	-0.1	6.4E-05	1.9E-02
chr3:14161394-14161834	441	3.1	3.5	2.5	↑	0.9	1.5E-05	6.2E-03
chr3:141661604-141662769	1166	5.8	5.6	6.1	↓	-0.5	5.9E-09	8.1E-06
chr3:141672598-141673424	827	5.4	5.1	5.6	↓	-0.5	1.6E-06	1.1E-03
chr3:141968739-141969544	806	6.0	5.3	6.4	↓	-1.1	1.4E-17	1.3E-13
chr3:142325161-142328950	3790	7.8	7.7	7.9	↓	-0.2	5.3E-04	7.9E-02
chr3:14425764-14425992	229	2.3	1.7	2.7	↓	-1.0	3.9E-04	6.7E-02
chr3:14466434-14466954	521	3.6	3.2	4.0	↓	-0.8	1.0E-05	4.7E-03
chr3:1447669-1448334	666	4.8	4.1	5.2	↓	-1.1	2.5E-13	1.2E-09
chr3:14537302-14537913	612	4.7	4.9	4.5	↑	0.5	7.8E-04	9.9E-02
chr3:14561609-14561996	388	3.0	3.4	2.5	↑	0.9	4.5E-05	1.4E-02
chr3:14568430-14569341	912	5.4	5.5	5.2	↑	0.4	2.9E-04	5.6E-02
chr3:14740365-14741555	1191	5.4	5.5	5.2	↑	0.3	3.0E-04	5.7E-02

chr3:14745898-14746496	599	3.8	4.1	3.5	↑	0.6	6.5E-04	8.9E-02
chr3:14852692-14853148	457	4.0	4.4	3.5	↑	0.8	6.7E-07	5.1E-04
chr3:14853380-14854254	875	4.6	5.2	3.6	↑	1.6	5.2E-23	2.1E-18
chr3:14856004-14857411	1408	5.4	5.7	4.9	↑	0.9	3.4E-19	3.7E-15
chr3:14857459-14861373	3915	6.6	6.9	6.2	↑	0.7	4.1E-07	3.5E-04
chr3:14870414-14870937	524	3.3	3.6	2.9	↑	0.7	2.8E-04	5.5E-02
chr3:15522486-15522933	448	4.0	4.3	3.6	↑	0.7	1.7E-05	6.9E-03
chr3:15794166-15795801	1636	5.5	5.2	5.8	↓	-0.5	1.2E-05	5.3E-03
chr3:16193148-16194172	1025	5.9	5.6	6.0	↓	-0.4	1.3E-04	3.2E-02
chr3:16500150-16500353	204	1.9	2.4	1.3	↑	1.1	5.4E-04	8.0E-02
chr3:16587596-16587856	261	2.7	3.1	2.1	↑	1.0	1.5E-04	3.6E-02
chr3:16913580-16914303	724	4.9	4.7	5.1	↓	-0.4	5.0E-04	7.8E-02
chr3:17207667-17208726	1060	5.2	5.0	5.4	↓	-0.4	1.2E-04	3.1E-02
chr3:17513864-17514794	931	5.2	4.9	5.4	↓	-0.5	9.5E-06	4.5E-03
chr3:17582657-17583824	1168	7.8	7.6	7.9	↓	-0.2	2.3E-04	4.7E-02
chr3:1765785-1776535	10751	9.8	9.7	9.8	↓	-0.1	7.2E-05	2.1E-02
chr3:17792031-17792739	709	4.2	3.7	4.6	↓	-0.9	3.7E-07	3.2E-04
chr3:18037483-18037976	494	4.0	3.6	4.4	↓	-0.8	1.8E-07	1.6E-04
chr3:18350754-18351377	624	4.0	3.6	4.3	↓	-0.7	3.8E-05	1.3E-02
chr3:18539621-18540591	971	5.6	5.4	5.8	↓	-0.4	1.1E-05	4.9E-03
chr3:18563706-18564073	368	2.1	2.7	1.2	↑	1.5	1.9E-06	1.2E-03
chr3:18738615-18740636	2022	6.6	6.4	6.7	↓	-0.4	1.3E-04	3.3E-02
chr3:18869262-18869903	642	4.2	4.6	3.7	↑	0.9	3.9E-08	4.7E-05
chr3:18965613-18966108	496	4.4	4.0	4.6	↓	-0.6	2.3E-04	4.7E-02
chr3:18973678-18974152	475	3.9	3.6	4.2	↓	-0.6	3.5E-04	6.2E-02
chr3:19101540-19103320	1781	6.9	6.7	7.0	↓	-0.3	1.6E-06	1.1E-03
chr3:19146999-19147931	933	5.3	5.0	5.5	↓	-0.4	2.8E-04	5.4E-02
chr3:19187965-19188813	849	5.1	4.9	5.3	↓	-0.4	5.4E-04	8.0E-02
chr3:19635990-19636672	683	4.4	4.0	4.7	↓	-0.7	3.9E-06	2.1E-03
chr3:19636773-19637887	1115	5.7	5.5	5.9	↓	-0.4	2.2E-04	4.6E-02
chr3:19651852-19653527	1676	6.1	5.8	6.3	↓	-0.5	4.1E-13	1.6E-09
chr3:19798219-19798931	713	3.8	4.1	3.4	↑	0.7	4.4E-04	7.2E-02
chr3:2007113-2008436	1324	5.6	5.4	5.8	↓	-0.4	3.1E-05	1.1E-02
chr3:20542953-20544013	1061	4.8	4.5	5.1	↓	-0.6	3.1E-04	5.8E-02
chr3:20988006-20988540	535	5.0	4.7	5.2	↓	-0.5	3.9E-04	6.7E-02
chr3:21684547-21684793	247	2.7	3.2	2.2	↑	1.0	1.8E-04	4.1E-02
chr3:2175566-2176548	983	4.6	4.2	5.0	↓	-0.8	1.6E-05	6.5E-03
chr3:2272131-2272552	422	3.3	2.7	3.7	↓	-1.0	3.5E-06	2.0E-03
chr3:22960465-22960935	471	3.9	3.4	4.3	↓	-1.0	1.8E-08	2.3E-05
chr3:22982628-22983101	474	3.4	2.9	3.8	↓	-0.9	2.6E-05	9.7E-03
chr3:23029575-23030062	488	4.3	4.0	4.5	↓	-0.5	1.5E-04	3.6E-02
chr3:23058133-23058985	853	4.9	4.6	5.1	↓	-0.5	2.1E-05	7.9E-03
chr3:23548811-23549575	765	5.1	4.9	5.3	↓	-0.4	4.7E-04	7.4E-02
chr3:23714000-23714714	715	4.8	4.5	5.0	↓	-0.5	2.8E-05	1.0E-02
chr3:25810146-25810402	257	1.9	2.4	1.1	↑	1.3	2.1E-04	4.5E-02
chr3:26638901-26639260	360	3.7	3.4	4.0	↓	-0.6	7.0E-04	9.2E-02
chr3:27466093-27466428	336	2.9	3.2	2.4	↑	0.8	4.3E-04	7.1E-02

chr3:27770292-27771557	1266	5.7	5.5	5.8	↓	-0.3	3.1E-04	5.8E-02
chr3:28068790-28069109	320	2.9	2.4	3.3	↓	-0.9	4.3E-04	7.0E-02
chr3:28532572-28532787	216	2.2	1.6	2.7	↓	-1.0	7.2E-04	9.4E-02
chr3:2932703-2933142	440	4.5	4.1	4.7	↓	-0.6	4.1E-05	1.3E-02
chr3:2960064-2960281	218	2.3	2.7	1.7	↑	1.0	4.0E-04	6.8E-02
chr3:29620404-29621644	1241	5.1	4.8	5.3	↓	-0.4	8.4E-05	2.4E-02
chr3:29904160-29904685	526	4.1	3.7	4.5	↓	-0.8	6.3E-06	3.2E-03
chr3:31220705-31221516	812	4.8	4.3	5.1	↓	-0.8	7.2E-08	7.9E-05
chr3:32341881-32342624	744	4.0	3.7	4.2	↓	-0.5	5.2E-04	7.9E-02
chr3:32640630-32640855	226	2.0	1.4	2.4	↓	-1.1	7.0E-04	9.2E-02
chr3:3278422-3278641	220	2.3	1.7	2.8	↓	-1.1	7.4E-05	2.1E-02
chr3:33193028-33194175	1148	4.9	5.1	4.6	↑	0.5	6.4E-05	1.9E-02
chr3:33932298-33932741	444	3.2	2.8	3.5	↓	-0.7	7.7E-04	9.8E-02
chr3:34667918-34670476	2559	6.9	6.7	7.1	↓	-0.3	2.1E-06	1.3E-03
chr3:3467383-3468046	664	4.1	3.7	4.3	↓	-0.7	1.3E-04	3.3E-02
chr3:35491238-35491664	427	3.0	2.5	3.4	↓	-0.9	3.9E-05	1.3E-02
chr3:35621924-35622171	248	2.1	1.4	2.5	↓	-1.1	4.8E-04	7.5E-02
chr3:35643154-35643679	526	2.9	3.3	2.5	↑	0.8	6.6E-04	8.9E-02
chr3:36165799-36166144	346	4.3	3.4	4.9	↓	-1.6	1.7E-22	3.0E-18
chr3:3623316-3624923	1608	7.2	7.3	7.0	↑	0.2	1.6E-06	1.1E-03
chr3:3684885-3687963	3079	7.2	7.2	7.1	↑	0.2	5.3E-04	7.9E-02
chr3:37103875-37104075	201	2.2	2.6	1.6	↑	1.0	6.0E-04	8.5E-02
chr3:37105175-37105833	659	3.3	2.8	3.7	↓	-0.9	9.2E-05	2.6E-02
chr3:37182934-37183353	420	4.7	4.3	4.9	↓	-0.6	3.7E-06	2.0E-03
chr3:37187747-37188175	429	3.6	3.1	4.0	↓	-0.9	3.2E-05	1.1E-02
chr3:37376372-37377237	866	3.9	3.6	4.2	↓	-0.6	2.0E-04	4.4E-02
chr3:37587243-37587922	680	5.5	5.2	5.8	↓	-0.5	2.7E-05	1.0E-02
chr3:38357258-38357618	361	3.5	3.1	3.8	↓	-0.7	7.7E-04	9.8E-02
chr3:38478439-38479008	570	5.6	5.4	5.7	↓	-0.3	7.4E-04	9.5E-02
chr3:39007012-39008472	1461	5.4	5.1	5.6	↓	-0.4	6.5E-04	8.9E-02
chr3:39014556-39016120	1565	6.1	5.9	6.3	↓	-0.3	2.3E-04	4.8E-02
chr3:39561227-39562766	1540	6.6	6.4	6.8	↓	-0.3	3.7E-05	1.2E-02
chr3:4048659-4049350	692	4.1	3.7	4.3	↓	-0.6	4.1E-04	6.8E-02
chr3:40591893-40592320	428	3.0	2.4	3.5	↓	-1.0	7.6E-05	2.2E-02
chr3:40774884-40775170	287	4.8	4.5	5.0	↓	-0.4	4.9E-04	7.6E-02
chr3:4080691-4083445	2755	7.1	6.9	7.2	↓	-0.3	3.0E-06	1.8E-03
chr3:40859996-40860645	650	5.0	5.2	4.8	↑	0.4	6.0E-04	8.5E-02
chr3:40883750-40885242	1493	7.0	6.8	7.1	↓	-0.2	2.1E-04	4.5E-02
chr3:4103258-4105708	2451	6.5	6.3	6.7	↓	-0.4	1.3E-08	1.7E-05
chr3:41144227-41144571	345	3.1	2.6	3.5	↓	-0.9	3.2E-05	1.1E-02
chr3:41636909-41640248	3340	7.9	7.9	8.0	↓	-0.2	5.2E-04	7.9E-02
chr3:41701121-41703436	2316	6.5	6.3	6.7	↓	-0.4	8.7E-08	9.3E-05
chr3:41811095-41811375	281	2.8	2.3	3.2	↓	-0.9	2.4E-04	4.9E-02
chr3:41812496-41815458	2963	7.3	7.2	7.4	↓	-0.3	1.5E-07	1.4E-04
chr3:41933735-41934167	433	3.9	3.4	4.3	↓	-0.8	1.0E-06	7.2E-04
chr3:42311583-42313675	2093	6.5	6.4	6.6	↓	-0.2	6.1E-04	8.5E-02
chr3:42320335-42321074	740	4.5	4.1	4.8	↓	-0.6	1.9E-06	1.2E-03

chr3:42321906-42322339	434	2.7	2.1	3.1	↓	-1.0	4.6E-04	7.3E-02
chr3:42323380-42324606	1227	5.0	4.7	5.3	↓	-0.6	3.0E-05	1.1E-02
chr3:42324630-42326604	1975	7.0	6.9	7.2	↓	-0.4	4.7E-07	3.8E-04
chr3:4322215-4323231	1017	5.4	5.2	5.6	↓	-0.4	1.4E-04	3.4E-02
chr3:4357200-4358121	922	4.4	3.9	4.7	↓	-0.8	3.3E-06	1.9E-03
chr3:43815879-43816184	306	3.4	3.0	3.7	↓	-0.7	7.0E-04	9.3E-02
chr3:44085053-44085932	880	4.5	3.9	4.8	↓	-0.9	1.3E-11	3.9E-08
chr3:44557169-44557935	767	5.0	4.8	5.2	↓	-0.4	1.3E-04	3.3E-02
chr3:46209327-46209723	397	3.5	3.0	3.8	↓	-0.8	1.0E-04	2.7E-02
chr3:46681387-46681834	448	3.3	3.6	2.8	↑	0.8	9.4E-05	2.6E-02
chr3:46720041-46720615	575	3.9	3.6	4.2	↓	-0.6	7.8E-04	9.9E-02
chr3:4717433-4718242	810	4.2	3.6	4.6	↓	-0.9	2.5E-10	5.8E-07
chr3:47186475-47187023	549	3.7	3.4	4.0	↓	-0.6	5.3E-04	7.9E-02
chr3:47333438-47334461	1024	4.8	5.1	4.3	↑	0.8	2.4E-04	4.9E-02
chr3:47336930-47337189	260	1.8	2.3	1.0	↑	1.3	7.0E-04	9.2E-02
chr3:47513284-47513920	637	2.8	3.2	2.3	↑	0.9	5.5E-04	8.1E-02
chr3:48859209-48860074	866	4.6	4.3	4.8	↓	-0.5	3.3E-04	6.0E-02
chr3:48881871-48882032	162	1.4	1.9	0.5	↑	1.4	3.4E-04	6.1E-02
chr3:4930034-4930808	775	5.2	4.9	5.4	↓	-0.5	8.5E-05	2.4E-02
chr3:49786356-49787826	1471	5.7	5.4	5.8	↓	-0.4	1.0E-04	2.8E-02
chr3:4993791-4994123	333	4.0	3.6	4.4	↓	-0.7	2.0E-05	7.9E-03
chr3:50370005-50370326	322	3.3	3.6	2.9	↑	0.7	5.1E-04	7.9E-02
chr3:50539927-50540469	543	3.0	2.4	3.4	↓	-1.0	2.0E-05	7.9E-03
chr3:50810995-50811586	592	3.9	3.6	4.1	↓	-0.6	5.3E-04	7.9E-02
chr3:51136119-51136965	847	4.5	4.8	4.2	↑	0.5	2.1E-04	4.5E-02
chr3:51228077-51228294	218	1.6	0.8	2.1	↓	-1.3	3.5E-04	6.3E-02
chr3:51604514-51605190	677	4.0	3.5	4.3	↓	-0.8	5.4E-05	1.7E-02
chr3:51708155-51708470	316	2.9	2.4	3.3	↓	-0.9	2.4E-04	4.9E-02
chr3:51749461-51749711	251	2.3	1.7	2.8	↓	-1.1	1.5E-04	3.6E-02
chr3:51778158-51778990	833	5.3	5.1	5.4	↓	-0.4	4.7E-04	7.4E-02
chr3:52016012-52016414	403	3.0	2.1	3.5	↓	-1.5	4.4E-10	9.4E-07
chr3:52245516-52245776	261	2.7	2.3	3.1	↓	-0.8	5.7E-04	8.3E-02
chr3:52420624-52421206	583	4.8	4.5	5.0	↓	-0.4	6.7E-04	9.0E-02
chr3:5379027-5379720	694	4.6	4.3	4.8	↓	-0.5	3.8E-04	6.7E-02
chr3:5398773-5399057	285	3.2	2.8	3.6	↓	-0.9	2.7E-04	5.4E-02
chr3:55113740-55114474	735	4.9	4.6	5.1	↓	-0.5	4.6E-04	7.3E-02
chr3:55260747-55261033	287	2.3	1.7	2.7	↓	-1.0	3.5E-04	6.2E-02
chr3:55728989-55729422	434	3.7	3.2	4.0	↓	-0.8	5.8E-05	1.8E-02
chr3:55983254-55983478	225	2.3	1.8	2.7	↓	-0.9	7.2E-04	9.4E-02
chr3:55995875-55996165	291	2.2	1.5	2.7	↓	-1.1	1.9E-04	4.3E-02
chr3:56278390-56278754	365	2.0	2.4	1.3	↑	1.1	3.8E-04	6.6E-02
chr3:56725036-56725306	271	3.0	2.2	3.4	↓	-1.2	1.0E-07	1.0E-04
chr3:5715583-5715989	407	3.2	2.7	3.6	↓	-1.0	2.3E-06	1.4E-03
chr3:57207462-57207960	499	4.7	4.5	4.9	↓	-0.4	4.6E-04	7.4E-02
chr3:57231843-57232297	455	3.7	3.2	4.0	↓	-0.8	7.3E-06	3.7E-03
chr3:58022141-58022686	546	4.0	3.6	4.3	↓	-0.8	2.7E-05	1.0E-02
chr3:58445861-58446041	181	1.8	2.4	1.0	↑	1.4	5.6E-05	1.7E-02

chr3:58472906-58473977	1072	5.9	5.7	6.0	↓	-0.3	2.4E-04	4.9E-02
chr3:58672874-58673156	283	2.1	1.4	2.6	↓	-1.2	1.5E-04	3.6E-02
chr3:59518474-59518701	228	2.1	2.5	1.4	↑	1.2	2.5E-04	5.1E-02
chr3:60149240-60149581	342	2.8	2.3	3.1	↓	-0.9	3.1E-04	5.8E-02
chr3:61294886-61295274	389	2.5	1.9	2.9	↓	-1.0	1.1E-04	3.0E-02
chr3:61665191-61665638	448	3.9	3.6	4.2	↓	-0.6	5.7E-04	8.3E-02
chr3:61779478-61779783	306	3.7	4.1	3.2	↑	0.9	4.3E-07	3.6E-04
chr3:62345732-62346263	532	4.3	4.1	4.6	↓	-0.5	5.8E-04	8.3E-02
chr3:62540797-62541724	928	4.8	4.5	5.1	↓	-0.7	1.0E-07	1.0E-04
chr3:62721403-62721818	416	2.3	1.7	2.7	↓	-1.0	6.8E-04	9.1E-02
chr3:64101897-64102131	235	2.0	1.3	2.4	↓	-1.1	6.5E-04	8.9E-02
chr3:66670558-66670757	200	2.7	2.2	3.1	↓	-0.9	4.6E-04	7.4E-02
chr3:67016072-67016315	244	3.3	2.8	3.6	↓	-0.8	3.4E-04	6.1E-02
chr3:68228716-68229213	498	4.0	3.5	4.4	↓	-0.9	2.6E-05	9.8E-03
chr3:693545-699614	6070	8.8	8.7	8.9	↓	-0.1	3.2E-04	5.9E-02
chr3:69447011-69447235	225	2.5	1.6	3.1	↓	-1.5	1.4E-07	1.3E-04
chr3:70022-73125	3104	7.3	7.4	7.1	↑	0.3	2.8E-06	1.7E-03
chr3:7108235-7108440	206	2.4	1.9	2.8	↓	-1.0	5.1E-04	7.8E-02
chr3:71526169-71526704	536	4.1	3.8	4.4	↓	-0.6	2.0E-05	7.9E-03
chr3:7176789-7177113	325	3.9	3.5	4.2	↓	-0.6	3.9E-04	6.7E-02
chr3:71782747-71782930	184	1.4	0.5	1.9	↓	-1.4	6.3E-04	8.7E-02
chr3:71809713-71810562	850	5.0	4.6	5.3	↓	-0.8	4.6E-08	5.2E-05
chr3:72401839-72402211	373	4.0	3.7	4.3	↓	-0.6	7.7E-05	2.2E-02
chr3:73459253-73459859	607	3.4	3.0	3.8	↓	-0.8	5.7E-05	1.8E-02
chr3:74293929-74294423	495	3.9	3.5	4.3	↓	-0.8	2.1E-04	4.5E-02
chr3:7429790-7430045	256	3.6	3.2	3.9	↓	-0.7	3.5E-05	1.2E-02
chr3:74326777-74327092	316	2.7	3.0	2.1	↑	0.9	4.7E-04	7.4E-02
chr3:74437817-74438407	591	3.9	3.5	4.2	↓	-0.7	3.2E-04	5.9E-02
chr3:74438496-74439318	823	5.2	5.4	4.9	↑	0.5	1.5E-05	6.2E-03
chr3:74540330-74542845	2516	6.4	6.1	6.5	↓	-0.4	7.8E-06	3.8E-03
chr3:74849819-74850300	482	4.9	4.6	5.1	↓	-0.5	6.5E-04	8.9E-02
chr3:748511-750687	2177	7.0	6.8	7.1	↓	-0.2	1.2E-04	3.0E-02
chr3:750765-754135	3371	7.7	7.6	7.8	↓	-0.2	5.3E-04	7.9E-02
chr3:75198728-75199159	432	4.0	3.7	4.3	↓	-0.5	5.7E-04	8.3E-02
chr3:76622810-76623028	219	2.3	2.7	1.7	↑	1.0	3.9E-04	6.7E-02
chr3:7687933-7688307	375	2.4	1.6	2.9	↓	-1.3	3.5E-06	2.0E-03
chr3:7691608-7691870	263	3.0	2.2	3.5	↓	-1.4	6.4E-07	4.9E-04
chr3:7692356-7692856	501	3.4	2.1	4.1	↓	-2.0	1.4E-20	1.9E-16
chr3:7692954-7693310	357	2.1	0.7	2.8	↓	-2.2	4.8E-11	1.3E-07
chr3:7695006-7695799	794	3.5	2.6	4.0	↓	-1.4	3.1E-12	1.0E-08
chr3:77018247-77018896	650	4.9	4.5	5.1	↓	-0.6	1.3E-07	1.3E-04
chr3:77045139-77045979	841	4.9	5.3	4.3	↑	1.0	5.3E-10	1.1E-06
chr3:77734352-77734938	587	4.5	4.2	4.7	↓	-0.5	6.0E-05	1.8E-02
chr3:778187-779073	887	5.0	4.8	5.2	↓	-0.4	1.5E-04	3.6E-02
chr3:77857574-77857876	303	4.0	3.7	4.3	↓	-0.6	7.7E-04	9.8E-02
chr3:77896643-77897656	1014	4.5	4.1	4.9	↓	-0.8	5.8E-09	8.1E-06
chr3:7819035-7819323	289	2.8	3.2	2.2	↑	1.0	2.4E-04	4.9E-02

chr3:78263708-78263863	156	1.8	1.0	2.2	↓	-1.2	4.9E-04	7.6E-02
chr3:78959072-78959509	438	3.4	3.8	2.9	↑	0.9	4.3E-05	1.4E-02
chr3:7918190-7918608	419	4.3	3.6	4.7	↓	-1.1	6.9E-08	7.7E-05
chr3:7919059-7922609	3551	7.1	7.4	6.7	↑	0.7	1.2E-15	7.8E-12
chr3:793818-794943	1126	5.6	5.3	5.9	↓	-0.7	6.1E-10	1.2E-06
chr3:79423369-79424177	809	4.8	5.0	4.6	↑	0.4	2.2E-04	4.7E-02
chr3:8002888-8003581	694	3.9	3.6	4.1	↓	-0.6	5.6E-04	8.2E-02
chr3:8005352-8005856	505	3.4	2.9	3.8	↓	-0.9	1.0E-05	4.7E-03
chr3:80103455-80103768	314	4.1	3.5	4.5	↓	-1.0	3.6E-10	8.0E-07
chr3:80134692-80135161	470	4.5	4.2	4.7	↓	-0.5	4.7E-04	7.4E-02
chr3:80946099-80946503	405	2.4	1.7	2.8	↓	-1.1	2.9E-04	5.6E-02
chr3:818028-821537	3510	7.9	7.8	8.0	↓	-0.2	3.4E-06	2.0E-03
chr3:82605125-82605428	304	2.7	2.1	3.2	↓	-1.0	2.1E-05	7.9E-03
chr3:82606554-82606938	385	3.5	3.1	3.8	↓	-0.7	4.0E-04	6.8E-02
chr3:82611637-82612662	1026	4.5	4.1	4.9	↓	-0.8	1.5E-05	6.4E-03
chr3:82613774-82614077	304	2.3	1.7	2.7	↓	-1.1	1.8E-04	4.1E-02
chr3:829315-829577	263	2.3	1.5	2.8	↓	-1.3	8.9E-06	4.3E-03
chr3:83473640-83473923	284	2.2	1.5	2.7	↓	-1.2	1.3E-04	3.4E-02
chr3:83625337-83625748	412	3.5	3.8	3.1	↑	0.7	1.0E-04	2.8E-02
chr3:85323871-85324622	752	4.3	3.9	4.6	↓	-0.6	1.8E-05	7.2E-03
chr3:8573244-8573745	502	5.0	4.6	5.4	↓	-0.7	1.1E-09	2.0E-06
chr3:8600337-8600809	473	2.8	2.3	3.2	↓	-0.9	3.5E-04	6.3E-02
chr3:863259-863943	685	5.0	4.8	5.2	↓	-0.4	5.0E-04	7.7E-02
chr3:89945797-89946378	582	2.8	2.3	3.3	↓	-1.0	2.8E-05	1.0E-02
chr3:9007241-9007921	681	4.2	4.4	3.9	↑	0.6	1.1E-04	2.9E-02
chr3:90138225-90138472	248	2.0	1.3	2.4	↓	-1.1	6.2E-04	8.7E-02
chr3:9092984-9093557	574	5.3	5.0	5.5	↓	-0.5	1.7E-05	6.9E-03
chr3:91604713-91605201	489	3.2	2.6	3.6	↓	-1.0	3.2E-06	1.9E-03
chr3:91986122-91986364	243	2.3	1.7	2.8	↓	-1.1	2.0E-04	4.5E-02
chr3:92233610-92234216	607	3.3	2.7	3.7	↓	-0.9	3.1E-06	1.9E-03
chr3:92269034-92269250	217	1.6	0.7	2.2	↓	-1.5	4.8E-05	1.5E-02
chr3:93988035-93988350	316	2.5	1.9	2.9	↓	-1.1	8.1E-05	2.3E-02
chr3:95923796-95924269	474	4.2	3.8	4.5	↓	-0.7	5.0E-06	2.6E-03
chr3:962341-963060	720	4.6	4.2	4.9	↓	-0.7	2.3E-09	3.5E-06
chr3:9636001-9636302	302	2.7	2.2	3.1	↓	-0.9	2.7E-04	5.4E-02
chr3:967770-970049	2280	6.7	6.4	6.8	↓	-0.4	3.3E-06	1.9E-03
chr3:97840818-97841099	282	2.9	3.3	2.4	↑	0.8	4.2E-04	6.9E-02
chr3:978786-990981	12196	9.5	9.4	9.5	↓	-0.1	6.9E-06	3.5E-03
chr3:99816947-99819438	2492	6.1	5.9	6.2	↓	-0.3	2.8E-04	5.5E-02
chr3:99899693-99900022	330	3.3	2.8	3.7	↓	-0.9	1.3E-05	5.7E-03
chr3:999412-1001703	2292	6.6	6.5	6.8	↓	-0.2	7.4E-04	9.5E-02
chr4:102515908-102516163	256	2.4	2.8	1.8	↑	1.0	5.5E-04	9.9E-02
chr4:10253876-10254318	443	3.0	2.5	3.4	↓	-0.9	8.0E-05	2.7E-02
chr4:102627458-102627756	299	2.7	3.1	2.0	↑	1.1	8.1E-06	4.6E-03
chr4:103496823-103498759	1937	6.4	5.7	6.8	↓	-1.1	3.2E-48	1.5E-43
chr4:103724592-103725106	515	4.4	4.7	4.2	↑	0.5	4.2E-04	8.6E-02
chr4:103744781-103745952	1172	5.1	5.3	4.9	↑	0.4	1.3E-04	4.0E-02

chr4:104381861-104382110	250	1.8	2.3	1.1	↑	1.2	3.3E-04	7.3E-02
chr4:106391378-106392708	1331	5.6	5.3	5.8	↓	-0.4	2.7E-07	2.7E-04
chr4:10836492-10836752	261	3.1	2.6	3.5	↓	-0.9	4.3E-05	1.6E-02
chr4:109866252-109867043	792	3.3	2.9	3.7	↓	-0.8	7.8E-05	2.7E-02
chr4:109900558-109901119	562	3.2	3.5	2.8	↑	0.7	5.5E-04	9.9E-02
chr4:1100625-1101172	548	4.0	3.5	4.4	↓	-0.9	1.5E-07	1.7E-04
chr4:11064089-11065355	1267	5.3	4.9	5.6	↓	-0.7	5.4E-10	1.2E-06
chr4:110835623-110836394	772	4.7	4.2	5.0	↓	-0.8	1.2E-10	3.7E-07
chr4:110963721-110964136	416	2.2	2.7	1.5	↑	1.2	4.1E-05	1.5E-02
chr4:11121279-11125125	3847	7.8	7.7	7.9	↓	-0.2	6.1E-08	8.3E-05
chr4:111576172-111576990	819	3.3	3.6	2.8	↑	0.8	1.6E-04	4.3E-02
chr4:112016067-112016958	892	4.3	4.1	4.6	↓	-0.5	4.4E-04	8.8E-02
chr4:113144748-113146255	1508	5.9	6.1	5.7	↑	0.4	4.0E-04	8.3E-02
chr4:113146518-113147332	815	5.3	5.0	5.5	↓	-0.5	1.9E-07	2.0E-04
chr4:113675243-113675608	366	2.2	2.6	1.5	↑	1.1	2.4E-04	5.9E-02
chr4:113797350-113798342	993	4.6	4.2	4.9	↓	-0.7	1.5E-06	1.1E-03
chr4:114095325-114095580	256	2.2	2.7	1.5	↑	1.1	1.4E-04	4.1E-02
chr4:114096686-114097450	765	3.8	3.3	4.1	↓	-0.8	1.8E-05	8.5E-03
chr4:114776757-114777498	742	3.4	3.7	3.0	↑	0.7	4.3E-04	8.7E-02
chr4:114885480-114885914	435	2.6	3.1	2.1	↑	1.0	2.5E-04	6.2E-02
chr4:114895923-114896315	393	3.3	3.6	2.8	↑	0.8	1.5E-04	4.2E-02
chr4:115663613-115664264	652	4.0	3.6	4.4	↓	-0.8	6.7E-05	2.4E-02
chr4:116082756-116083347	592	3.5	3.9	3.0	↑	0.9	2.1E-06	1.5E-03
chr4:116989219-116989484	266	2.5	2.9	1.9	↑	1.0	3.4E-04	7.3E-02
chr4:118123779-118124138	360	3.2	2.6	3.7	↓	-1.1	1.4E-05	7.0E-03
chr4:118369946-118370590	645	2.7	2.2	3.1	↓	-1.0	1.3E-04	4.0E-02
chr4:118612901-118613215	315	3.1	2.6	3.4	↓	-0.8	1.2E-04	3.8E-02
chr4:118627167-118628680	1514	5.4	5.1	5.6	↓	-0.5	1.2E-05	6.3E-03
chr4:118659703-118660131	429	5.1	4.8	5.4	↓	-0.6	7.9E-09	1.4E-05
chr4:119062331-119062717	387	2.9	3.2	2.4	↑	0.8	3.1E-04	6.9E-02
chr4:119413587-119413913	327	2.8	2.3	3.2	↓	-0.8	5.4E-04	9.7E-02
chr4:119950689-119951108	420	2.5	1.9	2.9	↓	-1.0	2.9E-04	6.6E-02
chr4:120218387-120219069	683	4.7	5.0	4.4	↑	0.6	2.4E-05	1.0E-02
chr4:120508951-120509657	707	4.7	4.3	4.9	↓	-0.7	2.5E-07	2.6E-04
chr4:120691507-120691804	298	1.9	1.0	2.5	↓	-1.5	1.2E-05	6.2E-03
chr4:122330064-122330944	881	5.1	4.9	5.3	↓	-0.4	5.1E-04	9.5E-02
chr4:123674114-123674464	351	2.3	1.7	2.8	↓	-1.1	2.0E-04	5.2E-02
chr4:12416929-12417554	626	3.7	4.0	3.3	↑	0.6	2.8E-04	6.6E-02
chr4:127198870-127199325	456	2.5	1.9	2.9	↓	-1.0	1.6E-04	4.4E-02
chr4:127488249-127488797	549	2.6	2.0	3.0	↓	-1.0	1.8E-04	4.7E-02
chr4:128630391-128630883	493	3.2	2.8	3.5	↓	-0.7	2.3E-04	5.7E-02
chr4:128982617-128983886	1270	4.7	4.3	5.0	↓	-0.7	5.0E-07	4.4E-04
chr4:129406227-129406557	331	2.4	1.9	2.8	↓	-1.0	4.8E-04	9.1E-02
chr4:13011444-13012364	921	3.8	3.3	4.1	↓	-0.8	5.8E-06	3.6E-03
chr4:130551991-130552278	288	2.2	1.6	2.7	↓	-1.1	1.8E-04	4.8E-02
chr4:132365124-132365369	246	2.3	1.6	2.7	↓	-1.1	1.6E-04	4.5E-02
chr4:132497517-132498069	553	3.9	4.2	3.6	↑	0.6	4.6E-04	9.0E-02

chr4:132518468-132519528	1061	5.9	5.7	6.0	↓	-0.4	4.3E-04	8.7E-02
chr4:132765477-132766450	974	5.4	5.1	5.6	↓	-0.5	7.5E-06	4.4E-03
chr4:132903572-132903840	269	2.0	1.3	2.5	↓	-1.2	2.9E-04	6.6E-02
chr4:132982487-132983009	523	4.2	3.7	4.5	↓	-0.8	3.6E-06	2.4E-03
chr4:133000183-133000490	308	2.6	2.0	3.1	↓	-1.1	3.3E-04	7.3E-02
chr4:133038943-133039393	451	3.9	3.2	4.3	↓	-1.1	2.6E-09	5.1E-06
chr4:133058750-133059697	948	3.4	2.9	3.8	↓	-0.9	5.0E-05	1.8E-02
chr4:133337438-133337796	359	2.1	2.6	1.4	↑	1.2	9.5E-05	3.1E-02
chr4:133664833-133665378	546	3.9	4.2	3.6	↑	0.7	7.8E-05	2.7E-02
chr4:133670153-133671154	1002	4.8	4.4	5.1	↓	-0.7	5.1E-06	3.2E-03
chr4:133913268-133913760	493	3.8	3.2	4.2	↓	-1.0	4.2E-08	6.1E-05
chr4:134049959-134050271	313	2.2	2.7	1.5	↑	1.2	1.1E-04	3.4E-02
chr4:134109969-134110473	505	2.8	2.0	3.2	↓	-1.2	6.0E-07	5.1E-04
chr4:134115245-134116357	1113	5.6	5.4	5.7	↓	-0.3	5.1E-04	9.5E-02
chr4:134145139-134146284	1146	4.7	4.4	5.0	↓	-0.6	2.8E-05	1.2E-02
chr4:134192528-134193464	937	3.9	3.5	4.3	↓	-0.7	1.0E-05	5.5E-03
chr4:134253686-134254248	563	3.3	2.8	3.7	↓	-0.9	3.3E-05	1.3E-02
chr4:134255987-134256233	247	2.1	1.3	2.6	↓	-1.3	9.4E-05	3.1E-02
chr4:134258991-134259491	501	2.5	1.7	3.0	↓	-1.2	5.8E-06	3.6E-03
chr4:134259832-134262630	2799	6.6	6.5	6.8	↓	-0.4	8.6E-05	2.9E-02
chr4:134262868-134265368	2501	5.8	5.2	6.3	↓	-1.1	7.0E-38	1.6E-33
chr4:134265942-134266943	1002	3.5	2.7	4.0	↓	-1.2	8.7E-10	1.9E-06
chr4:134285330-134285769	440	3.1	2.2	3.7	↓	-1.5	3.3E-11	1.2E-07
chr4:134388949-134389262	314	2.7	2.0	3.1	↓	-1.1	1.4E-05	7.0E-03
chr4:1347353-1347949	597	4.2	3.7	4.6	↓	-0.8	1.4E-07	1.6E-04
chr4:134837825-134838191	367	2.6	2.0	3.0	↓	-0.9	2.8E-04	6.6E-02
chr4:134882211-134882626	416	4.3	4.1	4.6	↓	-0.5	4.7E-04	9.1E-02
chr4:135203821-135204130	310	1.9	2.4	1.1	↑	1.4	2.9E-05	1.2E-02
chr4:136195177-136196444	1268	4.0	3.2	4.5	↓	-1.3	1.6E-07	1.7E-04
chr4:136196668-136199318	2651	6.8	6.0	7.4	↓	-1.4	1.0E-16	1.1E-12
chr4:136201329-136201873	545	3.7	3.1	4.1	↓	-1.1	1.2E-07	1.4E-04
chr4:136246971-136247484	514	3.6	2.9	4.1	↓	-1.1	4.6E-07	4.2E-04
chr4:136333212-136334540	1329	5.1	4.7	5.3	↓	-0.6	1.3E-05	6.6E-03
chr4:136683737-136685316	1580	5.8	5.5	6.0	↓	-0.5	7.6E-06	4.4E-03
chr4:137582151-137582802	652	4.1	3.6	4.5	↓	-0.9	3.7E-06	2.4E-03
chr4:137602340-137603944	1605	5.6	5.2	5.9	↓	-0.7	1.6E-08	2.5E-05
chr4:138388303-138388625	323	2.3	1.7	2.8	↓	-1.1	2.9E-04	6.6E-02
chr4:13859096-13859438	343	3.0	2.5	3.4	↓	-0.9	1.8E-04	4.8E-02
chr4:138669486-138669821	336	2.2	2.6	1.5	↑	1.1	4.5E-04	8.9E-02
chr4:139256805-139257250	446	3.3	2.7	3.7	↓	-1.0	8.8E-06	4.8E-03
chr4:139257261-139258688	1428	5.8	5.6	6.1	↓	-0.4	2.2E-04	5.6E-02
chr4:139497038-139497892	855	4.2	3.9	4.5	↓	-0.6	8.9E-05	3.0E-02
chr4:139761156-139761951	796	5.7	5.5	5.9	↓	-0.4	3.4E-04	7.3E-02
chr4:140055792-140057644	1853	6.1	6.0	6.3	↓	-0.3	2.0E-05	8.8E-03
chr4:140157860-140158914	1055	5.2	4.9	5.4	↓	-0.5	3.6E-04	7.8E-02
chr4:141115780-141116139	360	2.9	2.4	3.2	↓	-0.9	2.7E-04	6.4E-02
chr4:141119774-141122812	3039	6.6	6.4	6.7	↓	-0.3	1.7E-05	7.9E-03

chr4:141288524-141290534	2011	6.5	6.3	6.7	↓	-0.3	1.9E-05	8.6E-03
chr4:141379653-141380408	756	4.1	3.7	4.4	↓	-0.7	5.2E-04	9.5E-02
chr4:141410274-141410707	434	3.7	3.3	4.0	↓	-0.7	2.7E-04	6.5E-02
chr4:14142238-14142731	494	2.6	1.8	3.2	↓	-1.4	9.1E-07	6.9E-04
chr4:141520940-141521642	703	5.0	4.7	5.2	↓	-0.5	9.8E-05	3.1E-02
chr4:141710648-141710974	327	2.3	2.7	1.5	↑	1.2	1.0E-04	3.2E-02
chr4:141934644-141935209	566	4.0	4.3	3.6	↑	0.7	2.3E-05	9.8E-03
chr4:142231712-142232106	395	3.8	3.5	4.1	↓	-0.6	4.7E-04	9.0E-02
chr4:14622472-14622796	325	2.5	3.0	1.9	↑	1.0	1.6E-04	4.5E-02
chr4:15626422-15628839	2418	6.6	6.8	6.5	↑	0.3	2.7E-04	6.5E-02
chr4:16497139-16497527	389	2.3	2.8	1.7	↑	1.0	1.8E-04	4.7E-02
chr4:1672134-1673223	1090	5.5	5.3	5.7	↓	-0.4	2.8E-04	6.6E-02
chr4:18103954-18104222	269	1.9	2.4	1.1	↑	1.3	1.3E-04	4.0E-02
chr4:18110262-18111272	1011	5.4	5.2	5.6	↓	-0.4	8.9E-05	3.0E-02
chr4:18117139-18117546	408	2.8	3.2	2.3	↑	0.9	1.9E-04	4.8E-02
chr4:18138456-18138744	289	3.8	4.1	3.3	↑	0.7	1.1E-04	3.6E-02
chr4:19958855-19959234	380	3.5	3.1	3.9	↓	-0.8	3.8E-05	1.5E-02
chr4:20323177-20323601	425	3.4	3.7	2.9	↑	0.8	2.2E-04	5.6E-02
chr4:20336612-20336915	304	2.3	0.9	3.0	↓	-2.1	6.8E-12	3.4E-08
chr4:20338816-20340015	1200	4.7	4.2	5.1	↓	-0.9	2.2E-10	5.9E-07
chr4:22250336-22250657	322	2.3	1.7	2.7	↓	-1.0	4.6E-04	9.0E-02
chr4:24657542-24658005	464	3.7	2.8	4.3	↓	-1.4	3.0E-13	1.9E-09
chr4:24705941-24706270	330	1.9	1.2	2.3	↓	-1.1	4.5E-04	8.9E-02
chr4:2477256-2478806	1551	6.2	6.0	6.4	↓	-0.3	1.7E-04	4.6E-02
chr4:24830113-24830607	495	3.3	2.9	3.7	↓	-0.8	1.5E-04	4.2E-02
chr4:26503625-26504343	719	5.4	5.2	5.5	↓	-0.4	3.8E-05	1.5E-02
chr4:27592826-27593446	621	2.8	1.9	3.4	↓	-1.5	2.0E-09	4.1E-06
chr4:27600072-27600591	520	2.4	1.6	3.0	↓	-1.3	1.8E-06	1.2E-03
chr4:2896565-2899110	2546	6.6	6.4	6.8	↓	-0.3	1.4E-06	1.0E-03
chr4:29044796-29045293	498	3.1	2.5	3.5	↓	-0.9	4.5E-04	8.9E-02
chr4:29212826-29213015	190	1.5	0.8	2.1	↓	-1.3	5.1E-04	9.5E-02
chr4:30193577-30194043	467	2.6	2.9	2.1	↑	0.9	5.1E-04	9.5E-02
chr4:31923557-31925281	1725	5.9	6.1	5.8	↑	0.3	2.1E-04	5.4E-02
chr4:32655094-32655513	420	2.5	1.7	3.1	↓	-1.4	4.9E-07	4.4E-04
chr4:3487613-3488282	670	4.0	3.6	4.3	↓	-0.7	3.1E-05	1.3E-02
chr4:34925983-34927514	1532	5.0	4.7	5.3	↓	-0.5	1.7E-05	8.0E-03
chr4:35548729-35549156	428	2.4	1.8	2.9	↓	-1.0	5.3E-04	9.6E-02
chr4:3627222-3627866	645	3.7	3.3	4.0	↓	-0.7	2.8E-04	6.6E-02
chr4:37320657-37321088	432	2.5	1.9	2.9	↓	-1.0	3.0E-04	6.8E-02
chr4:37541904-37544140	2237	5.9	5.6	6.1	↓	-0.5	1.1E-10	3.5E-07
chr4:37545323-37547487	2165	5.4	5.1	5.6	↓	-0.5	1.6E-05	7.7E-03
chr4:3820309-3821983	1675	5.7	5.5	5.9	↓	-0.4	4.5E-06	2.9E-03
chr4:38289539-38290456	918	5.3	5.0	5.6	↓	-0.5	2.1E-05	9.1E-03
chr4:38369187-38369434	248	1.9	0.9	2.5	↓	-1.5	5.9E-06	3.6E-03
chr4:38586311-38587045	735	3.4	2.8	3.8	↓	-1.0	7.7E-07	6.2E-04
chr4:3904136-3908068	3933	7.9	7.9	8.0	↓	-0.1	1.4E-04	4.1E-02
chr4:3982311-3984927	2617	7.3	7.2	7.4	↓	-0.2	1.3E-04	4.0E-02

chr4:3988004-3989065	1062	5.3	4.8	5.7	↓	-0.9	2.5E-10	5.9E-07
chr4:40027897-40028199	303	2.6	2.0	3.1	↓	-1.1	7.7E-06	4.4E-03
chr4:4006856-4007449	594	3.8	3.1	4.3	↓	-1.2	4.4E-13	2.5E-09
chr4:4116198-4116748	551	3.7	3.3	4.0	↓	-0.8	2.0E-05	8.9E-03
chr4:4136845-4137456	612	3.9	3.6	4.2	↓	-0.6	1.4E-04	4.1E-02
chr4:4178693-4178969	277	2.0	1.3	2.5	↓	-1.2	4.2E-04	8.6E-02
chr4:41959445-41959717	273	1.9	1.2	2.4	↓	-1.2	4.0E-04	8.3E-02
chr4:43106859-43107394	536	4.2	4.4	3.8	↑	0.6	1.3E-04	4.0E-02
chr4:43446358-43446684	327	2.4	1.8	2.8	↓	-1.0	4.9E-04	9.2E-02
chr4:43984014-43985330	1317	4.5	4.1	4.7	↓	-0.6	2.9E-04	6.6E-02
chr4:44109624-44110699	1076	4.5	4.2	4.9	↓	-0.7	1.4E-07	1.6E-04
chr4:4553362-4554384	1023	4.4	4.0	4.6	↓	-0.6	3.3E-05	1.3E-02
chr4:48230450-48230673	224	2.3	2.8	1.7	↑	1.0	3.8E-04	8.0E-02
chr4:4916705-4918049	1345	5.8	5.0	6.4	↓	-1.4	1.5E-22	2.2E-18
chr4:54077033-54077668	636	4.3	4.0	4.5	↓	-0.5	4.1E-04	8.5E-02
chr4:54191846-54192209	364	2.9	2.4	3.3	↓	-0.9	2.3E-04	5.8E-02
chr4:54695381-54695633	253	1.8	1.1	2.3	↓	-1.2	4.2E-04	8.6E-02
chr4:55631462-55631853	392	3.3	2.7	3.8	↓	-1.1	3.7E-07	3.4E-04
chr4:55634849-55635219	371	3.7	3.2	4.1	↓	-0.9	2.3E-07	2.4E-04
chr4:55636252-55636939	688	4.2	3.8	4.5	↓	-0.6	2.6E-05	1.1E-02
chr4:56202018-56202374	357	2.2	2.7	1.5	↑	1.2	4.9E-05	1.8E-02
chr4:5656427-5657058	632	3.4	3.0	3.7	↓	-0.7	4.3E-04	8.7E-02
chr4:5850056-5850563	508	3.9	3.2	4.4	↓	-1.3	2.3E-10	5.9E-07
chr4:5910533-5911491	959	4.8	4.3	5.1	↓	-0.8	4.7E-11	1.6E-07
chr4:59117799-59118218	420	3.1	2.6	3.4	↓	-0.8	5.2E-04	9.5E-02
chr4:5954276-5955343	1068	5.8	5.6	6.0	↓	-0.3	2.0E-05	8.9E-03
chr4:59605795-59606227	433	2.5	1.8	3.0	↓	-1.2	1.2E-05	6.2E-03
chr4:59846018-59846445	428	2.4	1.8	2.8	↓	-1.0	2.7E-04	6.4E-02
chr4:59848115-59848385	271	1.5	0.7	2.0	↓	-1.4	3.0E-04	6.8E-02
chr4:60575694-60575927	234	1.8	2.3	1.0	↑	1.3	3.0E-04	6.8E-02
chr4:60698462-60698735	274	2.3	1.7	2.8	↓	-1.1	1.6E-04	4.4E-02
chr4:6267641-6268417	777	5.0	4.5	5.3	↓	-0.8	5.9E-08	8.2E-05
chr4:63496739-63497064	326	2.7	2.0	3.1	↓	-1.1	2.6E-05	1.1E-02
chr4:63497078-63497388	311	2.6	1.6	3.2	↓	-1.5	1.2E-07	1.4E-04
chr4:63648163-63649572	1410	4.8	4.1	5.3	↓	-1.2	6.8E-16	6.1E-12
chr4:6375556-6376362	807	3.9	3.5	4.2	↓	-0.7	1.8E-05	8.4E-03
chr4:64879031-64879578	548	3.7	3.2	4.1	↓	-0.9	1.1E-07	1.4E-04
chr4:66857283-66857713	431	2.8	3.2	2.0	↑	1.2	5.8E-07	5.0E-04
chr4:67624029-67625201	1173	4.7	4.4	5.0	↓	-0.6	9.8E-06	5.4E-03
chr4:67626775-67627110	336	3.0	2.5	3.3	↓	-0.9	4.5E-04	8.9E-02
chr4:69287604-69287953	350	2.2	1.5	2.6	↓	-1.1	4.6E-04	9.0E-02
chr4:70694872-70695268	397	3.2	2.3	3.8	↓	-1.5	2.6E-11	1.1E-07
chr4:70695524-70697284	1761	4.7	4.0	5.1	↓	-1.1	1.3E-11	5.7E-08
chr4:70698105-70698706	602	3.6	3.0	4.0	↓	-1.1	1.2E-07	1.4E-04
chr4:70698752-70699187	436	3.4	2.7	3.9	↓	-1.1	1.1E-08	1.9E-05
chr4:70699195-70700490	1296	4.7	4.0	5.1	↓	-1.1	2.4E-15	1.8E-11
chr4:70700852-70701288	437	3.4	2.8	3.9	↓	-1.1	1.2E-06	8.9E-04

chr4:70704509-70704793	285	2.2	1.4	2.7	↓	-1.4	5.5E-06	3.4E-03
chr4:70812063-70812599	537	3.5	3.8	3.1	↑	0.8	4.5E-05	1.7E-02
chr4:70825633-70828172	2540	6.5	6.7	6.2	↑	0.5	1.4E-08	2.2E-05
chr4:70830764-70835175	4412	7.2	7.4	7.0	↑	0.4	5.1E-05	1.8E-02
chr4:70835246-70837021	1776	5.2	5.4	4.8	↑	0.6	2.9E-07	2.8E-04
chr4:7087516-7088031	516	4.4	4.1	4.7	↓	-0.6	3.7E-04	7.9E-02
chr4:71351988-71352331	344	2.8	2.2	3.3	↓	-1.1	1.2E-05	6.3E-03
chr4:71585376-71585697	322	3.2	3.5	2.8	↑	0.7	5.2E-04	9.5E-02
chr4:73019542-73019946	405	2.1	1.1	2.7	↓	-1.5	3.0E-06	2.0E-03
chr4:74866954-74867630	677	5.5	5.2	5.7	↓	-0.4	4.8E-04	9.2E-02
chr4:75792601-75793149	549	5.0	4.8	5.2	↓	-0.5	1.4E-04	4.1E-02
chr4:75885109-75885637	529	3.1	3.5	2.7	↑	0.7	5.5E-04	9.9E-02
chr4:76548896-76549481	586	3.3	2.7	3.7	↓	-1.0	1.2E-06	8.9E-04
chr4:7718687-7722434	3748	6.7	6.8	6.6	↑	0.2	3.6E-05	1.4E-02
chr4:77379435-77379848	414	3.0	3.4	2.6	↑	0.8	2.2E-04	5.6E-02
chr4:78103838-78104239	402	3.2	2.6	3.7	↓	-1.0	9.4E-05	3.1E-02
chr4:78131582-78132014	433	4.2	3.8	4.5	↓	-0.7	6.7E-06	4.0E-03
chr4:78935914-78936751	838	4.9	4.6	5.1	↓	-0.5	3.9E-05	1.5E-02
chr4:79075980-79076444	465	5.1	4.7	5.5	↓	-0.8	5.2E-09	9.6E-06
chr4:80727194-80727555	362	3.4	3.8	2.8	↑	1.0	4.5E-05	1.7E-02
chr4:80912218-80912543	326	2.1	1.3	2.6	↓	-1.3	9.8E-05	3.1E-02
chr4:80912938-80913304	367	2.3	2.8	1.7	↑	1.1	1.4E-04	4.1E-02
chr4:81345067-81345489	423	3.9	3.4	4.3	↓	-0.9	8.1E-07	6.4E-04
chr4:81532356-81532622	267	1.7	2.2	0.9	↑	1.3	4.3E-04	8.7E-02
chr4:82077266-82077619	354	2.4	1.8	2.8	↓	-1.0	3.7E-04	7.8E-02
chr4:82167575-82168381	807	3.6	3.2	4.0	↓	-0.8	7.9E-05	2.7E-02
chr4:83672857-83674218	1362	4.7	4.4	4.9	↓	-0.6	3.3E-05	1.3E-02
chr4:83975859-83977529	1671	4.9	4.6	5.1	↓	-0.5	3.2E-04	7.1E-02
chr4:84044525-84046652	2128	6.6	6.3	6.8	↓	-0.5	6.8E-07	5.6E-04
chr4:84388946-84389641	696	4.2	3.8	4.4	↓	-0.6	1.8E-05	8.5E-03
chr4:84624940-84625427	488	2.3	2.7	1.8	↑	1.0	4.9E-04	9.2E-02
chr4:84958451-84959227	777	4.6	4.3	4.8	↓	-0.5	1.0E-04	3.2E-02
chr4:85128059-85128653	595	5.0	4.6	5.3	↓	-0.7	5.4E-09	9.6E-06
chr4:8530082-8530390	309	3.2	2.7	3.5	↓	-0.8	7.7E-05	2.7E-02
chr4:85713057-85713408	352	2.4	1.7	2.9	↓	-1.2	4.1E-05	1.5E-02
chr4:86514918-86515439	522	3.9	3.4	4.3	↓	-0.9	6.9E-06	4.0E-03
chr4:8668324-8668895	572	4.2	3.7	4.6	↓	-0.9	9.1E-07	6.9E-04
chr4:8676834-8677395	562	3.4	3.0	3.8	↓	-0.8	7.9E-05	2.7E-02
chr4:8730537-8731054	518	3.5	2.8	3.9	↓	-1.1	7.1E-07	5.8E-04
chr4:8753002-8753838	837	4.4	4.0	4.6	↓	-0.6	1.4E-04	4.1E-02
chr4:88045615-88046472	858	4.7	4.4	4.9	↓	-0.5	1.4E-04	4.1E-02
chr4:88205987-88206545	559	3.0	2.5	3.3	↓	-0.9	4.9E-04	9.2E-02
chr4:88393860-88394241	382	3.5	2.7	4.0	↓	-1.3	2.4E-10	5.9E-07
chr4:8919231-8920634	1404	6.3	6.1	6.5	↓	-0.3	3.9E-04	8.1E-02
chr4:90315111-90315913	803	4.6	4.2	5.0	↓	-0.8	2.7E-08	4.1E-05
chr4:90482884-90483252	369	3.8	3.4	4.1	↓	-0.6	3.2E-04	7.1E-02
chr4:91400911-91401307	397	4.6	4.2	4.9	↓	-0.7	2.5E-04	6.1E-02

chr4:91484180-91485054	875	3.4	3.0	3.8	↓	-0.8	1.6E-04	4.5E-02
chr4:92304214-92304523	310	2.4	1.7	2.8	↓	-1.1	1.8E-04	4.8E-02
chr4:92736772-92737078	307	2.7	2.2	3.1	↓	-0.9	4.9E-04	9.2E-02
chr4:92982511-92982861	351	3.5	3.1	3.8	↓	-0.7	2.8E-04	6.6E-02
chr4:93363835-93364637	803	4.0	3.4	4.4	↓	-1.0	2.9E-07	2.8E-04
chr4:93717932-93720352	2421	6.3	6.5	6.1	↑	0.4	1.6E-04	4.5E-02
chr4:94507794-94508326	533	3.9	3.5	4.3	↓	-0.8	2.9E-04	6.6E-02
chr4:9696856-9697169	314	2.4	1.6	2.8	↓	-1.2	1.6E-05	7.9E-03
chr4:97937728-97938169	442	2.7	2.1	3.1	↓	-0.9	3.3E-04	7.3E-02
chr4:98325252-98325774	523	3.0	3.3	2.5	↑	0.8	1.3E-04	4.0E-02
chr5:100289379-100289731	353	3.2	2.9	3.5	↓	-0.7	6.3E-04	8.7E-02
chr5:10203741-10204530	790	5.3	4.8	5.6	↓	-0.8	5.9E-11	2.3E-07
chr5:102884436-102885562	1127	4.6	4.4	4.8	↓	-0.4	3.9E-04	6.6E-02
chr5:103119948-103120680	733	3.7	3.2	4.1	↓	-0.9	1.3E-06	8.5E-04
chr5:103152063-103152330	268	2.3	2.8	1.7	↑	1.0	1.9E-04	4.1E-02
chr5:103152464-103153287	824	3.3	3.7	2.7	↑	1.0	3.7E-06	2.1E-03
chr5:103255864-103256380	517	3.4	3.8	3.0	↑	0.7	2.9E-04	5.4E-02
chr5:1034106-1035916	1811	6.5	6.4	6.6	↓	-0.2	1.9E-04	4.2E-02
chr5:10343412-10343935	524	3.8	3.3	4.2	↓	-0.9	8.2E-08	1.0E-04
chr5:103518642-103520803	2162	6.1	5.9	6.2	↓	-0.3	1.0E-04	2.5E-02
chr5:103521547-103524491	2945	7.3	7.1	7.4	↓	-0.3	2.7E-06	1.7E-03
chr5:103524493-103525206	714	4.1	3.7	4.4	↓	-0.7	5.9E-05	1.8E-02
chr5:103526589-103528717	2129	6.2	6.0	6.4	↓	-0.3	1.1E-06	8.2E-04
chr5:103726898-103727672	775	3.8	3.4	4.1	↓	-0.8	8.2E-05	2.1E-02
chr5:10377641-10377947	307	2.0	2.5	1.2	↑	1.2	9.1E-05	2.3E-02
chr5:103786406-103786879	474	3.0	2.5	3.4	↓	-0.9	2.2E-04	4.5E-02
chr5:103797452-103797792	341	2.2	1.5	2.6	↓	-1.1	3.0E-04	5.5E-02
chr5:103813384-103813820	437	4.8	4.6	5.0	↓	-0.4	5.9E-04	8.3E-02
chr5:103902076-103902936	861	4.1	3.5	4.5	↓	-1.0	5.6E-08	7.8E-05
chr5:103904274-103904583	310	3.4	3.0	3.7	↓	-0.7	5.1E-04	7.7E-02
chr5:103945452-103945789	338	3.8	3.4	4.1	↓	-0.7	6.7E-05	1.9E-02
chr5:10409900-10410629	730	4.5	4.7	4.2	↑	0.5	7.0E-04	9.5E-02
chr5:104127896-104128411	516	4.2	3.9	4.4	↓	-0.5	2.1E-04	4.3E-02
chr5:105203082-105203453	372	2.9	2.1	3.4	↓	-1.3	1.3E-07	1.4E-04
chr5:105476904-105477519	616	3.3	3.6	2.9	↑	0.7	2.1E-04	4.5E-02
chr5:105525564-105525822	259	3.4	3.7	2.9	↑	0.8	3.2E-05	1.1E-02
chr5:105966228-105966671	444	4.2	3.8	4.5	↓	-0.7	4.1E-05	1.4E-02
chr5:107731571-107731819	249	2.1	2.6	1.4	↑	1.2	2.9E-04	5.4E-02
chr5:108167815-108168390	576	4.9	4.2	5.4	↓	-1.2	1.3E-12	7.6E-09
chr5:108310916-108311301	386	2.3	1.6	2.8	↓	-1.2	4.3E-05	1.4E-02
chr5:108355875-108356197	323	2.1	2.6	1.4	↑	1.2	2.0E-04	4.2E-02
chr5:108450255-108450556	302	2.2	1.7	2.7	↓	-1.0	4.9E-04	7.5E-02
chr5:108606294-108606489	196	1.7	1.0	2.2	↓	-1.3	5.3E-04	7.8E-02
chr5:110795670-110798411	2742	7.9	7.8	8.0	↓	-0.2	4.4E-04	7.2E-02
chr5:111170901-111172820	1920	6.5	6.3	6.6	↓	-0.3	2.9E-04	5.4E-02
chr5:111255129-111257282	2154	7.2	7.1	7.4	↓	-0.3	1.2E-06	8.5E-04
chr5:111357946-111358482	537	2.5	1.9	2.9	↓	-1.1	9.1E-05	2.3E-02

chr5:111399134-111401587	2454	6.6	6.5	6.7	↓	-0.2	3.1E-04	5.7E-02
chr5:111470356-111474184	3829	7.4	7.3	7.5	↓	-0.2	1.3E-05	6.0E-03
chr5:11614635-11615041	407	2.7	3.1	2.1	↑	1.0	3.2E-05	1.1E-02
chr5:12285971-12287776	1806	5.6	5.4	5.8	↓	-0.4	1.7E-04	3.8E-02
chr5:12397066-12397271	206	2.1	1.3	2.6	↓	-1.3	7.6E-05	2.0E-02
chr5:12773550-12774048	499	3.8	3.4	4.1	↓	-0.7	9.9E-05	2.4E-02
chr5:12796575-12796976	402	3.2	2.8	3.6	↓	-0.8	1.4E-04	3.2E-02
chr5:12822052-12822559	508	2.9	2.3	3.4	↓	-1.1	4.2E-05	1.4E-02
chr5:13596100-13596499	400	2.7	2.1	3.2	↓	-1.1	1.4E-05	6.2E-03
chr5:14223110-14223544	435	4.0	4.3	3.6	↑	0.7	2.8E-05	1.1E-02
chr5:14374565-14374909	345	2.9	2.3	3.4	↓	-1.1	1.3E-05	5.8E-03
chr5:14395082-14396156	1075	3.8	3.3	4.2	↓	-0.9	4.8E-06	2.7E-03
chr5:14829485-14829812	328	3.3	2.8	3.6	↓	-0.8	8.6E-05	2.2E-02
chr5:16381603-16382259	657	4.0	4.3	3.6	↑	0.7	3.8E-06	2.2E-03
chr5:17497673-17498698	1026	4.3	3.9	4.6	↓	-0.7	1.4E-06	9.3E-04
chr5:17775560-17777804	2245	6.7	6.9	6.4	↑	0.5	1.8E-05	7.5E-03
chr5:1785071-1789456	4386	7.9	7.8	8.0	↓	-0.3	1.6E-05	6.8E-03
chr5:17934432-17936617	2186	7.3	7.1	7.4	↓	-0.3	1.8E-04	3.9E-02
chr5:19657367-19657605	239	2.2	1.3	2.8	↓	-1.5	1.4E-06	9.4E-04
chr5:19681388-19681662	275	2.2	1.6	2.7	↓	-1.1	4.6E-04	7.4E-02
chr5:19693692-19693971	280	1.6	0.4	2.2	↓	-1.8	2.7E-06	1.7E-03
chr5:20194562-20195548	987	4.1	3.7	4.5	↓	-0.7	7.0E-05	1.9E-02
chr5:2025541-2026512	972	5.3	5.0	5.5	↓	-0.5	2.3E-06	1.4E-03
chr5:2043763-2047005	3243	6.8	6.7	7.0	↓	-0.2	6.0E-05	1.8E-02
chr5:20459216-20459640	425	2.3	2.8	1.7	↑	1.1	4.0E-04	6.7E-02
chr5:20732872-20733303	432	4.5	4.3	4.7	↓	-0.5	4.4E-04	7.2E-02
chr5:2240221-2240703	483	3.2	2.7	3.6	↓	-0.9	6.2E-04	8.7E-02
chr5:22922172-22923213	1042	3.6	3.0	3.9	↓	-0.9	3.1E-04	5.7E-02
chr5:24152571-24152956	386	2.9	2.1	3.5	↓	-1.4	1.0E-07	1.2E-04
chr5:24726142-24726811	670	4.3	4.6	3.9	↑	0.6	6.1E-05	1.8E-02
chr5:24732915-24734298	1384	5.1	4.6	5.4	↓	-0.8	7.9E-10	2.3E-06
chr5:2534308-2534707	400	4.4	4.1	4.6	↓	-0.5	1.4E-04	3.2E-02
chr5:25958598-25959162	565	4.3	4.7	3.9	↑	0.8	7.6E-06	3.9E-03
chr5:25959277-25960270	994	4.6	5.0	4.0	↑	0.9	2.0E-07	2.0E-04
chr5:25962662-25963986	1325	5.3	5.5	5.0	↑	0.5	6.8E-05	1.9E-02
chr5:25965924-25966217	294	2.5	3.0	1.7	↑	1.3	5.2E-06	2.9E-03
chr5:2617465-2618112	648	3.8	3.5	4.1	↓	-0.6	4.4E-04	7.2E-02
chr5:2623120-2626907	3788	8.0	7.9	8.1	↓	-0.1	7.4E-04	1.0E-01
chr5:26760984-26761255	272	2.3	2.7	1.6	↑	1.1	3.1E-04	5.7E-02
chr5:26897730-26898290	561	3.5	3.1	3.8	↓	-0.7	8.2E-05	2.1E-02
chr5:26929122-26930130	1009	3.9	4.1	3.5	↑	0.6	5.4E-05	1.7E-02
chr5:2794511-2796515	2005	5.7	5.5	5.9	↓	-0.4	1.5E-05	6.5E-03
chr5:2801467-2803233	1767	6.0	5.9	6.2	↓	-0.3	2.0E-04	4.2E-02
chr5:3066818-3067369	552	4.3	3.9	4.5	↓	-0.6	7.4E-05	2.0E-02
chr5:31268039-31268562	524	3.3	2.7	3.7	↓	-1.0	2.8E-06	1.7E-03
chr5:31818863-31819058	196	1.9	1.2	2.4	↓	-1.1	5.1E-04	7.7E-02
chr5:33136471-33137645	1175	5.2	4.9	5.5	↓	-0.7	1.0E-08	2.2E-05

chr5:33175988-33176279	292	3.6	3.1	3.9	↓	-0.7	6.5E-05	1.9E-02
chr5:33250969-33251913	945	4.5	4.2	4.7	↓	-0.5	5.0E-04	7.6E-02
chr5:34102883-34103421	539	3.4	3.7	2.9	↑	0.7	2.0E-04	4.2E-02
chr5:34327306-34328446	1141	4.9	4.6	5.1	↓	-0.4	2.1E-04	4.5E-02
chr5:3470414-3471694	1281	5.1	4.6	5.4	↓	-0.8	4.2E-08	6.8E-05
chr5:34833413-34834040	628	4.3	4.5	4.0	↑	0.5	4.8E-04	7.5E-02
chr5:35080180-35080650	471	3.8	3.4	4.2	↓	-0.7	1.1E-05	5.2E-03
chr5:3700954-3701529	576	3.9	3.5	4.2	↓	-0.7	2.2E-05	8.8E-03
chr5:37142527-37142931	405	4.7	4.4	4.9	↓	-0.4	4.5E-04	7.4E-02
chr5:37261027-37261424	398	3.1	3.4	2.6	↑	0.8	4.3E-04	7.1E-02
chr5:37261955-37262781	827	4.5	4.7	4.2	↑	0.5	1.1E-04	2.7E-02
chr5:39064054-39064244	191	1.5	0.5	2.1	↓	-1.6	4.2E-05	1.4E-02
chr5:39069088-39069686	599	2.8	2.1	3.3	↓	-1.2	5.5E-05	1.7E-02
chr5:4139989-4142150	2162	6.0	5.7	6.2	↓	-0.5	2.9E-07	2.6E-04
chr5:4160497-4160889	393	3.7	3.2	4.1	↓	-0.9	5.1E-07	4.2E-04
chr5:4175082-4175478	397	4.2	4.0	4.5	↓	-0.5	4.8E-04	7.5E-02
chr5:4360187-4360411	225	1.8	1.1	2.3	↓	-1.2	5.2E-04	7.7E-02
chr5:46235363-46235561	199	1.4	1.9	0.5	↑	1.4	4.6E-04	7.4E-02
chr5:47868241-47868496	256	2.0	1.3	2.4	↓	-1.1	5.8E-04	8.3E-02
chr5:48170956-48171295	340	2.6	1.9	3.0	↓	-1.1	2.2E-05	8.7E-03
chr5:49155259-49156321	1063	4.3	4.5	4.0	↑	0.6	2.2E-04	4.5E-02
chr5:49512591-49512991	401	3.0	3.4	2.6	↑	0.8	3.7E-04	6.4E-02
chr5:49837684-49838213	530	3.2	3.6	2.6	↑	0.9	3.1E-05	1.1E-02
chr5:49911412-49912006	595	2.6	3.1	2.0	↑	1.1	1.6E-04	3.6E-02
chr5:52889395-52889822	428	3.3	2.8	3.6	↓	-0.8	3.3E-04	5.9E-02
chr5:53429330-53429967	638	3.3	2.8	3.6	↓	-0.8	5.0E-05	1.7E-02
chr5:53755492-53756241	750	4.2	3.5	4.7	↓	-1.3	1.2E-14	1.0E-10
chr5:5379787-5380616	830	3.8	4.1	3.5	↑	0.6	5.6E-04	8.1E-02
chr5:5429096-5429783	688	4.9	5.2	4.7	↑	0.5	5.5E-05	1.7E-02
chr5:54685013-54685687	675	4.0	4.3	3.6	↑	0.7	6.4E-05	1.9E-02
chr5:55370278-55371591	1314	5.5	5.2	5.7	↓	-0.5	6.8E-04	9.3E-02
chr5:5572269-5573278	1010	5.9	5.7	6.1	↓	-0.4	5.8E-05	1.7E-02
chr5:5646535-5648423	1889	6.0	5.8	6.3	↓	-0.5	7.3E-08	9.7E-05
chr5:5651857-5652854	998	4.5	4.2	4.7	↓	-0.5	1.1E-04	2.7E-02
chr5:56627378-56628464	1087	4.7	4.4	5.0	↓	-0.6	1.1E-04	2.6E-02
chr5:56633421-56633736	316	2.9	2.4	3.3	↓	-0.9	4.9E-04	7.5E-02
chr5:56635662-56636013	352	2.8	2.3	3.1	↓	-0.8	5.2E-04	7.7E-02
chr5:56672413-56672800	388	1.9	1.1	2.3	↓	-1.2	4.3E-04	7.1E-02
chr5:56833497-56834027	531	4.2	3.8	4.5	↓	-0.8	6.5E-06	3.5E-03
chr5:56931454-56931892	439	2.9	2.5	3.3	↓	-0.8	3.5E-04	6.2E-02
chr5:57295725-57295983	259	1.9	2.3	1.2	↑	1.1	5.8E-04	8.3E-02
chr5:57849667-57849976	310	2.2	1.6	2.7	↓	-1.0	4.9E-04	7.5E-02
chr5:58568347-58568558	212	1.5	0.4	2.1	↓	-1.7	3.2E-05	1.1E-02
chr5:5928700-5930581	1882	5.5	5.3	5.8	↓	-0.4	7.5E-05	2.0E-02
chr5:5936663-5937750	1088	5.5	5.2	5.7	↓	-0.5	2.4E-05	9.4E-03
chr5:5957095-5957576	482	2.8	2.1	3.3	↓	-1.3	6.8E-06	3.5E-03
chr5:6265672-6266528	857	4.7	4.4	5.0	↓	-0.6	1.3E-05	6.0E-03

chr5:63017271-63017523	253	1.9	2.3	1.2	↑	1.2	4.8E-04	7.5E-02
chr5:63128654-63129082	429	3.4	2.9	3.7	↓	-0.8	4.2E-04	7.1E-02
chr5:63855870-63856189	320	2.7	3.1	2.2	↑	0.9	3.0E-04	5.5E-02
chr5:646962-647832	871	5.7	5.4	5.9	↓	-0.4	6.9E-05	1.9E-02
chr5:65477244-65478803	1560	5.4	5.6	5.3	↑	0.3	4.1E-04	6.8E-02
chr5:65497494-65497778	285	2.2	2.6	1.6	↑	1.0	4.7E-04	7.5E-02
chr5:65674575-65675162	588	2.4	1.7	2.8	↓	-1.1	2.5E-04	4.9E-02
chr5:66011129-66011736	608	4.9	4.6	5.1	↓	-0.5	4.9E-05	1.6E-02
chr5:66518310-66519897	1588	6.2	5.9	6.4	↓	-0.4	1.0E-04	2.5E-02
chr5:66836362-66837817	1456	5.2	5.0	5.3	↓	-0.4	5.3E-04	7.7E-02
chr5:67031504-67031837	334	2.3	2.7	1.7	↑	1.0	3.7E-04	6.5E-02
chr5:67342363-67342791	429	2.5	3.0	1.8	↑	1.2	9.8E-06	4.8E-03
chr5:67345272-67345890	619	3.2	2.8	3.5	↓	-0.8	7.1E-04	9.7E-02
chr5:67485177-67485419	243	2.3	2.7	1.7	↑	1.1	1.6E-04	3.6E-02
chr5:67514658-67515809	1152	5.5	5.7	5.2	↑	0.4	7.9E-05	2.1E-02
chr5:67525386-67525831	446	3.6	4.1	3.0	↑	1.0	2.5E-08	4.2E-05
chr5:67546092-67547686	1595	5.3	5.5	5.0	↑	0.6	2.4E-05	9.4E-03
chr5:67723591-67724034	444	3.3	2.4	3.8	↓	-1.3	6.9E-09	1.6E-05
chr5:67755881-67756210	330	3.3	2.9	3.6	↓	-0.7	3.4E-04	6.0E-02
chr5:67920261-67920666	406	2.2	2.6	1.5	↑	1.1	2.8E-04	5.4E-02
chr5:68055782-68056339	558	3.4	3.7	2.9	↑	0.9	4.0E-05	1.4E-02
chr5:6810944-6811768	825	4.5	4.2	4.8	↓	-0.5	5.5E-04	7.9E-02
chr5:68578309-68579189	881	4.7	4.3	5.0	↓	-0.8	1.6E-08	2.9E-05
chr5:68892726-68893711	986	4.9	4.6	5.1	↓	-0.5	3.6E-04	6.3E-02
chr5:68965577-68966817	1241	5.1	4.9	5.2	↓	-0.4	3.8E-04	6.6E-02
chr5:69084457-69085641	1185	4.6	4.2	4.9	↓	-0.7	2.8E-07	2.6E-04
chr5:69249753-69252303	2551	6.2	6.0	6.4	↓	-0.5	7.9E-08	1.0E-04
chr5:69292240-69294702	2463	6.6	6.5	6.7	↓	-0.3	1.2E-05	5.7E-03
chr5:69783157-69784096	940	4.9	4.6	5.0	↓	-0.4	7.4E-04	1.0E-01
chr5:69794919-69795829	911	4.5	4.1	4.8	↓	-0.7	5.3E-07	4.3E-04
chr5:69871255-69872182	928	4.7	4.4	5.0	↓	-0.7	1.4E-04	3.2E-02
chr5:69904039-69904637	599	5.4	4.6	5.9	↓	-1.3	1.5E-17	2.6E-13
chr5:70226625-70227442	818	4.4	4.1	4.7	↓	-0.6	1.8E-04	4.1E-02
chr5:7038162-7039359	1198	4.9	4.6	5.1	↓	-0.5	2.4E-04	4.9E-02
chr5:71341514-71343273	1760	5.2	5.0	5.5	↓	-0.5	3.0E-06	1.7E-03
chr5:71389946-71391675	1730	5.4	5.2	5.6	↓	-0.4	6.1E-04	8.6E-02
chr5:71391934-71395956	4023	7.5	7.3	7.6	↓	-0.3	2.1E-07	2.1E-04
chr5:71526475-71526995	521	3.3	2.7	3.6	↓	-0.9	2.9E-05	1.1E-02
chr5:72506950-72507719	770	3.8	3.5	4.1	↓	-0.6	2.5E-04	5.0E-02
chr5:74336386-74336935	550	3.1	3.4	2.6	↑	0.8	1.7E-04	3.8E-02
chr5:75865127-75866003	877	5.1	4.6	5.4	↓	-0.8	6.1E-10	1.9E-06
chr5:76054757-76055125	369	3.0	2.5	3.4	↓	-0.9	5.4E-05	1.7E-02
chr5:77429086-77429755	670	4.3	4.0	4.6	↓	-0.6	6.3E-05	1.8E-02
chr5:78455956-78456248	293	2.5	2.9	1.9	↑	0.9	5.0E-04	7.6E-02
chr5:78972820-78973169	350	2.2	1.5	2.7	↓	-1.2	5.7E-05	1.7E-02
chr5:7916913-7917812	900	5.0	5.2	4.8	↑	0.4	3.8E-04	6.6E-02
chr5:81313327-81313555	229	2.1	2.5	1.4	↑	1.1	4.7E-04	7.5E-02

chr5:81578607-81579567	961	4.1	3.8	4.4	↓	-0.6	2.4E-04	4.8E-02
chr5:83239951-83241886	1936	6.2	6.0	6.3	↓	-0.4	4.1E-07	3.5E-04
chr5:83534558-83534883	326	2.1	1.5	2.6	↓	-1.0	6.6E-04	9.1E-02
chr5:84464870-84465113	244	2.0	2.5	1.2	↑	1.3	9.4E-05	2.3E-02
chr5:84612590-84612881	292	3.2	2.5	3.6	↓	-1.1	2.3E-07	2.2E-04
chr5:8475874-8476292	419	2.8	1.9	3.3	↓	-1.4	1.2E-08	2.3E-05
chr5:85029291-85030098	808	5.6	5.4	5.8	↓	-0.5	5.7E-05	1.7E-02
chr5:85306513-85306844	332	3.0	2.5	3.4	↓	-0.9	2.2E-04	4.5E-02
chr5:85339895-85340320	426	3.0	3.3	2.5	↑	0.8	5.9E-04	8.3E-02
chr5:85773864-85774274	411	3.3	2.2	3.8	↓	-1.6	1.6E-12	7.8E-09
chr5:85776722-85777248	527	4.7	4.4	5.0	↓	-0.6	5.2E-04	7.7E-02
chr5:85783057-85783423	367	3.0	2.5	3.4	↓	-0.9	2.8E-04	5.3E-02
chr5:85784789-85785526	738	3.7	3.1	4.1	↓	-1.0	1.4E-07	1.5E-04
chr5:8581709-8582978	1270	5.1	4.7	5.4	↓	-0.7	7.0E-05	1.9E-02
chr5:86254289-86254652	364	2.2	2.7	1.6	↑	1.1	2.5E-04	4.9E-02
chr5:8681448-8681865	418	2.9	2.3	3.3	↓	-1.0	1.4E-05	6.3E-03
chr5:87230683-87231052	370	2.6	2.0	3.0	↓	-1.0	2.2E-04	4.5E-02
chr5:87243077-87243547	471	3.2	2.8	3.5	↓	-0.7	2.8E-04	5.4E-02
chr5:87426132-87426378	247	2.1	1.4	2.6	↓	-1.2	9.7E-05	2.4E-02
chr5:87542765-87543669	905	3.4	3.7	3.0	↑	0.7	5.1E-04	7.7E-02
chr5:88642086-88642388	303	2.1	1.5	2.6	↓	-1.1	2.0E-04	4.2E-02
chr5:8878793-8879408	616	4.7	4.3	5.0	↓	-0.7	9.6E-07	7.3E-04
chr5:88997388-88997957	570	3.4	2.7	3.8	↓	-1.1	9.1E-06	4.6E-03
chr5:89017511-89018165	655	5.1	4.8	5.4	↓	-0.6	3.1E-05	1.1E-02
chr5:89085459-89085823	365	3.9	3.4	4.3	↓	-0.9	1.1E-08	2.2E-05
chr5:89873312-89873605	294	2.7	2.2	3.1	↓	-1.0	8.9E-05	2.3E-02
chr5:90500155-90501094	940	4.7	4.3	5.0	↓	-0.7	4.4E-08	6.8E-05
chr5:90588712-90589093	382	3.0	2.5	3.4	↓	-0.8	2.6E-04	5.0E-02
chr5:91533612-91534391	780	4.3	4.0	4.6	↓	-0.6	7.0E-04	9.5E-02
chr5:91793239-91793445	207	1.7	0.9	2.3	↓	-1.4	5.3E-05	1.7E-02
chr5:91839178-91840965	1788	5.0	4.8	5.3	↓	-0.5	3.0E-04	5.6E-02
chr5:91847110-91848343	1234	5.0	5.3	4.8	↑	0.5	5.3E-05	1.7E-02
chr5:91857884-91858212	329	4.1	3.6	4.4	↓	-0.8	1.9E-05	7.9E-03
chr5:91874618-91875575	958	3.4	2.3	4.0	↓	-1.7	3.5E-16	4.0E-12
chr5:91875655-91876174	520	3.0	2.1	3.6	↓	-1.5	2.2E-09	5.8E-06
chr5:91879582-91880838	1257	4.5	4.1	4.9	↓	-0.8	6.0E-10	1.9E-06
chr5:91882335-91882584	250	1.7	0.9	2.3	↓	-1.4	6.9E-05	1.9E-02
chr5:91882744-91883657	914	3.5	3.1	3.8	↓	-0.7	5.2E-04	7.7E-02
chr5:91884210-91884570	361	2.2	1.1	2.8	↓	-1.6	2.4E-07	2.3E-04
chr5:91930121-91930356	236	2.1	1.5	2.5	↓	-1.0	5.4E-04	7.9E-02
chr5:91965750-91966340	591	3.4	3.0	3.8	↓	-0.7	3.1E-04	5.7E-02
chr5:91997778-91998515	738	3.7	3.1	4.1	↓	-1.0	4.6E-08	6.8E-05
chr5:92002920-92003386	467	2.9	2.4	3.2	↓	-0.8	4.9E-04	7.5E-02
chr5:92005949-92006567	619	3.6	2.8	4.1	↓	-1.2	1.2E-07	1.3E-04
chr5:92008026-92008660	635	4.0	3.6	4.3	↓	-0.7	2.2E-04	4.5E-02
chr5:92008947-92009394	448	3.2	2.6	3.6	↓	-1.0	1.1E-06	7.7E-04
chr5:92009607-92010952	1346	5.7	5.2	6.1	↓	-0.8	4.9E-22	1.7E-17

chr5:92011771-92013069	1299	5.7	5.5	5.8	↓	-0.4	9.6E-05	2.4E-02
chr5:92013130-92013329	200	1.6	0.4	2.2	↓	-1.7	6.6E-06	3.5E-03
chr5:92013455-92013830	376	2.8	2.3	3.2	↓	-0.9	1.2E-04	2.8E-02
chr5:92013968-92014283	316	1.7	0.7	2.3	↓	-1.7	6.3E-06	3.4E-03
chr5:92014613-92014899	287	2.0	0.6	2.6	↓	-2.0	5.0E-09	1.2E-05
chr5:92015409-92015668	260	1.7	0.5	2.3	↓	-1.8	6.3E-07	4.9E-04
chr5:92032722-92033158	437	3.6	3.2	3.9	↓	-0.6	4.9E-04	7.5E-02
chr5:92066439-92066999	561	2.7	2.1	3.1	↓	-1.0	6.7E-05	1.9E-02
chr5:92304732-92305274	543	3.7	3.2	4.1	↓	-0.9	3.1E-07	2.7E-04
chr5:93362950-93364078	1129	5.6	5.4	5.8	↓	-0.4	1.5E-05	6.5E-03
chr5:93924402-93925057	656	4.6	4.2	5.0	↓	-0.7	1.5E-06	9.6E-04
chr5:93925370-93925891	522	4.0	3.6	4.3	↓	-0.7	3.0E-05	1.1E-02
chr5:94042971-94044563	1593	5.3	4.9	5.7	↓	-0.8	2.5E-12	1.1E-08
chr5:94044654-94046940	2287	5.9	6.2	5.6	↑	0.6	4.6E-05	1.5E-02
chr5:94304568-94309659	5092	7.1	7.3	7.0	↑	0.3	1.1E-05	5.2E-03
chr5:94314227-94314576	350	3.0	3.3	2.5	↑	0.8	5.4E-04	7.8E-02
chr5:94692509-94693605	1097	6.6	6.7	6.5	↑	0.2	6.6E-04	9.1E-02
chr5:95030829-95031501	673	4.2	4.5	3.9	↑	0.5	3.9E-04	6.6E-02
chr5:97162949-97163234	286	2.6	1.9	3.1	↓	-1.1	8.6E-05	2.2E-02
chr5:97164894-97165489	596	3.7	2.9	4.2	↓	-1.4	6.6E-13	4.5E-09
chr5:97168762-97168989	228	1.9	1.0	2.5	↓	-1.5	9.5E-06	4.7E-03
chr5:97223367-97223765	399	3.2	2.8	3.5	↓	-0.7	4.0E-04	6.8E-02
chr5:97227759-97228223	465	5.0	4.8	5.3	↓	-0.5	1.0E-06	7.7E-04
chr5:97839155-97839431	277	2.4	1.5	2.9	↓	-1.4	5.2E-07	4.2E-04
chr5:98096171-98096701	531	4.1	3.8	4.4	↓	-0.5	6.6E-04	9.1E-02
chr5:98098249-98099596	1348	5.8	5.6	5.9	↓	-0.4	3.3E-04	5.9E-02
chr5:98797113-98797657	545	4.9	4.6	5.1	↓	-0.5	2.5E-04	5.0E-02
chr5:9943538-9943861	324	2.8	3.3	2.1	↑	1.3	5.7E-08	7.8E-05
chr5:99937810-99938353	544	5.5	5.2	5.7	↓	-0.4	1.6E-05	6.7E-03
chr5:99938816-99939116	301	2.8	3.1	2.3	↑	0.9	4.8E-04	7.5E-02
chr6:100551334-100551616	283	3.2	2.6	3.6	↓	-1.0	3.8E-04	7.1E-02
chr6:100694754-100695224	471	5.1	5.4	4.8	↑	0.6	4.2E-09	1.0E-05
chr6:101315096-101316555	1460	5.7	5.5	5.9	↓	-0.4	2.4E-06	1.7E-03
chr6:102222047-102222668	622	3.6	3.1	4.0	↓	-0.8	6.2E-06	3.6E-03
chr6:102719500-102719726	227	1.9	1.2	2.4	↓	-1.2	1.8E-04	4.4E-02
chr6:102749383-102749747	365	3.6	3.2	3.9	↓	-0.7	2.3E-04	5.1E-02
chr6:103551958-103552443	486	2.7	2.0	3.1	↓	-1.1	5.4E-05	1.9E-02
chr6:104269346-104270157	812	4.8	5.0	4.5	↑	0.5	6.2E-04	9.3E-02
chr6:105653577-105653882	306	2.8	1.9	3.3	↓	-1.4	9.0E-08	1.5E-04
chr6:108114365-108114734	370	4.1	3.6	4.4	↓	-0.8	2.8E-05	1.1E-02
chr6:108191833-108192024	192	2.2	1.6	2.6	↓	-1.0	5.5E-04	8.7E-02
chr6:108496020-108496228	209	1.6	2.1	0.8	↑	1.3	4.8E-04	8.2E-02
chr6:109113246-109113738	493	3.1	2.4	3.6	↓	-1.2	9.2E-06	5.0E-03
chr6:110735316-110735623	308	4.0	4.2	3.7	↑	0.6	6.8E-04	9.9E-02
chr6:1108513-1109271	759	3.8	3.4	4.2	↓	-0.8	1.9E-05	8.6E-03
chr6:1109369-1109682	314	2.2	1.5	2.7	↓	-1.1	2.3E-04	5.2E-02
chr6:111003007-111003289	283	2.9	3.3	2.2	↑	1.1	7.6E-05	2.4E-02

chr6:111444503-111444771	269	2.4	1.7	2.8	↓	-1.1	7.5E-05	2.4E-02
chr6:111934347-111934561	215	1.7	2.3	0.9	↑	1.3	1.5E-04	3.8E-02
chr6:111978138-111978465	328	3.0	3.4	2.5	↑	0.9	1.7E-04	4.2E-02
chr6:111978814-111979069	256	2.9	3.3	2.5	↑	0.8	6.2E-04	9.3E-02
chr6:112566293-112566818	526	2.8	3.2	2.3	↑	0.9	1.9E-04	4.6E-02
chr6:112866400-112866721	322	2.8	2.2	3.2	↓	-1.0	4.9E-05	1.8E-02
chr6:112927108-112927610	503	3.1	3.4	2.6	↑	0.8	4.1E-04	7.4E-02
chr6:113143329-113143629	301	2.9	3.2	2.5	↑	0.8	5.2E-04	8.5E-02
chr6:113166722-113167024	303	3.1	3.5	2.6	↑	1.0	1.1E-05	5.7E-03
chr6:114052644-114052758	115	1.4	0.6	2.0	↓	-1.3	6.1E-04	9.2E-02
chr6:114191373-114191666	294	2.9	3.3	2.4	↑	0.8	2.0E-04	4.7E-02
chr6:117564352-117564608	257	3.4	2.9	3.8	↓	-0.9	6.4E-04	9.5E-02
chr6:119439937-119440632	696	4.2	3.7	4.5	↓	-0.8	1.4E-07	1.9E-04
chr6:120017532-120017941	410	2.8	3.3	2.2	↑	1.0	5.4E-05	1.9E-02
chr6:120341640-120341955	316	2.4	1.4	3.0	↓	-1.6	1.0E-07	1.5E-04
chr6:120398966-120399494	529	4.3	4.7	3.9	↑	0.7	9.9E-08	1.5E-04
chr6:1204951-1208127	3177	7.8	7.7	7.9	↓	-0.1	3.2E-04	6.4E-02
chr6:1208133-1209361	1229	5.2	5.3	5.0	↑	0.4	6.7E-04	9.8E-02
chr6:12089814-12090611	798	4.8	4.6	5.1	↓	-0.5	1.7E-04	4.2E-02
chr6:12199220-12199602	383	2.9	2.4	3.3	↓	-1.0	1.8E-05	8.3E-03
chr6:12252251-12253273	1023	5.8	5.4	6.1	↓	-0.7	1.1E-07	1.5E-04
chr6:12446486-12446935	450	3.7	3.3	4.0	↓	-0.7	4.6E-04	7.9E-02
chr6:124717243-124717508	266	2.5	1.8	2.9	↓	-1.1	1.1E-04	3.1E-02
chr6:125836944-125837137	194	2.0	2.4	1.4	↑	1.1	5.6E-04	8.8E-02
chr6:126697917-126698711	795	3.6	3.2	3.9	↓	-0.8	8.6E-05	2.7E-02
chr6:135380353-135380586	234	2.3	1.5	2.8	↓	-1.3	6.2E-05	2.1E-02
chr6:135400057-135400306	250	2.1	1.4	2.6	↓	-1.2	1.9E-04	4.5E-02
chr6:135404583-135404899	317	2.9	1.8	3.5	↓	-1.7	2.3E-11	1.6E-07
chr6:135410825-135411086	262	2.6	2.0	2.9	↓	-0.9	2.2E-04	5.0E-02
chr6:135412367-135412559	193	2.0	1.3	2.5	↓	-1.3	4.7E-05	1.7E-02
chr6:135412887-135413285	399	2.9	1.9	3.4	↓	-1.5	3.5E-09	9.1E-06
chr6:135416367-135416692	326	2.6	2.0	3.0	↓	-1.0	4.3E-05	1.6E-02
chr6:135416730-135418053	1324	4.4	3.9	4.8	↓	-0.9	4.7E-06	2.9E-03
chr6:135418227-135418906	680	3.6	2.9	4.1	↓	-1.2	6.0E-08	1.0E-04
chr6:135433233-135433956	724	5.5	5.2	5.7	↓	-0.5	3.8E-04	7.1E-02
chr6:135473511-135474144	634	3.8	3.4	4.1	↓	-0.7	9.6E-05	2.9E-02
chr6:135547183-135547645	463	3.2	2.4	3.7	↓	-1.3	2.0E-09	6.2E-06
chr6:135550029-135550319	291	2.4	1.6	2.9	↓	-1.3	4.7E-06	2.9E-03
chr6:135559082-135559653	572	3.2	2.5	3.6	↓	-1.1	2.5E-06	1.8E-03
chr6:1356143-1356942	800	4.5	4.0	4.9	↓	-0.9	2.1E-06	1.5E-03
chr6:136306396-136307000	605	3.1	2.6	3.5	↓	-0.9	1.1E-04	3.0E-02
chr6:136332798-136333349	552	2.9	2.3	3.3	↓	-1.1	1.1E-05	5.7E-03
chr6:136892905-136893578	674	3.8	4.1	3.4	↑	0.7	2.6E-04	5.7E-02
chr6:136908348-136908642	295	2.8	3.2	2.1	↑	1.1	3.0E-06	2.1E-03
chr6:137057775-137058284	510	4.1	4.3	3.8	↑	0.6	3.5E-04	6.8E-02
chr6:137078311-137079071	761	4.3	4.6	3.8	↑	0.8	8.7E-08	1.4E-04
chr6:137080038-137080668	631	3.7	4.0	3.3	↑	0.7	6.6E-05	2.2E-02

chr6:138245625-138245860	236	1.8	1.0	2.2	↓	-1.2	4.8E-04	8.2E-02
chr6:138684760-138685208	449	4.4	4.0	4.7	↓	-0.7	1.6E-04	4.0E-02
chr6:139028671-139029063	393	4.0	3.5	4.3	↓	-0.8	6.9E-06	3.9E-03
chr6:139074042-139074201	160	1.8	1.0	2.3	↓	-1.3	1.4E-04	3.7E-02
chr6:139114191-139114550	360	2.5	2.0	2.9	↓	-1.0	1.3E-04	3.5E-02
chr6:139648214-139648810	597	5.1	4.8	5.4	↓	-0.5	4.7E-04	8.0E-02
chr6:139746205-139746732	528	3.3	2.7	3.7	↓	-0.9	5.5E-06	3.3E-03
chr6:139763679-139764057	379	3.4	3.1	3.7	↓	-0.7	3.4E-04	6.6E-02
chr6:140537640-140538259	620	3.5	3.1	3.8	↓	-0.8	5.6E-05	1.9E-02
chr6:140969655-140970160	506	4.2	3.9	4.5	↓	-0.6	3.8E-05	1.5E-02
chr6:141416499-141417081	583	3.2	3.5	2.8	↑	0.7	4.5E-04	7.9E-02
chr6:14152995-14153421	427	5.1	4.9	5.3	↓	-0.4	4.3E-04	7.6E-02
chr6:14186420-14186961	542	3.0	3.4	2.6	↑	0.7	4.5E-04	7.8E-02
chr6:142100661-142100958	298	2.2	1.5	2.6	↓	-1.1	4.0E-04	7.2E-02
chr6:142956963-142957853	891	5.2	4.9	5.5	↓	-0.6	9.7E-06	5.2E-03
chr6:143087694-143088117	424	2.8	2.2	3.2	↓	-0.9	1.4E-04	3.6E-02
chr6:143378903-143379246	344	6.2	6.1	6.4	↓	-0.3	1.3E-04	3.5E-02
chr6:143893957-143894369	413	3.9	3.4	4.2	↓	-0.9	8.2E-07	7.7E-04
chr6:144073825-144074663	839	4.4	4.0	4.8	↓	-0.8	2.9E-08	6.3E-05
chr6:145165428-145165947	520	3.1	2.6	3.5	↓	-0.9	1.8E-05	8.2E-03
chr6:146292781-146293925	1145	5.6	5.3	5.9	↓	-0.6	3.9E-07	4.2E-04
chr6:146385503-146386183	681	4.1	3.8	4.4	↓	-0.7	4.5E-05	1.7E-02
chr6:146477925-146478292	368	2.3	2.7	1.7	↑	1.0	5.8E-04	9.0E-02
chr6:146890284-146890767	484	3.7	3.3	4.0	↓	-0.7	6.0E-05	2.0E-02
chr6:146904499-146905092	594	4.5	4.1	4.8	↓	-0.7	1.9E-07	2.4E-04
chr6:146910451-146913532	3082	6.8	6.6	6.9	↓	-0.3	2.6E-05	1.1E-02
chr6:147004716-147004954	239	2.8	2.1	3.2	↓	-1.1	3.8E-05	1.4E-02
chr6:148177730-148177959	230	2.4	2.8	1.6	↑	1.2	1.7E-05	7.9E-03
chr6:148280135-148280325	191	1.8	1.0	2.3	↓	-1.4	2.7E-05	1.1E-02
chr6:148280488-148281822	1335	4.9	4.4	5.2	↓	-0.9	1.5E-07	1.9E-04
chr6:148519102-148519667	566	4.4	4.1	4.6	↓	-0.6	6.5E-04	9.7E-02
chr6:148754851-148755124	274	1.7	2.2	1.0	↑	1.3	2.2E-04	5.0E-02
chr6:150186524-150186945	422	2.3	2.7	1.7	↑	1.0	3.6E-04	6.8E-02
chr6:150501500-150502201	702	5.0	4.8	5.2	↓	-0.4	3.7E-04	7.1E-02
chr6:150577916-150579684	1769	6.4	6.3	6.5	↓	-0.2	5.0E-04	8.3E-02
chr6:150661151-150661562	412	4.2	3.9	4.4	↓	-0.5	6.6E-04	9.7E-02
chr6:151133539-151134808	1270	4.8	4.4	5.1	↓	-0.7	1.0E-06	8.7E-04
chr6:15115653-15116230	578	4.2	4.5	3.8	↑	0.7	1.7E-05	8.0E-03
chr6:15131321-15132030	710	3.9	3.4	4.3	↓	-0.9	5.5E-07	5.6E-04
chr6:151321565-151321989	425	4.8	4.4	5.1	↓	-0.6	2.7E-05	1.1E-02
chr6:15132727-15133286	560	3.9	4.1	3.5	↑	0.6	4.6E-04	8.0E-02
chr6:151351711-151353606	1896	6.0	5.8	6.2	↓	-0.4	3.8E-04	7.1E-02
chr6:151437303-151438735	1433	5.4	5.7	5.0	↑	0.7	3.6E-06	2.3E-03
chr6:15147074-15149712	2639	7.1	7.2	7.0	↑	0.2	5.9E-04	9.1E-02
chr6:151589065-151589392	328	2.8	2.3	3.2	↓	-0.8	3.9E-04	7.2E-02
chr6:151653315-151654186	872	4.8	5.1	4.4	↑	0.7	8.7E-05	2.7E-02
chr6:151654554-151654886	333	2.3	2.8	1.6	↑	1.2	2.9E-05	1.2E-02

chr6:151655423-151656099	677	5.0	5.2	4.7	↑	0.4		3.9E-04	7.2E-02
chr6:151657699-151658053	355	4.1	4.5	3.5	↑	1.0		1.2E-05	5.8E-03
chr6:151658151-151659100	950	4.1	4.5	3.5	↑	1.0		1.1E-05	5.7E-03
chr6:151660385-151664641	4257	7.4	7.6	7.2	↑	0.4		3.4E-04	6.7E-02
chr6:151667993-151669913	1921	6.8	7.1	6.5	↑	0.6		1.0E-07	1.5E-04
chr6:152660235-152660480	246	2.7	3.1	2.2	↑	0.9		5.0E-04	8.3E-02
chr6:15302132-15302454	323	2.6	3.0	2.0	↑	1.0		4.0E-04	7.3E-02
chr6:154263582-154265991	2410	7.2	7.0	7.4	↓	-0.4		5.6E-09	1.3E-05
chr6:154338460-154340372	1913	7.4	7.2	7.5	↓	-0.3		3.2E-06	2.1E-03
chr6:154352107-154356434	4328	8.1	8.0	8.2	↓	-0.2		8.2E-06	4.6E-03
chr6:154357838-154359046	1209	6.1	5.9	6.3	↓	-0.3		9.5E-05	2.9E-02
chr6:154857144-154857836	693	4.5	4.1	4.8	↓	-0.7		2.8E-07	3.2E-04
chr6:154870650-154870871	222	2.7	2.2	3.1	↓	-0.9		3.9E-04	7.2E-02
chr6:154954902-154956297	1396	6.9	6.8	7.0	↓	-0.3		5.0E-07	5.3E-04
chr6:155085996-155087645	1650	7.6	7.5	7.8	↓	-0.3		6.7E-05	2.2E-02
chr6:155479786-155480910	1125	5.5	5.3	5.8	↓	-0.5		1.2E-04	3.4E-02
chr6:156128387-156128905	519	4.5	4.2	4.7	↓	-0.5		5.2E-04	8.5E-02
chr6:156176559-156177044	486	3.9	3.2	4.3	↓	-1.1		5.6E-10	2.4E-06
chr6:156197277-156197579	303	2.1	1.4	2.6	↓	-1.2		1.0E-04	3.0E-02
chr6:156225004-156225511	508	3.2	3.6	2.8	↑	0.8		7.1E-05	2.3E-02
chr6:156572035-156572315	281	2.4	1.3	3.1	↓	-1.8		2.9E-10	1.4E-06
chr6:156667338-156669423	2086	7.2	7.0	7.3	↓	-0.3		1.4E-09	4.6E-06
chr6:156734389-156735769	1381	6.1	5.9	6.2	↓	-0.3		1.7E-05	7.9E-03
chr6:156881279-156883105	1827	7.1	6.9	7.2	↓	-0.4		4.1E-10	1.9E-06
chr6:156887452-156890604	3153	6.7	6.6	6.8	↓	-0.2		5.8E-04	9.0E-02
chr6:157131721-157131982	262	2.9	2.2	3.4	↓	-1.1		9.7E-07	8.4E-04
chr6:157210253-157210866	614	5.3	5.0	5.5	↓	-0.6		3.0E-04	6.0E-02
chr6:157211181-157211533	353	3.0	2.3	3.5	↓	-1.1		4.4E-07	4.7E-04
chr6:157296516-157296858	343	2.5	1.8	3.0	↓	-1.2		1.6E-05	7.5E-03
chr6:157370882-157371131	250	2.9	2.3	3.3	↓	-1.0		2.3E-05	9.8E-03
chr6:15903632-15903813	182	1.5	0.6	2.0	↓	-1.4		2.3E-04	5.1E-02
chr6:16250374-16250829	456	3.7	3.3	4.0	↓	-0.8		2.0E-05	8.8E-03
chr6:1640575-1641288	714	5.1	4.7	5.5	↓	-0.8		6.6E-09	1.5E-05
chr6:16929383-16929650	268	4.1	3.4	4.5	↓	-1.2		8.2E-14	1.3E-09
chr6:169610-172119	2510	11.2	11.1	11.2	↓	-0.1		1.8E-04	4.4E-02
chr6:17581095-17582361	1267	6.1	5.9	6.3	↓	-0.3		7.8E-05	2.5E-02
chr6:1770483-1779798	9316	9.5	9.5	9.6	↓	-0.1		2.9E-04	6.0E-02
chr6:17818729-17819987	1259	5.5	5.7	5.3	↑	0.4		9.3E-05	2.8E-02
chr6:17945073-17946651	1579	5.0	5.2	4.8	↑	0.4		5.0E-04	8.3E-02
chr6:1869154-1870450	1297	5.5	5.3	5.6	↓	-0.3		5.6E-04	8.8E-02
chr6:18990042-18990328	287	2.1	1.4	2.6	↓	-1.2		7.0E-05	2.3E-02
chr6:1916085-1917188	1104	4.6	4.2	4.9	↓	-0.7		1.4E-04	3.6E-02
chr6:2160161-2160650	490	4.1	4.4	3.7	↑	0.6		1.1E-04	3.1E-02
chr6:22817650-22817845	196	1.8	1.1	2.3	↓	-1.3		1.9E-04	4.5E-02
chr6:22888874-22889044	171	2.0	2.5	1.2	↑	1.3		5.1E-05	1.8E-02
chr6:2368755-2368990	236	2.2	1.6	2.6	↓	-1.0		5.4E-04	8.6E-02
chr6:23975190-23975542	353	4.5	4.1	4.9	↓	-0.8		2.8E-09	7.9E-06

chr6:24004967-24005266	300	3.9	3.4	4.3	↓	-0.9	4.8E-06	3.0E-03
chr6:24032315-24032956	642	3.6	3.2	3.9	↓	-0.7	2.0E-04	4.7E-02
chr6:24539524-24540152	629	3.8	4.1	3.5	↑	0.6	5.7E-04	8.9E-02
chr6:2505385-2507665	2281	7.3	7.4	7.1	↑	0.2	5.3E-05	1.9E-02
chr6:25130862-25131849	988	5.4	5.0	5.7	↓	-0.7	2.1E-07	2.6E-04
chr6:25360057-25360845	789	4.4	4.0	4.8	↓	-0.7	1.8E-06	1.4E-03
chr6:25361219-25362091	873	4.0	3.7	4.3	↓	-0.6	3.8E-04	7.1E-02
chr6:25362864-25365092	2229	6.7	6.2	7.0	↓	-0.9	8.1E-12	6.3E-08
chr6:25365216-25365886	671	4.3	3.9	4.6	↓	-0.7	3.3E-06	2.2E-03
chr6:25368610-25369213	604	3.8	3.4	4.2	↓	-0.8	9.1E-06	5.0E-03
chr6:25493438-25495038	1601	5.6	5.4	5.8	↓	-0.4	1.8E-04	4.4E-02
chr6:2748523-2750332	1810	6.6	6.8	6.4	↑	0.3	2.4E-04	5.3E-02
chr6:27545380-27546078	699	4.7	4.4	5.0	↓	-0.6	7.7E-06	4.4E-03
chr6:27789250-27789519	270	2.7	2.1	3.1	↓	-1.0	1.5E-04	3.9E-02
chr6:2794516-2799013	4498	8.1	8.0	8.2	↓	-0.2	3.3E-04	6.4E-02
chr6:28103512-28104102	591	3.4	3.0	3.7	↓	-0.7	2.7E-04	5.8E-02
chr6:28105518-28105928	411	4.1	3.6	4.4	↓	-0.8	2.1E-05	9.2E-03
chr6:28204834-28205490	657	4.2	3.7	4.5	↓	-0.8	4.0E-09	1.0E-05
chr6:28249089-28249525	437	4.0	3.6	4.3	↓	-0.7	5.9E-05	2.0E-02
chr6:28256200-28256649	450	3.2	2.7	3.6	↓	-1.0	1.9E-05	8.6E-03
chr6:28400215-28400567	353	2.2	1.5	2.6	↓	-1.1	2.4E-04	5.2E-02
chr6:2905663-2908396	2734	7.4	7.2	7.5	↓	-0.3	9.8E-07	8.4E-04
chr6:2914473-2915506	1034	6.3	6.1	6.5	↓	-0.4	1.0E-07	1.5E-04
chr6:29327706-29327867	162	1.9	2.3	1.2	↑	1.1	5.3E-04	8.6E-02
chr6:29712669-29713077	409	2.9	2.4	3.3	↓	-0.9	2.3E-04	5.1E-02
chr6:3000570-3001792	1223	6.6	5.9	7.1	↓	-1.2	5.1E-17	1.1E-12
chr6:3003642-3003956	315	2.9	2.4	3.3	↓	-0.9	1.0E-04	2.9E-02
chr6:30053708-30054200	493	4.3	3.7	4.7	↓	-0.9	1.1E-05	5.7E-03
chr6:30132296-30133428	1133	5.6	5.4	5.8	↓	-0.4	4.4E-05	1.7E-02
chr6:30258054-30258588	535	5.0	4.7	5.3	↓	-0.5	1.3E-04	3.5E-02
chr6:30360187-30360613	427	2.9	2.1	3.4	↓	-1.2	8.9E-07	8.2E-04
chr6:30495184-30495816	633	3.6	2.8	4.1	↓	-1.4	1.3E-10	7.0E-07
chr6:30497477-30497968	492	3.7	3.1	4.2	↓	-1.0	4.0E-08	8.1E-05
chr6:30903002-30903310	309	2.6	3.0	2.1	↑	1.0	3.0E-04	6.1E-02
chr6:30911646-30914031	2386	6.9	7.0	6.7	↑	0.2	3.4E-04	6.7E-02
chr6:3094715-3095272	558	4.2	3.8	4.4	↓	-0.6	4.2E-04	7.5E-02
chr6:3095306-3096482	1177	5.4	4.7	5.9	↓	-1.3	7.6E-28	4.8E-23
chr6:3097705-3098677	973	4.6	4.0	5.0	↓	-1.1	5.7E-13	6.0E-09
chr6:31024260-31024524	265	2.0	2.5	1.3	↑	1.2	1.4E-04	3.6E-02
chr6:31024568-31025285	718	4.4	4.1	4.6	↓	-0.6	3.8E-04	7.1E-02
chr6:31027948-31029108	1161	4.9	5.2	4.6	↑	0.5	5.4E-05	1.9E-02
chr6:3164545-3165235	691	4.9	4.6	5.1	↓	-0.4	3.3E-04	6.6E-02
chr6:3177817-3179082	1266	5.9	5.7	6.1	↓	-0.4	5.0E-04	8.4E-02
chr6:3179098-3180777	1680	6.5	6.4	6.6	↓	-0.2	3.3E-04	6.4E-02
chr6:3181558-3182781	1224	4.6	4.1	5.0	↓	-0.9	5.8E-08	1.0E-04
chr6:31916116-31916409	294	3.0	3.4	2.4	↑	1.0	1.2E-04	3.2E-02
chr6:3201687-3202135	449	3.1	2.5	3.5	↓	-1.0	8.2E-06	4.6E-03

chr6:3204338-3205532	1195	5.6	5.4	5.8	↓	-0.4	1.1E-04	3.0E-02
chr6:3209607-3210217	611	3.7	3.2	4.1	↓	-0.9	1.2E-05	6.1E-03
chr6:3210532-3211126	595	4.4	4.0	4.7	↓	-0.6	1.9E-05	8.6E-03
chr6:3223241-3225229	1989	6.9	6.7	7.1	↓	-0.4	1.9E-06	1.4E-03
chr6:3234724-3235253	530	5.2	5.0	5.5	↓	-0.5	6.8E-04	9.9E-02
chr6:32485618-32486822	1205	4.8	5.0	4.6	↑	0.4	5.1E-04	8.4E-02
chr6:32791151-32791551	401	4.3	4.0	4.5	↓	-0.6	1.3E-04	3.5E-02
chr6:32864894-32865174	281	2.5	1.6	3.0	↓	-1.4	2.7E-07	3.1E-04
chr6:32915247-32915455	209	2.5	2.9	2.0	↑	0.9	4.3E-04	7.7E-02
chr6:32948908-32949269	362	2.8	3.1	2.3	↑	0.9	3.2E-04	6.4E-02
chr6:3312665-3314021	1357	5.9	5.7	6.1	↓	-0.4	5.1E-05	1.8E-02
chr6:33224018-33224143	126	1.7	2.2	1.0	↑	1.2	6.8E-04	9.9E-02
chr6:33260940-33261274	335	2.8	2.3	3.2	↓	-0.9	5.1E-04	8.4E-02
chr6:33885039-33887655	2617	6.9	6.8	7.0	↓	-0.2	3.2E-04	6.3E-02
chr6:33920472-33921082	611	8.1	7.9	8.2	↓	-0.4	4.7E-07	5.0E-04
chr6:339838-340259	422	4.0	4.3	3.8	↑	0.5	6.7E-04	9.8E-02
chr6:34522761-34523109	349	3.4	3.7	3.0	↑	0.7	4.5E-04	7.9E-02
chr6:3488108-3488746	639	3.4	2.9	3.7	↓	-0.7	1.8E-04	4.4E-02
chr6:35493816-35494551	736	5.1	5.3	5.0	↑	0.3	6.3E-04	9.5E-02
chr6:35553331-35554124	794	4.6	4.1	5.0	↓	-0.9	1.2E-10	7.0E-07
chr6:35573306-35573523	218	1.8	2.3	1.1	↑	1.2	2.6E-04	5.5E-02
chr6:35793054-35794420	1367	5.8	5.9	5.5	↑	0.4	2.7E-04	5.7E-02
chr6:35979523-35979914	392	2.5	2.9	1.9	↑	1.0	3.0E-04	6.1E-02
chr6:36602617-36602829	213	2.4	2.8	1.9	↑	0.9	6.1E-04	9.2E-02
chr6:36652318-36652918	601	3.3	2.3	3.9	↓	-1.6	5.4E-13	6.0E-09
chr6:36684994-36685278	285	2.5	2.0	3.0	↓	-1.0	1.4E-04	3.6E-02
chr6:36702175-36702518	344	3.1	2.5	3.4	↓	-0.9	9.7E-05	2.9E-02
chr6:36703672-36705005	1334	5.7	5.6	5.9	↓	-0.3	1.6E-04	4.1E-02
chr6:37042495-37042773	279	2.7	1.9	3.2	↓	-1.2	9.6E-07	8.4E-04
chr6:372973-373913	941	5.3	5.0	5.5	↓	-0.5	1.4E-05	6.7E-03
chr6:37707331-37707954	624	5.4	5.2	5.6	↓	-0.4	4.5E-04	7.9E-02
chr6:38625776-38626185	410	4.1	3.6	4.4	↓	-0.8	1.5E-06	1.2E-03
chr6:38974867-38976179	1313	5.2	5.0	5.4	↓	-0.4	1.8E-04	4.4E-02
chr6:39923826-39924125	300	2.5	2.0	2.9	↓	-0.9	4.1E-04	7.4E-02
chr6:4006150-4007029	880	5.0	4.8	5.2	↓	-0.4	3.2E-04	6.4E-02
chr6:40113537-40113911	375	4.2	3.8	4.4	↓	-0.6	6.2E-04	9.3E-02
chr6:40285530-40286103	574	3.7	3.3	4.0	↓	-0.7	3.9E-04	7.2E-02
chr6:40571122-40571440	319	2.4	2.8	1.8	↑	1.0	5.2E-04	8.5E-02
chr6:4086344-4088291	1948	6.3	6.2	6.4	↓	-0.3	5.6E-04	8.8E-02
chr6:40940176-40940431	256	2.0	1.2	2.5	↓	-1.3	1.5E-04	3.8E-02
chr6:40962897-40963291	395	4.2	3.9	4.4	↓	-0.5	6.5E-04	9.7E-02
chr6:41188108-41188323	216	1.7	2.2	0.9	↑	1.2	4.9E-04	8.3E-02
chr6:42808923-42809725	803	4.5	4.8	4.0	↑	0.7	2.8E-05	1.1E-02
chr6:4327816-4328261	446	3.1	2.5	3.5	↓	-1.0	5.0E-06	3.0E-03
chr6:43428666-43429609	944	5.0	4.8	5.2	↓	-0.4	4.4E-04	7.8E-02
chr6:43739702-43739912	211	1.5	2.0	0.5	↑	1.6	7.9E-05	2.5E-02
chr6:43987181-43987486	306	3.6	3.3	3.9	↓	-0.6	3.8E-04	7.1E-02

chr6:4415924-4420931	5008	7.6	7.5	7.7	↓	-0.2	1.5E-04	3.9E-02
chr6:45499548-45500020	473	3.7	3.3	4.1	↓	-0.8	5.2E-04	8.5E-02
chr6:45795022-45795337	316	2.9	2.4	3.3	↓	-1.0	1.6E-04	4.1E-02
chr6:45965413-45966811	1399	6.0	5.8	6.2	↓	-0.5	6.5E-05	2.2E-02
chr6:46395572-46395931	360	2.4	2.9	1.6	↑	1.3	1.1E-05	5.7E-03
chr6:46822490-46822970	481	4.2	3.9	4.5	↓	-0.6	2.0E-04	4.6E-02
chr6:47122920-47123922	1003	5.4	5.0	5.7	↓	-0.6	1.9E-06	1.4E-03
chr6:47459004-47459335	332	3.8	3.4	4.1	↓	-0.6	5.1E-04	8.4E-02
chr6:4750114-4750494	381	2.8	2.2	3.2	↓	-1.0	8.8E-05	2.7E-02
chr6:50519713-50520188	476	4.7	4.4	5.0	↓	-0.6	1.3E-06	1.1E-03
chr6:5066616-5066933	318	3.0	3.4	2.6	↑	0.8	2.3E-04	5.1E-02
chr6:50792281-50792548	268	2.9	2.4	3.4	↓	-1.0	4.0E-05	1.5E-02
chr6:50835379-50835722	344	2.6	2.0	3.0	↓	-1.0	2.9E-04	6.0E-02
chr6:5092971-5094965	1995	6.4	6.3	6.5	↓	-0.2	3.0E-04	6.0E-02
chr6:51174911-51176807	1897	5.9	5.6	6.1	↓	-0.4	6.3E-07	6.2E-04
chr6:51191922-51194077	2156	6.6	6.5	6.7	↓	-0.2	6.6E-04	9.7E-02
chr6:51271874-51272255	382	3.9	3.5	4.2	↓	-0.6	9.8E-05	2.9E-02
chr6:5141461-5141919	459	3.8	3.3	4.2	↓	-0.9	6.2E-06	3.6E-03
chr6:51858139-51858968	830	5.2	4.9	5.4	↓	-0.5	2.5E-04	5.4E-02
chr6:51882741-51883468	728	3.8	4.2	3.2	↑	1.0	1.7E-04	4.1E-02
chr6:5334678-5335766	1089	5.5	5.3	5.6	↓	-0.4	3.5E-04	6.8E-02
chr6:53429374-53429778	405	3.6	3.0	4.0	↓	-1.0	3.6E-06	2.3E-03
chr6:54425930-54426266	337	2.6	3.1	1.9	↑	1.2	6.7E-06	3.8E-03
chr6:54509387-54513446	4060	9.0	8.9	9.2	↓	-0.3	5.6E-04	8.8E-02
chr6:54691637-54692420	784	5.2	4.9	5.4	↓	-0.6	8.0E-07	7.6E-04
chr6:56556173-56558174	2002	6.7	6.6	6.9	↓	-0.3	1.0E-04	2.9E-02
chr6:56571490-56572296	807	4.0	3.6	4.2	↓	-0.6	4.2E-04	7.6E-02
chr6:57106017-57106230	214	1.7	2.3	0.9	↑	1.3	1.3E-04	3.4E-02
chr6:57673895-57674408	514	4.6	4.4	4.8	↓	-0.5	1.7E-04	4.2E-02
chr6:57870522-57872194	1673	6.5	6.3	6.7	↓	-0.4	1.5E-04	3.8E-02
chr6:58302067-58302944	878	5.4	5.2	5.6	↓	-0.4	5.6E-04	8.8E-02
chr6:58325909-58326477	569	4.6	4.3	4.8	↓	-0.5	6.6E-04	9.7E-02
chr6:58398292-58398640	349	2.7	3.2	1.9	↑	1.3	1.5E-06	1.2E-03
chr6:5878573-5879040	468	3.2	2.6	3.5	↓	-0.9	2.7E-05	1.1E-02
chr6:58853907-58854328	422	3.7	3.3	4.0	↓	-0.7	1.3E-04	3.4E-02
chr6:59125678-59126852	1175	5.7	5.4	5.9	↓	-0.5	1.1E-05	5.7E-03
chr6:59294363-59296771	2409	7.5	7.3	7.6	↓	-0.3	1.7E-06	1.3E-03
chr6:59459144-59459442	299	2.2	1.6	2.6	↓	-1.0	5.3E-04	8.6E-02
chr6:59503016-59505204	2189	7.5	7.4	7.6	↓	-0.2	3.5E-04	6.7E-02
chr6:5952453-5952658	206	2.1	1.5	2.6	↓	-1.1	4.2E-04	7.6E-02
chr6:59671726-59672208	483	4.2	4.5	3.9	↑	0.6	4.9E-06	3.0E-03
chr6:59764555-59765122	568	4.9	4.5	5.2	↓	-0.7	2.5E-05	1.1E-02
chr6:59856911-59857663	753	5.8	5.6	6.0	↓	-0.4	2.1E-06	1.5E-03
chr6:59922418-59922741	324	2.1	1.5	2.5	↓	-1.0	4.8E-04	8.2E-02
chr6:59924794-59925669	876	4.7	4.3	4.9	↓	-0.6	1.5E-04	3.8E-02
chr6:59948924-59949569	646	5.5	5.2	5.7	↓	-0.5	3.5E-05	1.4E-02
chr6:60529770-60530279	510	4.3	4.1	4.5	↓	-0.5	5.5E-04	8.8E-02

chr6:6095029-6095584	556	4.2	3.6	4.6	↓	-1.0	8.6E-10	3.2E-06
chr6:61907532-61907816	285	2.2	2.6	1.6	↑	1.0	6.4E-04	9.5E-02
chr6:62128497-62129231	735	4.9	4.4	5.4	↓	-1.0	3.0E-09	8.1E-06
chr6:62280244-62280535	292	3.1	3.5	2.7	↑	0.7	4.2E-04	7.5E-02
chr6:62682343-62683114	772	4.7	4.3	5.0	↓	-0.7	2.4E-07	2.8E-04
chr6:62772825-62773110	286	3.6	3.0	4.0	↓	-1.0	2.8E-06	1.9E-03
chr6:62815433-62815747	315	2.2	1.5	2.7	↓	-1.2	1.6E-04	4.0E-02
chr6:62878767-62879193	427	3.2	2.7	3.6	↓	-0.9	2.0E-05	8.9E-03
chr6:62909999-62910347	349	3.2	2.6	3.7	↓	-1.0	5.7E-07	5.8E-04
chr6:6300930-6301213	284	3.4	3.7	3.0	↑	0.6	5.8E-04	9.0E-02
chr6:63840515-63840751	237	1.7	0.3	2.4	↓	-2.0	7.1E-08	1.2E-04
chr6:64797370-64799326	1957	5.8	5.9	5.6	↑	0.3	6.5E-05	2.2E-02
chr6:65067977-65068530	554	2.8	3.2	2.2	↑	1.0	3.7E-05	1.4E-02
chr6:65082599-65083570	972	5.9	5.7	6.1	↓	-0.4	5.4E-08	1.0E-04
chr6:65172406-65173983	1578	6.6	6.3	6.8	↓	-0.5	4.8E-08	9.2E-05
chr6:65243357-65245505	2149	6.0	6.1	5.8	↑	0.3	3.0E-04	6.0E-02
chr6:65246643-65249000	2358	6.3	6.5	6.2	↑	0.3	7.2E-05	2.3E-02
chr6:65249432-65249928	497	4.8	5.0	4.5	↑	0.5	4.0E-06	2.5E-03
chr6:65252983-65253641	659	5.8	5.6	6.0	↓	-0.4	2.3E-04	5.1E-02
chr6:65256840-65257461	622	3.7	3.1	4.1	↓	-1.0	1.1E-07	1.5E-04
chr6:65468002-65468464	463	3.2	2.7	3.5	↓	-0.8	2.7E-04	5.7E-02
chr6:65536344-65537028	685	6.2	6.0	6.4	↓	-0.4	4.2E-08	8.2E-05
chr6:65732114-65732808	695	5.0	4.7	5.2	↓	-0.6	4.9E-05	1.8E-02
chr6:66489914-66491182	1269	4.8	4.4	5.2	↓	-0.8	9.7E-07	8.4E-04
chr6:66491256-66491706	451	3.2	2.8	3.5	↓	-0.7	2.6E-04	5.6E-02
chr6:66492355-66493212	858	4.3	3.5	4.8	↓	-1.3	2.0E-12	1.8E-08
chr6:66545127-66545792	666	4.1	3.8	4.4	↓	-0.6	6.1E-04	9.2E-02
chr6:66581816-66583020	1205	4.3	3.9	4.6	↓	-0.6	2.1E-05	9.2E-03
chr6:66720559-66721174	616	4.2	3.8	4.5	↓	-0.7	3.2E-06	2.1E-03
chr6:66722475-66722793	319	3.0	2.5	3.4	↓	-0.9	5.0E-05	1.8E-02
chr6:66728471-66728743	273	2.9	2.4	3.3	↓	-0.9	1.1E-04	3.1E-02
chr6:66728970-66729917	948	4.4	4.1	4.7	↓	-0.6	5.1E-05	1.8E-02
chr6:67073051-67073286	236	3.4	2.9	3.8	↓	-0.9	1.2E-05	5.9E-03
chr6:67477516-67478822	1307	5.6	5.3	5.8	↓	-0.4	3.5E-05	1.4E-02
chr6:67704910-67705221	312	3.6	3.2	3.9	↓	-0.7	5.4E-04	8.6E-02
chr6:68202783-68203096	314	2.3	1.8	2.8	↓	-1.0	4.7E-04	8.0E-02
chr6:68302575-68303179	605	4.6	3.9	5.1	↓	-1.2	6.9E-10	2.7E-06
chr6:68326924-68327175	252	2.6	3.0	2.1	↑	1.0	1.9E-04	4.5E-02
chr6:68377272-68378034	763	4.9	4.6	5.2	↓	-0.6	1.9E-07	2.4E-04
chr6:68867214-68868804	1591	6.0	5.9	6.2	↓	-0.3	3.3E-05	1.3E-02
chr6:69001571-69001771	201	2.1	2.6	1.5	↑	1.1	2.4E-04	5.3E-02
chr6:69019197-69019465	269	2.1	1.4	2.5	↓	-1.1	2.1E-04	4.8E-02
chr6:69088799-69089219	421	3.2	2.8	3.6	↓	-0.8	4.3E-04	7.6E-02
chr6:69745760-69745988	229	2.8	2.3	3.2	↓	-0.8	5.8E-04	9.0E-02
chr6:69808694-69808992	299	1.6	0.7	2.2	↓	-1.5	3.9E-05	1.5E-02
chr6:70311267-70311665	399	3.1	3.5	2.6	↑	0.8	2.6E-04	5.6E-02
chr6:70766203-70766861	659	4.0	3.7	4.3	↓	-0.6	1.4E-04	3.7E-02

chr6:71072609-71072868	260	2.3	2.8	1.7	↑	1.1	2.5E-04	5.3E-02
chr6:71128774-71129084	311	2.7	3.2	2.0	↑	1.2	1.1E-05	5.7E-03
chr6:7150369-7150977	609	3.7	3.3	4.0	↓	-0.7	5.0E-04	8.4E-02
chr6:71592132-71592418	287	2.7	2.2	3.1	↓	-0.9	4.4E-04	7.7E-02
chr6:71943091-71943833	743	4.0	3.7	4.3	↓	-0.7	3.0E-04	6.1E-02
chr6:72493240-72493557	318	2.8	2.3	3.1	↓	-0.9	3.8E-04	7.1E-02
chr6:72547711-72547982	272	2.5	1.9	2.9	↓	-1.0	3.0E-04	6.0E-02
chr6:7263877-7265779	1903	7.2	7.3	7.1	↑	0.2	5.2E-04	8.5E-02
chr6:7265919-7267064	1146	6.4	6.2	6.5	↓	-0.3	1.2E-04	3.3E-02
chr6:72873043-72873332	290	3.8	3.5	4.1	↓	-0.6	6.0E-04	9.2E-02
chr6:72982746-72983319	574	4.5	4.2	4.8	↓	-0.6	1.4E-05	6.7E-03
chr6:73147788-73148402	615	4.5	4.2	4.8	↓	-0.5	8.9E-05	2.7E-02
chr6:7322012-7323943	1932	7.2	7.3	7.1	↑	0.2	9.9E-05	2.9E-02
chr6:7324059-7326045	1987	6.6	6.8	6.4	↑	0.3	8.2E-05	2.6E-02
chr6:7326084-7326629	546	5.2	5.0	5.4	↓	-0.4	2.4E-05	1.0E-02
chr6:7362845-7363746	902	5.0	5.3	4.8	↑	0.5	4.0E-04	7.3E-02
chr6:7387008-7387451	444	3.3	2.9	3.7	↓	-0.8	1.2E-04	3.2E-02
chr6:74019683-74020033	351	2.8	3.2	2.3	↑	0.8	5.9E-04	9.1E-02
chr6:74341626-74341998	373	3.2	3.6	2.7	↑	0.9	1.0E-04	2.9E-02
chr6:74721490-74722066	577	4.2	4.5	3.9	↑	0.6	3.6E-04	6.9E-02
chr6:74724535-74725185	651	4.6	4.8	4.3	↑	0.5	1.7E-04	4.1E-02
chr6:74750406-74751230	825	4.8	5.1	4.5	↑	0.6	7.4E-07	7.2E-04
chr6:74758936-74759625	690	4.4	4.7	3.9	↑	0.9	1.2E-07	1.6E-04
chr6:74760127-74760549	423	3.3	2.6	3.8	↓	-1.2	1.0E-07	1.5E-04
chr6:74772900-74774158	1259	4.6	4.8	4.3	↑	0.5	5.3E-05	1.9E-02
chr6:74850485-74851024	540	3.7	4.0	3.4	↑	0.6	5.3E-04	8.6E-02
chr6:75517651-75518796	1146	4.5	4.2	4.7	↓	-0.6	2.7E-04	5.7E-02
chr6:75889213-75890080	868	5.4	5.3	5.6	↓	-0.3	6.0E-04	9.2E-02
chr6:76677624-76678225	602	3.9	3.4	4.2	↓	-0.8	5.8E-05	2.0E-02
chr6:7677607-7679094	1488	5.7	5.5	5.9	↓	-0.4	1.0E-05	5.4E-03
chr6:76783005-76783580	576	4.1	4.5	3.6	↑	0.8	3.4E-07	3.8E-04
chr6:7680217-7681029	813	5.0	4.6	5.3	↓	-0.7	3.2E-08	6.6E-05
chr6:78141206-78143167	1962	5.5	5.2	5.7	↓	-0.4	1.7E-06	1.3E-03
chr6:78313590-78314704	1115	5.4	5.1	5.6	↓	-0.5	5.9E-07	5.8E-04
chr6:79917664-79918500	837	5.0	5.4	4.5	↑	1.0	1.0E-09	3.6E-06
chr6:80052371-80052846	476	5.1	4.7	5.4	↓	-0.7	2.4E-09	7.2E-06
chr6:80134077-80134437	361	3.7	3.4	4.0	↓	-0.6	2.2E-04	5.0E-02
chr6:80135210-80135862	653	4.8	4.5	5.1	↓	-0.5	2.2E-06	1.6E-03
chr6:80834633-80835035	403	3.8	3.3	4.1	↓	-0.8	9.7E-05	2.9E-02
chr6:81130099-81130542	444	3.9	4.2	3.6	↑	0.6	2.7E-04	5.8E-02
chr6:81652660-81652858	199	1.8	2.3	1.0	↑	1.3	1.9E-04	4.6E-02
chr6:83053500-83054390	891	5.6	5.4	5.7	↓	-0.3	5.0E-04	8.3E-02
chr6:83067567-83067998	432	3.2	2.8	3.6	↓	-0.7	6.4E-04	9.5E-02
chr6:83651247-83651510	264	2.1	1.4	2.6	↓	-1.1	2.8E-04	5.8E-02
chr6:8389173-8389670	498	4.5	4.3	4.7	↓	-0.5	2.9E-04	6.0E-02
chr6:84023313-84025048	1736	6.5	6.3	6.6	↓	-0.2	2.3E-04	5.1E-02
chr6:8503888-8505263	1376	5.1	4.8	5.4	↓	-0.6	2.7E-05	1.1E-02

chr6:8542782-8543175	394	3.5	3.1	3.8	↓	-0.8	3.4E-05	1.3E-02
chr6:8550278-8551577	1300	6.0	5.8	6.2	↓	-0.4	1.1E-06	8.9E-04
chr6:8570359-8570848	490	2.7	3.1	2.2	↑	1.0	2.8E-04	5.8E-02
chr6:85808766-85809010	245	2.5	2.0	2.9	↓	-0.9	5.1E-04	8.4E-02
chr6:86094343-86094749	407	3.5	3.1	3.8	↓	-0.7	3.4E-04	6.6E-02
chr6:86122536-86124808	2273	5.7	5.6	5.9	↓	-0.3	5.5E-04	8.8E-02
chr6:86253615-86253935	321	2.5	1.9	2.9	↓	-1.1	7.9E-05	2.5E-02
chr6:86802612-86803819	1208	6.2	6.1	6.4	↓	-0.3	3.8E-06	2.4E-03
chr6:87015177-87015830	654	4.1	3.7	4.4	↓	-0.7	2.7E-05	1.1E-02
chr6:87186044-87186440	397	4.6	4.3	4.8	↓	-0.5	3.7E-04	7.0E-02
chr6:8762717-8763748	1032	5.6	5.4	5.8	↓	-0.4	3.1E-04	6.1E-02
chr6:88106123-88106386	264	2.3	1.6	2.7	↓	-1.1	2.0E-04	4.7E-02
chr6:88130324-88130612	289	2.3	1.7	2.7	↓	-1.0	4.5E-04	7.9E-02
chr6:88304434-88304791	358	2.5	1.9	2.9	↓	-1.0	5.2E-04	8.5E-02
chr6:8946394-8947760	1367	5.8	5.7	6.0	↓	-0.3	9.4E-05	2.9E-02
chr6:89935976-89937038	1063	4.6	4.9	4.3	↑	0.6	6.8E-04	9.9E-02
chr6:90251534-90252167	634	3.1	3.5	2.7	↑	0.8	2.4E-04	5.2E-02
chr6:90255301-90256069	769	4.3	4.0	4.6	↓	-0.7	1.3E-05	6.3E-03
chr6:90441339-90441685	347	2.6	2.1	3.1	↓	-1.0	1.0E-04	3.0E-02
chr6:90733751-90734131	381	2.8	2.3	3.2	↓	-0.9	3.0E-04	6.1E-02
chr6:91303703-91303915	213	1.7	2.2	0.9	↑	1.4	9.9E-05	2.9E-02
chr6:91353952-91354710	759	3.6	2.8	4.1	↓	-1.4	2.9E-11	1.8E-07
chr6:918869-920676	1808	6.2	6.0	6.4	↓	-0.4	5.2E-06	3.1E-03
chr6:92049092-92049390	299	2.5	1.9	2.9	↓	-1.1	7.2E-05	2.3E-02
chr6:92453073-92453647	575	3.6	3.9	3.2	↑	0.7	8.9E-05	2.7E-02
chr6:92456489-92457252	764	5.0	5.2	4.7	↑	0.5	1.5E-05	7.3E-03
chr6:9316174-9316717	544	4.4	4.0	4.6	↓	-0.6	2.3E-05	9.8E-03
chr6:9321899-9322822	924	6.7	6.5	6.9	↓	-0.3	1.7E-06	1.3E-03
chr6:94269421-94269755	335	2.0	1.3	2.5	↓	-1.2	1.1E-04	3.2E-02
chr6:94337641-94337969	329	3.4	2.8	3.8	↓	-1.0	9.3E-07	8.4E-04
chr6:9434301-9435909	1609	5.7	5.5	5.8	↓	-0.3	4.3E-04	7.7E-02
chr6:94353457-94353858	402	4.6	4.4	4.8	↓	-0.4	3.1E-04	6.1E-02
chr6:94529811-94530054	244	2.8	3.2	2.2	↑	0.9	2.4E-04	5.3E-02
chr6:9490676-9490998	323	2.7	3.0	2.2	↑	0.8	5.9E-04	9.1E-02
chr6:950232-950522	291	4.3	4.6	4.1	↑	0.5	6.1E-04	9.2E-02
chr6:96536836-96537595	760	4.1	3.9	4.4	↓	-0.5	6.2E-04	9.3E-02
chr6:98534030-98534892	863	4.3	3.1	4.9	↓	-1.8	1.1E-22	3.3E-18
chr6:9964352-9965234	883	4.3	3.9	4.6	↓	-0.7	1.1E-06	9.3E-04
chr6:99837068-99837425	358	2.8	3.2	2.4	↑	0.8	5.3E-04	8.6E-02
chr6:99933729-99934276	548	3.6	3.9	3.3	↑	0.7	1.9E-04	4.5E-02
chr7:101276713-101277029	317	2.7	2.1	3.1	↓	-1.1	1.5E-05	5.7E-03
chr7:101720275-101720626	352	3.1	2.7	3.5	↓	-0.9	5.3E-04	7.2E-02
chr7:102111010-102111591	582	5.3	4.9	5.7	↓	-0.8	6.0E-08	7.1E-05
chr7:102416929-102417310	382	3.6	3.3	3.9	↓	-0.7	3.7E-04	5.8E-02
chr7:102682137-102683653	1517	6.1	5.9	6.3	↓	-0.4	2.1E-07	1.9E-04
chr7:103112818-103113334	517	5.6	5.3	5.7	↓	-0.4	4.9E-04	6.9E-02
chr7:1032848-1033975	1128	5.7	5.3	5.9	↓	-0.7	8.2E-05	2.0E-02

chr7:103799369-103799589	221	1.8	2.4	1.0	↑	1.3	8.7E-05	2.1E-02
chr7:104607467-104607910	444	3.0	2.5	3.3	↓	-0.8	6.0E-04	7.8E-02
chr7:104696640-104697227	588	4.2	3.3	4.7	↓	-1.4	1.1E-14	6.6E-11
chr7:105230324-105231193	870	4.3	4.0	4.5	↓	-0.6	2.8E-04	4.8E-02
chr7:105591594-105592943	1350	5.8	5.5	6.0	↓	-0.5	2.4E-04	4.4E-02
chr7:105626257-105626520	264	2.3	1.8	2.7	↓	-0.9	7.7E-04	9.0E-02
chr7:105892331-105892792	462	2.6	1.9	3.1	↓	-1.2	2.1E-06	1.1E-03
chr7:106003867-106004240	374	4.2	3.9	4.4	↓	-0.5	4.2E-04	6.3E-02
chr7:106029404-106029637	234	1.9	1.0	2.4	↓	-1.4	4.4E-05	1.3E-02
chr7:106035088-106035380	293	2.5	3.0	1.6	↑	1.4	7.6E-07	5.5E-04
chr7:106035763-106036449	687	4.1	3.5	4.4	↓	-0.9	1.1E-06	6.8E-04
chr7:106257121-106257445	325	3.3	2.9	3.6	↓	-0.7	6.9E-04	8.5E-02
chr7:10698169-10698562	394	2.4	1.7	2.8	↓	-1.1	5.4E-05	1.5E-02
chr7:106990957-106992061	1105	4.7	5.0	4.2	↑	0.8	3.7E-06	1.8E-03
chr7:107156867-107157231	365	2.6	3.1	1.9	↑	1.2	4.5E-06	2.1E-03
chr7:107157886-107160436	2551	6.6	6.7	6.4	↑	0.3	5.7E-05	1.5E-02
chr7:107203319-107204528	1210	4.3	4.7	3.9	↑	0.8	2.3E-06	1.2E-03
chr7:10721923-10722961	1039	5.7	5.3	5.9	↓	-0.6	1.7E-08	2.2E-05
chr7:107289082-107289481	400	3.1	3.5	2.6	↑	0.8	3.0E-04	5.1E-02
chr7:107545536-107545873	338	2.7	3.1	2.0	↑	1.1	8.5E-06	3.6E-03
chr7:107561465-107561954	490	3.4	3.8	2.9	↑	0.9	5.2E-06	2.4E-03
chr7:107693709-107694051	343	2.7	3.1	2.1	↑	1.0	4.4E-05	1.3E-02
chr7:107730049-107730479	431	2.6	3.0	2.1	↑	0.9	6.5E-04	8.1E-02
chr7:107788041-107788437	397	2.7	2.2	3.1	↓	-0.9	7.2E-04	8.7E-02
chr7:107828447-107829392	946	5.2	5.5	4.9	↑	0.6	3.6E-05	1.1E-02
chr7:108019658-108019856	199	2.1	2.6	1.2	↑	1.4	6.8E-06	3.0E-03
chr7:108478626-108478978	353	2.4	2.8	1.8	↑	1.0	4.0E-04	6.2E-02
chr7:1087092-1087636	545	4.2	3.9	4.5	↓	-0.6	5.0E-04	7.0E-02
chr7:10941847-10942223	377	2.5	1.9	3.0	↓	-1.1	2.8E-05	9.1E-03
chr7:109442146-109442471	326	3.4	3.6	3.0	↑	0.7	5.2E-04	7.2E-02
chr7:110031632-110032109	478	3.5	3.1	3.8	↓	-0.7	5.2E-04	7.1E-02
chr7:110034527-110035104	578	3.6	2.8	4.2	↓	-1.4	9.5E-14	5.2E-10
chr7:110035292-110035504	213	1.8	1.1	2.3	↓	-1.2	2.3E-04	4.4E-02
chr7:110036410-110036798	389	3.6	3.0	4.1	↓	-1.0	2.8E-06	1.4E-03
chr7:110037511-110039565	2055	6.7	6.4	6.9	↓	-0.5	5.9E-07	4.5E-04
chr7:110040032-110040758	727	4.6	4.2	4.8	↓	-0.6	5.4E-06	2.5E-03
chr7:110040782-110043257	2476	6.3	5.8	6.6	↓	-0.8	3.4E-09	5.5E-06
chr7:110043299-110043969	671	4.3	3.9	4.6	↓	-0.7	8.0E-06	3.4E-03
chr7:110044029-110044502	474	3.0	2.3	3.5	↓	-1.1	4.5E-07	3.7E-04
chr7:110230390-110231257	868	5.5	5.3	5.7	↓	-0.4	7.3E-04	8.7E-02
chr7:110232124-110232442	319	2.6	2.2	3.0	↓	-0.8	7.5E-04	8.9E-02
chr7:11130464-11131901	1438	5.4	5.2	5.6	↓	-0.3	4.4E-04	6.6E-02
chr7:11229483-11230399	917	5.4	5.1	5.6	↓	-0.5	2.1E-04	4.1E-02
chr7:11280895-11281271	377	3.5	3.0	3.9	↓	-0.9	9.1E-06	3.8E-03
chr7:113055944-113056524	581	3.3	3.6	2.9	↑	0.7	7.9E-04	9.2E-02
chr7:113267222-113267534	313	2.7	2.1	3.1	↓	-1.1	1.9E-05	6.9E-03
chr7:113268140-113268366	227	2.1	1.0	2.7	↓	-1.6	5.0E-07	4.0E-04

chr7:113268751-113269377	627	3.9	3.5	4.1	↓	-0.6	3.8E-04	5.9E-02
chr7:11332890-11333132	243	2.0	1.3	2.4	↓	-1.1	6.3E-04	8.0E-02
chr7:113402789-113403452	664	3.1	2.6	3.5	↓	-0.9	6.5E-05	1.7E-02
chr7:113428560-113429555	996	5.0	5.4	4.5	↑	0.9	1.7E-05	6.1E-03
chr7:113431889-113432650	762	4.2	4.5	3.8	↑	0.7	1.6E-05	5.9E-03
chr7:113551105-113551309	205	1.6	2.2	0.7	↑	1.5	4.7E-05	1.4E-02
chr7:113623915-113624190	276	3.2	3.6	2.7	↑	0.9	5.6E-05	1.5E-02
chr7:113796139-113796479	341	2.7	3.1	2.1	↑	0.9	4.9E-04	6.9E-02
chr7:113898175-113898545	371	2.2	2.6	1.6	↑	1.0	5.3E-04	7.2E-02
chr7:114130654-114131303	650	3.2	3.6	2.8	↑	0.7	3.6E-04	5.7E-02
chr7:114136626-114136928	303	3.4	3.9	2.8	↑	1.0	1.7E-06	9.4E-04
chr7:114139506-114140933	1428	6.0	6.2	5.6	↑	0.6	1.1E-05	4.5E-03
chr7:114158490-114158693	204	1.5	2.1	0.7	↑	1.3	4.6E-04	6.8E-02
chr7:114231520-114231977	458	3.3	3.7	2.8	↑	0.9	1.3E-05	5.0E-03
chr7:11439355-11440219	865	4.0	3.5	4.4	↓	-0.9	2.4E-09	4.0E-06
chr7:11453447-11454023	577	3.9	3.4	4.2	↓	-0.7	1.8E-04	3.6E-02
chr7:11490083-11492291	2209	6.1	5.8	6.3	↓	-0.5	1.9E-07	1.8E-04
chr7:115091868-115092191	324	1.8	0.8	2.3	↓	-1.5	7.0E-05	1.8E-02
chr7:115301037-115301412	376	1.9	2.5	0.8	↑	1.6	1.4E-06	8.3E-04
chr7:11569955-11570591	637	4.4	4.8	4.0	↑	0.8	9.4E-07	6.5E-04
chr7:11601407-11602429	1023	6.0	5.9	6.2	↓	-0.3	1.7E-05	6.1E-03
chr7:116184556-116184981	426	2.8	2.3	3.2	↓	-0.8	3.2E-04	5.3E-02
chr7:11651972-11652651	680	4.4	4.1	4.7	↓	-0.6	4.4E-04	6.6E-02
chr7:117753493-117753721	229	1.8	2.3	1.1	↑	1.2	7.4E-04	8.8E-02
chr7:117770007-117770276	270	2.2	1.7	2.7	↓	-1.0	7.2E-04	8.7E-02
chr7:118104718-118104952	235	2.5	1.8	2.9	↓	-1.2	2.3E-05	7.9E-03
chr7:118187808-118188518	711	4.4	4.1	4.6	↓	-0.4	8.8E-04	9.9E-02
chr7:118259586-118259941	356	3.4	3.0	3.7	↓	-0.7	3.8E-04	5.9E-02
chr7:118569402-118569800	399	2.8	2.2	3.2	↓	-0.9	2.3E-04	4.3E-02
chr7:118942643-118942981	339	2.8	2.3	3.2	↓	-0.9	2.6E-04	4.7E-02
chr7:119694002-119694236	235	2.0	2.5	1.4	↑	1.1	6.4E-04	8.0E-02
chr7:120208887-120209196	310	3.1	2.7	3.4	↓	-0.7	7.0E-04	8.6E-02
chr7:12030128-12030293	166	1.8	2.3	1.1	↑	1.2	4.0E-04	6.2E-02
chr7:120362077-120362962	886	5.4	4.9	5.7	↓	-0.7	1.8E-07	1.7E-04
chr7:120457548-120457992	445	4.3	3.7	4.7	↓	-1.0	1.2E-09	2.3E-06
chr7:120491687-120492210	524	3.0	2.6	3.4	↓	-0.8	8.9E-04	9.9E-02
chr7:120789209-120789742	534	5.2	5.0	5.4	↓	-0.4	4.7E-04	6.8E-02
chr7:12080874-12081436	563	3.0	2.5	3.4	↓	-0.9	1.2E-04	2.7E-02
chr7:121024362-121025676	1315	6.2	5.9	6.5	↓	-0.7	1.2E-07	1.2E-04
chr7:121540119-121540341	223	2.7	3.1	2.0	↑	1.1	2.8E-04	4.8E-02
chr7:12155741-12156126	386	2.8	2.0	3.3	↓	-1.3	1.7E-06	9.4E-04
chr7:121675634-121676148	515	4.9	4.6	5.1	↓	-0.4	4.4E-04	6.6E-02
chr7:121891774-121892282	509	4.3	4.0	4.5	↓	-0.5	4.7E-04	6.8E-02
chr7:121899723-121899989	267	2.7	2.2	3.0	↓	-0.9	5.6E-04	7.4E-02
chr7:122138940-122139702	763	4.4	4.1	4.6	↓	-0.5	2.7E-04	4.8E-02
chr7:122914410-122914666	257	2.0	1.0	2.5	↓	-1.5	4.6E-06	2.2E-03
chr7:123331023-123331574	552	5.0	4.6	5.3	↓	-0.7	1.6E-10	4.5E-07

chr7:123353533-123354260	728	5.1	4.8	5.4	↓	-0.6	1.2E-06	7.6E-04
chr7:123473503-123474277	775	5.0	4.5	5.4	↓	-0.9	9.4E-08	1.0E-04
chr7:123478585-123479697	1113	5.6	5.8	5.5	↑	0.3	8.5E-04	9.7E-02
chr7:123565413-123566885	1473	5.3	4.9	5.5	↓	-0.6	6.1E-08	7.1E-05
chr7:123567670-123568801	1132	6.5	6.3	6.7	↓	-0.4	4.5E-09	7.0E-06
chr7:123589267-123589619	353	3.9	3.6	4.2	↓	-0.7	1.3E-04	2.9E-02
chr7:123632328-123632569	242	2.4	2.8	1.9	↑	0.9	5.2E-04	7.1E-02
chr7:123686388-123688134	1747	5.8	6.0	5.6	↑	0.4	5.2E-07	4.1E-04
chr7:123697346-123698385	1040	4.9	4.6	5.1	↓	-0.5	5.2E-05	1.5E-02
chr7:123928441-123929101	661	3.6	3.2	4.0	↓	-0.8	6.3E-05	1.7E-02
chr7:123976295-123976624	330	2.8	2.1	3.2	↓	-1.1	1.0E-05	4.2E-03
chr7:123982697-123983358	662	4.3	3.9	4.6	↓	-0.6	2.4E-04	4.4E-02
chr7:124148458-124150216	1759	6.8	6.6	6.9	↓	-0.3	8.4E-04	9.6E-02
chr7:124242238-124242845	608	4.0	3.6	4.3	↓	-0.7	2.0E-04	3.9E-02
chr7:124446712-124447523	812	4.9	4.7	5.1	↓	-0.4	7.2E-04	8.7E-02
chr7:124577503-124578121	619	3.3	2.9	3.6	↓	-0.7	2.8E-04	4.8E-02
chr7:125161613-125162127	515	4.1	3.7	4.3	↓	-0.6	2.0E-04	3.9E-02
chr7:125611451-125611848	398	3.3	2.8	3.7	↓	-0.9	1.1E-04	2.5E-02
chr7:125730634-125731162	529	4.1	3.7	4.4	↓	-0.7	4.2E-07	3.5E-04
chr7:125923734-125925247	1514	5.7	5.3	5.9	↓	-0.6	4.4E-11	1.3E-07
chr7:126062657-126062944	288	2.7	3.2	1.8	↑	1.4	4.9E-08	6.0E-05
chr7:126548511-126548823	313	2.5	1.7	3.0	↓	-1.3	1.2E-06	7.6E-04
chr7:127058381-127058937	557	3.7	3.2	4.0	↓	-0.8	1.4E-04	3.0E-02
chr7:127134529-127135016	488	4.2	3.9	4.4	↓	-0.5	6.3E-04	8.0E-02
chr7:127473000-127473355	356	2.4	1.9	2.8	↓	-0.9	8.6E-04	9.7E-02
chr7:127565304-127565851	548	4.2	3.5	4.6	↓	-1.2	1.2E-12	4.5E-09
chr7:127896867-127897964	1098	4.9	4.7	5.1	↓	-0.4	2.8E-04	4.8E-02
chr7:127906828-127908140	1313	5.7	5.4	5.9	↓	-0.5	8.8E-05	2.1E-02
chr7:128627933-128628256	324	1.8	2.3	1.2	↑	1.1	4.7E-04	6.8E-02
chr7:128682114-128683368	1255	6.3	6.4	6.2	↑	0.2	5.5E-04	7.4E-02
chr7:12872887-12873324	438	3.0	3.3	2.5	↑	0.8	6.5E-04	8.1E-02
chr7:128876679-128880455	3777	7.8	7.6	7.9	↓	-0.2	3.3E-05	1.1E-02
chr7:128943894-128944505	612	5.8	5.6	5.9	↓	-0.3	3.0E-04	5.1E-02
chr7:129242595-129242792	198	2.0	1.2	2.5	↓	-1.3	5.8E-05	1.6E-02
chr7:129590230-129590545	316	3.4	3.0	3.7	↓	-0.7	7.7E-04	9.0E-02
chr7:12995974-12996374	401	4.7	4.3	5.0	↓	-0.7	1.2E-04	2.8E-02
chr7:130104778-130104995	218	2.1	1.5	2.6	↓	-1.1	2.2E-04	4.1E-02
chr7:130272970-130277155	4186	7.6	7.4	7.7	↓	-0.2	8.4E-07	5.8E-04
chr7:130395365-130396684	1320	6.0	5.7	6.2	↓	-0.5	1.3E-06	7.9E-04
chr7:130401467-130402011	545	5.4	5.1	5.6	↓	-0.5	9.9E-07	6.6E-04
chr7:130537331-130537850	520	3.5	3.1	3.8	↓	-0.7	1.9E-04	3.8E-02
chr7:13098520-13099285	766	4.4	4.0	4.8	↓	-0.8	5.7E-06	2.6E-03
chr7:13121446-13122019	574	3.3	3.6	2.9	↑	0.7	3.1E-04	5.2E-02
chr7:132032409-132033545	1137	5.0	4.8	5.2	↓	-0.4	2.5E-04	4.6E-02
chr7:132033586-132034319	734	4.9	4.6	5.1	↓	-0.5	1.2E-05	4.6E-03
chr7:132392344-132393346	1003	4.4	4.1	4.7	↓	-0.6	1.2E-04	2.8E-02
chr7:132495560-132497823	2264	6.5	6.4	6.6	↓	-0.2	3.4E-04	5.5E-02

chr7:133966941-133967678	738	4.6	4.3	4.8	↓	-0.5	2.8E-04	4.8E-02
chr7:134045112-134046345	1234	5.9	5.6	6.1	↓	-0.4	7.9E-05	2.0E-02
chr7:1367064-1368393	1330	5.3	4.8	5.6	↓	-0.8	1.1E-08	1.5E-05
chr7:1369105-1371465	2361	6.3	5.9	6.7	↓	-0.8	1.3E-08	1.7E-05
chr7:1385776-1387222	1447	5.6	5.3	5.8	↓	-0.5	3.4E-04	5.5E-02
chr7:14294894-14295391	498	3.0	3.4	2.5	↑	0.9	4.9E-04	6.9E-02
chr7:14406152-14406813	662	3.3	3.6	2.9	↑	0.7	5.0E-04	7.0E-02
chr7:14422519-14422838	320	2.8	3.2	2.2	↑	1.0	5.9E-04	7.7E-02
chr7:14463396-14463675	280	1.9	2.3	1.2	↑	1.1	4.7E-04	6.8E-02
chr7:1555188-1556599	1412	7.0	6.8	7.2	↓	-0.4	1.8E-06	9.7E-04
chr7:1563788-1567898	4111	7.9	7.7	8.0	↓	-0.3	1.5E-04	3.2E-02
chr7:15732155-15732409	255	2.1	1.5	2.6	↓	-1.1	4.4E-04	6.6E-02
chr7:15733145-15733575	431	2.7	1.8	3.3	↓	-1.4	1.7E-07	1.6E-04
chr7:16090516-16091029	514	3.9	3.4	4.4	↓	-1.0	7.3E-08	8.2E-05
chr7:1631136-1632256	1121	5.5	5.2	5.8	↓	-0.6	1.2E-09	2.3E-06
chr7:1704809-1706918	2110	7.2	7.0	7.4	↓	-0.4	2.5E-05	8.5E-03
chr7:17443362-17443730	369	3.4	3.0	3.7	↓	-0.7	3.2E-04	5.3E-02
chr7:17883072-17883715	644	3.2	2.8	3.5	↓	-0.7	8.5E-04	9.7E-02
chr7:18084271-18084800	530	4.0	3.7	4.3	↓	-0.7	7.3E-04	8.8E-02
chr7:18204652-18205761	1110	4.6	4.8	4.2	↑	0.6	1.9E-04	3.8E-02
chr7:18209048-18209260	213	2.5	3.0	1.8	↑	1.2	1.5E-05	5.6E-03
chr7:18415192-18415534	343	3.1	3.5	2.6	↑	0.9	5.4E-05	1.5E-02
chr7:18832065-18832308	244	2.1	2.5	1.4	↑	1.1	4.1E-04	6.2E-02
chr7:18988181-18988523	343	2.3	2.7	1.7	↑	1.0	2.8E-04	4.8E-02
chr7:2108941-2111517	2577	7.8	7.7	7.8	↓	-0.2	7.6E-04	9.0E-02
chr7:2132976-2133661	686	5.8	5.6	6.0	↓	-0.5	2.0E-06	1.1E-03
chr7:21400338-21400627	290	3.8	3.5	4.0	↓	-0.6	6.1E-04	7.8E-02
chr7:220701-223087	2387	7.0	6.8	7.1	↓	-0.4	5.1E-07	4.0E-04
chr7:22399206-22399624	419	3.4	2.8	3.9	↓	-1.1	1.3E-06	7.9E-04
chr7:22401296-22401591	296	1.5	2.0	0.8	↑	1.3	5.5E-04	7.4E-02
chr7:224942-225988	1047	5.8	5.6	6.0	↓	-0.4	8.6E-04	9.7E-02
chr7:22735-25379	2645	7.0	6.9	7.1	↓	-0.2	8.5E-05	2.1E-02
chr7:228244-229461	1218	5.5	5.3	5.6	↓	-0.3	4.9E-04	6.9E-02
chr7:232217-234966	2750	6.9	6.6	7.1	↓	-0.4	2.3E-09	4.0E-06
chr7:2353812-2354655	844	3.9	3.4	4.2	↓	-0.8	2.6E-04	4.7E-02
chr7:24398784-24399223	440	3.1	2.5	3.5	↓	-0.9	5.2E-05	1.5E-02
chr7:253571-255670	2100	6.5	6.4	6.7	↓	-0.2	4.6E-04	6.8E-02
chr7:2576254-2577730	1477	6.3	6.2	6.5	↓	-0.3	2.7E-05	8.8E-03
chr7:2676856-2678474	1619	6.1	5.9	6.3	↓	-0.4	7.0E-06	3.1E-03
chr7:2683906-2685885	1980	6.5	6.3	6.7	↓	-0.4	2.1E-05	7.2E-03
chr7:2686011-2686534	524	2.8	2.2	3.2	↓	-0.9	1.8E-04	3.6E-02
chr7:27337989-27338323	335	2.4	1.7	2.8	↓	-1.2	4.7E-05	1.4E-02
chr7:28004578-28006837	2260	6.3	6.1	6.5	↓	-0.4	4.1E-05	1.3E-02
chr7:28076488-28076938	451	2.8	2.3	3.1	↓	-0.8	6.5E-04	8.1E-02
chr7:29186606-29187754	1149	4.2	4.5	4.0	↑	0.5	6.8E-04	8.4E-02
chr7:29188204-29188678	475	3.0	3.4	2.5	↑	0.9	1.9E-04	3.8E-02
chr7:299916-300642	727	5.5	5.3	5.6	↓	-0.4	2.7E-04	4.8E-02

chr7:31191599-31191987	389	2.4	2.8	1.8	↑	1.0	2.7E-04	4.8E-02
chr7:31195614-31196396	783	4.3	4.6	3.8	↑	0.9	1.3E-07	1.3E-04
chr7:31200054-31200285	232	1.7	2.3	0.8	↑	1.5	3.2E-05	1.0E-02
chr7:31233286-31233549	264	2.4	2.8	1.9	↑	0.9	5.6E-04	7.4E-02
chr7:31908548-31908830	283	2.5	1.9	2.8	↓	-0.9	7.5E-04	8.9E-02
chr7:32317851-32318201	351	3.6	3.3	3.9	↓	-0.6	5.8E-04	7.6E-02
chr7:33481499-33481831	333	2.7	3.1	2.1	↑	0.9	1.2E-04	2.8E-02
chr7:33661338-33662862	1525	5.1	4.5	5.4	↓	-0.9	2.5E-10	6.0E-07
chr7:33674257-33674860	604	5.7	5.6	5.9	↓	-0.4	1.0E-04	2.5E-02
chr7:33919375-33919724	350	3.8	3.4	4.2	↓	-0.8	1.6E-04	3.3E-02
chr7:34180610-34181265	656	5.8	5.6	6.0	↓	-0.4	5.6E-04	7.4E-02
chr7:34657296-34658090	795	5.4	5.0	5.7	↓	-0.6	8.8E-05	2.1E-02
chr7:34669183-34670317	1135	5.4	5.2	5.7	↓	-0.5	6.0E-06	2.7E-03
chr7:34841377-34841921	545	3.5	3.1	3.8	↓	-0.7	1.5E-04	3.2E-02
chr7:34950129-34950366	238	1.8	2.3	0.9	↑	1.3	1.3E-04	2.8E-02
chr7:36223982-36225900	1919	6.8	6.6	7.0	↓	-0.4	1.1E-09	2.3E-06
chr7:36518675-36519194	520	4.6	4.3	4.8	↓	-0.5	3.6E-05	1.1E-02
chr7:36534749-36535671	923	4.3	4.0	4.5	↓	-0.5	2.2E-04	4.2E-02
chr7:37474726-37475111	386	2.7	2.1	3.1	↓	-1.0	7.4E-05	1.9E-02
chr7:38031742-38032000	259	2.7	2.0	3.2	↓	-1.1	2.1E-04	4.1E-02
chr7:38171034-38171384	351	3.1	3.5	2.6	↑	0.9	1.5E-05	5.6E-03
chr7:38996913-38997288	376	2.4	1.7	2.8	↓	-1.1	5.6E-05	1.5E-02
chr7:38997949-39001072	3124	6.5	6.1	6.9	↓	-0.8	5.8E-10	1.3E-06
chr7:39002297-39004102	1806	5.7	5.2	6.1	↓	-0.9	3.0E-13	1.3E-09
chr7:39007075-39008149	1075	5.5	5.3	5.7	↓	-0.5	1.0E-05	4.0E-03
chr7:39128685-39129209	525	2.8	2.3	3.2	↓	-0.9	1.2E-04	2.8E-02
chr7:39480841-39481121	281	2.1	1.2	2.7	↓	-1.5	1.2E-06	7.6E-04
chr7:39568026-39568446	421	3.1	2.6	3.5	↓	-0.8	4.9E-04	6.9E-02
chr7:39568578-39569274	697	4.2	3.9	4.5	↓	-0.6	3.1E-04	5.2E-02
chr7:39601241-39601568	328	2.5	1.9	3.0	↓	-1.0	6.2E-04	8.0E-02
chr7:39663135-39663717	583	3.4	2.9	3.8	↓	-1.0	4.4E-06	2.1E-03
chr7:39697889-39698240	352	4.5	4.2	4.7	↓	-0.5	3.7E-04	5.8E-02
chr7:39720616-39721016	401	3.0	2.6	3.3	↓	-0.8	5.0E-04	7.0E-02
chr7:39947553-39948441	889	5.4	5.2	5.6	↓	-0.4	4.0E-04	6.2E-02
chr7:39974027-39974487	461	2.9	2.4	3.3	↓	-0.9	8.4E-05	2.1E-02
chr7:40049129-40049523	395	3.0	2.6	3.4	↓	-0.8	2.1E-04	4.1E-02
chr7:40367797-40368127	331	2.2	2.6	1.5	↑	1.1	1.8E-04	3.6E-02
chr7:4045662-4046196	535	4.7	4.4	4.9	↓	-0.5	6.6E-04	8.2E-02
chr7:4053725-4054361	637	3.5	3.8	3.1	↑	0.7	1.4E-04	3.0E-02
chr7:40690453-40691902	1450	5.1	4.9	5.3	↓	-0.4	3.8E-04	5.9E-02
chr7:41351738-41352038	301	2.0	1.4	2.5	↓	-1.1	4.2E-04	6.4E-02
chr7:41863600-41864336	737	3.4	3.8	2.8	↑	1.0	2.5E-06	1.3E-03
chr7:41897926-41899364	1439	6.3	6.6	5.9	↑	0.7	7.9E-09	1.1E-05
chr7:42038839-42039472	634	3.6	3.2	3.9	↓	-0.7	4.2E-04	6.4E-02
chr7:42100229-42100481	253	2.0	2.4	1.3	↑	1.1	4.1E-04	6.3E-02
chr7:42193036-42194044	1009	4.3	4.0	4.5	↓	-0.5	3.1E-04	5.2E-02
chr7:42997583-42997997	415	3.5	3.8	3.1	↑	0.6	8.2E-04	9.4E-02

chr7:43151145-43151433	289	1.8	2.3	1.1	↑	1.2	3.4E-04	5.5E-02
chr7:43179287-43179751	465	2.2	2.6	1.6	↑	1.0	6.1E-04	7.8E-02
chr7:43344237-43344714	478	3.4	2.9	3.7	↓	-0.9	4.0E-05	1.2E-02
chr7:43630250-43630915	666	3.6	3.9	3.2	↑	0.7	1.9E-04	3.8E-02
chr7:43697265-43697699	435	3.5	3.1	3.8	↓	-0.7	5.6E-04	7.4E-02
chr7:43764210-43765592	1383	5.4	5.5	5.2	↑	0.3	2.0E-04	3.9E-02
chr7:44069177-44070571	1395	6.1	6.2	6.0	↑	0.2	7.8E-04	9.1E-02
chr7:44617885-44618499	615	3.6	4.0	3.0	↑	1.0	3.6E-06	1.8E-03
chr7:45092531-45092897	367	2.5	2.9	1.8	↑	1.2	2.7E-05	9.0E-03
chr7:45389809-45391447	1639	5.5	5.8	5.3	↑	0.5	8.6E-05	2.1E-02
chr7:45446918-45447839	922	4.6	4.8	4.3	↑	0.5	2.7E-04	4.8E-02
chr7:45500561-45502138	1578	6.4	6.2	6.6	↓	-0.4	3.3E-04	5.4E-02
chr7:4579467-4580014	548	3.3	2.9	3.6	↓	-0.7	6.5E-04	8.1E-02
chr7:4580107-4582780	2674	6.8	6.6	6.9	↓	-0.3	1.9E-06	1.0E-03
chr7:4606143-4607271	1129	5.2	5.3	5.0	↑	0.4	4.0E-04	6.2E-02
chr7:46402578-46403304	727	4.7	4.4	4.9	↓	-0.5	5.8E-06	2.6E-03
chr7:46403648-46404110	463	3.6	3.1	4.0	↓	-0.9	1.4E-06	8.1E-04
chr7:46434737-46436860	2124	5.4	5.2	5.6	↓	-0.4	2.7E-05	9.0E-03
chr7:46821820-46822205	386	4.2	3.9	4.5	↓	-0.6	1.3E-04	2.9E-02
chr7:46850438-46850867	430	2.4	1.8	2.8	↓	-1.0	2.8E-04	4.9E-02
chr7:46996106-46996598	493	3.4	3.0	3.7	↓	-0.7	3.9E-04	6.0E-02
chr7:46999813-47000803	991	4.9	4.6	5.1	↓	-0.5	5.3E-05	1.5E-02
chr7:47000873-47001465	593	3.4	2.9	3.7	↓	-0.8	5.2E-05	1.5E-02
chr7:47069040-47069682	643	3.8	3.3	4.2	↓	-0.9	1.8E-06	9.7E-04
chr7:47205532-47205887	356	4.0	3.6	4.3	↓	-0.7	2.3E-04	4.3E-02
chr7:48298778-48300013	1236	4.3	4.1	4.6	↓	-0.5	7.1E-04	8.6E-02
chr7:48371316-48371536	221	2.0	1.2	2.5	↓	-1.3	5.3E-05	1.5E-02
chr7:48393250-48394044	795	3.4	2.8	3.8	↓	-1.0	1.2E-06	7.6E-04
chr7:48395027-48395737	711	4.2	3.6	4.6	↓	-1.0	1.8E-10	4.7E-07
chr7:48397322-48398195	874	4.5	4.1	4.8	↓	-0.8	3.8E-07	3.2E-04
chr7:48399078-48402439	3362	7.2	6.9	7.4	↓	-0.5	1.5E-13	7.4E-10
chr7:48408602-48409762	1161	5.3	5.0	5.5	↓	-0.5	1.4E-05	5.2E-03
chr7:48514878-48515296	419	2.9	3.4	2.3	↑	1.2	7.5E-07	5.5E-04
chr7:49635082-49635478	397	4.7	4.5	4.9	↓	-0.4	2.3E-04	4.3E-02
chr7:4969473-4970228	756	4.3	4.6	3.9	↑	0.7	4.5E-05	1.3E-02
chr7:50063123-50063323	201	2.0	2.5	1.3	↑	1.2	3.1E-04	5.2E-02
chr7:5010815-5011121	307	2.4	2.8	1.9	↑	1.0	5.9E-04	7.6E-02
chr7:50874965-50875247	283	2.6	2.0	2.9	↓	-0.9	5.2E-04	7.1E-02
chr7:52287430-52287654	225	2.5	1.9	2.9	↓	-1.0	3.2E-04	5.3E-02
chr7:5232234-5234768	2535	6.2	6.4	6.1	↑	0.3	5.9E-05	1.6E-02
chr7:52856733-52857183	451	2.8	3.2	2.1	↑	1.0	2.0E-04	3.9E-02
chr7:53143442-53144896	1455	5.0	4.7	5.3	↓	-0.6	9.9E-05	2.4E-02
chr7:53156865-53157616	752	3.6	2.9	4.0	↓	-1.1	7.9E-09	1.1E-05
chr7:53374104-53374370	267	2.5	2.9	2.0	↑	0.9	6.6E-04	8.2E-02
chr7:53408259-53408857	599	4.0	3.7	4.3	↓	-0.6	8.0E-04	9.2E-02
chr7:53869900-53870970	1071	4.8	4.4	5.1	↓	-0.7	2.0E-05	7.0E-03
chr7:53946787-53946993	207	2.1	2.6	1.5	↑	1.1	2.2E-04	4.2E-02

chr7:53992398-53992736	339	2.8	2.2	3.2	↓	-1.1	2.5E-05	8.5E-03
chr7:54212358-54213195	838	4.1	4.4	3.7	↑	0.7	1.2E-04	2.8E-02
chr7:54841614-54842254	641	3.7	4.0	3.3	↑	0.8	4.8E-05	1.4E-02
chr7:54909640-54910101	462	4.0	3.5	4.4	↓	-0.9	1.3E-06	7.9E-04
chr7:5494005-5494487	483	2.8	2.4	3.2	↓	-0.8	5.0E-04	7.0E-02
chr7:55368315-55370018	1704	6.3	6.1	6.4	↓	-0.3	2.7E-04	4.8E-02
chr7:55574663-55574923	261	2.2	1.3	2.7	↓	-1.4	1.5E-05	5.6E-03
chr7:55889215-55889831	617	3.5	3.1	3.8	↓	-0.8	7.2E-04	8.7E-02
chr7:56482030-56482211	182	1.7	0.9	2.3	↓	-1.3	1.3E-04	2.9E-02
chr7:56578347-56578722	376	3.2	3.6	2.9	↑	0.7	5.6E-04	7.4E-02
chr7:56586503-56587207	705	4.6	4.9	4.4	↑	0.5	3.6E-04	5.7E-02
chr7:56736920-56737375	456	3.5	3.9	3.0	↑	0.8	2.0E-05	6.9E-03
chr7:56857334-56857879	546	2.5	2.9	1.9	↑	1.0	3.2E-04	5.3E-02
chr7:56888162-56888494	333	3.5	3.1	3.8	↓	-0.7	1.4E-04	3.0E-02
chr7:57098280-57098776	497	4.1	3.8	4.3	↓	-0.5	8.2E-04	9.4E-02
chr7:57171096-57171535	440	4.7	4.4	4.9	↓	-0.5	1.2E-04	2.8E-02
chr7:57640736-57640981	246	2.4	1.8	2.8	↓	-1.0	4.9E-04	6.9E-02
chr7:58190185-58190750	566	3.6	3.2	3.9	↓	-0.7	6.1E-05	1.6E-02
chr7:58316910-58317494	585	3.0	2.2	3.5	↓	-1.3	4.2E-06	2.0E-03
chr7:58517050-58517235	186	1.8	1.0	2.4	↓	-1.4	7.5E-05	1.9E-02
chr7:58678525-58679046	522	5.0	4.8	5.2	↓	-0.4	4.8E-04	6.8E-02
chr7:58818074-58821360	3287	7.0	6.8	7.1	↓	-0.2	3.6E-05	1.1E-02
chr7:59312250-59312428	179	1.4	1.9	0.5	↑	1.4	3.2E-04	5.3E-02
chr7:5945622-5945803	182	1.5	0.7	2.0	↓	-1.3	4.5E-04	6.7E-02
chr7:60027530-60027887	358	2.3	1.8	2.7	↓	-1.0	5.2E-04	7.1E-02
chr7:60238338-60238659	322	3.6	3.3	3.9	↓	-0.6	7.9E-04	9.2E-02
chr7:60570030-60570464	435	5.4	5.2	5.5	↓	-0.4	6.1E-04	7.8E-02
chr7:60800825-60801142	318	3.7	3.3	4.1	↓	-0.7	5.0E-05	1.4E-02
chr7:61542339-61542568	230	2.4	1.6	3.0	↓	-1.3	6.5E-07	4.9E-04
chr7:61542692-61543032	341	3.7	3.0	4.1	↓	-1.1	1.2E-07	1.2E-04
chr7:61545868-61546234	367	3.2	2.5	3.7	↓	-1.2	1.2E-07	1.2E-04
chr7:61916287-61916647	361	2.0	1.4	2.5	↓	-1.1	6.6E-04	8.2E-02
chr7:63872159-63872384	226	1.9	2.4	1.2	↑	1.2	4.8E-04	6.8E-02
chr7:6420501-6420973	473	3.2	2.7	3.5	↓	-0.8	6.7E-04	8.2E-02
chr7:64899565-64900887	1323	4.5	4.8	4.1	↑	0.7	3.5E-06	1.8E-03
chr7:64924834-64925102	269	1.6	2.2	0.8	↑	1.4	1.3E-04	2.8E-02
chr7:64984806-64985231	426	2.9	3.4	2.1	↑	1.2	9.0E-06	3.7E-03
chr7:6501570-6502042	473	5.3	5.0	5.5	↓	-0.5	7.6E-04	8.9E-02
chr7:65199644-65199891	248	2.2	2.7	1.6	↑	1.1	1.6E-04	3.3E-02
chr7:65426637-65427069	433	3.9	4.2	3.3	↑	0.9	2.3E-06	1.2E-03
chr7:66522123-66522570	448	3.2	2.7	3.6	↓	-0.9	1.3E-04	2.9E-02
chr7:66594563-66595065	503	3.1	2.6	3.4	↓	-0.9	1.9E-04	3.8E-02
chr7:66598228-66598556	329	2.4	1.7	2.9	↓	-1.2	2.2E-05	7.5E-03
chr7:66796380-66796950	571	3.4	2.9	3.7	↓	-0.8	1.7E-04	3.5E-02
chr7:67736596-67736826	231	2.4	1.6	2.8	↓	-1.2	1.3E-05	4.9E-03
chr7:68074593-68074841	249	2.4	1.8	2.8	↓	-1.0	7.1E-04	8.6E-02
chr7:6878081-6878386	306	2.6	1.8	3.1	↓	-1.3	7.2E-07	5.4E-04

chr7:68880-69616	737	5.5	5.4	5.7	↓	-0.3	5.8E-04	7.6E-02
chr7:69634243-69634641	399	3.0	3.5	2.3	↑	1.2	2.3E-07	2.0E-04
chr7:69730532-69730730	199	1.9	2.3	1.3	↑	1.1	7.0E-04	8.6E-02
chr7:70018206-70019949	1744	6.5	6.6	6.4	↑	0.3	8.0E-05	2.0E-02
chr7:7005129-7005588	460	3.0	2.5	3.4	↓	-0.8	5.9E-04	7.7E-02
chr7:7019167-7019481	315	2.2	1.3	2.7	↓	-1.4	5.8E-06	2.6E-03
chr7:70469087-70469393	307	2.1	2.6	1.5	↑	1.1	7.7E-04	9.0E-02
chr7:70647794-70648108	315	3.4	3.0	3.7	↓	-0.7	3.5E-04	5.6E-02
chr7:70666341-70667030	690	3.8	4.2	3.3	↑	0.8	4.3E-05	1.3E-02
chr7:70668133-70668543	411	3.2	3.7	2.5	↑	1.2	2.1E-07	1.9E-04
chr7:70668590-70669811	1222	5.0	5.7	3.8	↑	1.9	2.7E-21	6.6E-17
chr7:70675211-70675473	263	2.9	3.5	1.9	↑	1.5	9.1E-10	1.9E-06
chr7:70676401-70676615	215	2.4	3.0	1.5	↑	1.5	7.9E-07	5.6E-04
chr7:70677656-70678397	742	4.1	4.4	3.8	↑	0.6	1.9E-05	6.9E-03
chr7:70678658-70678915	258	2.1	2.6	1.4	↑	1.2	1.3E-04	2.8E-02
chr7:70680259-70680451	193	1.7	2.2	1.0	↑	1.2	8.1E-04	9.4E-02
chr7:70682199-70683391	1193	5.7	6.4	4.5	↑	1.9	1.1E-19	1.3E-15
chr7:70683424-70684769	1346	5.3	6.0	4.1	↑	1.9	6.5E-39	3.2E-34
chr7:70686097-70686945	849	4.1	4.7	3.1	↑	1.5	1.9E-17	1.5E-13
chr7:70686964-70688585	1622	6.3	6.5	6.0	↑	0.6	1.9E-09	3.3E-06
chr7:70689301-70690003	703	3.9	4.5	2.8	↑	1.7	5.9E-12	2.1E-08
chr7:70697132-70697719	588	2.7	3.1	2.2	↑	0.9	2.8E-04	4.8E-02
chr7:70700128-70700767	640	3.5	4.2	2.3	↑	1.9	4.8E-16	3.3E-12
chr7:70702104-70702730	627	3.2	3.6	2.8	↑	0.8	1.8E-04	3.7E-02
chr7:70703456-70703982	527	3.6	4.0	3.1	↑	0.9	9.6E-07	6.5E-04
chr7:70754565-70754846	282	2.3	2.8	1.7	↑	1.1	1.0E-04	2.5E-02
chr7:70903072-70903435	364	3.3	2.8	3.7	↓	-0.9	3.5E-05	1.1E-02
chr7:71095615-71096017	403	2.6	3.0	2.1	↑	0.9	4.7E-04	6.8E-02
chr7:71775467-71776192	726	4.4	4.2	4.7	↓	-0.5	1.7E-04	3.5E-02
chr7:72349992-72350542	551	2.7	3.1	2.2	↑	0.9	2.6E-04	4.7E-02
chr7:736108-740739	4632	8.0	7.9	8.1	↓	-0.2	1.6E-04	3.3E-02
chr7:7450151-7450471	321	3.0	2.5	3.3	↓	-0.8	6.1E-04	7.8E-02
chr7:7612796-7613905	1110	5.6	5.4	5.7	↓	-0.3	7.0E-04	8.6E-02
chr7:7672471-7672706	236	1.9	1.2	2.4	↓	-1.2	4.5E-04	6.6E-02
chr7:78883362-78884051	690	3.8	3.4	4.1	↓	-0.7	4.3E-05	1.3E-02
chr7:78886084-78886439	356	2.9	2.3	3.4	↓	-1.1	9.2E-06	3.8E-03
chr7:79106903-79107102	200	2.3	1.6	2.7	↓	-1.2	6.7E-05	1.7E-02
chr7:79570797-79571107	311	3.6	3.9	3.3	↑	0.7	3.2E-04	5.2E-02
chr7:79851080-79851303	224	2.1	1.4	2.5	↓	-1.1	5.1E-04	7.0E-02
chr7:79896638-79897113	476	2.9	2.2	3.3	↓	-1.1	1.3E-04	2.9E-02
chr7:80250168-80251713	1546	5.5	5.3	5.8	↓	-0.5	3.1E-04	5.2E-02
chr7:80255116-80255382	267	2.0	2.4	1.4	↑	1.1	5.5E-04	7.4E-02
chr7:80816004-80816285	282	3.2	2.5	3.7	↓	-1.2	2.9E-08	3.7E-05
chr7:81322908-81323632	725	6.5	6.3	6.6	↓	-0.3	9.9E-05	2.4E-02
chr7:81439627-81439916	290	3.0	3.4	2.4	↑	1.0	1.2E-04	2.8E-02
chr7:82830337-82830770	434	3.5	3.1	3.8	↓	-0.7	5.6E-04	7.4E-02
chr7:8297099-8297636	538	4.2	3.8	4.5	↓	-0.7	8.4E-05	2.1E-02

chr7:8301163-8301390	228	1.9	1.1	2.4	↓	-1.2	1.1E-04	2.6E-02
chr7:83439633-83440045	413	2.8	1.7	3.4	↓	-1.7	4.3E-11	1.3E-07
chr7:83440616-83441028	413	2.7	1.0	3.5	↓	-2.5	5.9E-18	5.8E-14
chr7:84163017-84163260	244	1.7	1.0	2.2	↓	-1.2	8.8E-04	9.9E-02
chr7:86541956-86542442	487	3.5	3.1	3.9	↓	-0.8	7.9E-04	9.2E-02
chr7:86648521-86648818	298	2.4	2.8	1.9	↑	0.9	7.8E-04	9.1E-02
chr7:87082424-87082827	404	2.8	2.3	3.1	↓	-0.8	5.3E-04	7.2E-02
chr7:877257-879163	1907	6.0	5.9	6.2	↓	-0.3	5.5E-04	7.4E-02
chr7:87837469-87838251	783	4.0	3.5	4.3	↓	-0.8	6.2E-06	2.8E-03
chr7:88308087-88308341	255	2.3	1.6	2.8	↓	-1.3	2.0E-05	7.0E-03
chr7:88362518-88363171	654	3.9	3.5	4.2	↓	-0.7	1.5E-04	3.1E-02
chr7:88904-89795	892	5.7	5.4	5.9	↓	-0.5	2.7E-08	3.4E-05
chr7:88944815-88945520	706	3.3	3.9	2.4	↑	1.5	4.2E-07	3.5E-04
chr7:88946567-88946973	407	2.5	2.9	1.9	↑	1.0	8.5E-04	9.7E-02
chr7:88947104-88948713	1610	5.3	5.8	4.6	↑	1.2	1.0E-12	4.1E-09
chr7:88948770-88949332	563	3.5	3.9	2.9	↑	1.0	1.0E-06	6.8E-04
chr7:88951007-88951843	837	5.0	5.2	4.7	↑	0.6	6.6E-05	1.7E-02
chr7:88960433-88962591	2159	5.3	5.0	5.6	↓	-0.7	5.7E-09	8.7E-06
chr7:88963505-88964166	662	4.1	3.8	4.4	↓	-0.6	3.7E-05	1.2E-02
chr7:88974981-88975449	469	3.4	3.9	2.5	↑	1.4	6.3E-10	1.4E-06
chr7:88975502-88976275	774	3.6	4.3	2.3	↑	2.0	1.2E-20	2.0E-16
chr7:89074938-89075206	269	3.4	3.0	3.7	↓	-0.7	2.7E-04	4.8E-02
chr7:8956747-8958236	1490	5.7	5.5	5.8	↓	-0.3	3.6E-04	5.7E-02
chr7:89760239-89760589	351	2.5	2.9	1.8	↑	1.1	5.4E-05	1.5E-02
chr7:89799744-89800276	533	3.8	3.5	4.1	↓	-0.6	5.6E-04	7.4E-02
chr7:89808472-89808925	454	3.8	4.1	3.4	↑	0.7	5.5E-04	7.4E-02
chr7:89889959-89890358	400	3.6	3.9	3.2	↑	0.7	1.4E-04	3.0E-02
chr7:9042170-9042624	455	3.1	2.7	3.4	↓	-0.7	6.7E-04	8.3E-02
chr7:9045544-9046549	1006	5.3	5.1	5.4	↓	-0.4	1.3E-04	2.8E-02
chr7:90517283-90517591	309	3.4	2.9	3.7	↓	-0.8	1.7E-04	3.5E-02
chr7:90750697-90751043	347	3.7	3.4	4.0	↓	-0.7	2.5E-04	4.6E-02
chr7:9100937-9101488	552	3.6	3.1	3.9	↓	-0.9	4.1E-05	1.3E-02
chr7:91138687-91138968	282	2.3	2.8	1.7	↑	1.1	3.3E-04	5.3E-02
chr7:91295723-91296497	775	4.7	4.4	4.9	↓	-0.5	2.2E-04	4.2E-02
chr7:9154385-9154824	440	3.1	2.5	3.5	↓	-0.9	1.8E-04	3.6E-02
chr7:91841629-91842416	788	3.8	4.1	3.4	↑	0.7	2.9E-05	9.5E-03
chr7:92327614-92328101	488	3.2	3.5	2.7	↑	0.9	4.2E-05	1.3E-02
chr7:9244929-9245573	645	5.0	4.7	5.2	↓	-0.5	2.5E-04	4.6E-02
chr7:9358452-9359229	778	4.2	3.8	4.5	↓	-0.7	8.8E-06	3.7E-03
chr7:93915982-93916236	255	2.1	1.5	2.5	↓	-1.1	4.6E-04	6.8E-02
chr7:93917267-93917687	421	4.1	3.8	4.4	↓	-0.6	1.6E-05	6.0E-03
chr7:95321179-95321413	235	2.9	2.3	3.3	↓	-1.0	1.9E-05	6.9E-03
chr7:96230540-96230876	337	2.7	1.9	3.2	↓	-1.3	9.2E-08	9.9E-05
chr7:96372928-96373199	272	2.1	1.4	2.6	↓	-1.1	2.9E-04	4.9E-02
chr7:98308604-98309108	505	3.2	3.5	2.8	↑	0.7	7.4E-04	8.8E-02
chr7:98523822-98524713	892	5.0	5.2	4.7	↑	0.6	7.7E-06	3.3E-03
chr7:98604515-98605719	1205	4.5	4.0	4.9	↓	-0.9	7.8E-08	8.7E-05

chr7:98606439-98607521	1083	5.4	5.0	5.8	↓	-0.8	1.0E-11	3.3E-08
chr7:98611466-98612932	1467	6.5	6.3	6.6	↓	-0.3	4.6E-04	6.8E-02
chr7:98780975-98781806	832	4.9	4.7	5.1	↓	-0.4	8.7E-04	9.8E-02
chr7:9882651-9883230	580	4.1	3.6	4.4	↓	-0.8	1.7E-06	9.4E-04
chr7:99743357-99744418	1062	4.3	4.6	4.0	↑	0.6	3.6E-04	5.7E-02
chr7:99877461-99877772	312	2.6	3.0	2.1	↑	0.9	2.1E-04	4.0E-02
chr8:10341478-10341776	299	2.2	1.6	2.7	↓	-1.1	1.7E-04	3.9E-02
chr8:103466439-103467096	658	3.9	3.5	4.2	↓	-0.7	4.3E-04	7.0E-02
chr8:103530299-103530707	409	2.9	2.4	3.2	↓	-0.8	3.4E-04	6.1E-02
chr8:103639669-103640114	446	2.8	2.3	3.2	↓	-0.9	4.0E-04	6.7E-02
chr8:103641626-103642106	481	2.5	1.5	3.1	↓	-1.6	1.1E-08	1.4E-05
chr8:103643254-103644304	1051	4.2	3.6	4.6	↓	-1.0	2.6E-09	4.0E-06
chr8:103645981-103647253	1273	5.1	4.4	5.5	↓	-1.1	5.1E-20	2.9E-16
chr8:103647507-103647985	479	2.3	1.5	2.8	↓	-1.3	1.0E-05	5.0E-03
chr8:103648394-103649009	616	2.6	1.7	3.1	↓	-1.4	2.0E-07	1.9E-04
chr8:103650915-103651914	1000	4.8	4.5	5.0	↓	-0.5	5.5E-04	8.1E-02
chr8:103720131-103720686	556	3.0	2.4	3.4	↓	-1.0	7.7E-06	3.9E-03
chr8:105060262-105061513	1252	4.3	3.9	4.6	↓	-0.7	2.1E-04	4.4E-02
chr8:106588425-106588781	357	2.8	2.3	3.2	↓	-0.9	8.4E-05	2.3E-02
chr8:107151022-107151461	440	2.4	1.5	3.0	↓	-1.5	3.2E-07	2.8E-04
chr8:107170438-107171116	679	4.0	3.7	4.3	↓	-0.6	7.6E-04	9.9E-02
chr8:107182224-107182582	359	2.3	1.6	2.8	↓	-1.2	1.2E-05	5.3E-03
chr8:107279159-107279517	359	2.4	2.9	1.6	↑	1.2	1.1E-05	5.1E-03
chr8:107329099-107329700	602	2.8	3.2	2.3	↑	0.9	1.6E-04	3.6E-02
chr8:10740451-10741404	954	3.8	3.5	4.1	↓	-0.6	3.6E-04	6.3E-02
chr8:107445518-107446186	669	3.0	3.5	2.4	↑	1.1	1.2E-06	8.3E-04
chr8:107661420-107661745	326	2.4	2.9	1.5	↑	1.4	6.1E-07	4.5E-04
chr8:107669934-107671169	1236	4.4	4.7	4.1	↑	0.6	5.9E-04	8.7E-02
chr8:107713450-107713753	304	2.0	2.5	1.2	↑	1.3	1.3E-04	3.2E-02
chr8:107797006-107797436	431	2.4	2.8	1.9	↑	0.9	7.2E-04	9.6E-02
chr8:107856584-107857147	564	3.2	3.6	2.6	↑	1.0	6.6E-05	1.9E-02
chr8:108218355-108218908	554	3.3	2.8	3.6	↓	-0.9	3.4E-05	1.2E-02
chr8:108307874-108308740	867	3.7	4.0	3.4	↑	0.6	5.0E-04	7.7E-02
chr8:108309471-108309867	397	2.6	3.0	2.1	↑	0.9	4.4E-04	7.1E-02
chr8:108413591-108414050	460	2.9	3.2	2.4	↑	0.8	6.5E-04	9.0E-02
chr8:108414103-108414913	811	4.9	5.2	4.5	↑	0.7	1.2E-05	5.5E-03
chr8:112318027-112318680	654	3.7	3.2	4.0	↓	-0.7	7.4E-05	2.1E-02
chr8:112404193-112405659	1467	4.8	4.2	5.2	↓	-1.0	1.5E-10	2.9E-07
chr8:112406066-112406590	525	2.6	2.0	3.0	↓	-1.1	3.6E-05	1.2E-02
chr8:112408978-112409321	344	1.8	1.1	2.3	↓	-1.2	3.6E-04	6.2E-02
chr8:114429377-114429823	447	2.0	2.4	1.3	↑	1.2	5.1E-04	7.8E-02
chr8:115096761-115096965	205	1.6	0.7	2.1	↓	-1.4	1.3E-04	3.2E-02
chr8:115965146-115965663	518	3.0	3.3	2.5	↑	0.8	6.1E-04	8.8E-02
chr8:116567418-116567773	356	2.6	2.0	3.1	↓	-1.0	4.0E-05	1.3E-02
chr8:116721249-116721551	303	2.7	2.0	3.1	↓	-1.1	1.8E-05	7.2E-03
chr8:116785606-116786578	973	3.8	3.4	4.1	↓	-0.6	3.2E-04	5.8E-02
chr8:117346713-117347246	534	3.9	4.3	3.5	↑	0.7	5.9E-05	1.7E-02

chr8:117859526-117859873	348	3.5	3.0	3.9	↓	-1.0	3.3E-06	2.1E-03
chr8:119638101-119638487	387	2.4	2.8	1.8	↑	1.0	6.0E-04	8.8E-02
chr8:119909380-119913356	3977	6.3	7.0	4.9	↑	2.1	9.1E-24	1.1E-19
chr8:119913677-119914112	436	2.3	2.8	1.5	↑	1.3	9.6E-06	4.8E-03
chr8:119914134-119914526	393	2.1	2.7	0.8	↑	2.0	6.4E-09	9.3E-06
chr8:119914573-119915904	1332	4.5	5.2	3.1	↑	2.1	1.3E-28	4.5E-24
chr8:119918692-119919119	428	2.2	2.7	1.3	↑	1.4	4.1E-06	2.5E-03
chr8:119921081-119922011	931	3.0	3.6	1.8	↑	1.8	3.3E-13	1.1E-09
chr8:119922140-119922911	772	3.9	4.6	2.5	↑	2.1	7.0E-27	1.2E-22
chr8:119923238-119925564	2327	6.2	6.8	4.9	↑	1.9	1.7E-23	1.5E-19
chr8:119927387-119928645	1259	4.3	5.0	3.1	↑	1.9	1.2E-18	6.0E-15
chr8:119928836-119929444	609	2.6	3.1	1.7	↑	1.5	2.4E-07	2.2E-04
chr8:119931928-119932853	926	3.6	4.2	2.6	↑	1.6	1.1E-12	2.6E-09
chr8:120643851-120644276	426	2.5	2.9	1.9	↑	1.0	8.6E-05	2.3E-02
chr8:121797067-121797596	530	3.8	3.3	4.2	↓	-0.9	5.6E-07	4.3E-04
chr8:122189965-122190493	529	5.3	4.7	5.8	↓	-1.1	1.7E-12	3.7E-09
chr8:122208197-122210072	1876	6.3	6.1	6.4	↓	-0.3	1.3E-04	3.2E-02
chr8:122435660-122436075	416	2.6	3.0	2.1	↑	0.9	5.8E-04	8.6E-02
chr8:123573185-123573495	311	2.3	2.7	1.8	↑	0.9	6.4E-04	9.0E-02
chr8:12452081-12452639	559	3.5	3.1	3.8	↓	-0.6	7.2E-04	9.6E-02
chr8:125499301-125499621	321	2.5	1.9	3.0	↓	-1.0	1.0E-04	2.6E-02
chr8:12617080-12617416	337	2.4	1.8	2.9	↓	-1.2	1.5E-04	3.6E-02
chr8:128043511-128044158	648	2.8	2.1	3.2	↓	-1.1	2.1E-05	7.9E-03
chr8:128845027-128845728	702	3.4	3.0	3.8	↓	-0.7	2.3E-04	4.7E-02
chr8:129865815-129866771	957	5.8	5.4	6.1	↓	-0.7	5.4E-13	1.5E-09
chr8:130889139-130889506	368	3.0	2.6	3.4	↓	-0.8	1.9E-04	4.2E-02
chr8:130924501-130924939	439	2.8	3.2	2.1	↑	1.2	2.1E-05	8.0E-03
chr8:131829071-131829893	823	3.9	3.6	4.2	↓	-0.6	4.7E-04	7.4E-02
chr8:13356589-13357011	423	3.1	3.6	2.3	↑	1.3	4.1E-08	4.6E-05
chr8:133596341-133597016	676	3.6	3.9	3.2	↑	0.7	6.1E-04	8.8E-02
chr8:133702599-133702882	284	2.2	2.7	1.4	↑	1.3	6.3E-05	1.8E-02
chr8:133956235-133956564	330	2.2	1.4	2.6	↓	-1.2	1.1E-04	2.9E-02
chr8:133958627-133959671	1045	3.4	2.9	3.8	↓	-0.9	6.0E-06	3.2E-03
chr8:134458505-134459955	1451	4.6	4.8	4.4	↑	0.5	2.7E-04	5.1E-02
chr8:135352513-135353003	491	3.1	2.4	3.6	↓	-1.3	1.4E-07	1.4E-04
chr8:135545705-135546027	323	2.4	2.8	1.7	↑	1.0	5.3E-04	8.0E-02
chr8:136289528-136290362	835	3.4	3.9	2.8	↑	1.0	1.1E-05	5.1E-03
chr8:138063743-138064304	562	2.4	2.9	1.9	↑	1.0	4.8E-04	7.5E-02
chr8:138750198-138750937	740	3.2	2.7	3.5	↓	-0.8	3.6E-04	6.3E-02
chr8:139306940-139307452	513	2.5	2.9	1.9	↑	1.0	2.8E-04	5.3E-02
chr8:139343775-139344104	330	2.2	2.7	1.6	↑	1.1	3.9E-04	6.6E-02
chr8:140634127-140634891	765	2.4	2.8	1.8	↑	1.0	3.9E-04	6.6E-02
chr8:141052634-141053102	469	3.0	2.4	3.4	↓	-1.1	6.6E-06	3.5E-03
chr8:141535057-141535698	642	3.4	3.8	3.0	↑	0.7	8.2E-05	2.2E-02
chr8:141537317-141537837	521	3.3	3.7	2.6	↑	1.1	1.8E-04	4.0E-02
chr8:141547756-141548265	510	2.9	3.3	2.4	↑	1.0	6.6E-05	1.9E-02
chr8:141634520-141635023	504	2.8	3.1	2.3	↑	0.8	5.1E-04	7.7E-02

chr8:141649222-141649580	359	2.1	2.5	1.5	↑	1.0	6.0E-04	8.8E-02
chr8:141658857-141659556	700	3.6	3.9	3.1	↑	0.8	3.8E-04	6.5E-02
chr8:141819517-141819792	276	1.9	0.9	2.5	↓	-1.6	1.8E-05	7.3E-03
chr8:142304769-142305733	965	4.1	3.6	4.5	↓	-0.8	1.8E-06	1.2E-03
chr8:142307861-142309296	1436	4.7	4.4	5.0	↓	-0.6	5.3E-07	4.1E-04
chr8:142344110-142344703	594	3.3	2.9	3.7	↓	-0.8	2.7E-05	9.6E-03
chr8:142693376-142693735	360	1.9	1.2	2.4	↓	-1.2	1.5E-04	3.6E-02
chr8:143215002-143215969	968	5.2	4.9	5.4	↓	-0.5	5.7E-05	1.7E-02
chr8:143320422-143321877	1456	5.5	5.3	5.7	↓	-0.4	6.5E-04	9.0E-02
chr8:143677657-143678235	579	3.8	3.3	4.1	↓	-0.8	1.3E-05	5.6E-03
chr8:144198663-144199533	871	3.7	3.3	4.0	↓	-0.7	2.3E-04	4.8E-02
chr8:144914386-144915741	1356	5.2	4.9	5.5	↓	-0.5	1.3E-05	5.6E-03
chr8:145350121-145350963	843	4.3	4.0	4.6	↓	-0.7	2.3E-04	4.7E-02
chr8:145839476-145840541	1066	4.4	4.1	4.6	↓	-0.5	4.9E-04	7.6E-02
chr8:145893404-145894372	969	5.8	5.6	6.1	↓	-0.5	5.3E-04	8.0E-02
chr8:145913523-145916640	3118	6.9	6.8	7.1	↓	-0.2	1.2E-04	2.9E-02
chr8:145992252-145993462	1211	6.1	5.9	6.3	↓	-0.3	2.9E-04	5.5E-02
chr8:146108422-146109183	762	4.0	3.6	4.3	↓	-0.7	1.4E-05	5.9E-03
chr8:146179096-146180471	1376	5.2	4.8	5.4	↓	-0.6	4.4E-05	1.4E-02
chr8:146224623-146226774	2152	7.1	7.0	7.2	↓	-0.3	1.9E-04	4.2E-02
chr8:146583665-146584413	749	5.6	5.4	5.8	↓	-0.4	1.5E-04	3.5E-02
chr8:146647957-146648208	252	2.5	2.9	1.9	↑	1.0	7.1E-04	9.6E-02
chr8:146736553-146737805	1253	6.1	6.0	6.3	↓	-0.3	6.7E-04	9.1E-02
chr8:146999656-146999980	325	3.2	2.8	3.5	↓	-0.7	6.3E-04	8.9E-02
chr8:147165658-147166035	378	1.8	2.3	1.1	↑	1.2	3.5E-04	6.2E-02
chr8:147374817-147375606	790	5.2	5.0	5.5	↓	-0.5	3.3E-07	2.9E-04
chr8:147515659-147516162	504	3.1	2.6	3.5	↓	-0.9	2.5E-05	9.3E-03
chr8:147525471-147526073	603	3.9	3.5	4.1	↓	-0.6	6.3E-04	8.9E-02
chr8:147529721-147530224	504	5.4	5.1	5.7	↓	-0.5	1.3E-05	5.5E-03
chr8:147609985-147611006	1022	4.7	4.3	5.0	↓	-0.7	2.3E-07	2.2E-04
chr8:14856759-14857160	402	2.7	3.2	2.1	↑	1.1	7.0E-05	2.0E-02
chr8:14897080-14898309	1230	3.8	4.1	3.3	↑	0.8	7.9E-05	2.2E-02
chr8:14935361-14936049	689	3.7	4.0	3.4	↑	0.6	4.3E-04	6.9E-02
chr8:14957441-14957882	442	2.6	3.0	1.8	↑	1.2	4.2E-06	2.5E-03
chr8:15000304-15001327	1024	3.7	2.8	4.2	↓	-1.4	5.9E-13	1.5E-09
chr8:15151013-15151279	267	1.6	2.2	0.8	↑	1.3	2.7E-04	5.1E-02
chr8:15491679-15492244	566	3.4	2.7	3.8	↓	-1.1	3.6E-08	4.2E-05
chr8:15705043-15705426	384	2.3	2.7	1.7	↑	1.1	2.6E-04	5.0E-02
chr8:16720913-16721360	448	3.0	3.4	2.6	↑	0.8	6.4E-04	9.0E-02
chr8:16978949-16979261	313	1.7	2.3	0.6	↑	1.7	3.3E-05	1.1E-02
chr8:1704648-1708306	3659	6.9	6.8	7.0	↓	-0.3	1.9E-04	4.2E-02
chr8:1719411-1720029	619	4.9	4.7	5.1	↓	-0.4	2.5E-04	4.9E-02
chr8:18282626-18283173	548	4.4	3.7	4.8	↓	-1.2	5.3E-13	1.5E-09
chr8:18623267-18625589	2323	5.8	5.2	6.2	↓	-0.9	5.4E-23	3.7E-19
chr8:18686418-18686849	432	3.6	2.8	4.2	↓	-1.4	1.8E-13	6.2E-10
chr8:18824281-18825609	1329	5.7	5.3	6.0	↓	-0.7	1.1E-10	2.3E-07
chr8:18959766-18960622	857	3.9	3.4	4.3	↓	-0.9	4.6E-07	3.8E-04

chr8:19508693-19509302	610	3.4	3.0	3.8	↓	-0.7	4.1E-04	6.8E-02
chr8:19876072-19876799	728	3.9	3.6	4.2	↓	-0.6	1.6E-04	3.8E-02
chr8:19981541-19981927	387	2.3	1.3	2.9	↓	-1.5	5.1E-07	4.1E-04
chr8:19991181-19992216	1036	4.4	4.7	4.0	↑	0.6	3.4E-05	1.1E-02
chr8:20364547-20365963	1417	5.8	5.6	5.9	↓	-0.3	4.4E-04	7.1E-02
chr8:20453584-20453909	326	2.6	3.0	2.1	↑	0.9	5.9E-04	8.7E-02
chr8:20562747-20563292	546	2.8	2.2	3.3	↓	-1.0	1.0E-04	2.6E-02
chr8:20565295-20566122	828	5.0	4.7	5.2	↓	-0.5	2.6E-04	5.1E-02
chr8:20641350-20641691	342	2.9	2.2	3.3	↓	-1.1	1.1E-05	5.1E-03
chr8:21109712-21111424	1713	5.1	4.8	5.3	↓	-0.5	3.9E-05	1.3E-02
chr8:24324018-24325163	1146	4.9	4.0	5.5	↓	-1.4	1.3E-16	5.8E-13
chr8:24367878-24368410	533	4.2	3.6	4.7	↓	-1.0	1.0E-07	1.1E-04
chr8:24436851-24437555	705	3.4	3.0	3.8	↓	-0.8	3.0E-04	5.5E-02
chr8:25263083-25263411	329	2.4	1.7	2.9	↓	-1.3	4.1E-06	2.5E-03
chr8:2670849-2671245	397	2.1	1.4	2.6	↓	-1.2	8.0E-05	2.2E-02
chr8:2679584-2680122	539	3.4	2.9	3.8	↓	-0.9	1.1E-05	5.1E-03
chr8:29313294-29313733	440	2.6	3.0	2.0	↑	1.0	4.2E-05	1.3E-02
chr8:30013641-30013996	356	2.0	1.3	2.5	↓	-1.1	3.5E-04	6.2E-02
chr8:30133849-30134319	471	2.8	2.2	3.2	↓	-1.0	9.8E-05	2.5E-02
chr8:30210170-30211930	1761	4.5	4.9	4.1	↑	0.8	9.2E-09	1.2E-05
chr8:30212028-30212595	568	2.9	3.5	2.0	↑	1.5	4.9E-07	4.0E-04
chr8:30298221-30299050	830	3.8	4.1	3.4	↑	0.7	7.2E-04	9.6E-02
chr8:30396718-30397005	288	2.0	2.5	1.3	↑	1.2	2.0E-04	4.3E-02
chr8:3089226-3090464	1239	5.0	4.7	5.2	↓	-0.5	1.9E-04	4.2E-02
chr8:31209799-31210665	867	3.3	3.7	2.8	↑	0.9	9.4E-06	4.7E-03
chr8:31266730-31267056	327	2.3	1.6	2.7	↓	-1.1	1.6E-04	3.6E-02
chr8:32191722-32192075	354	2.9	2.3	3.3	↓	-1.0	4.9E-05	1.5E-02
chr8:32280484-32280910	427	1.9	2.3	1.2	↑	1.2	3.1E-04	5.6E-02
chr8:32608394-32608897	504	3.0	3.4	2.4	↑	1.0	5.3E-05	1.6E-02
chr8:32992466-32992748	283	1.9	2.4	1.1	↑	1.3	5.8E-05	1.7E-02
chr8:33326083-33327212	1130	5.2	4.6	5.6	↓	-1.0	3.9E-15	1.5E-11
chr8:34112326-34112783	458	3.2	2.6	3.6	↓	-1.0	5.4E-05	1.6E-02
chr8:3538552-3543122	4571	7.7	7.6	7.8	↓	-0.2	2.4E-05	9.0E-03
chr8:36135810-36136163	354	3.3	3.7	2.9	↑	0.7	1.6E-04	3.6E-02
chr8:3639102-3642367	3266	7.4	7.3	7.6	↓	-0.3	2.0E-07	1.9E-04
chr8:3659088-3660888	1801	6.0	5.8	6.2	↓	-0.4	3.3E-06	2.1E-03
chr8:3691197-3692232	1036	5.3	5.1	5.4	↓	-0.4	6.5E-04	9.0E-02
chr8:3697243-3698522	1280	4.8	4.6	5.0	↓	-0.4	3.0E-04	5.5E-02
chr8:3782532-3785397	2866	7.1	7.0	7.2	↓	-0.2	6.0E-04	8.7E-02
chr8:3842705-3844034	1330	5.5	5.3	5.7	↓	-0.3	7.2E-04	9.6E-02
chr8:39670014-39670469	456	3.0	3.4	2.5	↑	0.8	2.4E-04	4.9E-02
chr8:3973166-3973645	480	3.5	3.1	3.8	↓	-0.7	5.1E-04	7.7E-02
chr8:39757249-39757647	399	2.7	2.1	3.2	↓	-1.0	4.6E-05	1.5E-02
chr8:3996191-4000594	4404	7.9	7.8	8.0	↓	-0.2	3.9E-10	7.1E-07
chr8:40578382-40579058	677	3.1	2.7	3.5	↓	-0.7	4.7E-04	7.5E-02
chr8:41173602-41174075	474	2.1	2.6	1.4	↑	1.2	2.1E-04	4.4E-02
chr8:41256424-41257051	628	2.8	2.2	3.3	↓	-1.0	2.1E-05	7.9E-03

chr8:41327380-41328066	687	3.5	3.8	3.1	↑	0.7	4.8E-04	7.5E-02
chr8:41606579-41607332	754	3.1	2.7	3.5	↓	-0.8	3.2E-04	5.8E-02
chr8:4210200-4215047	4848	7.6	7.5	7.7	↓	-0.2	5.0E-06	2.8E-03
chr8:42923290-42924231	942	5.4	5.2	5.6	↓	-0.3	2.2E-04	4.7E-02
chr8:43019623-43021251	1629	4.8	5.0	4.6	↑	0.4	1.3E-04	3.2E-02
chr8:43123743-43124273	531	4.2	3.9	4.4	↓	-0.6	7.5E-05	2.1E-02
chr8:4316855-4319706	2852	7.0	7.2	6.7	↑	0.5	2.8E-06	1.9E-03
chr8:43918441-43919012	572	3.0	3.4	2.3	↑	1.1	5.3E-06	2.9E-03
chr8:4488508-4488940	433	2.1	2.6	1.5	↑	1.1	5.0E-04	7.7E-02
chr8:4501781-4502470	690	4.2	3.8	4.5	↓	-0.7	1.0E-05	5.1E-03
chr8:45026324-45026585	262	2.0	1.4	2.5	↓	-1.1	6.1E-04	8.8E-02
chr8:45891873-45892252	380	2.4	2.9	1.8	↑	1.1	3.5E-05	1.2E-02
chr8:45983728-45984236	509	3.1	2.5	3.5	↓	-0.9	2.7E-05	9.7E-03
chr8:4628336-4628667	332	1.9	2.3	1.2	↑	1.2	4.0E-04	6.7E-02
chr8:46484388-46485340	953	3.8	4.0	3.4	↑	0.6	6.1E-04	8.8E-02
chr8:46868454-46868876	423	2.1	1.3	2.7	↓	-1.4	6.4E-06	3.4E-03
chr8:46892466-46893014	549	3.3	2.8	3.6	↓	-0.8	1.0E-04	2.6E-02
chr8:46905460-46905909	450	3.5	2.8	3.9	↓	-1.1	3.2E-08	3.8E-05
chr8:47029558-47030129	572	3.7	3.0	4.2	↓	-1.2	4.5E-10	7.9E-07
chr8:47042660-47043233	574	3.2	2.7	3.6	↓	-0.9	3.4E-05	1.1E-02
chr8:47101262-47101647	386	3.3	2.7	3.8	↓	-1.0	2.6E-05	9.3E-03
chr8:47150063-47150331	269	1.8	1.0	2.3	↓	-1.2	4.2E-04	6.9E-02
chr8:47757855-47758562	708	4.2	3.9	4.5	↓	-0.6	5.1E-05	1.6E-02
chr8:47764464-47765437	974	4.2	3.9	4.5	↓	-0.6	2.4E-04	4.8E-02
chr8:49338684-49338946	263	1.8	2.3	1.1	↑	1.2	5.2E-04	7.8E-02
chr8:49983100-49983451	352	3.3	3.6	2.9	↑	0.7	7.2E-04	9.6E-02
chr8:50105873-50106318	446	3.8	3.3	4.2	↓	-0.9	1.9E-05	7.5E-03
chr8:51108776-51109301	526	2.3	2.7	1.8	↑	0.9	7.5E-04	9.9E-02
chr8:5190454-5191366	913	5.4	5.2	5.5	↓	-0.4	2.1E-04	4.5E-02
chr8:5227897-5228695	799	3.5	3.1	3.9	↓	-0.8	1.2E-04	3.1E-02
chr8:5731823-5733428	1606	5.5	5.2	5.7	↓	-0.5	3.0E-08	3.8E-05
chr8:5733577-5734073	497	4.8	4.4	5.0	↓	-0.6	1.5E-05	5.9E-03
chr8:57629573-57630056	484	2.7	2.1	3.2	↓	-1.0	5.0E-05	1.5E-02
chr8:58425419-58425875	457	3.0	2.3	3.4	↓	-1.1	4.4E-06	2.6E-03
chr8:58467652-58468082	431	4.3	4.0	4.5	↓	-0.6	2.6E-04	5.0E-02
chr8:589346-591516	2171	7.0	6.8	7.1	↓	-0.3	8.2E-08	9.0E-05
chr8:6064158-6069229	5072	7.4	7.3	7.5	↓	-0.2	3.6E-05	1.2E-02
chr8:61269470-61269890	421	2.6	3.0	2.1	↑	1.0	4.0E-04	6.7E-02
chr8:6243925-6244520	596	3.3	2.9	3.7	↓	-0.8	2.4E-04	4.9E-02
chr8:6273966-6274350	385	2.9	2.4	3.2	↓	-0.8	7.6E-04	9.9E-02
chr8:63947843-63948434	592	4.0	3.5	4.3	↓	-0.8	1.4E-05	5.9E-03
chr8:71029609-71030067	459	3.0	2.5	3.4	↓	-0.8	2.3E-04	4.7E-02
chr8:71440855-71441232	378	2.2	1.6	2.7	↓	-1.1	2.4E-04	4.9E-02
chr8:72672745-72673132	388	2.8	2.3	3.2	↓	-0.8	4.2E-04	6.9E-02
chr8:74074955-74077166	2212	5.2	5.0	5.4	↓	-0.4	2.6E-04	5.0E-02
chr8:74185051-74187103	2053	4.9	4.3	5.3	↓	-1.0	8.4E-09	1.2E-05
chr8:74187894-74188430	537	2.7	2.2	3.2	↓	-1.0	2.8E-04	5.4E-02

chr8:74763092-74764007	916	4.6	4.3	4.9	↓	-0.6	4.8E-06	2.7E-03
chr8:74845483-74846302	820	5.2	5.0	5.3	↓	-0.3	7.3E-04	9.7E-02
chr8:76067420-76067786	367	2.2	2.6	1.5	↑	1.1	4.5E-04	7.1E-02
chr8:76203474-76203971	498	5.2	5.0	5.5	↓	-0.5	1.4E-05	5.9E-03
chr8:76429240-76430144	905	4.8	4.4	5.1	↓	-0.7	4.4E-06	2.6E-03
chr8:76476405-76477406	1002	4.3	4.0	4.6	↓	-0.6	6.6E-04	9.0E-02
chr8:76749135-76749509	375	3.2	2.7	3.5	↓	-0.8	3.9E-04	6.6E-02
chr8:77989215-77989825	611	2.5	2.9	1.9	↑	1.0	2.5E-04	4.9E-02
chr8:79148367-79150010	1644	6.2	6.0	6.4	↓	-0.4	3.7E-04	6.4E-02
chr8:80471297-80471974	678	3.5	3.8	3.1	↑	0.8	6.5E-04	9.0E-02
chr8:80674992-80676280	1289	5.9	6.1	5.6	↑	0.6	1.5E-09	2.4E-06
chr8:80715855-80716413	559	2.9	2.2	3.3	↓	-1.1	1.4E-06	9.4E-04
chr8:80953615-80954052	438	2.6	3.1	2.0	↑	1.0	1.0E-04	2.6E-02
chr8:81250151-81251101	951	5.6	5.3	5.8	↓	-0.5	1.3E-04	3.2E-02
chr8:81512509-81512925	417	2.6	2.0	3.0	↓	-0.9	3.0E-04	5.5E-02
chr8:81575613-81576076	464	3.1	2.6	3.5	↓	-0.9	9.4E-05	2.5E-02
chr8:82713459-82714640	1182	5.0	5.2	4.8	↑	0.4	2.3E-04	4.7E-02
chr8:83721003-83721707	705	3.6	3.2	3.9	↓	-0.7	2.1E-05	7.9E-03
chr8:8386735-8387086	352	2.3	1.5	2.8	↓	-1.2	2.3E-05	8.5E-03
chr8:839342-839862	521	3.7	3.3	4.0	↓	-0.7	9.6E-05	2.5E-02
chr8:84166933-84167283	351	1.9	1.2	2.3	↓	-1.2	4.9E-04	7.6E-02
chr8:84610007-84610379	373	2.1	1.4	2.5	↓	-1.1	3.0E-04	5.6E-02
chr8:84614775-84615638	864	4.7	4.2	5.0	↓	-0.8	8.7E-07	6.2E-04
chr8:85977966-85978783	818	4.2	4.5	3.8	↑	0.7	3.0E-04	5.5E-02
chr8:88046611-88046864	254	2.3	2.7	1.6	↑	1.1	7.7E-04	1.0E-01
chr8:89042585-89043457	873	3.7	4.0	3.3	↑	0.7	2.0E-04	4.4E-02
chr8:89045592-89046322	731	3.5	4.0	2.7	↑	1.3	6.9E-10	1.1E-06
chr8:89051137-89051560	424	2.2	2.7	1.2	↑	1.5	8.3E-07	6.0E-04
chr8:89250884-89251338	455	3.7	3.4	4.0	↓	-0.6	7.6E-04	9.9E-02
chr8:90727014-90727612	599	3.1	2.6	3.5	↓	-0.9	5.2E-05	1.6E-02
chr8:90731548-90732885	1338	4.8	4.5	5.1	↓	-0.7	4.6E-07	3.8E-04
chr8:90734620-90734954	335	1.7	0.7	2.3	↓	-1.6	3.2E-05	1.1E-02
chr8:9123971-9124212	242	1.7	1.0	2.2	↓	-1.3	3.1E-04	5.7E-02
chr8:91561882-91562169	288	2.0	1.3	2.4	↓	-1.1	4.3E-04	6.9E-02
chr8:91568270-91569152	883	4.7	4.5	4.9	↓	-0.4	6.3E-04	8.9E-02
chr8:92150437-92150826	390	3.0	2.6	3.4	↓	-0.7	6.6E-04	9.0E-02
chr8:94480021-94480434	414	2.5	1.9	2.8	↓	-0.9	4.6E-04	7.3E-02
chr8:96672474-96673171	698	3.7	3.2	4.0	↓	-0.8	4.7E-06	2.7E-03
chr8:99687695-99687992	298	2.4	1.9	2.8	↓	-1.0	3.6E-04	6.2E-02
chr9:100486949-100487741	793	4.6	5.0	4.2	↑	0.8	1.6E-04	3.9E-02
chr9:10056530-10057826	1297	5.5	5.7	5.3	↑	0.4	2.1E-05	9.1E-03
chr9:100856901-100857701	801	3.7	3.3	4.1	↓	-0.8	2.3E-06	1.6E-03
chr9:101097706-101097917	212	1.6	0.8	2.1	↓	-1.2	5.0E-04	8.3E-02
chr9:103307732-103308342	611	3.2	3.7	2.5	↑	1.2	1.3E-06	1.0E-03
chr9:103309919-103310213	295	2.4	3.1	0.9	↑	2.2	4.9E-12	2.1E-08
chr9:105205792-105206022	231	1.9	1.1	2.4	↓	-1.2	2.7E-04	5.6E-02
chr9:10812000-10812720	721	4.6	4.2	4.8	↓	-0.6	9.3E-06	5.1E-03

chr9:10875864-10879129	3266	7.4	7.3	7.5	↓	-0.2	6.4E-04	9.6E-02
chr9:109110642-109110969	328	1.6	0.5	2.2	↓	-1.7	7.8E-06	4.5E-03
chr9:109111466-109111864	399	2.4	1.8	2.9	↓	-1.1	1.6E-04	3.9E-02
chr9:11034514-11034865	352	3.0	2.5	3.4	↓	-0.9	3.9E-05	1.4E-02
chr9:11174675-11175141	467	3.2	2.5	3.6	↓	-1.2	4.6E-08	6.7E-05
chr9:112491586-112491888	303	2.5	2.9	1.9	↑	1.0	3.3E-04	6.3E-02
chr9:113109596-113110370	775	5.3	5.1	5.6	↓	-0.5	9.5E-07	7.7E-04
chr9:11361059-11361548	490	3.8	4.1	3.5	↑	0.6	2.7E-04	5.5E-02
chr9:114112258-114112785	528	4.0	4.2	3.7	↑	0.6	2.3E-04	4.9E-02
chr9:114595528-114596235	708	5.0	4.6	5.3	↓	-0.7	1.4E-06	1.0E-03
chr9:114599053-114599447	395	3.2	2.8	3.5	↓	-0.7	6.7E-04	9.8E-02
chr9:11461154-11461621	468	3.0	3.3	2.4	↑	0.9	8.8E-05	2.5E-02
chr9:11473495-11474148	654	3.4	2.9	3.8	↓	-0.9	2.3E-05	9.8E-03
chr9:116320889-116321286	398	4.5	4.8	4.2	↑	0.5	3.9E-05	1.4E-02
chr9:116807573-116807898	326	3.1	2.7	3.5	↓	-0.8	4.5E-04	7.8E-02
chr9:11841433-11842899	1467	5.3	5.5	5.0	↑	0.5	8.9E-05	2.5E-02
chr9:118449048-118449557	510	4.1	3.8	4.4	↓	-0.6	6.7E-04	9.8E-02
chr9:118963238-118963677	440	4.2	3.8	4.5	↓	-0.7	1.2E-05	5.9E-03
chr9:120343085-120343899	815	3.7	3.3	4.1	↓	-0.8	1.2E-05	5.9E-03
chr9:120903364-120903847	484	4.1	3.8	4.4	↓	-0.6	1.6E-04	3.9E-02
chr9:12355027-12355621	595	2.6	3.0	2.0	↑	1.0	2.6E-04	5.5E-02
chr9:124209-126598	2390	7.3	7.4	7.2	↑	0.2	2.3E-04	5.0E-02
chr9:125211980-125212281	302	3.0	2.4	3.4	↓	-1.0	4.0E-05	1.5E-02
chr9:125600715-125600956	242	1.8	2.3	1.1	↑	1.2	5.7E-04	9.0E-02
chr9:125775864-125776139	276	2.9	2.3	3.3	↓	-1.0	2.1E-04	4.8E-02
chr9:126103102-126103429	328	2.8	2.0	3.3	↓	-1.3	2.5E-07	2.5E-04
chr9:126105203-126105786	584	2.9	2.0	3.4	↓	-1.4	7.6E-09	1.4E-05
chr9:126313750-126314171	422	2.9	2.2	3.4	↓	-1.2	9.7E-07	7.8E-04
chr9:12759646-12760025	380	2.6	3.0	1.8	↑	1.2	9.2E-06	5.1E-03
chr9:128047631-128047957	327	2.0	2.4	1.4	↑	1.1	6.5E-04	9.7E-02
chr9:129013888-129014350	463	3.1	3.5	2.6	↑	0.9	3.5E-05	1.3E-02
chr9:129107073-129107720	648	3.4	2.9	3.7	↓	-0.8	6.6E-05	2.1E-02
chr9:129256678-129257540	863	3.7	3.3	4.0	↓	-0.8	2.4E-04	5.0E-02
chr9:130036665-130036969	305	2.1	2.5	1.4	↑	1.1	5.7E-04	9.0E-02
chr9:130093430-130093983	554	2.9	2.1	3.4	↓	-1.4	1.3E-08	2.2E-05
chr9:130149712-130150543	832	4.0	4.3	3.7	↑	0.6	4.4E-04	7.6E-02
chr9:131503834-131504236	403	2.7	3.0	2.2	↑	0.9	5.2E-04	8.7E-02
chr9:131983308-131983782	475	2.6	3.0	1.9	↑	1.1	3.6E-05	1.4E-02
chr9:132534810-132535437	628	3.4	2.7	3.9	↓	-1.1	4.7E-06	2.9E-03
chr9:132688604-132688913	310	2.6	2.0	3.1	↓	-1.0	2.2E-04	4.8E-02
chr9:132692603-132693956	1354	4.5	4.1	4.8	↓	-0.7	1.2E-05	6.1E-03
chr9:132735199-132735538	340	2.8	2.2	3.2	↓	-1.0	3.0E-05	1.2E-02
chr9:132851266-132851616	351	3.3	2.8	3.7	↓	-0.8	3.3E-04	6.4E-02
chr9:133594768-133595121	354	3.0	2.4	3.3	↓	-0.9	1.9E-04	4.5E-02
chr9:134014803-134016931	2129	6.0	6.2	5.8	↑	0.4	6.1E-04	9.3E-02
chr9:135106522-135107121	600	5.0	4.7	5.3	↓	-0.6	1.4E-07	1.4E-04
chr9:13545282-13545891	610	5.5	5.6	5.3	↑	0.3	2.1E-04	4.8E-02

chr9:135470364-135471380	1017	5.3	5.5	5.1	↑	0.4	4.3E-04	7.5E-02
chr9:135707852-135708368	517	3.0	2.5	3.4	↓	-0.8	4.1E-04	7.3E-02
chr9:135900144-135900816	673	3.0	3.4	2.4	↑	1.0	4.4E-05	1.5E-02
chr9:136565395-136566500	1106	4.8	4.6	5.1	↓	-0.5	6.5E-06	3.9E-03
chr9:137339333-137340055	723	3.1	2.5	3.6	↓	-1.0	3.1E-05	1.2E-02
chr9:137478568-137478917	350	2.8	2.1	3.2	↓	-1.1	1.1E-05	5.7E-03
chr9:137767130-137767661	532	3.6	3.3	3.9	↓	-0.7	3.4E-04	6.5E-02
chr9:139011948-139012215	268	1.7	2.2	0.9	↑	1.3	2.2E-04	4.8E-02
chr9:140419845-140420164	320	1.8	0.9	2.4	↓	-1.5	2.0E-05	9.0E-03
chr9:141992617-141993116	500	2.6	2.0	3.0	↓	-0.9	2.8E-04	5.7E-02
chr9:142133565-142134617	1053	5.2	5.4	4.9	↑	0.4	9.6E-05	2.6E-02
chr9:142134644-142135375	732	3.6	3.9	3.3	↑	0.6	2.2E-04	4.8E-02
chr9:142174373-142175405	1033	4.8	5.0	4.5	↑	0.4	3.7E-04	6.9E-02
chr9:142192634-142193911	1278	6.3	6.1	6.4	↓	-0.3	1.9E-04	4.5E-02
chr9:142684952-142685346	395	3.7	4.1	3.2	↑	0.9	1.1E-06	8.3E-04
chr9:142744007-142745781	1775	5.5	5.3	5.6	↓	-0.3	4.1E-04	7.4E-02
chr9:143436746-143437353	608	3.5	2.8	3.9	↓	-1.1	2.6E-08	4.0E-05
chr9:143443056-143444069	1014	4.7	4.4	4.9	↓	-0.5	3.2E-04	6.2E-02
chr9:144309460-144309971	512	3.2	3.5	2.8	↑	0.7	3.1E-04	6.0E-02
chr9:14532642-14534357	1716	5.7	5.5	5.9	↓	-0.4	5.6E-05	1.8E-02
chr9:145479464-145482140	2677	5.9	5.7	6.1	↓	-0.3	1.2E-04	3.1E-02
chr9:145631431-145631888	458	2.6	3.0	2.1	↑	0.9	6.7E-04	9.8E-02
chr9:145727669-145731599	3931	7.3	7.4	7.2	↑	0.2	6.9E-05	2.1E-02
chr9:145731732-145732262	531	3.8	3.5	4.1	↓	-0.7	2.1E-04	4.7E-02
chr9:146403053-146403663	611	4.2	3.9	4.5	↓	-0.6	2.5E-05	1.0E-02
chr9:146458753-146460090	1338	5.7	5.5	5.9	↓	-0.4	1.0E-04	2.7E-02
chr9:146516962-146517914	953	4.3	4.0	4.6	↓	-0.6	9.6E-06	5.1E-03
chr9:146530722-146531949	1228	6.0	5.8	6.2	↓	-0.4	8.6E-07	7.4E-04
chr9:146917268-146918204	937	4.4	3.9	4.7	↓	-0.9	7.9E-07	7.0E-04
chr9:146998817-146999425	609	5.3	4.8	5.6	↓	-0.8	9.2E-11	2.7E-07
chr9:147043471-147044129	659	4.7	4.3	5.0	↓	-0.6	1.2E-07	1.3E-04
chr9:147583089-147583922	834	3.4	3.1	3.7	↓	-0.7	4.7E-04	8.0E-02
chr9:147660997-147661794	798	3.8	3.2	4.3	↓	-1.1	2.1E-11	7.1E-08
chr9:147754715-147756421	1707	6.2	6.0	6.4	↓	-0.4	6.8E-10	1.8E-06
chr9:148348505-148352900	4396	7.6	7.7	7.5	↑	0.2	7.1E-04	1.0E-01
chr9:148356070-148356550	481	2.9	2.3	3.3	↓	-1.0	8.7E-05	2.5E-02
chr9:148524629-148525679	1051	4.6	4.2	4.8	↓	-0.6	5.3E-04	8.7E-02
chr9:14861852-14862547	696	3.8	3.4	4.1	↓	-0.8	1.6E-04	3.9E-02
chr9:148813315-148814930	1616	7.1	7.0	7.2	↓	-0.2	2.4E-04	5.0E-02
chr9:148833903-148834497	595	4.8	4.3	5.2	↓	-0.9	2.2E-07	2.2E-04
chr9:148895413-148896162	750	3.8	3.4	4.1	↓	-0.7	3.0E-04	5.9E-02
chr9:149047272-149048639	1368	5.5	5.3	5.7	↓	-0.4	4.2E-06	2.6E-03
chr9:14994679-14995172	494	3.0	2.5	3.4	↓	-0.9	4.5E-05	1.5E-02
chr9:15060453-15061080	628	4.0	3.6	4.3	↓	-0.7	1.4E-05	6.6E-03
chr9:150745707-150747361	1655	6.7	6.6	6.8	↓	-0.3	2.5E-05	1.0E-02
chr9:151691278-151692278	1001	4.8	4.5	5.0	↓	-0.5	3.7E-05	1.4E-02
chr9:151739667-151740572	906	5.1	4.9	5.3	↓	-0.4	3.7E-04	6.8E-02

chr9:152225567-152226295	729	4.0	3.2	4.4	↓	-1.2	3.2E-11	1.0E-07
chr9:152249044-152249460	417	3.0	2.4	3.4	↓	-1.0	1.9E-05	8.5E-03
chr9:152434878-152435527	650	3.8	3.4	4.1	↓	-0.8	2.1E-05	9.1E-03
chr9:152537962-152538252	291	3.3	2.9	3.7	↓	-0.8	5.4E-04	8.8E-02
chr9:1526088-1527353	1266	6.2	6.0	6.3	↓	-0.2	4.9E-04	8.3E-02
chr9:152789539-152790470	932	4.4	4.0	4.7	↓	-0.8	4.4E-05	1.5E-02
chr9:152806671-152808118	1448	5.4	5.1	5.6	↓	-0.4	1.0E-04	2.8E-02
chr9:152893408-152894089	682	4.4	4.1	4.7	↓	-0.6	1.3E-04	3.3E-02
chr9:153163153-153164107	955	4.9	4.6	5.1	↓	-0.4	3.9E-04	7.1E-02
chr9:153374678-153376217	1540	5.2	5.4	5.0	↑	0.4	2.7E-04	5.5E-02
chr9:153607483-153607811	329	2.4	1.3	3.0	↓	-1.6	5.7E-08	7.8E-05
chr9:153611282-153611815	534	2.8	3.2	2.2	↑	1.0	1.6E-05	7.6E-03
chr9:18030571-18030941	371	2.2	1.6	2.6	↓	-1.0	4.6E-04	7.9E-02
chr9:19496382-19497331	950	5.3	4.9	5.5	↓	-0.6	6.7E-05	2.1E-02
chr9:22527755-22528222	468	3.8	3.4	4.1	↓	-0.7	2.3E-04	5.0E-02
chr9:22652955-22653362	408	4.0	3.4	4.3	↓	-0.9	7.8E-05	2.3E-02
chr9:22904399-22905165	767	3.5	3.1	3.9	↓	-0.7	6.2E-05	2.0E-02
chr9:22992892-22994108	1217	6.6	6.2	6.8	↓	-0.6	2.2E-08	3.5E-05
chr9:24333949-24334495	547	3.7	3.3	4.1	↓	-0.8	9.1E-05	2.5E-02
chr9:25400379-25401788	1410	4.8	4.3	5.2	↓	-0.9	6.8E-08	8.8E-05
chr9:25463835-25464782	948	5.2	5.0	5.3	↓	-0.4	2.1E-04	4.8E-02
chr9:25766042-25766326	285	2.3	1.4	2.8	↓	-1.4	8.7E-07	7.4E-04
chr9:26014084-26014319	236	1.8	2.3	1.0	↑	1.3	2.4E-04	5.1E-02
chr9:2622084-2622916	833	4.3	3.8	4.7	↓	-0.9	7.9E-08	9.7E-05
chr9:27425943-27426382	440	3.2	2.8	3.5	↓	-0.7	4.8E-04	8.2E-02
chr9:27534024-27534364	341	1.9	2.4	1.1	↑	1.4	7.2E-05	2.2E-02
chr9:2770382-2771105	724	3.9	3.6	4.2	↓	-0.6	3.2E-04	6.2E-02
chr9:28636255-28637648	1394	5.5	5.7	5.3	↑	0.3	5.5E-04	8.8E-02
chr9:2887846-2888363	518	4.2	3.7	4.5	↓	-0.8	7.6E-08	9.6E-05
chr9:29513207-29513785	579	4.6	4.3	4.8	↓	-0.5	1.3E-04	3.3E-02
chr9:29708233-29710351	2119	5.7	5.5	5.8	↓	-0.3	6.7E-04	9.8E-02
chr9:2978680-2979657	978	4.5	4.0	4.9	↓	-0.9	1.5E-09	3.5E-06
chr9:30333654-30334926	1273	5.1	5.3	4.9	↑	0.4	3.2E-05	1.2E-02
chr9:30574794-30575019	226	2.0	1.3	2.4	↓	-1.1	7.0E-04	9.9E-02
chr9:31030878-31031262	385	2.7	2.0	3.2	↓	-1.2	3.2E-06	2.1E-03
chr9:31387104-31387885	782	5.1	5.0	5.3	↓	-0.4	5.5E-04	8.8E-02
chr9:31711550-31711928	379	2.3	2.7	1.7	↑	1.0	3.8E-04	6.9E-02
chr9:32686510-32686895	386	3.7	2.8	4.2	↓	-1.5	2.1E-12	1.2E-08
chr9:35688465-35689946	1482	4.9	4.6	5.2	↓	-0.6	4.5E-06	2.8E-03
chr9:36304904-36305530	627	3.5	3.1	3.8	↓	-0.7	8.6E-05	2.5E-02
chr9:36361386-36361658	273	2.4	1.6	2.9	↓	-1.3	4.3E-05	1.5E-02
chr9:36574603-36575011	409	2.3	1.6	2.8	↓	-1.2	7.0E-05	2.1E-02
chr9:36577840-36578438	599	3.3	2.5	3.8	↓	-1.3	4.4E-09	8.5E-06
chr9:36579162-36579667	506	3.9	3.4	4.3	↓	-0.9	3.4E-07	3.3E-04
chr9:36703516-36703904	389	3.9	3.4	4.2	↓	-0.8	9.5E-06	5.1E-03
chr9:37236185-37237227	1043	4.6	4.3	4.9	↓	-0.6	4.1E-05	1.5E-02
chr9:37240739-37241615	877	4.5	4.2	4.7	↓	-0.5	6.0E-04	9.3E-02

chr9:37482204-37483480	1277	3.7	3.3	4.1	↓	-0.9	3.7E-04	6.9E-02
chr9:386209-390281	4073	8.5	8.4	8.6	↓	-0.2	8.7E-06	4.9E-03
chr9:38691625-38692018	394	3.3	2.5	3.8	↓	-1.3	3.8E-09	8.1E-06
chr9:38836368-38837203	836	4.2	3.6	4.6	↓	-1.0	8.8E-08	1.0E-04
chr9:41134619-41135120	502	3.2	2.7	3.5	↓	-0.8	7.0E-04	9.9E-02
chr9:42361564-42361938	375	2.3	1.7	2.7	↓	-1.0	4.7E-04	8.0E-02
chr9:44484700-44485147	448	4.7	4.5	4.9	↓	-0.4	6.8E-04	9.8E-02
chr9:45429924-45430251	328	2.2	2.6	1.6	↑	1.0	6.3E-04	9.6E-02
chr9:45741323-45741841	519	3.3	2.8	3.6	↓	-0.8	6.7E-05	2.1E-02
chr9:45814830-45815651	822	5.2	5.0	5.4	↓	-0.4	1.3E-04	3.3E-02
chr9:45835189-45835663	475	3.8	3.4	4.1	↓	-0.7	3.5E-04	6.5E-02
chr9:45886501-45887944	1444	5.7	5.5	5.9	↓	-0.4	1.1E-04	2.8E-02
chr9:46155237-46155947	711	4.5	4.2	4.7	↓	-0.6	3.0E-04	5.9E-02
chr9:46156490-46156863	374	2.7	3.1	2.0	↑	1.1	2.4E-05	1.0E-02
chr9:46518750-46521075	2326	6.2	6.0	6.4	↓	-0.4	5.1E-05	1.7E-02
chr9:46999865-47001457	1593	6.2	5.9	6.4	↓	-0.5	7.7E-06	4.5E-03
chr9:47148867-47149159	293	2.4	1.8	2.8	↓	-0.9	6.6E-04	9.8E-02
chr9:47270473-47271089	617	3.0	2.5	3.3	↓	-0.8	5.3E-04	8.7E-02
chr9:47482264-47483330	1067	5.6	5.4	5.8	↓	-0.4	2.3E-05	9.9E-03
chr9:47711957-47712836	880	4.6	4.1	4.9	↓	-0.8	6.0E-07	5.5E-04
chr9:47722457-47722754	298	1.8	2.3	1.1	↑	1.3	1.2E-04	3.1E-02
chr9:47901656-47902069	414	3.5	3.0	3.8	↓	-0.9	5.4E-05	1.8E-02
chr9:48077042-48078127	1086	5.7	5.6	5.9	↓	-0.3	2.6E-04	5.5E-02
chr9:48144956-48145628	673	3.4	3.0	3.7	↓	-0.8	2.0E-04	4.6E-02
chr9:48196311-48197438	1128	5.6	5.4	5.8	↓	-0.4	1.0E-04	2.8E-02
chr9:48210865-48212476	1612	6.0	5.8	6.2	↓	-0.3	2.0E-04	4.7E-02
chr9:48780853-48781550	698	3.4	3.8	3.0	↑	0.8	5.6E-05	1.8E-02
chr9:48875975-48876367	393	2.1	2.6	1.5	↑	1.1	4.3E-04	7.6E-02
chr9:50136401-50136968	568	3.8	3.4	4.1	↓	-0.7	2.9E-05	1.2E-02
chr9:50221483-50222060	578	2.8	3.2	2.4	↑	0.9	2.9E-04	5.8E-02
chr9:50280760-50281965	1206	4.9	4.7	5.1	↓	-0.4	3.0E-04	5.9E-02
chr9:50306131-50307126	996	4.7	4.4	4.9	↓	-0.5	9.5E-06	5.1E-03
chr9:50307350-50308224	875	4.1	3.3	4.6	↓	-1.3	4.9E-13	3.1E-09
chr9:50309902-50312085	2184	5.7	4.8	6.2	↓	-1.4	6.6E-56	2.9E-51
chr9:50312125-50312636	512	2.9	2.3	3.3	↓	-1.0	4.8E-05	1.6E-02
chr9:50312892-50314245	1354	5.3	4.8	5.7	↓	-0.9	2.9E-15	4.2E-11
chr9:50315701-50316533	833	3.6	3.2	4.0	↓	-0.8	2.5E-05	1.0E-02
chr9:50376048-50376991	944	4.4	4.1	4.7	↓	-0.6	8.4E-05	2.4E-02
chr9:50494544-50495526	983	4.4	4.2	4.6	↓	-0.4	5.8E-04	9.0E-02
chr9:51153572-51154125	554	3.1	2.7	3.5	↓	-0.8	1.6E-04	3.9E-02
chr9:5148875-5149558	684	3.8	3.5	4.1	↓	-0.6	1.5E-04	3.7E-02
chr9:51853171-51854527	1357	5.3	5.0	5.6	↓	-0.6	5.3E-08	7.6E-05
chr9:52231045-52232101	1057	4.6	4.2	5.0	↓	-0.8	7.5E-10	1.8E-06
chr9:52392323-52393894	1572	5.9	6.0	5.7	↑	0.4	2.2E-04	4.8E-02
chr9:52431338-52431851	514	3.6	3.9	3.3	↑	0.7	1.3E-04	3.3E-02
chr9:52473456-52473721	266	2.5	1.9	2.9	↓	-0.9	5.7E-04	9.0E-02
chr9:52473867-52475152	1286	4.8	5.0	4.6	↑	0.5	5.9E-05	1.9E-02

chr9:52481277-52481613	337	2.5	2.9	1.8	↑	1.1	2.7E-04	5.6E-02
chr9:52589444-52589961	518	2.5	2.9	1.9	↑	1.0	6.1E-04	9.3E-02
chr9:52744473-52745542	1070	3.9	4.2	3.5	↑	0.6	5.4E-04	8.8E-02
chr9:52748722-52750096	1375	4.8	5.0	4.6	↑	0.4	2.7E-04	5.6E-02
chr9:52812615-52813316	702	4.0	4.4	3.6	↑	0.8	3.0E-06	2.0E-03
chr9:52863584-52864034	451	3.8	3.4	4.1	↓	-0.7	1.5E-04	3.7E-02
chr9:52971901-52972414	514	2.5	2.9	2.0	↑	0.9	4.4E-04	7.6E-02
chr9:53002947-53003410	464	2.9	3.4	2.2	↑	1.2	1.8E-05	8.5E-03
chr9:53102997-53103622	626	3.4	3.7	3.0	↑	0.7	6.2E-04	9.5E-02
chr9:53113020-53115165	2146	5.6	5.8	5.4	↑	0.4	5.4E-04	8.8E-02
chr9:53236923-53237501	579	3.1	3.4	2.6	↑	0.8	5.5E-04	8.8E-02
chr9:53241088-53241756	669	3.5	3.8	3.1	↑	0.7	6.9E-04	9.9E-02
chr9:53416820-53417196	377	2.9	3.2	2.4	↑	0.9	4.5E-04	7.8E-02
chr9:5395921-5396301	381	3.6	3.9	3.2	↑	0.7	8.3E-05	2.4E-02
chr9:54263882-54264325	444	3.9	3.4	4.4	↓	-1.0	3.1E-10	8.6E-07
chr9:54332904-54333251	348	2.7	2.1	3.1	↓	-1.0	4.3E-04	7.5E-02
chr9:545839-546718	880	5.1	4.8	5.4	↓	-0.5	1.3E-05	6.5E-03
chr9:54793365-54793720	356	2.9	2.2	3.5	↓	-1.3	1.3E-07	1.4E-04
chr9:54977607-54978115	509	3.8	4.1	3.4	↑	0.7	6.4E-04	9.7E-02
chr9:55701396-55701996	601	5.2	4.8	5.5	↓	-0.6	6.9E-07	6.2E-04
chr9:57872485-57873841	1357	3.8	2.6	4.5	↓	-1.9	8.8E-09	1.5E-05
chr9:57873846-57874504	659	3.0	1.8	3.6	↓	-1.8	4.2E-09	8.5E-06
chr9:57875725-57876367	643	3.3	2.7	3.7	↓	-1.0	2.7E-06	1.8E-03
chr9:57877140-57877434	295	2.3	1.7	2.7	↓	-1.1	2.2E-04	4.8E-02
chr9:57921526-57923193	1668	5.5	5.0	5.9	↓	-0.8	6.6E-12	2.6E-08
chr9:58067300-58067942	643	3.3	2.5	3.9	↓	-1.4	1.2E-06	9.0E-04
chr9:58070017-58071190	1174	3.7	3.1	4.1	↓	-0.9	1.6E-05	7.7E-03
chr9:58084077-58084699	623	3.9	3.6	4.2	↓	-0.5	4.7E-04	8.0E-02
chr9:58096005-58097048	1044	4.1	3.7	4.3	↓	-0.6	1.6E-04	3.9E-02
chr9:58114153-58115209	1057	4.1	3.7	4.4	↓	-0.7	4.4E-06	2.8E-03
chr9:58772493-58774036	1544	5.2	5.0	5.4	↓	-0.4	3.5E-04	6.5E-02
chr9:59039568-59039938	371	2.7	2.2	3.1	↓	-0.9	3.0E-04	5.9E-02
chr9:59112768-59113506	739	3.4	2.9	3.7	↓	-0.8	3.1E-04	6.0E-02
chr9:59385566-59385934	369	1.9	1.3	2.4	↓	-1.1	6.8E-04	9.8E-02
chr9:5968571-5968978	408	4.2	3.7	4.5	↓	-0.8	3.0E-07	2.9E-04
chr9:59912160-59912507	348	2.6	2.1	3.0	↓	-0.9	6.7E-04	9.8E-02
chr9:60264501-60265448	948	4.6	4.8	4.3	↑	0.5	4.9E-05	1.7E-02
chr9:60483698-60484794	1097	5.1	4.7	5.3	↓	-0.6	5.0E-05	1.7E-02
chr9:60920594-60921089	496	3.9	4.2	3.6	↑	0.6	3.0E-04	5.9E-02
chr9:61249095-61250184	1090	5.7	5.5	5.9	↓	-0.4	5.8E-04	9.0E-02
chr9:61389369-61389795	427	3.1	2.6	3.5	↓	-0.9	6.3E-05	2.0E-02
chr9:62597190-62597850	661	3.4	3.0	3.8	↓	-0.8	4.0E-04	7.3E-02
chr9:62726015-62726818	804	4.4	4.1	4.6	↓	-0.6	2.6E-05	1.0E-02
chr9:63399115-63399391	277	1.8	1.0	2.4	↓	-1.4	2.0E-05	9.0E-03
chr9:63478291-63479743	1453	4.8	4.2	5.2	↓	-1.0	1.6E-09	3.5E-06
chr9:63485393-63486114	722	3.2	2.7	3.7	↓	-1.0	8.9E-07	7.4E-04
chr9:63538896-63540654	1759	6.4	6.5	6.3	↑	0.3	2.0E-04	4.6E-02

chr9:63583715-63584835	1121	6.3	5.8	6.6	↓	-0.8	2.1E-14	1.9E-10
chr9:63586679-63587044	366	2.3	1.3	2.8	↓	-1.5	2.5E-06	1.7E-03
chr9:63587330-63588249	920	3.8	3.3	4.1	↓	-0.8	3.5E-05	1.3E-02
chr9:63687666-63688075	410	3.9	3.6	4.2	↓	-0.6	6.0E-04	9.3E-02
chr9:63800394-63800706	313	2.3	2.8	1.7	↑	1.0	3.7E-04	6.9E-02
chr9:64228067-64228801	735	5.0	4.8	5.2	↓	-0.5	2.1E-04	4.8E-02
chr9:64243213-64243743	531	4.3	3.9	4.6	↓	-0.6	2.0E-05	9.0E-03
chr9:65890946-65891442	497	2.5	1.9	3.0	↓	-1.1	4.1E-05	1.5E-02
chr9:66487488-66488073	586	2.9	3.4	2.4	↑	1.0	7.9E-05	2.3E-02
chr9:66733170-66733665	496	4.0	3.3	4.4	↓	-1.1	1.3E-07	1.4E-04
chr9:66921856-66922267	412	4.8	4.5	5.1	↓	-0.6	2.5E-06	1.7E-03
chr9:67120347-67121673	1327	4.5	4.7	4.3	↑	0.4	3.9E-04	7.1E-02
chr9:67746787-67747305	519	3.8	3.3	4.1	↓	-0.9	1.6E-05	7.6E-03
chr9:6825970-6826433	464	4.2	3.8	4.5	↓	-0.7	9.0E-06	5.0E-03
chr9:683875-684630	756	4.2	3.9	4.5	↓	-0.6	7.5E-05	2.2E-02
chr9:70541681-70542617	937	4.7	4.2	5.0	↓	-0.8	5.9E-08	7.9E-05
chr9:70959168-70959420	253	1.9	2.4	1.1	↑	1.3	1.1E-04	2.8E-02
chr9:70960607-70961019	413	3.1	3.5	2.7	↑	0.7	6.8E-04	9.8E-02
chr9:71373317-71373857	541	4.0	3.5	4.3	↓	-0.8	2.0E-06	1.4E-03
chr9:7142705-7143287	583	4.8	5.0	4.6	↑	0.4	6.1E-04	9.3E-02
chr9:71451275-71452527	1253	4.0	4.2	3.6	↑	0.6	3.4E-04	6.5E-02
chr9:71705540-71706227	688	5.0	4.7	5.2	↓	-0.5	6.9E-04	9.9E-02
chr9:71855374-71856684	1311	4.8	4.5	5.1	↓	-0.6	3.3E-06	2.1E-03
chr9:719069-719588	520	3.0	2.6	3.3	↓	-0.8	4.6E-04	8.0E-02
chr9:72768734-72769071	338	2.2	2.6	1.6	↑	1.0	4.9E-04	8.3E-02
chr9:73117751-73118004	254	2.6	3.0	2.0	↑	1.0	2.1E-04	4.7E-02
chr9:73333418-73333942	525	3.2	2.7	3.5	↓	-0.7	3.7E-04	6.9E-02
chr9:73594049-73594315	267	2.2	1.6	2.7	↓	-1.1	2.8E-04	5.7E-02
chr9:73932201-73933813	1613	5.7	5.4	6.0	↓	-0.5	1.3E-05	6.2E-03
chr9:75409035-75409303	269	1.9	1.2	2.3	↓	-1.2	3.8E-04	6.9E-02
chr9:75614210-75614759	550	5.6	5.4	5.8	↓	-0.4	7.1E-05	2.1E-02
chr9:77005133-77005666	534	2.7	2.0	3.2	↓	-1.2	9.6E-05	2.6E-02
chr9:77272064-77272583	520	3.7	3.3	4.0	↓	-0.7	5.0E-04	8.3E-02
chr9:7766265-7766744	480	3.9	3.3	4.3	↓	-1.0	2.4E-08	3.7E-05
chr9:78310859-78311151	293	3.1	2.7	3.5	↓	-0.8	1.2E-04	3.1E-02
chr9:78317782-78318644	863	3.4	3.7	3.0	↑	0.7	6.9E-04	9.9E-02
chr9:7997843-7998447	605	4.1	3.8	4.4	↓	-0.5	6.6E-04	9.8E-02
chr9:80651755-80652273	519	3.7	3.2	4.0	↓	-0.7	7.4E-05	2.2E-02
chr9:81481141-81481435	295	2.4	2.9	1.7	↑	1.1	2.4E-05	1.0E-02
chr9:82861789-82862613	825	3.7	3.2	4.0	↓	-0.7	1.8E-04	4.2E-02
chr9:8358485-8358849	365	3.9	4.2	3.4	↑	0.9	1.1E-07	1.2E-04
chr9:84775382-84775800	419	4.2	4.5	3.9	↑	0.6	6.7E-05	2.1E-02
chr9:84977364-84977878	515	3.0	2.3	3.5	↓	-1.2	1.6E-06	1.2E-03
chr9:85498509-85498757	249	1.8	2.2	1.0	↑	1.2	4.7E-04	8.0E-02
chr9:86172785-86173167	383	4.1	3.7	4.3	↓	-0.6	5.7E-04	9.0E-02
chr9:86203244-86203659	416	2.0	2.5	1.3	↑	1.2	2.4E-04	5.2E-02
chr9:86235703-86236191	489	2.9	3.3	2.5	↑	0.8	6.3E-04	9.6E-02

chr9:87544733-87545288	556	2.9	2.3	3.3	↓	-1.0	2.3E-05	1.0E-02
chr9:87546652-87547256	605	4.0	3.3	4.5	↓	-1.3	1.1E-14	1.2E-10
chr9:87547349-87547695	347	1.8	1.1	2.3	↓	-1.2	1.7E-04	4.1E-02
chr9:87883082-87883299	218	2.2	1.4	2.6	↓	-1.2	9.3E-05	2.6E-02
chr9:8872440-8873507	1068	4.9	4.6	5.1	↓	-0.4	2.0E-04	4.7E-02
chr9:89217643-89218141	499	3.1	2.4	3.6	↓	-1.2	2.6E-06	1.8E-03
chr9:89218323-89218631	309	2.5	1.1	3.3	↓	-2.2	3.0E-14	2.2E-10
chr9:93822353-93822829	477	2.9	2.5	3.2	↓	-0.8	6.7E-04	9.8E-02
chr9:94501923-94502247	325	2.4	1.7	2.9	↓	-1.2	2.4E-05	1.0E-02
chr9:94663633-94664340	708	2.9	2.4	3.3	↓	-0.9	9.9E-05	2.7E-02
chr9:9502421-9502905	485	3.5	3.8	3.1	↑	0.7	2.7E-04	5.5E-02
chr9:95552669-95553008	340	1.7	2.1	0.9	↑	1.2	4.9E-04	8.3E-02
chr9:95602226-95602612	387	1.9	2.4	1.2	↑	1.1	5.4E-04	8.8E-02
chr9:95975382-95976022	641	3.2	3.6	2.8	↑	0.8	6.4E-04	9.6E-02
chr9:97319815-97320688	874	3.6	4.1	2.9	↑	1.2	1.9E-11	7.1E-08
chr9:97322598-97323407	810	4.2	4.7	3.5	↑	1.2	2.4E-12	1.2E-08
chr9:97324253-97324915	663	3.5	4.1	2.1	↑	2.0	2.7E-17	5.9E-13
chr9:97325838-97326198	361	2.5	2.9	1.9	↑	1.0	8.8E-05	2.5E-02
chr9:97330184-97331311	1128	4.7	5.1	4.2	↑	1.0	5.3E-07	4.9E-04
chr9:97548309-97548523	215	2.0	2.5	1.4	↑	1.1	5.5E-04	8.8E-02
chr9:99364605-99364911	307	1.8	1.0	2.3	↓	-1.3	1.9E-04	4.5E-02
chr9:99367479-99368185	707	3.2	2.7	3.6	↓	-0.9	7.8E-06	4.5E-03
chrX:124797716-124798226	511	3.4	2.4	3.9	↓	-1.460355	2.6E-06	1.9E-02
chrX:122007584-122008404	821	3.5	2.9	4.0	↓	-1.114052	1.9E-05	6.7E-02
chrX:142168950-142171433	2484	5.8	5.5	6.0	↓	-0.483541	4.2E-05	7.7E-02
