# Supplementary material for: Arterial endothelial methylome: differential DNA methylation in athero-susceptible disturbed flow regions in vivo
Source: BMC Genomics. 2015 Jul 7;16:506. doi: 10.1186/s12864-015-1656-4 (PMC4492093; doi:10.1186/s12864-015-1656-4)
Supplement: Additional file 3: Table S1b. — AA vs DT DMRs in the peri-promoter region (10 kb upstream and 1 kb downstream TSS). [file 12864_2015_1656_MOESM3_ESM.pdf]

Supplementary Table 1b
AA vs DT DMRs in the promoter region (10kb upstream and 1kb downstream TSS)

DMR	Ensembl Gene| Transcripts		Fold, log2(AA/DT)	p-value	FDR
chr1:10072648-10073607	ENSSSCG00000004055|RSPH3	↓	-0.66	1.15E-05	5.80E-03
chr1:101194381-101195145	ENSSSCG00000019589|U1	↓	-0.48	5.18E-04	9.01E-02
chr1:106455466-106455916	ENSSSCG00000004503|	↑	1.27	6.57E-06	3.89E-03
chr1:10897515-10897806	ENSSSCG00000012314|	↓	-1.47	2.21E-06	1.56E-03
chr1:117807968-117808808	ENSSSCG00000004545|ANKDD1A	↑	0.57	1.36E-05	6.48E-03
chr1:119725750-119726592	ENSSSCG00000029654|	↑	0.85	4.60E-06	2.88E-03
chr1:149725943-149727104	ENSSSCG00000004795|	↓	-1.01	1.60E-07	1.69E-04
chr1:149728920-149729779	ENSSSCG00000004795|	↓	-1.28	6.19E-09	1.09E-05
chr1:153861939-153862402	ENSSSCG00000004812|IGF1R	↓	-0.72	5.59E-04	9.42E-02
chr1:153861939-153862402	ENSSSCG00000025487|U6	↓	-0.72	5.59E-04	9.42E-02
chr1:154770011-154770694	ENSSSCG00000030487|LYSMD4	↓	-0.75	2.32E-05	9.40E-03
chr1:155739539-155740117	ENSSSCG00000018227|	↓	-0.78	1.65E-04	4.28E-02
chr1:157469564-157470108	ENSSSCG00000004830|ATP10A	↓	-1.14	4.74E-07	4.09E-04
chr1:164777826-164778963	ENSSSCG00000004862|	↓	-0.86	1.24E-11	5.40E-08
chr1:164779262-164781377	ENSSSCG00000004862|	↓	-1.34	4.15E-16	3.86E-12
chr1:164782977-164783908	ENSSSCG00000004862|	↓	-0.64	3.68E-05	1.34E-02
chr1:179672958-179674187	ENSSSCG00000004917|MALT1	↑	0.90	1.68E-07	1.72E-04
chr1:179675803-179676642	ENSSSCG00000004917|MALT1	↑	0.83	2.44E-04	5.59E-02
chr1:179680040-179680939	ENSSSCG00000004917|MALT1	↑	0.77	9.61E-06	5.09E-03
chr1:184688283-184688548	ENSSSCG00000019567|7SK	↑	1.11	5.87E-04	9.65E-02
chr1:209537063-209537850	ENSSSCG00000005078|DAAM1	↓	-0.71	1.48E-08	2.20E-05
chr1:210233494-210233818	ENSSSCG00000005082|PCNXL4	↓	-0.94	4.15E-04	7.72E-02
chr1:210736110-210737153	ENSSSCG00000005087|SIX1	↑	1.33	2.37E-07	2.38E-04
chr1:210737190-210738562	ENSSSCG00000005087|SIX1	↑	0.94	5.63E-11	2.29E-07
chr1:210739364-210741392	ENSSSCG00000005087|SIX1	↑	0.54	5.67E-14	3.08E-10
chr1:24857406-24857638	ENSSSCG00000004137|	↓	-1.53	3.34E-04	6.91E-02
chr1:256665909-256668072	ENSSSCG00000005279|FOXB2	↓	-0.70	3.55E-06	2.34E-03
chr1:266864477-266865119	ENSSSCG00000025337|	↓	-0.74	4.45E-04	8.17E-02
chr1:269046677-269047747	ENSSSCG00000005380|	↓	-0.41	2.91E-05	1.11E-02
chr1:279062208-279063677	ENSSSCG00000005437|KLF4	↓	-0.56	1.98E-06	1.43E-03
chr1:284870613-284871938	ENSSSCG00000005480|	↓	-0.56	3.36E-04	6.93E-02
chr1:286014672-286016544	ENSSSCG00000018765|ssc-mir-455	↑	0.40	5.93E-04	9.70E-02
chr1:302311986-302313808	ENSSSCG00000005623|ST6GALNAC6	↑	0.73	1.03E-04	3.13E-02
chr1:305465806-305466491	ENSSSCG00000005717|UCK1	↓	-0.57	1.14E-04	3.37E-02
chr1:307135779-307143457	ENSSSCG00000026442|	↓	-0.18	7.68E-06	4.28E-03
chr1:308660765-308670086	ENSSSCG00000005752|PAEP	↓	-0.15	2.00E-04	4.83E-02
chr1:308700700-308707497	ENSSSCG00000005756|	↓	-0.27	1.12E-07	1.28E-04
chr1:310103777-310104476	ENSSSCG00000005776|	↑	1.13	3.23E-04	6.76E-02
chr1:311922269-311922695	ENSSSCG00000005788|	↓	-0.63	2.48E-05	9.79E-03
chr1:76884335-76884889	ENSSSCG00000029152|	↑	1.18	1.33E-05	6.45E-03
chr1:87192325-87192965	ENSSSCG00000004421|FYN	↑	0.78	2.59E-04	5.86E-02
chr1:88080994-88081617	ENSSSCG00000004427|	↑	0.81	2.34E-04	5.43E-02

chr1:9469418-9469959	ENSSSCG00000029876|SOD2	↑	0.85	1.81E-04	4.48E-02
chr1:97176947-97178807	ENSSSCG00000004468|SH3BGRL2	↓	-1.59	2.76E-53	1.80E-48
chr1:97179907-97180964	ENSSSCG00000004468|SH3BGRL2	↓	-2.02	7.56E-20	1.23E-15
chr1:99461207-99461582	ENSSSCG00000004475|HTR1B	↓	-1.17	6.54E-05	2.16E-02
chr10:16084850-16085177	ENSSSCG00000028491|SRP9	↓	-0.92	1.74E-05	7.87E-03
chr10:28617022-28617595	ENSSSCG00000030640|IPO9	↑	0.50	4.06E-04	7.89E-02
chr10:28666165-28667254	ENSSSCG00000010922|ELF3	↓	-0.77	3.25E-08	4.05E-05
chr10:29510029-29510357	ENSSSCG00000010934|HABP4	↑	0.84	3.68E-04	7.44E-02
chr10:31305405-31305949	ENSSSCG00000020444|U6	↓	-0.76	2.19E-04	5.12E-02
chr10:31305405-31305949	ENSSSCG00000023464|	↓	-0.76	2.19E-04	5.12E-02
chr10:31307468-31310241	ENSSSCG00000020444|U6	↓	-0.22	4.07E-04	7.89E-02
chr10:31307468-31310241	ENSSSCG00000023464|	↓	-0.22	4.07E-04	7.89E-02
chr10:45387502-45387935	ENSSSCG00000020112|5S_rRNA	↓	-1.30	1.56E-06	1.13E-03
chr10:45390285-45390538	ENSSSCG00000020112|5S_rRNA	↓	-1.56	2.95E-09	4.66E-06
chr10:48730593-48730906	ENSSSCG00000011037|	↑	0.76	8.28E-05	2.79E-02
chr10:54071823-54072144	ENSSSCG00000011067|	↑	0.66	5.10E-04	8.76E-02
chr10:59603519-59603761	ENSSSCG00000011090|	↓	-0.99	8.07E-06	4.38E-03
chr11:15553130-15553772	ENSSSCG00000009370|FOXO1	↓	-0.60	3.46E-05	1.05E-02
chr11:22072253-22072705	ENSSSCG00000009417|SPERT	↓	-0.97	2.67E-04	4.78E-02
chr11:22110750-22111330	ENSSSCG00000030250|7SK	↑	0.87	1.96E-04	3.84E-02
chr11:2810808-2811920	ENSSSCG00000027577|	↓	-0.49	2.78E-06	1.64E-03
chr11:298627-299139	ENSSSCG00000009276|XPO4	↓	-1.02	1.64E-07	1.98E-04
chr11:32001130-32001709	ENSSSCG00000009446|PCDH17	↓	-1.02	5.84E-04	7.73E-02
chr11:55831796-55832335	ENSSSCG00000024442|U6	↓	-0.86	8.37E-05	2.03E-02
chr11:5661169-5661894	ENSSSCG00000019964|U1	↓	-0.76	9.26E-07	7.20E-04
chr11:72413155-72413469	ENSSSCG00000009502|	↓	-0.94	5.70E-04	7.68E-02
chr11:7391431-7391902	ENSSSCG00000009331|C13orf33	↓	-0.50	4.77E-07	4.50E-04
chr11:75985174-75985794	ENSSSCG00000009519|CLYBL	↑	0.64	4.17E-04	6.30E-02
chr11:76490273-76490615	ENSSSCG00000029926|U6	↓	-1.54	7.99E-06	3.52E-03
chr11:77021483-77022000	ENSSSCG00000023476|	↓	-0.31	3.73E-04	5.83E-02
chr11:84605177-84606203	ENSSSCG00000009545|COL4A2	↓	-0.62	6.67E-07	5.88E-04
chr11:84606347-84608141	ENSSSCG00000009545|COL4A2	↓	-0.27	7.95E-04	9.73E-02
chr11:85069823-85073335	ENSSSCG00000024196|	↓	-0.18	1.84E-05	6.75E-03
chr11:86432630-86434705	ENSSSCG00000009563|TMCO3	↓	-0.21	2.11E-05	7.43E-03
chr11:86541930-86545080	ENSSSCG00000009564|FAM70B	↑	0.20	2.25E-04	4.19E-02
chr11:86755504-86755889	ENSSSCG00000009566|	↑	1.09	2.86E-04	4.96E-02
chr12:12357901-12358502	ENSSSCG00000017261|ARSG	↓	-0.45	8.62E-06	2.17E-03
chr12:12670811-12670982	ENSSSCG00000017265|AXIN2	↑	1.05	7.14E-04	7.26E-02
chr12:14910436-14910546	ENSSSCG00000017279|ERN1	↑	1.33	1.59E-04	2.33E-02
chr12:15041820-15042028	ENSSSCG00000017283|CD79B	↑	1.03	4.33E-04	5.12E-02
chr12:15044518-15045400	ENSSSCG00000017283|CD79B	↑	0.67	1.97E-06	7.10E-04
chr12:1506850-1509065	ENSSSCG00000024675|ssc-mir-338	↓	-0.28	2.54E-06	8.68E-04
chr12:15979165-15979348	ENSSSCG00000017300|MRC2	↓	-0.86	3.27E-04	4.10E-02
chr12:1694803-1695147	ENSSSCG00000017139|RPTOR	↑	1.02	5.87E-06	1.65E-03
chr12:18691220-18691550	ENSSSCG00000017343|GFAP	↓	-0.68	7.72E-04	7.63E-02
chr12:18954521-18954906	ENSSSCG00000030592|	↓	-0.59	7.10E-04	7.23E-02
chr12:18954521-18954906	ENSSSCG00000017352|	↓	-0.59	7.10E-04	7.23E-02

chr12:1895950-1896966	ENSSSCG00000021150|	↓	-0.38	1.44E-04	2.14E-02
chr12:19244898-19245350	ENSSSCG00000017357|ITGA2B	↓	-0.57	7.52E-04	7.54E-02
chr12:20380255-20380724	ENSSSCG00000017392|CCR10	↓	-0.96	1.12E-07	6.96E-05
chr12:20380255-20380724	ENSSSCG00000017391|PLEKHH3	↓	-0.96	1.12E-07	6.96E-05
chr12:20380255-20380724	ENSSSCG00000017393|	↓	-0.96	1.12E-07	6.96E-05
chr12:21737041-21737788	ENSSSCG00000017450|KRTAP3-1	↑	0.42	7.00E-04	7.16E-02
chr12:22161953-22162449	ENSSSCG00000017468|SMARCE1	↓	-0.43	3.15E-04	4.03E-02
chr12:22307585-22307808	ENSSSCG00000017473|TOP2A	↑	1.02	1.11E-03	9.94E-02
chr12:24036603-24036816	ENSSSCG00000017524|CDK5RAP3	↑	1.02	7.70E-04	7.63E-02
chr12:24036603-24036816	ENSSSCG00000023963|	↑	1.02	7.70E-04	7.63E-02
chr12:24635588-24636164	ENSSSCG00000026307|	↓	-0.70	8.07E-08	5.29E-05
chr12:24637268-24637698	ENSSSCG00000026307|	↓	-1.49	4.72E-18	1.60E-14
chr12:24743463-24744094	ENSSSCG00000017532|HOXB1	↓	-0.93	1.55E-11	2.06E-08
chr12:24744283-24745147	ENSSSCG00000017532|HOXB1	↓	-0.99	8.03E-15	1.76E-11
chr12:24745462-24746030	ENSSSCG00000017532|HOXB1	↓	-0.80	1.79E-10	1.80E-07
chr12:24768346-24768832	ENSSSCG00000017534|HOXB3	↑	0.88	5.52E-07	2.67E-04
chr12:24791361-24791535	ENSSSCG00000017535|HOXB4	↑	1.05	6.53E-04	6.86E-02
chr12:24791361-24791535	ENSSSCG00000019270|ssc-mir-10a	↑	1.05	6.53E-04	6.86E-02
chr12:24827590-24827951	ENSSSCG00000019834|ssc-mir-196a-1	↓	-1.65	5.76E-15	1.34E-11
chr12:24831209-24831655	ENSSSCG00000019834|ssc-mir-196a-1	↓	-1.48	5.03E-11	5.86E-08
chr12:24834468-24834767	ENSSSCG00000017536|HOXB9	↓	-1.65	7.69E-11	8.69E-08
chr12:24834468-24834767	ENSSSCG00000019834|ssc-mir-196a-1	↓	-1.65	7.69E-11	8.69E-08
chr12:24835086-24835290	ENSSSCG00000017536|HOXB9	↓	-2.20	2.71E-12	4.05E-09
chr12:24835086-24835290	ENSSSCG00000019834|ssc-mir-196a-1	↓	-2.20	2.71E-12	4.05E-09
chr12:24835745-24836051	ENSSSCG00000017536|HOXB9	↓	-1.71	1.30E-10	1.35E-07
chr12:24835745-24836051	ENSSSCG00000019834|ssc-mir-196a-1	↓	-1.71	1.30E-10	1.35E-07
chr12:24838227-24838388	ENSSSCG00000017536|HOXB9	↓	-1.12	2.04E-04	2.86E-02
chr12:24838675-24838918	ENSSSCG00000017536|HOXB9	↓	-1.11	5.34E-06	1.56E-03
chr12:24840865-24841303	ENSSSCG00000017536|HOXB9	↓	-1.83	4.74E-17	1.26E-13
chr12:24841392-24842007	ENSSSCG00000017536|HOXB9	↓	-1.90	2.03E-29	1.51E-25
chr12:24842159-24842534	ENSSSCG00000017536|HOXB9	↓	-1.70	3.18E-11	3.95E-08
chr12:24846060-24846435	ENSSSCG00000017537|HOXB8	↓	-2.50	3.27E-34	4.07E-30
chr12:24847341-24848292	ENSSSCG00000017537|HOXB8	↓	-2.13	4.98E-33	4.64E-29
chr12:24848350-24848589	ENSSSCG00000017537|HOXB8	↓	-2.08	2.63E-14	5.45E-11
chr12:24848753-24849845	ENSSSCG00000017538|HOXB7	↓	-2.55	3.82E-49	1.43E-44
chr12:24848753-24849845	ENSSSCG00000017537|HOXB8	↓	-2.55	3.82E-49	1.43E-44
chr12:24850919-24852307	ENSSSCG00000017538|HOXB7	↓	-2.61	8.63E-45	1.61E-40
chr12:24850919-24852307	ENSSSCG00000017537|HOXB8	↓	-2.61	8.63E-45	1.61E-40
chr12:24853395-24853694	ENSSSCG00000017538|HOXB7	↓	-2.31	5.89E-21	2.20E-17
chr12:24853395-24853694	ENSSSCG00000017537|HOXB8	↓	-2.31	5.89E-21	2.20E-17
chr12:24853803-24854149	ENSSSCG00000017538|HOXB7	↓	-2.38	3.49E-28	2.17E-24
chr12:24853803-24854149	ENSSSCG00000017537|HOXB8	↓	-2.38	3.49E-28	2.17E-24
chr12:24861067-24861467	ENSSSCG00000017539|HOXB6	↓	-1.01	5.96E-07	2.81E-04
chr12:24872400-24872756	ENSSSCG00000017540|HOXB5	↓	-1.77	1.63E-13	3.04E-10
chr12:24997022-24997295	ENSSSCG00000017543|CALCOCO2	↓	-0.76	1.20E-04	1.87E-02
chr12:25353363-25353637	ENSSSCG00000030269|B4GALNT2	↓	-0.79	3.03E-05	6.42E-03
chr12:25363318-25363664	ENSSSCG00000027677|ABI3	↓	-0.71	6.44E-04	6.80E-02

chr12:25701151-25701522	ENSSSCG00000017548|NGFR	↓	-0.53	9.45E-05	1.57E-02
chr12:26418901-26419267	ENSSSCG00000027593|	↓	-0.78	1.97E-04	2.78E-02
chr12:26418901-26419267	ENSSSCG00000021015|	↓	-0.78	1.97E-04	2.78E-02
chr12:26503062-26503386	ENSSSCG00000017580|TMEM92	↑	0.61	4.75E-05	9.05E-03
chr12:26927204-26927371	ENSSSCG00000017563|MYCBPAP	↓	-0.95	1.20E-04	1.87E-02
chr12:27281446-27282299	ENSSSCG00000017557|WFIKKN2	↓	-0.63	3.46E-07	1.77E-04
chr12:32253320-32253472	ENSSSCG00000017604|HLF	↑	1.13	6.03E-04	6.48E-02
chr12:3347815-3348034	ENSSSCG00000017165|USP36	↑	1.13	1.82E-04	2.61E-02
chr12:41278388-41278593	ENSSSCG00000017707|	↑	1.20	1.61E-04	2.35E-02
chr12:41541773-41542639	ENSSSCG00000025796|NLE1	↓	-0.51	6.40E-07	2.95E-04
chr12:41541773-41542639	ENSSSCG00000017717|UNC45B	↓	-0.51	6.40E-07	2.95E-04
chr12:41774112-41774397	ENSSSCG00000021313|LIG3	↓	-0.90	6.52E-04	6.86E-02
chr12:41774112-41774397	ENSSSCG00000022196|	↓	-0.90	6.52E-04	6.86E-02
chr12:43187495-43187802	ENSSSCG00000018170|	↓	-0.51	2.07E-06	7.33E-04
chr12:43722167-43722740	ENSSSCG00000017726|SPACA3	↓	-0.36	3.23E-04	4.08E-02
chr12:45282356-45282695	ENSSSCG00000018272|5S_rRNA	↓	-0.67	9.02E-04	8.63E-02
chr12:4821092-4822134	ENSSSCG00000017181|CYGB	↑	0.28	3.86E-04	4.63E-02
chr12:4822291-4822796	ENSSSCG00000017181|CYGB	↓	-0.80	1.75E-04	2.53E-02
chr12:4994689-4995481	ENSSSCG00000029901|UBE2O	↓	-0.73	3.12E-10	2.99E-07
chr12:50021755-50024961	ENSSSCG00000019370|ssc-mir-132	↑	0.41	1.13E-04	1.80E-02
chr12:50021755-50024961	ENSSSCG00000019896|ssc-mir-212	↑	0.41	1.13E-04	1.80E-02
chr12:50021755-50024961	ENSSSCG00000025717|	↑	0.41	1.13E-04	1.80E-02
chr12:51610546-51610872	ENSSSCG00000017867|EMC6	↓	-0.74	4.15E-04	4.93E-02
chr12:52822102-52822540	ENSSSCG00000017892|	↓	-0.46	5.36E-05	9.94E-03
chr12:53994021-53994398	ENSSSCG00000017902|CAMTA2	↓	-0.87	2.56E-05	5.72E-03
chr12:53994021-53994398	ENSSSCG00000017901|INCA1	↓	-0.87	2.56E-05	5.72E-03
chr12:53994021-53994398	ENSSSCG00000017900|KIF1C	↓	-0.87	2.56E-05	5.72E-03
chr12:5455586-5455764	ENSSSCG00000020309|U6	↑	1.17	2.20E-04	3.03E-02
chr12:55457559-55457872	ENSSSCG00000017962|KDM6B	↓	-2.00	2.97E-15	7.38E-12
chr12:5575157-5575344	ENSSSCG00000017202|H3F3A	↑	0.87	1.09E-03	9.79E-02
chr12:5575157-5575344	ENSSSCG00000017201|UNK	↑	0.87	1.09E-03	9.79E-02
chr12:5575157-5575344	ENSSSCG00000024769|	↑	0.87	1.09E-03	9.79E-02
chr12:56724453-56724688	ENSSSCG00000017990|	↓	-1.16	8.98E-04	8.63E-02
chr12:57508949-57510093	ENSSSCG00000028770|RCVRN	↓	-0.32	5.10E-04	5.79E-02
chr12:57986797-57987011	ENSSSCG00000029441|	↓	-1.03	8.41E-04	8.19E-02
chr12:58101772-58102028	ENSSSCG00000018007|MYH3	↓	-0.77	2.45E-05	5.58E-03
chr12:60954263-60954441	ENSSSCG00000025046|	↓	-0.92	2.12E-04	2.95E-02
chr12:62264035-62264555	ENSSSCG00000018456|SNORD49	↓	-0.36	5.45E-04	6.05E-02
chr12:62264035-62264555	ENSSSCG00000018462|SNORD65	↓	-0.36	5.45E-04	6.05E-02
chr12:62818005-62818149	ENSSSCG00000018050|	↑	1.20	1.88E-04	2.67E-02
chr12:8115785-8116187	ENSSSCG00000017250|SLC39A11	↓	-0.64	7.12E-05	1.28E-02
chr12:9036007-9036495	ENSSSCG00000017251|SOX9	↑	0.65	1.97E-04	2.78E-02
chr12:958455-958928	ENSSSCG00000024018|SLC16A3	↑	0.61	3.54E-04	4.36E-02
chr13:119394519-119395142	ENSSSCG00000011751|TMEM212	↓	-0.71	5.59E-04	9.45E-02
chr13:12058359-12058972	ENSSSCG00000026488|	↓	-0.70	5.40E-04	9.27E-02
chr13:122422432-122423287	ENSSSCG00000023871|NAALADL2	↓	-0.64	3.72E-04	7.53E-02
chr13:130794706-130795293	ENSSSCG00000022926|SNORA63	↓	-0.59	7.72E-05	2.69E-02

chr13:130794706-130795293	ENSSSCG00000020700|SNORA63	↓	-0.59	7.72E-05	2.69E-02
chr13:130794706-130795293	ENSSSCG00000027448|SNORA81	↓	-0.59	7.72E-05	2.69E-02
chr13:131575714-131576530	ENSSSCG00000029263|ssc-mir-1224	↓	-0.51	8.86E-06	5.45E-03
chr13:131690032-131690935	ENSSSCG00000021581|CLCN2	↓	-0.56	4.34E-04	8.44E-02
chr13:146919527-146922052	ENSSSCG00000011870|PDIA5	↓	-0.37	7.73E-05	2.69E-02
chr13:155162842-155163357	ENSSSCG00000023641|	↓	-1.09	2.45E-04	5.67E-02
chr13:155981255-155982673	ENSSSCG00000024429|SIDT1	↑	0.46	7.12E-05	2.51E-02
chr13:163031642-163031920	ENSSSCG00000011946|ALCAM	↓	-1.17	2.55E-04	5.84E-02
chr13:191181993-191182684	ENSSSCG00000012009|	↓	-0.72	2.24E-04	5.39E-02
chr13:204863801-204864389	ENSSSCG00000027655|	↓	-0.63	5.22E-04	9.21E-02
chr13:208168932-208170395	ENSSSCG00000012052|	↓	-0.46	7.76E-06	4.90E-03
chr13:213033381-213033850	ENSSSCG00000028428|	↑	0.85	9.30E-05	3.04E-02
chr13:23871273-23871593	ENSSSCG00000025502|	↓	-1.05	3.47E-04	7.23E-02
chr13:25002024-25002924	ENSSSCG00000028740|CTDSPL	↓	-0.67	1.79E-04	4.60E-02
chr13:30527257-30528115	ENSSSCG00000022195|ZNF167	↑	0.63	5.30E-04	9.21E-02
chr13:36024916-36027454	ENSSSCG00000011411|TMEM115	↓	-0.27	5.43E-04	9.28E-02
chr13:37327790-37328196	ENSSSCG00000027573|RPL29	↓	-1.03	5.30E-04	9.21E-02
chr13:37327790-37328196	ENSSSCG00000025477|U6	↓	-1.03	5.30E-04	9.21E-02
chr13:89275482-89275938	ENSSSCG00000011667|	↑	1.02	1.20E-04	3.66E-02
chr13:97333050-97333645	ENSSSCG00000025293|HLTF	↓	-1.79	3.96E-13	2.25E-09
chr13:97333761-97334049	ENSSSCG00000025293|HLTF	↓	-1.39	2.68E-05	1.28E-02
chr14:118349987-118350369	ENSSSCG00000010518|EXOSC1	↑	1.04	1.50E-04	3.57E-02
chr14:12388001-12388504	ENSSSCG00000009665|CHRNA2	↓	-0.60	2.94E-05	1.05E-02
chr14:12388001-12388504	ENSSSCG00000009666|EPHX2	↓	-0.60	2.94E-05	1.05E-02
chr14:132404047-132405639	ENSSSCG00000010629|ADRA2A	↓	-1.09	5.79E-09	1.09E-05
chr14:135864234-135864831	ENSSSCG00000010650|AFAP1L2	↓	-0.83	3.46E-05	1.17E-02
chr14:145186837-145189206	ENSSSCG00000029482|CHST15	↑	0.35	3.82E-06	2.13E-03
chr14:149002153-149003009	ENSSSCG00000010755|PTPRE	↓	-0.46	6.50E-04	8.67E-02
chr14:152932107-152939378	ENSSSCG00000010768|	↓	-0.13	3.67E-05	1.23E-02
chr14:15483332-15483747	ENSSSCG00000009692|PINX1	↓	-0.76	6.00E-04	8.26E-02
chr14:16279463-16280541	ENSSSCG00000021726|RNaseP_nuc	↓	-0.72	4.23E-08	5.50E-05
chr14:17532357-17533224	ENSSSCG00000009703|HAND2	↑	1.26	9.21E-08	1.04E-04
chr14:17537156-17537500	ENSSSCG00000009703|HAND2	↑	1.12	2.85E-04	5.25E-02
chr14:17538467-17539013	ENSSSCG00000009703|HAND2	↑	1.37	1.31E-06	8.52E-04
chr14:17936020-17936469	ENSSSCG00000009705|	↑	0.85	2.03E-04	4.32E-02
chr14:31323331-31323952	ENSSSCG00000009775|CDK2AP1	↓	-0.73	2.47E-04	4.91E-02
chr14:31340861-31341984	ENSSSCG00000009774|C12orf65	↓	-0.50	8.16E-07	5.83E-04
chr14:3264853-3266822	ENSSSCG00000009592|NFIL3	↑	0.24	1.92E-04	4.17E-02
chr14:47991496-47991937	ENSSSCG00000009966|MN1	↓	-1.30	4.88E-12	1.77E-08
chr14:50273300-50273819	ENSSSCG00000009996|LIF	↓	-0.92	2.79E-05	1.01E-02
chr14:53070613-53071540	ENSSSCG00000010056|GGT1	↑	0.44	3.37E-04	5.86E-02
chr14:53358528-53359204	ENSSSCG00000010073|CHCHD10	↓	-0.66	2.96E-05	1.05E-02
chr14:53358528-53359204	ENSSSCG00000010071|MMP11	↓	-0.66	2.96E-05	1.05E-02
chr14:53645436-53646475	ENSSSCG00000030379|YPEL1	↑	0.52	7.57E-04	9.58E-02
chr14:54883483-54884316	ENSSSCG00000010128|SEPT5	↓	-0.57	9.80E-08	1.08E-04
chr14:59029347-59029573	ENSSSCG00000023708|	↑	1.30	4.15E-04	6.76E-02
chr14:64072444-64072747	ENSSSCG00000010179|ARV1	↑	1.02	5.83E-04	8.16E-02

chr14:64072444-64072747	ENSSSCG00000010180|TTC13	↑	1.02	5.83E-04	8.16E-02
chr14:64398057-64398498	ENSSSCG00000010184|AGT	↑	1.04	7.69E-04	9.62E-02
chr14:6842235-6842902	ENSSSCG00000009616|HR	↓	-0.45	2.84E-05	1.02E-02
chr14:68684928-68685716	ENSSSCG00000010212|ANK3	↓	-0.78	2.21E-04	4.55E-02
chr14:7282955-7283843	ENSSSCG00000009626|SORBS3	↓	-0.48	7.77E-04	9.70E-02
chr14:77677881-77678307	ENSSSCG00000022303|SLC25A16	↓	-0.60	7.30E-04	9.37E-02
chr14:77677881-77678307	ENSSSCG00000020712|	↓	-0.60	7.30E-04	9.37E-02
chr14:78775350-78776322	ENSSSCG00000010256|COL13A1	↓	-0.75	6.52E-07	4.93E-04
chr14:79767156-79767558	ENSSSCG00000024847|U6	↓	-0.95	1.24E-05	5.35E-03
chr14:82622276-82624172	ENSSSCG00000010307|FUT11	↑	0.31	3.23E-04	5.69E-02
chr14:881935-883082	ENSSSCG00000009580|S1PR3	↑	0.60	6.57E-05	1.95E-02
chr14:8821922-8822582	ENSSSCG00000009642|STC1	↓	-0.39	7.18E-04	9.26E-02
chr14:93516188-93516578	ENSSSCG00000010352|GRID1	↑	1.00	2.52E-04	4.92E-02
chr14:93516188-93516578	ENSSSCG00000026950|	↑	1.00	2.52E-04	4.92E-02
chr14:94992801-94993488	ENSSSCG00000020477|U3	↓	-0.91	5.35E-04	7.75E-02
chr14:9522401-9523018	ENSSSCG00000009646|ADAM7	↑	0.62	5.76E-05	1.75E-02
chr14:97811994-97812279	ENSSSCG00000010389|C10orf71	↓	-1.10	3.51E-04	6.06E-02
chr14:98572577-98572862	ENSSSCG00000010399|NCOA4	↓	-1.20	1.64E-05	6.59E-03
chr14:98590778-98591197	ENSSSCG00000010400|MSMB	↓	-1.15	8.60E-05	2.35E-02
chr15:118303363-118303821	ENSSSCG00000016119|RAPH1	↓	-1.24	1.15E-06	6.37E-04
chr15:134237288-134237662	ENSSSCG00000016205|NHEJ1	↓	-1.08	4.02E-04	6.78E-02
chr15:146036871-146037953	ENSSSCG00000016278|C2orf57	↓	-0.43	5.11E-05	1.50E-02
chr15:152121112-152122355	ENSSSCG00000026600|KLHL30	↓	-0.29	4.44E-04	7.28E-02
chr15:152577876-152578355	ENSSSCG00000016343|TWIST2	↓	-1.04	6.32E-04	9.22E-02
chr15:152584095-152585137	ENSSSCG00000016343|TWIST2	↑	0.48	4.12E-04	6.90E-02
chr15:154233948-154236860	ENSSSCG00000026309|	↓	-0.34	1.59E-06	8.22E-04
chr15:155451202-155457529	ENSSSCG00000021922|	↓	-0.12	1.74E-05	6.63E-03
chr15:22098868-22099776	ENSSSCG00000015706|LYPD1	↑	1.57	4.22E-07	2.59E-04
chr15:38047782-38048770	ENSSSCG00000029796|KBTBD11	↓	-0.87	2.15E-05	7.57E-03
chr15:52915254-52915760	ENSSSCG00000015790|SLC25A4	↑	0.87	3.51E-04	6.15E-02
chr15:53329672-53330640	ENSSSCG00000015796|PDLIM3	↓	-0.88	9.51E-05	2.40E-02
chr15:54672443-54672927	ENSSSCG00000028592|TM2D2	↓	-1.27	2.34E-06	1.12E-03
chr15:55482454-55483096	ENSSSCG00000015817|	↓	-1.24	9.22E-07	5.20E-04
chr15:55736223-55736948	ENSSSCG00000015825|GPR124	↓	-0.62	3.08E-04	5.69E-02
chr15:55814330-55816217	ENSSSCG00000015828|ZNF703	↑	0.35	1.26E-04	2.94E-02
chr15:71749231-71750632	ENSSSCG00000015874|ACVR1	↓	-0.51	2.06E-04	4.36E-02
chr15:86678115-86678644	ENSSSCG00000015944|TLK1	↓	-0.98	1.35E-05	5.42E-03
chr15:91356348-91356735	ENSSSCG00000023146|EVX2	↓	-1.46	1.84E-06	9.03E-04
chr15:91356348-91356735	ENSSSCG00000015979|HOXD13	↓	-1.46	1.84E-06	9.03E-04
chr15:91369015-91369433	ENSSSCG00000015980|HOXD12	↓	-0.91	5.40E-04	8.31E-02
chr15:91377308-91378073	ENSSSCG00000015981|HOXD10	↓	-1.32	3.50E-10	4.45E-07
chr15:91392209-91393390	ENSSSCG00000015983|HOXD8	↓	-1.93	3.33E-26	1.69E-22
chr15:91393905-91394559	ENSSSCG00000015983|HOXD8	↓	-1.19	8.59E-09	8.73E-06
chr15:91396125-91397757	ENSSSCG00000015983|HOXD8	↓	-1.75	1.53E-14	3.12E-11
chr15:91415665-91417285	ENSSSCG00000015984|HOXD4	↓	-2.07	1.72E-32	2.62E-28
chr15:91415665-91417285	ENSSSCG00000019942|ssc-mir-10b	↓	-2.07	1.72E-32	2.62E-28
chr15:91418147-91418787	ENSSSCG00000015984|HOXD4	↓	-0.93	2.81E-05	8.84E-03

chr15:91418147-91418787	ENSSSCG00000019942|ssc-mir-10b	↓	-0.93	2.81E-05	8.84E-03
chr15:91419540-91421853	ENSSSCG00000015984|HOXD4	↓	-2.19	6.74E-28	6.85E-24
chr15:91427408-91428894	ENSSSCG00000015985|HOXD3	↓	-1.85	3.72E-16	9.45E-13
chr15:91428908-91430392	ENSSSCG00000015985|HOXD3	↓	-1.86	3.28E-26	1.69E-22
chr15:91430932-91432101	ENSSSCG00000015985|HOXD3	↓	-1.66	1.81E-16	5.01E-13
chr15:91433788-91434591	ENSSSCG00000015985|HOXD3	↓	-1.48	4.52E-18	1.53E-14
chr15:91447031-91447425	ENSSSCG00000015986|HOXD1	↓	-1.61	3.75E-06	1.68E-03
chr16:18896302-18897687	ENSSSCG00000030991|PDZD2	↓	-0.54	1.88E-06	6.14E-04
chr16:21031067-21031470	ENSSSCG00000022589|	↑	1.12	2.46E-04	3.45E-02
chr16:24680563-24681647	ENSSSCG00000016848|EGFLAM	↓	-0.65	6.09E-05	1.16E-02
chr16:25302648-25303287	ENSSSCG00000016851|OSMR	↓	-0.66	4.24E-04	4.91E-02
chr16:26463062-26463630	ENSSSCG00000019345|5S_rRNA	↓	-0.75	1.24E-04	2.04E-02
chr16:29283827-29284909	ENSSSCG00000016868|	↓	-0.98	5.02E-07	2.13E-04
chr16:29289461-29290205	ENSSSCG00000016868|	↓	-0.67	7.40E-04	7.13E-02
chr16:29518279-29518986	ENSSSCG00000016873|	↓	-0.72	7.31E-04	7.11E-02
chr16:32563250-32563601	ENSSSCG00000016883|ISL1	↑	1.91	2.14E-11	2.78E-08
chr16:32563879-32565177	ENSSSCG00000016883|ISL1	↑	1.67	9.99E-14	2.12E-10
chr16:38807362-38808121	ENSSSCG00000024319|SNORA42	↓	-0.43	8.03E-04	7.49E-02
chr16:51600864-51601417	ENSSSCG00000028786|GTF2H2	↓	-0.87	5.85E-06	1.61E-03
chr16:58029185-58029900	ENSSSCG00000017006|LCP2	↑	1.11	2.63E-05	5.74E-03
chr16:6499572-6500072	ENSSSCG00000016794|MYO10	↓	-0.95	6.84E-06	1.83E-03
chr16:71913644-71914404	ENSSSCG00000023210|	↑	0.48	6.23E-04	6.43E-02
chr16:80836916-80838562	ENSSSCG00000027191|	↑	0.33	1.05E-03	8.60E-02
chr16:81606336-81607236	ENSSSCG00000017102|PAPD7	↓	-0.62	1.28E-03	9.92E-02
chr16:83520036-83520500	ENSSSCG00000017110|	↓	-1.00	5.23E-04	5.68E-02
chr16:85900807-85902418	ENSSSCG00000017119|	↓	-0.28	1.09E-06	3.79E-04
chr17:11345322-11345539	ENSSSCG00000007010|ZMAT4	↓	-1.08	3.81E-04	6.43E-02
chr17:14553288-14553541	ENSSSCG00000024817|RASSF2	↓	-1.26	1.36E-04	3.40E-02
chr17:18873223-18873601	ENSSSCG00000027439|HAO1	↓	-0.54	6.55E-04	9.12E-02
chr17:36323348-36323847	ENSSSCG00000007146|SIGLEC1	↑	0.60	1.53E-04	3.67E-02
chr17:42185938-42187088	ENSSSCG00000021769|	↑	0.33	5.11E-04	7.82E-02
chr17:43916954-43917359	ENSSSCG00000023261|SSC.28726	↓	-0.60	5.14E-04	7.82E-02
chr17:48702830-48703418	ENSSSCG00000030898|MAFB	↑	1.32	2.01E-06	1.19E-03
chr17:48703534-48704027	ENSSSCG00000030898|MAFB	↑	0.88	1.74E-04	4.03E-02
chr17:48704370-48704678	ENSSSCG00000030898|MAFB	↑	0.91	4.17E-04	6.90E-02
chr17:5094639-5095025	ENSSSCG00000006980|FGF20	↑	0.86	1.87E-04	4.18E-02
chr17:53726868-53727315	ENSSSCG00000007431|CTSA	↑	0.65	1.98E-04	4.32E-02
chr17:53726868-53727315	ENSSSCG00000007429|SPATA25	↑	0.65	1.98E-04	4.32E-02
chr17:53813931-53814365	ENSSSCG00000007436|MMP9	↑	0.73	4.23E-04	6.92E-02
chr17:59923255-59926231	ENSSSCG00000007481|ZFP64	↑	0.32	4.86E-05	1.58E-02
chr17:64841666-64843237	ENSSSCG00000007501|BMP7	↓	-0.55	1.06E-05	4.64E-03
chr17:65648847-65649497	ENSSSCG00000007511|ANKRD60	↑	0.34	5.34E-05	1.71E-02
chr17:65889750-65891179	ENSSSCG00000007515|APCDD1L	↓	-0.28	1.38E-04	3.40E-02
chr17:66414708-66415982	ENSSSCG00000007520|GNAS	↓	-0.64	2.04E-07	1.82E-04
chr17:68889406-68892820	ENSSSCG00000025784|CDH4	↓	-0.20	7.18E-06	3.51E-03
chr18:12820631-12820902	ENSSSCG00000028951|	↓	-1.36	5.06E-10	9.13E-07
chr18:20760298-20760870	ENSSSCG00000016573|IRF5	↑	0.51	1.83E-06	1.18E-03

chr18:20888056-20888832	ENSSSCG00000023540|	↓	-0.24	2.42E-04	5.28E-02
chr18:21043738-21044181	ENSSSCG00000016585|IMPDH1	↓	-0.78	8.17E-05	2.24E-02
chr18:27540413-27540621	ENSSSCG00000016618|CPED1	↓	-1.23	5.71E-04	8.53E-02
chr18:36943732-36944187	ENSSSCG00000016649|	↓	-0.48	6.57E-04	9.35E-02
chr18:42459008-42459249	ENSSSCG00000016662|TBX20	↑	2.47	3.06E-11	6.38E-08
chr18:42459356-42459711	ENSSSCG00000016662|TBX20	↑	1.79	1.46E-12	5.64E-09
chr18:42459803-42460125	ENSSSCG00000016662|TBX20	↑	1.05	4.06E-04	6.99E-02
chr18:42461249-42461824	ENSSSCG00000016662|TBX20	↑	1.22	1.83E-09	2.91E-06
chr18:42461841-42462696	ENSSSCG00000016662|TBX20	↑	2.01	2.60E-16	1.76E-12
chr18:42465700-42465970	ENSSSCG00000016662|TBX20	↑	1.44	2.35E-05	9.62E-03
chr18:42467345-42467653	ENSSSCG00000016662|TBX20	↑	1.14	6.59E-05	1.98E-02
chr18:50014116-50015260	ENSSSCG00000016698|HOXA11	↓	-0.74	7.78E-05	2.20E-02
chr18:50016609-50017276	ENSSSCG00000016698|HOXA11	↓	-1.56	2.80E-05	1.05E-02
chr18:50024067-50024353	ENSSSCG00000021204|	↓	-1.49	1.83E-06	1.18E-03
chr18:50028086-50028506	ENSSSCG00000026322|ssc-mir-196b-1	↓	-1.71	7.63E-08	7.37E-05
chr18:50030428-50030914	ENSSSCG00000026322|ssc-mir-196b-1	↓	-1.50	7.25E-08	7.27E-05
chr18:50031124-50031838	ENSSSCG00000026322|ssc-mir-196b-1	↓	-2.02	5.03E-11	9.72E-08
chr18:50056659-50057053	ENSSSCG00000016703|HOXA5	↑	1.25	3.08E-06	1.73E-03
chr18:50056659-50057053	ENSSSCG00000016702|HOXA6	↑	1.25	3.08E-06	1.73E-03
chr18:50057118-50057405	ENSSSCG00000016703|HOXA5	↑	2.19	5.61E-10	9.49E-07
chr18:50057118-50057405	ENSSSCG00000016702|HOXA6	↑	2.19	5.61E-10	9.49E-07
chr18:50058249-50058464	ENSSSCG00000016703|HOXA5	↑	1.63	1.44E-05	6.83E-03
chr18:50058249-50058464	ENSSSCG00000016702|HOXA6	↑	1.63	1.44E-05	6.83E-03
chr18:50058538-50058831	ENSSSCG00000016703|HOXA5	↑	1.42	2.26E-06	1.36E-03
chr18:50058538-50058831	ENSSSCG00000016702|HOXA6	↑	1.42	2.26E-06	1.36E-03
chr18:50060829-50062868	ENSSSCG00000016703|HOXA5	↑	0.42	2.58E-04	5.37E-02
chr18:50066403-50068266	ENSSSCG00000016704|HOXA4	↑	0.69	8.97E-20	1.21E-15
chr18:50075553-50075747	ENSSSCG00000016704|HOXA4	↑	1.09	2.67E-04	5.43E-02
chr18:50086847-50087249	ENSSSCG00000016705|HOXA3	↑	1.10	3.62E-04	6.61E-02
chr18:50095682-50096557	ENSSSCG00000016706|HOXA2	↓	-1.13	9.80E-16	5.30E-12
chr18:50095682-50096557	ENSSSCG00000016705|HOXA3	↓	-1.13	9.80E-16	5.30E-12
chr18:53694314-53695397	ENSSSCG00000016725|TNS3	↓	-0.70	2.46E-04	5.28E-02
chr18:55266095-55266815	ENSSSCG00000016734|CCM2	↑	0.49	1.32E-07	1.19E-04
chr18:5886100-5886955	ENSSSCG00000016433|	↓	-1.16	7.30E-09	1.10E-05
chr18:6531571-6532335	ENSSSCG00000024070|CDK5	↓	-0.44	5.83E-04	8.66E-02
chr2:1046275-1053664	ENSSSCG00000022736|	↓	-0.19	1.91E-05	8.97E-03
chr2:11893807-11894388	ENSSSCG00000013147|FAM111B	↓	-0.93	3.57E-04	6.87E-02
chr2:140448980-140449519	ENSSSCG00000014282|IL4	↓	-0.82	8.25E-06	4.81E-03
chr2:143879120-143880326	ENSSSCG00000020087|Vault	↓	-0.44	4.29E-04	7.72E-02
chr2:14787265-14787594	ENSSSCG00000023620|	↓	-0.79	1.59E-04	4.29E-02
chr2:148422098-148422471	ENSSSCG00000026471|PCDHA3	↑	1.47	1.09E-05	5.99E-03
chr2:149257321-149257732	ENSSSCG00000023932|PCDHGB7	↓	-0.76	3.61E-05	1.44E-02
chr2:151045-152606	ENSSSCG00000012840|CD151	↓	-0.58	5.85E-09	9.97E-06
chr2:151045-152606	ENSSSCG00000012838|TSPAN4	↓	-0.58	5.85E-09	9.97E-06
chr2:15385740-15386192	ENSSSCG00000024703|	↑	0.88	1.02E-04	3.02E-02
chr2:158288832-158289045	ENSSSCG00000014445|	↑	1.09	4.50E-04	7.74E-02
chr2:15935536-15935781	ENSSSCG00000019928|SNORA20	↑	0.98	2.45E-04	5.72E-02

chr2:161501025-161501484	ENSSSCG00000029373|OR2T1	↓	-0.83	1.34E-04	3.77E-02
chr2:162290052-162291453	ENSSSCG00000024389|	↑	0.40	2.99E-04	6.33E-02
chr2:162300667-162301306	ENSSSCG00000024389|	↓	-0.47	2.94E-09	6.14E-06
chr2:162300667-162301306	ENSSSCG00000025023|	↓	-0.47	2.94E-09	6.14E-06
chr2:18943012-18943356	ENSSSCG00000013277|TP53I11	↑	0.84	6.28E-04	9.60E-02
chr2:19023954-19025765	ENSSSCG00000013278|TSPAN18	↓	-0.39	4.72E-04	8.05E-02
chr2:19411941-19413149	ENSSSCG00000020526|U5	↓	-0.56	9.78E-09	1.41E-05
chr2:29013246-29014034	ENSSSCG00000019322|SNORA18	↑	0.56	2.27E-04	5.47E-02
chr2:30159134-30159381	ENSSSCG00000013312|HIPK3	↑	0.84	4.35E-04	7.73E-02
chr2:32543451-32543862	ENSSSCG00000013327|MPPED2	↓	-1.50	2.06E-06	1.55E-03
chr2:35883330-35884258	ENSSSCG00000013337|	↓	-0.74	3.19E-05	1.31E-02
chr2:42138681-42139019	ENSSSCG00000020098|	↓	-0.86	3.00E-04	6.33E-02
chr2:50492272-50492870	ENSSSCG00000013400|	↓	-0.51	7.36E-07	6.77E-04
chr2:5488993-5491727	ENSSSCG00000012964|	↓	-0.32	3.64E-06	2.62E-03
chr2:59530316-59530775	ENSSSCG00000013886|B3GNT3	↑	0.74	2.06E-04	5.06E-02
chr2:6282481-6282749	ENSSSCG00000013011|	↑	0.78	4.02E-04	7.44E-02
chr2:66397804-66398192	ENSSSCG00000013742|NFIX	↓	-0.96	2.73E-05	1.18E-02
chr2:66402031-66402806	ENSSSCG00000013742|NFIX	↓	-0.59	3.23E-04	6.64E-02
chr2:684415-686013	ENSSSCG00000012860|ATF4	↓	-0.42	8.71E-07	7.71E-04
chr2:6866828-6867211	ENSSSCG00000013030|PRDX5	↓	-0.67	4.43E-04	7.74E-02
chr2:6866828-6867211	ENSSSCG00000013029|TRMT112	↓	-0.67	4.43E-04	7.74E-02
chr2:69767905-69768705	ENSSSCG00000013639|SLC44A2	↓	-0.60	6.65E-05	2.30E-02
chr2:70088967-70089489	ENSSSCG00000013634|YIPF2	↑	0.70	3.16E-04	6.61E-02
chr2:70357909-70358873	ENSSSCG00000013627|PRKCSH	↑	0.64	2.09E-04	5.10E-02
chr2:70357909-70358873	ENSSSCG00000013625|RGL3	↑	0.64	2.09E-04	5.10E-02
chr2:7050881-7051379	ENSSSCG00000013043|MACROD1	↑	0.86	4.24E-05	1.62E-02
chr2:71166145-71167025	ENSSSCG00000024810|PRAM1	↓	-1.27	7.57E-10	2.05E-06
chr2:71871465-71871828	ENSSSCG00000013575|RETN	↓	-1.04	5.46E-04	8.60E-02
chr2:75814645-75814865	ENSSSCG00000013472|	↓	-1.28	6.63E-05	2.30E-02
chr2:75914557-75915930	ENSSSCG00000013475|NCLN	↑	0.44	3.22E-04	6.64E-02
chr2:75914557-75915930	ENSSSCG00000013474|S1PR4	↑	0.44	3.22E-04	6.64E-02
chr2:79769142-79769854	ENSSSCG00000014012|GFPT2	↓	-0.60	8.14E-05	2.62E-02
chr2:8152039-8152990	ENSSSCG00000023571|SLC22A6	↓	-0.44	8.91E-05	2.81E-02
chr2:93783277-93783702	ENSSSCG00000014136|VCAN	↓	-1.25	4.46E-05	1.68E-02
chr3:101514259-101514689	ENSSSCG00000008446|SIX2	↑	0.71	4.99E-05	1.58E-02
chr3:101515184-101516132	ENSSSCG00000008446|SIX2	↑	1.02	1.19E-09	2.12E-06
chr3:106875768-106876103	ENSSSCG00000008474|	↓	-0.44	1.80E-04	4.10E-02
chr3:10931100-10931450	ENSSSCG00000030378|LIMK1	↓	-0.80	4.27E-04	7.04E-02
chr3:11107270-11108073	ENSSSCG00000007719|CLIP2	↓	-0.56	7.09E-07	5.34E-04
chr3:11271103-11271584	ENSSSCG00000007720|GTF2IRD1	↓	-1.23	1.58E-08	2.06E-05
chr3:11341391-11342520	ENSSSCG00000020388|	↓	-0.32	4.76E-04	7.46E-02
chr3:11342676-11343231	ENSSSCG00000020388|	↓	-0.73	1.93E-04	4.29E-02
chr3:115135644-115135935	ENSSSCG00000019689|U6	↑	1.17	7.75E-06	3.83E-03
chr3:118491974-118492247	ENSSSCG00000008548|SUPT7L	↑	0.78	6.58E-04	8.91E-02
chr3:121801414-121801919	ENSSSCG00000008582|FAM228A	↓	-0.85	2.09E-09	3.38E-06
chr3:125337324-125337620	ENSSSCG00000008596|	↑	0.88	1.46E-04	3.59E-02
chr3:128237166-128237438	ENSSSCG00000008614|SMC6	↓	-0.79	3.05E-04	5.75E-02

chr3:128403355-128403848	ENSSSCG00000008615|VSNL1	↑	0.66	1.82E-04	4.10E-02
chr3:134947589-134948404	ENSSSCG00000008637|TAF1B	↓	-0.50	4.33E-04	7.09E-02
chr3:14561609-14561996	ENSSSCG00000018380|U6	↑	0.91	4.50E-05	1.44E-02
chr3:14852692-14853148	ENSSSCG00000007727|	↑	0.82	6.66E-07	5.09E-04
chr3:14853380-14854254	ENSSSCG00000007727|	↑	1.61	5.21E-23	2.08E-18
chr3:16193148-16194172	ENSSSCG00000007730|CALN1	↓	-0.40	1.27E-04	3.25E-02
chr3:18539621-18540591	ENSSSCG00000022996|C16orf53	↓	-0.42	1.08E-05	4.89E-03
chr3:18539621-18540591	ENSSSCG00000027946|MVP	↓	-0.42	1.08E-05	4.89E-03
chr3:18869262-18869903	ENSSSCG00000027933|SBK1	↑	0.88	3.87E-08	4.71E-05
chr3:32640630-32640855	ENSSSCG00000021337|PRM1	↓	-1.06	6.95E-04	9.23E-02
chr3:40591893-40592320	ENSSSCG00000029641|ZSCAN10	↓	-1.02	7.56E-05	2.16E-02
chr3:4080691-4083445	ENSSSCG00000028248|	↓	-0.31	3.02E-06	1.83E-03
chr3:40859996-40860645	ENSSSCG00000007973|IL9R	↑	0.43	6.00E-04	8.47E-02
chr3:40859996-40860645	ENSSSCG00000007962|	↑	0.43	6.00E-04	8.47E-02
chr3:40883750-40885242	ENSSSCG00000007982|MPG	↓	-0.24	2.09E-04	4.52E-02
chr3:40883750-40885242	ENSSSCG00000007968|RHBDF1	↓	-0.24	2.09E-04	4.52E-02
chr3:41636909-41640248	ENSSSCG00000008013|ALS	↓	-0.17	5.22E-04	7.94E-02
chr3:41933735-41934167	ENSSSCG00000008032|	↓	-0.81	9.95E-07	7.19E-04
chr3:4357200-4358121	ENSSSCG00000029665|PAPOLB	↓	-0.81	3.32E-06	1.93E-03
chr3:49786356-49787826	ENSSSCG00000008137|LIMS1	↓	-0.40	1.04E-04	2.80E-02
chr3:51136119-51136965	ENSSSCG00000008143|UXS1	↑	0.51	2.12E-04	4.52E-02
chr3:51778158-51778990	ENSSSCG00000025772|GPR45	↓	-0.36	4.69E-04	7.42E-02
chr3:66670558-66670757	ENSSSCG00000023859|LRRTM1	↓	-0.89	4.64E-04	7.40E-02
chr3:7108235-7108440	ENSSSCG00000023410|	↓	-0.96	5.12E-04	7.84E-02
chr3:71809713-71810562	ENSSSCG00000023156|	↓	-0.78	4.60E-08	5.24E-05
chr3:72401839-72402211	ENSSSCG00000008299|C2orf78	↓	-0.62	7.66E-05	2.18E-02
chr3:72401839-72402211	ENSSSCG00000008298|DUSP11	↓	-0.62	7.66E-05	2.18E-02
chr3:748511-750687	ENSSSCG00000007550|COX19	↓	-0.24	1.16E-04	3.04E-02
chr3:750765-754135	ENSSSCG00000007550|COX19	↓	-0.15	5.29E-04	7.94E-02
chr3:750765-754135	ENSSSCG00000007549|CYP2W1	↓	-0.15	5.29E-04	7.94E-02
chr3:7691608-7691870	ENSSSCG00000025958|	↓	-1.36	6.38E-07	4.95E-04
chr3:7692356-7692856	ENSSSCG00000025958|	↓	-2.01	1.40E-20	1.87E-16
chr3:7692954-7693310	ENSSSCG00000025958|	↓	-2.18	4.79E-11	1.28E-07
chr3:7695006-7695799	ENSSSCG00000025958|	↓	-1.45	3.07E-12	1.03E-08
chr3:77045139-77045979	ENSSSCG00000008344|ARHGAP25	↑	1.04	5.29E-10	1.09E-06
chr3:7918190-7918608	ENSSSCG00000023255|MOSPD3	↓	-1.07	6.87E-08	7.65E-05
chr3:7918190-7918608	ENSSSCG00000027466|PCOLCE	↓	-1.07	6.87E-08	7.65E-05
chr3:7919059-7922609	ENSSSCG00000023255|MOSPD3	↑	0.67	1.16E-15	7.77E-12
chr3:8002888-8003581	ENSSSCG00000007672|POP7	↓	-0.57	5.61E-04	8.19E-02
chr3:8005352-8005856	ENSSSCG00000007673|EPO	↓	-0.95	1.01E-05	4.72E-03
chr3:818028-821537	ENSSSCG00000026422|GPER	↓	-0.18	3.42E-06	1.97E-03
chr3:818028-821537	ENSSSCG00000007552|	↓	-0.18	3.42E-06	1.97E-03
chr3:829315-829577	ENSSSCG00000026422|GPER	↓	-1.33	8.91E-06	4.25E-03
chr3:89945797-89946378	ENSSSCG00000019079|	↓	-1.00	2.77E-05	1.02E-02
chr3:9636001-9636302	ENSSSCG00000007695|	↓	-0.90	2.71E-04	5.35E-02
chr4:102515908-102516163	ENSSSCG00000006492|BGLAP	↑	0.99	5.52E-04	9.87E-02
chr4:102627458-102627756	ENSSSCG00000006495|SEMA4A	↑	1.11	8.13E-06	4.56E-03

chr4:106391378-106392708	ENSSSCG00000026427|RORC	↓	-0.44	2.74E-07	2.73E-04
chr4:106391378-106392708	ENSSSCG00000021540|	↓	-0.44	2.74E-07	2.73E-04
chr4:1100625-1101172	ENSSSCG00000028265|	↓	-0.89	1.49E-07	1.67E-04
chr4:11121279-11125125	ENSSSCG00000005961|	↓	-0.25	6.10E-08	8.29E-05
chr4:111576172-111576990	ENSSSCG00000030726|CH242-150C11.4	↑	0.82	1.55E-04	4.35E-02
chr4:114895923-114896315	ENSSSCG00000018412|U6atac	↑	0.84	1.47E-04	4.15E-02
chr4:134388949-134389262	ENSSSCG00000006888|	↓	-1.14	1.39E-05	6.96E-03
chr4:134837825-134838191	ENSSSCG00000006890|ABCA4	↓	-0.92	2.83E-04	6.58E-02
chr4:139497038-139497892	ENSSSCG00000027014|	↓	-0.61	8.89E-05	2.99E-02
chr4:141710648-141710974	ENSSSCG00000022032|	↑	1.19	1.01E-04	3.20E-02
chr4:142231712-142232106	ENSSSCG00000006935|CLCA2	↓	-0.59	4.67E-04	9.03E-02
chr4:19958855-19959234	ENSSSCG00000005997|COL14A1	↓	-0.81	3.79E-05	1.49E-02
chr4:30193577-30194043	ENSSSCG00000006023|SYBU	↑	0.87	5.06E-04	9.46E-02
chr4:56202018-56202374	ENSSSCG00000006141|CA3	↑	1.22	4.92E-05	1.81E-02
chr4:70695524-70697284	ENSSSCG00000006194|NCOA2	↓	-1.12	1.28E-11	5.72E-08
chr4:70698105-70698706	ENSSSCG00000006194|NCOA2	↓	-1.08	1.17E-07	1.42E-04
chr4:70698752-70699187	ENSSSCG00000006194|NCOA2	↓	-1.14	1.13E-08	1.88E-05
chr4:70699195-70700490	ENSSSCG00000006194|NCOA2	↓	-1.08	2.44E-15	1.83E-11
chr4:70700852-70701288	ENSSSCG00000006194|NCOA2	↓	-1.08	1.20E-06	8.93E-04
chr4:70704509-70704793	ENSSSCG00000006194|NCOA2	↓	-1.36	5.45E-06	3.44E-03
chr4:70830764-70835175	ENSSSCG00000021133|	↑	0.40	5.08E-05	1.84E-02
chr4:70835246-70837021	ENSSSCG00000021133|	↑	0.63	2.92E-07	2.79E-04
chr4:81532356-81532622	ENSSSCG00000006240|FAM110B	↑	1.27	4.32E-04	8.72E-02
chr4:98325252-98325774	ENSSSCG00000006392|IGSF8	↑	0.81	1.30E-04	3.98E-02
chr5:1034106-1035916	ENSSSCG00000024864|	↓	-0.23	1.94E-04	4.21E-02
chr5:105476904-105477519	ENSSSCG00000000938|LIN7A	↑	0.74	2.13E-04	4.45E-02
chr5:108167815-108168390	ENSSSCG00000029249|	↓	-1.19	1.34E-12	7.64E-09
chr5:110795670-110798411	ENSSSCG00000000976|MOV10L1	↓	-0.19	4.43E-04	7.25E-02
chr5:16381603-16382259	ENSSSCG00000027522|ASIC1	↑	0.74	3.84E-06	2.19E-03
chr5:17934432-17936617	ENSSSCG00000000234|GRASP	↓	-0.29	1.76E-04	3.88E-02
chr5:19657367-19657605	ENSSSCG00000030509|ssc-mir-196a-2	↓	-1.49	1.42E-06	9.36E-04
chr5:19681388-19681662	ENSSSCG00000028104|HOXC11	↓	-1.08	4.58E-04	7.37E-02
chr5:19681388-19681662	ENSSSCG00000021974|HOXC8	↓	-1.08	4.58E-04	7.37E-02
chr5:19693692-19693971	ENSSSCG00000029056|ssc-mir-615	↓	-1.81	2.72E-06	1.66E-03
chr5:19693692-19693971	ENSSSCG00000030654|	↓	-1.81	2.72E-06	1.66E-03
chr5:20459216-20459640	ENSSSCG00000000293|ITGA5	↑	1.09	3.98E-04	6.75E-02
chr5:22922172-22923213	ENSSSCG00000000374|RPS26	↓	-0.90	3.09E-04	5.65E-02
chr5:24732915-24734298	ENSSSCG00000000435|	↓	-0.83	7.94E-10	2.27E-06
chr5:25958598-25959162	ENSSSCG00000000455|LRIG3	↑	0.75	7.56E-06	3.87E-03
chr5:25959277-25960270	ENSSSCG00000000455|LRIG3	↑	0.94	1.96E-07	2.04E-04
chr5:39064054-39064244	ENSSSCG00000000515|TRH-DE	↓	-1.57	4.20E-05	1.45E-02
chr5:4360187-4360411	ENSSSCG00000000062|CSDC2	↓	-1.17	5.23E-04	7.71E-02
chr5:49155259-49156321	ENSSSCG00000000544|PTHLH	↑	0.57	2.16E-04	4.45E-02
chr5:49837684-49838213	ENSSSCG00000000550|C12orf70	↑	0.93	3.12E-05	1.14E-02
chr5:53755492-53756241	ENSSSCG00000000567|SOX5	↓	-1.25	1.21E-14	1.03E-10
chr5:5646535-5648423	ENSSSCG00000000079|	↓	-0.48	7.33E-08	9.66E-05
chr5:5651857-5652854	ENSSSCG00000000079|	↓	-0.49	1.14E-04	2.67E-02

chr5:65674575-65675162	ENSSSCG00000000669|GDF3	↓	-1.13	2.45E-04	4.86E-02
chr5:66518310-66519897	ENSSSCG00000000699|	↓	-0.42	1.04E-04	2.48E-02
chr5:68055782-68056339	ENSSSCG00000000721|DYRK4	↑	0.87	4.01E-05	1.42E-02
chr5:69904039-69904637	ENSSSCG00000030076|SLC6A13	↓	-1.29	1.52E-17	2.60E-13
chr5:72506950-72507719	ENSSSCG00000000773|	↓	-0.61	2.55E-04	4.96E-02
chr5:92304732-92305274	ENSSSCG00000000901|FGD6	↓	-0.92	3.06E-07	2.69E-04
chr5:93362950-93364078	ENSSSCG00000000907|PLXNC1	↓	-0.44	1.51E-05	6.48E-03
chr6:108114365-108114734	ENSSSCG00000023686|TTR	↓	-0.82	2.80E-05	1.13E-02
chr6:111444503-111444771	ENSSSCG00000003739|ZNF397	↓	-1.10	7.52E-05	2.42E-02
chr6:12252251-12253273	ENSSSCG00000023012|	↓	-0.73	1.14E-07	1.55E-04
chr6:126697917-126698711	ENSSSCG00000024494|ST6GALNAC3	↓	-0.79	8.62E-05	2.69E-02
chr6:135559082-135559653	ENSSSCG00000003807|DNAJC6	↓	-1.07	2.51E-06	1.75E-03
chr6:136908348-136908642	ENSSSCG00000003811|ROR1	↑	1.10	3.01E-06	2.05E-03
chr6:139746205-139746732	ENSSSCG00000003823|C1orf87	↓	-0.93	5.50E-06	3.26E-03
chr6:14186420-14186961	ENSSSCG00000022502|	↑	0.75	4.48E-04	7.85E-02
chr6:147004716-147004954	ENSSSCG00000003853|SCP2	↓	-1.07	3.76E-05	1.45E-02
chr6:154338460-154340372	ENSSSCG00000021001|U6	↓	-0.35	3.21E-06	2.14E-03
chr6:154954902-154956297	ENSSSCG00000023597|ST3GAL3	↓	-0.27	5.04E-07	5.26E-04
chr6:155085996-155087645	ENSSSCG00000021630|	↓	-0.32	6.72E-05	2.22E-02
chr6:155479786-155480910	ENSSSCG00000030177|EBNA1BP2	↓	-0.47	1.25E-04	3.41E-02
chr6:156128387-156128905	ENSSSCG00000021745|ERMAP	↓	-0.54	5.20E-04	8.48E-02
chr6:156225004-156225511	ENSSSCG00000028485|YBX1	↑	0.84	7.07E-05	2.31E-02
chr6:157370882-157371131	ENSSSCG00000003974|CITED4	↓	-0.99	2.29E-05	9.85E-03
chr6:169610-172119	ENSSSCG00000029761|	↓	-0.11	1.81E-04	4.39E-02
chr6:17581095-17582361	ENSSSCG00000002817|CCDC135	↓	-0.32	7.80E-05	2.48E-02
chr6:25130862-25131849	ENSSSCG00000002786|ELMO3	↓	-0.75	2.12E-07	2.56E-04
chr6:25130862-25131849	ENSSSCG00000002788|EXOC3L1	↓	-0.75	2.12E-07	2.56E-04
chr6:25493438-25495038	ENSSSCG00000024253|	↓	-0.38	1.81E-04	4.39E-02
chr6:25493438-25495038	ENSSSCG00000021522|	↓	-0.38	1.81E-04	4.39E-02
chr6:2748523-2750332	ENSSSCG00000002658|FOXL1	↑	0.35	2.41E-04	5.27E-02
chr6:32864894-32865174	ENSSSCG00000002847|GPT2	↓	-1.44	2.67E-07	3.10E-04
chr6:32948908-32949269	ENSSSCG00000002846|DNAJA2	↑	0.87	3.24E-04	6.42E-02
chr6:35979523-35979914	ENSSSCG00000029234|ZNF536	↑	1.01	3.00E-04	6.08E-02
chr6:372973-373913	ENSSSCG00000024687|FANCA	↓	-0.46	1.39E-05	6.71E-03
chr6:372973-373913	ENSSSCG00000002635|SPIRE2	↓	-0.46	1.39E-05	6.71E-03
chr6:40285530-40286103	ENSSSCG00000002881|FFAR2	↓	-0.71	3.91E-04	7.21E-02
chr6:41188108-41188323	ENSSSCG00000025590|ZNF461	↑	1.25	4.88E-04	8.28E-02
chr6:41188108-41188323	ENSSSCG00000002932|ZNF567	↑	1.25	4.88E-04	8.28E-02
chr6:42808923-42809725	ENSSSCG00000002959|FAM98C	↑	0.75	2.77E-05	1.12E-02
chr6:42808923-42809725	ENSSSCG00000002957|GGN	↑	0.75	2.77E-05	1.12E-02
chr6:46395572-46395931	ENSSSCG00000003066|IRGC	↑	1.26	1.12E-05	5.70E-03
chr6:47122920-47123922	ENSSSCG00000003081|CEACAM16	↓	-0.63	1.86E-06	1.39E-03
chr6:5066616-5066933	ENSSSCG00000002675|DNAAF1	↑	0.81	2.28E-04	5.14E-02
chr6:50792281-50792548	ENSSSCG00000003204|VRK3	↓	-1.00	3.96E-05	1.50E-02
chr6:50792281-50792548	ENSSSCG00000003205|ZNF473	↓	-1.00	3.96E-05	1.50E-02
chr6:5092971-5094965	ENSSSCG00000002682|MBTPS1	↓	-0.25	2.96E-04	6.05E-02
chr6:51191922-51194077	ENSSSCG00000023211|ASPDH	↓	-0.23	6.58E-04	9.67E-02

chr6:51191922-51194077	ENSSSCG00000003217|JOSD2	↓	-0.23	6.58E-04	9.67E-02
chr6:51271874-51272255	ENSSSCG00000029960|LRRC4B	↓	-0.64	9.75E-05	2.89E-02
chr6:51858139-51858968	ENSSSCG00000018644|ssc-let-7e	↓	-0.49	2.47E-04	5.35E-02
chr6:51858139-51858968	ENSSSCG00000019341|ssc-mir-125a	↓	-0.49	2.47E-04	5.35E-02
chr6:51858139-51858968	ENSSSCG00000018735|ssc-mir-99b	↓	-0.49	2.47E-04	5.35E-02
chr6:51858139-51858968	ENSSSCG00000003238|	↓	-0.49	2.47E-04	5.35E-02
chr6:51882741-51883468	ENSSSCG00000003239|HAS1	↑	0.97	1.67E-04	4.14E-02
chr6:53429374-53429778	ENSSSCG00000030857|LENG8	↓	-1.01	3.57E-06	2.31E-03
chr6:54509387-54513446	ENSSSCG00000022936|FIZ1	↓	-0.26	5.59E-04	8.81E-02
chr6:54691637-54692420	ENSSSCG00000023137|	↓	-0.56	7.95E-07	7.55E-04
chr6:58302067-58302944	ENSSSCG00000003343|	↓	-0.37	5.62E-04	8.81E-02
chr6:59764555-59765122	ENSSSCG00000024223|ARHGEF16	↓	-0.70	2.52E-05	1.07E-02
chr6:59922418-59922741	ENSSSCG00000019934|ssc-mir-551a	↓	-1.03	4.78E-04	8.16E-02
chr6:59924794-59925669	ENSSSCG00000019934|ssc-mir-551a	↓	-0.56	1.47E-04	3.78E-02
chr6:61907532-61907816	ENSSSCG00000024843|	↑	1.02	6.42E-04	9.54E-02
chr6:62280244-62280535	ENSSSCG00000029135|TNFRSF25	↑	0.73	4.19E-04	7.53E-02
chr6:65246643-65249000	ENSSSCG00000003414|ANGPTL7	↑	0.28	7.18E-05	2.32E-02
chr6:65249432-65249928	ENSSSCG00000003414|ANGPTL7	↑	0.54	3.97E-06	2.51E-03
chr6:65252983-65253641	ENSSSCG00000003414|ANGPTL7	↓	-0.35	2.28E-04	5.14E-02
chr6:70311267-70311665	ENSSSCG00000003484|PADI6	↑	0.83	2.62E-04	5.65E-02
chr6:73147788-73148402	ENSSSCG00000003505|DDOST	↓	-0.54	8.91E-05	2.72E-02
chr6:74019683-74020033	ENSSSCG00000003519|	↑	0.83	5.89E-04	9.06E-02
chr6:74750406-74751230	ENSSSCG00000003527|EPHB2	↑	0.59	7.44E-07	7.17E-04
chr6:7677607-7679094	ENSSSCG00000027458|CDYL2	↓	-0.39	9.98E-06	5.35E-03
chr6:7680217-7681029	ENSSSCG00000027458|CDYL2	↓	-0.69	3.17E-08	6.62E-05
chr6:79917664-79918500	ENSSSCG00000003587|TMEM200B	↑	0.97	1.02E-09	3.56E-06
chr6:81652660-81652858	ENSSSCG00000023946|ZCCHC17	↑	1.33	1.95E-04	4.58E-02
chr6:83053500-83054390	ENSSSCG00000021232|SYNC	↓	-0.30	4.97E-04	8.33E-02
chr6:83651247-83651510	ENSSSCG00000027035|	↓	-1.14	2.76E-04	5.79E-02
chr6:86802612-86803819	ENSSSCG00000003646|POU3F1	↓	-0.33	3.76E-06	2.41E-03
chr6:918869-920676	ENSSSCG00000002642|CDH15	↓	-0.36	5.15E-06	3.07E-03
chr6:99933729-99934276	ENSSSCG00000003703|POLR2J	↑	0.67	1.88E-04	4.47E-02
chr7:102682137-102683653	ENSSSCG00000002346|PNMA1	↓	-0.40	2.08E-07	1.87E-04
chr7:105626257-105626520	ENSSSCG00000002388|ESRRB	↓	-0.94	7.68E-04	8.99E-02
chr7:105892331-105892792	ENSSSCG00000018299|	↓	-1.19	2.09E-06	1.11E-03
chr7:106003867-106004240	ENSSSCG00000002391|C14orf166B	↓	-0.50	4.18E-04	6.33E-02
chr7:106257121-106257445	ENSSSCG00000026481|ZDHHC22	↓	-0.72	6.93E-04	8.53E-02
chr7:108478626-108478978	ENSSSCG00000028718|	↑	0.96	4.05E-04	6.18E-02
chr7:120208887-120209196	ENSSSCG00000002445|TRIP11	↓	-0.70	7.02E-04	8.60E-02
chr7:122914410-122914666	ENSSSCG00000002481|SERPINA5	↓	-1.54	4.62E-06	2.19E-03
chr7:123331023-123331574	ENSSSCG00000026899|	↓	-0.75	1.64E-10	4.45E-07
chr7:123632328-123632569	ENSSSCG00000028225|DICER1	↑	0.94	5.16E-04	7.10E-02
chr7:128943894-128944505	ENSSSCG00000002525|TRAF3	↓	-0.30	3.03E-04	5.14E-02
chr7:130395365-130396684	ENSSSCG00000021294|	↓	-0.48	1.32E-06	7.91E-04
chr7:133966941-133967678	ENSSSCG00000002620|EFHC1	↓	-0.46	2.77E-04	4.81E-02
chr7:1385776-1387222	ENSSSCG00000000994|GMDS	↓	-0.49	3.42E-04	5.50E-02
chr7:14422519-14422838	ENSSSCG00000025400|U6	↑	0.99	5.90E-04	7.66E-02

chr7:28004578-28006837	ENSSSCG00000001433|DUROC-PPT2	↓	-0.40	4.12E-05	1.26E-02
chr7:28076488-28076938	ENSSSCG00000001440|DUROC-NOTCH4	↓	-0.84	6.48E-04	8.11E-02
chr7:29186606-29187754	ENSSSCG00000030750|DUROC-SLA-DQB2	↑	0.49	6.83E-04	8.42E-02
chr7:29188204-29188678	ENSSSCG00000001457|DUROC-SLA-DQB1	↑	0.88	1.91E-04	3.80E-02
chr7:29188204-29188678	ENSSSCG00000030750|DUROC-SLA-DQB2	↑	0.88	1.91E-04	3.80E-02
chr7:33919375-33919724	ENSSSCG00000001500|COL9A3	↓	-0.79	1.60E-04	3.35E-02
chr7:34180610-34181265	ENSSSCG00000001507|TAPBP	↓	-0.38	5.58E-04	7.37E-02
chr7:34180610-34181265	ENSSSCG00000001508|ZBTB22	↓	-0.38	5.58E-04	7.37E-02
chr7:34657296-34658090	ENSSSCG00000001520|LEMD2	↓	-0.62	8.78E-05	2.13E-02
chr7:36223982-36225900	ENSSSCG00000001539|PPARD	↓	-0.42	1.11E-09	2.25E-06
chr7:36518675-36519194	ENSSSCG00000001550|ARMC12	↓	-0.53	3.63E-05	1.14E-02
chr7:43344237-43344714	ENSSSCG00000001646|KIAA0240	↓	-0.86	3.97E-05	1.23E-02
chr7:43630250-43630915	ENSSSCG00000001658|MRPL2	↑	0.71	1.88E-04	3.76E-02
chr7:44069177-44070571	ENSSSCG00000001688|	↑	0.24	7.77E-04	9.08E-02
chr7:4580107-4582780	ENSSSCG00000001021|RREB1	↓	-0.28	1.91E-06	1.04E-03
chr7:47069040-47069682	ENSSSCG00000020083|U6	↓	-0.91	1.76E-06	9.70E-04
chr7:5010815-5011121	ENSSSCG00000021528|U6	↑	0.98	5.85E-04	7.62E-02
chr7:53946787-53946993	ENSSSCG00000001772|ANKRD34C	↑	1.10	2.23E-04	4.20E-02
chr7:55574663-55574923	ENSSSCG00000001787|IL16	↓	-1.40	1.49E-05	5.65E-03
chr7:56736920-56737375	ENSSSCG00000001794|	↑	0.83	1.95E-05	6.89E-03
chr7:58190185-58190750	ENSSSCG00000001814|	↓	-0.72	6.09E-05	1.62E-02
chr7:60570030-60570464	ENSSSCG00000029010|	↓	-0.37	6.08E-04	7.81E-02
chr7:66522123-66522570	ENSSSCG00000023760|CLEC14A	↓	-0.90	1.31E-04	2.90E-02
chr7:66598228-66598556	ENSSSCG00000001938|SSTR1	↓	-1.19	2.19E-05	7.52E-03
chr7:69730532-69730730	ENSSSCG00000019709|U2	↑	1.08	7.03E-04	8.60E-02
chr7:70018206-70019949	ENSSSCG00000001960|EAPP	↑	0.27	7.98E-05	2.02E-02
chr7:70018206-70019949	ENSSSCG00000027708|U1	↑	0.27	7.98E-05	2.02E-02
chr7:70018206-70019949	ENSSSCG00000024478|U1	↑	0.27	7.98E-05	2.02E-02
chr7:70666341-70667030	ENSSSCG00000001963|EGLN3	↑	0.82	4.29E-05	1.28E-02
chr7:70668133-70668543	ENSSSCG00000001963|EGLN3	↑	1.21	2.10E-07	1.87E-04
chr7:80250168-80251713	ENSSSCG00000001989|CIDE-B	↓	-0.52	3.08E-04	5.20E-02
chr7:80250168-80251713	ENSSSCG00000027911|LTB4R	↓	-0.52	3.08E-04	5.20E-02
chr7:80250168-80251713	ENSSSCG00000020941|LTB4R2	↓	-0.52	3.08E-04	5.20E-02
chr7:80255116-80255382	ENSSSCG00000001991|DHRS1	↑	1.06	5.49E-04	7.37E-02
chr7:80255116-80255382	ENSSSCG00000027911|LTB4R	↑	1.06	5.49E-04	7.37E-02
chr7:80255116-80255382	ENSSSCG00000020941|LTB4R2	↑	1.06	5.49E-04	7.37E-02
chr7:81322908-81323632	ENSSSCG00000002037|CDH24	↓	-0.30	9.88E-05	2.39E-02
chr7:81322908-81323632	ENSSSCG00000002036|	↓	-0.30	9.88E-05	2.39E-02
chr7:81439627-81439916	ENSSSCG00000002040|MRPL52	↑	0.98	1.18E-04	2.76E-02
chr7:81439627-81439916	ENSSSCG00000002041|SLC7A7	↑	0.98	1.18E-04	2.76E-02
chr7:86648521-86648818	ENSSSCG00000026370|	↑	0.87	7.84E-04	9.13E-02
chr7:88974981-88975449	ENSSSCG00000002254|NR2F2	↑	1.38	6.32E-10	1.40E-06
chr7:88975502-88976275	ENSSSCG00000002254|NR2F2	↑	2.00	1.21E-20	1.97E-16
chr8:103641626-103642106	ENSSSCG00000020999|LARP1B	↓	-1.58	1.05E-08	1.35E-05
chr8:103643254-103644304	ENSSSCG00000020999|LARP1B	↓	-0.97	2.61E-09	3.95E-06
chr8:103645981-103647253	ENSSSCG00000020999|LARP1B	↓	-1.14	5.05E-20	2.93E-16
chr8:103647507-103647985	ENSSSCG00000020999|LARP1B	↓	-1.26	1.01E-05	4.97E-03

chr8:103648394-103649009	ENSSSCG00000020999|LARP1B	↓	-1.40	2.01E-07	1.95E-04
chr8:115965146-115965663	ENSSSCG00000025714|UGT8	↑	0.81	6.13E-04	8.75E-02
chr8:119909380-119913356	ENSSSCG00000009131|PITX2	↑	2.07	9.11E-24	1.06E-19
chr8:119913677-119914112	ENSSSCG00000009131|PITX2	↑	1.30	9.59E-06	4.77E-03
chr8:119914134-119914526	ENSSSCG00000009131|PITX2	↑	1.96	6.37E-09	9.25E-06
chr8:119914573-119915904	ENSSSCG00000009131|PITX2	↑	2.07	1.29E-28	4.51E-24
chr8:119918692-119919119	ENSSSCG00000009131|PITX2	↑	1.40	4.11E-06	2.53E-03
chr8:119921081-119922011	ENSSSCG00000009131|PITX2	↑	1.79	3.33E-13	1.05E-09
chr8:145839476-145840541	ENSSSCG00000029507|RASGEF1B	↓	-0.48	4.94E-04	7.64E-02
chr8:147374817-147375606	ENSSSCG00000018854|U2	↓	-0.53	3.30E-07	2.87E-04
chr8:19981541-19981927	ENSSSCG00000008759|	↓	-1.53	5.12E-07	4.05E-04
chr8:20641350-20641691	ENSSSCG00000027005|	↓	-1.09	1.08E-05	5.10E-03
chr8:30396718-30397005	ENSSSCG00000008770|KIAA1239	↑	1.18	1.96E-04	4.26E-02
chr8:40578382-40579058	ENSSSCG00000018511|U6	↓	-0.74	4.74E-04	7.47E-02
chr8:41327380-41328066	ENSSSCG00000008833|SGCB	↑	0.69	4.77E-04	7.49E-02
chr8:42923290-42924231	ENSSSCG00000008840|GSX2	↓	-0.33	2.20E-04	4.65E-02
chr8:43918441-43919012	ENSSSCG00000008843|	↑	1.09	5.30E-06	2.88E-03
chr8:4488508-4488940	ENSSSCG00000008723|HTRA3	↑	1.06	5.03E-04	7.72E-02
chr8:57629573-57630056	ENSSSCG00000008898|HOPX	↓	-1.05	4.95E-05	1.53E-02
chr8:58425419-58425875	ENSSSCG00000022882|	↓	-1.05	4.42E-06	2.57E-03
chr8:72672745-72673132	ENSSSCG00000008946|ADAMTS3	↓	-0.85	4.20E-04	6.94E-02
chr8:74074955-74077166	ENSSSCG00000008953|IL8	↓	-0.40	2.56E-04	5.00E-02
chr8:76749135-76749509	ENSSSCG00000008985|SOWAHB	↓	-0.76	3.92E-04	6.63E-02
chr8:76749135-76749509	ENSSSCG00000019002|U1	↓	-0.76	3.92E-04	6.63E-02
chr8:80715855-80716413	ENSSSCG00000009011|FHDC1	↓	-1.15	1.38E-06	9.43E-04
chr8:83721003-83721707	ENSSSCG00000009023|DCLK2	↓	-0.74	2.05E-05	7.94E-03
chr8:839342-839862	ENSSSCG00000008689|ZFYVE28	↓	-0.70	9.58E-05	2.51E-02
chr9:10812000-10812720	ENSSSCG00000014855|RPS3	↓	-0.59	9.31E-06	5.05E-03
chr9:10812000-10812720	ENSSSCG00000025715|SNORD15	↓	-0.59	9.31E-06	5.05E-03
chr9:113109596-113110370	ENSSSCG00000015412|CCDC146	↓	-0.47	9.45E-07	7.72E-04
chr9:126103102-126103429	ENSSSCG00000018758|ssc-mir-214	↓	-1.32	2.50E-07	2.50E-04
chr9:126105203-126105786	ENSSSCG00000019027|ssc-mir-199a-1	↓	-1.39	7.62E-09	1.40E-05
chr9:126105203-126105786	ENSSSCG00000018758|ssc-mir-214	↓	-1.39	7.62E-09	1.40E-05
chr9:133594768-133595121	ENSSSCG00000015535|QSOX1	↓	-0.88	1.90E-04	4.51E-02
chr9:135106522-135107121	ENSSSCG00000015543|CACNA1E	↓	-0.60	1.36E-07	1.42E-04
chr9:135470364-135471380	ENSSSCG00000018414|	↑	0.39	4.25E-04	7.54E-02
chr9:143443056-143444069	ENSSSCG00000015589|VASH2	↓	-0.52	3.22E-04	6.19E-02
chr9:146403053-146403663	ENSSSCG00000015611|	↓	-0.59	2.49E-05	1.01E-02
chr9:22527755-22528222	ENSSSCG00000014917|CCDC81	↓	-0.67	2.34E-04	5.03E-02
chr9:36577840-36578438	ENSSSCG00000014978|ANGPTL5	↓	-1.29	4.42E-09	8.48E-06
chr9:36579162-36579667	ENSSSCG00000014978|ANGPTL5	↓	-0.92	3.44E-07	3.30E-04
chr9:45814830-45815651	ENSSSCG00000015047|TTC12	↓	-0.41	1.29E-04	3.30E-02
chr9:46155237-46155947	ENSSSCG00000015049|	↓	-0.58	3.02E-04	5.92E-02
chr9:46156490-46156863	ENSSSCG00000015049|	↑	1.06	2.43E-05	1.00E-02
chr9:46999865-47001457	ENSSSCG00000015059|	↓	-0.49	7.72E-06	4.48E-03
chr9:50309902-50312085	ENSSSCG00000021548|	↓	-1.38	6.59E-56	2.90E-51
chr9:50312125-50312636	ENSSSCG00000021548|	↓	-0.99	4.81E-05	1.64E-02

chr9:50312892-50314245	ENSSSCG00000021548|	↓	-0.88	2.86E-15	4.21E-11
chr9:50315701-50316533	ENSSSCG00000021548|	↓	-0.79	2.52E-05	1.02E-02
chr9:5148875-5149558	ENSSSCG00000014688|	↓	-0.63	1.45E-04	3.65E-02
chr9:53416820-53417196	ENSSSCG00000024215|TECTA	↑	0.85	4.47E-04	7.79E-02
chr9:62597190-62597850	ENSSSCG00000015239|BARX2	↓	-0.76	4.05E-04	7.29E-02
chr9:72768734-72769071	ENSSSCG00000020783|SLC41A1	↑	1.05	4.92E-04	8.25E-02
chr9:73932201-73933813	ENSSSCG00000030821|FCAMR	↓	-0.53	1.26E-05	6.24E-03
chr9:77272064-77272583	ENSSSCG00000015307|	↓	-0.68	5.01E-04	8.33E-02
chr9:80651755-80652273	ENSSSCG00000015322|TFPI2	↓	-0.74	7.36E-05	2.21E-02
chr9:85498509-85498757	ENSSSCG00000015342|COL28A1	↑	1.22	4.71E-04	8.01E-02
chr9:8872440-8873507	ENSSSCG00000014827|PLEKHB1	↓	-0.42	2.02E-04	4.65E-02
chr9:95602226-95602612	ENSSSCG00000018568|	↑	1.13	5.43E-04	8.77E-02
chr9:97330184-97331311	ENSSSCG00000015370|TWIST1	↑	0.97	5.26E-07	4.93E-04
chrX:124797716-124798226	ENSSSCG00000020215|7SK	↓	-1.46	2.60E-06	1.89E-02
chrX:142168950-142171433	ENSSSCG00000012787|L1CAM	↓	-0.48	4.25E-05	7.71E-02
