# Supplementary material for: Arterial endothelial methylome: differential DNA methylation in athero-susceptible disturbed flow regions in vivo
Source: BMC Genomics. 2015 Jul 7;16:506. doi: 10.1186/s12864-015-1656-4 (PMC4492093; doi:10.1186/s12864-015-1656-4)
Supplement: Additional file 4: Table S2a. — Male-DT vs Female-DT DMR (FDR < 0.1). [file 12864_2015_1656_MOESM4_ESM.pdf]

Supplementary Table 2a. M-DT vs F-DT DMR (FDR<0.1)

DMR	Close Emsembl Genes	Human Homolog Gene	Conc	Conc_M	Conc_F		Fold, log2 (M/F)	p-value	FDR
chr1:8393356-8394063			4.8	4.2	5.2	↓	-1.1	3.2E-07	9.1E-03
chr10:17454117-17454518			3.5	2.3	4.2	↓	-1.9	8.5E-06	5.0E-02
chr10:18557823-18558179			3.9	3.0	4.4	↓	-1.4	6.6E-06	5.0E-02
chr10:44032820-44033186	ENSSSCG00000019575	U6	3.2	2.0	3.9	↓	-1.9	4.8E-06	5.0E-02
chr10:59726442-59727126			4.1	3.4	4.5	↓	-1.1	1.2E-05	5.4E-02
chr11:18715761-18716089			3.1	1.9	3.7	↓	-1.8	6.9E-06	8.8E-03
chr11:24828387-24828713			2.8	1.8	3.4	↓	-1.6	4.0E-05	3.7E-02
chr11:26540979-26541398	ENSSSCG00000026206		3.1	2.4	3.6	↓	-1.3	1.8E-04	8.2E-02
chr11:3988771-3990100			5.8	5.4	6.2	↓	-0.8	3.7E-06	5.6E-03
chr11:403791-404223			4.1	3.4	4.6	↓	-1.2	1.8E-06	3.1E-03
chr11:50628440-50628845			2.9	2.1	3.5	↓	-1.4	1.2E-04	6.3E-02
chr11:57133485-57133981			3.7	4.2	3.1	↑	1.1	1.5E-04	7.2E-02
chr11:6744428-6745865			5.5	5.8	5.2	↑	0.6	1.4E-04	7.2E-02
chr11:7042481-7042832			2.9	2.1	3.5	↓	-1.4	1.1E-04	6.3E-02
chr11:70761778-70762451			4.6	3.9	5.0	↓	-1.2	2.4E-04	9.9E-02
chr11:70827753-70828354			3.6	2.6	4.2	↓	-1.5	1.4E-05	1.4E-02
chr11:7097151-7097597			3.6	2.7	4.2	↓	-1.5	6.9E-07	1.9E-03
chr11:71730021-71730516	ENSSSCG00000023598		3.3	2.0	3.9	↓	-1.9	1.2E-06	2.9E-03
chr11:7215446-7216213	ENSSSCG00000028065		5.0	4.4	5.5	↓	-1.1	3.3E-07	1.5E-03
chr11:74261505-74262160			3.6	2.6	4.2	↓	-1.6	1.3E-07	9.4E-04
chr11:74627947-74628487			3.2	3.8	2.4	↑	1.3	2.6E-04	1.0E-01
chr11:74641802-74642533	ENSSSCG00000009512		5.3	5.8	4.7	↑	1.1	8.5E-05	5.9E-02
chr11:74912602-74912990			3.4	3.9	2.6	↑	1.3	1.2E-04	6.3E-02
chr11:75771395-75772178	ENSSSCG00000020438	5S_rRNA	5.3	5.6	4.9	↑	0.7	5.0E-05	4.1E-02
chr11:76505020-76506081	ENSSSCG00000029926	U6	5.8	5.5	6.0	↓	-0.5	1.7E-04	8.1E-02
chr11:76649016-76650253			5.5	5.8	5.2	↑	0.6	1.2E-04	6.3E-02
chr11:77379274-77379538			3.1	3.7	2.0	↑	1.7	5.7E-05	4.2E-02
chr11:79685997-79686309			3.6	4.2	2.5	↑	1.6	4.5E-07	1.6E-03
chr11:8042345-8042709			3.5	2.8	4.0	↓	-1.2	1.1E-04	6.3E-02
chr11:81252307-81254702			6.6	6.3	6.8	↓	-0.5	9.1E-06	1.1E-02
chr11:81566984-81567461			3.8	4.3	3.1	↑	1.2	5.7E-05	4.2E-02
chr11:81576425-81577428			5.7	5.2	6.1	↓	-1.0	3.5E-08	4.9E-04
chr11:81668110-81669580			5.0	5.4	4.5	↑	0.9	4.0E-06	5.6E-03
chr11:82152727-82153346			3.8	2.7	4.4	↓	-1.7	1.3E-05	1.4E-02
chr11:82415642-82416963			5.6	5.9	5.2	↑	0.8	1.9E-04	8.2E-02
chr11:82423688-82424298			4.8	5.2	4.1	↑	1.1	1.2E-04	6.3E-02
chr11:82441524-82442354			4.3	4.7	3.7	↑	1.0	2.6E-04	1.0E-01
chr11:82526244-82526852			4.5	5.1	3.4	↑	1.8	1.5E-06	2.9E-03
chr11:82871915-82872525			4.5	4.8	4.1	↑	0.8	2.5E-04	9.9E-02
chr11:83559517-83560749			5.4	5.7	5.1	↑	0.6	1.2E-04	6.3E-02
chr11:83758874-83759483			4.2	3.6	4.6	↓	-1.0	2.4E-04	9.9E-02
chr11:83966689-83967378			4.5	4.0	4.9	↓	-1.0	4.3E-05	3.7E-02
chr12:1672068-1672573			4.3	3.5	4.8	↓	-1.3	1.8E-08	1.2E-04
chr12:18978464-18978772	ENSSSCG00000027878; ENSSSCG00000026355	DBF4B	3.3	3.9	2.3	↑	1.7	3.8E-06	1.9E-02
chr12:2719801-2720267			3.5	2.6	4.0	↓	-1.3	3.9E-05	1.0E-01
chr12:34623482-34624216			5.2	4.8	5.6	↓	-0.8	7.3E-06	3.0E-02
chr12:4611837-4612794			5.6	5.2	6.0	↓	-0.7	2.4E-05	7.1E-02
chr12:56660684-56661111	ENSSSCG00000017992	MFSD6L	3.3	3.8	2.4	↑	1.4	2.1E-05	7.1E-02
chr12:7936001-7936598			4.9	3.9	5.4	↓	-1.5	1.2E-08	1.2E-04
chr12:7942124-7942517			4.0	2.6	4.6	↓	-2.0	2.3E-12	4.7E-08
chr13:134188139-134188614			3.3	2.4	3.9	↓	-1.4	2.1E-05	6.9E-02
chr13:194633660-194634097			3.2	3.8	2.3	↑	1.6	6.9E-06	4.0E-02
chr13:20622286-20623666			4.1	3.5	4.6	↓	-1.0	2.6E-05	6.9E-02

chr13:213684600-213685108			3.4	2.0	4.1	↓	-2.1	8.1E-10	1.5E-05
chr13:214224190-214225333			4.8	4.3	5.2	↓	-1.0	1.5E-06	1.3E-02
chr13:216099272-216099922	ENSSSCG00000021869	TFF1	4.6	5.0	4.1	↑	0.9	3.5E-05	7.9E-02
chr13:55933062-55934018			4.4	3.8	4.8	↓	-1.0	2.6E-05	6.9E-02
chr13:56224828-56225206			2.9	1.6	3.5	↓	-1.9	8.7E-06	4.0E-02
chr13:59403421-59404107			4.5	4.0	4.9	↓	-0.9	4.6E-05	9.4E-02
chr14:114236236-114236817			6.2	7.1	2.5	↑	4.6	5.9E-69	1.4E-64
chr14:144632182-144632484			2.8	3.5	1.6	↑	1.8	9.7E-06	4.6E-02
chr14:147537047-147537393			3.2	3.9	1.9	↑	2.0	1.1E-07	8.4E-04
chr14:21679863-21682086			5.9	6.5	4.6	↑	1.9	2.3E-36	2.7E-32
chr14:26664757-26665426			4.0	4.5	3.1	↑	1.5	4.2E-07	2.5E-03
chr14:99353243-99353922	ENSSSCG00000022256	C10orf10	3.7	4.2	2.9	↑	1.3	1.9E-05	7.4E-02
chr15:143502137-143503029			5.3	6.0	3.8	↑	2.1	5.1E-06	1.9E-02
chr15:146080485-146080949			4.1	3.1	4.7	↓	-1.6	5.7E-06	1.9E-02
chr15:152326700-152327720	ENSSSCG00000018694; ENSSSCG00000025228	TRAF3IP1	5.7	5.1	6.1	↓	-1.0	1.9E-05	4.1E-02
chr15:152646144-152647014			3.8	2.4	4.5	↓	-2.1	3.2E-08	4.2E-04
chr15:153002643-153003041			3.8	3.0	4.4	↓	-1.4	4.3E-06	1.9E-02
chr15:92130747-92131139			3.3	3.9	2.3	↑	1.5	1.0E-05	2.6E-02
chr17:12231714-12232821			5.1	4.5	5.5	↓	-1.0	7.0E-06	2.7E-02
chr17:12247609-12248624			5.7	6.2	4.9	↑	1.2	9.4E-09	1.2E-04
chr17:12300033-12300427			4.3	3.7	4.8	↓	-1.1	4.1E-06	2.7E-02
chr17:16308503-16309539			5.4	5.8	4.8	↑	1.0	8.1E-06	2.7E-02
chr18:15295506-15295905			3.6	4.1	2.6	↑	1.5	1.2E-05	5.6E-02
chr18:16457217-16457491			3.2	2.1	3.8	↓	-1.7	2.5E-06	1.8E-02
chr18:5948692-5949528			4.7	5.3	3.6	↑	1.7	1.0E-11	1.5E-07
chr2:11343029-11343410	ENSSSCG00000018435; ENSSSCG00000013125	U6; OSBP	3.0	1.8	3.6	↓	-1.8	1.4E-06	6.1E-03
chr2:118320803-118321254	ENSSSCG00000014192		3.8	4.2	3.1	↑	1.2	3.8E-05	8.5E-02
chr2:122991751-122992247			3.6	4.1	2.8	↑	1.4	6.3E-06	2.0E-02
chr2:150151849-150152343	ENSSSCG00000020485	U5	4.1	4.7	3.1	↑	1.5	4.9E-07	2.7E-03
chr2:15916373-15917043	ENSSSCG00000022684	U2	3.7	3.0	4.2	↓	-1.2	3.5E-05	8.5E-02
chr2:162483757-162484395			4.3	3.0	4.9	↓	-1.9	8.5E-08	6.3E-04
chr2:162518726-162519178			4.9	4.2	5.4	↓	-1.2	4.4E-09	4.9E-05
chr2:49426459-49426886			3.2	2.3	3.7	↓	-1.4	4.3E-05	8.7E-02
chr2:779749-780375	ENSSSCG00000012858		3.3	2.1	4.0	↓	-1.9	3.9E-06	1.4E-02
chr2:83982627-83983254			4.1	4.7	3.1	↑	1.6	9.1E-10	2.0E-05
chr2:954892-955756			5.4	5.0	5.7	↓	-0.7	2.3E-05	6.5E-02
chr3:101324422-101324801			3.2	2.3	3.7	↓	-1.4	2.4E-05	5.7E-02
chr3:10223508-10223890	ENSSSCG00000007700	HIP1	4.0	3.2	4.5	↓	-1.3	2.1E-05	5.7E-02
chr3:142907641-142908514			4.7	3.4	5.4	↓	-2.0	5.0E-08	1.4E-03
chr3:1895100-1895631			4.6	3.0	5.3	↓	-2.3	3.5E-05	7.5E-02
chr3:21173640-21174062			3.6	2.7	4.1	↓	-1.4	1.6E-05	5.6E-02
chr3:2645255-2646322			4.7	4.0	5.1	↓	-1.1	2.3E-07	3.1E-03
chr3:3046111-3046372			3.3	2.5	3.8	↓	-1.4	2.2E-05	5.7E-02
chr3:3484247-3484989			5.0	4.6	5.4	↓	-0.9	5.0E-06	2.8E-02
chr3:36705757-36706152			3.4	4.0	2.5	↑	1.6	1.3E-05	5.6E-02
chr3:41815529-41816007	ENSSSCG00000008021	TMEM204	3.5	4.1	2.6	↑	1.5	1.5E-05	5.6E-02
chr3:4393108-4393735	ENSSSCG00000007578	MMD2	5.1	4.6	5.5	↓	-0.8	4.9E-06	2.8E-02
chr3:48428204-48428847			4.3	3.6	4.8	↓	-1.2	4.9E-07	4.5E-03
chr3:51456689-51457182			3.3	2.4	3.9	↓	-1.4	2.5E-05	5.7E-02
chr4:114147375-114147825			3.8	4.4	3.0	↑	1.4	1.3E-06	9.7E-03
chr4:116214231-116214941			7.0	8.0	2.7	↑	5.2	1.6E-83	3.4E-79
chr4:36870434-36871011			4.1	4.6	3.4	↑	1.2	6.4E-06	2.8E-02
chr4:38403864-38404359	ENSSSCG00000006060	GRHL2	3.7	2.7	4.4	↓	-1.7	2.1E-07	2.2E-03
chr4:44516563-44517096			4.0	4.5	3.1	↑	1.4	4.5E-06	2.5E-02
chr5:108076917-108077289			3.3	3.8	2.4	↑	1.4	5.6E-05	7.9E-02
chr5:14480164-14480504			3.5	2.5	4.1	↓	-1.5	8.3E-06	2.0E-02

chr5:18127041-18128091	ENSSSCG00000020838		5.3	5.7	4.7	↑	1.0	6.6E-05	8.6E-02
chr5:20665512-20666141	ENSSSCG00000026482		3.6	2.7	4.2	↓	-1.6	6.0E-06	1.8E-02
chr5:21941009-21941555	ENSSSCG00000000333		3.4	4.0	2.3	↑	1.7	1.2E-06	1.0E-02
chr5:21976137-21977026			4.0	4.5	3.3	↑	1.2	6.2E-06	1.8E-02
chr5:2422213-2422648			3.3	2.0	4.0	↓	-2.0	3.3E-08	5.7E-04
chr5:24339808-24340109	ENSSSCG00000023150	LPR1	2.9	1.8	3.5	↓	-1.7	3.6E-05	6.8E-02
chr5:32707589-32708058	ENSSSCG00000000468		3.7	3.0	4.1	↓	-1.1	1.0E-04	9.5E-02
chr5:34272781-34273107			3.3	2.4	3.8	↓	-1.4	8.9E-05	9.5E-02
chr5:5279616-5280450	ENSSSCG00000000077	ADSL	4.5	4.9	4.0	↑	0.9	1.1E-04	9.5E-02
chr5:6243400-6243785			4.4	4.8	3.8	↑	1.0	4.8E-05	7.4E-02
chr5:63332670-63333050			3.0	3.6	1.9	↑	1.7	9.5E-05	9.5E-02
chr5:67197169-67197686	ENSSSCG00000000714	NTF3	4.6	5.0	3.9	↑	1.1	1.6E-05	3.5E-02
chr5:67329409-67329813			3.3	2.2	3.9	↓	-1.7	1.9E-06	1.1E-02
chr5:72220617-72220987			3.1	2.0	3.6	↓	-1.6	8.7E-05	9.5E-02
chr5:76638490-76639136	ENSSSCG00000027887	7SK	4.4	3.7	4.9	↓	-1.2	6.4E-06	1.8E-02
chr5:83819211-83819788			2.9	3.5	2.0	↑	1.4	9.3E-05	9.5E-02
chr5:94292599-94292907			3.1	2.1	3.6	↓	-1.5	4.2E-05	7.2E-02
chr6:1278642-1280520			7.1	6.9	7.3	↓	-0.4	9.1E-06	5.0E-02
chr6:153493717-153494126			4.0	3.2	4.4	↓	-1.2	6.9E-06	4.5E-02
chr6:154924194-154924782			3.8	4.3	3.1	↑	1.3	5.1E-06	4.2E-02
chr6:27387592-27387875	ENSSSCG00000002826		3.5	2.3	4.1	↓	-1.8	2.2E-06	2.4E-02
chr6:28090081-28090820			4.2	3.6	4.6	↓	-1.0	2.7E-05	9.8E-02
chr6:32822972-32823382			3.1	2.1	3.7	↓	-1.7	1.3E-05	5.9E-02
chr6:33915403-33915732			3.7	4.4	2.5	↑	1.9	1.6E-08	5.1E-04
chr6:8739515-8739831			3.3	2.1	3.9	↓	-1.9	4.2E-07	6.9E-03
chr6:8762743-8763598			5.2	4.8	5.5	↓	-0.7	1.9E-05	7.6E-02
chr7:120933149-120933791			5.0	4.4	5.4	↓	-0.9	1.7E-06	8.5E-03
chr7:12975780-12976203			3.5	1.9	4.3	↓	-2.3	7.2E-10	1.7E-05
chr7:28199625-28200065	ENSSSCG00000030901	DUROC-BTNL6	3.7	2.8	4.2	↓	-1.4	2.6E-05	7.3E-02
chr7:3905107-3905578			4.8	5.1	4.3	↑	0.8	2.2E-05	7.3E-02
chr7:47784397-47784676			3.3	1.9	3.9	↓	-2.0	1.8E-06	8.5E-03
chr7:62288418-62288871			3.5	2.4	4.1	↓	-1.6	4.7E-07	3.8E-03
chr7:74281422-74281936			3.9	2.8	4.6	↓	-1.8	3.3E-09	4.0E-05
chr7:78088004-78088874			3.9	4.4	3.2	↑	1.2	2.7E-05	7.3E-02
chr7:86536766-86537172			3.2	2.0	3.8	↓	-1.8	2.3E-06	9.3E-03
chr8:132617613-132618070			2.9	1.8	3.6	↓	-1.7	1.8E-05	5.7E-02
chr8:141826630-141827184			3.5	2.7	4.1	↓	-1.3	4.2E-05	8.1E-02
chr8:145954939-145956030			4.3	4.9	3.2	↑	1.7	1.2E-06	1.0E-02
chr8:147203121-147204330	ENSSSCG00000020131	U6	5.5	5.8	5.1	↑	0.7	1.8E-05	5.7E-02
chr8:19393613-19394894	ENSSSCG00000025315		4.1	3.4	4.7	↓	-1.3	1.3E-06	1.0E-02
chr8:7647185-7647766			3.3	2.4	3.8	↓	-1.4	3.9E-05	8.1E-02
chr8:77151688-77152241			4.8	5.1	4.2	↑	0.9	2.2E-05	5.7E-02
chr8:80678244-80678596			3.4	2.4	4.0	↓	-1.6	2.1E-05	5.7E-02
chr9:1098557-1098873			3.1	3.8	1.9	↑	1.9	5.6E-07	1.2E-02
chr9:1175440-1176323			4.1	3.0	4.7	↓	-1.7	3.1E-05	6.1E-02
chr9:13142767-13143248			3.7	3.0	4.2	↓	-1.3	5.4E-05	8.1E-02
chr9:132028863-132029285			3.1	2.1	3.7	↓	-1.6	3.4E-05	6.1E-02
chr9:142174523-142175405			4.3	4.8	3.7	↑	1.1	5.1E-05	8.1E-02
chr9:145033265-145033673			3.2	2.2	3.8	↓	-1.7	2.3E-05	6.1E-02
chr9:146390780-146392239	ENSSSCG00000015611		6.7	6.4	6.9	↓	-0.5	7.6E-06	5.4E-02
chr9:146398660-146399215	ENSSSCG00000015611		4.4	3.7	4.8	↓	-1.1	3.9E-06	4.1E-02
chr9:146499873-146500486	ENSSSCG00000028125; ENSSSCG00000015617	5S_rRNA; G0S2	3.7	2.8	4.2	↓	-1.4	3.4E-05	6.1E-02
chr9:149010581-149011352			4.4	4.8	3.7	↑	1.1	1.9E-05	6.1E-02
chr9:151531815-151532259			2.9	1.9	3.5	↓	-1.6	2.5E-05	6.1E-02
chr9:2589644-2590852			4.8	4.0	5.3	↓	-1.4	2.4E-05	6.1E-02
chr9:43506912-43507184			3.0	3.6	2.0	↑	1.6	2.8E-05	6.1E-02
chr9:57872485-57873841			4.5	5.0	3.8	↑	1.2	1.7E-05	6.1E-02
