# Supplementary material for: Arterial endothelial methylome: differential DNA methylation in athero-susceptible disturbed flow regions in vivo
Source: BMC Genomics. 2015 Jul 7;16:506. doi: 10.1186/s12864-015-1656-4 (PMC4492093; doi:10.1186/s12864-015-1656-4)
Supplement: Additional file 5: Table S2b. — Male-AA vs Female-AA DMR (FDR < 0.1). [file 12864_2015_1656_MOESM5_ESM.pdf]

Supplementary Table 2b. M-AA vs F-AA DMR (FDR<0.1)

DMR	Close Emsembl Genes	Human Homolog Gene	Conc	Conc_ M	Conc_ F		Fold, log2 (M/F)	p-value	FDR
chr10:17454148-17454499			3.5	2.5	4.1	↓	-1.6	9.3E-06	6.1E-02
chr10:221144-221681			4.0	4.5	3.3	↑	1.2	1.5E-05	7.3E-02
chr10:58058325-58058772			4.0	3.1	4.5	↓	-1.4	7.0E-07	1.4E-02
chr10:60343312-60343638			3.8	4.4	2.5	↑	2.0	1.9E-06	1.9E-02
chr11:25393502-25394275			3.8	3.0	4.4	↓	-1.4	1.3E-06	3.5E-03
chr11:25403726-25404312			3.2	2.0	3.8	↓	-1.8	4.6E-06	9.1E-03
chr11:3144847-3145100			2.9	1.9	3.5	↓	-1.6	5.7E-05	6.4E-02
chr11:3441950-3442496			3.4	3.9	2.7	↑	1.3	1.0E-04	8.6E-02
chr11:5155092-5155566			3.5	2.7	4.0	↓	-1.3	7.4E-05	7.3E-02
chr11:59146749-59147040			3.2	2.2	3.8	↓	-1.6	5.4E-05	6.4E-02
chr11:71154839-71155313			3.5	1.7	4.3	↓	-2.5	2.0E-08	3.1E-04
chr11:74429343-74430107			3.8	3.0	4.3	↓	-1.3	6.1E-05	6.4E-02
chr11:74829071-74829672			4.4	4.8	3.9	↑	0.9	8.1E-05	7.5E-02
chr11:81332880-81333422			4.6	5.0	4.0	↑	0.9	3.2E-05	4.6E-02
chr11:81576451-81577438			5.8	5.4	6.1	↓	-0.7	1.2E-04	9.4E-02
chr11:81699641-81699962			3.4	3.9	2.6	↑	1.3	8.8E-05	7.7E-02
chr11:82068819-82069302			4.8	5.2	4.2	↑	0.9	1.8E-05	3.2E-02
chr11:82141341-82141612			2.8	1.3	3.5	↓	-2.2	5.8E-07	2.3E-03
chr11:82217764-82218478	ENSSSCG00000009537	FAM155A	3.8	4.5	2.4	↑	2.1	2.5E-06	5.6E-03
chr11:82423796-82424351			4.7	5.3	3.8	↑	1.5	5.4E-07	2.3E-03
chr11:82526273-82526624			3.9	4.6	2.6	↑	1.9	3.6E-07	2.3E-03
chr11:82528350-82529340			5.4	5.7	4.9	↑	0.8	7.9E-07	2.5E-03
chr11:82531122-82531428			3.1	2.2	3.7	↓	-1.5	4.4E-05	5.8E-02
chr11:83105607-83106118			3.7	4.2	2.9	↑	1.3	2.9E-05	4.5E-02
chr12:3750542-3750856			3.4	2.3	4.0	↓	-1.6	4.4E-06	3.2E-02
chr12:57044581-57045239			4.8	5.2	4.2	↑	1.1	1.3E-07	1.4E-03
chr12:7936005-7936548			4.6	3.4	5.2	↓	-1.7	3.3E-12	7.2E-08
chr13:142601491-142601880			3.8	4.3	3.0	↑	1.3	9.6E-06	6.9E-02
chr13:213684551-213685031			3.4	2.1	4.1	↓	-2.0	7.6E-08	1.6E-03
chr13:36988236-36988590	ENSSSCG00000011422; ENSSSCG00000011419		2.8	1.5	3.5	↓	-2.0	7.7E-06	6.9E-02
chr14:114236258-114236768			6.2	7.1	3.0	↑	4.1	1.6E-79	4.3E-75
chr14:120817949-120818348			2.8	3.5	1.4	↑	2.0	8.9E-06	6.0E-02
chr14:21680664-21682103			5.3	5.8	4.5	↑	1.3	9.8E-12	1.3E-07
chr14:23562327-23562600			3.2	1.8	3.9	↓	-2.1	1.0E-07	9.2E-04
chr15:143502213-143503063			5.2	5.9	3.7	↑	2.2	4.6E-06	1.7E-02
chr15:146079987-146081418			5.0	4.5	5.4	↓	-0.9	2.9E-06	1.5E-02
chr15:152326613-152327679	ENSSSCG00000018694; ENSSSCG00000025228	TRAF3IP1	5.7	5.1	6.1	↓	-1.0	1.2E-05	3.6E-02
chr15:36158422-36159294	ENSSSCG00000015742	TMEM177	4.0	4.6	3.1	↑	1.4	2.3E-06	1.5E-02
chr15:92659464-92659882			4.1	3.2	4.6	↓	-1.4	1.1E-07	1.6E-03
chr17:12247638-12248606			5.6	6.1	4.7	↑	1.4	3.8E-18	5.7E-14
chr17:13394687-13394972			3.4	3.9	2.5	↑	1.4	1.9E-05	9.4E-02
chr17:9918703-9919284			3.6	2.6	4.2	↓	-1.6	1.4E-05	9.4E-02
chr18:15295476-15295879			3.7	4.3	2.7	↑	1.6	1.2E-06	9.3E-03
chr18:46378573-46378995			3.3	2.2	4.0	↓	-1.7	3.5E-06	1.2E-02
chr18:56028165-56028716			4.0	3.2	4.5	↓	-1.3	3.1E-06	1.2E-02
chr18:57403083-57403702			3.8	2.8	4.4	↓	-1.5	1.7E-06	9.3E-03
chr18:57588741-57589554			4.2	3.6	4.7	↓	-1.1	2.0E-05	5.5E-02
chr18:5948850-5949516			4.4	5.0	3.4	↑	1.7	6.0E-08	9.8E-04
chr2:158290250-158290666	ENSSSCG00000014445		3.1	3.8	1.7	↑	2.1	2.5E-07	3.5E-03
chr2:18074-19445			6.3	6.5	5.9	↑	0.6	2.8E-07	3.5E-03
chr3:1352629-1352906			3.1	3.7	1.8	↑	1.9	5.0E-07	7.8E-03
chr3:1355858-1356151			2.8	3.5	1.6	↑	1.9	3.3E-06	2.0E-02

chr3:139836732-139837125	ENSSSCG00000024285	DLG4	4.0	4.4	3.3	↑	1.2	1.1E-05	4.3E-02
chr3:24435795-24436648			4.6	4.0	5.0	↓	-1.0	7.1E-06	3.1E-02
chr3:36871089-36871382			3.4	4.1	2.0	↑	2.1	1.1E-09	3.3E-05
chr3:48428171-48428915			4.3	3.5	4.8	↓	-1.3	1.8E-06	1.4E-02
chr3:54694697-54695003			3.8	4.3	3.1	↑	1.3	3.2E-05	9.9E-02
chr3:55513002-55513473			4.0	3.2	4.6	↓	-1.3	1.4E-06	1.4E-02
chr3:56000336-56000742			3.7	4.2	2.9	↑	1.3	1.6E-05	5.5E-02
chr3:72888096-72888362			3.1	3.7	2.0	↑	1.7	6.4E-06	3.1E-02
chr4:114334801-114335150			4.0	4.5	3.4	↑	1.1	1.6E-05	9.8E-02
chr4:116214260-116214917			7.0	8.0	3.2	↑	4.7	3.7E-88	9.1E-84
chr4:42310963-42311625			3.9	3.1	4.4	↓	-1.3	1.4E-05	9.8E-02
chr4:42848379-42849055			4.2	4.7	3.4	↑	1.3	4.8E-06	5.9E-02
chr5:2027909-2028675			4.6	5.0	4.1	↑	1.0	1.3E-05	4.8E-02
chr5:20665705-20666161	ENSSSCG00000026482		3.6	2.8	4.1	↓	-1.3	3.0E-05	9.7E-02
chr5:21589985-21590443			2.8	3.5	1.4	↑	2.1	1.4E-06	1.4E-02
chr5:34074807-34076488	ENSSSCG00000019414	U1	5.4	4.9	5.8	↓	-0.9	7.8E-07	1.4E-02
chr5:67329424-67329790			3.0	1.8	3.7	↓	-1.8	1.1E-05	4.8E-02
chr5:89657816-89658203			3.7	2.9	4.2	↓	-1.4	8.2E-06	4.8E-02
chr6:30368331-30368557			3.2	3.8	2.1	↑	1.7	7.0E-06	8.6E-02
chr6:33915416-33915717			3.8	4.3	2.9	↑	1.3	2.9E-06	5.3E-02
chr6:34366891-34367163			2.7	3.4	1.5	↑	1.9	1.1E-05	9.7E-02
chr6:8798684-8799000			3.3	1.7	4.0	↓	-2.3	4.8E-09	1.8E-04
chr7:24018404-24019537	ENSSSCG00000001203	ZNF193	4.6	3.8	5.2	↓	-1.4	3.7E-08	5.1E-04
chr7:47784400-47784675			3.4	2.4	4.0	↓	-1.6	4.3E-06	2.9E-02
chr7:4996785-4997110			3.4	4.1	2.1	↑	1.9	2.4E-08	5.1E-04
chr7:74318862-74319526			4.1	3.3	4.7	↓	-1.4	2.8E-07	2.6E-03
chr7:74327191-74327819			3.8	2.9	4.4	↓	-1.5	6.4E-06	3.5E-02
chr8:10415714-10416338			4.6	5.0	4.1	↑	0.9	3.3E-05	9.9E-02
chr8:109402612-109403065	ENSSSCG00000009093	BBS7	3.1	3.8	2.0	↑	1.7	5.1E-06	9.4E-02
chr8:145501645-145503243			6.2	6.5	5.9	↑	0.5	1.9E-05	9.9E-02
chr8:145967200-145968110			4.5	4.9	3.8	↑	1.1	1.8E-05	9.9E-02
chr8:77151721-77152250			4.7	5.1	4.2	↑	0.9	2.2E-05	9.9E-02
chr8:92568983-92569378			4.1	4.6	3.5	↑	1.1	3.1E-05	9.9E-02
chr9:146398689-146399163	ENSSSCG00000015611		4.3	3.5	4.8	↓	-1.2	3.1E-06	3.7E-02
chr9:43233727-43234688			4.8	4.0	5.3	↓	-1.2	4.6E-08	1.1E-03
