# Supplementary material for: Arterial endothelial methylome: differential DNA methylation in athero-susceptible disturbed flow regions in vivo
Source: BMC Genomics. 2015 Jul 7;16:506. doi: 10.1186/s12864-015-1656-4 (PMC4492093; doi:10.1186/s12864-015-1656-4)
Supplement: Additional file 6: Table S3. — Primers for qPCR analysis. [file 12864_2015_1656_MOESM6_ESM.pdf]

Supplementary Table 3. Primers for qPCR analysis

	Swine Genes	Primers	Amplicon Size (bp)
1	ARHGAP25	F. CAGGAGCTGCGAAAGGAAATAC	145
		R. AGGCTGCAGACTTCTTCTTCTC	
2	HOXA1	F.CCCTGGCCACGTATAATAACTC	101
		R.AAATGTCTGCGCTGGAGAAG	
3	HOXA2	F.CCCTGTCGCTGATACATTTC	93
		R.GGAATGGTCTGCTCAAAAGG	
4	HOXA3	F.ACCGTGGCCAAACAAATC	110
		R.GGTGGGCTCTTGTCACCTG	
5	HOXA4	F.ATGACCATGAGCTCGTTTTTG	80
		R.CTGTGCTGCGCGTACTCC	
6	HOXA5	F. CAGATCTACCCCTGGATGCG	134
		R.TAGCGGTTGAAGTGGAACTCC	
7	HOXA6	F.ATGCAGCGGATGAACTCC	122
		R.AGGTAGCGGTTGAAGTGGAAC	
8	HOXA7	F.GCCAATTTCCGCATTTATCC	114
		R.GTTGAAGTGGAACTCCTTCTCC	
9	HOXA10	F.GAGAAGCCAGCTCTCCCTTG	130
		R.GTCTGGTGCTTGGTGTAGGG	
10	HOXA11	F.CTTCGCGCCCAATGACATAC	84
		R.TTGACTTGACGGTCGGTGAG	
11	HOXA13	F.CCAAATGTACTGCCCCAAAG	126
		R.TAAGGGACGCGCTTCTTTC	
12	HOXB5	F. GATGAGGAAGCTTCACATCAGC	115
		R. GCGGTTGAAGTGGAACTCTTTC	
13	HOXD4	F.TCCGTGCGAGGAATATTTGC	133
		R.GAAGGTCTGCTCACTAAAGTCG	
14	UBB	F.TGAGGGGTGGCTGCTAATTC	73
		R.TGGCTAGAGTGCAGAGTAATGC	
15	PECAM1	F.CCTCGCCCATTTCCTACCAACTTT	237
		R.CAGACTCCACCTCCTCGCTCAG	
16	GAPDH	F.CCTGTGACTTCAACAGTGACAC	123
		R. CCCTGTTGCTGTAGCCAAATTC	
