# Supplementary material for: Arterial endothelial methylome: differential DNA methylation in athero-susceptible disturbed flow regions in vivo
Source: BMC Genomics. 2015 Jul 7;16:506. doi: 10.1186/s12864-015-1656-4 (PMC4492093; doi:10.1186/s12864-015-1656-4)
Supplement: Additional file 7: Table S4. — Primers for MSP analysis of swine gene promoter methylation. [file 12864_2015_1656_MOESM7_ESM.pdf]

Supplementary Table 4. Primers for MSP analysis of swine gene promoter methylation.

	Swine Genes	Methylation Primers	Genomic DNA Sequence
1	ARHGAP25, (-27/+104)	F. GTTTAGTTTGGTTGGCGC<br>R. ACGACCATCTACTCACCGAT	GCCCAGCCTGGCTGGCGCGCAGCGCTCCTTGGCCCTTCG<br>CTGCCTACTGCTCACGTCCCCGGTCAGCTCGCTTGTCTGT<br>CCCTCTCCACAGCTCGGTCAAGGAGCGTGATGACCGGTGA<br>GCAGATGGCCGC
2	ATF4, (-7072/-6918)	F. TTTTAGGAATCGAATTTGAGTC<br>R. AATCGTATAACCTCTATCGCC	TTTCAGGAATCGAACCTGAGCCGCAGCAGTGACAACACCG<br>GATCCTTAACCTGCTGATCCAGCAGGGAAGTCCTCAAATG<br>CCGTCTTTGGGTTGTGTTGATGTGTCTGTCCCTTCTCCAGA<br>CACTGAGCTCCTTGGCGACAGAGGCCACACGACC
3	HOXA5, (-1694/-1479)	F. GTTTTCGGTCGTTTTTGTTC<br>R. TCTTCCACTTCATACGACGA	GCTCCCGGCCGTTTCTGCCCGCAGGTGCTGTGTACGGGA<br>GTCACGGGCGCCGAGGCCGCCAGACCTACACCCGCTACC<br>AGACGCTGGAGCTGGAGAAGGAGTTCCACTTCAACCGCTA<br>CCTGACGCGGCGCCGCCGCATCGAGATCGCCAACGCGCT<br>CTGCCTCACCGAGCGCCAGATCAAGATCTGGTTCCAGAAC<br>CGCCGCATGAAGTGGAAGA
4	HOXB5, (-1238/-1082)	F. TACGTGGTTTGATTTTCGTC<br>R. CTACCGACAACTTCGCATT	CACGTGGCCTGATCTCCGCCGGCCTTGGCCCCGCTCATG<br>GCCTAAATTGGGGCCACCGGGTATCTATTTCTTTGCCTGAA<br>GCTAAAGGCTCCAGGCACCCCTTTCTGCCTCAAGCAGTAG<br>CTCAGCATCAAGTAGGGGAATGCGAAGCTGCCGGCAG
5	HOXD4, (+173/+345)	F.GTGAGTAGATTTTCGGAGGC<br>R.CTAACTACAAACCCGAACGC	GTGAGCAGACCTTCGGAGGCGGCGGCCCCGGGCCAGGCT<br>CGGCGCTGCCCACGCGGAGTCACGGGCCAGAACCCAGCG<br>GCCCCGGCGGCCACTACGGTGCCCCGGGAGAGCCGTGCC<br>CGGCACCCCCGGCGCCCCCGCCCGCACCGCTGCCCGGC<br>GCCCGGGCCTGCAGCCAG
6	mir10a, (-219/-45)	F. CGTGTTTTTTTGCGGAATC,<br>R. TCGCGTTCTTCCTTTAAAAT	CGTGTCCCTTTGCGGAACCGGCTGCTTCTGTTTCTAAGAGA<br>AGAGAAAGAGAGTTGAAACCCTTGTAATCCCAAGAATGGAC<br>CCACAGCGCCTTTTCCTGTTCCCAGAGCGCAAACCTAGAA<br>CAAAACGAAATAAAACCAAAGCACTCAAACCACACCCCAAA<br>GGAAGAACGCGA
7	UBB, (-432/-328)	F.GGGAAGGTGGGAAAGAGGTAG<br>R. AGCATTGAAATTCCCGTTGGG	GGGAAGGTGGGAAAGAGGTAGTATAGCCGGGCTTCTGGA<br>AACCTTGTTCCGAATCCTGACGTGGAGAGGGTGTCGCACC<br>CACCTCCCAACGGGAATTTCAATGCT
